# Chromatin Landscape Distinguishes the Genomic Loci of Hundreds of Androgen-Receptor-Associated LincRNAs From the Loci of Non-associated LincRNAs

**DOI:** 10.3389/fgene.2018.00132

**Published:** 2018-04-25

**Authors:** Lucas F. daSilva, Felipe C. Beckedorff, Ana C. Ayupe, Murilo S. Amaral, Vinícius Mesel, Alexandre Videira, Eduardo M. Reis, João C. Setubal, Sergio Verjovski-Almeida

**Affiliations:** ^1^Departamento de Bioquímica, Instituto de Química, Universidade de São Paulo, São Paulo, Brazil; ^2^Laboratório de Expressão Gênica em Eucariotos, Instituto Butantan, São Paulo, Brazil; ^3^Biocomplexity Institute of Virginia Tech, Blacksburg, VA, United States

**Keywords:** long intergenic non-coding RNAs, androgen receptor, androgen receptor associated lincRNAs, genome-wide profiling, epigenetic marks, LNCaP prostate cancer cell line, machine learning, random forest algorithm

## Abstract

Cell signaling events triggered by androgen hormone in prostate cells is dependent on activation of the androgen receptor (AR) transcription factor. Androgen hormone binding to AR promotes its displacement from the cytoplasm to the nucleus and AR binding to DNA motifs, thus inducing activatory and inhibitory transcriptional programs through a complex regulatory mechanism not yet fully understood. In this work, we performed RNA-seq deep-sequencing of LNCaP prostate cancer cells and found over 7000 expressed long intergenic non-coding RNAs (lincRNAs), of which ∼4000 are novel lincRNAs, and 258 lincRNAs have their expression activated by androgen. Immunoprecipitation of AR, followed by large-scale sequencing of co-immunoprecipitated RNAs (RIP-Seq) has identified in the LNCaP cell line a total of 619 lincRNAs that were significantly enriched (FDR < 10%, DESeq2) in the anti-Androgen Receptor (antiAR) fraction in relation to the control fraction (non-specific IgG), and we named them Androgen-Receptor-Associated lincRNAs (ARA-lincRNAs). A genome-wide analysis showed that protein-coding gene neighbors to ARA-lincRNAs had a significantly higher androgen-induced change in expression than protein-coding genes neighboring lincRNAs not associated to AR. To find relevant epigenetic signatures enriched at the ARA-lincRNAs’ transcription start sites (TSSs) we used a machine learning approach and identified that the ARA-lincRNA genomic loci in LNCaP cells are significantly enriched with epigenetic marks that are characteristic of *in cis* enhancer RNA regulators, and that the H3K27ac mark of active enhancers is conspicuously enriched at the TSS of ARA-lincRNAs adjacent to androgen-activated protein-coding genes. In addition, LNCaP topologically associating domains (TADs) that comprise chromatin regions with ARA-lincRNAs exhibit transcription factor contents, epigenetic marks and gene transcriptional activities that are significantly different from TADs not containing ARA-lincRNAs. This work highlights the possible involvement of hundreds of lincRNAs working in synergy with the AR on the genome-wide androgen-induced gene regulatory program in prostate cells.

## Introduction

The AR transcription factor mediates the effect of androgen hormone and modulates the gene transcriptional program in prostate cells. When not interacting with androgen the AR is maintained in the cytoplasmic cell portion associated with a protein scaffold (Ni et al., 2013) and after androgen interaction a tridimensional change in the AR structure occurs that allows the AR migration to the nucleus (Ni et al., 2013). In the nucleus, AR interacts with co-activators, co-repressors and the chromatin-remodeling complex (Ni et al., 2013) binds to genomic DNA target sites and promotes a highly regulated androgen-dependent gene activation program.

There is ample evidence that long non-coding RNAs (lncRNAs) can interact with a number of proteins and regulate gene expression (Khalil and Rinn, 2011). LncRNAs are transcripts longer than >200 nucleotides that do not code for proteins and in the human genome they are transcribed from intronic and intergenic regions (Kapranov et al., 2010; Derrien et al., 2012). They exhibit features similar to those of mRNAs, such as 5′ cap, poly-A tail and Pol II enrichment at their TSS (Guttman et al., 2009; Kapranov et al., 2010; Conley and Jordan, 2012). LncRNAs are known to bind to transcription factors and modulate their downstream gene activating function; for example, *NRCP* lncRNA strongly binds to STAT1 transcription factor and *NRCP* silencing decreases the interaction of STA1 with RNA polymerase II (Rupaimoole et al., 2015), causing a decreased expression of genes in the glycolysis pathway. Also, *EVF2* lncRNA binds to DLX2 transcription factor and increases its transcriptional activity (Feng et al., 2006). The above examples have measured the association of a transcription factor with a given single lncRNA, and large-scale assays such as native RIP-seq (Zhao et al., 2010) and CLIP-seq (Licatalosi et al., 2008) have not been used with transcription factors. In fact, RIP-seq has been applied with typical RNA binding proteins (RBPs) such as ELAVL1 (HuR) and PTBP1 (HNRNP-1), leading to the observation that in general a given RBP associates with hundreds of mRNAs and/or lncRNAs that may regulate their functions (Cook et al., 2015; Ferre et al., 2016). Nevertheless, little is known about the ability of transcription factors to associate with a diverse number of lncRNAs.

An important finding that arises from the analysis of AR binding to the genomic DNA target sites across the human genome and from the genome-wide gene expression regulation by androgen is that AR and its coactivators are preferentially enriched at genomic regions of histone H3 lysine 4 di-methylation (H3K4me2) (Taslim et al., 2012), an active histone mark present on both enhancer and promoter regions. In addition, H3K4me1 and H3K27ac, known to be associated with enhancer sites (Creyghton et al., 2010; Marques et al., 2013) are colocalized with AR genomic binding sites in LNCaP prostate cancer cells (He et al., 2014). Another feature of AR transcriptional activity is that it is dependent on the chromatin structure organization around blocks delimited by CTCF enhancer insulator (Taslim et al., 2012). CTCF enhancer insulator is known to participate in the definition of the structural organization of chromatin into what is known as TADs (Bonev and Cavalli, 2016), and TADs have been well characterized in LNCaP prostate cancer cells (Taberlay et al., 2016). TADs boundaries are conserved between different organisms and cell lines (Dixon et al., 2012; Le et al., 2013) and boundaries isolate the chromatin within ∼1 Mb-sized regions containing an elevated internal number of chromatin contacts (Rao et al., 2014). A large part of these intra-TAD contacts occurs between enhancers and promoters (Smith et al., 2016). Either TAD structure disruption or *de novo* TADs formation events can contribute to prostate cancer and other diseases (Franke et al., 2016; Luo et al., 2017).

It is known that the histone enhancer marks are enriched at the promoter regions of lncRNAs in human cells, which is in line with the local *cis*-regulatory enhancer function of lncRNAs on the neighbor genes (Orom et al., 2010; Marques et al., 2013; Ilott et al., 2014). In mouse embryonic fibroblasts, it has been shown that CBP, a transcription co-activator whose binding is a signature of enhancers, interacts with a wide range of enhancer RNAs (eRNAs) and stimulates histone acetylation mediated by CBP such as H3K27ac; this contributes to the unique chromatin structure at active enhancers, which, in turn, is required for regulation of target genes (Bose et al., 2017). We have therefore hypothesized that the AR transcription factor might be associated to a diverse range of lncRNAs and that the transcription loci of these AR-associated lincRNAs might have a specific epigenetic signature eventually related to an enhancer-like function. In the present work, we performed deep-coverage RNA-seq in LNCaP prostate cancer cells to identify their lncRNAs complement. In parallel, we performed native RIP-seq (Zhao et al., 2010) with antiAR antibody and a non-specific IgG control, and identified hundreds of lncRNAs associated to AR. We investigated the chromatin marks present in the vicinity of the transcription loci of these AR-associated lincRNAs (ARA-lincRNAs), compared with the marks at the loci of non-significantly AR-associated lincRNAs (NonA-lincRNAs), and using a machine learning approach (Pedregosa et al., 2012) we found a significantly enriched set of marks that characterize an epigenetic signature of ARA-lincRNA loci. Protein-coding genes in the vicinity of ARA-lincRNAs also showed a particular epigenetic signature, distinct from that of protein-coding genes in the vicinity of NonA-lincRNAs.

## Materials and Methods

### Cell Lines

LNCaP cells were obtained from the ATCC and grown as recommended in media supplemented with 10% fetal bovine serum and 1% penicillin/streptomycin (Gibco). Three to four biological replicates were obtained (*n* = 3–4). Cells were cultured for 24 h in androgen-deprived RPMI 1640 (Sigma) with 10% (w/v) charcoal-stripped fetal bovine serum (Sigma) as described in Louro et al. (2007). After 24 h, cells were placed in fresh RPMI 1640 with 10% (w/v) charcoal-stripped FBS and either 0.1 or 1 nM of the synthetic androgen R1881 (Sigma) or the equivalent volume of vehicle (ethanol) was added, followed by incubation for different times, as detailed below. R1881, a non-metabolized synthetic analog of testosterone was used to minimize metabolic degradation during incubation (Decker et al., 2012).

### Reverse Transcription and Quantitative Real-Time PCR (RT-qPCR)

Oligo-dT-primed reverse transcription (RT) was performed using 1 μg of total RNA according to the Super Script III kit protocol (Invitrogen). The relative levels of the transcripts were determined through quantitative real-time PCR (qPCR) (primers are shown in Supplementary Table S1) with Power SYBR Green (Applied Biosystems) using the 7500 Real Time PCR System (Applied Biosystems). The levels of these transcripts were normalized relative to the percentage levels of input.

### Native RNA-Binding Protein Immunoprecipitation (RIP)

A native RIP (Zhao et al., 2010) was performed using the Magna RIP RNA-Binding Protein Immunoprecipitation Kit (Millipore) following the exact instructions of the manufacturer. To increase reproducibility, for each replicate we used exactly 2.0 × 10^7^ LNCaP cells treated for 6 h with 0.1 nM R1881 (Sigma) or with vehicle (ethanol) and 5 μg of each antibody. The following antibodies were used from Millipore: Normal Rabbit IgG (12-370) and Anti-Androgen Receptor (06-680). The RNAs were extracted using Trizol, treated with TURBO DNase (Ambion) at 37°C for 30 min, purified using an RNeasy Micro Kit (Qiagen) and quantified with RiboGreen (Invitrogen). RIP-Seq was performed in biological duplicates (*n* = 2), RIP-qPCR in biological triplicates (*n* = 3); co-precipitated RNAs were detected either by high-throughput sequencing as described below or by RT-qPCR with two to three technical replicates for each biological replicate (primers in Supplementary Table S1).

### RNA Extraction Preparation for Next-Generation Sequencing

For RNA-seq experiments poly(A)^+^ RNA was extracted from LNCaP cells treated for 24 h with 1 nM of the synthetic androgen R1881 (Sigma) (*n* = 2) or with an equivalent volume of vehicle (ethanol) (*n* = 2) using FastTrack MAG Maxi mRNA Isolation Kit (Invitrogen) as described in Beckedorff et al. (2013b), which essentially included a modification of the kit protocol to have a larger amount of DNase I and a longer DNase treatment time (Beckedorff et al., 2013b) in order to ensure the elimination of contaminant genomic DNA from the polyA+ RNA fraction. DNase treatment conditions optimization was monitored by PCR with 40 cycles and 1 μg of the resulting purified polyA+ RNA (without reverse transcription), using four different pairs of primers (Supplementary Table S1), one pair for tubulin TUBA1C gene, two for multiple copy genes, namely Histone H3 and cytochrome b, and one for a mitochondrial intergenic region. Stranded cDNA libraries were prepared using the TruSeq RNA kit (version 1, rev A) then sequenced on the Illumina HiSeq 2000 using 75 bp paired-end reads. We sequenced two biological replicate samples per each treatment and obtain 80–90 million mate pairs per each of the four samples. The raw reads are deposited at NCBI in the GEO repository under Accession numbers GSE79301 and GSE100710.

For RIP-seq experiments (Zhao et al., 2010), RNA samples from Native RIP (see above) were used to prepare sequencing libraries with the SMARTer Ultra Low Input RNA Kit for Sequencing v3 (Clontech) according to the manufacturer’s instructions, then sequenced on the Illumina NextSeq500 using 150 bp paired-end reads sequencing. We sequenced two biological replicate samples per RIP with anti-Androgen Receptor (antiAR) and two with control antibody (non-specific Rabbit IgG) and we obtained 29 – 33 million mate-pairs per each of the four samples. The raw reads are deposited at NCBI in the GEO repository under Accession number GSE100710.

### RNA-Seq Data Assembly and Analysis

All RNA-seq data were trimmed of low-quality reads and clipped of sequencing adaptors with Trimmomatic (Bolger et al., 2014). Trimmed reads were aligned to hg19 version of the human genome with TopHat2 (Kim et al., 2013), followed by an assembly using the Cufflinks 2.2 tool (Trapnell et al., 2013) with the default parameters. For the alignment and assembly we used a comprehensive_reference_transcriptome custom GTF file comprised of the human transcripts from GENCODE v19 (Harrow et al., 2012) plus the lincRNAs already annotated in three other publications (Cabili et al., 2011; Prensner et al., 2011; Hangauer et al., 2013). This custom GTF can be downloaded from http://verjolab.usp.br/tracks/hg/hg19/genes/. We generated transcriptome assemblies for each of the four samples separately, and then used Cuffmerge (Trapnell et al., 2013) to combine the transcripts from all samples. The resulting merged GTF file is deposited at NCBI in the GEO repository under Accession number GSE100710. Next, we merged both GTF files mentioned above to generate a comprehensive_LNCaP_ transcriptome GTF file, which can also be downloaded from http://verjolab.usp.br/tracks/hg/hg19/genes/. We estimated the RNA abundance defined as the FPKM using Cuffdiff v 2.2 (Trapnell et al., 2013); the FPKM values were used in the corre- lation analyses with the machine learning approach (see below).

For differential expression (DE) analyses we performed count-based analysis as described in Anders et al. (2013). In summary we calculated the sum of exon read count per gene with HTSeq (Anders et al., 2015) and DE was calculated with DESeq2 (Love et al., 2014). In all DE tests, a gene was considered significantly changed if the *q*-value was less than 0.05 and fold-change (2 or ≤ -2).

### Novel LncRNAs Discovery

To classify the transcripts as either previously annotated lincRNAs or putative novel lncRNAs we used the following pipeline: first we retained all transcripts from the above assembly with FPKM ≥ 1 that were previously annotated as lncRNAs in the comprehensive reference transcriptome (custom GTF); next, we retained novel transcripts that were identified in these analyses (class code “u” identified by Cuffmerge – putative novel intergenic transcripts) with FPKM ≥ 1. All transcripts with a length < 200 nt were removed. We discarded all transcripts with coding potential, as assessed by the Coding Potential Calculator (CPC version 0.9-r2) (Kong et al., 2007), thus resulting in the set of all lincRNAs expressed in LNCaP. A GTF file with these LNCaP_lincRNAs can be downloaded from http://verjolab.usp.br/tracks/hg/hg19/genes/.

### Gene Set Enrichment Analysis (GSEA)

Gene expression fold changes obtained from our RNA-seq data (androgen treated cells relative to control samples) were calculated with DESeq2 as described above and the entire list of expressed genes was pre-ranked and imported into GSEA program (Subramanian et al., 2005) to perform gene set enrichment analyses.

### DESeq2 Analysis of RIP-Seq Data and Identification of LincRNAs Associated to AR

RIP-seq data from each of two independent biological replicates of each condition (two antiAR replicates and two control non-specific IgG replicates) were trimmed of low-quality reads and clipped of sequencing adaptors, aligned to hg19 version of the human genome and assembled with Cufflinks 2.2 as described for RNA-seq above. We used as reference for the alignment and assembly of the RIP-seq reads the comprehensive_LNCaP_transcriptome GTF file.

To detect the set of lincRNAs significantly associated to the AR, which we named AR-Associated lincRNAs (ARA-lincRNAs), we used the approach of Soh et al. (Soh et al., 2017), namely a DESeq2 analysis using HTSeq counting. A lincRNA was considered significantly associated to AR when the reads count ratio of log2 (antiAR/IgG) was greater than 0 and its FDR calculated with DEseq2 were below a threshold of 10%. A GTF file of ARA-lincRNAs can be downloaded from http://verjolab.usp.br/tracks/hg/hg19/genes/.

The entire set of 619 ARA-lincRNAs has an average expression FPKM = 2.40 in the RNA-seq analysis. The subset of 258 ARA-lincRNAs with expression significantly increased by androgen has an average expression FPKM = 3.58 in the RNA-seq analysis; this subset was further sub-divided into the 177 ARA-lincRNAs that had a protein-coding neighbor that also showed an increase in expression with androgen, and those 177 ARA-lincRNAs have an average expression FPKM = 3.12. The remaining 81 ARA-lincRNAs that had a protein-coding neighbor showing a decrease in expression with androgen have an average expression FPKM = 4.59.

### Definition of a Set of LincRNAs Non-associated to AR

In order to search for features with biological relevance in the set of ARA-lincRNAs, we created a control set of Non-significantly Associated lincRNAs (NonA-lincRNAs), which was used in the comparisons described further below. First, lincRNAs with read counts in the RIP-seq datasets (antiAR or IgG) equal to zero were excluded. The remaining lincRNA-genes with non-zero read counts were sorted in an ascending order using the absolute value of log2 (antiAR/IgG) and the first 619 top-ranked lincRNAs, corresponding to the lincRNAs with log2 (antiAR/IgG) normally distributed around zero, were assigned as the set of NonA-lincRNAs. We chose a size of 619 in order to make it identical to the size of the set of identified ARA-lincRNAs.

This selected control set of 619 NonA-lincRNAs exhibited a very similar (non-significantly different) pattern of average expression in LNCaP in the presence of androgen as that of ARA-lincRNAs. Thus, the entire set of NonA-lincRNAs have an average expression FPKM = 2.27 in the RNA-seq analysis (*p*-value = 0.79 in the comparison with the 619 ARA-lincRNAs). A selected subset of 258 NonA-lincRNAs with expression significantly increased by androgen have an average expression FPKM = 3.55 in the RNA-seq analysis (*p*-value = 0.97 in the comparison with the 258 ARA-lincRNAs); this subset was further sub-divided into a selected set of 177 NonA-lincRNAs that had a protein-coding neighbor that also showed an increase in expression with androgen, and those 177 NonA-lincRNAs have an average expression FPKM = 3.86 (*p*-value = 0.55 in the comparison with the 177 ARA-lincRNAs). A remaining set of 81 NonA-lincRNAs that had a protein-coding neighbor showing a decrease in expression with androgen has an average expression FPKM = 2.78 (*p*-value = 0.25 in the comparison with the 81 ARA-lincRNAs).

### ARA-LincRNAs Neighborhood Analysis

To associate an expressed protein-coding gene to a neighbor lincRNA, we used as candidates those protein-coding genes with FPKM > 1 in the androgen-treated or control (vehicle) RNA-seq experiments. We matched the lincRNA-protein-coding pairs using BedTools (Quinlan and Hall, 2010) with the subcommand closest to search in the human genome for the closest protein-coding TSS coordinate up or downstream the lincRNA TSS coordinate.

The gene ontology analysis on the set of protein-coding neighbor genes was performed using the functional annotation tool DAVID 6.8 (Huang et al., 2007) with the hypergeometric-test and FDR correction threshold of 5%.

### ChIP-Seq and DNAseI Datasets

The public ChIP and DNAseI datasets used in our analyses were from the following papers: (Wang et al., 2011; Zhang et al., 2011; Harrow et al., 2012; Tan et al., 2012; Kim et al., 2014; Takayama et al., 2015; Metzger et al., 2016). These are public ChIP-seq data from LNCaP cells treated with androgen hormone for histone marks H3T11P [GSM1333367], H3K27me3 [GSM1527833], H3K4me1 [GSM686928], H3K4me2 [GSM686932], H3K4me3 [GSM686935], H3K36me3 [GSM686936], H3K27ac [GSM686937], H4K5ac [GSM686939], and H2A.Z [GSM686941] (Supplementary Table S2). In addition to these histone marks, we included public data from LNCaP cells treated with androgen hormone for genome-wide mapping of the following modulators, transcription factors and histone modifying enzymes: p300 [GSM686943], FoxA1 [GSM686926], MED12 [GSM686945], NKX3-1 [GSM699633], PolII [GSM699637], AR [GSM1527823], FOXP1 [GSM1527837], RUNX1 [GSM1527840], EZH2 [GSM1527841], WDR5 [GSM1333369], AP4 [GSM714610], CTCF [GSM1006887], and LSD1 [GSM1573656], as well as for DNAse-Seq [GSM816634] (Supplementary Table S2). For all datasets, FASTQ files were trimmed of low-quality reads and clipped of sequencing adaptors as described above. Trimmed reads were aligned to hg19 using Bowtie2 (Langmead and Salzberg, 2012) with the default parameters. Enriched peaks were determined using HOMER (v 4.8) (Heinz et al., 2010) by calculating their relative significance when compared to a local background estimated through a 10-kb window centralized at the candidate peak. Peaks were considered significant with FDR values < 0.001.

### Random Forest Analysis

To find the relevant epigenetic signatures at the ARA-lincRNAs TSS we used a machine learning approach employing the Random Forest algorithm implemented in the python Scikit-Learn (v. 0.18.1) package (Pedregosa et al., 2012) with data from all epigenetic marks described above. The groups used for the Random Forest training were composed by ARA- or NonA-lincRNAs. For each ARA- or NonA-lincRNA, their epigenetic features were encoded as a vector containing the normalized read counts of all epigenetic marks at the TSS of the lincRNA and at the TSS of the corresponding expressed neighbor protein-coding gene. The counts of ChIP-Seq or DNAse-seq reads at the TSS of lincRNAs and neighbor protein-coding genes were obtained using the bedtools coverage command and were normalized by the total number of mapped reads in each dataset. As input for these coverage computations we have used the alignment bam files generated by Bowtie2 (Langmead and Salzberg, 2012) and the gene TSS coordinates (±500 bp).

Briefly, we trained the models with a different number of trees [100, 500, 1000, and 10000], setting as True the following parameters: warm start, out of bag score and gini criterion. The mean accuracy score for the models was obtained through a fivefold cross validation scheme (Fushiki, 2011). After selecting the most accurate model, we extracted the epigenetic features importance using the gini-index score generated during the training of the model. In order to create a statistical significance cut-off for those gini-index scores, we created a bootstrapped null distribution of gini-index scores shuffling 1000× the classes of ARA-lincRNAs and NonA-lincRNAs. For each shuffle round, we performed Random Forest training and extracted the gini-index scores. We consider as statistically significant only those features from the final models with gini-index score higher than the empirical null bootstrapped distribution (setting a 95% confidence interval).

### Topological Associating Domains Analysis

Topological Associating Domains (TADs) in LNCaP cells at 100 kb bin resolution were determined by Taberlay et al. (2016) and the Bed file with processed genomic coordinates was downloaded from GEO database GSE73782. The ARA- or NonA-lincRNAs were assigned to a TAD using their genomic coordinates and the bedtools intersect command. ARA-lincRNA-containing TADs were defined as TADs containing at least one ARA-lincRNA locus, irrespective of the presence or absence of NonA-lincRNA loci in those TADs, and the NonA-lincRNA-containing TADs were those containing at least one NonA-lincRNA locus, irrespective of the presence or absence of ARA-lincRNA loci. For each of the TADs the number of significant ChIP-Seq peaks for all the chromatin marks present inside the corresponding TADs were counted with the bedtools intersect command.

To measure the ARA- or NonA-lincRNA TSS distance from the closest TAD boundary we normalized the TADs length, making the maximum boundary distance (100%) the one corresponding to the intersection between the lincRNA TSS coordinate with the exact middle of the TAD, and the minimum distance (0%), that corresponding to the lincRNA TSS overlapping the TAD boundary coordinate.

## Results

### Deep RNA Sequencing Reveals the Expression of Thousands of Novel LincRNAs in Prostate

**FIGURE 1:**
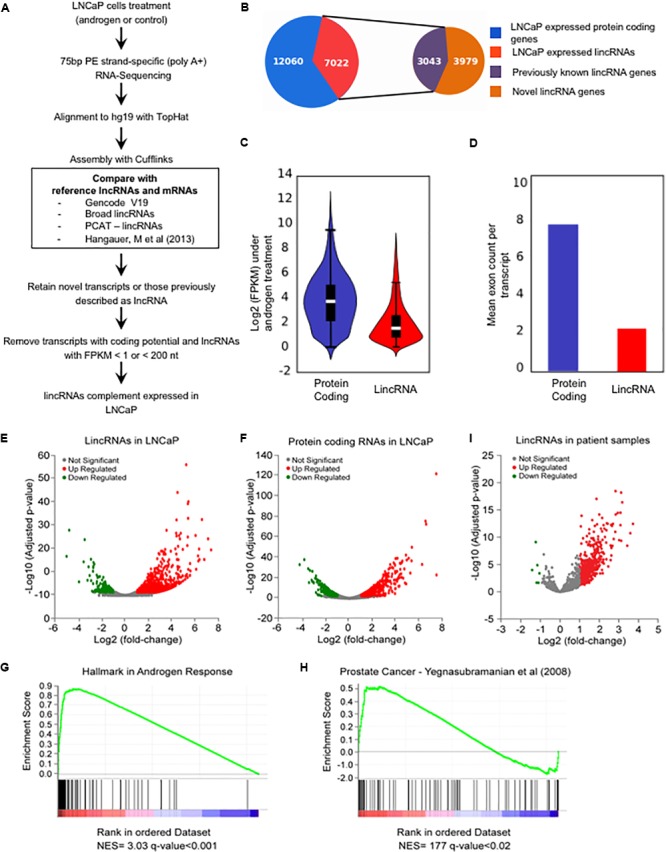
The androgen-responsive Long Intergenic Non-coding RNAs transcriptome in LNCaP cells. **(A)** A schematic illustration of the procedure used to discover and define the complement of lincRNAs expressed in LNCaP cells. **(B)** Pizza plot depicting the LNCaP-expressed transcripts already listed as lincRNAs in the comprehensive reference transcriptome from the literature (purple) as well as the novel lincRNAs (brown) identified here. **(C)** Violin plot representing the distribution of log2 (FPKM) values of protein-coding genes (blue) and lincRNAs (red) expressed in LNCaP cells (FPKM > 1) in the presence of 1 nM androgen. **(D)** Mean number of exons per transcript among the protein-coding genes (blue) and the lincRNAs (red) expressed in LNCaP cells. **(E,F)** Volcano plots displaying the differentially expressed genes when comparing androgen-treated and control LNCaP cells (*n* = 2). Significance cutoff at adjusted *p*-value ≤ 0.05 and fold-change ≥ 2 (red) or ≤–2 (green). **(E)** Androgen-responsive lincRNAs. **(F)** Androgen-responsive protein-coding genes. **(G,H)** GSEA analyses depicting the enrichment of hallmark androgen-responsive genes **(G)** and of prostate cancer genes **(H)** among the protein-coding genes with androgen-induced changes in expression in LNCaP cells. The gene sets are ordered by normalized enrichment score (NES). The *q*-value is the false discovery rate, that is, the estimated probability that the normalized enrichment score represents a false positive finding. **(I)** Volcano plot representation of the differentially expressed lincRNAs in prostate cancer patient tissue samples compared with adjacent non-tumor tissue [re-analysis of raw RNA-seq data from Ren et al. (2012) to include all lincRNAs described here]; significance cutoff at adjusted *p*-value ≤ 0.05 and fold-change ≥ 2 (red) or ≤–2 (green).

In order to identify the androgen-responsive lincRNAs complement in the prostate, RNA-seq data was obtained for prostate cancer LNCaP cells treated with 1 nM synthetic androgen (R1881) for 24 h, or with vehicle; deep sequencing was achieved, with approximately 80–90 million strand-specific reads per library, in duplicate. RNA-seq reads were aligned to the genome and assembled using a comprehensive reference transcriptome that includes the lincRNAs from four papers in the literature (Cabili et al., 2011; Prensner et al., 2011; Harrow et al., 2012; Hangauer et al., 2013) (**Figure 1A**), thus creating a high-quality catalog of the lincRNAs complement expressed in LNCaP prostate cancer cells. We detected a total of 7022 lincRNAs, of which 3979 lincRNAs (57%) were identified as putative novel transcripts expressed in LNCaP cells and not present in the reference transcriptome (**Figure 1B**); in addition, 3043 known lincRNAs (43%) present in the reference transcriptome were detected as expressed in LNCaP (**Figure 1B**), of which 683 match lincRNAs annotated in GENCODE v19 (Harrow et al., 2012), 461 are lincRNAs from Cabili et al. (2011), 88 are PCAT lincRNAs from Prensner et al. (2011) and 1811 are lincRNAs from Hangauer et al. (2013). The list of all 7022 lincRNAs expressed at FPKM ≥ 1 in LNCaP (comprised of 14223 transcript isoforms) is given in Supplementary Table S3 and can be viewed in a custom track at the UCSC human genome browser^1^.

We observed that on average the lincRNAs have lower expression when compared with the protein-coding genes, with mean 1.2 and 3.2 FPKM (**Figure 1C**), respectively, and lincRNAs are less spliced than protein-coding genes with an average of 2.3 and 7.9 exons per transcript, respectively (**Figure 1D**); these findings are in line with previous results from the literature on lncRNAs (Nakaya et al., 2007; Cabili et al., 2011; Derrien et al., 2012). Overall, this effort has cataloged a comprehensive mapping of lincRNA expression in LNCaP prostate cells.

### Androgen Induced Widespread Changes in LincRNAs Expression

A total of 893 lincRNAs were detected as differentially expressed under androgen stimulation (700 up-regulated and 193 down-regulated) (**Figure 1E**) (Supplementary Table S4), as well as 1000 differentially expressed protein-coding genes (606 up-regulated and 394 down-regulated) (**Figure 1F**) (Supplementary Table S5). Interestingly, out of the 893 lincRNAs, a total of 524 are novel androgen-stimulated lincRNAs identified in our study (**Table 1**). In addition, several known androgen-stimulated protein-coding genes such as *FASN, NDRG1, PSA, TMPRSS2, KLK2*, and *KLK4* (Yang et al., 2013) were significantly and highly activated when we compared stimuli and control conditions; also, lincRNAs that were described in the literature as up-regulated by androgen, such as *PCAT18* (Crea et al., 2014), or down-regulated by androgen, such as *PCAT29* and *DRAIC* (Sakurai et al., 2015), were confirmed here, thus attesting to an overall accuracy of our RNA-Seq experiments. Among the lincRNAs detected by RNA-seq as differentially expressed with androgen we selected four to be further confirmed by RT–qPCR; these four were among the lincRNAs also detected as up-regulated in the patient tumor samples (see below). All of them showed an androgen-induced 1.8–3.8-fold increase in expression (Supplementary Figure S1).

**Table 1:** Number of novel and previously known lincRNAs expressed in LNCaP cells.

LincRNAs expressed in LNCaP		Not responsive	Androgen responsive		
			Up-regulated	Down-regulated	Total
In the reference	3043	2674	268	101	369
Novel	3979	3455	432	92	524
Total	7022	6129	700	193	893

We conducted a gene set enrichment analysis (GSEA) with the protein-coding genes detected as expressed in our RNA-seq assay. Briefly, a rank-ordered list was generated for all protein-coding genes, comparing androgen treatment with the control, and this list was used as input to a pre-ranked GSEA analysis. As expected, the analysis demonstrated a significant enrichment (*q*-value < 0.001) of hallmark genes in androgen response (**Figure 1G**) and of genes belonging to the prostate cancer signature (*q*-value < 0.02) (**Figure 1H**); overall, these results again add confidence to the set of novel lincRNAs identified by RNA-seq analyses of LNCaP cells upon treatment with androgen hormone.

### LincRNAs in Prostate Cancer

Numerous studies demonstrated that lincRNAs contribute to cancer development and progression (Huarte and Rinn, 2010; Beckedorff et al., 2013a). We have re-analyzed a publicly available prostate cancer RNA-seq dataset (Ren et al., 2012) to investigate the expression levels in these samples of the lincRNAs detected here in LNCaP; we mapped the raw RNA-seq data from paired prostate cancer tumor samples and their matched non-tumor tissues (Ren et al., 2012) to the human genome (hg19) and calculated the differentially expressed genes between cancer and normal tissues. From our 7022 LNCaP-expressed lincRNAs we identified 1281 lincRNAs that were expressed in at least 75% of the patient cancer samples. From these 1281 lincRNAs, 424 were significantly differentially expressed in prostate tumor tissue compared with adjacent non-tumor (418 up-regulated and 6 down-regulated in tumor, **Table 2**) (**Figure 1I**). A detailed list of all 424 lincRNAs is given in Supplementary Table S6.

**Table 2:** LincRNAs differentially expressed in prostate patient tumor samples compared with adjacent non-tumor tissues.

LincRNAs expressed in LNCaP cells anddetected in patient samples^a^		No significant differential expression^b^	Differentially expressed in patient tumor samples^b^		
			Up-regulated	Down-regulated	Total
In the reference	915	630	280	5	285
Novel	366	227	138	1	139
Total	1281	857	418	6	424

*^*a*^Re-analysis of raw RNA-seq data from Ren et al. (2012) to include the lincRNAs described in the present work. The number of lincRNAs that were expressed in at least 75% of the patient cancer samples is shown. ^*b*^Comparison of tumor samples with respect to adjacent non-tumor tissue for each of the paired patient samples.*

We detected *PCA3, PVT1*, and *GAS5* lincRNAs as differentially expressed genes in the patient cancer samples compared with adjacent non-tumor tissues; these lincRNAs have been recently shown to be associated with prostate cancer (Bawa et al., 2015; Shih et al., 2015). Interestingly, among the 1281 lincRNAs identified as expressed in the patients’ dataset, 157 were responsive to androgen in LNCaP cells (**Table 3**). Among them, 67 lincRNAs were differentially expressed in patient tumor samples compared with non-tumor tissue (**Table 3**), and the majority was up-regulated in the tumors (65 out of 67). A total of 23 of these lincRNAs differentially expressed in patient tumor samples were novel lincRNAs detected here, and Supplementary Table S7 gives the list of all 67 lincRNAs. They might be potential lincRNA candidates to be further explored as possible targets for treatment of hormone dependent prostate cancer.

**Table 3:** Androgen-responsive lincRNAs expressed in prostate patient samples and identification of the ones differentially expressed in tumor.

LincRNAs responsive to androgen in LNCaP cells, and detected in patient samples^a^		No significant differential expression^b^	Differentially expressed in patient tumor samples^b^		
			Up-regulated	Down-regulated	Total
In the reference up-regulated by androgen	73	43	29	1	30
In the reference down-regulated by androgen	44	30	13	1	14
Novel up-regulated by androgen	26	13	13	0	13
Novel down-regulated by androgen	14	4	10	0	10
Total	157	90	65	2	67

*^*a*^Re-analysis of raw RNA-seq data from Ren et al. (2012) to include the lincRNAs described in the present work. The number of lincRNAs that were androgen responsive in LNCaP and that were expressed in at least 75% of the patient cancer samples is shown. ^*b*^Comparison of tumor samples with respect to adjacent non-tumor tissue in each of the paired patient samples.*

### Identification of Hundreds of AR-Associated LincRNAs

To identify lincRNAs that potentially interact with AR and could mediate the AR gene regulatory programs, we performed native RNA-binding protein immunoprecipitation (RIP) (Zhao et al., 2010) of AR with a specific antiAR antibody followed by RNA-seq of co-immunoprecipitated RNAs. Before sequencing, we performed control RT-qPCR tests with two lincRNA genes expected to bind to AR, namely *PCGEM1* (Yang et al., 2013) and *eKLK3* (Hsieh et al., 2014) and one negative control, namely *MALAT1* (Yang et al., 2013). Supplementary Figure S2 shows that the two positive lincRNA controls, *PCGEM1* and *eKLK3* were detected in the IP fraction, whereas the negative control *MALAT1* was not. After sequencing, mapping to the genome and counting the reads abundance at each lincRNA locus, and in order to evaluate the reproducibility of the RIP-Seq experiments, we calculated the Pearson correlation between the two replicates and found it to be *r* = 0.9967 for the antiAR assay and *r* = 0.9988 for the control non-specific IgG assay.

**FIGURE 2:**
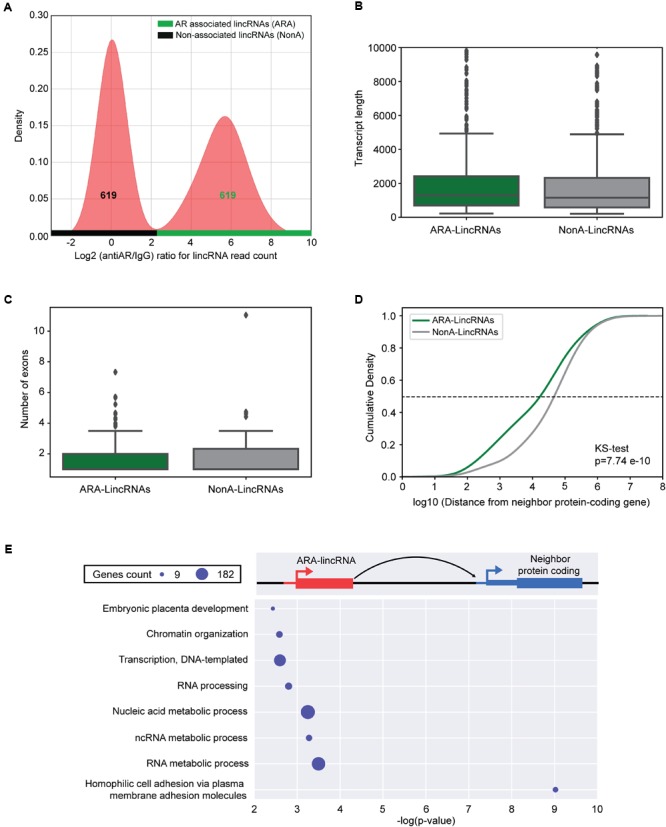
lincRNAs are associated to the androgen receptor in LNCaP cells. **(A)** Density distribution of log2 ratio (antiAR/IgG) for the lincRNAs read counts detected in the RIP-seq assay with antiAR antibody (antiAR) or with control non-immune serum (IgG). RIP-seq assay was performed in duplicate in LNCaP cells treated for 6 h with 0.1 nM androgen. The curve on the right (over the horizontal green bar) shows the distribution of 619 lincRNAs that were significantly enriched (FDR < 10%, DESeq2) in the anti-androgen receptor (antiAR) fraction in relation to the control fraction (non-specific IgG), which are the androgen-receptor associated lincRNAs (ARA-lincRNAs). The curve on the left (over the horizontal black bar) shows distribution of an equivalent set of 619 lincRNAs with log2 ratio (antiAR/IgG) distributed around zero in the RIP-seq assay, representing lincRNAs non-associated to the androgen receptor (NonA-lincRNAs), which were chosen as a control group. **(B)** Box plot of the lengths of ARA-lincRNAs (red) and of NonA-lincRNAs (gray). **(C)** Box plot of the number of exons of ARA-lincRNAs (red) and of NonA-lincRNAs (gray). **(D)** Cumulative distribution of the distance in bases between the lincRNA of the indicated group (ARA-lincRNAs, green or NonA-lincRNAs gray) and the nearest neighbor protein coding genes in the genome (KS, Kolmogorov–Smirnov test; *p*-value = 7.74 × 10^-10^). **(E)** Protein-coding genes (blue) neighbor to the 619 ARA-lincRNAs belong to the indicated significantly enriched biological process GO terms. The *x*-axis shows the enrichment significance [-log10 (*p*-value), hypergeometric statistical test]; all GO terms have a corrected *p*-value < 0.05 for the enrichment significance. The sizes of the blue circles are proportional to the number of protein-coding genes in each enriched term, as indicated in the scale on the upper left corner.

A total of 619 lincRNAs were identified as significantly enriched (FDR < 10%, DESeq2) in the anti-Androgen Receptor (antiAR) fraction in relation to the control fraction (non-specific IgG), and we named them AR-Associated-lincRNAs (ARA-lincRNAs) (**Figure 2A**, green horizontal bar). Among these ARA-lincRNAs, 267 were novel lincRNAs not present in the comprehensive reference dataset of public lincRNAs. The list of 619 ARA-lincRNAs (comprising 1506 transcript isoforms) is given in Supplementary Table S8 and their sequences in Supplementary Table S9. The mean length of the ARA-lincRNAs is 2111 nt (**Figure 2B**, green) and the average number of exons per transcript is 1.6 (median 1.0) (**Figure 2C**, green). To our knowledge, this is the first report of hundreds of lincRNAs significantly associated to the AR in the presence of androgen. All ARA-lincRNAs can be viewed in a custom track at the UCSC human genome browser^2^.

Assuming that lincRNAs can act in *cis*, regulating genes in their neighborhood (Hsieh et al., 2014; Ilott et al., 2014; Quinn and Chang, 2016), we searched for the expressed protein-coding gene closest to each ARA-lincRNA (Supplementary Table S8). Further, in order to identify the chromatin modification marks with biological relevance in this set of ARA-lincRNAs and their neighbor protein-coding genes, we defined a control set of Non-significantly AR-associated lincRNAs (NonA-lincRNAs) having the same number of elements as the ARA-lincRNAs set and corresponding to the lincRNAs with log_2_ (antiAR/IgG) ratio normally distributed around zero, i.e., lincRNAs that were equally associated to AR or IgG (**Figure 2A**, black horizontal bar) (see section “Materials and Methods”). This was used as a control set (Supplementary Table S8) in the comparisons described further below. The mean length of the NonA-lincRNAs is 1957 nt (**Figure 2B**, gray) and the average number of exons per transcript is 1.7 (median 1.0) (**Figure 2C**, gray), very similar to the characteristics of the set of ARA-lincRNAs (see above). In contrast, the median distance (see dashed line, **Figure 2D**) between the ARA-lincRNA and the closest expressed protein coding gene is 21 kb, while for the NonA-lincRNAs the median distance is significantly longer (*p*-value = 7.74 × 10^-10^, Kolmogorov–Smirnov test), namely 49 kb (**Figure 2D**, green and gray curves, respectively).

**FIGURE 3:**
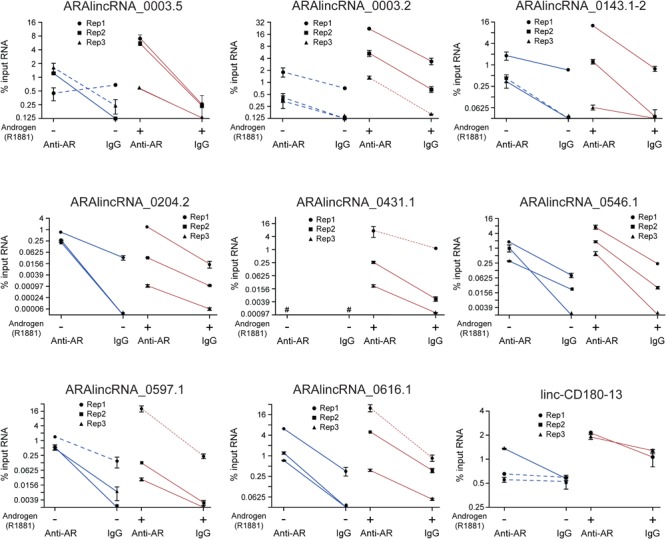
Validation by RIP-qPCR of a set of ARA-lincRNAs, which were detected in the RIP-seq assay as associated to AR. LNCaP cells treated with 0.1 nM androgen (red lines) or with vehicle control (blue lines) were assayed as indicated on the *x*-axis. The amount of the indicated lincRNA that was co-immunoprecipitated with antiAR antibody or with IgG from non-immunized rabbit (negative control) was measured by RT-qPCR in three different biological replicates (each represented with a different symbol), and the corresponding points for antiAR and IgG for each replicate are connected with a straight line. The results are shown as % input RNA (mean ± SEM) of three technical replicates for each individual biological replicate. For the four lincRNA genes in the experiment with androgen whose experimental points are connected with red dotted lines, the amount of RIP material was only enough for two technical replicates each, and the enrichment *t*-test was not applied. Red and blue solid lines = significant difference between antiAR and IgG (*p* < 0.05); blue dashed lines = non-significant difference, *t*-test.

To validate the RIP-seq results, we performed RIP-qPCR with a selected set of nine different ARA-lincRNAs (**Figure 3**), which were chosen based on their neighbor protein coding genes (Supplementary Table S8). Selected ARA-lincRNAs are neighbors of protein-coding genes *WDR5, ZNF706, TFRC*, and *FLNA*, which have been described as up-regulated in prostate cancer, and of *CENPH* and *RAB11FIP3*, which have been shown to play a role in many other types of cancer. Also chosen were two randomly selected ARA-lincRNAs that are neighbors of *C1orf174* and *GNPTAB*, which have not been related to any cancer, however, they encode proteins that are shown at The Human Protein Atlas^3^ as being possible prognostic markers of a number of cancers (Uhlen et al., 2017).

We confirmed by RIP-qPCR in LNCaP cells in the presence of androgen that for each of three different biological replicates (**Figure 3**, three individual red lines in each panel), the amount of lincRNA associated to the antiAR fraction was significantly higher than that associated to the non-specific IgG control (**Figure 3**, red solid lines), even though the absolute value of the % input of antiAR-associated lincRNA was different for each biological replicate (each symbol). In LNCaP cells without androgen (**Figure 3**, blue lines) there was an important decrease in the amount of ARA-lincRNA associated to AR for eight out of the nine lincRNAs tested, with the exception of ARA-lincRNA_0204.2 (**Figure 3**, compare blue and red lines), and in the absence of androgen a significant enrichment was detected in the antiAR fraction compared with the IgG control for only three out of the nine ARA-lincRNAs tested (**Figure 3**, blue solid lines).

Next, we looked for enriched Gene Ontology (GO) terms among the protein-coding genes that are neighbors to ARA-lincRNAs, and separately also those that are neighbors to NonA-lincRNAs. In this analysis, the protein-coding genes neighboring ARA-lincRNAs exhibited statistically significant (FDR < 5%, hypergeometric-test) enriched GO terms (**Figure 2E**), while the NonA-lincRNAs control group did not show any significantly enriched term. Protein-coding genes neighbor to ARA-lincRNAs are enriched for GO terms such as chromatin organization and cell adhesion, both relevant for prostate cancer development and maintenance (Mason et al., 2002; Prensner et al., 2013; Gu et al., 2015). Transcription, DNA-dependent and RNA processing are also enriched GO terms (**Figure 2E**) among the protein-coding genes near ARA-lincRNAs. These terms relate to anabolic processes preceding cell division, which are increased in cancer (White, 2005; Lin et al., 2012), and are in line with the role of androgen in prostate cancer cells.

### Epigenetic Profile of ARA-LincRNAs and Protein-Coding Neighbors

**FIGURE 4:**
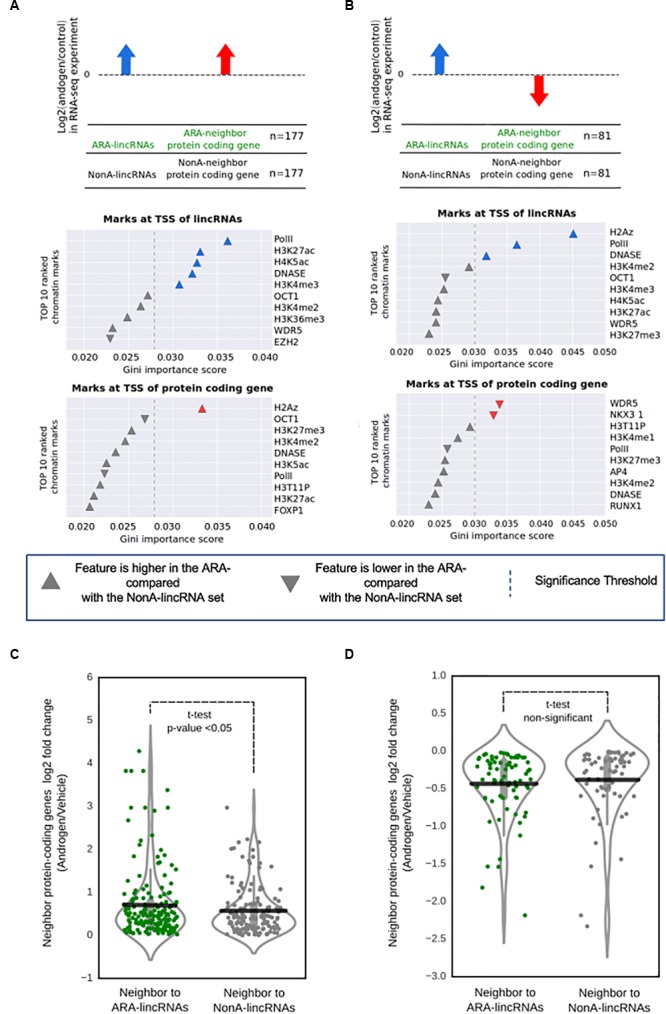
Promoter regions at the ARA-lincRNAs and at the neighbor protein-coding genes exhibit a specific epigenetic signature. **(A)** Enriched epigenetic marks for the set of ARA-lincRNAs that were neighbors to protein-coding genes whose transcription was activated by androgen. **(B)** Enriched epigenetic marks for the set*of ARA-lincRNAs that were neighbors to protein-coding genes whose transcription was inhibited by androgen. Each panel shows the rank of the top ten most relevant epigenetic marks at the TSS of genes, which were determined by the Random Forest machine learning classifier algorithm to discriminate between ARA-lincRNAs and NonA-lincRNAs; these epigenetic marks were ranked based on their discriminatory potential given by the Gini importance score. Up arrow indicates that the feature is more abundant at the TSS of the ARA-lincRNA (or at the TSS of the protein-coding neighbor) compared with the NonA-lincRNA or the protein-coding neighbor; down arrow indicates that the feature is less abundant at the TSS of the ARA-lincRNA (or at the TSS of the protein-coding neighbor) compared with the NonA-lincRNA or the protein-coding neighbor. Colored arrows indicate that the epigenetic mark enrichment was statistically significant; the statistical significance threshold (dashed line) of the Gini importance score was determined by shuffling the feature vectors between the ARA- and NonA-lincRNAs in a bootstrap procedure (see section “Materials and Methods”). **(C)** Distribution of the log2 ratio (androgen/vehicle) in the RNA-seq experiment for protein-coding genes with androgen-activated transcription that are neighbors to ARA-lincRNAs (green points, *n* = 167) compared with protein-coding genes that are neighbors to NonA-lincRNAs (black points, *n* = 145). All ARA-lincRNAs and NonA-lincRNAs that have a Pol II epigenetic mark at their TSS were included in this analysis. The statistical significance (*p* < 0.05) of the difference between the average expressions (black horizontal lines) of the two groups was computed using a *t*-test. **(D)** Distribution of the log2 ratio (androgen/vehicle) in the RNA-seq experiment for protein-coding genes with androgen-inhibited transcription that are neighbors to ARA-lincRNAs (green points, *n* = 75) compared with protein-coding genes that are neighbors to NonA-lincRNAs (black points, *n* = 67). No statistical difference between the average expressions (black horizontal lines) of the two groups was detected.*

We looked for the presence of a particular epigenetic signature at the TSS of ARA-lincRNAs and of protein-coding neighbors, in order to shed light on the possible mechanism by which the ARA-lincRNAs could affect the expression of neighbor protein-coding genes. Thus, we selected the 258 ARA-lincRNAs whose expression was increased upon androgen treatment (as detected in the RNA-Seq experiment). These ARA-lincRNAs with androgen-activated expression were further separated into two groups, the first comprising 177 lincRNAs whose protein-coding gene neighbors also had their expression activated by androgen (**Figure 4A**, upper panel), and the second having 81 lincRNAs whose neighbor protein-coding genes had a reduced expression upon androgen treatment (**Figure 4B**, upper panel). To characterize this epigenetic profile, we used 23 public ChIP-seq datasets from LNCaP cells treated with androgen, comprising the genomic coordinates for binding sites of several histone marks and transcription factors, as well as the coordinates of Polymerase II and DNAseI accessibility (see section “Materials and Methods” and Supplementary Table S2 for the accession numbers of the datasets).

We first concentrated the analysis on the set of 177 ARA-lincRNAs with androgen-activated expression of protein-coding neighbors. Using a ± 500 bp window centered at the TSS of each ARA-lincRNA and of the respective neighboring protein-coding gene, we computed for each ChIP-seq mark the normalized number of reads inside the TSS regions (Supplementary Figure S3A). Examples of the distribution of the abundance of different marks at the TSS of ARA-lincRNAs (Supplementary Figure S4A, red lines) and of neighbor protein-coding genes (Supplementary Figure S4B, red lines) can be seen. The top most relevant marks (as later defined in the machine learning classifier analyses below) are given as examples for each group. In parallel, the same analysis was done for the set of NonA-lincRNAs (Supplementary Figures S4A,B, blue lines).

We used a Random-Forest machine learning algorithm to identify the epigenetic marks having different abundances that separate ARA-lincRNAs from NonA-lincRNAs, as schematically described in Supplementary Figures S3B,C (see also section “Materials and Methods”). The group of 177 ARA-lincRNAs with androgen-activated expression of the protein-coding neighbors was separated from the NonA-lincRNAs (control) by the machine learning algorithm with a mean accuracy score of 0.629 (Supplementary Table S10). In a similar way, the group of 81 ARA-lincRNAs whose neighbor protein-coding genes had a reduced expression upon androgen treatment was separated from the control (NonA-lincRNAs) with a mean accuracy score of 0.597 (Supplementary Table S10).

To access the relative relevance among the chromatin mark features that permitted the separation of ARA-lincRNAs from NonA-lincRNAs, we ranked the epigenetic marks identified by the machine learning algorithm according to the Gini importance score (Gini-index) generated by the Random-Forest training process (**Figures 4A,B**). A set of significantly enriched epigenetic marks were at the top of the rank when looking at the TSS of ARA-lincRNAs whose neighbor protein-coding genes had an androgen-induced increase in expression (**Figure 4A**, middle panel). A different set of marks was enriched at the TSS of ARA-lincRNAs whose neighbor protein-coding genes had an androgen-induced inhibition of expression (**Figure 4B**, middle panel). Also, three other significantly enriched epigenetic marks were found at the TSS of the neighbor protein-coding genes (**Figures 4A,B**, bottom panels).

It is noteworthy that PolII and DNAseI accessibility marks were significantly more abundant at the TSS of ARA-lincRNAs compared with NonA-lincRNAs, for ARA-lincRNAs with neighbor protein-coding genes with activated or inhibited expression upon androgen stimulation (**Figures 4A,B**, middle panels). The H3K27ac mark, an important mark present in active enhancers (Creyghton et al., 2010) and the H4K5ac mark, important for the post-mitotic gene activation (Zhao et al., 2011), were enriched at the TSS of ARA-lincRNAs that have protein-coding gene neighbors with androgen-activated transcription (**Figure 4A**, middle panel). In contrast, the ARA-lincRNAs that have protein-coding gene neighbors with inhibited transcription upon androgen addition showed an enrichment of the H2A.Z histone variant at their TSS (**Figure 4B**, middle panel). The H2A.Z histone variant acts establishing and maintaining the interaction between enhancer and promoters, in addition to Polymerase II recruitment to eRNAs transcribed regions (Brunelle et al., 2015). Interestingly, H2A.Z was also enriched at the TSS of protein-coding genes with androgen-activated transcription (**Figure 4A**, bottom panel).

For those protein-coding gene neighbors of ARA-lincRNAs whose expression was inhibited upon androgen treatment, we found at their TSS a decrease in abundance of the AR co-activators WDR5 and NKX3-1 compared with the protein-coding gene neighbors of NonA-lincRNAs (**Figure 4B**, bottom panel). Both proteins are important drivers of the proliferation process and cell survival triggered by androgen stimulus (Tan et al., 2012; Kim et al., 2014).

These characteristic epigenetic profiles identified here at the TSS of ARA-lincRNAs and neighbor protein-coding genes suggest that elements associated with chromatin and the chromatin accessibility itself can contribute to the fine-tuning of AR regulation of expression when the receptor is associated to lincRNAs.

### Protein-Coding Genes Have Their Expression Affected by the Presence of Neighbor AR-Associated LincRNAs

In order to look for evidence that the presence of ARA-lincRNAs and their characteristic epigenetic signature at the TSS could modify the expression of the neighbor protein-coding genes, we picked all ARA-lincRNAs and NonA-lincRNAs that have RNA PolII signal at their TSS. We compared the expression levels of the protein-coding genes whose transcription had been activated by androgen and are neighbors to these ARA-lincRNAs or NonA-lincRNAs.

We detected that the protein-coding genes with androgen-activated transcription that are neighbors to ARA-lincRNAs have a higher average expression ratio (*p*-value < 0.05, *t*-test) (**Figure 4C**, green points) when compared with the control group comprising the protein-coding genes that are neighbors to NonA-lincRNAs (**Figure 4C**, black points). These results show that in LNCaP cells, the presence of ARA-lincRNAs could enhance the change in expression of neighbor protein-coding genes induced by androgen, causing an enhanced androgen-activated expression. ARA-lincRNAs transcription and binding to AR could direct the remodeling of epigenetic marks at these loci; alternatively, we cannot rule out the possibility that pre-existing epigenetic marks and chromatin structure could be exploited by ARA-lincRNAs that are transcribed at these loci, in order to fine-tune the androgen-activated expression.

When the expression of the protein-coding genes that had androgen-inhibited transcription was analyzed, the average expression ratio was lower for the neighbors of ARA-lincRNAs (**Figure 4D**, green points), when compared with the control group, comprising the protein-coding genes that are neighbors of NonA-lincRNAs (**Figure 4D**, black points), however, the difference in the average expressions ratios was not statistically significant, possibly related to the fact that this analysis included a small set of only 81 ARA-lincRNAs whose neighbor protein-coding genes have a reduced expression, compared with the 171 ARA-lincRNAs in the androgen-activated set.

### Chromatin Profile Inside Topologically Associating Domains Are Modified by the Presence of ARA-LincRNAs

**FIGURE 5:**
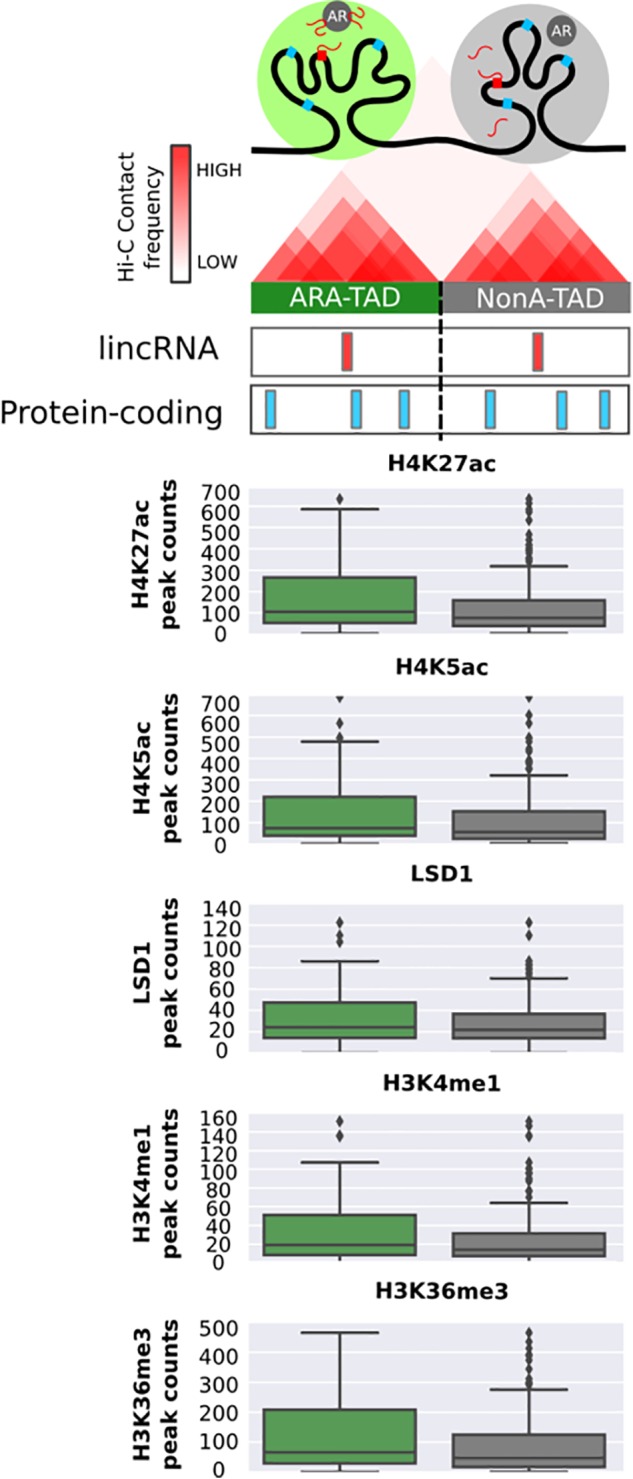
Topologically associating domains containing ARA-lincRNAs are enriched with a set of epigenetic marks in LNCaP cells. Box plots with peak counts (*Y*-axis) for a group of epigenetic marks that are present in TADs containing ARA-lincRNAs (green boxes) or containing NonA-lincRNAs (gray boxes). Only the marks with a significant difference (one-sided *t*-test, *p* < 0.05) between the two groups are shown.

In order to take into account the influence of the genomic architecture on the chromatin profile in the neighborhood of ARA-lincRNAs, we used public data of LNCaP TADs (Taberlay et al., 2016). We cataloged all TADs containing at least one of those lincRNAs (**Figure 5**, upper scheme) by cross-matching the genomic TAD coordinates with the genomic coordinates of all androgen-activated ARA- and NonA-lincRNAs. Next, we computed the number of significantly enriched epigenetic marks, available in the public ChIP-Seq datasets for LNCaP cells (see section “Materials and Methods”) that were present in the TADs containing ARA-lincRNAs or NonA-lincRNAs and compared the two sets. We found a significantly higher (*p*-value < 0.05, *t*-test) average abundance of the H3K27ac, H4K5ac, LSD1, H3K4me1, and H3K36me3 epigenetic marks in TADs containing ARA-lincRNAs compared with TADs containing NonA-lincRNAs (**Figure 5**).

### Transcription Start Sites of ARA-LincRNAs Show an Active Enhancer Profile

**FIGURE 6:**
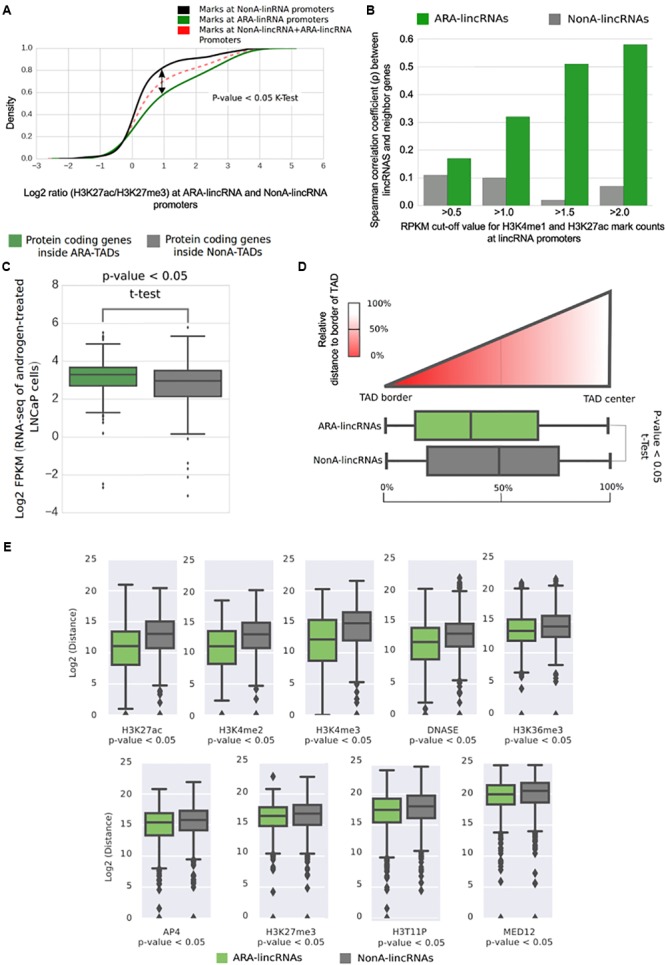
Transcription start sites of ARA-lincRNAs have an active enhancer epigenetic profile and ARA-lincRNA expression correlates with that of the protein-coding gene neighbor. **(A)** Cumulative distribution of the log2 ratio (H3K27ac/H3K27me3) of active enhancer marks mapped at the ARA-lincRNA promoters*(green) and at the NonA-lincRNA promoters (black). ARA-lincRNAs have a significantly higher (one-sided Kolmogorov–Smirnov *K*-test; *p* < 0.05) positive log2 ratio than NonA-lincRNAs. **(B)** Correlation between androgen-induced expression activation of lincRNAs and of neighbor protein-coding genes. Correlation was computed for the set of ARA-lincRNAs (green) and for the control set of NonA-lincRNAs (gray). Different, more restricted sets of lincRNA/protein-coding gene pairs were used, with increasingly more stringent cut-off values for the read-counts of enhancer marks H3K4me1 and H3K27ac at the TSS of the lincRNAs (ranging from >0.5 to >2.0), as indicated on the *x*-axis. **(C)** Box plot of the log2 FPKM in LNCaP cells for all protein-coding genes whose genomic coordinates are inside TADs that contain ARA-lincRNAs (green) and for all protein-coding genes inside TADs that contain NonA-lincRNAs (gray). The mean FPKMs are significantly different (one-sided *t*-test < 0.05) between the two groups, showing that protein-coding genes inside ARA-lincRNA-containing TADs are on average more highly expressed than those in TADs not containing ARA-lincRNAs. **(D)** The *x*-axis shows the distribution of TSS genomic coordinates of ARA-lincRNAs (green) and of NonA-lincRNAs (gray) relative to the closest TAD border and TAD-center. The ARA-lincRNAs (green) exhibited a significantly (one-sided *t*-test < 0.05) higher proximity to TAD borders than NonA-lincRNAs (gray). **(E)** Box plot of log2 (distance) in bp along the genome between the genomic locations of the indicated epigenetic mark and of the nearest TSS for either the ARA-lincRNAs (green) or the NonA-lincRNAs (gray) expressed in LNCaP cells. The average distances were significantly different (*p* < 0.05, *t*-test) between the two groups of lincRNAs, for each epigenetic mark shown here.*

To gain further insight into the possible ARA-lincRNAs epigenetic similarity with enhancers, we used the ratio between H3K27ac and H3K27me3 marks at their TSS to investigate if ARA-lincRNA promoters were similar to poised or to active enhancers (Heinz et al., 2015). The cumulative distribution of the log_2_ ratio (H3K27ac/H3K27me3) indicated that ARA-lincRNA promoters exhibited an H3K27ac/H3K27me3 ratio significantly higher (KS-test, *p* < 0.05) than NonA-lincRNA promoters (**Figure 6A**), and therefore the promoters of ARA-lincRNAs in LNCaP cells are more similar to active enhancers than those of NonA-lincRNAs.

Because the expression of lincRNAs with enhancer signature at promoters is known to have a higher correlation with neighbor genes than genes with non-enhancer signature (Ilott et al., 2014; Mao et al., 2015), and considering that ARA-lincRNAs have characteristics of active enhancers, we decided to investigate the correlation between the expression fold-change of ARA- or NonA-lincRNAs and the fold-change of the neighbor protein-coding genes upon androgen stimulation. We included in the analysis different threshold values for the abundance of the canonical enhancer marks H3K4me1 and H3K27ac at their promoters (varying from RPKM > 0.5 to >2.0) (**Figure 6B**). For all groups tested we found a significant (*p*-value < 0.05) correlation of expression fold-change between ARA-lincRNAs and their closest protein-coding gene neighbor. The correlation coefficient increased from ρ = 0.17 to ρ = 0.58 (**Figure 6B**, green) when the analysis was performed with increasingly more restricted sets of genes comprising ARA-lincRNAs with a more stringent canonical enhancer signature, i.e., with more abundant H3K4me1 and H3K27ac marks at their promoters. This effect was not observed when the same approach was applied to NonA-lincRNAs (**Figure 6B**, gray). This result shows that the *cis*-regulatory potential of enhancer-like lincRNAs in LNCaP cells stimulated with androgen is higher for lincRNAs associated to the AR compared with non-associated.

### LNCaP Cell TADs Containing ARA-LincRNAs Have Protein-Coding Genes With Higher Expression Than Those Containing NonA-lincRNAs

Next, we investigated if the expression of all protein-coding genes inside TADs containing ARA-lincRNAs was significantly different from that of all protein-coding genes inside TADs containing NonA-lincRNAs. Analyzing our RNA-seq data from LNCaP cells, we found a significantly higher (*p*-value < 0.05, *t*-test) average expression for all protein-coding genes inside TADs containing ARA-lincRNAs (**Figure 6C**, green) compared with all protein-coding genes inside TADs containing NonA-lincRNAs (**Figure 6C**, gray).

Together this data show that chromatin has a particular epigenetic composition inside TADs containing ARA-lincRNAs, mainly comprising activate enhancer marks, which agree with a higher average expression of protein-coding genes observed inside TADs containing ARA-lincRNAs.

Topologically associating domains boundaries are important chromatin regions flanking TAD elements. These regions have a small count of contacts with chromatin outside of their TADs and bind to several architectural protein clusters (Van Bortle et al., 2014). To examine the possible differential proximity of ARA-lincRNAs to the TAD boundaries, we calculated the relative distance of ARA-promoters and NonA-promoters to the closest TAD border; the dispersion of distances for each group suggests that ARA-lincRNA promoters (**Figure 6D**, green) are significantly closer (*p*-value < 0.05, one-tail *t*-test) to TAD-border regions than NonA-lincRNA promoters (the control group) (**Figure 6D**, gray).

Interestingly, we found no difference between the distance of AR ChIP-seq peaks and the TSS of lincRNAs when ARA- and NonA-lincRNA groups were compared (Supplementary Figure S5). This indicates that recruitment of AR to DNA sites is not strictly dependent on the presence of an ARA-lincRNA in the vicinity of the AR binding sites on the genome. In contrast, peaks of MED12, a component of the transcriptional pre-initiation complex and of AP4 transcription factor, are localized significantly closer to ARA-lincRNAs than to NonA-lincRNAs (**Figure 6E**), indicating a higher density of these transcriptional regulators at loci enriched with ARA-lincRNAs compared with loci with NonA-lincRNAs, and suggesting that ARA-lincRNAs might act as scaffolds. In fact, a large number of histone marks were localized closer to ARA-lincRNA promoters than to NonA-lincRNA promoters (e.g., H3K4me3, H3K36me3, H3T11P and H3K4me3) (**Figure 6E**); also, marks representing enhancer signatures (H3K27ac and H3K4me2) are distributed more frequently in closer vicinity to ARA-lincRNAs than to NonA-lincRNAs (**Figure 6E**).

## Discussion

Characterization of lncRNAs is continuously being expanded, thus increasing our understanding of the biological relevance of each of the mammalian lncRNAs for diverse cellular mechanisms (St Laurent et al., 2015; Engreitz et al., 2016). LncRNAs are typically expressed in more restricted tissue patterns, and these features are consistent with their role as modular epigenetic regulators (Deveson et al., 2017). In this context, our work has focused on using deep-coverage RNA-seq for characterization of the lincRNAs complement in prostate cancer cells in the presence of androgen. We found 3979 novel lincRNAs not present in the human lincRNAs comprehensive reference transcriptome, which amounts to 57% of 7022 lincRNAs detected as expressed in LNCaP cells.

It has been show that the androgen hormone affects the expression of intronic antisense lncRNAs (Louro et al., 2007) and enhancer eRNAs (Hsieh et al., 2014). Here we provide the first extensive identification of 893 androgen-responsive lincRNAs in LNCaP cells. The majority of these lincRNAs (78%) were up-regulated by androgen, indicating an increased participation of lincRNAs in the androgen-controlled gene regulation program in LNCaP cells, compared with cells in the absence of androgen. Interestingly, 59% of these 893 androgen-controlled lincRNAs are novel lincRNAs identified in our study, and not previously listed in the comprehensive reference human lincRNAs datasets, which is consistent with the fact that lincRNAs generally play a role as specialized tissue-specific gene regulators in eukaryotes (Ulitsky and Bartel, 2013). In addition to the novel lincRNAs, our dataset of androgen-responsive lincRNAs included some of the previously known androgen-regulated lincRNAs such as *PCAT18* (Crea et al., 2014) and *DRAIC/PCAT29* (Sakurai et al., 2015). Moreover, we found a significant overlap between our list of androgen-responsive protein-coding genes and the known gene signature of hallmark genes in androgen response (Liberzon et al., 2015), which attests to the good gene coverage of our RNA-seq assay.

Interestingly, a total of 424 lincRNAs were detected as significantly differentially expressed in at least 75% of the patient cancer samples compared with adjacent non-tumor (**Figure 1I**), when we re-analyzed the prostate cancer data from Ren et al. (2012). This number is higher than the 137 lncRNAs that were found in the original publication to be differentially expressed in at least 50% of prostate cancers (Ren et al., 2012). This higher number of lncRNAs detected in the same samples is probably due to the use of a comprehensive reference set of lncRNAs, now including a set of prostate specific lincRNAs. In fact, among the 424 lincRNAs differentially expressed in the patient tumor samples compared with non-tumor samples, a total of 139 were novel lincRNAs identified here, and not present in the comprehensive list of lincRNAs in the public domain. This result points to the importance of characterizing lincRNAs involved in prostate tumor biology, and further studies may contribute to the definition of a robust, comprehensive signature of lincRNAs in this type of cancer, eventually permitting to define the degree of pathology of a given patient sample or to predict tumor recurrence after prostatectomy (Mortensen et al., 2015; Albitar et al., 2016).

It has been well documented that lncRNAs exert their regulatory functions by associating to a number of protein complexes (Khalil and Rinn, 2011), especially those that are involved in editing, reading and remodeling chromatin (Beckedorff et al., 2013b; Simon et al., 2013; Yang et al., 2015). In this context, little is known about the interaction of lncRNAs with transcription factors, especially in such complex regulatory events as the gene activation program induced by the AR transcription factor in LNCaP cells. Two lncRNAs, *PRNCR1* and *PCGEM1* were shown to bind to AR (Yang et al., 2013), although this result has been challenged (Prensner et al., 2014). The work of Hsieh et al. (2014) is an example of an enhancer lincRNA (eRNA), the monoexonic *eKLK3* that physically associates to the AR and regulates its neighbor *PSA* and *KLK2* protein-coding genes in the genome (Hsieh et al., 2014), opening the possibility that other eRNAs and lincRNAs might bind to AR and participate in the regulation of protein-coding gene expression induced by androgen.

The AR protein is comprised of stably folded globular domains in the C-terminal region, involved in hormone and DNA binding, and extensive regions with physical-chemical characteristics of intrinsically disordered proteins (IDPs) at its N-terminal domain (NTD) (McEwan, 2012). Intrinsically disordered regions (IDRs) can serve as the structural basis for hub protein promiscuity, promoting the ability of one IDP-hub to interact with many binding partners. Recently, using protein-RNA photocrosslinking and mass spectrometry on embryonic stem cell nuclei, the RNA-binding regions in ∼800 known and previously unknown RBPs were identified, many of which are transcriptional regulators and chromatin modifiers (Albitar et al., 2016); several protein domains previously unknown to function in RNA recognition, located in intrinsically disordered regions were detected, suggesting that many functional protein-RNA contacts remain unexplored (Castello et al., 2016). Also, it has been shown in HeLa cells that among the 1,174 RNA-binding sites within RBPs, nearly half of the sites map to intrinsically disordered regions, uncovering unstructured domains as prevalent partners in protein-RNA interactions (Castello et al., 2016). The AR-NTD has the propensity to change from an intrinsically disordered state to an a-helical conformation in response to a natural osmolyte or a co-regulatory binding partner (McEwan, 2012). It is known that AR binds to *HOTAIR* lincRNA, blocking AR interaction with the E3 ubiquitin ligase MDM2 (Zhang et al., 2015). Our work points to 619 androgen-responsive lincRNAs with enhancer-like characteristics that are significantly associated to AR (ARA-lincRNAs), of which 267 are novel lincRNAs detected for the first time in LNCaP cells; we speculate that these ARA-lincRNAs might be another yet uncharacterized set of binders of the AR-NTD intrinsically disordered region, exerting a co-regulatory function along with AR-NTD protein binding partners. The ARA-lincRNAs should be the first candidates to be explored as possible regulators of AR-induced transcriptional activation of protein-coding genes in their genomic neighborhood.

Based on the fact that the regulatory function of lincRNAs is frequently associated with the *cis* regulation of neighbor protein-coding genes (Marques et al., 2013; Hsieh et al., 2014; Ilott et al., 2014; Quinn and Chang, 2016), we related each of the ARA-lincRNAs with its neighbor protein-coding gene and found that the latter genes are enriched in GO functional categories, such as chromatin organization, cell adhesion, DNA transcription, and RNA processing. This suggests that in LNCaP cells these ARA-lincRNA/protein-coding gene pairs could participate in such cellular processes in response to androgen stimulation.

Those ARA-lincRNAs that have neighbor genes whose expression was increased in LNCaP upon androgen stimulation were enriched at their promoter regions with the H3K27ac histone mark, which is a mark present in active enhancer elements (Creyghton et al., 2010), when compared with the promoter regions of NonA-lincRNAs. In fact, lincRNAs with enhancer-like promoter regions have been described in other cell types, and the expression of these lincRNAs has been correlated with the expression regulation of their neighbor protein-coding genes (Marques et al., 2013; Ilott et al., 2014; Brazão et al., 2016). In this respect, it has recently been pointed out that in seven tested cell lines, the only histone mark consistently enriched at active lincRNA promoters compared with mRNAs was H3K9me3 (Mele et al., 2017); interestingly, a closer inspection of the data (Mele et al., 2017) shows that the H3K27ac mark is also enriched at active lincRNA promoters in two of the cell lines, and equally abundant at lincRNA and mRNA promoters in other two cell lines tested.

The ARA-lincRNAs are polyadenylated RNAs, most of them are un-spliced (median 1.0 exons) with an average 2.1 kb length. These are features that are common to known enhancers, which are typically un-spliced and either short (1–3 kb), bidirectional, and non-polyadenylated, or long (>3 kb), unidirectional, and can be polyadenylated or non-polyadenylated (Darrow and Chadwick, 2013).

It is known that besides promoting increased transcription of a number of target genes, AR can cause transcriptional repression of a set of genes (Prescott et al., 2007; Grosse et al., 2012), and the balance between AR activating and repressive functions is essential for maintaining prostate homeostasis (Zhao et al., 2012). The WDR5 and NKX3.1 transcription factors operate synergistically with AR to activate the androgen-regulated program (Gurel et al., 2010; Kim et al., 2014), and notably, the promoter regions of protein-coding genes whose transcription was repressed by androgen and were neighbors to ARA-lincRNAs had a decrease in the abundance of transcription factors WDR5 and NKX3.1, when compared with the promoters of protein-coding genes adjacent to NonA-lincRNAs. Nevertheless, the association of ARA-lincRNAs and androgen-mediated gene repression was not significant compared with control NonA-lincRNAs, suggesting that if the mechanism of repression should involve ARA-lincRNAs and depletion of WDR5 and NKX3.1, it would not be the only mechanism, and other factors should function as well for androgen-mediated gene repression independent of ARA-lincRNAs.

Chromatin architecture in eukaryotes is highly complex, and chromosome looping results in partitioning the genome into contact domains, which are associated with distinct patterns of histone marks (Rao et al., 2014), and define what are often called TADs (Dixon et al., 2012). TAD boundaries are enriched for the insulator protein CTCF (Dixon et al., 2012). Amaral et al. (2018) described a new class of lncRNAs, the topologically anchor point RNAs (tapRNAs), which are enriched at TAD boundaries, regulate developmental genes in *cis* and are related with the metastatic phenotype of cancer cells *in vitro*. Interestingly, we found that ARA-lincRNAs have a tendency to localize closer to the boundaries of a TAD, suggesting that they may participate in the protein complex that defines and maintains the border structural organization. We also found that protein-coding genes localized within a given TAD containing ARA-lincRNAs were more highly androgen-responsive than those within TADs not containing ARA-lincRNAs, which suggests that the tight and more frequent contact of regulatory elements within a TAD sub-compartment (Bonev and Cavalli, 2016) could spread the effect of the ARA-lincRNA to all elements inside that TAD. Accordingly, we found that TADs containing ARA-lincRNAs are more highly enriched in histone marks H3K27ac, H4K5ac, H3K4me1, and H3K36me3, all of them related to gene expression activation, when compared with the abundance of these marks in TADs not containing ARA-lincRNAs. Also, TADs containing ARA-lincRNAs showed a higher occupancy of LSD1 histone demethylase when compared with TADs not containing ARA-lincRNAs. It is known that LSD1 co-localizes with AR to demethylate H3K9 and de-repress AR target genes (Metzger et al., 2005) and that LSD1 binds to lincRNAs such as *HOTAIR* (Tsai et al., 2010). Our data suggest that the interaction between ARA-lincRNAs and AR may favor the co-localization of LSD1 and AR at the TADs where ARA-lincRNAs are transcribed.

Enhancer RNAs typically promote the engagement of transcription factors and bridging factors such as the Mediator and WDR5 complexes, where the activating RNAs direct the recruitment of specific complexes to target loci and modulate chromatin architecture, leading to transcriptional activation of the target genes (Orom and Shiekhattar, 2013). Overall, the ARA-lincRNAs described in the present work seem to act as *cis*-regulatory RNA enhancers that may cooperate with AR to control the androgen regulatory program of prostate cells. Further investigation of the molecular mechanisms involved with such interplay between lincRNAs and AR is warranted.

## Author Contributions

FB, LdS, and SV-A conceived and designed the experiments and wrote the paper. FB, MA, AA, VM, and AV performed the experiments. FB and LdS analyzed the data. ER, JS, and SV-A contributed reagents/materials/analysis tools.

## Conflict of Interest Statement

The authors declare that the research was conducted in the absence of any commercial or financial relationships that could be construed as a potential conflict of interest.

## Footnotes

1 http://verjolab.usp.br

2 http://verjolab.usp.br

3 https://www.proteinatlas.org

## Abbreviations

AR: androgen receptor
ARA-lincRNAs: androgen-receptor-associated long intergenic non-coding RNAs
NonA-lincRNAs: non-significantly-AR-associated lincRNAs
TADs: topologically associating domains
