# Supplementary material for: Chromatin Landscape Distinguishes the Genomic Loci of Hundreds of Androgen-Receptor-Associated LincRNAs From the Loci of Non-associated LincRNAs
Source: Front Genet. 2018 Apr 25;9:132. doi: 10.3389/fgene.2018.00132 (PMC5985396; doi:10.3389/fgene.2018.00132)
Supplement: Supplementary file 9 [file Table_9.docx]

**Supplementary Table S9 – The sequences of the 619 ARA-lincRNAs (comprising 1506 transcript isoforms) in FASTA format.**

>hg19_ct_ARAlincRNAs_9727_ARAlincRNA_0001.1 range=chr1:856646-857889 5'pad=0 3'pad=0 strand=- repeatMasking=none

GACACCAGCACATCCCTGACCCCAGCTGTGGCTGCCAGTGAGTTCCTGTG

CCTTCCTCTCCTGAAGTCTGTGTCTTCTCCCAGGTCTGATACCATGTGGA

AAGTCTGCTCCTGGGGCCAGCTGGAGGCCATGTGGACTGGCCCTGAGTGC

CCACTGTCTGGATGCAGCCCTGACCCTACTGCCCGTGTGTGGGCTACTAG

GTTCACCTGCACCTGACCCTCCTTCTAGGTTCGCCTGCACCTGACTCCCT

GATGTTCACACAGTGATTTGGAGAGGGAGGCTGGGGAGGCGCAGGTCTGC

ATGGCAGTGCCTCTCAGGGTCTGAGGCCCTTGGGCCAGGCCTCTCCTCTG

ACCTGGGCCAGCAGAGGGGCCTCCTTTCCCTTCCAAGAGCCCTCCCCTGT

TCACCAGCATCTCATCATGCTCAGGCCCACAGTGTGGTTAAGGAGGGGCA

TGGGGCTGGCCCTGGGTTTCTGGCTGGGTGTGGGGGCCTACCCGGGGCCC

ATGGTGTCTTCCAGAAAAGACCCTGACTGGGTCCAAAGCTGGAGGCACCC

CTCTATCCTCACTGTCCACTCCCTGATGTTCTGGAGGTCCCTCCAGGGGC

TGGGTGAGGAGGCCTTCCCCACCACCTCCTTGTCTTCCTGGGAAGCAGGC

ACCCGTTCAGAGGCCACGTTCCCACAGGACCAGGCTCCTCACCCCCAGCA

TCACCCTTTTCCACGGGGTTGTGCCCCTTCCTTGGTCCTGTCCACTCACA

CACACCCGTGTCCGTGTCCATGTTCACACCTGTTCTGTGTCCACTCCCAC

ACACCTGTGTCCACTCCCACACCTGTGTCTCGTGTCCACTCGCTCACACC

TGTACTGTCCGTGCCCACTCACACGTGTCCCGTGTCCACTCGCTCACACC

CGTGTCTCATGCCCGCTGGCTTACACCAGTCCCGTGTTCACTGGCGCACC

TGTGTCCCATGTCTTGTGTCTGCTCGCACATCTGTGTGATGTGTTCACTT

GCTCACACGTGTCCTCCACTACCACACCTGTGTCCCATGTCCACTCACAC

ACCTGTCTTGGTCTGGGACACCTGGGCCCCACTCTCCCCTGCACTCCTCA

GGCTGGCGGCAGAGGCCAGAGGAACGGAGTTCTGGCGCTGGAATCATCCT

GAGCGAGGCAGGGACCTGGCGGTGTGGACTCCACCGCCCCTCCTCCGGCC

CACCGAGCCCCGCCTGCCTGGGCCTTGGTGCCTCTGTCGCTGCT

>hg19_ct_ARAlincRNAs_9727_ARAlincRNA_0001.2 range=chr1:856646-859063 5'pad=0 3'pad=0 strand=- repeatMasking=none

CTGGGGGCTGCTCGCCGGGGTGTGAGCGTGCGCCGGCGGCTCTGGGACTT

TGCGCAGCGAATCTGATGGTGGGCGTTGGTGTCCGGGAGGGCGTGGGTGG

TCTCAGTGCACCTGCAGGGAGCGAGGTTGTGGATTTGTAGTGTGTTGCCT

GGGCAAAGTGTTCTGTGAATAACAGGGCAGCACCTCCCCGCCCACTCGTG

CCCCTTCCTATGCACTTGACAGGAGTGAGTTCGCCCGAGCCACGGAGTTC

CTGTTCAGTCTTTTGCAGAAGACGCTGAGACAGAAAGGTCAAGTAACTCT

CCTGAAGTCACACAGCTGTTTCAAATGCAGGATGTCTCACACCAGGACCG

CGCCAGGACCATGGTGTGGGGCAAGAGCTTCCTGTCACCCCACACCTCCC

AGCAGGAAGGTGGTGTGGATCCGAGGCAGGGTGAGGGTGGGGGATAGCGG

GTTGGCAGAGTGCACGGCCTCCCACTAGGAGGAGCAAGGCTAGTGAAGCA

GGACCGGCCACACCACGTCCACCTTGGTGCCTTCTGACAGGTCAGTCTGG

GCCCCAGTCCCTGGTTCCTGGTGAGCTTCCCATCACCCCCCACCTCCCTG

CGTGCCCAGATCGGCATTGCATCTGGAGCGTTCCGAAGAGAAACACAACC

TGGGGGCGTTGGTAACAGCAGCCAACCCGCGGAGTGAGGTCTGGGCCAGG

AGTGCTCTGCTCAGGCAAGGAACATGCCCAGAAGCCTCCCCCGCTATCCT

GGCCCTGTTCTGTGGGGGCTCGTTGGTCTGTTGGGGTCTGGGGGGCCAGG

TAGGAGTGTGGGTGTGAGTGTCGGTGACCCTTCCTTATGTGGCCACCCTT

GGCCCTATCAACCTGTCTGCATCCAGGACACCAGCACATCCCTGACCCCA

GCTGTGGCTGCCAGTGAGTTCCTGTGCCTTCCTCTCCTGAAGTCTGTGTC

TTCTCCCAGGTCTGATACCATGTGGAAAGTCTGCTCCTGGGGCCAGCTGG

AGGCCATGTGGACTGGCCCTGAGTGCCCACTGTCTGGATGCAGCCCTGAC

CCTACTGCCCGTGTGTGGGCTACTAGGTTCACCTGCACCTGACCCTCCTT

CTAGGTTCGCCTGCACCTGACTCCCTGATGTTCACACAGTGATTTGGAGA

GGGAGGCTGGGGAGGCGCAGGTCTGCATGGCAGTGCCTCTCAGGGTCTGA

GGCCCTTGGGCCAGGCCTCTCCTCTGACCTGGGCCAGCAGAGGGGCCTCC

TTTCCCTTCCAAGAGCCCTCCCCTGTTCACCAGCATCTCATCATGCTCAG

GCCCACAGTGTGGTTAAGGAGGGGCATGGGGCTGGCCCTGGGTTTCTGGC

TGGGTGTGGGGGCCTACCCGGGGCCCATGGTGTCTTCCAGAAAAGACCCT

GACTGGGTCCAAAGCTGGAGGCACCCCTCTATCCTCACTGTCCACTCCCT

GATGTTCTGGAGGTCCCTCCAGGGGCTGGGTGAGGAGGCCTTCCCCACCA

CCTCCTTGTCTTCCTGGGAAGCAGGCACCCGTTCAGAGGCCACGTTCCCA

CAGGACCAGGCTCCTCACCCCCAGCATCACCCTTTTCCACGGGGTTGTGC

CCCTTCCTTGGTCCTGTCCACTCACACACACCCGTGTCCGTGTCCATGTT

CACACCTGTTCTGTGTCCACTCCCACACACCTGTGTCCACTCCCACACCT

GTGTCTCGTGTCCACTCGCTCACACCTGTACTGTCCGTGCCCACTCACAC

GTGTCCCGTGTCCACTCGCTCACACCCGTGTCTCATGCCCGCTGGCTTAC

ACCAGTCCCGTGTTCACTGGCGCACCTGTGTCCCATGTCTTGTGTCTGCT

CGCACATCTGTGTGATGTGTTCACTTGCTCACACGTGTCCTCCACTACCA

CACCTGTGTCCCATGTCCACTCACACACCTGTCTTGGTCTGGGACACCTG

GGCCCCACTCTCCCCTGCACTCCTCAGGCTGGCGGCAGAGGCCAGAGGAA

CGGAGTTCTGGCGCTGGAATCATCCTGAGCGAGGCAGGGACCTGGCGGTG

TGGACTCCACCGCCCCTCCTCCGGCCCACCGAGCCCCGCCTGCCTGGGCC

TTGGTGCCTCTGTCGCTGCT

>hg19_ct_ARAlincRNAs_9727_ARAlincRNA_0001.3 range=chr1:857991-859063 5'pad=0 3'pad=0 strand=- repeatMasking=none

CTGGGGGCTGCTCGCCGGGGTGTGAGCGTGCGCCGGCGGCTCTGGGACTT

TGCGCAGCGAATCTGATGGTGGGCGTTGGTGTCCGGGAGGGCGTGGGTGG

TCTCAGTGCACCTGCAGGGAGCGAGGTTGTGGATTTGTAGTGTGTTGCCT

GGGCAAAGTGTTCTGTGAATAACAGGGCAGCACCTCCCCGCCCACTCGTG

CCCCTTCCTATGCACTTGACAGGAGTGAGTTCGCCCGAGCCACGGAGTTC

CTGTTCAGTCTTTTGCAGAAGACGCTGAGACAGAAAGGTCAAGTAACTCT

CCTGAAGTCACACAGCTGTTTCAAATGCAGGATGTCTCACACCAGGACCG

CGCCAGGACCATGGTGTGGGGCAAGAGCTTCCTGTCACCCCACACCTCCC

AGCAGGAAGGTGGTGTGGATCCGAGGCAGGGTGAGGGTGGGGGATAGCGG

GTTGGCAGAGTGCACGGCCTCCCACTAGGAGGAGCAAGGCTAGTGAAGCA

GGACCGGCCACACCACGTCCACCTTGGTGCCTTCTGACAGGTCAGTCTGG

GCCCCAGTCCCTGGTTCCTGGTGAGCTTCCCATCACCCCCCACCTCCCTG

CGTGCCCAGATCGGCATTGCATCTGGAGCGTTCCGAAGAGAAACACAACC

TGGGGGCGTTGGTAACAGCAGGTGAGACAGCCTTGTCCTGCGCTGCTGTG

CGGTGTTGCATCCACTAGAAAGGGGCCTCCTGTTCTGACCCTGGCCCCTG

CCCTTGAGATGCAGCTGGCATGTCCCTGTGGGCATCTCCCAGAGCAGGTC

CAGGGGTGAGTGGGCCACAGGCCTGTTTGCACAGGTGCCTTCTTCGAGGG

AGGGAACAGCCCACCACCACCTCCTGCCAGACCAGCTTCCTTGAGCATAA

ACCTGACCCTGGGTGACCACGCTGGGCTCCTTCACCTTATCTCACCAGAG

GCTTCTGCTGGTCCCACAGCCAACCCGCGGAGTGAGGTCTGGGCCAGGAG

TGCTCTGCTCAGGCAAGGAACATGCCCAGAAGCCTCCCCCGCTATCCTGG

CCCTGTTCTGTGGGGGCTCGTTG

>hg19_ct_ARAlincRNAs_9727_ARAlincRNA_0002.1 range=chr1:3695469-3696592 5'pad=0 3'pad=0 strand=+ repeatMasking=none

ACTCCAGCAGAGTCCTGGCCTGGGGAAGCCCCCGTGCCGCCTCAAGCCTC

CAGCAGGGCAGGCCCCTGGCCAACTGTGGCATCTGCCTTGACTGCCCAGA

GCAGCAGGAATCAGGTTTTGGGAGGACACAAGGCAAGGCAGTCATCTGAC

ACACCCACGGCAGTCCAGCTGGCACTGAGTGTGTGGGCCTCGTCCTGTCT

GGAGTCCCCTCCCTCCCACAGTGGGCGAGACGGGAAGGCCTGGCGGTGAC

CAGACAAGTGGGTCCAGGTCTCAAGGCAGCCTGGCCCATGTCCTGCAAGG

CTCTGAAGAGCCACCCAGCTGTTAGTCTGCCCACTGCACAGGGCCCCGGG

CCTCTCAGGTTGACAACTAAAGGTGTGTGGGCTGTGGGGCTCTTGCAGAT

GCTTCCCAAAATCACCAGTCATTTCTACAGACACAGAAATCGAGACACTG

AACAGAGAACAAATGGTGTCATTCGATAAAGCTGATGAGTCTGCTTCTCA

CATGGCACCCAGATCGTAAGGCAGTGGGGTCTGTAAAATCATCGCGGTGG

CTGGCCGTGTCCTCATGAGCCAAGCACACTGACTCCCGGGAGTGGTGTGC

ACGCACATGTGGTGCCTGGACATACACGAAGACACATGGACGTGGGGGTC

TGACCTGGACATTCACAACGGAAACGTCTACAACAGAAGGCGCCCAGGAC

CCACCTTTCTGGCGCATAAATGAAGATTTTTGGCTCAAAAACCTAAGCAT

CCTCCAGCTTTTGCTACCATCTGTCCCTCGGCTGGCTGAGGACTGCCGAT

GCTTCCCAAGCAGAGGCGTCTGGTCCACTCCTGGGGCTCCACCTGCCTGT

CACTGCAGCACCAGCGTGCACACGATGGAAACCCCACCGATGCCGCTCTC

CTCCGCTTGGCCCCGCTGAGCCAGGGTGAGGTGCTGCCACTGCCTTCAGC

CTACAGGCTGCTTGCCTGAAGGGGTCTCCACATCTCCACCCCCACAAAGC

AATCCAACAGCAGTATGAAGAGACGCAATTCTGCCAATTCTAAGGTCACA

TTAAAACTTTTCCCCCCAAGATTATGATCACTGCCAGCTGGAGAACCCAA

CGCCCTTGCACATGCCAAGCTGCA

>hg19_ct_ARAlincRNAs_9727_ARAlincRNA_0003.4 range=chr1:3816936-3820600 5'pad=0 3'pad=0 strand=+ repeatMasking=none

GCGAAGGTCACAGGCCTTGGGCCAGTGGTGGGACACTGGAGCAGGAAGTC

GGCCTGTGGCCGGCCATGTTCATGAGGGCGCGTCCCTTGGCACCCCTGTA

GCCGCTATGTTTCCCACGCCCGTGGCAGGCCTGAGAGATTATGTTTGCGG

GGGGTGCTTTTTTTGTAGCTTCTGTGTTCGCCATATTTGAAAGGGGCAGA

CGGCCATGTTCGTGAACGGCGCTTCCCTTGGTGCACCTGTGGCCGCCATG

TTTGAGCGAGGACCCCGTGGCAGGCCAGGACCGTTTCTCCGGTGCTATCC

CAGAGCATGGCGCATCCACTCATTCACTCAAG

>hg19_ct_ARAlincRNAs_9727_ARAlincRNA_0003.5 range=chr1:3816936-3823432 5'pad=0 3'pad=0 strand=+ repeatMasking=none

GCGAAGGTCACAGGCCTTGGGCCAGTGGTGGGACACTGGAGCAGGAAGTC

GGCCTGTGGCCGGCCATGTTCATGAGGGCGCGTCCCTTGGCACCCCTGTA

GCCGCTATGTTTCCCACGCCCGTGGCAGGCCTGAGAGATTATGTTTGCGG

GGGGTGCTTTTTTTGTAGCTTCTGTGTTCGCCATATTTGAAAGGGGCAGA

CGGCCATGTTCGTGAACGGCGCTTCCCTTGGTGCACCTGTGGCCGCCATG

TTTGAGCGAGGACCCCGTGGCAGGCCAGGACCGTTTCTCCGGTGCTATCC

CAGAGCATGGCGCATCCACTCATTCACTCAAGCCCACCCTCTCCTATCCT

CTCGGTCCACCCGACTTAGCTGGGCAAAAATAACCCTGTGGTAGGACAGC

CGTCTGCTGTTGGAAAGTATCGCAGCTCCTCCCGTGTCTGCCCCGTGCAC

ATGTGCCAAGGTCCTGCCTTGAATGAAGGACACAGCGGAGAAGGATGTAG

GCTCTGAGCCTGCTAGGGTGCCTGAGAGACTCACAGGACTACCCCTGGGG

CAGTTGGAAGATGCCCAGAAACTTCAGGGAGCCACCAGCATGGGGCAGAG

GGTCTCTTCTGGAGTTGGTGGCATCAGAATCAGGTGTAGAGTACATAGGA

ACTGTTGGCGCCGTGCAGAGATGGGGAAGGAGGTGCCGGTGGAAGGGCCC

AATGCCTGCGCCCCTGGGGCCCTCACTGGAGGCTGAGCATCCACTGGACT

CTTGTAGCCCGCTTTGTAGACTGATCAGTAGTCTGTGACATGACGATGTT

TGAGTAGAGTAGACCATTTTCTGCTTCATATATCCTACAACGTGCTGGGA

TCAGGAGGCAGTGGTGGGTCCATGGCATCTGACCCAGCCCGACGCCCTTG

TGGTCTCCAGGCGGTCATGACCCATGGCCTGACCCGGCTTGGTCAGCCCG

GCCTGGCCGTGTCTTCCCAGGTTCACACCCAGCCCTGGCCCCTCTCTGCC

CCCTTGCCAGGAAATTTGAGTCCTCCTTAACGTTTCTCAATATTGTGGTT

TTGTTTTGTTCTTTCTCATTCATTTGTCTTTTAATTTTTGGCTTCCGTTT

TTTATGGGCACCTAATTGTGTGTGTTCTAAAAATTGAATTGTGCTTCTGT

TCTTATTGATGGCAGCTAACCATCACTGTGTAAGTAGGCAGGATGTGGAG

GTCTACCTAGGAGAACTCGCTTGCCTCTGCTTTTCACCGACGCAGGCCCG

GTGGAGATGCTCCAGCGGAAGCCAGGACTTGTCTCCTGGTGGAGTCCACC

GGTTTCATTCATCAAAAGTTTGTTGGGCTCTCTGCACACACGTTGTAAAG

AGCACTGCAGGCAGTGCACAGGCAGGGCTGTGGGAGCTGTGGGCGTGGCT

CCCGCCAGGGTGGAGCTGCACCCTCCATGGGGAGTCATGGTGCATGTGCA

CAGCTGCAGAGAGAGGCTGCAGGGCAGGAGAACCTCGGGAACCCCGTAGT

CTCCGCTTGCCCTGACTGTATGAAGAACATGCTCCATTTGGGTATCAGTG

AAACTGCACACCAGGGGGGAACATTCAAATCGTGAACTCCAACATCATCC

AGCAGAAAAGCTCCGAGCGATGGGCCACTTTGTGCCTTGCTGCCAGCCCT

CCGCTGGGATCACGCTGCAGTGTCCTTTGAAGAAAAGGCCTGGGATTTGC

AGAGCCTGGAGAAGGCTAAATTTCCAACTAGATGAAAAAAAAAATTGCCA

TTTTATGAGAACCTTTGGGCAGTCTGGAAGAAAAACACCAAGATGAAAAG

CCCTATTTTGAGAGCTCCCTTGCCTTCAAGCAGGGGCCGCTGGCAGTGGA

AACCGGCTGGGGATCGGGCTGATGGCAGAGTGAGGTTCAGGCTGGGAGCG

GGCATGTGGACAGGTCCCTCCCTGCAGATCCACGTCTGCCTCCCACTCGC

AGATGCCCCTGTGCCCAGCG

>hg19_ct_ARAlincRNAs_9727_ARAlincRNA_0003.1 range=chr1:3816936-3830854 5'pad=0 3'pad=0 strand=+ repeatMasking=none

GCGAAGGTCACAGGCCTTGGGCCAGTGGTGGGACACTGGAGCAGGAAGTC

GGCCTGTGGCCGGCCATGTTCATGAGGGCGCGTCCCTTGGCACCCCTGTA

GCCGCTATGTTTCCCACGCCCGTGGCAGGCCTGAGAGATTATGTTTGCGG

GGGGTGCTTTTTTTGTAGCTTCTGTGTTCGCCATATTTGAAAGGGGCAGA

CGGCCATGTTCGTGAACGGCGCTTCCCTTGGTGCACCTGTGGCCGCCATG

TTTGAGCGAGGACCCCGTGGCAGGCCAGGACCGTTTCTCCGGTGCTATCC

CAGAGCATGGCGCATCCACTCATTCACTCAAGGGAAAGGCCTGAAAGTAA

AATTGTATCAACATTAGGTTTGGCTAATGTTGCCATTAGGCAAACCTAAT

TTTAATGAAGCCAGGAGGTTCTCGTTTTGGGCCTTGACTGGACAGGTTTG

AGCTAGAAACCACTTGAAGTATTGATCATCCATCTGATCATCAAGTTTTT

TTTTTTTTTTGAGTTGGAGTCTCACTCTGTTGCTCACGCTGGAGTGCAGT

GGCACGATCTCAGCTCCCTGCAACCTCCACCTCCCGGGTTCTCCTGCCTC

AGCCTTCCAAGTAGCTGGGATTATAAGCGTGTGCCACCATGCCTGGCTAC

TTTTTTATATTTTCGGTTGAGATGGGGTTTCACCGCGTGGCCAGGCTGGT

CTCAAACTCCTGACCTCAAGTGATCCGCCTGCCTCGGCCTCCCAAAGTGC

TGGGATTACAGGCGTGAGCCGCCACTCCCAGCTTGTTCATCCAGTTTTTA

TTGAGCATCTATTATGTGCCGATATTGAGGTAGACTCTGGGAGTCACAGC

AGTGAATGCAGCTGCCTGGATGCCTCGTGAGGGCCGGAGCCTCACTGGGG

AGATGGACATGAACAGCAAATGCACAGCGAG

>hg19_ct_ARAlincRNAs_9727_ARAlincRNA_0003.2 range=chr1:3816968-3833789 5'pad=0 3'pad=0 strand=+ repeatMasking=none

ACACTGGAGCAGGAAGTCGGCCTGTGGCCGGCCATGTTCATGAGGGCGCG

TCCCTTGGCACCCCTGTAGCCGCTATGTTTCCCACGCCCGTGGCAGGCCT

GAGAGATTATGTTTGCGGGGGGTGCTTTTTTTGTAGCTTCTGTGTTCGCC

ATATTTGAAAGGGGCAGACGGCCATGTTCGTGAACGGCGCTTCCCTTGGT

GCACCTGTGGCCGCCATGTTTGAGCGAGGAAAGCCAGGGGTCTGTGCGGG

GATGCTGCAGGACACGAGAACCAGCCAAGACCTATGGCCCCTGCAGCCAC

CGAGGGCTCGAGAGCGCCCCTGTGGACTTGGCTGGGCCCTTCATGGTGAC

ATCCAGGAATGCGGCTGAGCGCGGGTGTTGACCTCGCTGTTTGCTGGAAT

GTTTGGGTTCTTGGACCATGTCAGTGACGGCTGTGTCACCATGGCCTTGC

TATCCCCAGGAAAACCTAACATGCACCCCGCGCCTGTCCTCTGGGACTCC

CATCCTCCTCCTTTGTCTGACCCCTGAGAGGCTTACCGTTTCCCCAGGGC

AGCCCCGGCTGTGGCCCTCGGCCCTGCTCCAGGCGGCCATGTCAGTGTCC

TCCCTCGGCGCTCCCAACACCCTACCTGGCTCTGCGATGGCCACCCTGGA

CTCCTAAACACCCCAGAAAGGGAAGAGATTTAAGAGGAACCTTGATTCCT

GCCATTCTGCGCAGTCGTGGGTCCCTCCTCCACCTGGGGCCCTCAAGGGG

ATTTCTGTCCGCACTCCCAGCCAGCGACCCCTCCCAGTTCCTGCAGCTGG

AGCGGCCCTCTCTCCTGAACATTCGGGCACCTGCGTCTGTCACTGCAGCC

AGAGGGCTGGAGTGTCTTCGGTCAACTCGCCATCCCTGCCACGGCTGGAG

GCCACATGTCCCAGAACCTACCTTGCTGGGATGGGGGTTGGGGTGGCAGA

GGCTGAACGCGTGTGAGGTTTGGGGGAGGACAAGCGGAGCGGGGATGTGT

GGGAGGTCCTGGTGTCCACCGTGAGGATGGTCACACCCCATAGCTTTGTT

GATGCTGTGGCTGTAGCAGGCCCAGTGGCTCCGCTGAGCCTCCCTAGCAC

CAGTGCCCAGTGAAGTGTGGGGGGCTGGACATAGGTGGCCTCAGTTCCTG

GGACTCCCCCAAAGCTCTGGTTTCCCCCCTGCTCCCAGGCTTCAGGTGGA

CGAGTTAGTGACCACCCCCACTCCAGACCTCCCTCCCCTAGCCACCCCCA

CAGTTATAAAAACCTTCATTCTCATATGGAACCCCCTTTCCTGAAATCCG

TAGAGTGACTGACTGCTTTCTTGAGTGAATCTGGACTGGGCCACATGATG

ATCGCTGTACAGAGCAGCTGAGCTCCTCTGTCTCAGCCTCCCTGAGTTCA

CAGCAGGCTCTGGGCATCATCTCCGTGTCATCCTAAGGCCACGGGCGGGG

TTCCCACCAAACAGGAGAGCAGCTCTCCCGAGATGAAGCCTTCTGATAGC

CCTAGAACCAAGAGGAACCGTGTGGGGTTGGGTGGGGTGTTTACTGTGCA

CTCCTGATGTTCCCTCCCAGTGAAGGACACCCACCTGGGACACTGTGGCC

CCTGGCCCTCCCTCCCTCCCCTCGGTGGCAGAGAGAACTTCCTGGTGGGT

GACAGCCCATGGTCCACCCTTGCCAGGTGGATTCAGGCAAAATACGCCAG

GCTGTGTGCTGAAGGCATCCCATCTCTTCCTCGTCCCCTCTCGGCTGGAT

CGGGGTGGGGCAGCGGGTGGTGAGTGTGTCTGTCCTGCCAGTTCAGCCTC

CAACTGGTCTGCTGTGGGGCAGGAGCCCACCTGGCTCTCCTGCAGAGCTG

CATGGCCTGCCTTGCCTCACCCGTGACAACAGAGACTTTGGTTCTCCATC

TGCAGACGCATTTGTCTTGTTTCTTATTCGGTCCAGAACTCGGGTGGGAA

GAAGGGTGATGTTATTTGGGTCCCCTCAAGACCTTGACAACAGATAGTTT

TTAATATCACATTTTAAAGCCGCCAACTTTCTCTCCTCCACTTTGGTATT

TCCCTGATTTTTAAACAGAATGTCGGCTCTGGAGGCAGAAAGCTTGGGCT

TGGATCTGGGCCCTGCCACGCAGTAGCTATGTGGCCAGGGAATACATAGC

TTCCGGATCTCCGATCCCTACAGTAAAGTACAGATAAAAATACTTTTCAT

TGATGTGTTTGAAATCGAATGAGATAGTTAATGAATGAGTAAGTGCTCTG

CAAACTCCAGAGCGGGGTGCGCGTTCTGATCTGTTTCATAGAATCTGACA

CGTACCCTTTCCCACCCCAGCGTCTCTGAATTGGGATGCATCTGACAGCA

AGTGTGGCATCCGGGCTGCAGTTGCCGTTGTCTGCTCACATGTGAATTAA

AAAAACAATCTCAGCATATGAAATCTCTAATCGCGTATGAACTTGGTGGT

TATTCCTGGTGCCGTGTGGATAACTGCAGCCTCAACACCCCAGTCCACAA

ACCACGTACGGACCAACTGAGGAAGGAGTAGGGGTTCTTGTTGTTGCAGA

AAACCCTCCCTCAACTTGCTCTAGAAGATACCAGCATTCATACTTGGAGT

GGGCATCAGCAGCTTGGAAGACACAGCAGTGGGCCCCTCTTAGCAGGAGT

GCCCCATCTCATGGCTCCCGACGACGACCCAGAGGGTGATGCTGCGTAGG

GGCTCACGGACATTGGCACTCTAAGTCAGAGATGCTCAGGTGAAGGGGGC

TCTGATGGTGAGGACATTCAGAAATAACAGAGATTGGCTGGGTACGGTGG

CTCACACCTGTAATCCCAGCACTTTGGGAGGGAAGCCGAGGCAGGTGGAT

CACTGGAGGTCAGCCTGGCCAACATGGTGAAACCCCGTCTCTACTAAAAA

TACAAAAATTAGCTGGGCGTGGCGGCATGTGTCTGTAATCCCAGTTACTC

GGGAGGCTGAGGCAGGAGAATTGCTTGAACCTGGGAGGCAGAGGTTGCAG

TGAGCCAAGAGTGCGCCACTGCACTCCAGCCTGGGCAACAGAGCGAGACT

CTTTGTCTC

>hg19_ct_ARAlincRNAs_9727_ARAlincRNA_0003.3 range=chr1:3816980-3832011 5'pad=0 3'pad=0 strand=+ repeatMasking=none

GAAGTCGGCCTGTGGCCGGCCATGTTCATGAGGGCGCGTCCCTTGGCACC

CCTGTAGCCGCTATGTTTCCCACGCCCGTGGCAGGCCTGAGAGATTATGT

TTGCGGGGGGTGCTTTTTTTGTAGCTTCTGTGTTCGCCATATTTGAAAGG

GGCAGACGGCCATGTTCGTGAACGGCGCTTCCCTTGGTGCACCTGTGGCC

GCCATGTTTGAGCGAGGACCCCGTGGCAGGCCAGGACCGTTTCTCCGGTG

CTATCCCAGAGCATGGCGCATCCACTCATTCACTCAAGGGAAAGGCCTGA

AAGTAAAATTGTATCAACATTAGGTTTGGCTAATGTTGCCATTAGGCAAA

CCTAATTTTAATGAAGCCAGGAGGTTCTCGTTTTGGGCCTTGACTGGACA

GGTTTGAGCTAGAAACCACTTGAAGTATTGATCATCCATCTGATCATCAA

GTTTTTTTTTTTTTTTGAGTTGGAGTCTCACTCTGTTGCTCACGCTGGAG

TGCAGTGGCACGATCTCAGCTCCCTGCAACCTCCACCTCCCGGGTTCTCC

TGCCTCAGCCTTCCAAGTAGCTGGGATTATAAGCGTGTGCCACCATGCCT

GGCTACTTTTTTATATTTTCGGTTGAGATGGGGTTTCACCGCGTGGCCAG

GCTGGTCTCAAACTCCTGACCTCAAGTGATCCGCCTGCCTCGGCCTCCCA

AAGTGCTGGGATTACAGGCGTGAGCCGCCACTCCCAGCTTGTTCATCCAG

TTTTTATTGAGCATCTATTATGTGCCGATATTGAGGTAGACTCTGGGAGT

CACAGCAGTGAATGCAGCTGCCTGGATGCCTCGTGAGGGCCGGAGCCTCA

CTGGGGAGATGGACATGAACAGCAAATGCACAGCGAGGAAAGCCAGGGGT

CTGTGCGGGGATGCTGCAGGACACGAGAACCAGCCAAGACCTATGGCCCC

TGCAGCCACCGAGGGCTCGAGAGCGCCCCTGTGGACTTGGCTGGGCCCTT

CATGGTGACATCCAGGAATGCGGCTGAGCGCGGGTGTTGACCTCGCTGTT

TGCTGGAATGTTTGGGTTCTTGGACCATGTCAGTGACGGCTGTGTCACCA

TGGCCTTGCTATCCCCAGGAAAACCTAACATGCACCCCGCGCCTGTCCTC

TGGGACTCCCATCCTCCTCCTTTGTCTGACCCCTGAGAGGCTTACCGTTT

CCCCAGGGCAGCCCCGGCTGTGGCCCTCGGCCCTGCTCCAGGCGGCCATG

TCAGTGTCCTCCCTCGGCGCTCCCAACACCCTACCTGGCTCTGCGATGGC

CACCCTGGACTCCTAAACACCCCAGAAAGGGAAGAGATTTAAGAGGAACC

TTGATTCCTGCCATTCTGCGCAGTCGTGGGTCCCTCCTCCACCTGGGGCC

CTCAAGGGGATTTCTGTCCGCACTCCCAGCCAGCGACCCCTCCCAGTTCC

TGCAGCTGGAGCGGCCCTCTCTCCTGAACATTCGGGCACCTGCGTCTGTC

ACTGCAGCCAGAGGGCTGGAGTGTCTTCGGTCAACTCGCCATCCCTGCCA

CGGCTGGAGGCCACATGTCCCAGAACCTACCTTGCTGGGATGGGGGTTGG

GGTGGCAGAGGCTGAACGCGTGTGAGGTTTGGGGGAGGACAAGCGGAGCG

GGGATGTGTGGGAGGTCCTGGTGTCCACCGTGAGGATGGTCACACCCCAT

AGCTTTGTTGATGCTGTGGCTGTAGCAGGCCCAGTGGCTCCGCTGAGCCT

CCCTAGCACCAGTGCCCAGTGAAGTGTGGGGGGCTGGACATAGGTGGCCT

CAGTTCCTGGGACTCCCCCAAAGCTCTGGTTTCCCCCCTGCTCCCAGGCT

TCAGGTGGACGAGTTAGTGACCACCCCCACTCCAGACCTCCCTCCCCTAG

CCACCCCCACAGTTATAAAAACCTTCATTCTCATATGGAA

>hg19_ct_ARAlincRNAs_9727_ARAlincRNA_0004.2 range=chr1:4036189-4069751 5'pad=0 3'pad=0 strand=+ repeatMasking=none

GCTTTTTGGTCTTTCCTGCTGCCTTGTGAAGAAGGTGCTTGTTTCTTCTT

TGCCTTCTGCCATGATTGCCATGTGAATCGCACCCCTGGAACTGTGCAGG

GCCCACCTGTGTGGCCACACGCAGTGACCCTGAGCAAAGGAGTGACCACA

GAAGTGCATGGCAAAGCAGAAAGGTGTGACGTCTTGGAGGACGCGGGAGG

AAAGCACGCCCAGCGGACAGGAGCCATCACGCGGCTGCGGGCTGCTGCTG

AGAACCTGGCGTGGGCCACCGTCGTCAGCAGCGTGGAGGAACGCATATGC

TGCCTCCTTTCTGA

>hg19_ct_ARAlincRNAs_9727_ARAlincRNA_0004.1 range=chr1:4037028-4058260 5'pad=0 3'pad=0 strand=+ repeatMasking=none

GTGCATGGCAAAGCAGAAAGGTGTGACGTCTTGGAGGACGCGGGAGGAAA

GCACGCCCAGCGGACAGGAGCCATCACGCGGCTGCGGGCTGCTGCTGAGA

ACCTGGCGTGGGCCACCGTCGTCAGCAGCGTGGAGGACACTTGTTATTGC

AGTCAGAGTCCATCTGGATAATCCTGGATAAATCTCATCTGGAGATCCTT

CACCAGATCACATCGGCAATGACCTGTTCCCCAAATAAGGCCCTGTGCAC

AGGTTCTGGGGTGCTAGGAGTGAGCCACCATGCCTGGCCCAGTTTTTAAT

GCTTGGACATCTCCAAAATCAGGACATATCTTGTAATTGAAGACGTCTCA

CCATAGCGGCCAGCCGTGCAACCACTGGGCCTGTCTGTGCCTGAACAACA

CACTGG

>hg19_ct_ARAlincRNAs_9727_ARAlincRNA_0005.3 range=chr1:9207989-9262678 5'pad=0 3'pad=0 strand=- repeatMasking=none

AGCATCCAGGCAGGACGGCAGCAGCTGAGCAGAGGAGAGAGGAGGAATGA

GTCCCCCGTTCTCACCCCGGGGAGCGGTTGCCTCGTGAGTCCAAGGAGAA

TCCGCCCTTTCGTTTTGCGCAGGTGGAGATCTCGCGGGCCCAGTTCGGAG

TCCTGCAAACTGTGGAGGAGATGCCGCTGTCCCGTCGGTCTGGGGACAGC

CCAGCTCCCCGGATCCCGGGCTGGAGAGACGCGTCGCGGCCCCGGGGCCT

GGTGGCACGAGCAGGAAGGAGGACCCGGCGGCGGGCTCTGCCTGGGCTTG

CCTGGGCTTGTTCCGAGCCGGGCTGCTTCTCGGTGACCACGCAGATCGGG

GGCATTTGGAGATTTTGCGGGAGTCCTGCAGCCAAGCTCCGGGGCAGGAG

AGGCCTGGAAGCCTGCACTACCTGCTCGCCCCGTCCCAGCATGCACCCAG

GTAAACGCTTGTGTTTCTCAGTCCGTGCGAAAGTTTGCAAAGAAGGAGGC

GGGAACTAGACCAACAACTTTAATAATCATAGTCATAATAATGAAAACCC

TGTTCAATTGTGTAGCCTCCGTAAGGGGAAGAATTTCCAAGAAGCAACAA

AATGTTCTTTGATTTCATAATCTCTGGGGAGAAAGGATTCCTTTTTTGGG

GTTTTGCTCGGCTAGTTCTTTCTTCCCTCCTCTATCTCCAGCGTGGGGCG

AGGAAGGCCGACTGGTTCATGGGTGGCAAATGGGGACCCTGGCTCTCTTG

CGCTGCTGGCCACGGTGATATTGCAGCAAATGCTCAACCTCCCCAGTTCC

TCTCCTCACCTTGAAAGTGGAGGCATTGATACTAGCCACCCATGGTTGCA

AAAAACTCGCATGAAAGGGAAAGGAAAAGTGCTTCGGTCACTAAAGTAGC

GTTGTCTAAGAGGTCAAGGCTTTGGAAGACTTTTGCTCTAGAAGAGGGGA

ACGGGAGAGGGGCTTTCAGCCAACCTGGCCCCGAGGTGAACGCGCGCGCC

CCCTGCTGGCTGGAGCCCTGTCCAGCCTGTGAGCCGCCAGTGGGGAAGGA

GACTGAGGGGAGGTGGTCCCTGGAGTTCCTTGCTCCTGGCCTGGAGCAGC

AGGTGCCAGTGCTCCTGAGGCCCCCTCTCTCAGGTTACGTGGATGGGGCC

TGTAGCACAGACAGGGCACATGCTTCAGCACCTCCCTGTTTCTGCGTCAG

GTGGGGGACATGAGCTAGGCTCTGCAGAAGGTTCCAGAGCCTGGGCCTTC

CTGTTGCATCCACACTAGGCTCCTGGAGCCCAGAGGTGGCTGGGATCTCT

GCATACATGCATGTGAAACTCTAGCAGATACCAAGTCATGTTAAGGGGGC

TAAACGGCCCACTGCAGTTATGTTAGCCAGTTCTGCCTCTGCCCAGTTCT

GAGATATCTTTGGGACAAATGGCCACTCCTCTGAGCCCCTGTGTCTTCCT

GCATGAGCCACAAATGCTGTGTAGTCATGGCTGAGGAAGAGGGAAGGTAC

CTGTGCTTGCAAACATTCCTCTTGAAGGACTATCCAGGAGCAGAGGGAGC

AGCTGGGGGCAGCATCAGTTGGTACCTGAGCCCATTGGCACCTACTGCCA

CATGGGGTAGTGGCCTGTCTCCTGAGCTTCCCCAAGCTGGCCAAAACTGG

CCAGGACTGCCCTGCTCTCCCCTCCCAATCCAGCTTTCTCTTGGGCCTCC

AGGATCCCTTGGCTAGCTGGTGTGGTGGCCATGAGGACTGCACTTTTGCA

CCCCACCCCTGCCCTTGGCACACATCGCAAATCAATAATGCTAAACCTAG

ACAGGCCCCCAGGTCTTTCTGATCATAGCATCCATGCAGTGCTTCCCATT

GATTGTGTCACCTAATTGCCAACCCAGCCCTACACCTGCCCTTGTAACTG

GTCCCTGGGCCGGGGGGTGTAGGAGGATGCAAGGATGAACAAGATGCTGC

CCTTGGTTATAGGATACACTACCTCCAGCTAGAGGTTATCAGATCTGTGC

ATAAGAAAATAACTAGGCGGGACTCATTCATTCATGCGACAAATATTTAC

AGAGTCCTTGCTATACGCAATGAGTCATTGAGGAGAAACCTACAGAAGGA

TGAGGGGGACGGCTTTTCAGGCAGAAGGGTTAGTAAGTGCAAAGGCCCTG

TGTTTGGTGCATTGGAGAAATTGCAAGAAAGCGGGTGAGACTGAAGATCA

ACTCGTGATACTTGGTAATGAATATCACATGAAAACAAGGAAGTTTTCTT

GTCTAGCTCTGAAATTTGCCTTGGTTGGCAGATCCTTCCTTCCCAGAGAG

CACACATCAGTACTGCAGCTACAGCTGTCTGTCTGACATACTTGTAAGAA

CGAACTGTTTAGTGCAGGCTCTGGGAGGCAGTGCCATGCGGGTCACTGAC

ATACATGGTGCCAATCATTTTTCTCCATTCGTAAGGCACCTCAGGACCCC

ACTGGGGCCCCAGTGTTCCAGTAAGGATACGGGTTTTGAGTTAAGGACCC

GCCTTCCAGCTCCGTCTCTGTCACTAGCTGTGCTAATTTGGAAATAGGAC

TTCCTTTCCTGGCTTTGGTTCCTCATCTATAAAAATTGCCCTTATGTTAG

GACCTACTTGGTAGACGCATGAGATAAAATAATTCGAGAATGTGCATAAA

CCAGGGAGCAGGGTGCTGTGGCAACTATTAGGTAGCATCAGTGATAAAGC

ACTTTGCTTTCATGGATATTTTATGTGTTCACTAAATCAGAACTTTCCTC

TATGCTGATGTAGTCTAGTGAGGTCTGGGGAGTTTCTTGTTTGATTTTGC

ATTGCAAATATTCCTCTCTTCCCCTCCCTAAAAAACCACTGCTTTTTTTT

TTTTTTTTTTTTTTTTTCTGAGACAGGGCCTCACTCTGTCACCCAGGCTG

GAGTGCAGTGGCACAACCGTAGCTCACTGGAACCTGGAACTCCTGAGCTC

AAGCGATCATCCCGCCTCAGCATCCTGAGTAGCTAGCACTACAGGCATAT

GTCACCATGCCCAGCTAATTTTTAAATTTTCTGTTGAGATGGGATCTTAC

CATGTTTCCCAGGTTGGTCTCAACCTCCTGGGCTCAAGTGATCCTCCCGC

CCCAGCCTCCCAAAGTGCTGGGATTACAGGTGTGAGCCACCTCGCCCAGC

CTACATTTTTTTTTTTCATTAACTAGTATTGCAAATATTTAAATGGAGTA

AAATGCCTTGAGTTGATGCCAAAAGGAAAAATAAGGGTCTATAAGGGGAT

CCATTCTGACTGAAGCATGACACGGTTTGAGTACGCCCTCTCCCGTGTCC

CTGGCTGTGGGTTACCACGCCTGGGGCAGGCAGTGGCACCTGGGGAAGGC

TTGGCAGAAGCTCCTGCCTCTCACGCTCATAGACCTTTTGACCCACCAGG

CTCACCCCAGGGCCCCGCTCTGACCTCCGTGGCACCAGGTGACTCTTACT

CTGCACTGTCATTTGGGGCCCTCAGCCATCCCACCTCACCCCACACGGTC

CCAGCAGCTCCTGCCCGATTCCCATGCCTCCTCTGGACAGAGAGCTCACA

CTCCTCTTGCCAGATCAGTAACTCCTTATGAGGAAGATGGTTTCCATACT

CAGTAGATGGCACAGTGGGGGCAGCAAGGGAGGGTCTGCCAGAGGAGACA

GAGCTGGCCTGACAGTGGTGGGGGTTGGGGGTGTCTGTTGACTTGTTGAA

CCAAATTGATTCCTAGTCCTGGCATTAAACATCCTGCGAGGCTGTCACCT

CATCTCAAACAAGATTAGTTAAAACGGACTTGAATTGAAAGTGGGTAAGA

CTGCTGTCGCTGTCACCCCCTGTTACTCTGCAATCCACCACCAGTGAGGG

GTGAAGCGAGTTTAAAATCCTGGGCCACATGGTCGACCACTGATCAGTCC

TCGTCCATGATCACCACAAGGAGAGTGGTGGTGAACTGGCTTCCCAAACC

CCAAAGCAAATAGAAAGCCCTCACCTTCAGGGCTGGGTGCGGTGGCTCAC

CTGTAATCCCAGCACTTTGGGGTGCTGAGGTGGGCGGATCACTTGAGGCC

AGGAGTTCGAGACCAGCCTGGACAACATGGTGAAACCCCTGTCTCTACTA

AAAATGCAAAAATTAGCTGGGCGTCATGGTGCGCGCCTGTAATCCCAGCT

ACTTGGGAGGCTGAGGCAGAAGAATCGCTTGAACCCGGAAGGCAGAGGTT

GCAGTGAGCCGAGATCATGCCACTGCTGCACTCCAGCCTGAGTGACAGAG

CGAGACTGTGTCTCAAAGAAAAACACAAAAAGAAATCCCTCACCTCCAGC

CTGAAAGACAGAAAAATAGTTCTTGATAATTTGCTGAGAAAAAAAACATT

GTTGAATTTATCACAACCTGGCCACCACCCAGCCACCCCCTGCCCTGGAC

ACCCAGCCTCATAGTGCTGGGTGGGAGGTAGAGAGACCCTGCTGGCATTG

GAGGACAGCTGCGATGGTCTTTTAGGGAGAAGGTGAGACAGGCCCAAAGT

AATACAGTCGAGCCTCTTTACCTGGGGGTTCCACATCAGCTGATTCAGCC

AGCCTAGGGTCCAAAATGTTTGAAAAAGCAAAAATAAATAATAATACAAC

AATAAAAAATAATATAAAAGGGCCGGCTGTGGTGGATCATGCGTGTAATC

CCAGCACCTATGGAGGCCAAGACTGGAGGATTGCTTGAGCTCAGGAGTTG

GAGTCCAGTCTGGGCAACATAGCAAGACCCCTGTCTAATAAAAAAATAAT

AATACAAATTAAAAAACCATGCGGTATAACAACTATTCACATGGCATTTA

CATGGTATTAAATATTATAAGTAATCTAGAAATGATTTAAAGTGTGTGGA

AAGGCCAGGAGCAGTGGCTGACGCCTGTAATCCCAGAACTTTGAGAGGCC

AAGGTGGGTGGATCACCTGAGGTCAGGAGTTCGAGACCAGCCTGGCCAAC

ATGGTGAAACCCCGTCTGTACTAAAAATGCAAAAATCAGCCAGGTGTGGT

GGCAGGTGCCTGTAATCCCAGATAATCGGGAGGCTGAGGCGGTAGAATCG

CTTGAACCTGGGAGGCGGAGGTTGCAGTGGCTGAGATGGCGCCATTGCAC

TCCAGCCTGGGCGACAAGAGCAAAACTCCATCTCAAAATAAATAAATATG

GTGGCGCGTGCCTGTAATCCCAGCTACTCAGGAAGCTGAGGCAGGAGAAT

TGCTTGAACCCCGTAGGTAGAGGTTGCAGTGAACTGAGATTGCACCACTG

GAAGTGACAGAGTAAGATGTATGTGGAGGATGTGTGTAGTTATATACAAA

TACCACACAATTGTATAAAAGGCGCTTGAGCATCCTCAGATTTTGGTATT

CACAGGGATCCTGGGACCAATCCCCCGGGACATTGAGAGACAACTATATT

TCAGACCAGGTATAATTCTCGTTTGTTCTCTTCCCTTTTGGTCTCAGGCA

TAATACTTTGCCCGCTGCCCCACAGCTCTGAGCCTACACTGTGGGGCCTG

GTAACTCTCCAGAATGCCTCAACTTTGACACCTAACAAGGACTGTCCTAT

CCCTTCAAATGCTGTTGAACCCTAAAATACTTCAAAACTTCTGTTCCCTT

AGACCCACTTATAAGCCACATCATTCCACACTGAATTTTTTAAAAAACAG

CAAACACTTCCCTGCCTCTCATTCTCTCCCCTTCATAAAGAATCACACCT

TCTCCAAATGCTGCCAGATTCGCACGCTTTGATGGCACACACAGCACACA

TTATGTTCATTTAAGTGAATTATTTCCAGTTAAGCCCAATTACCATACGA

AAGGCTCACACTTGTGCTTGTCCTTAAATGTGTGTCAAATTAACTTTGTT

AGAGGTTGTTTTTAAGGCAGTGGAGAAATATAAAGAACAGTTTCTGGGAG

TTAGTTGGCACTTGTTTTTTTTTTTTTTTTTTTTTTTTTTTTTGAGACAG

GGTCTCCCTCTGTTGCCCAGGCTGGAGTGCCGTGGTGCGATCTCGGCTCA

CTGCAGCCTTGACCTCCCCAGGCTCAAGTGATCCTCCCACCTCAGCCTTC

CAAGTAGCTGGGACTTTACATGTGCACCACACCCAACTAATTTTTTAAAT

TTTTCGTAGGGGCAGTGTCTCATTATGTTGCCCAGGCTGGTCTTCATTTT

ACCCCTTCATTATCTCAGTAGTGCATGACCAGAATATAACACCTCTCAAT

CCATTTTCTTTTGTTTTTTAATAAGAAAAAAAATAATAATTTTAGGCCGG

GCGCTGTGGCTTACATCTGTAATCCCAGAACTTTGGGAGGCCAAGGCGGG

TGGATCACTTGAGGTCAGGAATTCCAGACCAGCCTGGGCAACATGGTGAA

ACCCTGTCTCTATTAAAAATATAAAAATTAGCCAGGCACGGTGGTGTGCA

TCTGTGATCCCAGCTACTCAGTGGGCTGAGGTAGGAGAATCACTTGAACC

CAGGAGGCAGAGGTTGCAGTGAACCGAGACCACACCATTGCACTCCAGCC

TGGGTGAAAGAGTGAGACTTCATCTCAAAAAAAAAAAAAAAAAATTTTAA

AGGTGGTATCTCACTCTGTCACCCAGGCTAGTCTCAAATTCCTGGCCTCA

AGCAGTCCTCCCGCCTCAGCTCAACCTGTTTTCTTATCCTAAAATAAATC

ATTCCTGTCCTGCCTACCTCCCAGAGTCTCTAGGAGGATCACACAAGATG

GGTCCTTATTCCCAAATCCTCCCTGATACACCTCATGTTCAAAGCTCTGT

CCTCCAGGTACAATAGCTAGACAGCTCATTGCCACCCTGATGGTCTTTAC

AGTCTGGTCAGGACAACAGAAGGTAAATAACTAATGCACAAATAGCCAAC

ATTGACTGAATACTCGCAGTATGCTCAGCCCTTACTGTAAAGTGCTAAGC

AGTTTACAAATATTACCTTATTTCATCCCTATACTAGTCCTCTGAGGTGG

CTGCTATTGTTCCCATGTTTACACATAGGAAACTGAGGCTTAGAGGGGTT

ATGTTGATGGGCACATGCGTGATCATGGTAGAGCCTGGCTTGAACATGTG

TAGTATGACCCAGTGTCTGTGGACATGATCATGGTGCTTCTGTCAAGGTG

CAGGGTGACAGGCACGAGAACCATAAACCGCACACTTGTCCGGGACTGTT

GTCATGGCCACATCTTCATTATCATCATCAGATTAGTTTTCTATTGCTGC

GTAACAGAGGACTACAAAGTAGCTTAGGAAACACACATTTATTATCGCAC

AGTTTCTGTGGGTCAGGAGTCTGGGCACAGTTTAGCTGGGTCCTGTGTAA

GGCTGCCATTAAGGTCTAAGCCAGGGCTGGGGTCTCATCTGGTGACTCAG

CTGGGGAAAGATCCTCTTCCAAGCTCACTCAGGTTGTTGGTGGAATTTAT

CTCGTTGCGGTTGGACGACTGGAGTCCTTGGCTTCTTGCTGGCTGTTGAT

TGGCACTGCTCTCAGCTCCTAGAGTCCGCCCGCAGGCCTTTGCCTTGTAG

CCCCCTCCCTAGGCTCTCCCACAGCATGGCAGCTTGTTTCTTTAAAGCCA

GTGAGGGAGAGTGTCTTCAGCACGAGCCTGCTAGCAGGAGAGGGAATCTT

ATATAACGCAGCGCAATCCTTGAGTGACACTCTGCCGTCCATGCCATATT

CTGTTGGCTAGAAGCCAGTTACGGGTCTTGCCCATACTAAAGGAGGGGGT

CACACAAAGGCATGAACACCATGAGGCAGTGGCCATAGGGGCCACCTTAT

CATTTGTCGCCCACAATTTCTACCTTCATTTGTGCTTGTCCTTAAATATG

TGTCAAATGAACTTTGTTAGAAATTGTATGTAAGTCAGTGGAGAAATAGA

AAAAACATTGGGAGTGACAGTGTTCCTGATGGTTTCTTGTCGTGTCAACA

ACTTACAAGAACATTTAGGATGCCAGCAGCGTGTCTGACATGTTTTTTAT

TCCCTCCTGGATTCCATGGACCTGCCCTCTGGGGAGTTAATACAGTCAGG

GTTGCTTCTATGGTTAAATCATCCCCAGGAGTTTACAGAAGAGGAACTGA

GCCACGGGATCTTGAGACAGAGCAACAAGGGAAGAGAGAGCCCAGTGTTG

AATTTAGGCATTTGGTAAAAACTTTAAAATTCATCCACTTATTTATTGAT

TCCTCAGTTCATCTGTGCATGTGTTCATATATCCAGCACGTTCCAGTGAC

TTCCTTCTGTATGGTGTGTTCAGTACTGTGCTGGTGTTGACACAAACGTG

GCTGTGTCCCTACCGTGTCCAGGGCAGAGTTAACAGATAGGAGAGGTGGG

ACACCTCTGCCTGGGACACTGTGGGTGGCTTCATTCCCTTTGGGCGTGGG

GACCATCCTGGAGTCCACTGCAGGCACAAGCAGGCAAAGGGAACAGCAGG

TAGGAGTGCAGAGGTTCTGAGGGCAGGTGTGGGGCAACCCCATGGGGCTC

AGTCATGCCTCAGGGCAGGTAGGGGGCCAGAAGCAGTGGCCACTGCACCC

TGCCTCCTCACTTCACTAGGAAAATGGGAGGGAGCGGGATGTCACCCCTT

GAGGTGGTTTGTCTTTGGGGCACAAGATACCTCTGAACAGGAGCCTCAAC

ACCAGGCAGACAGGTTCCTGGGAAACCTTGAGTGTTTTATTTTTATTTTA

TTTTATTTTATTTTGAGACAGAGTCTCATTGCGACTCCCAGGCTGGAGTG

CAATGGCATGATCTCAGCTCACTGCAACCTCCGCTTCCCAGGTTCAAGCG

ATTCTCCTGCCTCAGCCTTGCAAGTGGCTGGGATTACAGGTGCATGCCAC

CACGCCTGGCTAATTTTTGTATTTTTAGTAGAAACGAGGTTTGACCATGT

TGGCCAGGCTGGTCTTGAACTCGTGACCTCAAGTGACCCACCTGCCTTAG

TCTCCCAAAATGTTGGGATTACAGGCGTGAGCCACCGCACCCAGCAAGTG

TTTTAGACAGCTAAAGATTCATCCTGGCTGGGTGCGGTGCCTCATGCCTG

TAATCCCAGCACTTTGGGAGGCTGAGGAGGGAGGATTGCTTGAGCCCAGG

AGTTCGAGACCAGCCTGGGCAACACGGCGAAACACTGTCTCTACAAAAAA

ATCAAAAAAGATGAAGCAGGAGGATTATTTGAGCCCTGGAGGTTGAGGCT

GCAGTGAGCCATGACTGTGCCACTGCACTCCCTCCTGGGTGACAGAGCGG

GACCCTGCCTCAAAAAAAAAAAAAAAAAAAAAATTCCTCCCTGTACCGGC

ACTTTTCAATTGTTTCGTTTTTGGTTGGAAGTCTTTCCAGAATATAATTC

ATGCTTCCCTCTTCTTAAAACAGAATAAACACAATTTTACCTGGCCTCAG

GTGGCAGGGCTTTTTTAAGTGTCACGAATGTGGTTTATAGAATTTCCATT

TCTTCTCTTGTTCTCTTTCTCTCACTTGTCTTTCCTTGTTCTTGCCGGTG

AATCTGATTTCAGCAAACCTTTCCCTCCCTTCCAAGTTGCTGCTTCTAAA

TTACTGTGGGGTCAGGAACCCTCATTCCTATTTAGGTTAAGATTTTGAAA

TAAGACCATCTTGGATGATCTGGGTGAGCCCTAAGACAGGTGTCCTTAGA

AACAGAGGCAGAAGGAAATTTAAGACAGTCAGAAGAGGAGAAAACACAGA

CACACAGAGAAGGCCACTGAAAGACAGAGGCAGAGACTGGAGTGATGCAG

CCAGCCCAGGAATGCTGGGACAGCCACCAAAGGTAAAAAGAGACAAGGGA

GGCTTCTTCAGGCTGGATATGGTGGGTCGTGCCTGTAATCCCAGCACTTT

TGGGAGGCTGAGGCAGGTGGATCATCTGAGGTCACGAGTTCGAGACCAGC

CTGGCCAACATGGTGAAACCCCATCTCTACTAAAAATACAAAAATTAGCC

AGGCGTGGTGGCGTGCACCTGTAATCCCAGCTACTCGGAAGGCTGGGGCA

GAATTGCTTGAACCTGGGAGGCGGAGATTGCAGTGAGCCAAGATCATGCC

ACTGCACTCCAGCCTGGGCGACAGAGTGAGACTCTGTGAAAAAAAAATAA

AAAATGAGTTCATACAGTGGAGTTCAAGGGCAAGCTGGGCCTCACCCCAA

TGTCAGAATGAGGGGCCAGAGGTCATTTGGAAACAAAGCATTGCTTTGAT

TCATCACTTTCCTTTAGCTTAGCTTTAGTTGTGCTATTTACTTGATATTG

AATTTCAGCTGTGAAGTTGGGAGTGGAGTGAGTGTTTATCCAAGGAGAGT

ATTGATTAACAGAATATGAGATATTAACAAAACACCATGTTGAGGGTAAC

AGTATGTTCTGAGGAGGGACAGGAACTTGAGCGCAGGAGTTTGAGCTGCT

CAGAGACTTTCTTTTTTTTAAGACAGTGTCTTGCTTTGTTGCTCAGGCTG

GAGTGCAGTGGGGTGATCATGGCTTACTGTAGCCTTGACTTCCCAGACTC

AAGCCGTCCTCCCACTTTAGCCTCCCAAGTAGCTGGGACTATAGGCATGT

GCCACCATGCCTGGCTAATTTTTTTTTTTTTTTGTAGAGATGGGTTTCGC

CATGTTGCCCAGGCTGGTCTTGAACTCCTGGGCTCAAGCGATCCACTCAC

CTCAGCCTCTCAACGTGCTGTGATTACAGGCATGAGCCACCAAAGTGCTG

TGATTACAGGCATAAGCCACCGTGCCCAGCCCAGAGGCTGCTTTCCTACC

CTGTCTCTCTGTAACAACCCCATTGCAGTTGTGAATGAGCTTATTCGTGA

CTCCAGGAGTATAATAATTTCCTGTGGCTGCTGGATGGCTTAAATTATTC

TCTCACAGTTCTGGAGGCCAGAAATCTGAAATCCAGCAGGGCCATACTCC

CACTGGAGGCTCTAGGGAAGAAGCCTCCCTTGTCTCTCTTTAGCTTTGGT

GACTGTCCCAGCATTCCTGGGCTGGTGGCTGCATCACTCCAGTCTCTGCC

TCTTTCAGTGGCCTTCTCTGTGTGTCTGTGTCTTCTCCTCTTCTGACTGT

CTTAAATTTCCTTCTGCCTCTGTTTCTAAGGACACCTGTCTTCGGGCTCA

CCCAGATCATCCAAGATGGTCTCATCTCATAATCTTAATCAGCTCCGCAA

AGACCCACATGCTCTGGTTGCAGGGATTTGATGTGGCTATCTCTCCAGGG

TGTTTTTCAGTCCACTGCAATGAGTGACGCAGTAAAAGAATGAAGAACAG

TGCCTGACGCGTAGTGGGGCCGCTGTTGATATTGCTATTGCTGCTGTTTT

TGGATTACTTCTCATCCTTTCATTCATTCACTGGTTATCCAGGTATTGGC

CCTGGCTCCCCATCATGCTAGGTGCCAAGGGGGAATAAGTGAAGTGAGGC

ACCCTGACCCACGAGGGGATGATGCCCCATGTTGCTGGATATCTGAGGTC

CCACAGTACAATGGACTGTCCTGGCTGAGCCTACAAACCTGCCACACTTA

TGGTATTGTGACTTTGGGGCGTGGCATTCCTAGTTGCAAGCAGCCAACTT

GCCAAATAACCTTGTGAAAAGTCCAAATCATTGTCAACTTATGAATTAGG

TGTACTTGGCCGGGCACGGTAGCTCACGCCTGTAATCTCAGCACTTTGGG

AGGCCGAGGTGGGTGGATCACCTGAGGTCAGGAGTTCGAGACCAGCCTGA

CCAACATGGTGAAATCCTGTCTCTACTAAAAATACAAAAAATTAGCCACG

CGTGGTGGCGGGTGCCTGTAATCCCAGCTACTCGGGAGGCTGAGGCAGGA

GAATCACTTGAACCCAGGAGTGGAGATTGCTGTGAGCTGAGATCGTGCTA

CTGCACTCCAGCCTGGGTGACAGAGCAAGTCTCAGTTTTAAAAAAAAATT

AGGTGTACTCAAAGATGGTCTGAAATGGAAGTGTGACTCCCTGCAGTTAC

AGTTTTGTTTTTTTATAATTTTTTTGAGACAGAGTCTCACACTGTCACCT

GGGCTGGAGTGCAATGGCATGATATCGGCTCACTGCAACCTCCACTTCCC

AGGTTCAAGCGATTCTCCTGCCTCAACCTCCTGAGTAGCTGGGACTGCAG

GTGTCTGCCACCACACCTGGCTAATTTTTTGTATTTTTAGTGGAGATGGG

GTTTCACCATGTTAGCCAGAATGGTCTCGATCTCTTGACCTTGTGATCCA

CCTGCCTCGGCCTCCCAAAGTGCTGGGATTACAGTCATGAGCCACAGCGC

TCGGCCCCAGTTACAGTTTTTAAAGCACTTTCCCATCTGCTGCCTCACCT

GTGTTTTCTGGGCTGACGACAGACTAAACACCTGAGGCTCAGCCTGATCA

TTAAGCTCTTGAAGGATTGGAACGTGCGGTCCGCTGACAGCGCAGGCTGG

CTGGCAGCGCTGCCCGTCTTTTTTTGCCCACTCCTCCTCCTGTGCTTTCT

CAACAGACCTTTACTGGCACTCACTATGTCCTGTCTGCTGGGCTAGGTGC

TGGGAGTAGGAACAGGAAGCTGACACTCAGTCTGGTGAGAGGGAGGTACA

AGCGAGTTGGCCACCATGATGCAGGGAGACGTGTGATGCTTGGGGTGGGC

ACTGGGAGCCCATAGCTGAGTGAGGGGGGTTGTAGGGCGCTTAACCGAGG

CCTGAAGGATGAGTAAGAGTTACTTGTGAAGCTAGAAGGGTGGGGCCTCA

AGGCAGAGAGGCTGGCCCCAGAAGGCCTGGGGGGCATAGAGCCAGCATCT

GAGTGAGGAGGGCCCTGGTCTGTGAGCTCAGGGACACCTCCCCTTATGCT

GCCTGCCTCCACCCAATCTCCTTCAGTGAGCTGGTTTGTGGCTGAAATTC

AGCCATTAGAGCTTCATTCTTTTGGCTTAAGCCAGATAGAAAATGTATTC

CAGTCCTGGCACCATGGCTCACGCCTATAATCCCAGCACTTTGGGAGGCC

GAGGCGGGTGGATCACCTGAGGTCAGGAGTTTAAGACCAGCCTGGCCAAC

ATGGGGAAACCCCGTGTCTGCAAAAATACAAAAATTAGCCGGGTGTGGTG

GTGCACACCTGTAATCCCAGCTACTTGGGAGGCTGAAGCAGGAGAATCAC

TTGAACCCGGGAGGCAGAGGTTGCAGTGAGCCAAGAGCATGTCATTGCAC

TCCAGCCTGGGCAACAGAGCGAGCCTCTGTCTCAAAAAAAGAAAAGAAAA

GAAAATGTATTCCATATTCAAATGCAGAATCCACTGAAGATGAGTCTCCT

GCGGCACCAACCCATTCCACATCAGGGCATCTGGTATTTACCCAAGGGTA

GGTTTTGCCATGAACCAGGGGTCAGGCAAGGGCCAAAGACACCGAGTACT

TGTAGATGTGATTGGAAAGCAAATCATCTCTATATGGGGACAGGAAGAGA

GAGGACAGCGCTTGGTGGTCCACACATGTTCCTTGGGCCAGCCGTGGGCT

ATCCCCCCACATTCAGACATTGGGTCCTGACACTCTTTATAAAAACACTC

TGTGTTGAGTCCCTTCCCTGTGGATGGAAGGAAGTAGGTTTCCTCCAGGA

ACGCATGGCCCAGTGTGGTGAACTTGTATCTGCTTGGCTCCTGTCTCTTC

CACTGCCTGCTCCCTTGCATCCCAGATCACTGGTTCACCCCCAGGTCTGG

CAGCCTTTACAGAGTACCAGGACCCCGGATGGGGGCTACAGGGAACTGTG

CTTCTCCCATGTGACCCAGAGAGGCAGCAAAGGGACTTCTCCATCTAGCC

CACTGGAAACATTGCTTTTATTTGCCTTTGCACCATATATTCTTTTTTTA

ATTTTTTTTTATTTTTTAGAGACAGGGTTTCACCATGTTGCCCCGGCTGG

TTTCGAACTCCTGGGCTCAAGTGATCTGCCGGCCTTGGCCTCCCAAAGTT

CTGGGATTACAGGTATGAGCCACTGTGCTCAGCCCTGCACCATATATTCT

TGAACTAAAGACACATACACACACGCATGCACACACACACACACACACTT

TAGATTATTTTGAATATTATCAACCTATCCATTTTCCTTGACATTTTTCA

ATGTATAGTATGTCCTCAAAATATGATGTTATGCCCACATTGCATTTATT

TTATTTTATTTTTAGAGACAGGGTCTTGCTCTGTTGCTCAGGCTGGAGTA

CAGTGGTGTGATCATAGCCTACTGCAGCCTCAAAATCCTGGGTTCAAGCG

ATCCTCCCTCCTCAGCCTCTTGAGTAACTGGGACTACAGGTGCACACCTG

GATGATTTTAAAATATTTTTTTAGAGATGGCCTCTCCCTGTGTTGCCCAA

GCTGGTCTCAAACTCCTGGGCTCAAGAGATCCTCCCACCTCAGCTTCCCA

AAGCATTAGGATTACAGGTGTGAGCCACCGTGCCTGGTCTGAATTTCTTG

CTTTACATGTTAGCTGGGAAGTAGATGCCCTCAAGAATGGTGGAGCATAT

GAGCTGTCCCTCTAGGTCAGTCCCATGGCCACCCAGCTCCAGCCCTGTCT

CTAGGTGTGTGAATCCCTTTTGCACAACAGAGAGCCTGGGGAGGTGGAAG

GTCTTGGTGACACCTTGTGAGTTATTCACACTGGCTGTGGGTGACAGAGC

CAGAGGTGGGAGCACAGAGCTCTCACAGCACAGAGGAGTAAGGAGTTGAA

GCTGCAGAAACAATGCTGAGGCCTGAGGTGGAGGAACAGTTGTAAATGGA

AAATTAGACGGCAGTTCTGCAGGTTGGAAAGTGGGTGATAAAGTGGGCGT

GCAGGCACCATTCCAGGAGGCTGTGCCTTGTGTGCATTGAGAGCAGGTCA

GGTGGCCAGGAGGCTCCAAGCTTAAAGCTGCCAGGTGAGCCAAGCATTTT

TTTTTTTTTTTTTTTTTTGAGACAGAGTCTCCTTCTGTCACCCAGGCTGG

AGTGCAGTAGCGCGATCTTGGCTCACTGCAACTTCTGCCTCCCAGGTTCA

AGTGATTCTCCTGCCTCAGCCTCCTGAATAGCTGGGATTACAGGCGTGCG

TCACACGCCCAGCTAATTTTTGTATTTTTAGTAGAGATGGGGTTTCACCA

TGTTGGTCAGGCTGGTCAGGCTGGTCTTGAACTCCTAACCTCGTGATCTG

CCTGCCTTGTCCTCCCAAAGTGCTGGGATTACAGGGTGAGCCACTGCGCC

TGGCCTTTCTTTCTTTTTTCTTTTTCTTTTTCTTTTTTTTTTTTTTTGAG

ACAGTCTCACTCTGTCACCCAGGCTGGAGTGCAGTGGTGCCATCTCGGCT

CACTGCAACTTCCACCTCTGGGGTTCATGCGATTCTCCTGCCTCAGCCTA

CCGAGTAGCTGAGATTACAGGTGTGTGCCACCACGCCTGGCTAATTTTTG

TATTTTTGGAGACACCAGTTTCACCATGTTGGCCAGGCTGATCTTGAACT

GCTGACCTCAGGTGATTCCGCCTGCCTCGGCCTCCCAAAGTGCTGGGATG

ACAGGCATGAGCCACCACGCCCGGCCTGGGCCAAGCATTTTTGCAGTGAC

TTTCCTCCTTGTGAGCAGCCTCAGAGGCAGGCACTGGATCCAGAACCTCG

TCTTGATCCTAACAGCCTCTGTGTGCCAGCTGCAATAGCATCCAAGCTAC

AGGGAAGCAGTTTCTATTTTCGCATTGGAGTCAGCGGCTCCTGCCTGCCT

GGTGGGTCGCATCCTCCCATTCTTTGCTCAAACTTCAAAAGGGCCTTCCA

GGATCCGCCTTCCCAGGGTACATTGAAGAGCTGGTGTTTCCCCACATCCA

CCCTCTCTCCCGAGGAAAGGCCGTGGTCTGAGGGGAACGGCAGTGGCAGG

GGCCACCTACTCAGGGGGAGTGCAGGCAGGAGGTCTAGGGGTGTAAGATG

GGCAGCCTCCCTGCTGCAGGGGGCTCCCAGCTGGAGCGTGGACTTGTTTC

TCTCATTTCCTGAGGTTTGCTGGAGACAGGCGTTTGCTTAGCGCCGGGAG

CTGTCTCCTGTGGTCTCTGTCCTTTGACTTATTCAGAGTCTCACTTGGCG

TCGGTTTTCCAGGGTCTGGTAACCAGGATGAATGACGCATGGAGTTGCAA

CCCAGACTGCAGTTCAGGAGTTTCCCAAGCAGGCAAGGCTCTGTATCCCG

TGAAAGGGGGTGTGGATACGAGATATTCTGCCCTTATTCTTATTTTTTCC

TAATGTTCTTTTAGATTTATAAAATAAGTAATAATACTCATAAAAACTCA

AACGGGCCAGACGCGGTGGCTCATGCTTGTAATCCCAGCACTTTGGGAGG

CTGAGGCTGGAGAAACACTTGAGCCCAGAGGTTTGAGACCAGCTTGAGCA

ACATAGTAAGACCCTGTCTCTAAAAGAATTGTTTTAAACATTAGCCGGGT

TGCCAGGTGCAGTGGCTGACGTCTGTAATCCCAGCACTCTGGGAGGCCGA

GATGGGCAGATCACCTGAGGTTAGGAGTTCGAGACTAGCCTGGTCAACAT

AGTGAAACCCCGTCTCTACTAAAAATACAAAAATTTAGCCAGGTGTGGTG

GCGCACACCTGTAATCCCAGCTACTTGGGAGGCTGAGGCAGGAGAATTGC

TTGAATCCGGGAGGCAGAGGTTGCAGTGAGCCGAGATCACACCACGGCAC

TCCAGCCTGAAGGACAGGGTGAGACTCCGTCTCAAAAAAAAAAAAAAAAA

AGATTAGCTAGGCGTGGTATGGTGGTGCACACCTGTGGTCCCAGCTACTA

GAGGAGCTGAGGGGGTAAGATGGATGGCTGGAGCCGGGGAGGTTGAGGCT

CTGGTGAGCTATGATCATGCCACTGTGCTGCAGCTCAGCGGGTGACAAAG

CCAGACCCTGTCTCTGAAAAAAAAAAAAAAAAAAAGTCAAGCAATTCATA

TGTAGATGCAGTTAATGAGCTGGGCCTATTTCTGTCCTGCTTCTTCCCTA

AGTCCCCAAACACCTCCCATATCAGCCTTGGGCATGTGTCCTTCCGGTTC

ACCCACTTCTTGGAGATGCTTTCTGTTGACTATTTGCTGAACTCTGTGGC

CGCCCAGAAAATACTCCAGGGAGTGTGATGTGCGTCTATTTCCGGAGCCT

CATTTCAGCAGGACTGGTGCCTGGTCAGGGTCTAGACCCTCCACCTCAGG

GCCTGCCGTGGAGCAGCCAGAACCCTGGCTTCCCCCAGGTCCCCTAGACA

GGCTGCTGTCACCTCTGCCCAGCTCTGGACTCTGGGGTCTCCCCAGATGA

ATGCACGGCTCCCTCTCCCCCAGGCCCTCATCGGGGACAGGTGGCCTCTG

CATGTTCCACTGGTCTGGCAGGTCAAGAACTCAGATTGTTTTAACAGTGG

GTGAGGCTTTTATGTATTCAGGTGAGACAACGAATGTGAAAGCTCATTGT

CACCTGTAAAGCACCTACCAGTCTGCTGTGACGATGACGGGATGATGAGA

ACTGTCATCGCTCCTTCTCATTCCTGTGGCTTTACTGTGGGACCTTGAGA

GCCAGAGCAGCAGGGGGGAGGTTCTGAGGGTCTAGAAGCCTCGGCAAGGG

GCTGTTGCAGCCAGGGGCAGTCAGGCAGGGTCACCCATGCAGGGGCAGCC

AAGTGGCTTGCCCAAGCTGGGGTCTATGCCTAGAGGACCCTCCAAAGATG

CTGACCCCCTGAAGCCTGGCATCACCCAGGGAGGAGCTGTCACCACCATG

GCCCTCTGGGCTACTAAAAACAACCTTTTGCCCAGGACTTGCAAGGAAGA

GAGAGAGAGTTCTCGGTGGATGAGAACAGTTTTTCAGTGGCAGTGCTACA

GACATTTGGGGCCAGATGATTTTTTGTGGTGGGGGCTGCCCTGTGCACTG

TAGGATGTTTAGCGGCATTCCTGGCTTCTACCCACTAGAAGCCAGGAGCA

CTCCCTACCCTTCAATTGTGATAATCAAAAACGTCTGGGTTTTTTTCTTC

TTTTTTTTTTTGAGACAGAGTCTTGCTCTGTCACCCAGGCTGGAGTGCAG

TGATGCGATCTTGGCTCACTGCAACCTCCACCTCCCAGTCTCAAGTGATT

CTCCTGCTTCAGCCTCCCAAGTAGTTGGGATTACAGGTGTGTGCCACCAT

GCCCAGTTAAGTTTTGCATTTTTAGTGGAGATAGGGTCTTGTCATGTTGG

CCAGGCTGGTCTCGAACTCCTGACCTCAAGTGATCTGCCCGCCTCGGCCT

CCCAAAGTGCTGGGATTACAGGCATGAGCCACCACACCCGGCCCCAAAAA

TGTCTTTAGATATTGCCAAATGTCCCCTGGGTGACAAAATCATCCCCCAA

ACTGCTGGATTAGAGGCTTTGGAAATGGATTTTCTGTGACCACTGCTTCA

CCATTGTAAGCTGCCTCTCATTTGGGTGCAGTTTTCTTTGTTCCTTTTCC

AAAAAGCTGACCTCATCGGGCCCATTTGTTTGTTTCGTTGACTCACCCAG

TCTAACCGCAAGCAGCCTTCCTCCTCTGGGGGATCCCAAATGACTTATCA

TGGGACTTTGTATGTAAATTTCAAAGATTATTTTCTACTAAATCAGAGGC

AGAAATCCTCAACTTCTTTTTTAGCAGCCTTGGTAAATACTTCCTTAGAG

TGTTTAAGGGGCGTGCCGCACTCCTGCTGGTGGTCTGATTGTGAACACCT

TTGCTGTTCAGTTCCCTGAAATCGCCAAATATTTCCCTCTCCTTTGGGTC

TCAGCCCAGTGACTAGCGGATGATCTAAGGGCCAGGGCGGTCTGAGTTGG

CTAGTGCACCGGGGATTTTCTTAAGGCCTTTGACCCGGGGACTGGTGTGG

GAGGAAGGCAAAGGCCTGGTTGTTTATGAGCAAGGAGGAGTCAGGTCCGA

GGATGGAGCCCTTCGGGGTCACTGCCCTGGGCCCTGCCCCTCTCCTGCAC

ATCAGGCCCCTTGGACAAGGCCCGTGTGTTTGCAGGACCCTCTTGGGGCA

GCAGAGCAAGGGCTGGCTTTGAGAGAGAGCCTCACTTTTTTGTTTTTTTT

TTTTTTTTTTTTTTTAAGTAGAGACGGGGTTTCTCCATGTTGGTCAGGCC

AGTCTTGAACTCCTCACCTCAGGTGATCTGCCCTCCTCGGCCTCCCAAAG

TGCTGGGATTACAGGCGTGAGCGACCGCGCTCGGCCAGGGCTTCACTTTC

AAGCTCAGTGCTGCTCTGAGGGGTGGACACAGCCCTGATGTCAGTAGCTG

AAGACCTGCCTGGCCACACTCAGCAGCAGTTAGCCTTCTCCTCCCGCGCC

CCGTGAACAAGGCTGTACCCTGGGAGGTTGGGCTCTGTCTGAATTCCATC

ACAGGTCCTCTCCTGGGCCTTGCCCTGCTTTGTTGCCTCCTTAGGAGGAT

GTCTTCTGGTTTCCTAGGAAGGGCTTTATATCTTAACATCCACCCTGCAT

TCAAAACCCAAGTTATCAAGACCACAGGGCACCACAACCCTCCTCTTGGC

CTTGCGGTTTCTATTCTTCAGTGACTCCCAGAGGTTTGGCTTGATTGGCA

GCTTAGAAGCACATGATTGAACTTGAGCAGTGGTTGAAACTCAGAGTCAG

ATGCCTGACCCGCCCCAATCCCAGGCACAGAGTCTGACCTCACACCCAGG

CTGTGCTGTGTACTCAGTTGTTCAGTATCGACTTGAGCTTGACGGGCATC

AGGGGCCAGGGAGTGAAAGGGAAGAGAAGCCAAATCTGGCTGCTAAGGGC

TCTCAGTCCTGGAGGGCAGGAAAATAGTGGCCTTGCCGCTAGACCTGATG

CCACCTGCCCCTCCAGCTGTACCCCCTCAACACCTTGGGTAATACCTCAA

GCCCCACGTCCTCTTCCAAGCCAGATAGGACCCCAAGTGCTGGGTGATCC

TTATTGAAGGGTAGGGACAGGAGGGACCCGTGGGATTGTGTGTGCTCAGA

GCGGAGTTCAGGGGCACCTGAGGGCAAACTGTCTCTTTGCTCCCAGGGCT

GTCCTCCGTGCCTTTACCTGTGCTGGGCCCTGGCAGAGCTCTGAGTCTTC

TGGATTCCAGCGTCCATAGTAGCTAGTCTGTCCACTGCTGGAGAAAAGCT

GCTTTCCCAAAATAGCCACCCAGGGGGAAAGAGCAGCCCTGGAATTTGAC

CCTGACGATCACCGTGACGGGGCTGCTGGCCCCACCTGGCCTGACCTCCA

CGACATCACGGAAGGACTGTTGGTTAGTTGTGGGGGCCAGGATTTTTCCT

CTGTCCCCCTCCCCTCTTCTTCTTCCCTTTCTTCTTTCTCTCTGCCAGCT

TCAGGTTTGAAATTCCAGTGACACCGAGGGAGATGCTCCAGTGCCTGTCC

GGCTGGGGGGCAGGTCACATGGTGGGAGCAGAGAGGGGAGGGCACTTGTG

CGCCTGCACCTCTTGGCAGGTGAATGCACACCTATGGAGGGGCCTGGGTG

CTGGCAGCGAGCCCCGCTCCCTGCGGCTGTGCCAGGCCCAGAGGCGGCAG

CGAGCCCAACAGGTCAGACAGGTGGAGCCAGGCTGGGGAAGCTGCAGGAC

CAGAGCTGACATCCGATCAATCCAAGGTCCTTCTTTGGCCCACAGAGCAG

GTAACCCACCCTTTTCTTTCTTTTTTTTTTTTCTTTCTTTCTTTTTTTTT

CTGGTTTAATTCACTGACAACTTTAGGGAACATTTACAACCCGAGAAAAA

GGCCATTGAGATTGGGGGGCAATCGCGAATTGTTTGGAGAGGGATTTGTG

GTGTTTTGTGTGGAGGAAATTTGGCCTCGGCATTAGAAAAGCATTTCTAA

TTTTCAGGTCTTTGATTCTGAAAGCAGATTTCTTATGGAGCCTGGGTGCT

GGGTGGCATATTGGGGAGGCAAAGCCCAGAAGCCATGAGGGTTTTCCTGT

GACTGCTGATGGAACACCGTGGGAGGCTGTAGTTTGGTGGCCTGTTTGCC

TCCATAAGGAGGGGTTGGGGCAGAGACAGGGTGGTCGGGGGACTGCTCCG

CGACAGGACTTGGCTGTATGGGGGAAGCTCTCCCAGTTCCTATCAGAGAA

ACACGTGGAGGGCTGGACTCCCTCCAAAATTCAAATTTATGCTACAGGCC

CTTACTGGGGCTGGATTTTACTTGGAGATTGTTTGGAGTTTTGAAATCAC

CTCTCCTGGTAAGAAGACTCGGTCTCCTGTGTTCTTGCCCCCTCTGTATC

TTGGCCAGTGATGGTTCCCTTAGACAAATTGATGGGGGATTTCTGGCCTG

AGGACAGGAAGAAAAGGAGCCCAGGTCTGTTTCAGTGAAGAACAAGCCCA

GGACACCCCACCTGAAACTAGGACTAGTTTTGCCCAGTTTGGCATCGTTG

AAAACGCATTTGCCCTCTTCCTTGAACCAGGCTGTGCCTTCTTCTATCTG

TTTCCCTGCCATGGAACATCACCCAAACATTTTACTGGGAAGGGGAGGGG

TAAGAGCAGATACAATGCTTGTCTGAAACCCCCGTTGCATGGCATTCTTT

GCAGGGACAGGACAGGGGAGCAGTTTTTGCTGTTACCGAACCCTTTGTGA

CAGAGATGCTGTTTGTTAAGTGATTTGCTTTTTTACAAAAAAAATTCCAA

GGTAATTGATGATGACACAGTTGCTTAGACTATGGAATATACCAGTTAGG

ATTCCTTCAGCTGCAAGTAAGAGAAAACCTTACTTAAAATGACTTAATCA

GAACAGTAAACATTTTTTAAGGCTAGGTGCAGTGGATCACGCCCATAATC

CTAACACTTTGGGAGGCTGAAGCAGGAGGATCCCTTGAGCCCAGGAGTTC

GAGACCAGCCTGGGCAAACAGGCAGATGTGGGGTATCTCTACAAACAAAC

CAAACCAAACCAAAACAAAAAAACTAGCCAGGTGTGGTGGCATGCACCTG

TGGTCCCAGCTACTCAGGAGGCTGAGGTGGGAGAATCGCTTGACCCCAGG

AGGTGGAGGCTGCAGTGAGCTGGGATTGTGCCACTGCACTCCAACCTAGG

CAAAAGAGCAATGCACTGTCTCAAAAATAAAAAAAGTAATAAACCAAGTA

GTCCAGAGGTAGGGCAGCTTCAGGACAGGTGACATCCAGGCGCTCAGTAA

TGTCAAGGACTTTGGTTCCCTCCGTATCTCTGCTCTGCTGCCCTCACCCC

ACTGGCTTCATTGCCTGCCAGTTCCTCTCCCATTGGGCTCCTCCCGGGGT

CTCCTCATGAGTGAGGAAGCCTTTCCCAAAGCCCATGCTGACTTTCTCAC

ACACCTCATTGGCCAGAATTGGGTCTTGTGCTCAGCCCCAAACCAATCAG

TGGCAAAGGAAGGGATGACCTCGAGGACTTCATGAGGCACGTGGCCAGGG

GAGGAGGGTGGGTTGCCCAGACCAAATCAGGGTCTATGAGCAGGGAGAAG

AGGGGGCGTGGATTTGGGACAGGCAACCAACAGCATGTGCTACATCCAAA

AATTTAAATTTACTCAGATTTTAAACTGATTTGGGGTAAAACAATAACCC

TTACTCCTCAAATATATATATATGTGTGTGTATATATATGTGTGTGTGTA

TATATATATGTGTGTGTGTATGTGTGTGTGTGTGTATATATATATATATT

TTTTTTTTTTTTTAAGAAATTCTCACCAGGCACAGTGGCTCATGCCTGTA

ATCCCAGCACTTTGGGAGGCCAAGGTGGACAGATCACCTGAGGTCAGGAG

TTCAAGACCAGCCTGGCCAACATGGGTGAAACCCCGTCTCTACTAAAAAT

ACAAAATTAGCCGAGTGTGGTGGCAGCCGCCTGTAATCCCAGCTACTTGG

GGGGCTGACGTAGGAGAATTGCTTGAATCCAGGAAGCAGAGGTTGCAGTG

AGCCGAGACCGTGCCATTGCACTCCAGCCTGGGCAACAAGAGTGAAACTC

CATCTCAAAACAAAAAAGAGATTCTCAATGCAGTGGTAATTTCCGACAAT

CCTAGCATCTACTCTTCACTTTTCACTGCCCAGGGAGACCCCTCTTCCCA

GTAAGCAGCTGGGCTGCTAGTTTTTAGGTGTTTGAGCCTTTTTTCTTGTT

TTTTTTTTTTTTTTAAATGAACCTGCTGCCTCCCAGAGTTGGCTGCTTAA

GGTTCCATCCATAAGAACCAGAAAAAGGGAAAAACAGAGTCTTAAATATT

AAGAGGTATGAGATGAATGAGTCTCTCGCTCTGCAAATGGCAAAGGCAAA

AGGCTTTTGGCTGGTGGCCCCAAAGAGCCAGCAAGGCTCTCTGCCATTTA

CCCAGGGATGCTACGGGAATCAGCAGAGGGACAGCATTCTAACACATCAT

CAATAGGGCAACTAGCAACCTGCTGCAGTTTTCTCCTGTCTCCTGGGGAC

ACGAGGGTGGTGGTACCCTGCCCCCCACCCCCCACAGCCGGTGAAAGCTT

GTTCCAGTAGCCACGGACCTGTCAGTCATTTTTGAGGGTCAAAACTTCCA

CATCCATCTTTTTGACCCCATCCCCAAGACAGCCACTCTCCTCTCTGCCA

TGGCTCCAACCTGGACAGAATTAGCTGCAGATCTGGATGGAGTCTCTGAA

GCCCCTCTTAGCCACCAGCTTCTTCCAGGCCAGGCCAGAATTTCTTTTTT

GTTGTTATTGTTTTTCTTTGAGACAGAGTCTTGCTCTTGTCACCCAGGCT

GGAGAGCAGTGGCATGATCTCAGCTCACTGCAACCTCTGCCTCCCGGGTT

CAAGCTATTCTCCTGCCTCAGCCTCCTGAGTAACTGGGATTACAGGCATG

CACCACCACTCCCAGCAATTTTTTTGTATTTTTAGTAGAGACGGGGTTTC

ACCATGTTGGCCAGGCTGGTCTCAAACTCCTGACCTCAGGTGATCTGCCC

ACCTCGGCCCCCCAAAGGGCGGGGATTACAGGCGTGAGCCACTGCACCTG

GCCCAGGCCAGGGTTTCAAGAGGAGATGACACAGTGGCAGAAAAATTCCT

TCAAGGGGACTCTGAGCTGGTGTTAAACATCCGCAGCTTCCTCTCAAAGC

CTGGGACGCTGGCTCCTCCCCAGCACTGTCCTCCCTCCACCAGGACGGGC

CCTTCACTAGTACTTGTGGTTTAGAGGGGATTCGAGGAAACAGTTGAACT

GCCCTGCAGAGGGAGAGAGGAGGGCTTTGGTCTCTGGAGATGTTTTTATT

TTTTATTTATTTTATTTTATTTATTTATTTATTTGAGATGGAGTTTCGGC

TCTCGTTGCCCAGGCTGGAGTACAATTGTGTGATCTTGACTCACTGCAAC

CTCTGCCTCCTGGGTTCAAATGATTCTCCCGCCTCAGCCTCCTGAATAGC

TGGGATTACAGGCATGTGCCACTACACCCAGCTAATTTTTTTTTTATTTC

TTAGTAGAGACAGGGTTTCACCATGTTGGCCAGGCTGGTCTTGAACTCCT

GACCTCAGGTGATCTGCCCACCTCGGCCTCCCAAAGTGCAGGGATTACAG

GTGTGAACCACCGTGCCCAGCCTATTTATTTTTTTAGAGACAAGGTCTCG

CTCTATCACCCAGGCTGGAGTGCAGTGGTGTGATCACGGCTCACTGCAGC

CTCCGCCTCCCAGGCTCAGGTGATCCTCCTGCCTCACAGGCGTGCACCAC

CATGCCCAGCTCACTTTTTAATTTTTTGTAGAGATAGCACCTTGCAATGT

TGCCCAGACAGGTCTTGAACTCCTGTCCTCAAGCAGTCCTCCCTCCTCAG

GCCCTCAAAGTGTTGGGGATTATATGCGTGAGCCACCAGACCAGGCCAGG

ATATACTTCTAAATGAAAAGCTACCGGCCCAGCCTTAGCCGATTTCGGTC

ATGAAAGTGGCTTTTCTTTCTGAATCCATCACCTGTGGCCCCTACTTTAG

CTGTGTTGGCCTTTAACCTGTTAGAGGACATTAAAGCAATCTCTTTGCTC

TCATATAGTCATATTGTGTTTGGATCAGAGGGACGTCTGAGGTTTCATCT

GCTTATGCAGACGGTGCCAGCTAAGCCGTGCCTTTTGGGTCCAGGTGACC

TCCAAAGCAGGCCTCTCCAACACATAAAGATCATTGGGACATAGTCACGT

GTGAAGGGTCTGCCCGTGTCCGCTGTGCATTAGGTGCTGTTGGGGAAATG

GGAGCAGAGGATGAGGTCATGACCCCACACACCCGCAACCTGATGTTTGC

TGTCATGGTGGAAGCATGCCCGTGGTGGGGGGTAAGGGTATGGGGCTTCG

TTTTGTTTGGTTTGGTTTTCAGTATATTTACCAGTCCCCGCCACCAGGCT

TCTAGAACAGTTCTGGCAAGAGATCCCTCCACCTCTAAACATGCTGTGTC

TTCCCATTGCTCCTAAAACAAAGGCGGGTTCTTCGTGGAGGCCTGCGAGG

TCGCCCACTTCTGCCTCACTTGTCCCCGCCTTCTTGGTTGCTTCAACACC

GTGGCCTCCTTGGCAACTTCAGCCACCACACGACCCTCCCAGGCTGCTGG

TCCTCCCTCCCCGAGCCCTACTTCACCCACCCTGATGCCCGTGGTGCGGC

GTGTTCCCTGTTACCCTTCCCTGATCTCATTGATGAGGCCACGGGGACGA

CATCCCAGACTACCTTCTCCTCTGGAAGAGAAGCTAGGGGTCTTTCCTCC

CTAGCACTGCTCCCCATTGTAATTTTGCCCAGCTGTGATTTCTTGCCTGT

GTCTGCTCCACGGACATTAGATTCTCCAAGGCCCAGGACACCTGTCTGTC

CCCCCGCCATGACTGGTGAACACCCAGCATGGTGTCCACAGGTCAGAGGC

TCTCGGTGGCTGTGTGTTGGATGTGGAGGGGACCATCATGCTTCTGGGAG

ATGTGCTGTGGGGCTGAGGAGGTTTCCTGAGTGAATTTTCACATCCCAGT

GATGGTGAGAGCTCCTGTGTTTCTGGGAATCATACCATCCGAGAGCATCA

TTACCAGAATCAGCATTTTCTTTTCTTCTTCTTCTTCTTTTTTTTTTTTT

TTTTTTGAGACAGAGTCTTCCTCTGTTACCCAGTCTGGAGTGCAGTGACA

CCATCTCGGCTCACTGTAACCTCCGCCTTATGGGTTCAAACAATCCTCCC

GCCTCAGCCTCCCAAGTAGTTGGGATTACAGGCACCTGCCACCATGCCCG

GCTAGTTTTTGTGTTTTTAGTAGAGACAGGAGTTTCACCATGTTGCCCAG

GTTGGTCTCAAACTCCTGACCTGAAGTGGTCCACCCACCTCAGCTTCCCA

AAGCAGTGGGATATACCGGTGTGAGCCACCGCACCCGGCCAGAATCAACG

TTTTCTTTTGTGCTGAAGGAGCTCACAGCCCCTCTGGAGTTCTATTTCCT

GCTAAAGATGGCTTCTGATTTTCTCGAATTCCTCCTGGTTTGCTGGACCA

GCAGGGCTGGCTGGGCAGCAAGACACATCAGGCCTGGGGCCTTATACATC

TTATGTTTAATGCAGCAGAAGCTGGGCCTGATACTTAGAGTGAAGGCCTT

TCTGTAGAAAGGAACATCATTTAAATCTCTGTCCTCAGGTTGTGACTCCA

GGGAAGTCTGACATGGTTAAAGATTGCTGACTTGGTACGTTTTGTATTTT

GTGGAACACAATCAATTTTCATGTATTGATTAATTATTCCCCATTGCCTA

AAATCCTGGCCTCCTCTCTCTTGATGGAGAAGGCTGTCCCCAAAGTCCAG

GCCTAGAATCTTTGACTGGAAAGACTGTCCTGAGAGTCCAGGCCTCCTCT

CTCTGACTGGAAAGACTGTCCTGAGAGTCCAGGCCTCCTCTCTCTGACTG

GAAAGCCTGTCCTCAGAGTCCAGACCTCCTCTCTCTGACTGGAAAGACTG

TCCTGAGAGTCCAGGCCTCCTCTCTCTGACTGGAAAGCCTGTCCTCAGAG

TCCAGACCTCCTCTCTCTGACTGGAAAGATTGTCCTGAGAGTCCAGGCCT

CCTCTCTCTGACTGGAAAGACTGTCCTCAGAGTCCAGGCCTCCTCTCTCT

GACTGGAAAGACTGTCCTCAGAGTCCAGGCCTCTTCCCTCTGACTGGAAA

GATTGCCCTCAGAGTCCAGGCCTCTTCTCTCTGACTGGAAAGATTGTCCT

CAGAGTCCAGGCCTCCTCTCTCTGACTGGAAAGACTGTCCTCAGAGTCCA

GGCCTCTTCTCTCTGACTGGAAAGACTGCCCTCAGAGTCCAGGCCTCTTC

TCTCTGACTGAAAAGATTGTCCTCAGAGTCCAGGACTCCTCCCTCTGACT

GGAAAGATTGCCCTCAGAGTCCAGGCCTCTTCTCTCTGACTGAAAAGACT

GTCCTCAGAGTCCAGGCCTCTTCTTTCTGACTGGAAAGACTGTCCTGAGA

GTCCAGGCCTCCTCTCTCTGACTGGAAAGCCTGTCCTCAGAGTCCAGACC

TCCTCTCTCTGACTGGAAAGATTGTCCTGAGAGTCCAGGCCTCCTCTCTC

TGACTGGAAAGACTGTCCTCAGGGTCCAGGCCTCCTCTCTGTGACTGGAA

AGACTGTCCTCAGAGTCCAGGCCTCCTCTCTCTGACTAGAGACTGTCCTC

AGAGTTCAGGCCTCTTCTCTCTGATTGGAAAGATTGCCCTCAGAGTCCAG

GCCTCTTCTCTCTGACTGGAAAGATTGTCCTCAGCGTCCAGGCCTCTTCT

CTCTGACTGGAAAGATTGTCCTCAGAGTCCAGGCCTCCTCTCTCTGACTG

GAAAGGCTGTCCCCAAGTGATTCTCCCCCCTTGGGCTCCCAAAGTGTTGG

GATTACGCATGTGAGCCACCATCCAATTTTTTATTTTTATTTTTAGAGAC

AGGGTCTTGCTCTGTCACCCAGGCTAGAGCGCAGTGGCTCACTGCAGCCT

CAATTCCTGGGCTCAAGTGATCCTCCCACCCTGGCCTCTTGAGTAGCTGG

GACTACAGGCATGCACTACCACCACCACCTCCTCTGTCTAACCAGAAAAA

ATTGTCCCTGAAGTCCAGGCCTTCTCTCTCTGACTGGAAAGATTGTCCAC

AGAGTTCAGACCTCCTCTCTCTAACTAGAAAGATTGTCCCTAAAGTCAGA

GCCTCCTCTTTCTGACTAGGAGGATTTTCTTCTCCATCTGAGGGTCCTGA

CTCAGGAGGAAGAGGAAGGGCCCCCTCCTGTCCTTCTGGTGCCTGGGGCA

GCAGCCTTCATGGGTAGATGCCTTCACCCAGGTGGCTGTCTAGATTCAGA

AAATGGAATTCTGTTTGGAAGTCTGGGACAGGGGCTGCTGAGCAGTGGAA

AGTGCGTGTGGTTTGGCTCAGATGTCCCCGGGATCTTACTCTCTCTCTCT

TCTGAACCATCTTTTCCTCTGGGACCGTTTCCGAGCCTGAGGATCGGGCT

GGCTGGAAGACTTGGCAGTTGCACTTCACTGTGTTAGTGTTTGCCTGACA

CCCCTGCCATGCCCACCCCTGAAAACACACGGCCCTCCCTCTGGTGGCAG

TGCTGGGGCTGAAGCCAGGAGCCACCATGAGGCCAGATTCCTGCCTCTTG

CACCCCATCTCTGGATGGGCCAGCCCATCCCACACGCTTCTGCTGTGCTT

ACAAAGTTCAAGTGTGCTGTGGTCACTGTTTCCATGTGGCTATCTGTTTT

CAGGATTCTGGGGTCCCCTGCCCCGTCCAGTCTTGGCTGGGAGTTGAGGG

AGGGAGGAAGCTTTGAGTTTTTGCTTGTCTCTGCGCTGACCTGGGGACAC

AGCAACTGCAGGATGTACCTTTGGCATCATCCCAGAGCTGCCAAGTCTGA

ACTTCTAGGTTTGAACCTGGGAATCTGCATTTTACCAAAAGCTCCCCAGC

CGATGCTTACACAGTGAAGCCGAGACCTGCCGCTCTAGCTGATGAGCATG

CCCTGCTGGAATTTACCTTTGGGTTAAAATCTGACATATTAGGAGCTTTT

TTTTTTTTTTTAAGACAGAGTCTCGCTCTGTCACCCAGGCTGGAGTGCAC

TGGTGTGATCTGCAACCTCTGCCTCCCAGGTTCAAGCAACTCTCCTGCCT

CAGCCTCCCAGGTACCTGGAACTACAGGCACATGCCACCATGCCTAGCTA

ATTTTTGTATTTTTTTTTTTTTTTTGGTAGTAGAGATGGCGTTTCACCAT

GTTGACCAGGCTGATCTCGAACTCCTGATCTCAAGTGATCCACCCACCTC

AGCCTCCCAAAGTACTGGGATTACAGGTGTGAGCCACCACGCCCGGCCTA

AGAGCATTTTTCCCACACTAAAAGATAACACAGTCCAGCTGGCTTGCTTT

TTAACCAGAGCATTACAGGGTCACCTGTTTGGAGGAGGAGGACTGAGGTC

AGAGCAGGCTCCTGTCAACTCCAACGGGACATTTCACAGTGGCCACAACA

GGCTGACCTCTGTGTCCTGTGTCCTGCTGACTGTGTGTGGAGGCCTCAGA

GGGGCTCTTTCCCCCTTGCACAGAGAGAGACAGCACTGGCTCATGAGCCT

GCTTTTTTCTGCACTACCTTACCTGGAACTCAAGTTTCTGGGACTTCCCA

TGCTGGGGTAGAGGTGACTTTGATCCTCCTGAAAACAGTCCTTCATGCCC

CCACACCCACATGGAAGAGGAACTGTTCCGTGGTGGTCCGTATCCAAAGT

CACTAGATCTTGGCCTGGCATAGTGGTTCATGCCTATAACCCCAGTATTT

TGGGAGGCCGAGGCGGAAGGATCACTTGAGCCCAGGAGTTTGAGACCATC

CTGGGCAACATGGCAAGAACCCATCTCTATAAAAACAACAACAACAAAAC

AACAAGAAGTCAGTAGATCTTAAAGCACTAGATCTTAAACCCTGTGATGG

GCAATTTCCTTGTCAATTTAGCGAGGCTGTGATACCCATGATTCAACCAA

ACGCTTGCCTGGGTGCTACTGTGAGGTGCGTTGTACATGTGGTCAGCACT

GATGATCACTGGCTGTAAGTCAAAGGTTCTCCTCTGCGAGGTGGGTGGGC

CTCATGCAATCGGTTGAAAGGCCTCAAGAGCAGAACCGAGGTTTCCCTGA

AGAAGAAGAAATCCTGCCTGGGGACTGCAGTGTCAGCTCCTCCGGGGAGC

TTCCGGCCTGCTGGCCTGCCCTCTGGATTTCAGACTTGCCTAGCCAGCCC

CCACAATCACGTAAGCCGATTCCTTGAAATAAACTTAATCTATATCTCCT

TCTGGTTCTGATTCTCTGGCAGAACTCTGATCCTCTCCCTAAACGGTGCC

CGGGACGGGGGAGGAGTTGGCGCTACTTCATTGCTTCCCTTTCACGCAGA

CATGCTGACTTTTCAACCACTGTCCTTTTCGAATTTTTTCTCTTCCAGGG

AAGTGCTGACTCTTTTTCTCCTTGTCAATTAGTCCTTTTGGAATCTGTTA

CTGGTCTCAGCCTTCTCCTCCATCCTCTCTCTTTCATTATTTTTATTTTC

ATTTTGTAGAGACAGTTGCTGAAGGTGGTGGTCTGGCTATGTTAACCAGG

CTGGTTTTGAACTTCTCGCCTCAAGTGATCCTCCTGTCTTGGCCTCCAAA

ATTGCTGGGATTACAGGTGTGAGCCATCACCCAATTTTTATTTTTAGAGA

CGGGGTCTCACCCTGTCACCCAGACTGGCGCAATCACAGCTCCCTGCAGC

CTCAACCCCTGGGCTCAAGCGATCCTCCTGCCCTGGCCTCTCCAGTAGCT

AGGACTACAGGCACGCATGTCACCATGCCTGGCTAATTTTTAAAATTTTT

TTGTAGAGATGGAGTCTTGCTAGTTGCCTGGGCTGGTCTTGAACTCCTGG

CCTGAAGCGATCCTCCCACCTCGGCCTCCTGCATCCTTTCTTTCCTCCCC

ACATTTCCTTCTTATCAACAGGTGCTGGGGAGAGGCAGGACAGGCCTGTC

CCCCGAGTCCCCTCCGGATGCCGTGGACCGGCCAGCTGTGAGTGTTTCTT

TGGCAGTGTCTTAGCTGGTTGTTGTGAGCAATAGTAAGGAAGCAATCAGC

AAGTATACTGCCCTAGAAGTGCTGCACGTTGTGGGGCCCAAGAGGGAAGA

TGAAGCGAGAGATGCCCAGACCAGTGGGAGACGCCAGGACTTCGGAAGCT

CTTCTGCGCCACGGTGGGTGGTGAGGGCGGCTGGGAAAGTGAGCTCCAGG

GCCCCAGGAGCAGCCTGCTCGTGGGTGCGGAAGGAAAAAGGCACAGGGGC

TTGGTGTGGGCGGCTTTTGGCTGGGAGAAGTTTGCACGTAGGGAGAATAG

TAGCCAGTGTTTGCAGAGCACTTACTATGCAGGAAGGCCTGTCCTAAGTA

TTGTAAGTGTATTACATCATGTACAAGTGTCTGTGATTAACCCCGTCTTG

CAGAGAAGGAAACAAAAGTACAAACAGAAAATGTAACTAAGCATGCAATT

AATAAAAAGGGACCAGGTTTTGAACGCGAGCAATCTGGCTCAAGAATCTG

CGCCCAACCACCGGCTCCTGTTCTTAGAGATGAACGTGGAGTCCTGGAGA

CTGCTCAACATTGTGACTTGACTGTGAGCGTACGCGCTCCCTGTCCCCAG

GAGACAGATTTCCAGTGCAATCATAGAAAGTGCCTGTGTGGGCTTCGGGA

GATGTGTCTGCCTTGGGGAGAATTTTCCTTTTCAGCTAGAGCCAGGCCCA

GGATGTTGACGTCAGTGAGACGCTGGTGACGTTCTCTGCTCCAGTGGCTG

ATGAGAAAAGTTCCTCCAAGCCAGCTCAGTTGAGAAGAATTAAGTTCTCT

GGGTCCCACTGGCTTCACCTACAGATGCCAACTTTGAGGCCAGTGAACTG

TGAGGCCAGCTGGGCTGATTGCCATGGCAACAGGAATTGGACCAAAGTCA

CCGGAGGATGGAGAGGGAAGACACAGTGGTGGCTTCCCCAGGTCTTGGAC

CACAAGGCACAGCCGTGGCCTCCAGGAACCCTGAGATAACCCGTTAGTGG

GTCCTGCACTCCAACAGAGCTCATGCAATCAGCCTCTGGTCCTCACCCTC

CTCCCATTGGTGTCGTTGTGCTCTCTAACATTGACATTGAGCAGTGAGTG

CTCCAGATCTTGTTCCACTGATTTTTTCCACTGTTCTCCAGTCTAGCACT

TTCTGAAATTCATCCAAGCCTAAGAGGTGACGCCAAACGTAAGTGTCTTA

CACCTCGGACCCCATGGACTAAGACAAGCCTCTGGGGTCCATGTGGCACC

AAGGATGGGGCCTTCTAGTACCTTTCACACAGGCCAGGGACAGCACCTGC

TGCGACCCCCACCCCCAGCCTCTGCTGCTTCTCCCGTTGCCCTTCCAGCC

ACTGCCCTGGGCACCTCCCCTGCTCCCGCCTCCCTGCCTGGGCCCATCTT

TCTTTGCACCTCACCAATTGCGGGGCTTCCTAGTGCTGGAGGGGAAGGGA

GCCTGCTTCCATTCTCACCACTCTGAAAGAAGACCCTGCAGAGAGGTTTT

CTGGAATTTGGAGCCTAACCTTGGCCTTGACACAGTCCAGATGAGAAGGC

CGTCCCCCAGTTAAGCCAGCCCTCCCTCCCTCTCAATGCTGGAGAGGAAA

CCAGGCAGCTGGTTGCTTCAGCAAACCCCCTCCCTATGGGAATTGTCCTT

TTCTTCCTCAAATAGACTGGAGTGCAGAGGACTTGCTTCAGAGAGAGCCC

CTTTGTAACGCACATTGCACGGGCCGTGCCCCATGCCCACTCTGCCACAA

ACTCCCCTCAGCTGCCCAGCCCCGTGGAGCCAGGCGTGGGCTGAAGTGGG

GCCAATGAAATGCCTGGAATGCCAGCCGCAGTGACAGGCCAGCACTCAGA

TGCCTCTCGGGAGTTCTGACCGAAGCAGCACTGAGGACTGCTTCAGAGCA

GAAGTCTACGCCATTTTCCCCAGTTCTAGGCAGAAGTAAAGAATGTCTCT

ACCTTCTCCCAACCAGCAATTGATTCAGAGTCCTGAGCTTTTAAATTAAA

GGGACCCCTCTGTTGCTTTATGCAGTAGCCAGCTTATTCATTTGAGTTCA

GGTGAAATCATTTTCAAGTAACCTCTTCCTATCCAGCTTTACTTTTAGTT

ATGCTGACATAAAAACAATAAAAATTGTCCTCCTCAGAGTTCACCTGTAA

AAGTTCCTGGATAATTAGTAAGGAAACAGAAAGAGCAGATAGTTGTTGCT

TCAGATAAAAGTGATGTCTGGGTTGGGCACGGTGGCGCATGCCTGTAATC

CCAGCACTTGGGGAGGCCCAGGTGGATGGATCCCTTGAGCTCAGGAGTTT

CAGACCAGCCTGGGCAACATCTCTACAAAAAATACAAAAATTAGCCAGGC

ATAGTGGCAGGACTGTAGTCCCAGCTACTCAGGAGGCTGAGGTCAGGGGA

TGGCTTGAGCCCAGGAGGCACAGATTGAAGTGAGCTGAGATTGCGCCACT

GTATTCCAGCCTAGGTGACAGAGCCAGACCCTGTCTCAAATTAAAAAAAA

CTCTTTGTGGTCATTTAATTTACTTCTGCATTCTTTTTTTTTTTTTTTTG

AAATGGGCAGTGGTACCATCTCAGCTCACTGCAACCTCTGCCTTGGGGGT

TCAAGTGATTCTCCTGCCTCAGCCTCCCGAGTAGCTGGGATTACAGGCAC

CCGCCACCACGCCCAGCTAATTTTTGTATTTTTAGTAGAGACGGGGCTTT

GCCATGTTGACCAGGCTGGACTCAAACTCCTGACCTCAGGTGATCCGCCC

ACCTCGGCCTCCCAAAGCACTGGGATTACAGGAATGAGCCACTGCACTCG

GCCTACTTCTGCATTCCTTAGAGTACATCCTAGCTAGGATATATTCACTT

GTCTTTAGAGCTAACAGCAGAAGAAAGGTACCTAGATGCTTATCTAGCTC

TTTATCTTTCTAGAAATTCTAGAAGATGAGAGGGGCAGTGGTGGCTCCAA

GGGAGTGATTTTAGGATGGACTGGGACAGAGAGGGGCTGCTTACACATAC

TCACACAGAGGCCATTAATTTAGGGAGTTCAAGGTCCAGGAGGTTGACGG

CATCTAGGCCTGTGGCAGAGTGCACTCCTGCTTTGGGGAATGTATGGGCA

GCTTCTTGCCTGTAGTTGGAGGGTTAGAGAGAGGGATCAATAATTTCATG

GTTGGACTTGAGTTTGGAGCGAGTGGAGTTAGATACAAATTCTATGTTTT

TGGAGAAAGTATTTGATCTTCTTGGCTGTGAGGCAAAGCGAGGTTTAAAA

ATATTTTTCACTTAAAAATATATATATATGCATGTATATTTAATACTGTC

TATGGATTGTGCCAATATCTATTTCATGGTTTTGATATTGTGTAGTTATA

CAAGGAGTTACCACTGAACGAAACTGGGGGAAGGGTACACAGAATCTCTG

TACTTTTTTTGTTGTTGTTGCAATTTCCTGTAAATCTATGATTATTTTAA

AATAAAAAAGTTAAAGCATACGTATTTACATTTGATTAAATGCAGTGTGA

TATCCTGGGTTGGATTTTGGAACAAAACAAGGCATGAGTGAAAAAACTGG

TAAAATCCAAAAAAGCCTTTAGTTAAAAGTAATACCAGTGTTAATTTCTT

AGTTTTGACAAATGTGCCATGGCTGTGTAAGATGCAAATGTTAGAGGAAA

CTGGGCGAAGAATTTGAGGGAACTCTCTGTACTATTCTTTGCAGCTTTTC

TGTAAATCTACAATTATTCCAAAATAAAAGTCTATTAAAAATGTAAGTAT

ACACACACACATTTATATTTGAACACATCTAGGCAGCCAATGCCCTTAAA

GAAGGCACTTTCTTCAAACGATGCCTC

>hg19_ct_ARAlincRNAs_9727_ARAlincRNA_0005.1 range=chr1:9211727-9243809 5'pad=0 3'pad=0 strand=- repeatMasking=none

GAGGAATGAGTCCCCCGTTCTCACCCCGGGGAGCGGTTGCCTCGTGAGTC

CAAGGAGAATCCGCCCTTTCGTTTTGCGCAGGTGGAGATCTCGCGGGCCC

AGTTCGGAGTCCTGCAAACTGTGGAGGAGATGCCGCTGTCCCGTCGGTCT

GGGGACAGCCCAGCTCCCCGGATCCCGGGCTGGAGAGACGCGTCGCGGCC

CCGGGGCCTGGTGGCACGAGCAGGAAGGAGGACCCGGCGGCGGGCTCTGC

CTGGGCTTGCCTGGGCTTGTTCCGAGCCGGGCTGCTTCTCGGTGACCACG

CAGATCGGGGGCATTTGGAGATTTTGCGGGAGTCCTGCAGCCAAGCTCCG

GGGCAGGAGAGGCCTGGAAGCCTGCACTACCTGCTCGCCCCGTCCCAGCA

TGCACCCAGGGCCAGCTGTGAGTGTTTCTTTGGCAGTGTCTTAGCTGGTT

GTTGTGAGCAATAGTAAGGAAGCAATCAGCAAGTATACTGCCCTAGAAGT

GCTGCACGTTGTGGGGCCC

>hg19_ct_ARAlincRNAs_9727_ARAlincRNA_0005.2 range=chr1:9262638-9262678 5'pad=0 3'pad=0 strand=- repeatMasking=none

AGCATCCAGGCAGGACGGCAGCAGCTGAGCAGAGGAGAGAG

>hg19_ct_ARAlincRNAs_9727_ARAlincRNA_0006.2 range=chr1:9474622-9487071 5'pad=0 3'pad=0 strand=- repeatMasking=none

CTGGGAGGCAGAGGTTGCAGTGAGCCGAGACCTCGCCGTTGCACTCCAGC

CTGGTTGACAGAGCAAGACTCTGTCTCAAAAAACAAAAAAACCCCAGCTT

TTGTCCAGAGGTTGCTTTTTGTGGGTCTGTACGCTGAGGCCAAGTTCTAT

GTAAGCAGAGCCCTCCCACCTCACCTCCAAACAATGCCTCTAAACCATCA

CAATCTGAAAGGAAAATAAATCCCTTTCTTCAATGGTGACCTGAAGCTAG

CCAAGGACCGGGTGCTGGCAGGTGGCCTGCGAGACTGGGTCGGATCCCGG

GTTACTGTCTGAGGAGGGATGCCGTGCTGTAAGGGTTGGGGAGCCAGTGT

CTGGCTTCCTTGTCACCACAAGACCTGTGGTTTCCAGCTCTGTCTGCATC

CAGGTCCCACTAACCAACAGCAGGAGAGAAAACAGTTGTGCACACAGGCG

GACGCGCACACGCGCACCTGGCCCCCCAGACGTGCACAGCCCTCAGAGGA

AGTGGAGCACCAGATGTTCTGGGTTTCCTGTTCGACGCTTCCACAGCTGG

TCGAGTCTGTGGTTGTGGTTTGTCTGTCTCGGGCTCATGTGAGAGACAAA

AGCTGGGCTGACGGGAAACCTCTTCTCTCGGCCCCCACTCCCCAGCTCTG

ACTGATTAGGAAACCTGGCAGGTCAGAAATTTCCTGAAACACCCAGAGCT

GGATTCTCCCAAGAAGAAGCTGAACCCCCAACTCCCATCCCTGAGGAGCA

GCCCCTGGGCTGATGATCAGGACTTACAGAACCTGGGGCGATGGAAACCT

CTACAAATATCCCATTTAGAAAGACCGCCAGCCCTGCCAACAGAATGAAA

CGGAGGCCAGGTTGCTATGGTCTCTACTCCAATGCCTGACATTGAGCCGC

TGCTGCAAAACACTCAGCACATGAAGGAATGAATGAGGAACAGAGAACTT

CACCTTTGGGGGTTGGGTGTACAAGCCCCAACTGGGCCCCAAGTCCCATA

AATAGCTTTGGATTCCAACATACAAGCATTTGACGCAGCGAGGTGCTGCC

GACTTGCTCTGTGACTTGGAGCAAGTCATTTAGCCTCTCTGGGCCTGGTG

TCCTCATCTGCAGAATGGAGATGATAATATCTACTTCACAAAGGTGTGAA

GGCCCTGTGAGACACCGCATGTGGATTTCCTCGACATTGTTTGCCCATCG

TAACTGCTCCAGAGAGGCTGGGCTCCCTTTTCTCGTTGCAACACTGCTGT

TTATTTCAGGAGATACTCTAAAAGGGAAGCAAGCTAAAACAAAGAACAAC

CAAAAACACTGATCCCGGGACAACTGCCCGGCAGGACAGCAGAGCAAGGG

AGAAACAGCAAAAGAAAACACAG

>hg19_ct_ARAlincRNAs_9727_ARAlincRNA_0006.1 range=chr1:9485034-9487440 5'pad=0 3'pad=0 strand=- repeatMasking=none

AGAACACCACCACTAATGGGAAGACTGCCCCCTGACCGGCACATGGCCTC

AGCATTCATCCACAGATGCTCCTCAAATTGTTTTAAAAACAGCTTTTTTT

AAAAGCTGTTTGTCTATGAAGATTAAATGAGTTAATAATATAAGCAAAGC

ACTTTGCATGGCTACTGGGCACGGTGGTTCATGCCTGTAATTCCAGCACT

TTGGGAGGCCGAAGCAGGTGGATCACCTGAGGTCAGGAGTTCAAGACCAG

CCTGATCAACATGGCGAAACCCTGTCTCTATTAAAAATACAAAAAAAATA

GCCGGGCATGGTGGCACGTGCCTGTAATCCCAGCTACTTGGGAGGCTAAG

GCAGGAGAATCGCTTGAACCTGGGAGGCAGAGGTTGCAGTGAGCCGAGAC

CTCGCCGTTGCACTCCAGCCTGGTTGACAGAGCAAGACTCTGTCTCAAAA

AACAAAAAAACCCCAGCTTTTGTCCAGAGGTTGCTTTTTGTGGGTCTGTA

CGCTGAGGCCAAGTTCTATGTAAGCAGAGCCCTCCCACCTCACCTCCAAA

CAATGCCTCTAAACCATCACAATCTGAAAGGAAAATAAATCCCTTTCTTC

AATGGTGACCTGAAGCTAGCCAAGGACCGGGTGCTGGCAGGTGGCCTGCG

AGACTGGGTCGGATCCCGGGTTACTGTCTGAGGAGGGATGCCGTGCTGTA

AGGGTTGGGGAGCCAGTGTCTGGCTTCCTTGTCACCACAAGACCTGTGGT

TTCCAGCTCTGTCTGCATCCAGGTCCCACTAACCAACAGCAGGAGAGAAA

ACAGTTGTGCACACAGGCGGACGCGCACACGCGCACCTGGCCCCCCAGAC

GTGCACAGCCCTCAGAGGAAGTGGAGCACCAGATGTTCTGGGTTTCCTGT

TCGACGCTTCCACAGCTGGTCGAGTCTGTGGTTGTGGTTTGTCTGTCTCG

GGCTCATGTGAGAGACAAAAGCTGGGCTGACGGGAAACCTCTTCTCTCGG

CCCCCACTCCCCAGCTCTGACTGATTAGGAAACCTGGCAGGTCAGAAATT

TCCTGAAACACCCAGAGCTGGATTCTCCCAAGAAGAAGCTGAACCCCCAA

CTCCCATCCCTGAGGAGCAGCCCCTGGGCTGATGATCAGGACTTACAGAA

CCTGGGGCGATGGAAACCTCTACAAATATCCCATTTAGAAAGACCGCCAG

CCCTGCCAACAGAATGAAACGGAGGCCAGGTTGCTATGGTCTCTACTCCA

ATGCCTGACATTGAGCCGCTGCTGCAAAACACTCAGCACATGAAGGAATG

AATGAGGAACAGAGAACTTCACCTTTGGGGGTTGGGTGTACAAGCCCCAA

CTGGGCCCCAAGTCCCATAAATAGCTTTGGATTCCAACATACAAGCATTT

GACGCAGCGAGGTGCTGCCGACTTGCTCTGTGACTTGGAGCAAGTCATTT

AGCCTCTCTGGGCCTGGTGTCCTCATCTGCAGAATGGAGATGATAATATC

TACTTCACAAAGGTGTGAAGGCCCTGTGAGACACCGCATGTGGATTTCCT

CGACATTGTTTGCCCATCGTAACTGCTCCAGAGAGGCTGGGCTCCCTTTT

CTCGTTGCAACACTGCTGTTTATTTCAGGAGATACTCTAAAAGGGAAGCA

AGCTAAAACAAAGAACAACCAAAAACACTGATCCCGGGACAACTGCCCGG

CAGGACAGCAGAGCAAGGGTACTTGGTGCTCTTCATTGGGGGGATGTTGG

GAGAAGGGGCTGGAGCCATCCCAGGGTCTCCAGGCTCTGGAAGGGAGCAC

CCCATGTCCTCCTCTGGGTGTGGAATAGGGTGAGTGTCTTAGGGGTGGAG

AGAGAACAGGCAGAGTGTGCTCTCACCCCTTAGCTCTGTAGTGATTTTAA

TGATTAAGGCTACTGTTCGGTCTGCATTTGGACCTGAGCAACAGGGACCC

CTGCCCAGAGGTCCATCCTTCAGCAGCGTGCACTGCATCCCCCTCCACTG

CAGCTGACTCTATGAGATGAAGCATCTGTCGCTCAGTGTAGCCATCAGGT

CCACTGCCCCTGAAATATGACTGACTAGGTTCAAATGTTGACTCTGACAC

TTAGAAGCTGTGCCACCTTGGGGCAAGTTACTTCATCTCTCTGTGCCTTA

CTTTCTTCCTTTATAAAATGGGTAAAATAATAACCACACCATCGGTTGTC

TATGAAGATTAAATGAGTTGATAATATATGCAAAGCACTTAGCATGGCTA

CTGGCACAAGGGTCAGTAAACGTTAACTGTTCCACACATGAATGTGTTTG

ATTCTTATAATGCCCCATGGGTAGATTCTGTTATTAGCTCTGTTTTACAC

ATGAGAAAACTTAGGCACAGGAGGTAAAGTAACATTTGCAAGGTCACACC

GTTAAGT

>hg19_ct_ARAlincRNAs_9727_ARAlincRNA_0006.3 range=chr1:9486352-9489003 5'pad=0 3'pad=0 strand=- repeatMasking=none

AAGAAGGCTCCGCAGGTCGGCAGAGAACACCACCACTAATGGGAAGACTG

CCCCCTGACCGGCACATGGCCTCAGCATTCATCCACAGATGCTCCTCAAA

TTGTTTTAAAAACAGCTTTTTTTAAAAGCTGTTTGTCTATGAAGATTAAA

TGAGTTAATAATATAAGCAAAGCACTTTGCATGGCTACTGGGCACGGTGG

TTCATGCCTGTAATTCCAGCACTTTGGGAGGCCGAAGCAGGTGGATCACC

TGAGGTCAGGAGTTCAAGACCAGCCTGATCAACATGGCGAAACCCTGTCT

CTATTAAAAATACAAAAAAAATAGCCGGGCATGGTGGCACGTGCCTGTAA

TCCCAGCTACTTGGGAGGCTAAGGCAGGAGAATCGCTTGAACCTGGGAGG

CAGAGGTTGCAGTGAGCCGAGACCTCGCCGTTGCACTCCAGCCTGGTTGA

CAGAGCAAGACTCTGTCTCAAAAAACAAAAAAACCCCAGCTTTTGTCCAG

AGGTTGCTTTTTGTGGGTCTGTACGCTGAGGCCAAGTTCTATGTAAGCAG

AGCCCTCCCACCTCACCTCCAAACAATGCCTCTAAACCATCACAATCTGA

AAGGAAAATAAATCCCTTTCTTCAATGGTGACCTGAAGCTAGCCAAGGAC

CGGGTGCTGGCAGGTGGCCTGCGAGACTGGGTCGGATCCCGGGTTACTGT

CTGAGGAGGGATGCCGTGCTGTAAGGGTTGGGGAGCCAGTGTCTGGCTTC

CTTGTCACCACAAGACCTGTGGTTTCCAGCTCTGTCTGCATCCAGGTCCC

ACTAACCAACAGCAGGAGAGAAAACAGTTGTGCACACAGGCGGACGCGCA

CACGCGCACCTGGCCCCCCAGACGTGCACAGCCCTCAGAGGAAGTGGAGC

ACCAGATGTTCTGGGTTTCCTGTTCGACGCTTCCACAGCTGGTCGAGTCT

GTGGTTGTGGTTTGTCTGTCTCGGGCTCATGTGAGAGACAAAAGCTGGGC

TGACGGGAAACCTCTTCTCTCGGCCCCCACTCCCCAGCTCTGACTGATTA

GGAAACCTGGCAGGTCAGAAATTTCCTGAAACACCCAGAGCTGGATTCTC

CCAAGAAGAAGC

>hg19_ct_ARAlincRNAs_9727_ARAlincRNA_0006.4 range=chr1:9488981-9489003 5'pad=0 3'pad=0 strand=- repeatMasking=none

AAGAAGGCTCCGCAGGTCGGCAG

>hg19_ct_ARAlincRNAs_9727_ARAlincRNA_0007.1 range=chr1:11111452-11112137 5'pad=0 3'pad=0 strand=- repeatMasking=none

GTCTATAACCCCAGCTACTCGGAAGGCTGAGGCAGGGGAATCACTTAAAC

CCAGGAAGCGGAAGTTGTGAAACTGCCTTTGCCAAATTATGACTGAGACA

GTGAAAGAGACCTAACTTAGCCAACTCCATCTTGCTTCTAGCCTCCAAGC

TGTCCTTCTTCATTCCTGGGCGTAGGCTGAACTAACGTTGGGAGAAATTT

AGTTTATAGTTTAAACAAAGACGGTCACAGCCCTTTCTCAAAGCAGATGT

TCTTGCCTGGGGACTAGACTGTCTTTGTAGGATTAACATTAGCCACAAGA

TTAGAAATTATGGTTTAGAGTCATGCAGCTGGAGGCTACGAGATTCCCAC

CCTCCCTAAACTGCTCCTAAGATCAGTGCTTGAGATATTTTGCAGATCCT

GCCTTGATGGATCAGCCGGCACCACCCAGATGGATAAACGGGCTCATCTG

ATCTTGTGACCCCCACCCAGGACTCAGTGCAAGAAAACAGCTCTGACTCC

GACTGCCTGTGATTTCATCTCTGACCAGTCAGCACTCCTGGCTCACTGGC

TTCCCCCTACCCACCAGGTTATCCTTAAAAACTCTACTCCCCGAATGTTC

GGGGAGACTGATTTGAGTAATAGTAAAATTCTGGTCTCCCGCACAGCCAG

CTCTGCGTGAATTACTCTTTCTCTATTGCAATTCCC

>hg19_ct_ARAlincRNAs_9727_ARAlincRNA_0008.1 range=chr1:14025768-14026552 5'pad=0 3'pad=0 strand=- repeatMasking=none

GGTCACCGCGGATCCCATGGCGGCGCCTCGGGAAGGAACCCCGGCGGCTG

GCGTCGCGGCAAGTCCGACCGCACCCTAGGGCTAGGATCCTGCGTGGTTC

AAAGATTCTCAAAGAATCAAAAAGACTGAAGGAGGTGTTGGGGGGGCATG

TCTATCGTTGGAGGTCATTAAATATCTGACAAGTACATAAACGTAGACCT

TATACTTCTACGTTTGGATCTATACCAAAAAGATCCAACTCCAGGCAGAC

AGACGCAGCTGCTGGGTTTATTTGCTGCCCTAGTACCCAGGAGCAGTGAC

CAACGCTGCCCTGTGCCCGCGATGTTCCAGTGCAGCGCCTCTGAGAGCGC

GGTAGTAGTCCTGCCCTTAAGGAGCTTGCTGTTCCCAAGCGGGCAAACAA

ATGGACTGCAGAGACGACGACTCATAGATGTTCTGATGAAGATGAACTGG

GTGGAACTGGAGATGGCTCTTCGACATCCAAGGGTGGAGGCCTAGCACGT

GGTTGAAAATGAGTTGAGTCGGGGAGAAGCACAGGCTGGAGATGAGCTGG

AAAGTTGGGAGTTGTCGTTATAGATGGTGCCTGGAGCCTTCAGCACTTCA

AGATAAAGTAATCCTCTGAGAATGAAGTCAGGAAGAATCCGAATGTGATG

GCTACGTTGGCCATCTGCAGAAAGCACCCAGTGGAAAAACAGGATACTAA

GGGCAGGTGTGCTTCCAAAAATTATGAACGATCATTTATGTAACAGGTTT

AATCTTAAGAAAACGTGTTGGCCGGGCGCAGTGGC

>hg19_ct_ARAlincRNAs_9727_ARAlincRNA_0009.1 range=chr1:16940084-16942437 5'pad=0 3'pad=0 strand=+ repeatMasking=none

GATGGGCGTGTCAGGATAACCCAAGGCGCAGGCGCGGCGGGGCCTTAAAG

GGACCCGGCGGCCTCTTCTGCACAACGGGTTCGAGCAGGTTAGGGGCCGC

GCAGGCGGAGGAAAGGAGTAACCCAGGGGAAGGACCGAGTGCAGCGGGGA

CGGGGAAATCCCTCTCTCCCCTCCGCCTGTCTTTCAAAGCACCAGCCCTC

GACCCTCCAAATCGCTGGTTTCCCCGGCCACTTGAACAGCCCCTGCCAGG

TTGAAGAGGCAGGAGACACACCCCCTCTGGGGCTGGAGCGACCCCGCGCT

TAGGACTGCAGGCCTCGCGCTGCCGCACCGCCCCGAATCTGACTTCCAGG

CACGGGCACGCGGTGCAGTCGGGCAGGCTTCAGAAGAGCCCCCAGCTCTC

CCAGAGGTGGCCTTAGGTCACTCTCAAAAAGAATAACCAATGTGTCAATG

GCACTTGTAGTTATTTTCTAATTTAAATTTATTAACAGATTTTGCAGATG

GGCTTCCACTGAAATAAGCCTTTGAGAAAAAGAAAAACTTTTTTTTTCAA

CAGGATTAGAATACCAAGAAATAGGAAGTAAAGCCATGCCGTCCACCCAG

CTAAAAACTTTGAAAACTTGATATTTTATCTAAGGCAAATGTTTGCATAA

CTTTAGGTCATGCCATTATTTAAAGTCAATTTCAGTTAACAATTTCATTG

CAACTGAATCTATCTTGTATAAGAAAACTAAGATACATCCCTGATAATCT

ACCTCTCCCCTCCTATACTCCATCCGTCAGCAAATCCTACTGGTTTTTAC

CTTCCAAATTTTCCTTGAATCTGTCCTCTTCTATCTCCTCCGTCACCACC

CTAGTCTAGGCTGCCTTCACCTGGGGAGTGAGGGTCGAGGGACTACTGAC

CTAGTCTTCTTGTGGTTTACCCATATCCCCTTTATCGCCTCTCTAATCTC

AACACAACAGGCAGAGTGGCTTTTCCAAAATATAATGTGACCGTGTCACT

TCCCAGCCTAAAGCACTTAAACACCTTCCCATTCTCTTAGACAAAACCTC

CTAACTAACCAAGCCCCGAATGGCCAGGCGCCTTGCCCACTTCTTCATCT

CTTCTCACTACTCAGACCAATTCTGTTCTGCTCTCTCTGCTCCAGCCAAA

CTGGCCTCTTTTATTCCCTATTTACCAGCTTCCTGGCCTCCACAGAGCTT

TTGCTCATGCTTTGCTCTGTGCCTGGAAAGTTCTTCTAACCCCTATTCTT

CTGAGAACAGCTGTAGTCTCCACCTCTGAAAAGCACCTTCTGACCTCCCT

ATGGTGGTCCCTTACTCCTATCACATTGCATGAAACTGTAATCGGGTGGT

TATTTTGGTGAATTTTTTACTATCGGCTACGTAAGCCCAGGGTTTGGTTT

TGTTTGCTTCATGATTTGTATTTCCACATTTGTTAAATACATATTTGTTC

ATAAATGAGAATGATGGATGCTAGAAGTTGAAGTAAATTTGGCATAAAGT

CAGAGAAAGAGAAAATGTCACAATCTGACAGTTGGAGTCAGAATGTAGAA

ACTTCAGTTATATTAAAACTGATTTTATATCGTTTTAGGCTGAGTTGTCT

TAGGATGCTTTGGGTAACCTTTAAAACTGAGTTCTCAAAGAGCCTTGTAT

AAACACCTGAATTAATAATGATAAGCCATGGGGTGGCCCACATAAAACTA

ATAACATTGGTTGCTTTAAGAGAGGAAGACTGAGCCAGGCACAGTGGCTC

ATACCTGTAATCCAAGCACTTTGGGAGGCCAAGGCCCGCAGATCATTTGA

GCTCAGGAGTTTGAGACTAACCTGGGCAACTTGGTGAGACCCTGTCTCTA

CATAAAATACAAGCAAAATTAGCCAGGCGTGGTGGCGGGCACCTGTACTC

CCAGCTATTTGGGAGGCTGAGGTGGGAGGATTGCTTGAGCTCAGGAGGTT

GAGGCTGCAGTGAGCCATGATCATGCCACTGCATTCCAGCCTGGATGACA

GAGCAAGATCTTGTCTCAAAGAGAGAGAGAGAGAGAGAGAGAAATGAAGA

CTGGTGACTGGTGGCTACTGGTGGGAAGGAGATTTTTACTAAATGCCCAA

AAGGTTTAAAATTGTAACTTTTTTATTTCAAAGTCTGTGAAGATAAATGA

CACAAAAGAGCACATACCTTCCCATTTATTTGAAATTCTAGAAAGGGGAA

AATTAATCTGTGGTGAAAAAATCATAGCAGTGGTTCCCTCTGGCAGGGGA

TTGACTGGGAAGTTACATGAGAGAAATTTCTGGGTGGTAAAAATGGTCTC

TGTCTTGATAGGGATATGAGTTATATAAGTGTATTCATTTGTCAAAAAAC

ATAC

>hg19_ct_ARAlincRNAs_9727_ARAlincRNA_0010.1 range=chr1:27020930-27022008 5'pad=0 3'pad=0 strand=- repeatMasking=none

ATCTCTGGAGCCTGCGCCGCCTCCCTCCCGGCGTCCCTATCCAGGTCTGT

CCCCTCCCGGCCGTACCGAGGGGAGCGGGCAGCGTGAACTTTACCCGCGG

CTGCAAAATTGGCCAGAGCCTGAGACCGCGGCCGCCTCGGCCCAGCCCTG

ACCCCCTGGCAGCGGGCCCGGTCCGTTGGCTGCTGCTTAGCACCTCTACA

GCACTTTTCGTCTTCAAAGCCTTCTACGGACCCCGCTAAAGACCCAACCT

TTTTTACCCTCTTTGCAAGCCCGAAAGAATGACTGATCATTGTTCAGACG

ATTCGTTTCCCACTCGCCTGCCCTCATTAGCCGGTCCTTACAAGGCTCTG

GGGGCATGAGGTCCGGATTATCCCCATTTTACAGAATTGAAAGAGAGGGG

AGGGAACTGCCTGTCACTTAAAGTCAGAGGGCAGAGGAGGGTGACTTTAA

AAAGAAGTGGAGTCACCAAAGCTGCCTTCGGTCTACTTGCCTTTAAAAAA

AAAAAAATCAAGTTGTGAGCACATTCAACAATGGGATCAGAATATCAAAA

TGGATTTGAAGTCAAAACTGATCATCTCACCACTAACTTAAAATGGAGGC

CACTGAAGTTGCTTTAGGAGCATAGAACAGTTACCCAGCAGGAAAACCTA

AACACCGTTTCTCACTCCCTCTTAAGAAAACTTAGCCTACCATGACAAGT

AGCCCCAGTCTCCAAAGACAGTACCTTCTAATACTGAAAACCCACAGAAG

AGGCTCCAGGGACTGCAGGAACCTGAGTGTTCAAACCATTACAGAAGAGA

GATGCAGAAAATGGAAACCATTTTCTCCTTGAAGAAAAGGCACAGTCCAA

CAAGTCACTTAGAAAATGGATGCTTGTCAGATTTGTTTTCTATCCTGTAG

CGTAAAGATTGTTTAAAGTTTACCATTACATTCTAAGAGACCCCGATTGA

GCTGGAGTTGGGTAGAAGGCCAGGTTTATCTGAAAAGATCGACATACACA

GAAAGCTCCAGTGTTCTCCTGATCTACAGGCCCCACCAAATCCCAGCAGC

AGCCTTTTCTTCCTCAGGCTGACTTGGAA

>hg19_ct_ARAlincRNAs_9727_ARAlincRNA_0011.1 range=chr1:31849735-31850277 5'pad=0 3'pad=0 strand=+ repeatMasking=none

CTATGGTGAGGTCACTGTTCTATGTTTCCACGGCAACCACAGGCTGGGAA

GATGGAGAAGAGGAGAGATGAGTAACCAGAAGCCAGCCAATGAGATGAGC

TGCAGCACCGGATGATCCATGGGGCCATGGGGAGCGGGGACAAGGGAGGC

CTGCTCAGCATGTTCAGCTGTTTCCTGGACCCTCCCTGCCATCTGGATGC

CTCCACTAGAGAATGTGCTACTAGCACCAGGAAAGAGCTAGAGGCTGAAG

AAGCTGAGTAGAAGCTCTGCTGACCCAAGAACATGGCCCTCCTTTTTTGC

CTCTTCAGGGTCTAAGTCTATGGTTGCTGACTACCCCCGCCCCATCCCAC

CCCTAATATCTGCAACTGGGAACTTTTCCCATTTGCTGGGAATGATGGTG

GGGCTCTTTCTGCCTCTGCCTGCCATAGAACCAGCAAGTCTTAGACATCC

CTTCTTTTTATGCCCTTGGTCCATGGGGTCTTTGTTACCAGAAAGGGGTC

CCAATCCAGACCCCAAGAGAGAGTTCTTGCATCTCACACAAGA

>hg19_ct_ARAlincRNAs_9727_ARAlincRNA_0012.1 range=chr1:32707688-32709102 5'pad=0 3'pad=0 strand=+ repeatMasking=none

TTTCTACTAAAAATACAAAAAATTGGCCAGGCGTGGTGGCTCACACCTGT

AATCCCAGCACTTTGGGAGGCCGAGGCGGGCCGATCACGAGGTCAGGAGA

TCGAGACCATCGTGGCCAACATAATGAAACCCCGTCTCCACTAAAAATAC

AAAACTAACTAGGCATGGTGGCGTGCACCTGTAATCCCAGCTACTCGGGA

GGCTGAGGCAGGAGAATCGCTTGAACCCAGGAGACCAAGGTTGCAGTGAG

ATGAGATAGCGCCATTGCACTCCAGCCTGGGCAACAAGAGCAAACTCCGT

CAAAAAAGAGAAAAGAAAGAAGAAAGGAAGGAAGGAGGGAGGGAGGGAAG

GAAGGAAGGAAGGAAAGAAAGAAGGAAGGAAGGAAGGAAGGAAAGGAAGA

AAGATAACTATCTCTTCCACCATACCCTGGTGGAAGGGCTGGAAAGTGGG

TGTGAAACTGGGAACTGCCTACTTCCTCCCATACATAAGAAACTTGGGTT

GGGGAAATCCTTTGGTCCACCTTAAATGCTGCAGCTGTGTAGCTGGCGTC

CCTCCTCGACCTCGCAGCTGGAAGTCCCTGCCTGAGGGCGCCCTCTGTGC

AGCCCACTTCTGGCTGAGTCCAGACCTCAAGAACCCCATCAGCTATCCAC

CATCGTTTCAGCAAGTGTGTATTACCTGTACAGTGAGTTAGCTGGGCCCT

GTGTTTGGTGCTGGGGCCCAGGGATGACTGGGGAAGATTCTGTTCACAGG

GTTTTCAGTCTGGCAAGAGACACAATTAAGCTGGTAAAATTGCAGGACGA

GAGCTGAGCTGATTTACCACAGGACCCATCTATTCATCCATCCGTCTTCT

TTTTCTTTTGTAAATATTTTTGTGGAGATGGGGGTCTCCCTGTTTTGCCC

AGGCTGGTCTTCAACTCCAAGCCTCAAGCAATGCTCCTGCTGCATGGCCT

CCCAAAGTGCTGGGATTACAGGCATGAGCCACCATGCCCAGCTTATTTTA

TTTATTTATTTATTTATTTATTTTCTTTCTTTCTTTTTAATTGAGAATAA

AGGAATAGGGCTGGGTGCGGTGGCTTACCCCTGTAATCCCAACCCTTTGG

GAGGCTGAGGTGGGTGGATACCTTGAGGCCAGAAGTTCAAGAACAGCCTG

GCCAACATGGCTAAATCTGGTCTCTACTAAAAATACAAAAATTAGCCAGG

CATGGTGGTATGTGCTTGTAGTCCCAGCTACTCGGGAAGCTGAGGCAGGA

GAGTCCCTTGAACCCGAGAAACGGAGGCTGCAGTGAGCTGAGATCGCACC

ACTGCACTCTAGCCTGGGCAACAGAGCAAGACTCTGCCTCAAAAAAAAAA

AAAAAAAAAAAAGCCAAGCGAGTTGGCTCACTCCTGTAATCCCAGCACTT

TGGGAGTCCAAGGCA

>hg19_ct_ARAlincRNAs_9727_ARAlincRNA_0013.1 range=chr1:36688149-36688636 5'pad=0 3'pad=0 strand=- repeatMasking=none

CCTGCCTAAGCCTCCTGAGTAGCTGATTTTACGGGCATATGCCACCACCC

GGCTAATTTTTCTACTTTTTGTGGAGACGGGGTTTCGCCACGTTGGCCAA

GCTGGTATCAGACTCCTGACCTCAAGTGATCCACCTGCCTCGGCCTTCCA

AAGTGCTGGGATTACAGGCGTGAGCCACGGGGCTGAACCAATGTAATTTT

AAATTCAAAGATTTTCTGGGTGTCTACTGTATACAATGCCCAGTGTAAAA

TACTGGGAACACGGTAGTAAACTAGATGGATACCAAAATACATAATTTTG

GCTCCCCTTTTAAGAAATACAGGCATAGGCCGGGCGCGGTGGCTCACGCC

GGTAATCCCAACGCTTTGGGAGAACCGAGGCGGGCAGCTCACCTGAGGTC

AGGAGTTCGTGATCAGCCCAGCCAACATGGCGAAACCCGTTCTCTACTAA

AAATACTACAGATTAGCCGGGCGTGGTGGTGGGCACCT

>hg19_ct_ARAlincRNAs_9727_ARAlincRNA_0014.1 range=chr1:36688989-36689658 5'pad=0 3'pad=0 strand=- repeatMasking=none

AGTCTCCCTGTTTTTGGTCTCTGCTGCGCTCCCTTCCGCTTTGTGACCTT

GAGCAAATTCATCCCTCCTCTCTAAACTTGTTTCCTCCCCTGTGAAATGA

GACTTGTAATTTCTACCCCACAGCGATGTTGCCAGGCTCCGACAAAGTAA

GGCACAGGAAAGCCTCAAGCACCCTGAAAGCGCAACGCAAATGGAGGGGT

TGGTTTGGTTTCGTTTTTTTAATTTTAATTGTGTGACCTCATTCGGAGAC

CCAGAAACCGCCTCTCCCCAGCCTGCCTTCTGTAACTATGATTTTCTGAC

TGTCGTCCTGGGAAGTTTGTTAAAATGGGTGCTACTGGGGTATTGAACCA

AAAGTTTACCGGGGAGTGTCTCCCTGCAGTCTACATTTTAAACCGATCTC

TCGGGTGATTCTTACCCACCTACAAATTTGAGAATCTCTGCGTTTATCAG

TTTTGCACAGGCTGGTTTCCTCCGGAATTTCTGCTCTAGTGTCTCTGAAC

ATTTAAGACCCATCTCATAGGTCATCTGTGATGCTTTCAAAGTTGACTAG

CTCCTCCCTATGCCTGTGTTCCCACAGTAAGTTTGCATCTTACTCTGTAA

GTATCGGGGCCTTCCCCGACAAGACTGTGAGCTCTTTGAGAGCCGAGATC

AGTTTTGATGGATTTCTGCA

>hg19_ct_ARAlincRNAs_9727_ARAlincRNA_0015.1 range=chr1:43583495-43583879 5'pad=0 3'pad=0 strand=- repeatMasking=none

CACCCGATACAGGAGCACCCAGATTCATAAAGCAAGTCCTGAGTGACATA

CAAAGAGACTTAGACTCCCACACATTAATAATGGGAGACTTTAACACCCC

ACTGTCAACATTAGACAGATCAACGAGACAGAAAGTCAACAAGGATACCC

AGGAATTGAACTCAGCTCTGCACCAAGTGAACCTAATAGACATCTACAGA

ACTCTCCACCCCAAATCAACAGAATATACATTCTTCTCAGCACCACACCA

CACCTATTCCAAAATTGACCACGTACTTGGAAGTAAAGCTCTCCTCAGCA

AACGTAAAAGAACAGAGATTATAACAAACTATCTCTCAGACCACAGTGCA

ATCAAACTAGAACTCAGGATTAAGAATCTCACTCA

>hg19_ct_ARAlincRNAs_9727_ARAlincRNA_0016.1 range=chr1:43821443-43823292 5'pad=0 3'pad=0 strand=- repeatMasking=none

GAAAAAAAAAAAAAAAGTTTACATACATTATCGAGTTCAATCCTTAATCC

CATAAGGCAGATAGATACCCCATCTTCCCTTTACAGAAAAAAGAAACTGG

GTCAGAAAGATTAGAAACTTGCCCAAAGGCATGCAGCCAATAATAAGTAG

GGGAGCCCAGGATTCAAACTATCTGACTAATCCCTGCAATCTAAACAATA

CGCTATACTGTCCCAGAAAGAGGCTGGAAAAATCATTTTCAGTCATTAGC

TTAAAGTCCACTCCGATGCTTACAGATAATGTTATTATCTTTGACTTGAA

GCCTTTGCATTCTCTATTTCCAATTACCTTGGAAAATAAGGAATTGGATA

TGCTTAGGTTAGAGGTGGGGAATTCCCAGAAAACATAGAAAAGGTTGTCT

CATGTCAAGTTGGTCAGGGTCTTAAATGTTAAGCTAAAGACTTTGGACTT

CATCTGCTAGGCATTGTGGTACCTGTGGGGTTTCTGTGTAGGGAAGGACC

AAATAAAACATCTGAGGGGGGGGATATGGCAATAATTTGCAACTTCTGGT

GGTAGCTATAGAGAAGGATAAAGGGTATAGCCTCAGGGAATTTGTACATT

TAAAAATAAAGGATTTGGGCAGGTTGTCATGGTTCACTCCTGTAATCCCA

GCACTTTGGGAGGCCAAGGCGGGCAGATCACTTGAAGTCAGTAGTTCAAG

ACTAGTCGGGCCAATGTGGCGAAACCCTGTCTCTACTAGAAGTACAAAAT

TAGCCAGGTGTGGTGGTGCACACCTGTAATCCCAGCTACCTGGGAGGCTG

AGGCAGGGGAATCGCTTGAACCTGGGAGGCGCAGGTTGTAGTGAGCGAGG

TCGCGCCACTGCACTCTAGCCTGGGTGACAGAGGGAGACTTTGTCTCAAA

ATAATAATAATAATAAATGAATGAAGGAAGGCAATGGAAATTATCCAGAC

ATGGATCTTGAAGTGGGAGATACCCTAATATATGTAGTCAAGTGGAATGA

TGACAGAAAGGGTCAGATTTGGCCATTGAAAATCAAATTACCGATGACTT

TGGAGAGAGCAGGATCAGGAAAGTGGGTGCTTGAGATCCATTTTGGAGGA

GTTGCGGAAAGAATGAAATAGGGAGAACGGGAAGCCGGCAAGTATAGGTG

ACTTGTGTAGCCCATCCCCTGATATCCTCTATATTCGTTTGCTAGAGTTA

CTGTAACAAAGTACCACAAACTGGCTTAAATGACAGAAATTGGTTGTCTC

ACAATTCTGGAGGTTAGAAGTTCCAGATCAAGGTGTCATGAGAGTTGTGT

GAGAACCGTGAGGAAGAATCTGTTCCATGCCTCTCTCCTGGCTTCTGGTG

GTGGTTTGCCGGCAATCTTTGTCATTCCTTGGCTTATAGAAGAATCGCCC

TGTTCTCTGCCTTCATCTTCACTTGGCTTTCTTTCTGTGTGTATGTGTGT

CCAATTTTCTCTTTTTATAAGAATACCAATCATATTGAACTAGGGCCCTG

ACCTCATCTTAACTAATTATATCTGCAATGACCCTATTTCCTAATAAGGT

CGTATTTGAAGTACTGGGAGTTAAGACTTCAACATCTGGCTGGGTGTGGT

GGCTTACACTTGTAATCCCAGCACTTTGGAAGGCCAAGGCCAGAGGATTG

CTTGAGCTCATGAGTTTGGGACCAGTCTGGGCAACATAGTATCACCTTGT

CTCTAAAAAAAATACAGAAAACAGGCCTGCGCGGTGGCTCACACCTGTAA

TCCCAGCACTTTGGGAGGCTGAGGCGGGCGGATCACTAGGTCAGGAGATC

GAGACCATCCTGGCTAACGCGGTGAAACCCTGTCCTGCGAAAAATATAAA

>hg19_ct_ARAlincRNAs_9727_ARAlincRNA_0017.1 range=chr1:51441717-51443912 5'pad=0 3'pad=0 strand=- repeatMasking=none

CAGGCACAGGTCGCCAGAACCGTCCAGTGGGATGGCTGCCGCCTCCTCGG

GGACCGCTGCCTGAGGGCCAGGACCGGGGTCGCCTGGCCGCCCGGGGAAC

CCGCAGCTCCCGGGGCGCTGCGCCGGCGGCCGGGAGGCGCCGGCGCCCGC

TCCCCCTCCCCGCCCCCCCGCTCCCGCCTCCTGCCCGCGGCGCGGTGCTC

CTGCTCGCCCCGGCCGGCCGCGCGGCCGCGCACACACTCACTCCGAGGGC

GCCTCCAAGGTTCCTGCGTGGAGCTGTTTTCATGGGACTGCCATATAAAA

GCAGAACAGTCTCCAGAGACCGAGACAGGGACTTTCCCCCGTAGCCTTTT

GATGAAGGTTGCTTAGGGCAGCCAGGAAGGCTGTGAGCACGACCTTTTCT

TGCTCCAGCACTGTGACTGCCCGGCGCCTATGGACTATTTACTTCTACCC

CCGGCAAAAGCCGAAAGCACCTTTTTGGGAACTGAGATTTCGAAACCAGT

CCTGGGCCACGGACTCCATCCACAACGTTCTTTTGATCAATACGATAAGG

GAAATACATCATATTGCTTTCTCGGGACAGAATACTTAACCTACTTATTG

ATCAGTCGCCCATTGTGGTGTGTGTGTACATGAACTCATCACACCGTTGC

AAGATGCATAATGTTTATCAGAAAACTTAATTCACTACCAAAATGTGATA

AAAGACACTTTGTTTAGCAGTTCGGGGACCAGAACTTGAACCGAGGTCAT

GTTAGTGCCCTCCACCCCCAGGTGTAAGCCTGGCTTCAGCTTCCTGGGCA

AAGGCTGAGACCAAGTTTCCCGACGTGAATGTGGACGCAGCGGTGAAGAA

CTGAATTTGAGAGCGCATCCTCTGCTTTGTATCCTCCATCCTTACAACAG

CGTCGTCTCCTGGAGCTCTGGACACTCCATTAATTAGCACTTGCATCCCC

ACTCTCTGCATCTGAATAAGTTCAGACCCCTCCCTCCCCACCTCAGGTCG

CTGAAAAATAGGTCCCCAGCAGCACCGTTTCATCCATCCTATACATTTCG

GCATGTTCAAGAGGCTTACAGAATATTGTTGAGGTTTTTGAGATTGAATA

ATGGCTTTTCATATTTCCTAGAGGGGACCACATAACTCATTTCTAAAATA

CTTAGATTAGCAAAGCTACTTCCCGCACAGAGCTAGAGTAATATGGCGAT

GTTTTAATGCCTAACAACTTTTAGACTGATGAAAAGATAGAGCGTCCTCT

CCCTCTATTGTGCTCCGACCACCCGAGAACAAAGCAACTGCAGAAGTAAC

TCCTCTTTTGCTTTATGAATGTCCTATGCTTTTATATTTGAAATCTCTAA

AGGGAAATTTTATTCTATTAAATAAAGTAAATGCCGAGACATGTTGGATG

CTGTGAAGTCCATTAAACTGGATTTTAAAATCATGACCGTCCATCTTTAC

AGTTGTAACTTGTAAG

>hg19_ct_ARAlincRNAs_9727_ARAlincRNA_0017.2 range=chr1:51442717-51443912 5'pad=0 3'pad=0 strand=- repeatMasking=none

CAGGCACAGGTCGCCAGAACCGTCCAGTGGGATGGCTGCCGCCTCCTCGG

GGACCGCTGCCTGAGGGCCAGGACCGGGGTCGCCTGGCCGCCCGGGGAAC

CCGCAGCTCCCGGGGCGCTGCGCCGGCGGCCGGGAGGCGCCGGCGCCCGC

TCCCCCTCCCCGCCCCCCCGCTCCCGCCTCCTGCCCGCGGCGCGGTGCTC

CTGCTCGCCCCGGCCGGCCGCGCGGCCGCGCACACACTCACTCCGAGGGC

GCCTCCAAGGTTCCTGCGTGGAGCTGTTTTCATGGGACTGCCATATAAAA

GCAGAACAGTCTCCAGAGACCGAGACAGGGACTTTCCCCCGTAGCCTTTT

GATGAAGGTTGCTTAGGGCAGCCAGGAAGGCTGTGAGCACGACCTTTTCT

TGCTCCAGCACTGTGACTGCCCGGCGCCTATGGACTATTTACTTCTACCC

CCGGCAAAAGCCGAAA

>hg19_ct_ARAlincRNAs_9727_ARAlincRNA_0018.5 range=chr1:53793905-53802181 5'pad=0 3'pad=0 strand=+ repeatMasking=none

CACTCTTCTCTCAGCGGGCGCTCCACGGCCCTGCTCTGGGCTCGCCTGAG

TGCTGGGCTCTGCAGACGCAGGGATGGGCCAGGCGAGCCCCTCACCACGG

AGGTAGCAGGTCCAGAGGAAGCAGCAGAGGCTTCCTGGAGGAGATTAATT

TACCCGAGGTCAACCACGGTCCAAAGATATTAAATGGAAAATTCCAGAAA

TAAACAATGTGTAAGTTTTCAGTTGCATGCTATTCTGAGTAGCAGGATGA

AATCTCGTGCTGTCCGGCTTCGTCCCACCTGGGATGTGAATCATCCCTTT

GTCCAGTGGATCCGCGCTATAGACACTACTCTCCCATCAGTCACTCAGCA

GCTGCTTCAGTTCCCAGATCAACTGTCGCAGGATCGCAGTGCTTCTGTTC

AAGTAATCCTTATTTTACTTCATTATGGGCTCCAAAGCACAAGAGGAGTG

TTGCTGGTGATTCCGATATGCCAAAGGAAAGCT

>hg19_ct_ARAlincRNAs_9727_ARAlincRNA_0018.4 range=chr1:53793905-53802889 5'pad=0 3'pad=0 strand=+ repeatMasking=none

CACTCTTCTCTCAGCGGGCGCTCCACGGCCCTGCTCTGGGCTCGCCTGAG

TGCTGGGCTCTGCAGACGCAGGGATGGGCCAGGCGAGCCCCTCACCACGG

AGGTAGCAGGTCCAGAGGAAGCAGCAGAGGCTTCCTGGAGGAGGATTTGT

ACCCCTAGGAGGACTATGTCTTCCTCATCTTTGTCTTCAGAGGATAAATA

CAAGGCCTGACACATAACAAGCATGAGATTAATTTACCCGAGGTCAACCA

CGGTCCAAAGATATTAAATGGAAAATTCCAGAAATAAACAATGTGTAAGT

TTTCAGTTGCATGCTATTCTGAGTAGCAGGATGAAATCTCGTGCTGTCCG

GCTTCGTCCCACCTGGGATGTGAATCATCCCTTTGTCCAGTGGATCCGCG

CTATAGACACTACTCTCCCATCAGTCACTCAGCAGCTGCTTCAGTTCCCA

GATCAACTGTCGCAGGATCGCAGTGCTTCTGTTCAAGTAATCCTTATTTT

ACTTCATTATGGGCTCCAAAGCACAAGAGGAGTGTTGCTGGTGATTCCGA

TATGCCAAAGGAAAGCTGTAAAGTACTTCCTTTAAGTAGAAAGGTGAAAG

TACTTGACTTAATAAGAAAATTTAAAAAACAAAATTGTATGCTGTGGTTG

CTAAGATCTACAGTAAGAATGATTCTATTCATGAAATCGTGAAGAAGGAA

AGAAATTTGTGCTAGTTTTGCTGTTGCAACTCAAGCTGCAAAAGTTACAA

CACCTGTGTGTGATGAGTGCTTAGTAATGATGGAAAAGCCATTAAATTTG

TGGGTGCAAGACACGGACAGAAACGTTCTGATTGACGGCAATCAGGTTCC

ATTGGGGGTATTGGAACACATCCCTGAGGATAATGGGGGACTACTGTATT

CTACCAACTTATTCCTCTTGAAGAATTCCCTTTCGGATTTCTGACTGCTG

AGTCCTGGACTGGTTGATGGCAAGGCCAGCTGCCCTGGTTGTCACCTGGA

GACTGTGCTGCACTTTTCTCCTGGGTCGAACCCCTCAATTCCTAGATCCC

ACGTCTTCCCCTTCTGGGTTCACTACCTTATTTGGGGGAACATATACCCA

TAGCTTTCTTTTTGTTTTATTTTTTGTTTACCTATTTATTTATCTTATTT

TATTTTTTGAGACGGAGTCTCCCTCTGTTGCCCAGTCTGGAGTGCAGTGG

TGTGACCTCGTCTCACTGCAGCCTCCACCCCCCAGGTTCAAGCAATTCTC

CTGCTTCAGCCTCCCAAGTAGCTGG

>hg19_ct_ARAlincRNAs_9727_ARAlincRNA_0018.2 range=chr1:53793905-53804931 5'pad=0 3'pad=0 strand=+ repeatMasking=none

CACTCTTCTCTCAGCGGGCGCTCCACGGCCCTGCTCTGGGCTCGCCTGAG

TGCTGGGCTCTGCAGACGCAGGGATGGGCCAGGCGAGCCCCTCACCACGG

AGGTAGCAGGTCCAGAGGAAGCAGCAGAGGCTTCCTGGAGGAGATTAATT

TACCCGAGGTCAACCACGGTCCAAAGATATTAAATGGAAAATTCCAGAAA

TAAACAATGTGTAAGTTTTCAGTTGCATGCTATTCTGAGTAGCAGGATGA

AATCTCGTGCTGTCCGGCTTCGTCCCACCTGGGATGTGAATCATCCCTTT

GTCCAGTGGATCCGCGCTATAGACACTACTCTCCCATCAGTCACTCAGCA

GCTGCTTCAGTTCCCAGATCAACTGTCGCAGGATCGCAGTGCTTCTGTTC

AAGTAATCCTTATTTTACTTCATTATGGGCTCCAAAGCACAAGAGGAGTG

TTGCTGGTGATTCCGATATGCCAAAGGAAAGCTGTAAAGTACTTCCTTTA

AGTAGAAAGGTGAAAGTACTTGACTTAATAAGAAAATTTAAAAAACAAAA

TTGTATGCTGTGGTTGCTAAGATCTACAGTAAGAATGATTCTATTCATGA

AATCGTGAAGAAGGAAAGAAATTTGTGCTAGTTTTGCTGTTGCAACTCAA

GCTGCAAAAGTTACAACACCTGTGTGTGATGAGTGCTTAGTAATGATGGA

AAAGCCATTAAATTTGTGGGTGCAAGACACGGACAGAAACGTTCTGATTG

ACGGCAATCAGGTTCCATTGGGGGTATTGGAACACATCCCTGAGGATAAT

GGGGGACTACTGTATTCTACCAACTTATTCCTCTTGAAGAATTCCCTTTC

GGATTTCTGACTGCTGAGTCCTGGACTGGTTGATGGCAAGGCCAGCTGCC

CTGGTTGTCACCTGGAGACTGTGCTGCACTTTTCTCCTGGGTCGAACCCC

TCAATTCCTAGATCCCACGTCTTCCCCTTCTGGGTTCACTACCTTATTTG

GGGGAACATATACCCATAGCTTTCTTTTTGTTTTATTTTTTGTTTACCTA

TTTATTTATCTTATTTTATTTTTTGAGACGGAGTCTCCCTCTGTTGCCCA

GTCTGGAGTGCAGTGGTGTGACCTCGTCTCACTGCAGCCTCCACCCCCCA

GGTTCAAGCAATTCTCCTGCTTCAGCCTCCCAAGTAGCTGGGACTACAGG

TACGTGCCACCATGTCTGGCTAATTTTTGTATTTCTAGTAGAGATGGGGT

TTCTCCATGTTGGCCAGGCTAGTCTCAAATTCCTGACCTCAGGTGATCCA

ACCGCCTTGGCCTCCCAAAGTGCTGGGATTGCAGGCATAAGCCACTGCGC

CTGGCCCCATGTATACCTATACTTTTCTAAAAGGATGTGTTTGGGGTGAA

ATCTTAAGACCTTGCCTTTCTAAAAGTGCCTCACATTTAATCAGATAGTT

TGACCAGGTATAAATTTCTAAACTGGAAATATTTTTCCCTCTGAATTTTG

AAGGCTTGCTCCATTGTCTTCCAGTTTCTGCTGAAAAGTCTGAGGCCATT

CTGTTTCCTAATCTTTTCTAGAGACTCTGTTCTCTCTTTCTGGAAGCTGT

GTCCTCAGTGGGTCTTTTCTTTTAAGAGAAGGGATCCCATCGCCACCGAG

GCTGGAGTTCAGTGGCACAATCATCGCTCACTGCAGCCTTGACCTCCTGG

GCTCAAGGATCCTCCTGCCCCAGCCTCCTGAGTAGTTAGGACTACAGGCT

CATACCACCATGCCTGCTCAGCCTTAGTGTGTCTTGAAGTGAATCGATTT

CCATTCACTGTGCTGGGTACCCAGTGAGTCCTCACGGACAAACTAATCAT

CTTCATTTTGGAGAAAATTTAAAAGAAGTACCTCTATAATGATTTTATTT

TTTCTTTCAGGAACTCCTTAATTATTCAAATACTGGCCCTCCTGGAGCAG

TTCTCTATATTTCTTATATCCTTTTTCCACCTATTTTTCATCCCATTTTG

CTCTTCATTCTGGAAATGTTCTTCAACTCTTCAACAGTTTTTTGTTGTTT

TTCAAAAATTTATCTTATCATATTTCCAAAAGCCTTTTGTTTGTTCTTTG

ACTATTCCTTTTTTTAGAGTATCTCGTTCTTTTCTCACGGATGCAATATT

TTCTCTTATTTCTTTGAGGATGTTAGTAATGTTTTGTTGTTGTTTCTGTT

GAAGTGTTTCTCACCATGCCTAATCTCTTCTTTGTCCAAGTTAATTTTTT

TTTCTGATTGTTGAAAAGACAAGCCACATACTGGGAGAATATATTACAGC

ACAAATAACGCACAAAGCATTACTATCCGGAATACATAAGTAATTCTTAC

AAATTGGTAAGAAGACAACTCAATTAAAAAAGAGCAAAAGATATGAATGA

GCATTTCACAGCAGAAAAATATGAATGAAAAATAAACCACAAGAAAAGAT

TCTCAGCCTTATAGAAATCAGGAAAATGTAAATTAAGACCACAGGAAGAT

TTTCCATGGAAAAGAAGTCCCATAGTGGCAAGTATTGGAGAGGCAGGAAT

CAACAGGGATCCTTATATGCTCCTAATGCAGTGTAAATTGCTGCAACCAC

TTTAGAATATTGCGATTTCATTACTAAGTGTAAACCCTGGAGATACACTT

GCATGCAAGCACCCAGAGACAGGTGCAAGGATATTCATAGCAGTGCTGTT

TATTATGGTAAAAACATGGAAACCACCCTAATGTCCTTAACAGTAAAATT

AATTAATTGTGGCATCACCATATAATGAAATCCTATACAGCAGTGGCAAT

GAGTCTAACATAGCTGTGGCTGGATACAGAAGGAATCATAGAATTATAAT

GCGGAGTGAGAAAAGCCTGTTGCAGAAGACTACATACAACAGGATTTGAC

ACTTGTAAGGCTCCAAAACAAAGAAAATTAAATGATATTGTTTAGGTTTT

CATACATAGGTGATAAAAGTGTGTTTCTTTGTTTTTAATGAGAAAATTAG

TCACAGAATTTAAGATCTTAGTTACTTCTATAGGGAAGGCAGGGGAATGG

GACAAGGAGGAAGCCCACAGCATTGGTCATGCTCTCATGTTGAAGTTGGG

TTCAAAGGTGTTCATTATTAAAATGCTTCATAATGATGACCATACATTTG

GTATTTCTAGGACAATCTTGGTTTACATCTATTGTCTCAACATAATTATT

CAGTGCAAGCCTTTCCTTTCTCAGAAAGGTCCT

>hg19_ct_ARAlincRNAs_9727_ARAlincRNA_0018.1 range=chr1:53794672-53796923 5'pad=0 3'pad=0 strand=+ repeatMasking=none

ATTTCAGCCTCAGACTTGTGAGCCGGGTCCAACCCACACAACCTTCTTTA

GAGAATGAGATTCCAGGGCATTGGCCACTCAAAACAACCGGCCTGCAGGT

GCCAAGAGCCAGTGTTAGGTCCACCCTCCCTCCCCTCTTGCCCCTTCACC

TCCCTCGCGGATGGGCAGGTGCTGTTAGCCCTTGGCCTGGAAACCACCCT

GTCTCCTCAGCAGCCTCCTGAGCCGTAGTCCCCCAAAGGGCAGCCCAGCG

GCAATATCTGTGGCCACCCCACCAGAAAGGGACTTAGATCCCCAGAGAGC

CCGGCACGGTGCTCCTGCCATCATCACCCCTCCTGTCCCTTGTTCATTCA

GTCATTCAGCAAGCATTTCAGGCCCTGTTCTGTGCCAACCCTGTGCTGGG

CCTGCGGGTACCAAGGTGAATCAGGCATGGTGCCAGCCCCGGAGAGGTCC

CTGACCTGTGGGGAGACAGACCATTAGCACACAGACAACGAAATGCAGGC

GTCCTTTCACCTATATGTGCCAAACGCCCTTCAAGGCTCAAGGATACAGT

GGAAACAGATAAGGTTCCTCCCTGCTCTCCTGCAGTTATGTCTTAGTTGG

AAGAGATACACACAAAAGCAGGAAAACAAAAGATTATTTCAGGTCATCAT

AACTTCTATGGAAAAAACAAAACAGACCCAGGTGAGAGCAATGGGGGTAA

CTGTAGAGGTGATCAGGCAAGGCCTCACAGAGGAGGTGACATTTCACTGA

ATGGCAAGATGGATCCTGACCCATGAAGATCAGGCGGAAGGGCATTCTTG

GCAAAGGGAATGGCAATCAAGGATTTGTACCCCTAGGAGGACTATGTCTT

CCTCATCTTTGTCTTCAGAGGATAAATACAAGGCCTGACACATAACAAGC

ATGAG

>hg19_ct_ARAlincRNAs_9727_ARAlincRNA_0018.3 range=chr1:53796138-53804931 5'pad=0 3'pad=0 strand=+ repeatMasking=none

CTGTCTCAAAAAAAAAAAAAGAAAGAAAGAAAAAGAAAGAAAGGAAGGAA

GGAAGGAAGGAAGGAAGGAAGGAAAAAAGAGAGAAATGGAGAGCATGGGC

CTGTGGGTGAGACTGCCTGGGTCTGAGTCTGTTCCTTCCCTTTCTAGTTA

TGTGAACTTGGGCAACTCAATCTCTGTGGCTCTAAATGTGAAAAATGAGA

AAGGTGATAGTACTCACCTCACAGCATTGTGGTGAGGATATGAGATGAGA

TTGTGTACGCACAGCATACAGTATATAGTAGCTGCATGAGAAATGTTAGT

TATTGTTACTATTATTCAAACATAATTCCCTGTTCACAAAACCCTGATGG

CTTTCCAATACCCAGAATCAAGACTGAGCCCAGGCATCCCCCGACCTCCC

CAAGGGAGAAGGATGCCAGTTGCCAAAGTGGGCAAATCCTTGTCTCTCAA

CAGTCCCCTCAGCAGAGGTCCTTCTGGCTCCTCTCAGCCTCCACCCACTT

TGGGCCACTTCCCCGGGCTCCTGCAGTCCCTGGGCTTCCCTTTGCCGCAG

CCCTGACCACCCTGTGTTGCCACAGTCTGCTTATGCAACTGTCTCCCTCG

TCACTCTGTGAGCTGCTGGAGGACAGGAGCCCGCTCTCCTGCATTCCTGG

ATTCTTGGCACAGGGCACATTTGACCAATGCTTGTTTCTGCCTTTATTGA

AGGATTTGTACCCCTAGGAGGACTATGTCTTCCTCATCTTTGTCTTCAGA

GGATAAATACAAGGCCTGACACATAACAAGCATGAGATTAATTTACCCGA

GGTCAACCACGGTCCAAAGATATTAAATGGAAAATTCCAGAAATAAACAA

TGTGTAAGTTTTCAGTTGCATGCTATTCTGAGTAGCAGGATGAAATCTCG

TGCTGTCCGGCTTCGTCCCACCTGGGATGTGAATCATCCCTTTGTCCAGT

GGATCCGCGCTATAGACACTACTCTCCCATCAGTCACTCAGCAGCTGCTT

CAGTTCCCAGATCAACTGTCGCAGGATCGCAGTGCTTCTGTTCAAGTAAT

CCTTATTTTACTTCATTATGGGCTCCAAAGCACAAGAGGAGTGTTGCTGG

TGATTCCGATATGCCAAAGGAAAGCTGTAAAGTACTTCCTTTAAGTAGAA

AGGTGAAAGTACTTGACTTAATAAGAAAATTTAAAAAACAAAATTGTATG

CTGTGGTTGCTAAGATCTACAGTAAGAATGATTCTATTCATGAAATCGTG

AAGAAGGAAAGAAATTTGTGCTAGTTTTGCTGTTGCAACTCAAGCTGCAA

AAGTTACAACACCTGTGTGTGATGAGTGCTTAGTAATGATGGAAAAGCCA

TTAAATTTGTGGGTGCAAGACACGGACAGAAACGTTCTGATTGACGGCAA

TCAGGTTCCATTGGGGGTATTGGAACACATCCCTGAGGATAATGGGGGAC

TACTGTATTCTACCAACTTATTCCTCTTGAAGAATTCCCTTTCGGATTTC

TGACTGCTGAGTCCTGGACTGGTTGATGGCAAGGCCAGCTGCCCTGGTTG

TCACCTGGAGACTGTGCTGCACTTTTCTCCTGGGTCGAACCCCTCAATTC

CTAGATCCCACGTCTTCCCCTTCTGGGTTCACTACCTTATTTGGGGGAAC

ATATACCCATAGCTTTCTTTTTGTTTTATTTTTTGTTTACCTATTTATTT

ATCTTATTTTATTTTTTGAGACGGAGTCTCCCTCTGTTGCCCAGTCTGGA

GTGCAGTGGTGTGACCTCGTCTCACTGCAGCCTCCACCCCCCAGGTTCAA

GCAATTCTCCTGCTTCAGCCTCCCAAGTAGCTGGGACTACAGGTACGTGC

CACCATGTCTGGCTAATTTTTGTATTTCTAGTAGAGATGGGGTTTCTCCA

TGTTGGCCAGGCTAGTCTCAAATTCCTGACCTCAGGTGATCCAACCGCCT

TGGCCTCCCAAAGTGCTGGGATTGCAGGCATAAGCCACTGCGCCTGGCCC

CATGTATACCTATACTTTTCTAAAAGGATGTGTTTGGGGTGAAATCTTAA

GACCTTGCCTTTCTAAAAGTGCCTCACATTTAATCAGATAGTTTGACCAG

GTATAAATTTCTAAACTGGAAATATTTTTCCCTCTGAATTTTGAAGGCTT

GCTCCATTGTCTTCCAGTTTCTGCTGAAAAGTCTGAGGCCATTCTGTTTC

CTAATCTTTTCTAGAGACTCTGTTCTCTCTTTCTGGAAGCTGTGTCCTCA

GTGGGTCTTTTCTTTTAAGAGAAGGGATCCCATCGCCACCGAGGCTGGAG

TTCAGTGGCACAATCATCGCTCACTGCAGCCTTGACCTCCTGGGCTCAAG

GATCCTCCTGCCCCAGCCTCCTGAGTAGTTAGGACTACAGGCTCATACCA

CCATGCCTGCTCAGCCTTAGTGTGTCTTGAAGTGAATCGATTTCCATTCA

CTGTGCTGGGTACCCAGTGAGTCCTCACGGACAAACTAATCATCTTCATT

TTGGAGAAAATTTAAAAGAAGTACCTCTATAATGATTTTATTTTTTCTTT

CAGGAACTCCTTAATTATTCAAATACTGGCCCTCCTGGAGCAGTTCTCTA

TATTTCTTATATCCTTTTTCCACCTATTTTTCATCCCATTTTGCTCTTCA

TTCTGGAAATGTTCTTCAACTCTTCAACAGTTTTTTGTTGTTTTTCAAAA

ATTTATCTTATCATATTTCCAAAAGCCTTTTGTTTGTTCTTTGACTATTC

CTTTTTTTAGAGTATCTCGTTCTTTTCTCACGGATGCAATATTTTCTCTT

ATTTCTTTGAGGATGTTAGTAATGTTTTGTTGTTGTTTCTGTTGAAGTGT

TTCTCACCATGCCTAATCTCTTCTTTGTCCAAGTTAATTTTTTTTTCTGA

TTGTTGAAAAGACAAGCCACATACTGGGAGAATATATTACAGCACAAATA

ACGCACAAAGCATTACTATCCGGAATACATAAGTAATTCTTACAAATTGG

TAAGAAGACAACTCAATTAAAAAAGAGCAAAAGATATGAATGAGCATTTC

ACAGCAGAAAAATATGAATGAAAAATAAACCACAAGAAAAGATTCTCAGC

CTTATAGAAATCAGGAAAATGTAAATTAAGACCACAGGAAGATTTTCCAT

GGAAAAGAAGTCCCATAGTGGCAAGTATTGGAGAGGCAGGAATCAACAGG

GATCCTTATATGCTCCTAATGCAGTGTAAATTGCTGCAACCACTTTAGAA

TATTGCGATTTCATTACTAAGTGTAAACCCTGGAGATACACTTGCATGCA

AGCACCCAGAGACAGGTGCAAGGATATTCATAGCAGTGCTGTTTATTATG

GTAAAAACATGGAAACCACCCTAATGTCCTTAACAGTAAAATTAATTAAT

TGTGGCATCACCATATAATGAAATCCTATACAGCAGTGGCAATGAGTCTA

ACATAGCTGTGGCTGGATACAGAAGGAATCATAGAATTATAATGCGGAGT

GAGAAAAGCCTGTTGCAGAAGACTACATACAACAGGATTTGACACTTGTA

AGGCTCCAAAACAAAGAAAATTAAATGATATTGTTTAGGTTTTCATACAT

AGGTGATAAAAGTGTGTTTCTTTGTTTTTAATGAGAAAATTAGTCACAGA

ATTTAAGATCTTAGTTACTTCTATAGGGAAGGCAGGGGAATGGGACAAGG

AGGAAGCCCACAGCATTGGTCATGCTCTCATGTTGAAGTTGGGTTCAAAG

GTGTTCATTATTAAAATGCTTCATAATGATGACCATACATTTGGTATTTC

TAGGACAATCTTGGTTTACATCTATTGTCTCAACATAATTATTCAGTGCA

AGCCTTTCCTTTCTCAGAAAGGTCCT

>hg19_ct_ARAlincRNAs_9727_ARAlincRNA_0019.1 range=chr1:59012781-59013230 5'pad=0 3'pad=0 strand=+ repeatMasking=none

GAGTATGGGAACAGTGCTTTCATATGATTTTTGGAGGGACGCATTTTAAG

AAAGATGAAGTATAATCTTAAGTGTCTGGAGGTTAAAGTAGCTGGCTCCA

AAATTATGTGTATAGTATGTTTCATTCATTTCACCAAAACATATTTCTTG

AGCATCAACTACATTTTAAGTGCCAAGGATGTAGCAGTGAACAAGACAGA

CCTGAATTTCGTGAACTTTATATCACAGTGAGGAAAGACAGGCGATTAAA

AAAAAGTGTACAAATAAACACAAATAAATAAAAGTGTACAAATAAGCCAG

TACAAATAAACACTGGATTGTGATGGATGAGTAGTGAGGTCTCATATTAG

TTTATTATGTTTACACTCTGTGCACAATGAAAACATCTCAAAGGACATAA

ACACTATGTCAAAGGACATAAACACTATGAACAATTTTAGGTATCTCTTT

>hg19_ct_ARAlincRNAs_9727_ARAlincRNA_0020.4 range=chr1:61005013-61053834 5'pad=0 3'pad=0 strand=- repeatMasking=none

GAATAAAATTATACGTACACATCTTAATTTCTTGGGATTTGATTACTTCT

TCTGCTTTGAAATTAGACCAAGTTTAGCCTGCCTTTGCTATTTTGGCGTA

TCTCCAATTCTTCATGATTTTCTCATAGTGTTGCCTCCATATGATGCAAA

ATATCCAACCTAGCCCTGACATGAAAACTTAATTGAAAGTATCCAACAGT

GGGAGTGACCCAAGGCAAACTTACAAAAAGAAGCACATGGCATGTGGAAA

GAAGAGAAGATTCAAAATCAGTAGTCAGGATTTGAGCCTCTTCTCTTCTG

CTTTCTAGCTGAAAAATGTCGAACAAAGAATTCAACATTACTTTTCTGAG

CCTCTCTTTCCTCTTCTATGGAATGGAAGTAATAAAATATCTACCTCCCA

GAGTTGTAGTTCAGACCCAACACATATATTTTTAGTGCTCTATCATATGT

TAGTTTCTGAACTTTGCTCTTTCTCAGGAAGCATCCCACTGAATTAAAAA

CATCTGTTGAGTTAAATACTATTGTCTCTTCAATCAAATGGAGAAATTGA

GGCTAATGAAGATAAAGAGCCTTGTTCCATTTCATACATCTAGAAGTAGA

AGAGCTGGTATTAAAAATCCTACCTGTTGGACTTTATATCAGGTGCTCTT

TTCCCAAACTACAGCTGCTTTATAAAGAAGCAAATGATAATTTTTAAAAC

AACACAATAGCAAAGCCATGGAATCAACTCAGTTTCCCATCAACTGTGGA

TTGGATAAAGAAAATGTGGTACATGTATATCATGGAATACTATGCAGACA

TAAAGAAGAACAAAATCGTGGCTTTTGCAGCAACATGGATGCAGCTGGAG

GCCATTACTCTGAGTGAACTAACGCAGAAACAGAAAACCAAATACCCTTT

GTTCTCCCTAATAAGTGGGAGCAAAACGATGGTACACGTGGACATACAGA

TGGGAACAGCAGACACTGCGGACAATTAGAGAGGGGAGAGAGGGAGGAGG

GCAAGGGTTGAAAAATGACACATTTTATTACCTGGGTGATGGATTCATTT

GTACTCCAAACCTCAACATCACTCAGTATACCTTTATAACAAATCTACAC

ATGCACCCTCCGATTCGAAAATAGAAGTTGAAAAAAACTGTATTTTCTCT

AAAAAATTTTGCTTATGTACTAGTTGGTGCCCTTCAAGTGGGAAAAATCC

ATGCCTGCTGAACTGTCACCTCTTTTTTTTTTGGAGGGCTTCTATTTTTC

AAGTGCCCTGTGTCTTCTTGGGCTTGTTTTTTATTCTAGACAAAATGCTT

CCACACACTGGGGGCTTTACCTGGCCTGCTCATGAAGGCATCATCTGGGT

CCAAACTCCTTATTGGCTACTTATCAAAATGGATCTTTCTCTGCTCTTCT

TTTGCATTAGATTCTTGCTAAGAACCTAGAATTTGCCTGATAGATTGACT

TACCCTAAAGTTAAAAAGGCTTTTATTAGCTTATTTTATGGCTCTATATT

GTGTATTTCCTTTTAAACAGAGGTTCCTTAGAGACTCAATTCCTTAATCC

AGCATTTCTTAAGTTTGCTTTTGGGAAGGTTAGTACAAGAAGATGGTCTC

TTTAAAAATAATTCTTGAGAATCACTATACTGGTTAACATATTAAAAGAA

CCAAGGAGTCCTACAGATTTTTAAAAAGCCTTTGCTTATTGATACTCTTT

GGTTTGACTGCCAATTCTCTTTTTGTGGATCATCTGCTAATATCTAATAA

ATTTAGCATGACACAGAACAAATGTTGAGAAACAGTCAATAAAAAACTGT

CTTTGTGAAAATTTTGGCATTGCACTATCTGGAAAAAGTGATTTTTGTGA

GTAGAGCCATGTGAGTATGCTTCTAAAAACACAACATATTGGCATTATTC

TCAGGAGTAGATCCAGGTTTTGTGGCACTTGAAGATTATACAGTTGATGG

GTGACTAAGAAAAAAATTACTGTGTTAATCAAAATTTAGGTTTTGAAAGG

GGCCTGTGTAAGCAAAGAGCCCACAGAGTAAGCTTCATTGTCTGTATTGT

CAATCTACAAAAGACTGACCTTTTTCAAGTGAGTATAAACCTGGCCTGGC

TTATGTTCATGTGGTGAGTGAAAGAATAGTAAACCAGGAAGAAGAAGGCT

TATGTCCTTGGTCAGAATTGTCCATTTTACTTCAATTCCCTAGACTCTGT

GACTTTGGATAAGTCACTTTATTTCTCATCATCAGTGACTTTATTTTTAG

AAAGGGGTATTAATTGTCCTCTGGATGATAAAGAGTCATTTTGAAATTAT

AATTAATGGGGAAGCATTTAGCACTATACAAATAGAGGTTATGATTCTTT

TCAAAATGCTTATTAATTCTGAGGACTACACATATGTTAACAATCATTAT

TGCTAACATATCAAATAAAAACAAATCTGGACTTAAGTAAATGGAGATTT

TATTTTTAAAATATTGCAATAGAAAAGGTAGACAATTGCAATAGATTAAT

GACCCTAAGATCTCCATCTCAAATGTCAGGCAGAGGACAGCTTTTTTTGT

TCTTTATGGCAAGGAATAAACAAGGCTAGAAAGAACAGGTATGGGGCGTG

GAAAGTGGGTAAGTGAGTGGCATGATGAGACAGTTGATATCCCTTCCCCT

GTCTCCCCATTTCAGCTGATTTTCAGGAGGGGACAGTAAGGAGAGGTTGC

TCCAAGTTCCATTGCTCAGCAGAGGATCAAAGATAAGAGACCTGGGGAGA

GGTGAGACACTTGAGTATAGTTTAAGCAAATCAAATTGGTAGGCATTTTG

TCCAGGTTGGTGAGTGGAAAGAAACAGTTAAGCTAATCATTTATGAGGCA

AAGGGTGGGAATTTGGAGGATCTGTGTCCAGCCTTGTCAAAGGCAAAAAA

GGAGTCATTCACAGGTCTTTTTTAAGTTATGTGCAGAAGAGTAGTTCTTT

GCAGTAAGCTATTCTTGGGGTACATATTAGGATAAGGGAATGTCTTAACT

TTTGCTGTTTTCCAGGAGCACAGGGCTCAGGTAAAGTTCAATATTATCAA

ATATCAACACCATCATTGCTTTTCTTGTGTCTGGTCAGTCATTCAAAATG

CTGTATCTTTGGAAAAATTACTTAACTTTTCAGACCTTCAGTTTTCTCAT

CAGCAAAATGAGAATAATTATAATAATAACTAGCTCACTAGGATACGGTG

ATTCTTGAATGACCTAATGACTATGCAAGCACCTCATAAGCTATAAATAT

TGTGGAAATATTATGCATTTTTTTGTTGTCATTGTTAGTAGCTCTATAGG

CTCTGACTTCTTCATTTATTTGATGCAGCAAAAATGGGGAAGGGATGAAA

CATTGTTTAAATGAATCCTAGATACCAGACACAGGGGTGCCCCATACTCT

TTCTCTCACCAGGTGCATACAACCCTGAATTGTCATGTGCATGAAATCAT

CTACTTTCATCCAAGGAATATGGAAAAGAGATGGGCAAAGAGATAACTCA

GCTACATTTGCCTGTTAATAATTGATTTATTTTACTCCGCAAAGGCCACA

TAAGGACAGTGAGAAGGCAGCCACCTGCAAGCCAGGAAGAAAGGTCTCAC

CAGATACCAACCCTGCCAGCATGTTGATCTTGGACTTCCAGCCTCCAAAA

CTTCCTCAGCTCTCAGCTGATGTGGCTATAATTTTTATTCTGTAAGGTGA

TTTTTTAGGACTTAATAAGATCTAAAGCCCTGTAAGTTCAAACAGAAGGT

GCTAAATAAACAGAGTTAACTTGGAACCTTATACTCCCGTGTTAAAGCTG

TGGGTCTGAGTAACCACACTTTTCCTTCACAGGGCCTTCTGCTCACATGG

GCTTCCGTGCACCTGCTTCAGGAGACAGCAACACTTGCGCCCGCACCGCG

GCAGGCTCCCATCACCTGGTGGATATGCCGCGCCGAGTCTCATCACACCT

TAGTGAAGGAACACATGGACCCATATGTTAGCGGATGTATTTACATCGTT

TTATACAGAAAGGGGAAGTATGTTTCACAATAAATGTTTGCATACTTTGG

AGCACATCTTTTTTTTCTTTCAGACAGAAAGAAAGAGACAAAAAGAGCGG

GAGAAACTTTGGCTGGAGCCATTGATTCTTTATTCCCTTACAATGTTTTT

ATCACCCAACCTTGAAAATAGTCTGTGTGTCAGCTGGGCAGGATCTGTGC

TGAGGCTTGCGTCCCTGGGGGCAAAAAAAGGGTAGTGACTTTTAGTTGAA

AGAGTCAGGAAGGGAAGAGTCAATAGTTCAGCGGTAAGTATTCTGAACCC

AGGCAGGGAAACCAAAATTCAGGGATACCTGAAGCCCTCAAGCTAAGGGC

CAGGAGCTAGGAGCTGAAAGGATACAGGAGCTATGTCATAAATAATTTAG

GAGGTGGCTAGATGATTGCCAGAGTGTCCACAGCATGCTTTACTTGGCTT

TTATGGATACAATGTGCATAGGTTGCTTTTAGGAACAATAAAGAAATGTT

TATTAAACTAATTATGATTCAATAATATTGACAATAATGACAATGTTGAT

AGTATGCAATATAATAAACTGGTGTGCTTTTATTTCTCCCTTTTCCTGTG

CACATCTGTTTTACTCTGTGACATTTTTCCATTTAGACTCAGAAATAAGA

AACCAGTATTGCTGCAAAGGTAGGCTGTCACTGGAGGAAATGAAGATTAA

AAATTAAAAAATCAAATTATAATTTTTATATTTTAAAAAAACAGCTTAGG

TGTAACAGAGAAATACTTGGAAGACGTTAGAGTATGAGACAAGGAAAAAT

TTGCTCCAAACCTCCTCCCGCATAATCACCTCTAATAAACAAATATCCTA

GACAAATATAATTTTCCAAGCTAGGGGAAAAAAAGAACAGTTGGAGAATA

TAATGTACAAAAGTTAATCACCAAA

>hg19_ct_ARAlincRNAs_9727_ARAlincRNA_0020.2 range=chr1:61005921-61106163 5'pad=0 3'pad=0 strand=- repeatMasking=none

CTGCTTTGTTTACCCAAGCAAGCCTGGGCAATGGCGGGCGCCCCTCCCCC

AGCCTTGCTGCGGCCTTGCAGTTTGATCTCAGACTGCTGTGCTAGCAATC

AGCGAGACTCCGTGGGCGTAGGACCCTCCTAGCCAGGTGCAAGATATAAT

CTCGCGGTGCGCCGTTTTTTAAGCCGGTCCGAAAGCGCAATATTCGGGTG

GGAGAGACCCGATTTTCCAGGAATGTGCCATATGTGGAGACCTGCATGGA

AAGGAACTGAGGATGCTCTCTCACTAAAAGCTAGAAGGAACTGAGGCCCT

CAGTCAACAGTCTACAAGACACTGAATCTTGCCAGCAGCCAAGCAGTGAG

CTTGAAAGCAGATCTATCATCAGCTGAACCTTCAGATGAGACCGCAGCCT

AAGCAGATACCTTGGTTGCAGCCATGTGAGACAACCTGAAACAGAGGGCA

CAGCTAAGCCTCAGCCAGGTTCATGACCCTCTGAGGAAACTGTGAGAAAG

CAAATGTCATTTTAAGCTATTTAATTTTGGAGGTAGTTTATTACACAACA

ATAAATAACTATTATAATTATCTTGAGGATTTATTTCCTGGGGAGAATAC

ATGGAATTGCATATATAGGTCCTCATATGCAGAATACTGGTACCCTAGAG

ATGTTCACCCCAGAGATGTTTTAGGAACCTGTGAATATGTTACATTAAAT

GGCAAAGTTTTCCTGGATGGTTTAAGTTTAAGATGGAAAAGTTTTACTGG

ATTATCCAGTGGGTCCAATCTAGTAACATGAGTCCTTAAAAATGGAGAAT

ATTTTTTGGCCATGATCAGAGAGACATGTGATGATGGAAAAAGTGTCAGA

GATGCAATGTGAGGGGACTAAAACTGCTGTTGCTGACTTTAAAGATGGAG

AGAGGGGAACCCGAGTCAAGGAATGCAAATGGCCTCTGGAAGCTGGAAAA

AGCAAGAAATGGATTCTCCTTTAGAGCCTGTGGAATAAAATGCAGTCATG

CTGACACCTTGATTTTACCTCAGTGAGACCAATACTGGAGTATGACCTTC

GGAGCTGAATAAAATTATACGTACACATCTTAATTTCTTGGGATTTGATT

ACTTCTTCTGCTTTGAAATTAGACCAAGTTTAGCCTGCCTTTGCTATTTT

GGCGTATCTCCAATTCTTCATGATTTTCTCATCGCAAAGGCCACATAAGG

ACAGTGAGAAGGCAGCCACCTGCAAGCCAGGAAGAAAGGTCTCACCAGAT

ACCAACCCTGCCAGCATGTTGATCTTGGACTTCCAGCCTCCAAAACTGGC

CTTCTGCTCACATGGGCTTCCGTGCACCTGCTTCAGGAGACAGCAACACT

TGCGCCCGCACCGCGGCAGGCTCCCATCACCTGGTGGATATGCCGCGCCG

AGTCTCATCACACCTTAGTGAAGGAACACATGGACCCATATGTTAGCGGA

TGTATTTACATCGTTTTATACAGAAAGGGGAA

>hg19_ct_ARAlincRNAs_9727_ARAlincRNA_0020.6 range=chr1:61031445-61106163 5'pad=0 3'pad=0 strand=- repeatMasking=none

CTGCTTTGTTTACCCAAGCAAGCCTGGGCAATGGCGGGCGCCCCTCCCCC

AGCCTTGCTGCGGCCTTGCAGTTTGATCTCAGACTGCTGTGCTAGCAATC

AGCGAGACTCCGTGGGCGTAGGACCCTCCTAGCCAGGTGCAAGATATAAT

CTCGCGGTGCGCCGTTTTTTAAGCCGGTCCGAAAGCGCAATATTCGGGTG

GGAGAGACCCGATTTTCCAGGAATGTGCCATATGTGGAGACCTGCATGGA

AAGGAACTGAGGATGCTCTCTCACTAAAAGCTAGAAGGAACTGAGGCCCT

CAGTCAACAGTCTACAAGACACTGAATCTTGCCAGCAGCCAAGCAGTGAG

CTTGAAAGCAGATCTATCATCAGCTGAACCTTCAGATGAGACCGCAGCCT

AAGCAGATACCTTGGTTGCAGCCATGTGAGACAACCTGAAACAGAGGGCA

CAGCTAAGCCTCAGCCAGGTTCATGACCCTCTGAGGAAACTGTGAGAAAG

CAAATGTCATTTTAAGCTATTTAATTTTGGAGGTAGTTTATTACACAACA

ATAAATAACTATTATAATTATCTTGAGGATTTATTTCCTGGGGAGAATAC

ATGGAATTGCATATATAGGTCCTCATATGCAGAATACTGGTACCCTAGAG

ATGTTCACCCCAGAGATGTTTTAGGAACCTGTGAATATGTTACATTAAAT

GGCAAAGTTTTCCTGGATGGTTTAAGTTTAAGATGGAAAAGTTTTACTGG

ATTATCCAGTGGGTCCAATCTAGTAACATGAGTCCTTAAAAATGGAGAAT

ATTTTTTGGCCATGATCAGAGAGACATGTGATGATGGAAAAAGTGTCAGA

GATGCAATGTGAGGGGACTAAAACTGCTGTTGCTGACTTTAAAGATGGAG

AGAGGGGAACCCGAGTCAAGGAATGCAAATGGCCTCTGGAAGCTGGAAAA

AGCAAGAAATGGATTCTCCTTTAGAGCCTGTGGAATAAAATGCAGTCATG

CTGACACCTTGATTTTACCTCAGTGAGACCAATACTGGAGTATGACCTTC

GGAGCTAGTGTTGCCTCCATATGATGCAAAATATCCAACCTAGCCCTGAC

ATGAAAACTTAATTGAAAGTATCCAACAGTGGGAGTGACCCAAGGCAAAC

TTACAAAAAGAAGCACATGGCATGTGGAAAGAAGAGAAGATTCAAAATCA

GTAGTCAGGATTTGAGCCTCTTCTCTTCTGCTTTCTAGCTGAAAAATGTC

GAACAAAGAATTCAACATTACTTTTCTGAGCCTCTCTTTCCTCTTCTATG

GAATGGAAGTAATAAAATATCTACCTCCCAGAGTTGTAGTTCAGACCCAA

CACATATATTTTTAGTGCTCTATCATATGTTAGTTTCTGAACTTTGCTCT

TTCTCAGGAAGCATCCCACTGAATTAAAAACATCTGTTGAGTTAAATACT

ATTGTCTCTTCAATCAAATGGAGAAATTGAGGCTAATGAAGATAAAGAGC

CTTGTTCCATTTCATACATCTAGAAGTAGAAGAGCTGGTATTAAAAATCC

TACCTGTTGGACTTTATATCAGGTGCTCTTTTCCCAAACTACAGCTGCTT

TATAAAGAAGCAAATGATAATTTTTAAAACAACACAATAGCAAAGCCATG

GAATCAACTCAGTTTCCCATCAACTGTGGATTGGATAAAGAAAATGTGGT

ACATGTATATCATGGAATACTATGCAGACATAAAGAAGAACAAAATCGTG

GCTTTTGCAGCAACATGGATGCAGCTGGAGGCCATTACTCTGAGTGAACT

AACGCAGAAACAGAAAACCAAATACCCTTTGTTCTCCCTAATAAGTGGGA

GCAAAACGATGGTACACGTGGACATACAGATGGGAACAGCAGACACTGCG

GACAATTAGAGAGGGGAGAGAGGGAGGAGGGCAAGGGTTGAAAAATGACA

CATTTTATTACCTGGGTGATGGATTCATTTGTACTCCAAACCTCAACATC

ACTCAGTATACCTTTATAACAAATCTACACATGCACCCTCCGATTCGAAA

ATAGAAGTTGAAAAAAACTGTATTTTCTCTAAAAAATTTTGCTTATGTAC

TAGTTGGTGCCCTTCAAGTGGGAAAAATCCATGCCTGCTGAACTGTCACC

TCTTTTTTTTTTGGAGGGCTTCTATTTTTCAAGTGCCCTGTGTCTTCTTG

GGCTTGTTTTTTATTCTAGACAAAATGCTTCCACACACTGGGGGCTTTAC

CTGGCCTGCTCATGAAGGCATCATCTGGGTCCAAACTCCTTATTGGCTAC

TTATCAAAATGGATCTTTCTCTGCTCTTCTTTTGCATTAGATTCTTGCTA

AGAACCTAGAATTTGCCTGATAGATTGACTTACCCTAAAGTTAAAAAGGC

TTTTATTAGCTTATTTTATGGCTCTATATTGTGTATTTCCTTTTAAACAG

AGGTTCCTTAGAGACTCAATTCCTTAATCCAGCATTTCTTAAGTTTGCTT

TTGGGAAGGTTAGTACAAGAAGATGGTCTCTTTAAAAATAATTCTTGAGA

ATCACTATACTGGTTAACATATTAAAAGAACCAAGGAGTCCTACAGATTT

TTAAAAAGCCTTTGCTTATTGATACTCTTTGGTTTGACTGCCAATTCTCT

TTTTGTGGATCATCTGCTAATATCTAATAAATTTAGCATGACACAGAACA

AATGTTGAGAAACAGTCAATAAAAAACTGTCTTTGTGAAAATTTTGGCAT

TGCACTATCTGGAAAAAGTGATTTTTGTGAGTAGAGCCATGTGAGTATGC

TTCTAAAAACACAACATATTGGCATTATTCTCAGGAGTAGATCCAGGTTT

TGTGGCACTTGAAGATTATACAGTTGATGGGTGACTAAGAAAAAAATTAC

TGTGTTAATCAAAATTTAGGTTTTGAAAGGGGCCTGTGTAAGCAAAGAGC

CCACAGAGTAAGCTTCATTGTCTGTATTGTCAATCTACAAAAGACTGACC

TTTTTCAAGTGAGTATAAACCTGGCCTGGCTTATGTTCATGTGGTGAGTG

AAAGAATAGTAAACCAGGAAGAAGAAGGCTTATGTCCTTGGTCAGAATTG

TCCATTTTACTTCAATTCCCTAGACTCTGTGACTTTGGATAAGTCACTTT

ATTTCTCATCATCAGTGACTTTATTTTTAGAAAGGGGTATTAATTGTCCT

CTGGATGATAAAGAGTCATTTTGAAATTATAATTAATGGGGAAGCATTTA

GCACTATACAAATAGAGGTTATGATTCTTTTCAAAATGCTTATTAATTCT

GAGGACTACACATATGTTAACAATCATTATTGCTAACATATCAAATAAAA

ACAAATCTGGACTTAAGTAAATGGAGATTTTATTTTTAAAATATTGCAAT

AGAAAAGGTAGACAATTGCAATAGATTAATGACCCTAAGATCTCCATCTC

AAATGTCAGGCAGAGGACAGCTTTTTTTGTTCTTTATGGCAAGGAATAAA

CAAGGCTAGAAAGAACAGGTATGGGGCGTGGAAAGTGGGTAAGTGAGTGG

CATGATGAGACAGTTGATATCCCTTCCCCTGTCTCCCCATTTCAGCTGAT

TTTCAGGAGGGGACAGTAAGGAGAGGTTGCTCCAAGTTCCATTGCTCAGC

AGAGGATCAAAGATAAGAGACCTGGGGAGAGGTGAGACACTTGAGTATAG

TTTAAGCAAATCAAATTGGTAGGCATTTTGTCCAGGTTGGTGAGTGGAAA

GAAACAGTTAAGCTAATCATTTATGAGGCAAAGGGTGGGAATTTGGAGGA

TCTGTGTCCAGCCTTGTCAAAGGCAAAAAAGGAGTCATTCACAGGTCTTT

TTTAAGTTATGTGCAGAAGAGTAGTTCTTTGCAGTAAGCTATTCTTGGGG

TACATATTAGGATAAGGGAATGTCTTAACTTTTGCTGTTTTCCAGGAGCA

CAGGGCTCAGGTAAAGTTCAATATTATCAAATATCAACACCATCATTGCT

TTTCTTGTGTCTGGTCAGTCATTCAAAATGCTGTATCTTTGGAAAAATTA

CTTAACTTTTCAGACCTTCAGTTTTCTCATCAGCAAAATGAGAATAATTA

TAATAATAACTAGCTCACTAGGATACGGTGATTCTTGAATGACCTAATGA

CTATGCAAGCACCTCATAAGCTATAAATATTGTGGAAATATTATGCATTT

TTTTGTTGTCATTGTTAGTAGCTCTATAGGCTCTGACTTCTTCATTTATT

TGATGCAGCAAAAATGGGGAAGGGATGAAACATTGTTTAAATGAATCCTA

GATACCAGACACAGGGGTGCCCCATACTCTTTCTCTCACCAGGTGCATAC

AACCCTGAATTGTCATGTGCATGAAATCATCTACTTTCATCCAAGGAATA

TGGAAAAGAGATGGGCAAAGAGATAACTCAGCTACATTTGCCTGTTAATA

ATTGATTTATTTTACTC

>hg19_ct_ARAlincRNAs_9727_ARAlincRNA_0020.5 range=chr1:61039010-61062045 5'pad=0 3'pad=0 strand=- repeatMasking=none

GAATGTGCCATATGTGGAGACCTGCATGGAAAGGAACTGAGGATGCTCTC

TCACTAAAAGCTAGAAGGAACTGAGGCCCTCAGTCAACAGTCTACAAGAC

ACTGAATCTTGCCAGCAGCCAAGCAGTGAGCTTGAAAGCAGATCTATCAT

CAGCTGAACCTTCAGATGAGACCGCAGCCTAAGCAGATACCTTGGTTGCA

GCCATGTGAGACAACCTGAAACAGAGGGCACAGCTAAGCCTCAGCCAGGT

TCATGACCCTCTGAGGAAACTGTGAGAAAGCAAATGTCATTTTAAGCTAT

TTAATTTTGGAGGTAGTTTATTACACAACAATAAATAACTATTATAATTA

TCTTGAGGATTTATTTCCTGGGGAGAATACATGGAATTGCATATATAGGT

CCTCATATGCAGAATACTGGTACCCTAGAGATGTTCACCCCAGAGATGTT

TTAGGAACCTGTGAATATGTTACATTAAATGGCAAAGTTTTCCTGGATGG

TTTAAGTTTAAGATGGAAAAGTTTTACTGGATTATCCAGTGGGTCCAATC

TAGTAACATGAGTCCTTAAAAATGGAGAATATTTTTTGGCCATGATCAGA

GAGACATGTGATGATGGAAAAAGTGTCAGAGATGCAATGTGAGGGGACTA

AAACTGCTGTTGCTGACTTTAAAGATGGAGAGAGGGGAACCCGAGTCAAG

GAATGCAAATGGCCTCTGGAAGCTGGAAAAAGCAAGAAATGGATTCTCCT

TTAGAGCCTGTGGAATAAAATGCAGTCATGCTGACACCTTGATTTTACCT

CAGTGAGACCAATACTGGAGTATGACCTTCGGAGCTCTCTAATGATCAGT

TGGAATGAGGATGTTAATGGAACACTATGCAGCCATTAAAAAAAAATGAG

TTCATGTCCTTTGCAGAGACATGAATGGAGCTGGAGGCCATTATCTTCAG

CAAACTAATGCAAAACAGAAAACCAAATACCACATGTTCTCAATTATAAG

TAGGAGCTAAATGATGAGAACACATGGACATATAGAGGGAAACGACACAC

ACTGAGGCCTACCTGAGGATGGAGGGTGGGAGGAGGAAGAGGATAAGGAA

AAATAACTAATGAGTACTAGGCTTAATATCTCGGTAATGAAATAATCTGT

ACAACAAACCCCCATGATGCAAGTTTACCTATAAAACAAACCTGCACATG

TACCCCTGAACTTATAATAAAACTTCAAAACAAAACAAACAAAACAAAAT

AAAGCCTCTAATTAAAATGGAGAATGAGTAGCTATGGAGACAAAGGCTTT

TCTGTAGATACTAGAGACAAGAAGGAGGCCCTGTTGCTTCAAAATGCTGA

GGTTCCTTGAGAATGTCAGTGTAGCCTTCATAAAGCTCCGGATTTCTCTC

TTTTGGGGCCATGAGGGGTTATTTGCTAATCTGGAAGTTGGCATTTCGGG

TGAACTGGATTTTAACTCAGCTCACTCTTCATTTTTTTGATGGCCATGGA

GAAGGGATAGTGAGAGATTGAGAAGTGGAGGCTCTGAACTCCTTGTGGAG

CTGTGCTCACCTCACCCTCCACCTGGGTGAGCAGGAGTTGCAGATCCAAG

GGCTTTGGGCCACTAGACCTCTGGGTGGTGAGGACAGACTGTGCAGAGAA

GTCATCAGCCTAGGGATGGCCTTACCACAAACTCTCTAACCCTGTGCTTT

ACACACTCCTGACAGGTAGAAAGTAAATTTGCATAACAAACTGTTTAAAT

ATTCAATTTTTATCCAGCTTCAAGGCACTCTTTGTCCTCGGTAGTTCTAA

TGATTAGTGACCCCTTCAAAATCAGCATCATTTGTCAAATCAAATTAGCC

TCCTTACTAACTGCAAGGCAGTGACATTCATCTTTTTGATGCTTTCAAAC

CAGTCGGAGGTAAAGTTCTCAGATGGAATGGGGCTTGGTATGCTGCATGA

GTCACTTCCTCAATGAGGCAATTGTCAGAAAAATAGGAGACGTAGCTCTA

AGGTAAATATTGTTGGGTCATTTGAAATGTATGCAGATTTGACATTTCCC

TTCTGATTAGCATGTCTACACATTAAAGTTTTGTGTATGTCACATTGGAT

AGTTCAGATGCCAGGGTAATGTCAGTGAGCACTTGATAGAAACTTAATTT

TGCTCCTGATGAAGAAAATGGAAGGATGAAGAGGAAAACCTAAGGTGAGA

ACCTTATTTAGAGTGTACAGGGGGTATTCAGAATAAAAAAAAAAAATGCT

GGGAAGGAAAGTGTCATTATCTACTATGTGCCAGTTACTTTATTACCTAA

TTTAGTCCTTTTAGGGATATTGACCCCGTTTGAATAATTAAAATAGGTGA

ATCAACAGGCTTAATGCCAACAGATAGTAGAATAGTAGAGCTGAAAGTTC

AAGCTCATGTGTGTTTGATGCTGAAGTCCATGCTTCTTCCTGCAGAGTGT

TGCCTCCATATGATGCAAAATATCCAACCTAGCCCTGACATGAAAACTTA

ATTGAAAGTATCCAACAGTGG

>hg19_ct_ARAlincRNAs_9727_ARAlincRNA_0020.3 range=chr1:61039010-61105798 5'pad=0 3'pad=0 strand=- repeatMasking=none

TGAGATGAACCCAGTACCTCAGATGGAAATGCAGAAATCACCCGTCTTCT

GCGTCGCTCACGCTGGGAGCTGTAGACCGGAGCTGTTGCTATTCGGCCAT

CTTGGCTCCTCTCCTGCACTGCAAGAATGGACTAATACGCTATGTGAAGA

CAAAGGGGATGTCTGCAAGCCAGGACGAGTGCGTCTACCAGAAACTGAGT

TCTGCTGGACTTTGATCTAGGACATCCAGCCTCCAGAATGCTGGCTTTAA

AAGTTATCACATGTTTCTCTTTCCTTCTTGCACTTCTGCTATTGCTGTGA

GAAGAACTGCCCTAGGATTTTACAGGCACCCCAGCTTGAACTTCAATGAA

GCAAACTTGCACTCACTCCACAACAAGGATTCAAGCAAAGGGGAACCGGT

AACTTGAATCAGAGTTGTTTGGCTGTGTCCCAGTTGACCCACAGATGTGA

ATAAGAGAGAAGAATTTTCATTTCAAGCCACTATGTTTGTGGTGACTTGT

CATGCAGCAATAGCAAAATGATACAAATACTGTGATAGACTGCTTCTGAA

ATGGCTCCCAATGATCTTTCCTCATGATGGTCACTCCATAGTGTATTTTC

TTTTCTTTGTATGTGGGCTGGTCTTGTTGATGTAGTAACTTACTTTTAAC

TAATAGAATATGGCAAAAGTGAAGGAATGGCACTCTTGAGGTTAGATTTT

AAAAGACCATGACAACCACCTTCCTGACACCTTTTCTCTCTCTCTCTCTG

CTTACTCACTCTGATGAAGGAATGTGCCATATGTGGAGACCTGCATGGAA

AGGAACTGAGGATGCTCTCTCACTAAAAGCTAGAAGGAACTGAGGCCCTC

AGTCAACAGTCTACAAGACACTGAATCTTGCCAGCAGCCAAGCAGTGAGC

TTGAAAGCAGATCTATCATCAGCTGAACCTTCAGATGAGACCGCAGCCTA

AGCAGATACCTTGGTTGCAGCCATGTGAGACAACCTGAAACAGAGGGCAC

AGCTAAGCCTCAGCCAGGTTCATGACCCTCTGAGGAAACTGTGAGAAAGC

AAATGTCATTTTAAGCTATTTAATTTTGGAGGTAGTTTATTACACAACAA

TAAATAACTATTATAATTATCTTGAGGATTTATTTCCTGGGGAGAATACA

TGGAATTGCATATATAGGTCCTCATATGCAGAATACTGGTACCCTAGAGA

TGTTCACCCCAGAGATGTTTTAGGAACCTGTGAATATGTTACATTAAATG

GCAAAGTTTTCCTGGATGGTTTAAGTTTAAGATGGAAAAGTTTTACTGGA

TTATCCAGTGGGTCCAATCTAGTAACATGAGTCCTTAAAAATGGAGAATA

TTTTTTGGCCATGATCAGAGAGACATGTGATGATGGAAAAAGTGTCAGAG

ATGCAATGTGAGGGGACTAAAACTGCTGTTGCTGACTTTAAAGATGGAGA

GAGGGGAACCCGAGTCAAGGAATGCAAATGGCCTCTGGAAGCTGGAAAAA

GCAAGAAATGGATTCTCCTTTAGAGCCTGTGGAATAAAATGCAGTCATGC

TGACACCTTGATTTTACCTCAGTGAGACCAATACTGGAGTATGACCTTCG

GAGCTGAATAAAATTATACGTACACATCTTAATTTCTTGGGATTTGATTA

CTTCTTCTGCTTTGAAATTAGACCAAGTTTAGCCTGCCTTTGCTATTTTG

GCGTATCTCCAATTCTTCATGATTTTCTCATAGTGTTGCCTCCATATGAT

GCAAAATATCCAACCTAGCCCTGACATGAAAACTTAATTGAAAGTATCCA

ACAGTGG

>hg19_ct_ARAlincRNAs_9727_ARAlincRNA_0020.16 range=chr1:61039349-61040061 5'pad=0 3'pad=0 strand=- repeatMasking=none

GCTCACTCTTCATTTTTTTGATGGCCATGGAGAAGGGATAGTGAGAGATT

GAGAAGTGGAGGCTCTGAACTCCTTGTGGAGCTGTGCTCACCTCACCCTC

CACCTGGGTGAGCAGGAGTTGCAGATCCAAGGGCTTTGGGCCACTAGACC

TCTGGGTGGTGAGGACAGACTGTGCAGAGAAGTCATCAGCCTAGGGATGG

CCTTACCACAAACTCTCTAACCCTGTGCTTTACACACTCCTGACAGGTAG

AAAGTAAATTTGCATAACAAACTGTTTAAATATTCAATTTTTATCCAGCT

TCAAGGCACTCTTTGTCCTCGGTAGTTCTAATGATTAGTGACCCCTTCAA

AATCAGCATCATTTGTCAAATCAAATTAGCCTCCTTACTAACTGCAAGGC

AGTGACATTCATCTTTTTGATGCTTTCAAACCAGTCGGAGGTAAAGTTCT

CAGATGGAATGGGGCTTGGTATGCTGCATGAGTCACTTCCTCAATGAGGC

AATTGTCAGAAAAATAGGAGACGTAGCTCTAAGGTAAATATTGTTGGGTC

ATTTGAAATGTATGCAGATTTGACATTTCCCTTCTGATTAGCATGTCTAC

ACATTAAAGTTTTGTGTATGTCACATTGGATAGTTCAGATGCCAGGGTAA

TGTCAGTGAGCACTTGATAGAAACTTAATTTTGCTCCTGATGAAGAAAAT

GGAAGGATGAAGA

>hg19_ct_ARAlincRNAs_9727_ARAlincRNA_0020.15 range=chr1:61040313-61040550 5'pad=0 3'pad=0 strand=- repeatMasking=none

CACATGTTCTCAATTATAAGTAGGAGCTAAATGATGAGAACACATGGACA

TATAGAGGGAAACGACACACACTGAGGCCTACCTGAGGATGGAGGGTGGG

AGGAGGAAGAGGATAAGGAAAAATAACTAATGAGTACTAGGCTTAATATC

TCGGTAATGAAATAATCTGTACAACAAACCCCCATGATGCAAGTTTACCT

ATAAAACAAACCTGCACATGTACCCCTGAACTTATAAT

>hg19_ct_ARAlincRNAs_9727_ARAlincRNA_0020.13 range=chr1:61044986-61046129 5'pad=0 3'pad=0 strand=- repeatMasking=none

TAGATTCTTAGGGATTCTGAAAAACACCTTACAGACCATGTGAGAGGTAA

CGAGGAGGCTCTCCTGGACTGAACTGTGTCCCCTCCAAATTCATATGTTG

AGACCCAAACTTCCAATGTGACTGTATTTGGAGCTAGGGCCTTTAAGGAG

TAATTGACATTAAATGAGGTCACAGGGTTGGGACTCTAATTTAATAGGAC

TGGTGTCCTTATGAGAAGAGAAAGAGGCACCAGGCACCAGGCATGTGAGC

CCACAGAGGAAAGGCTATGATGTGAAGACAGAGAGAAGGCCATCACACAC

AAGCCATGGAGAGAGGCCTCCCCAGAAACCAACCCCACCGACACCTTGAT

CTCGGACTTCCAAGCTTCAGAACTGTGAGAAAATAAATTCCTATTGTTTA

AGCCACTCAGTCAGTGGAATTCTGTTATAGCAGCCCTAACAGACTAATAC

GGAAGTCAAGTAGACAAGTCTGGTCTCTGTATTTCTATTGGAACTGGATT

TACTCTGCTCCTATGAGTTATCTCTATGGGAGTTTGATATAAATTGTTAA

AAAATTGAAGCAGATAAACATGAAATTGTAGAGCAATTTAACCAGGTGTT

GTAATGGAAACATTTCTTCCTGATGCTGGCCCCATGTTGTGTTTATAAGT

GGAAAGTTTCTTCCTCTCCAAGTCCACAGGACTCCTTTCCTTTCTAAGTA

CTCTACGTGGCTGGCCACATCATTAAAATATGAGTTATTTTGATGGCAGA

GAGAAGATATCACTTATCTTTGTGACTTCTCCTTCCCCTGTCTCTTCCAC

CCCTTTCGGCCAATGGCATACTTAGTCATGTGTACTTAGTAATGTTAAAT

ATGTAAGTAAATTTGACCTATTGAAGAATGGCCTCTATAGCCTCCTAGAC

AACATATAAACCTATATTGTAAATCTTCTAGTAACAGAAAGCAGAATAGA

ATACAAGTTTTTTACAAATCTTTACAGATTTCTTCTAAGAGTTTGGTTTT

TCATAAGTAGTTCTTACCATTTTTGTCTCTATAAAAATTTCTGTGCCCTC

TTATTGCCTAGAATATTTTCTATTCAGAGATTTTACAAGGAATATTCACC

TGATATTGGCACAAGCACAAAGTTAGAATTAGGGAGCTATTACT

>hg19_ct_ARAlincRNAs_9727_ARAlincRNA_0020.14 range=chr1:61044986-61046191 5'pad=0 3'pad=0 strand=- repeatMasking=none

GAGAAAGTTATTACTAATGATATGGACTAGCAATTTTCATGTTTTTTTTT

TTTTTCCCCTAGTAGATTCTTAGGGATTCTGAAAAACACCTTACAGACCA

TGTGAGAGGTAACGAGGAGGCTCTCCTGGACTGAACTGTGTCCCCTCCAA

ATTCATATGTTGAGACCCAAACTTCCAATGTGACTGTATTTGGAGCTAGG

GCCTTTAAGGAGTAATTGACATTAAATGAGGTCACAGGGTTGGGACTCTA

ATTTAATAGGACTGGTGTCCTTATGAGAAGAGAAAGAGGCACCAGGCACC

AGGCATGTGAGCCCACAGAGGAAAGGCTATGATGTGAAGACAGAGAGAAG

GCCATCACACACAAGCCATGGAGAGAGGCCTCCCCAGAAACCAACCCCAC

CGACACCTTGATCTCGGACTTCCAAGCTTCAGAACTGTGAGAAAATAAAT

TCCTATTGTTTAAGCCACTCAGTCAGTGGAATTCTGTTATAGCAGCCCTA

ACAGACTAATACGGAAGTCAAGTAGACAAGTCTGGTCTCTGTATTTCTAT

TGGAACTGGATTTACTCTGCTCCTATGAGTTATCTCTATGGGAGTTTGAT

ATAAATTGTTAAAAAATTGAAGCAGATAAACATGAAATTGTAGAGCAATT

TAACCAGGTGTTGTAATGGAAACATTTCTTCCTGATGCTGGCCCCATGTT

GTGTTTATAAGTGGAAAGTTTCTTCCTCTCCAAGTCCACAGGACTCCTTT

CCTTTCTAAGTACTCTACGTGGCTGGCCACATCATTAAAATATGAGTTAT

TTTGATGGCAGAGAGAAGATATCACTTATCTTTGTGACTTCTCCTTCCCC

TGTCTCTTCCACCCCTTTCGGCCAATGGCATACTTAGTCATGTGTACTTA

GTAATGTTAAATATGTAAGTAAATTTGACCTATTGAAGAATGGCCTCTAT

AGCCTCCTAGACAACATATAAACCTATATTGTAAATCTTCTAGTAACAGA

AAGCAGAATAGAATACAAGTTTTTTACAAATCTTTACAGATTTCTTCTAA

GAGTTTGGTTTTTCATAAGTAGTTCTTACCATTTTTGTCTCTATAAAAAT

TTCTGTGCCCTCTTATTGCCTAGAATATTTTCTATTCAGAGATTTTACAA

GGAATATTCACCTGATATTGGCACAAGCACAAAGTTAGAATTAGGGAGCT

ATTACT

>hg19_ct_ARAlincRNAs_9727_ARAlincRNA_0020.12 range=chr1:61044986-61062565 5'pad=0 3'pad=0 strand=- repeatMasking=none

AAAGTTATCACATGTTTCTCTTTCCTTCTTGCACTTCTGCTATTGCTGTG

AGAAGAACTGCCCTAGGATTTTACAGGCACCCCAGCTTGAACTTCAATGA

AGCAAACTTGCACTCACTCCACAACAAGGATTCAAGCAAAGGGGAACCGG

TAACTTGAATCAGAGTTGTTTGGCTGTGTCCCAGTTGACCCACAGATGTG

AATAAGAGAGAAGAATTTTCATTTCAAGCCACTATGTTTGTGGTGACTTG

TCATGCAGCAATAGCAAAATGATACAAATACTGTGATAGACTGCTTCTGA

AATGGCTCCCAATGATCTTTCCTCATGATGGTCACTCCATAGTGTATTTT

CTTTTCTTTGTATGTGGGCTGGTCTTGTTGATGTAGTAACTTACTTTTAA

CTAATAGAATATGGCAAAAGTGAAGGAATGGCACTCTTGAGGTTAGATTT

TAAAAGACCATGACAACCACCTTCCTGACACCTTTTCTCTCTCTCTCTCT

GCTTACTCACTCTGATGAAGGAATGTGCCATATGTGGAGACCTGCATGGA

AAGGAACTGAGGATGCTCTCTCACTAAAAGCTAGAAGGAACTGAGGCCCT

CAGTCAACAGTCTACAAGACACTGAATCTTGCCAGCAGCCAAGCAGTGAG

CTTGAAAGCAGATCTATCATCAGCTGAACCTTCAGATGAGACCGCAGCCT

AAGCAGATACCTTGGTTGCAGCCATGTGAGACAACCTGAAACAGAGGGCA

CAGCTAAGCCTCAGCCAGGTTCATGACCCTCTGAGGAAACTGTGAGAAAG

CAAATGTCATTTTAAGCTATTTAATTTTGGAGGTAGTTTATTACACAACA

ATAAATAACTATTATAATTATCTTGAGGATTTATTTCCTGGGGAGAATAC

ATGGAATTGCATATATAGGTCCTCATATGCAGAATACTGGTACCCTAGAG

ATGTTCACCCCAGAGATGTTTTAGGAACCTGTGAATATGTTACATTAAAT

GGCAAAGTTTTCCTGGATGGTTTAAGTTTAAGATGGAAAAGTTTTACTGG

ATTATCCAGTGGGTCCAATCTAGTAACATGAGTCCTTAAAAATGGAGAAT

ATTTTTTGGCCATGATCAGAGAGACATGTGATGATGGAAAAAGTGTCAGA

GATGCAATGTGAGGGGACTAAAACTGCTGTTGCTGACTTTAAAGATGGAG

AGAGGGGAACCCGAGTCAAGGAATGCAAATGGCCTCTGGAAGCTGGAAAA

AGCAAGAAATGGATTCTCCTTTAGAGCCTGTGGAATAAAATGCAGTCATG

CTGACACCTTGATTTTACCTCAGTGAGACCAATACTGGAGTATGACCTTC

GGAGCTGAATAAAATTATACGTACACATCTTAATTTCTTGGGATTTGATT

ACTTCTTCTGCTTTGAAATTAGACCAAGTTTAGCCTGCCTTTGCTATTTT

GGCGTATCTCCAATTCTTCATGATTTTCTCATATTCTTAGGGATTCTGAA

AAACACCTTACAGACCATGTGAGAGGTAACGAGGAGGCTCTCCTGGACTG

AACTGTGTCCCCTCCAAATTCATATGTTGAGACCCAAACTTCCAATGTGA

CTGTATTTGGAGCTAGGGCCTTTAAGGAGTAATTGACATTAAATGAGGTC

ACAGGGTTGGGACTCTAATTTAATAGGACTGGTGTCCTTATGAGAAGAGA

AAGAGGCACCAGGCACCAGGCATGTGAGCCCACAGAGGAAAGGCTATGAT

GTGAAGACAGAGAGAAGGCCATCACACACAAGCCATGGAGAGAGGCCTCC

CCAGAAACCAACCCCACCGACACCTTGATCTCGGACTTCCAAGCTTCAGA

ACTGTGAGAAAATAAATTCCTATTGTTTAAGCCACTCAGTCAGTGGAATT

CTGTTATAGCAGCCCTAACAGACTAATACGGAAGTCAAGTAGACAAGTCT

GGTCTCTGTATTTCTATTGGAACTGGATTTACTCTGCTCCTATGAGTTAT

CTCTATGGGAGTTTGATATAAATTGTTAAAAAATTGAAGCAGATAAACAT

GAAATTGTAGAGCAATTTAACCAGGTGTTGTAATGGAAACATTTCTTCCT

GATGCTGGCCCCATGTTGTGTTTATAAGTGGAAAGTTTCTTCCTCTCCAA

GTCCACAGGACTCCTTTCCTTTCTAAGTACTCTACGTGGCTGGCCACATC

ATTAAAATATGAGTTATTTTGATGGCAGAGAGAAGATATCACTTATCTTT

GTGACTTCTCCTTCCCCTGTCTCTTCCACCCCTTTCGGCCAATGGCATAC

TTAGTCATGTGTACTTAGTAATGTTAAATATGTAAGTAAATTTGACCTAT

TGAAGAATGGCCTCTATAGCCTCCTAGACAACATATAAACCTATATTGTA

AATCTTCTAGTAACAGAAAGCAGAATAGAATACAAGTTTTTTACAAATCT

TTACAGATTTCTTCTAAGAGTTTGGTTTTTCATAAGTAGTTCTTACCATT

TTTGTCTCTATAAAAATTTCTGTGCCCTCTTATTGCCTAGAATATTTTCT

ATTCAGAGATTTTACAAGGAATATTCACCTGATATTGGCACAAGCACAAA

GTTAGAATTAGGGAGCTATTACT

>hg19_ct_ARAlincRNAs_9727_ARAlincRNA_0020.11 range=chr1:61053709-61068751 5'pad=0 3'pad=0 strand=- repeatMasking=none

CTAGGTTAAAGCACCAGCAGATTTGGTGTCTGATGAGGGCCTGCTACCTG

GTTCACAGACAGCCATATTCTTGCTTTGTCCTCAACATAGCTGAGGGTCC

CAGGGAGCTCCCTCGGGTCGCTTTTATTAGAGCACTCATCCTATTTATGA

GGGCAGAGCCCTCCTAAAGCTCTCATTTCTAGTACCATCATGATGGTGAT

TAAATTTTAATTTAAGAACTTTGGGGGAACAAAAACATTCAGTCTATAGC

ATAGATAAGATCAAAGATGGCTTCCCTTTTCTATAAGGTAACCATTTATA

CAAGAAGACTTTGATTTCCTCTACTGTTTTTGGATCTTTATTAAACTCTG

GAATAATTATACTTGGAAGAGCAAATGTAAGACTACAGGGCTTTCTTATA

CAGTTATGCATATAAGATTTGCATATGTAATTCAGAGCTTTCTTATATGG

TTGTGGAAATTGTGCACTGGACAACTATAGGGAGAGTGGTTAACATGGTA

TACCATATGAATCTTGCTCCCTAGGGTTGAGCATTGCATATCCTGTGTGA

ATTCATGTGGTTGGCCTGCACAAATCTCATAGCTAAGTCTATGCAGCCTC

ATCTCTGATCCTTTTTTCTGATAAGCCAGCATCACAGCACAAGACTTCTG

CTGGGAAAAGAGGGAATGGATGAGTTTGCAATCAATTTTGAGATTAAAGT

TTAAGTTGTACATTTAGTAATGAAAAATGACTAGAAATGATAAAATCTAC

CTAAGATATTGCAAAGAAATTGGAAAGGATGATTTGTCAGAATGTGGCTA

AAGGTGTTAACGGCAAAAAAAGAAAATATATTCGTGTTTTTACCACATCA

TATTGAGAATCCTCAATAATATGGTTATATAAAATTGAGAGAAAAACATG

TCTTTTGACTCAGAGAAATCTTCATTCTTCCTAAGACAGACAGACATATG

ATAATGAATTAAGTACTACTGGAGAGAAGGGTTCCAGTAGGTGCAAGGAG

GGGGTAGGGATTAGTCCTGAGCGGGCAGCAGAGGAGACTTCTTTGAAAAG

CTGACATTTTATTTTTTATTTTTTCAAAAAAATTTTTTTAAATTTTAGAT

TCAGATGGTACATGTGCAGGTTTGTTACATAAATATATTGCATCATGCTG

GGATTTGGGCTTCTATGGAACCCACCAACCAAATAGTAAACACAGTACAC

AATAAATAGCTTTTCAACCATTTCTATTCTCCCTTCTTCTCCCCTTTTGG

GGTCCCCAGTGTCTATTGTCTCCATCTTTATGTCTGTATGTACCCATTGT

TTAGTTTCCACTTATACGTCAGAAGATGTGGTATTTGATTTTCTGCTTTT

GTGTTAGTTCATTTAGGATAACTACAGCTGCATCCATATTGCTGTAAAGT

ACATGATTTCATTCTTTTTATGGCTGAATAGTATTCCATGGTGTACATGT

ACAAAATTTACTTAATCTAATCCATCATTGATGGGCATCTAGGTTGATTC

CATATTTTTCTTGTTGTGAATAGTGCTGTGCTGCGGTGAACAACATATGA

GTGCATGTGTCTTTTTGGCAGAATGATTTATTTTCTGTAGGATATATACC

CAATAATATGATTGCTGGGTTGAATGATAGTTTTATTTATAGTTCTTTGA

GAAATTTCCAAAGTGGCTGAACTAATTTACATACCCACCAACAGTGTATA

AGCATTCCCTTTACTTTGCATCCTCACCAACAGCTTTTATTTTTGACTTT

TTAATAATAGCTATTCAGACTGGTGAGAGATGGTATTCCACTGTGGTTTT

GATTTGGATTTCCCTAATGATTAGTGATGCTGAGCATTTTTTCATATGTT

TGTTGGCTAAAAAGCTGATATTTCATTTCATTTTTTTAAAGGTTTATGTT

AGGTTCGGGGGTACATGTGAAGGTTTGTTACATAGGTGAACTTGTGTCAC

AGGGGTTTGTTGTACAGCTGATTTCATCACCCATGCAAAAAGCCCAGTAC

CCAATAATTATTATTTGTGCTTCTCTTCTTCCTCCCACCCTCCACCCTCA

AGTAGACCCCAGTGTCTTTCATTCCCCAAAAGCTAACATTTTAAACCAGC

CCAAAATATAATAATTTTAATCTGAAGTGGCATGTGATTTCTGTTAATGA

TAAAAAAGATAACATTTGAAAAGGAGTCCTTTTGATAACCCATGTATTAA

GGACTAAATGTTTGTACCTTCTCCCACCCCCAGAATCATATGTGAAACCC

TAACAACCAATGTGAATGTATTTGGAGATAGGGTCATAAGCAGATGGTAG

AGTTTAAATGAGGTCATAAGGGTGGAGCCCTAATCTGATAGAGCTGTTGC

ACTTTTCAGAAGAGGAAGAGATACTGGAACGTGTCTCTGTCTGTGTGCAT

TCAGAAAGAAGGCCATGTGATACAGTTTGGATGTTGCCTCCAACCAAATG

TCATGTCAAAATGTAATCTTCAGTATTGGAGGGGAGGCATGGTGGGGGGA

GATTGGGTCCCCCCACCCAGTGGAGGCAAGTTTCTCATGAATGGTTTAGC

ACCATCCCCCTTGGTACTGTCTTCACAATAGTGAGCTCTCATGATATTTG

GTCATTTAAAAGTGTGTAGCACTTCCCCCTTCACGTTCTCTTGCTCCTGC

TCTGGCCATGTGACATGCCTATTCCCTCTTCACCTTCTGGCATGACTGTT

AAGTTTCCTGTGGCCTCCCTGGAAGCTGAGCAGATTCCATCATCATGCTT

CCTGTACAGTCTGCAGAACCATGAGCCAATTAAGCCTCTTTTCTTTATAA

ATTACCCAGTCTGAGGTACTTCTTTATAGCACTGCAAGAATGGACTAATA

CGCTATGTGAAGACAAAGGGGATGTCTGCAAGCCAGGACGAGTGCGTCTA

CCAGAAACTGAGTTCTGCTGGACTTTGATCTAGGACATCCAGCCTCCAGA

ATGGAATAAAATTATACGTACACATCTTAATTTCTTGGGATTTGATTACT

TCTTCTGCTTTGAAATTAGACCAAGTTTAGCCTGCCTTTGCTATTTTGGC

GTATCTCCAATTCTTCATGATTTTCTCAT

>hg19_ct_ARAlincRNAs_9727_ARAlincRNA_0020.10 range=chr1:61061114-61088212 5'pad=0 3'pad=0 strand=- repeatMasking=none

GAAAAGTCAGTAGAGAAGGTAGGCAGTGCTTGCAAAGGCCTGAGCACTGC

AAGAATGGACTAATACGCTATGTGAAGACAAAGGGGATGTCTGCAAGCCA

GGACGAGTGCGTCTACCAGAAACTGAGTTCTGCTGGACTTTGATCTAGGA

CATCCAGCCTCCAGAATGGAAGCAAACTTGCACTCACTCCACAACAAGGA

TTCAAGCAAAGGGGAACCGGTAACTTGAATCAGAGTTGTTTGGCTGTGTC

CCAGTTGACCCACAGATGTGAATAAGAGAGAAGAATTTTCATTTCAAGCC

ACTATGTTTGTGGTGACTTGTCATGCAGCAATAGCAAAATGATACAAATA

CTGTGATAGACTGCTTCTGAAATGGCTCCCAATGATCTTTCCTCATGATG

GTCACTCCATAGTGTATTTTCTTTTCTTTGTATGTGGGCTGGTCTTGTTG

ATGTAGTAACTTACTTTTAACTAATAGAATATGGCAAAAGTGAAGGAATG

GCACTCTTGAGGTTAGATTTTAAAAGACCATGACAACCACCTTCCTGACA

CCTTTTCTCTCTCTCTCTCTGCTTACTCACTCTGATGAAGGAATGTGCCA

TATGTGGAGACCTGCATGGAAAGGAACTGAGGATGCTCTCTCACTAAAAG

CTAGAAGGAACTGAGGCCCTCAGTCAACAGTCTACAAGACACTGAATCTT

GCCAGCAGCCAAGCAGTGAGCTTGAAAGCAGATCTATCATCAGCTGAACC

TTCAGATGAGACCGCAGCCTAAGCAGATACCTTGGTTGCAGCCATGTGAG

ACAACCTGAAACAGAGGGCACAGCTAAGCCTCAGCCAGGTTCATGACCCT

CTGAGGAAACTGTGAGAAAGCAAATGTCATTTTAAGCTATTTAATTTTGG

AGGTAGTTTATTACACAACAATAAATAACTATTATAATTATCTTGAGGAT

TTATTTCCTGGGGAGAATACATGGAATTGCATATATAGGTCCTCATATGC

AGAATACTGGTACCCTAGAGATGTTCACCCCAGAGATGTTTTAGGAACCT

GTGAATATGTTACATTAAATGGCAAAGTTTTCCTGGATGGTTTAAGTTTA

AGATGGAAAAGTTTTACTGGATTATCCAGTGGGTCCAATCTAGTAACATG

AGTCCTTAAAAATGGAGAATATTTTTTGGCCATGATCAGAGAGACATGTG

ATGATGGAAAAAGTGTCAGAGATGCAATGTGAGGGGACTAAAACTGCTGT

TGCTGACTTTAAAGATGGAGAGAGGGGAACCCGAGTCAAGGAATGCAAAT

GGCCTCTGGAAGCTGGAAAAAGCAAGAAATGGATTCTCCTTTAGAGCCTG

TGGAATAAAATGCAGTCATGCTGACACCTTGATTTTACCTCAGTGAGACC

AATACTGGAGTATGACCTTCGGAGCTGTAAGTTAATAAATTTCTGTTGTC

TTATGCACTAGTTTATGGTAATTTGTTATAGCAACAATGGAAAATGGATA

CATAGCATAGCCTATTAGGACT

>hg19_ct_ARAlincRNAs_9727_ARAlincRNA_0020.17 range=chr1:61061210-61063558 5'pad=0 3'pad=0 strand=- repeatMasking=none

AAAAGGGGTCAAGCTGAAGGGCAGGGGATGGGTGGCGGGGATAAAGTTCT

GGTCAGGTAAGTAATGGTCAAATCATGATGGCTCCTGTGTGCCATATAAA

GAAGTCTGGATTTAACCCTGAAGATAATGAAATCTGGATTTAACCCTGAA

GAAATGAAATTCTGAGAAGCGAAGAATCACAGTCTGGTTAAATTCTAATG

AAATTTTTAAGTCCCTTGTCGGCCTTCAGGATTCAGCCAAATGTTCAGAG

TGTTGTTACACTCTTAGACTTCATCTGACCTATATGCTGTCTTTACTTTG

CAGAAAGTAGGAGAGCAGTTGCTGAGTCAGCACCGCAGGGAGACAAGTGT

GTAAGGAACATGGATTATTCATGGGCCCTGTGCACTGTTCCAGGTGCAGC

CATGATTGCCGTGGACTGGGCCCATTTGTAATGCAAAGGCATGGCAAGAA

AATCCATTTTCTCATTTTCAGAGACGATTTTATCTCCACGCTCCTGCCCA

TGTTACAAATTTAGGCTGATAAAGTGAAACAGGAGGCAGAAGAGCAAAGT

GAAATTTCTGATGACTGTTTGCAAAAGGAATTTTTAGATACCAATTTGTG

AACTCCTTTATTCAGAAGGAAATGATTCAGAGAGAATGTATCTTGAGTCG

TGGTGGTATAAAACAATGAAGAACGGGTTTTGAAGTCAGACAAATGTGAA

TTTGAATTCTGTGACCTTGAGCAAGTCAGGTAGCTTATCTGCACCACAAT

TTCTTTATCAGTAAAATGTGAGTGATAATATTGTGAGAGATGGAATTTTT

GGCCCCAATTTTTCACCTCTTCCTGGATCCTTGACCTTTACCTTGTGACT

GTGAAGTTGCTTCCACTAGAGGCAGAATATACTTTCTAGTCCTTTTATTT

TGGGTTCAGGCATGTGACTTATTTTGGCCAGTAGGGTACCTGGGGGTAGG

GAGGAGGGAAGTAACCATGCAATAGGCCTCAATCCTGGCTTTAAAAGTTA

TCACATGTTTCTCTTTCCTTCTTGCACTTCTGCTATTGCTGTGAGAAGAA

CTGCCCTAGGATTTTACAGGCACCCCAGCTTGAACTTCAATGAAGCAAAC

TTGCACTCACTCCACAACAAGGATTCAAGCAAAGGGGAACCGGTAACTTG

AATCAGAGTTGTTTGGCTGTGTCCCAGTTGACCCACAGATGTGAATAAGA

GAGAAGAATTTTCATTTCAAGCCACTATGTTTGTGGTGACTTGTCATGCA

GCAATAGCAAAATGATACAAATACTGTGATAGACTGCTTCTGAAATGGCT

CCCAATGATCTTTCCTCATGATGGTCACTCCATAGTGTATTTTCTTTTCT

TTGTATGTGGGCTGGTCTTGTTGATGTAGTAACTTACTTTTAACTAATAG

AATATGGCAAAAGTGAAGGAATGGCACTCTTGAGGTTAGATTTTAAAAGA

CCATGACAACCACCTTCCTGACACCTTTTCTCTCTCTCTCTCTGCTTACT

CACTCTGATGAAGGAATGTGCCATATGTGGAGACCTGCATGGAAAGGAAC

TGAGGATGCTCTCTCACTAAAAGCTAGAAGGAACTGAGGCCCTCAGTCAA

CAGTCTACAAGACACTGAATCTTGCCAGCAGCCAAGCAGTGAGCTTGAAA

GCAGATCTATCATCAGCTGAACCTTCAGATGAGACCGCAGCCTAAGCAGA

TACCTTGGTTGCAGCCATGTGAGACAACCTGAAACAGAGGGCACAGCTAA

GCCTCAGCCAGGTTCATGACCCTCTGAGGAAACTGTGAGAAAGCAAATGT

CATTTTAAGCTATTTAATTTTGGAGGTAGTTTATTACACAACAATAAATA

ACTATTATAATTATCTTGAGGATTTATTTCCTGGGGAGAATACATGGAAT

TGCATATATAGGTCCTCATATGCAGAATACTGGTACCCTAGAGATGTTCA

CCCCAGAGATGTTTTAGGAACCTGTGAATATGTTACATTAAATGGCAAAG

TTTTCCTGGATGGTTTAAGTTTAAGATGGAAAAGTTTTACTGGATTATCC

AGTGGGTCCAATCTAGTAACATGAGTCCTTAAAAATGGAGAATATTTTTT

GGCCATGATCAGAGAGACATGTGATGATGGAAAAAGTGTCAGAGATGCAA

TGTGAGGGGACTAAAACTGCTGTTGCTGACTTTAAAGATGGAGAGAGGGG

AACCCGAGTCAAGGAATGCAAATGGCCTCTGGAAGCTGGAAAAAGCAAGA

AATGGATTCTCCTTTAGAGCCTGTGGAATAAAATGCAGTCATGCTGACAC

CTTGATTTTACCTCAGTGAGACCAATACTGGAGTATGACCTTCGGAGCT

>hg19_ct_ARAlincRNAs_9727_ARAlincRNA_0020.9 range=chr1:61063029-61063558 5'pad=0 3'pad=0 strand=- repeatMasking=none

AAAAGGGGTCAAGCTGAAGGGCAGGGGATGGGTGGCGGGGATAAAGTTCT

GGTCAGGTAAGTAATGGTCAAATCATGATGGCTCCTGTGTGCCATATAAA

GAAGTCTGGATTTAACCCTGAAGATAATGAAATCTGGATTTAACCCTGAA

GAAATGAAATTCTGAGAAGCGAAGAATCACAGTCTGGTTAAATTCTAATG

AAATTTTTAAGTCCCTTGTCGGCCTTCAGGATTCAGCCAAATGTTCAGAG

TGTTGTTACACTCTTAGACTTCATCTGACCTATATGCTGTCTTTACTTTG

CAGAAAGTAGGAGAGCAGTTGCTGAGTCAGCACCGCAGGGAGACAAGTGT

GTAAGGAACATGGATTATTCATGGGCCCTGTGCACTGTTCCAGGTGCAGC

CATGATTGCCGTGGACTGGGCCCATTTGTAATGCAAAGGCATGGCAAGAA

AATCCATTTTCTCATTTTCAGAGACGATTTTATCTCCACGCTCCTGCCCA

TGTTACAAATTTAGGCTGATAAAGTGAAAC

>hg19_ct_ARAlincRNAs_9727_ARAlincRNA_0020.8 range=chr1:61065986-61068312 5'pad=0 3'pad=0 strand=- repeatMasking=none

TTCTTATATGGTTGTGGAAATTGTGCACTGGACAACTATAGGGAGAGTGG

TTAACATGGTATACCATATGAATCTTGCTCCCTAGGGTTGAGCATTGCAT

ATCCTGTGTGAATTCATGTGGTTGGCCTGCACAAATCTCATAGCTAAGTC

TATGCAGCCTCATCTCTGATCCTTTTTTCTGATAAGCCAGCATCACAGCA

CAAGACTTCTGCTGGGAAAAGAGGGAATGGATGAGTTTGCAATCAATTTT

GAGATTAAAGTTTAAGTTGTACATTTAGTAATGAAAAATGACTAGAAATG

ATAAAATCTACCTAAGATATTGCAAAGAAATTGGAAAGGATGATTTGTCA

GAATGTGGCTAAAGGTGTTAACGGCAAAAAAAGAAAATATATTCGTGTTT

TTACCACATCATATTGAGAATCCTCAATAATATGGTTATATAAAATTGAG

AGAAAAACATGTCTTTTGACTCAGAGAAATCTTCATTCTTCCTAAGACAG

ACAGACATATGATAATGAATTAAGTACTACTGGAGAGAAGGGTTCCAGTA

GGTGCAAGGAGGGGGTAGGGATTAGTCCTGAGCGGGCAGCAGAGGAGACT

TCTTTGAAAAGCTGACATTTTATTTTTTATTTTTTCAAAAAAATTTTTTT

AAATTTTAGATTCAGATGGTACATGTGCAGGTTTGTTACATAAATATATT

GCATCATGCTGGGATTTGGGCTTCTATGGAACCCACCAACCAAATAGTAA

ACACAGTACACAATAAATAGCTTTTCAACCATTTCTATTCTCCCTTCTTC

TCCCCTTTTGGGGTCCCCAGTGTCTATTGTCTCCATCTTTATGTCTGTAT

GTACCCATTGTTTAGTTTCCACTTATACGTCAGAAGATGTGGTATTTGAT

TTTCTGCTTTTGTGTTAGTTCATTTAGGATAACTACAGCTGCATCCATAT

TGCTGTAAAGTACATGATTTCATTCTTTTTATGGCTGAATAGTATTCCAT

GGTGTACATGTACAAAATTTACTTAATCTAATCCATCATTGATGGGCATC

TAGGTTGATTCCATATTTTTCTTGTTGTGAATAGTGCTGTGCTGCGGTGA

ACAACATATGAGTGCATGTGTCTTTTTGGCAGAATGATTTATTTTCTGTA

GGATATATACCCAATAATATGATTGCTGGGTTGAATGATAGTTTTATTTA

TAGTTCTTTGAGAAATTTCCAAAGTGGCTGAACTAATTTACATACCCACC

AACAGTGTATAAGCATTCCCTTTACTTTGCATCCTCACCAACAGCTTTTA

TTTTTGACTTTTTAATAATAGCTATTCAGACTGGTGAGAGATGGTATTCC

ACTGTGGTTTTGATTTGGATTTCCCTAATGATTAGTGATGCTGAGCATTT

TTTCATATGTTTGTTGGCTAAAAAGCTGATATTTCATTTCATTTTTTTAA

AGGTTTATGTTAGGTTCGGGGGTACATGTGAAGGTTTGTTACATAGGTGA

ACTTGTGTCACAGGGGTTTGTTGTACAGCTGATTTCATCACCCATGCAAA

AAGCCCAGTACCCAATAATTATTATTTGTGCTTCTCTTCTTCCTCCCACC

CTCCACCCTCAAGTAGACCCCAGTGTCTTTCATTCCCCAAAAGCTAACAT

TTTAAACCAGCCCAAAATATAATAATTTTAATCTGAAGTGGCATGTGATT

TCTGTTAATGATAAAAAAGATAACATTTGAAAAGGAGTCCTTTTGATAAC

CCATGTATTAAGGACTAAATGTTTGTACCTTCTCCCACCCCCAGAATCAT

ATGTGAAACCCTAACAACCAATGTGAATGTATTTGGAGATAGGGTCATAA

GCAGATGGTAGAGTTTAAATGAGGTCATAAGGGTGGAGCCCTAATCTGAT

AGAGCTGTTGCACTTTTCAGAAGAGGAAGAGATACTGGAACGTGTCTCTG

TCTGTGTGCATTCAGAAAGAAGGCCATGTGATACAGTTTGGATGTTGCCT

CCAACCAAATGTCATGTCAAAATGTAATCTTCAGTATTGGAGGGGAGGCA

TGGTGGGGGGAGATTGGGTCCCCCCACCCAGTGGAGGCAAGTTTCTCATG

AATGGTTTAGCACCATCCCCCTTGGTACTGTCTTCACAATAGTGAGCTCT

CATGATATTTGGTCATTTAAAAGTGTGTAGCACTTCCCCCTTCACGTTCT

CTTGCTCCTGCTCTGGCCATGTGACATGCCTATTCCCTCTTCACCTTCTG

GCATGACTGTTAAGTTTCCTGTGGCCTCCCTGGAAGCTGAGCAGATTCCA

TCATCATGCTTCCTGTACAGTCTGCAG

>hg19_ct_ARAlincRNAs_9727_ARAlincRNA_0020.7 range=chr1:61105456-61105798 5'pad=0 3'pad=0 strand=- repeatMasking=none

TGAGATGAACCCAGTACCTCAGATGGAAATGCAGAAATCACCCGTCTTCT

GCGTCGCTCACGCTGGGAGCTGTAGACCGGAGCTGTTGCTATTCGGCCAT

CTTGGCTCCTCTCCTGGTAATCCTCTTATACATTTTTAAAAATTCTTAGT

TCATTAGTTTTTAATCTTCCTTATTTTTAAAATAAATGTATTTATAATTT

TTATTTTAAATATTGCTATAGCTGTAACTCACAAATTTTGTCATTTGGCT

TTTTATTGTCTTTCAGATCTAAATTTTACTAATTTCCTTTCTGACTTAAT

TTTTACCTGTGATTTAGTAGTATTATTTTAATTATTGGACATA

>hg19_ct_ARAlincRNAs_9727_ARAlincRNA_0020.1 range=chr1:61105944-61106163 5'pad=0 3'pad=0 strand=- repeatMasking=none

CTGCTTTGTTTACCCAAGCAAGCCTGGGCAATGGCGGGCGCCCCTCCCCC

AGCCTTGCTGCGGCCTTGCAGTTTGATCTCAGACTGCTGTGCTAGCAATC

AGCGAGACTCCGTGGGCGTAGGACCCTCCTAGCCAGGTGCAAGATATAAT

CTCGCGGTGCGCCGTTTTTTAAGCCGGTCCGAAAGCGCAATATTCGGGTG

GGAGAGACCCGATTTTCCAG

>hg19_ct_ARAlincRNAs_9727_ARAlincRNA_0021.3 range=chr1:73214604-73364823 5'pad=0 3'pad=0 strand=- repeatMasking=none

CTCCGTGGGCGTAGGACCCTCCAAGCCAGGTGCGATATAATGTCGTGGTG

CGCCGTTTTTTAAGCTGGTTGGAAAAGCGCAGTATTCGCGTGGGAGTGAC

CCGATTTTCCAGATAGTAATGAAGATAGGGGCCACCATTCCCTGTGAAAA

TCAAGTGGGAAAGTCCTGAGTCCTTTAATGACTTTACAGAGGACAGAATA

AACTCCTATCATATTCATGC

>hg19_ct_ARAlincRNAs_9727_ARAlincRNA_0021.2 range=chr1:73214849-73364566 5'pad=0 3'pad=0 strand=- repeatMasking=none

TGAGATGAACCCGGTACCTCAGATTGAAATGCAGAAATCACCCGTCTTCT

GCGTCGCTCACGCTGGGAGCTGTAGACCGGAGCTGTTCCTATTCGGCCAT

CTTGGCTCCTCCCTCAGTTCTGGAGACTGGGACATCCAAGGTCAATGTGC

TGGCAGATTCAATGTCTGGAGTGAAATACATCACCGCTTTCCCTGCTCCC

GCCGCCATTAAAATTGTGTTTGGCATCTGACCACCAAGATACAGATATTT

CCTAGACTGTTTTGCAGCTAGGTACTTAG

>hg19_ct_ARAlincRNAs_9727_ARAlincRNA_0021.1 range=chr1:73316159-73364920 5'pad=0 3'pad=0 strand=- repeatMasking=none

TACCTTAGCAAGCCTGGGCAATGGCGGGCGCCCCTCCCCCAGCCTCGCTG

CCGCCTTGCAGTTTGATCTCAGACTGCTGTGCTAGCAATCAGCAAGACTC

CGTGGGCGTAGGACCCTCCAAGCCAGGTGCGATATAATGTCGTGGTGCGC

CGTTTTTTAAGCTGGTTGGAAAAGCGCAGTATTCGCGTGGGAGTGACCCG

ATTTTCCAGTGAGATGAACCCGGTACCTCAGATTGAAATGCAGAAATCAC

CCGTCTTCTGCGTCGCTCACGCTGGGAGCTGTAGACCGGAGCTGTTCCTA

TTCGGCCATCTTGGCTCCTCCCTCAGGTATAGAGTTTTCTTAATGAAAAT

ATTTTGATTGTAGATTCAAAGTTGGTTCTTTGGAACAAAGACTAATAAAA

TTGATTAATTGCTGGCAAGACCCCAGTGGTCTCTTTTTATTAGTTTTGCT

TCTTTTATTACTCATTTATATTGCCTTGACTCTTCATGTGACTGCATTTT

TGTTGTTTGTTTGCTTATTTGCTAGTCATTATAAATGCAAAATCATGTTA

AAAAATAGTTCGTCAGCTAAGATTATGTTGTCTTTCTCAAAAACTGGTTT

GTTTGTGATGCTTATTGGTATTTTCTGGAGACTGGGACATCCAAGGTCAA

TGTGCTGGCAGATTCAATGTCTGGTAAGTTCCCATTTTTCTGGATCATAG

ATGGCTGCTTTTTCTCAGTGTCTTCACAAGGTGGAAAAGATGAACAGACT

CTTTTTGGCCTCTCGCTCTTATAAGGGCACCGATTGGATTCGTGAGGGCT

CTGCCCTCATGACATAATCACCTCTGAAAAGTCCCACCTCATAATAACAT

TACCTTGAAGGTTACATTTCAAAATAAGAATTTTGGGGTAACAGAAGCAT

TCAAATCATAGCAGAACCTAACTCAGCCTGGGGCATGCTTGGGAAAA

>hg19_ct_ARAlincRNAs_9727_ARAlincRNA_0022.1 range=chr1:77685184-77686678 5'pad=0 3'pad=0 strand=+ repeatMasking=none

GTGGCCCTCTGGTCCGGCTTTAGAACTCTGCGGTTTTGTGGAGCTCGAGA

GTCCGGGACAAGGTAGACGCAGGATATTGACCTTTTTATTTTTAACTGCA

TTTTCAGTTTGGAGGTTGTAAAATAGGTATAACATTTAAGAAAAAAAATT

GAAAGATATCTATTGGATGCTGATGATTATGAAACAAAGTAGCAAAGATA

AGAAGCCATTTGCTCATTCTGCTCCTTTGCCAGCAATATTTCACAAAGCC

CCTGACTCATTGACTGTGAGCAGCCTTGTGGAAGAATGCCTTGAAGACCA

TAAGCAGGATAGGGAATAGGTTTGCACATCTCTTGCCTGAATCACTGAAT

TTTTACAAAAG

>hg19_ct_ARAlincRNAs_9727_ARAlincRNA_0022.2 range=chr1:77685214-77687610 5'pad=0 3'pad=0 strand=+ repeatMasking=none

CGGTTTTGTGGAGCTCGAGAGTCCGGGACAAGATGTAACTTCATACCCAC

ACTTTGATGAGACTTTACTTGTATTAACCCATTTTCATACTGCTATGAAG

AAATACCCAAGACTGCATAATTTCTAAAGAAAAAGGCTTAGTGGACTCAC

AGTTCCACATGGCTGGGGAAGCCTCACAATCATGGTAGAAGATGAAGAAG

GAGCAAAGGCACATCTTACATGGCAGCAGGTGAGAGAGCATGTGCAGAGG

AACTACCCTTTATAAAACCATCAGAGGCCAGGTGCAGTGGCTCACACCTG

TAATCCTAGCACTTTGGGAGGCCGAGGCAGGCAGATCATCTGAGGTCAGG

CATTCAAGACCAGCCTGGCCAACATGGCAAAACCCCATGTTTCAAAATTA

GCTCAGTGTGGGGGCACATGCCTGTAATACCAGCTACTAGGGAGGCTGAG

GCAGGAGAATCGCTTGAACCTGGGAGGCAGAGGTTGCAGTGAGCCAAGAT

CACGCTACTGTACTCCAGCCTGGGCAACAGAGCCAGACTTTGTCTCAAAA

AATAAAATGAAATAAAATAAAAATAAATAAATAGATAGATAAATAAAACC

ATCAGAGCTCGTGAGAACTCACTACCATGACAACATGAGGGTAACTGATC

CCATAATTAAATTACTTCCCATCAGGTCCCTCCCTTGACACTTGGGGATT

ATGGGAGCTACAATTCAAGATGAGATTTGGGTTGGGACACAGCCAAACCA

TATCATTCCACCCTGGCCCCTCCAAAATCTCCTGTCCTCACATTTCAAAA

GCAATCATGCCTTACCAGCAGTCCCCCAAAGTCTTAACTCATTTCAGCAT

TAACTCAAAAGTCTACAGTCCAAAG

>hg19_ct_ARAlincRNAs_9727_ARAlincRNA_0022.3 range=chr1:77685227-77687610 5'pad=0 3'pad=0 strand=+ repeatMasking=none

CTCGAGAGTCCGGGACAAGGTAGACGCAGGATATTGACCTTTTTATTTTT

AACTGCATTTTCAGTTTGGAGGTTGTAAAATAGGTATAACATTTAAGAAA

AAAAATTGAAAGATATCTATTGGATGCTGATGATTATGAAACAAAGTAGC

AAAGATAAGAAGCCATTTGCTCATTCTGCTCCTTTGCCAGCAATATTTCA

CAAAGCCCCTGACTCATTGACTGTGAGCAGCCTTGTGGAAGAATGCCTTG

AAGACCATAAGCAGGATAGGGAATAGGTTTGCACATCTCTTGCCTGAATC

ACTGAATTTTTACAAAAGGTAAGTTTGGTGATCCTAGCCCTTGCCTCTTC

CTGTACATAAGATAATGTCTGACAAGATTAATGATTATGCCTCAAATTCA

TGACCAGATGTAACTTCATACCCACACTTTGATGAGACTTTACTTGTATT

AACCCATTTTCATACTGCTATGAAGAAATACCCAAGACTGCATAATTTCT

AAAGAAAAAGGCTTAGTGGACTCACAGTTCCACATGGCTGGGGAAGCCTC

ACAATCATGGTAGAAGATGAAGAAGGAGCAAAGGCACATCTTACATGGCA

GCAGGTGAGAGAGCATGTGCAGAGGAACTACCCTTTATAAAACCATCAGA

GGCCAGGTGCAGTGGCTCACACCTGTAATCCTAGCACTTTGGGAGGCCGA

GGCAGGCAGATCATCTGAGGTCAGGCATTCAAGACCAGCCTGGCCAACAT

GGCAAAACCCCATGTTTCAAAATTAGCTCAGTGTGGGGGCACATGCCTGT

AATACCAGCTACTAGGGAGGCTGAGGCAGGAGAATCGCTTGAACCTGGGA

GGCAGAGGTTGCAGTGAGCCAAGATCACGCTACTGTACTCCAGCCTGGGC

AACAGAGCCAGACTTTGTCTCAAAAAATAAAATGAAATAAAATAAAAATA

AATAAATAGATAGATAAATAAAACCATCAGAGCTCGTGAGAACTCACTAC

CATGACAACATGAGGGTAACTGATCCCATAATTAAATTACTTCCCATCAG

GTCCCTCCCTTGACACTTGGGGATTATGGGAGCTACAATTCAAGATGAGA

TTTGGGTTGGGACACAGCCAAACCATATCATTCCACCCTGGCCCCTCCAA

AATCTCCTGTCCTCACATTTCAAAAGCAATCATGCCTTACCAGCAGTCCC

CCAAAGTCTTAACTCATTTCAGCATTAACTCAAAAGTCTACAGTCCAAAG

>hg19_ct_ARAlincRNAs_9727_ARAlincRNA_0022.4 range=chr1:77686473-77686969 5'pad=0 3'pad=0 strand=+ repeatMasking=none

ATATCTATTGGATGCTGATGATTATGAAACAAAGTAGCAAAGATAAGAAG

CCATTTGCTCATTCTGCTCCTTTGCCAGCAATATTTCACAAAGCCCCTGA

CTCATTGACTGTGAGCAGCCTTGTGGAAGAATGCCTTGAAGACCATAAGC

AGGATAGGGAATAGGTTTGCACATCTCTTGCCTGAATCACTGAATTTTTA

TGTAACTTCATACCCACACTTTGATGAGACTTTACTTGTATTAACCCATT

TTCATACTGCTATGAAGAAATACCCAAGACTGCATAATTTCTAAAGAAAA

AGGCTTAGTGGACTCACAGTTCCACATGGCTGGGGAAGCCTCACAATCAT

GGTAGAAGATGAAGAAGGAGCAAAGGCACATCTTACATGGCAGCAGGTGA

G

>hg19_ct_ARAlincRNAs_9727_ARAlincRNA_0023.1 range=chr1:84743114-84763620 5'pad=0 3'pad=0 strand=- repeatMasking=none

TAGTCAGAGAAAAGCTAAAGATGCAGGGAGTTTTTTGTTTGTTTGTTTGT

TTGTTTGTTTTTTGAGACGGGGTCTTGCTCTGTCGCCCAGGCTGGAGTGC

AGTAGCGCGATCTCGGCTCAATGCAGGGATTTTTGTGTATCTGCATCTGC

AGAGGTTGTTGGAGCCATCCTGTCTATTCGTTTCTTTCTCCAGCCTCTCT

TAGCAGCTGGGAAGAGAGAGTGGAGAGAAAGAGGGATCAGGACAGATGTG

GACACGGCAGAAGAAGAGGAGCCTGCTGTGACGGACTTCCTTGAAATGAC

AAACAAGTAAATATTTAAGGGAATAAGTAAAACCAAAAGAAGGACTCATG

GGTTGGAAGAATGGAAGGAGAAGGAAAAGGCAGATGGGATAGGGATAGAC

ATTGAAGATTGCAAACAGGCTAGTAGCAAAGAAAGCAAAAACAAACAGGG

TTTTTAGAAGCCAGATAAACCTTGGATTCCTGGTGTTGTCACTTCTGAGC

TATCTCTGTTACCTTAGATGTTTATTTCTAATGAATTGGACA

>hg19_ct_ARAlincRNAs_9727_ARAlincRNA_0023.5 range=chr1:84757031-84761478 5'pad=0 3'pad=0 strand=- repeatMasking=none

ATGAGACAAAAAGGCCTGGGTGACAGCATATCTGTTTACAACATGATTTT

CTGAAAATTTTAAGCCTACTGTTGAGAACTACTACTCACAAAAAAAGATT

CCTTTCAAAATATTAGTGCTCATTGACAATACACCTGTTCAGCCAAGAAC

TCTGATGGAGATGTACAAGAAGATGAATGTTGTTTTCATGCCAGCTAGTA

AAACATCCATTCTGTAGCCCATGGATCAAGCAGCAAATCTGACTCTCAAT

TCTTATTATTTAAGAAATCCTTTTTATAAGTCTATAGCATCCATAGATAA

TGATTCCTCTGATGGAGCTGGGCATCATAATTGAAAGCCTTCTGGAAAGG

ATTCATCATCTTATATGCCATTAAGAACGTTCATAATTCATGAGAGGAGG

TCAAAATATAACATTAACATGAGTTTGGAAGAAGCTGATTCCAAGCCTCA

TGGATAACTTTGAGGGGTTCAAGACTTCAGTGGATGAAGTCCCTGCAGAT

GTGATAGAAATAGCAAGAGAAATAGAATTGGAAGTGGTGCATGAAGATAT

GACTGAATTGCTGCAATCTCATGATCAAACTTGAATGGATGAGGAGGTGC

TTCTTATGATTGAGCAAATAAAGTGGTTTCTTGAGATGGAAACTATTCCT

GGTGAATATGCTATGAACATTGTTCAAGTGACAACAGAGAATTTAGAATA

TTTCATAAACATAGCTGATAAAGCAGCATCAGGGTTTTAGAGGATTGACT

GCAATTCTGAAAGAAGTTTTACTGTGGTTAAAATGCTGTTCAGTAGCATC

ACATGCTACAGGGAAATCTTTCATTAAGGGAAGAGTCAATTGATGTGGCA

AACTTTACTGTTATCTTAAGAAATTGCCACAGCCACGCCAACCATCAGCA

ACCGACTACCCTAATGAGTTACCAGCCATCAACATTGAAGCAAGACCCTC

CACCAGCAAACAGATTACAACTTGCTAAAGGCTTTAAGATCGTTAGCATT

TTTTAAGCAATAAAGTATTTTTAAATTAAGATATGCACTTTTTAAAAGAT

ATAATGCCATTGCATACTGAATATACTACAATATAGTGTAAACATTTATA

TGCACTGGGAAACAAAAAAATGTGTGTGACTTGCTTTATTGCAGTGGTCT

AGAACTGAGCCCACAATGTTCCCGAGGGGCCTGCAGTTTGATTTTGGACA

TGCATAACTTAAGGGTTTATCTGTGTGAATGACCAAGTGGAAGTTGGAAA

TGTGGATTTGGATTTTTCATGTAGAAATGGGACTTTGAGAGTCAATTACT

TAGGAGAGGAAGTTGAAGAGCATGTGAAGAGGAAAAAGGGAGGACAGCAG

GCATGGGCATGGGGAGGTGGGAAGTCAGATAATAATACCAGGCAGGAATT

GGTTAAGGAGGAAAGGAAAGCTGCAGGGGTGTAGTAACATGGGGTGCAAG

GGAGGATGGAGAGATGAGGAGCAGCTTCTTAACAGGTCATATGCTCTAGA

GAGGTAGCCAATGACAACAGAAAAGAGGCCTCTGGATTTATAGATCAAGA

AACTATTGGGATTGAACTGCTTCCAGGGTTTTTTTTTTGAAATTTACCAG

AGTGATTTTGCTTGTCCCAGTGATTGGGGAGTGTATTAGTGCGTTTTCAC

ACTGCTATAAAGAAATACCCAAGACTGAGTAATTTATAAAAAGGTTTAAT

CGACTCACAGTTCCACATGGCTGGGGAGGCCTCAGGAAACTTACAATCAT

GGAGGTAGGGGAAGCAGGCACTCTCTTCACAACATGGCAGGAAAGAGAGA

AGTGCTGTGAAGGAGGAACTTCCAAAGTTATAAAACCATAAGATCTCATG

AGAACTCACTCACTATCATGAGAAGAGCCTGGGGGAAACTGCCTGCATGA

TCCAATCACCTGCCTCCCTTGACACATGGAGATTACAATTTGAGATGAGA

TTTGGTTGGAGACACAGAGCCAAACCATATGAGGGAGTGGGGCACTACTG

GAATATAGTTGGTGGGGTCAAAGATTCTAGACATCTTCCATTGACCAGGA

CAGTCGCGCAGAGCGAAGAATTGTCCTGCATCCTGTGTGACTTAGTTGTC

CCACTGCTCAGCTCATTTATGTAGGTGAAAAATCTGTTTATAATTATCTG

AGCCTAGAATCTATTTTATATATAAACATAAATTGTTTTCCACACAGTTT

TAAGTTATATCAAATTTTCCTGGAATGTTGTAAATTGGATGGCAATATCA

TTTATGGAATTTGAATCACCAATACAGTTCATTTTGTGGCTGTCTCTTTC

ATGGTGATTCTGCATATGGAACAAGTATCTGACTACTTTATTGTGTCTTT

TAGCTTAGGGATGCTTGAGCAGTTGCATGTTGGATCATGCAGTTTTATTA

TTATTATTATTATTTTTTTTTTTTTTGAGACGAAGTCTCACTCTTGTCAT

CCAGGCTGGAGTGCAATAACGCTATCTCAGCTCACTGCAACCTCTGCCTC

CTGAGTTCAGCAGTTCTCCTGCCTCAGCCTCCCGAGTAGCTGGGATTATG

GGTGCCTGCCACCACGCCCACCTAATTTTTGTATTTTGAGTAGAGACGGG

GTTTCACCATGTTGGCCAGGCTGGTCTTGAACTCCTGACCTCAGGCAAAT

CACCTGCCTTGGCCTCCCAAAGTGCTGGGATTACAGGTGTGAGCCACCGC

GCCTGGTGGATCATGCAATATTTTATGGTAAATTACTTTATTTTGTACTT

TATATTATAGTTAAGGTATTGCTTTAATTTTAGAAAATTACATAGGTAGG

TAGCTTATGGCTTCTACTTCACAATGGTAAAAAGCATATTGTTTCATATT

TGTTATGAAAAGGGATCATTAAGATTGACAAGATGGAGAGCTATTTATTC

CTTCAGTCAATAAACATCTCTGGAGTCCCTACTCATTCTGGGTACTTGAG

ATACATCAATATATGAAACAGAAAAAGTTTATTGCCCTTTTGGAGTTAAT

ATTGTAATGTGGGAGAGGCAGATGATAAATAATAAACATAAACAAAATAA

GTAAATTCTCTATTAGAAAGTTATAAATGCTGTGAAAAATAAAAGGAGGA

TAAAGGCAGTTGGGAGTGCAGGAGAGGGTGTGGGTTGAAATGTTAAATAT

AATGATCAGGGTAAACTTCATTGAGAAGGTGATAATCAAGGAAAGATCTG

GAGTTGGTAAGAGAATGGCCTTGTGGATTTCTGAGAAAAGAAATAGCAAG

GATGCCAGTGTGGTTGGATATGGGCAGGGGTCAAGTAGAAGGAGGACAGG

AAAACCCCGGGGCTGGAAACAAATAATGTAGGGCTTGTGGGCATTGCAAG

AAATTTCAGCTTTCACTCTGAATGAAATGGGCAGCTGTTGGAGAGTTCAG

AGCGGAAGAGTGACATGGTCAGGCATAGGTTTAAAGGATTATTCTGGCTG

CTCTATCAAGGATAAATCTTAACAGGCAATGGTGGAAGCAGACTAGGCAA

GATGCTATTGTAGTAATCCAGGCAAGAAATATTAGTGGCTTGAACTAGAG

TGGTAGCAACGAAAAGTGATGGTGTGGATGTGGAAAAGAAATCAAGAATT

CTATAGGGCATATTGGAAGAGTTTCAGGGGTTTGGGAGGGACGGAAGATG

GAGATACATTAAAGTATGTTCAGGGCCTTATAGGAATGGGACTTGGGGGA

TGACCTAGTAGTCTAGGACTTCTTGTGGTGACTCACATAAACAGGGATAA

AGGGTATAATGAAATCAGTCCCAGAGAAGGCAAGAAATAAAATGGTGAAG

AGGTTGTGAGTGTGGGGGGTATTGATGAATGTTTGAGTTCCCTGACTCTG

GATGGAGCTGAGAATCAGGTGGACTCTTGATCCACGTTGATGGAATGTTG

AAGGCCTGGGGAGAGGAGGACTCAGTTCCTAGGCCAACGCTTATTCATCA

CTCCTGTCAGGAGCAGGCTGACTTGTTCAGAGTGAGTAGTCCTCTCTACC

TCTGCTCTTGACCTTTTTGATGGACTTAGCCCTTTTGATGCAGTTGAGGA

TTGGGGCAAGCTGGTAATTACTCTAAGCACAAAGGCCCAGAAACAATGAT

GATTCAATAAAGTATATTCTAGAACACAGACTGAGGTAGACTTAAAATTC

TGGAAATGGAGAAATTTTGAGAAATTTGGCAAATGGGGAATTTTGGCAAA

GAAGGGCAGGAGAAGGTTTGGTCCTTAATGTGGAAGGATGCTCTAAGATT

GGGGATAGCAGACAACTGTGTTTGTACAGTGAAGTGAAGAAGCCACTAGA

TGCAGAAATGTGAAAATTCAGAACAGAGGGTGTAACTGATGTAATATTTT

CCTAGAGAAGAAGGAACTATTGATTGTTGATCTTTGGAAGAAAAGTGGCA

TAGTTTCTTCCTATAAGAAAAGAAAGAGGCCAGGCGCAGTGGCTCATG

>hg19_ct_ARAlincRNAs_9727_ARAlincRNA_0023.3 range=chr1:84757031-84763656 5'pad=0 3'pad=0 strand=- repeatMasking=none

TTCATTATATAAGAACCGTAAAGCAACTCTGAGAGTTAGTCAGAGAAAAG

CTAAAGATGCAGGGAGTTTTTTGTTTGTTTGTTTGTTTGTTTGTTTTTTG

AGACGGGGTCTTGCTCTGTCGCCCAGGCTGGAGTGCAGTAGCGCGATCTC

GGCTCAATGCAGGGATTTTTGTGTATCTGCATCTGCAGAGGTTGTTGGAG

CCATCCTGTCTATTCGTTTCTTTCTCCAGCCTCTCTTAGCAGCTGGGAAG

AGAGAGTGGAGAGAAAGAGGGATCAGGACAGATGTGGACACGGCAGAAGA

AGAGGAGCCTGCTGTGACGGACTTCCTTGAAATGACAAACAAGTAAATAT

TTAAGGGAATAAGTAAAACCAAAAGAAGGACTCATGGGTTGGAAGAATGG

AAGGAGAAGGAAAAGGCAGATGGGATAGGGATAGACATTGAAGATTGCAA

ACAGGCTAGTAGCAAAGAAAGCAAAAACAAACAGGGTTTTTAGAAGCCAG

ATAAACCTTGGATTCCTGGTGTTGTCACTTCTGAGCTATCTCTGTTACCT

TAGGTAAGTTCTTTAACCTCCCCAAACCTCTTTTGCCTCATCTGTAAAAT

TAAAAAAAAAAAAGTTCTGACTTTATCATGTGGCTGTGCCTATTAAAAAG

AGACAGGAAGCACATAGTATAGTGCTGGGCATATGCTAGGCAATTAATGT

TATTTACTATTATTATCTGCAGAAATTCTCTTTGTGTTAGTATCAAAAAA

TAAATTGGAGTGGAAGTACCAGTTAGGAGGCTGTGACAATACTCTAAGCT

ACTGGCAATGATAGCTTGAACTACATTGACAGAAAGGCAACTATGTTGCC

ATATGAGATAGGGAAAACAGAGTACAATCAATTGGATGTGGGGAAGGAAG

AAAAAGAATCAAATTGGTTGTTATTTCTAGATTAAGTAGATGATGTTGTC

ATTTTCATAAATAGATACAACTGATGGAAAAACAGGTTTTGGAGGAAGAT

GACCACTGGCTTTCCTCATTTTATTGTGCTTTGCTTTATTGCAGTTCACA

GACAATGTGTTTTTGTTGTTGTTGTTGTTGTTTATTTTACAAATTGACAG

TTTTTGGTAACCCTGTGTTGAACAAGTCTATTAGTGCAATTTTTCCAGCA

GTATGTGCTCACTTCATGTCTCTGTGTCACATTTTGGGCATTCTCATAAT

ATTTGAAAGTTTTTCATTTTTATTGTATCTGTTGTGGTGATCTGTGATCA

CTGATCTTTGATGTTACGATTGTAATTGTTTTAGGGTGCCACAGACCATG

CCCATTAATCTATAAATGTTGTGTGTGTTCTGACTGCTCCACTTACCAGC

TGTTCCCCTCCTCTCTCCCTCTCCTGCTCCTCCCTATTCCCTGAGACACA

ACAATATTGAAATTCGGCCAATTAATATCCTTACCATGGCCTCTAAGTGT

TCAAGTGAAAGGAAGAGTCATACTTCTCTCACTTCAAATCAAAAGCTAGA

AATGATAAACTTAGTGAGGAAGGCATGTCAGAAATCCAGATAAGCAAAAG

TTAGACGTCCGGTGCCAAACAGTTAGCCAAATTGTGAATGCAAACAAAAA

GTTCTTGAAGGAAATTAAAAATGCTGTTCTAACGCATACCTGAATGATAA

GAAAGTGAAACAGTCTTATTCCTGATATGGAGAAAGTTTTAGCGGTCTGC

ATAGAGGATCAAACCAGCCCCAACATTCCTTTAAGCCAAAGCCTAATCCA

GAGCAAGGCCCTAACTCAAGGGTGGAGAGGTAAGGAAACTGTAGAAAAGT

TTGAAGCTAGCAGAGTTTGATTTCTTAAACGAGGTTTAAGAAAAGAAGCA

TAACAATGAAGTACAAGGTGAAGAAGCAAGTGCTGATGGAGAAGCTGTAA

CACGTTATCCGGAAGATCTAGCAAAGGTAGATGGAGGTGGCTACAATAAT

CAGCAGTTTATCAATGTAGACTTAATAGCCTTCTATTGGAAGAAGATGCC

ATGTAAGATTTTTCTAGCCAGAGAAAAGTCAAAGCCTAACTTCAAAGCTT

CAAAGGACAGGCTTATTAGGGGCTAATGCACCCAGTGATCTTAAGTTGAA

GCCAATTTTCATTTACCATTATGAAAATTCTAGGGCCCTCAAGAATTATG

CTAAATTGACTTTACTTGTGCTTTATAGATGAGACAAAAAGGCCTGGGTG

ACAGCATATCTGTTTACAACATGATTTTCTGAAAATTTTAAGCCTACTGT

TGAGAACTACTACTCACAAAAAAAGATTCCTTTCAAAATATTAGTGCTCA

TTGACAATACACCTGTTCAGCCAAGAACTCTGATGGAGATGTACAAGAAG

ATGAATGTTGTTTTCATGCCAGCTAGTAAAACATCCATTCTGTAGCCCAT

GGATCAAGCAGCAAATCTGACTCTCAATTCTTATTATTTAAGAAATCCTT

TTTATAAGTCTATAGCATCCATAGATAATGATTCCTCTGATGGAGCTGGG

CATCATAATTGAAAGCCTTCTGGAAAGGATTCATCATCTTATATGCCATT

AAGAACGTTCATAATTCATGAGAGGAGGTCAAAATATAACATTAACATGA

GTTTGGAAGAAGCTGATTCCAAGCCTCATGGATAACTTTGAGGGGTTCAA

GACTTCAGTGGATGAAGTCCCTGCAGATGTGATAGAAATAGCAAGAGAAA

TAGAATTGGAAGTGGTGCATGAAGATATGACTGAATTGCTGCAATCTCAT

GATCAAACTTGAATGGATGAGGAGGTGCTTCTTATGATTGAGCAAATAAA

GTGGTTTCTTGAGATGGAAACTATTCCTGGTGAATATGCTATGAACATTG

TTCAAGTGACAACAGAGAATTTAGAATATTTCATAAACATAGCTGATAAA

GCAGCATCAGGGTTTTAGAGGATTGACTGCAATTCTGAAAGAAGTTTTAC

TGTGGTTAAAATGCTGTTCAGTAGCATCACATGCTACAGGGAAATCTTTC

ATTAAGGGAAGAGTCAATTGATGTGGCAAACTTTACTGTTATCTTAAGAA

ATTGCCACAGCCACGCCAACCATCAGCAACCGACTACCCTAATGAGTTAC

CAGCCATCAACATTGAAGCAAGACCCTCCACCAGCAAACAGATTACAACT

TGCTAAAGGCTTTAAGATCGTTAGCATTTTTTAAGCAATAAAGTATTTTT

AAATTAAGATATGCACTTTTTAAAAGATATAATGCCATTGCATACTGAAT

ATACTACAATATAGTGTAAACATTTATATGCACTGGGAAACAAAAAAATG

TGTGTGACTTGCTTTATTGCAGTGGTCTAGAACTGAGCCCACAATGTTCC

CGAGGGGCCTGCAGTTTGATTTTGGACATGCATAACTTAAGGGTTTATCT

GTGTGAATGACCAAGTGGAAGTTGGAAATGTGGATTTGGATTTTTCATGT

AGAAATGGGACTTTGAGAGTCAATTACTTAGGAGAGGAAGTTGAAGAGCA

TGTGAAGAGGAAAAAGGGAGGACAGCAGGCATGGGCATGGGGAGGTGGGA

AGTCAGATAATAATACCAGGCAGGAATTGGTTAAGGAGGAAAGGAAAGCT

GCAGGGGTGTAGTAACATGGGGTGCAAGGGAGGATGGAGAGATGAGGAGC

AGCTTCTTAACAGGTCATATGCTCTAGAGAGGTAGCCAATGACAACAGAA

AAGAGGCCTCTGGATTTATAGATCAAGAAACTATTGGGATTGAACTGCTT

CCAGGGTTTTTTTTTTGAAATTTACCAGAGTGATTTTGCTTGTCCCAGTG

ATTGGGGAGTGTATTAGTGCGTTTTCACACTGCTATAAAGAAATACCCAA

GACTGAGTAATTTATAAAAAGGTTTAATCGACTCACAGTTCCACATGGCT

GGGGAGGCCTCAGGAAACTTACAATCATGGAGGTAGGGGAAGCAGGCACT

CTCTTCACAACATGGCAGGAAAGAGAGAAGTGCTGTGAAGGAGGAACTTC

CAAAGTTATAAAACCATAAGATCTCATGAGAACTCACTCACTATCATGAG

AAGAGCCTGGGGGAAACTGCCTGCATGATCCAATCACCTGCCTCCCTTGA

CACATGGAGATTACAATTTGAGATGAGATTTGGTTGGAGACACAGAGCCA

AACCATATGAGGGAGTGGGGCACTACTGGAATATAGTTGGTGGGGTCAAA

GATTCTAGACATCTTCCATTGACCAGGACAGTCGCGCAGAGCGAAGAATT

GTCCTGCATCCTGTGTGACTTAGTTGTCCCACTGCTCAGCTCATTTATGT

AGGTGAAAAATCTGTTTATAATTATCTGAGCCTAGAATCTATTTTATATA

TAAACATAAATTGTTTTCCACACAGTTTTAAGTTATATCAAATTTTCCTG

GAATGTTGTAAATTGGATGGCAATATCATTTATGGAATTTGAATCACCAA

TACAGTTCATTTTGTGGCTGTCTCTTTCATGGTGATTCTGCATATGGAAC

AAGTATCTGACTACTTTATTGTGTCTTTTAGCTTAGGGATGCTTGAGCAG

TTGCATGTTGGATCATGCAGTTTTATTATTATTATTATTATTTTTTTTTT

TTTTGAGACGAAGTCTCACTCTTGTCATCCAGGCTGGAGTGCAATAACGC

TATCTCAGCTCACTGCAACCTCTGCCTCCTGAGTTCAGCAGTTCTCCTGC

CTCAGCCTCCCGAGTAGCTGGGATTATGGGTGCCTGCCACCACGCCCACC

TAATTTTTGTATTTTGAGTAGAGACGGGGTTTCACCATGTTGGCCAGGCT

GGTCTTGAACTCCTGACCTCAGGCAAATCACCTGCCTTGGCCTCCCAAAG

TGCTGGGATTACAGGTGTGAGCCACCGCGCCTGGTGGATCATGCAATATT

TTATGGTAAATTACTTTATTTTGTACTTTATATTATAGTTAAGGTATTGC

TTTAATTTTAGAAAATTACATAGGTAGGTAGCTTATGGCTTCTACTTCAC

AATGGTAAAAAGCATATTGTTTCATATTTGTTATGAAAAGGGATCATTAA

GATTGACAAGATGGAGAGCTATTTATTCCTTCAGTCAATAAACATCTCTG

GAGTCCCTACTCATTCTGGGTACTTGAGATACATCAATATATGAAACAGA

AAAAGTTTATTGCCCTTTTGGAGTTAATATTGTAATGTGGGAGAGGCAGA

TGATAAATAATAAACATAAACAAAATAAGTAAATTCTCTATTAGAAAGTT

ATAAATGCTGTGAAAAATAAAAGGAGGATAAAGGCAGTTGGGAGTGCAGG

AGAGGGTGTGGGTTGAAATGTTAAATATAATGATCAGGGTAAACTTCATT

GAGAAGGTGATAATCAAGGAAAGATCTGGAGTTGGTAAGAGAATGGCCTT

GTGGATTTCTGAGAAAAGAAATAGCAAGGATGCCAGTGTGGTTGGATATG

GGCAGGGGTCAAGTAGAAGGAGGACAGGAAAACCCCGGGGCTGGAAACAA

ATAATGTAGGGCTTGTGGGCATTGCAAGAAATTTCAGCTTTCACTCTGAA

TGAAATGGGCAGCTGTTGGAGAGTTCAGAGCGGAAGAGTGACATGGTCAG

GCATAGGTTTAAAGGATTATTCTGGCTGCTCTATCAAGGATAAATCTTAA

CAGGCAATGGTGGAAGCAGACTAGGCAAGATGCTATTGTAGTAATCCAGG

CAAGAAATATTAGTGGCTTGAACTAGAGTGGTAGCAACGAAAAGTGATGG

TGTGGATGTGGAAAAGAAATCAAGAATTCTATAGGGCATATTGGAAGAGT

TTCAGGGGTTTGGGAGGGACGGAAGATGGAGATACATTAAAGTATGTTCA

GGGCCTTATAGGAATGGGACTTGGGGGATGACCTAGTAGTCTAGGACTTC

TTGTGGTGACTCACATAAACAGGGATAAAGGGTATAATGAAATCAGTCCC

AGAGAAGGCAAGAAATAAAATGGTGAAGAGGTTGTGAGTGTGGGGGGTAT

TGATGAATGTTTGAGTTCCCTGACTCTGGATGGAGCTGAGAATCAGGTGG

ACTCTTGATCCACGTTGATGGAATGTTGAAGGCCTGGGGAGAGGAGGACT

CAGTTCCTAGGCCAACGCTTATTCATCACTCCTGTCAGGAGCAGGCTGAC

TTGTTCAGAGTGAGTAGTCCTCTCTACCTCTGCTCTTGACCTTTTTGATG

GACTTAGCCCTTTTGATGCAGTTGAGGATTGGGGCAAGCTGGTAATTACT

CTAAGCACAAAGGCCCAGAAACAATGATGATTCAATAAAGTATATTCTAG

AACACAGACTGAGGTAGACTTAAAATTCTGGAAATGGAGAAATTTTGAGA

AATTTGGCAAATGGGGAATTTTGGCAAAGAAGGGCAGGAGAAGGTTTGGT

CCTTAATGTGGAAGGATGCTCTAAGATTGGGGATAGCAGACAACTGTGTT

TGTACAGTGAAGTGAAGAAGCCACTAGATGCAGAAATGTGAAAATTCAGA

ACAGAGGGTGTAACTGATGTAATATTTTCCTAGAGAAGAAGGAACTATTG

ATTGTTGATCTTTGGAAGAAAAGTGGCATAGTTTCTTCCTATAAGAAAAG

AAAGAGGCCAGGCGCAGTGGCTCATG

>hg19_ct_ARAlincRNAs_9727_ARAlincRNA_0023.4 range=chr1:84763104-84763483 5'pad=0 3'pad=0 strand=- repeatMasking=none

TATCTGCATCTGCAGAGGTTGTTGGAGCCATCCTGTCTATTCGTTTCTTT

CTCCAGCCTCTCTTAGCAGCTGGGAAGAGAGAGTGGAGAGAAAGAGGGAT

CAGGACAGATGTGGACACGGCAGAAGAAGAGGAGCCTGCTGTGACGGACT

TCCTTGAAATGACAAACAAGTAAATATTTAAGGGAATAAGTAAAACCAAA

AGAAGGACTCATGGGTTGGAAGAATGGAAGGAGAAGGAAAAGGCAGATGG

GATAGGGATAGACATTGAAGATTGCAAACAGGCTAGTAGCAAAGAAAGCA

AAAACAAACAGGGTTTTTAGAAGCCAGATAAACCTTGGATTCCTGGTGTT

GTCACTTCTGAGCTATCTCTGTTACCTTAG

>hg19_ct_ARAlincRNAs_9727_ARAlincRNA_0023.2 range=chr1:84763732-84763755 5'pad=0 3'pad=0 strand=- repeatMasking=none

AAGCCCTGAGCATGTAAAAGGAGG

>hg19_ct_ARAlincRNAs_9727_ARAlincRNA_0024.5 range=chr1:85063635-85084567 5'pad=0 3'pad=0 strand=- repeatMasking=none

CAGAAAAGCCTTTGATGCTGTATTCTTAGCACATGGAACCCTAAACCAAA

GCCTGCACTGGGGCATTTGTGAGAAGCGTGTGTGCAAAGCCTGAGAGACT

CCCCTTTTGGGCATGCTGCTGGGCAGGGGTGTTCTGGTTTGACTTTGTAC

ATGTTTCCGTTTACATCTTCATTCCATTTTGAGTTGATTTTTGTATATGG

TGAGAAATAGGTGTCTAGTTTCATTCTTCTGCATGTGGATATCCAGCTTC

CCCAGCACCATTTATTGAAGAAACTGTCATTTTGGGGTAACTGCTTTCTA

ACAGGTCTCTGGGGTTTGGTATTACTGCGCCAGAACCCTTTCATATAGCT

GTCAGAGAGATGGCAGTGGAACAGCTCAGTTTATATCTCTCTGCTCTTTA

AACCTATCTGATGATTCTTCATTGCCAGTAGGATAAAGTCCAAACACCTT

GGCTTGTCATATCAAGTCCTTTCCCATCTGGTGCCCATCATCCCAGCTAC

CTCCTCTGATACTCTCTACCATGCATCCTATGATTGCCTCCCAATTTTAC

ATTCTCTTCATCTTCCTGATTAAAATAGTGATTGCTGATTACATGTCCTC

TCTTTTGCAGTTAGATGTGGCCAGGTGACAAAGTTCTGTACAGTGGAATG

TAAACAGAAGTATCATGTGAGAGTTTCCAAGAACTTTTTCAGGAGGGAGG

AAACACATGTCCTTTGCCCTCCCTTTTTGTTTGCTGCCTTCTCCATCCTA

TAGCCTGGAATACAGACATGATGGCTACCATCTTGGACTATGAACATGAG

GATCACACCCAAGGGAAGGAGAGGCAGCAAGCTGGAAGGTACTATGGAGC

AGAACTGCCACAGCCTGAGTACCTACCTCTGGAACTTTCTGTGAGAAATG

AAATGGGCCCAACAGTTCCATAAACAGTTGTTTTGGGATAAACATAGAAA

TGGCCCCTAGCTTGAAATTTGCATTTGTTTTATCTGAGTTCCCTCCTCAG

GAAAGGACCTTCAGGCCTCTCAAAAAAGTATCAAGAGAACTGACACCAGA

TGCCTCCTTGCCCCTCCCTAGTTCCTGTTTTCTTAAACATTGTTACATAT

CTTCCCTGCTATGTAAGCCCCTAGATTTAGTCAGTCAGGGAGATGGATTT

GAGACTGAGCTCTCATAACTTGGCTGCAGCACCCGATTAAAGCCTTCTTC

CTTGGCAATACTTATTGTCTCAGTGATTGGCTTTCTGTGCGTCAGTACCT

AGACTGAACCCCTGGTGTTTCCATAACTTGGTCAAGCCACTGTTATTCTG

AGTATCTGTCCCTTGCTTGGCAGTTTTGGAGTTTGCCCACCAGATATGCC

ATCCAAGAAGACCAAAAGTACAGCAGGATGATTGTGAACAAGACCAGGGA

CCTCAGCCCTTGCAAAAAAAGGTTGAAGACTATGTACATGAACGACATTC

TAGGCTATCCT

>hg19_ct_ARAlincRNAs_9727_ARAlincRNA_0024.6 range=chr1:85079645-85084564 5'pad=0 3'pad=0 strand=- repeatMasking=none

AAAAGCCTTTGATGCTGTATTCTTAGCACATGGAACCCTAAACCAAAGCC

TGCACTGGGGCATTTGTGAGAAGCGTGTGTGCAAAGCCTGAGAGACTCCC

CTTTTGGGCATGCTGCTGGGCAGGGGTGTTCTGGTTTGACTTTGTACATG

TTTCCGTTTACATCTTCATTCCATTTTGAGTTGATTTTTGTATATGGTGA

GAAATAGGTGTCTAGTTTCATTCTTCTGCATGTGGATATCCAGCTTCCCC

AGCACCATTTATTGAAGAAACTGTCATTTTGGGGTAACTGCTTTCTAACA

GGTCTCTGGGGTTTGGTATTACTGCGCCAGAACCCTTTCATATAGCTGTC

AGAGAGATGGCAGTGGAACAGCTCAGTTTATATCTCTCTGCTCTTTAAAC

CTATCTGATGATTCTTCATTGCCAGTAGGATAAAGTCCAAACACCTTGGC

TTGTCATATCAAGTCCTTTCCCATCTGGTGCCCATCATCCCAGCTACCTC

CTCTGATACTCTCTACCATGCATCCTATGATTGCCTCCCAATTTTACATT

CTCTTCATCTTCCTGATTAAAATAGTGATTGCTGATTACATGTCCTCTCT

TTTGCAGTTAGATGTGGCCAGGTGACAAAGTTCTGTACAGTGGAATGTAA

ACAGAAGTATCATGTGAGAGTTTCCAAGAACTTTTTCAGGAGGGAGGAAA

CACATGTCCTTTGCCCTCCCTTTTTGTTTGCTGCCTTCTCCATCCTATAG

CCTGGAATACAGACATGATGGCTACCATCTTGGACTATGAACATGAGGAT

CACACCCAAGGGAAGGAGAGGCAGCAAGCTGGAAGGTACTATGGAGCAGA

ACTGCCACAGCCTGAGTACCTACCTCTGGAACTTTCTGTGAGAAATGAAA

TGGGCCCAACAGTTCCATAAACAGTTGTTTTGGGATAAACATAGAAATGG

CCCCTAGCTTGAAATTTGCATTTGTTTTATCTGAGTTCCCTCCTCAGGAA

AGGACCTTCAGGCCTCTCAAAAAAGTATCAAGAGAACTGACACCAGATGC

CTCCTTGCCCCTCCCTAGTTCCTGTTTTCTTAAACATTGTTACATATCTT

CCCTGCTATGTAAGCCCCTAGATTTAGTCAGTCAGGGAGATGGATTTGAG

ACTGAGCTCTCATAACTTGGCTGCAGCACCCGATTAAAGCCTTCTTCCTT

GGCAATACTTATTGTCTCAGTGATTGGCTTTCTGTGCGTCAGTACCTAGA

CTGAACCCCTGGTGTTTCCATAACTTGGTCAAGCCACTGTTATTCTGAGT

ATCTGTCCCTTGC

>hg19_ct_ARAlincRNAs_9727_ARAlincRNA_0024.7 range=chr1:85079645-85086673 5'pad=0 3'pad=0 strand=- repeatMasking=none

GTCGCGGGAGAGCCAGCGCAAAGTGGAAGGCACCTTGTACGCTGTGAAGC

GGAGATGCAGATGTTACCCTAACCAGAAGCCCACCCCGCCACAAATACTG

GAGCGCCCAGCATGCGTGCGCCACCCGCTTCCCTGCAGAGGGCGCCGCGG

GCCCGCCTCGCCGAGCGCGGGGTGAGATCCCAGTAGCCCAGAGCAGCCCG

CGGGGCAGGCCCGGGCTGCCGCGGACTCCTCTTGCCTGGAATACAGACAT

GATGGCTACCATCTTGGACTATGAACATGAGGATCACACCCAAGGGAAGG

AGAGGCAGCAAGCTGGAAGGTACTATGGAGCAGAACTGCCACAGCCTGAG

TACCTACCTCTGGAACTTTCTGTGAGAAATGAAATGGGCCCAACAGTTCC

ATAAACAGTTGTTTTGGGATAAACATAGAAATGGCCCCTAGCTTGAAATT

TGCATTTGTTTTATCTGAGTTCCCTCCTCAGGAAAGGACCTTCAGGCCTC

TCAAAAAAGTATCAAGAGAACTGACACCAGATGCCTCCTTGCCCCTCCCT

AGTTCCTGTTTTCTTAAACATTGTTACATATCTTCCCTGCTATGTAAGCC

CCTAGATTTAGTCAGTCAGGGAGATGGATTTGAGACTGAGCTCTCATAAC

TTGGCTGCAGCACCCGATTAAAGCCTTCTTCCTTGGCAATACTTATTGTC

TCAGTGATTGGCTTTCTGTGCGTCAGTACCTAGACTGAACCCCTGGTGTT

TCCATAACTTGGTCAAGCCACTGTTATTCTGAGTATCTGTCCCTTGC

>hg19_ct_ARAlincRNAs_9727_ARAlincRNA_0024.2 range=chr1:85079755-85086497 5'pad=0 3'pad=0 strand=- repeatMasking=none

ATCCCAGTAGCCCAGAGCAGCCCGCGGGGCAGGCCCGGGCTGCCGCGGAC

TCCTCTTGTTAGATGTGGCCAGGTGACAAAGTTCTGTACAGTGGAATGTA

AACAGAAGTATCATGTGAGAGTTTCCAAGAACTTTTTCAGGAGGGAGGAA

ACACATGTCCTTTGCCCTCCCTTTTTGTTTGCTGCCTTCTCCATCCTATA

GCCTGGAATACAGACATGATGGCTACCATCTTGGACTATGAACATGAGGA

TCACACCCAAGGGAAGGAGAGGCAGCAAGCTGGAAGGTACTATGGAGCAG

AACTGCCACAGCCTGAGTACCTACCTCTGGAACTTTCTGTGAGAAATGAA

ATGGGCCCAACAGTTCCATAAACAGTTGTTTTGGGATAAACATAGAAATG

GCCCCTAGCTTGAAATTTGCATTTGTTTTATCTGAGTTCCCTCCTCAGGA

AAGGACCTTCAGGCCTCTCAAAAAAGTATCAAGAGAACTGACACCAGATG

CCTCCTTGCCCCTCCCTAGTTCCTGTTTTCTTAAACATTGTTACATATCT

TCCCTGCTATGTAAGCCCCTAGATTTAGTCAGTCAGGGAGATGGATTTGA

GACTGAGCTCTCATAACTTGGCTGCAGCACCCGATTAAAGCCTTCTTCCT

TGGC

>hg19_ct_ARAlincRNAs_9727_ARAlincRNA_0024.3 range=chr1:85079755-85086673 5'pad=0 3'pad=0 strand=- repeatMasking=none

GTCGCGGGAGAGCCAGCGCAAAGTGGAAGGCACCTTGTACGCTGTGAAGC

GGAGATGCAGATGTTACCCTAACCAGAAGCCCACCCCGCCACAAATACTG

GAGCGCCCAGCATGCGTGCGCCACCCGCTTCCCTGCAGAGGGCGCCGCGG

GCCCGCCTCGCCGAGCGCGGGGTGAGATCCCAGTAGCCCAGAGCAGCCCG

CGGGGCAGGCCCGGGCTGCCGCGGACTCCTCTTGCAGAAAAGCCTTTGAT

GCTGTATTCTTAGCACATGGAACCCTAAACCAAAGCCTGCACTGGGGCAT

TTGTGAGAAGCGTGTGTGCAAAGCCTGAGAGACTCCCCTTTTGGGCATGC

TGCTGGGCAGGGGTGTTCTGGTTTGACTTTGTACATTTAGATGTGGCCAG

GTGACAAAGTTCTGTACAGTGGAATGTAAACAGAAGTATCATGTGAGAGT

TTCCAAGAACTTTTTCAGGAGGGAGGAAACACATGTCCTTTGCCCTCCCT

TTTTGTTTGCTGCCTTCTCCATCCTATAGCCTGGAATACAGACATGATGG

CTACCATCTTGGACTATGAACATGAGGATCACACCCAAGGGAAGGAGAGG

CAGCAAGCTGGAAGGTACTATGGAGCAGAACTGCCACAGCCTGAGTACCT

ACCTCTGGAACTTTCTGTGAGAAATGAAATGGGCCCAACAGTTCCATAAA

CAGTTGTTTTGGGATAAACATAGAAATGGCCCCTAGCTTGAAATTTGCAT

TTGTTTTATCTGAGTTCCCTCCTCAGGAAAGGACCTTCAGGCCTCTCAAA

AAAGTATCAAGAGAACTGACACCAGATGCCTCCTTGCCCCTCCCTAGTTC

CTGTTTTCTTAAACATTGTTACATATCTTCCCTGCTATGTAAGCCCCTAG

ATTTAGTCAGTCAGGGAGATGGATTTGAGACTGAGCTCTCATAACTTGGC

TGCAGCACCCGATTAAAGCCTTCTTCCTTGGC

>hg19_ct_ARAlincRNAs_9727_ARAlincRNA_0024.1 range=chr1:85080035-85086673 5'pad=0 3'pad=0 strand=- repeatMasking=none

GTCGCGGGAGAGCCAGCGCAAAGTGGAAGGCACCTTGTACGCTGTGAAGC

GGAGATGCAGATGTTACCCTAACCAGAAGCCCACCCCGCCACAAATACTG

GAGCGCCCAGCATGCGTGCGCCACCCGCTTCCCTGCAGAGGGCGCCGCGG

GCCCGCCTCGCCGAGCGCGGGGTGAGATCCCAGTAGCCCAGAGCAGCCCG

CGGGGCAGGCCCGGGCTGCCGCGGACTCCTCTTGAAAAGCCTTTGATGCT

GTATTCTTAGCACATGGAACCCTAAACCAAAGCCTGCACTGGGGCATTTG

TGAGAAGCGTGTGTGCAAAGCCTGAGAGACTCCCCTTTTGGGCATGCTGC

TGGGCAGGGGTGTTCTGGTTTGACTTTGTACATCCTGGAATACAGACATG

ATGGCTACCATCTTGGACTATGAACATGAGGATCACACCCAAGGGAAGGA

GAGGCAGCAAGCTGGAAGGTACTATGGAGCAGAACTGCCACAGCCTGAGT

ACCTACCTCTGGAACTTTCTGTGAGAAATGAAATGGGCCCAACAGTTCCA

TAAACA

>hg19_ct_ARAlincRNAs_9727_ARAlincRNA_0024.4 range=chr1:85084416-85086673 5'pad=0 3'pad=0 strand=- repeatMasking=none

GTCGCGGGAGAGCCAGCGCAAAGTGGAAGGCACCTTGTACGCTGTGAAGC

GGAGATGCAGATGTTACCCTAACCAGAAGCCCACCCCGCCACAAATACTG

GAGCGCCCAGCATGCGTGCGCCACCCGCTTCCCTGCAGAGGGCGCCGCGG

GCCCGCCTCGCCGAGCGCGGGGTGAGATCCCAGTAGCCCAGAGCAGCCCG

CGGGGCAGGCCCGGGCTGCCGCGGACTCCTCTTGAAAAGCCTTTGATGCT

GTATTCTTAGCACATGGAACCCTAAACCAAAGCCTGCACTGGGGCATTTG

TGAGAAGCGTGTGTGCAAAGCCTGAGAGACTCCCCTTTTGGGCATGCTGC

TGGGCAGGGGTGTTCTGGTTTGACTTTGTACAT

>hg19_ct_ARAlincRNAs_9727_ARAlincRNA_0025.1 range=chr1:90285733-90286427 5'pad=0 3'pad=0 strand=- repeatMasking=none

GACGGGGTCTGTGTGGCGAGGGAGGGGACCCGGGGTTGGAGACTGGCGGC

GAGCGACTGCTAGATGCGGAGCAGGTGCTTGGGAGGGGAAGAACGGCAGC

CTACATAACCATCGCCGTAATGCTCTCGCAAGCGTCCACCTGGGCAGGGC

TCGGTGCCGGGCAGGCGCAGCTGCCAGTTAGCTGCGAGGCATGGTCAGGA

GCCTTCACCCGTCCTGGGCGAAGGAGGCTGTCCCGTCGACTAAAACTCTG

ATATTAAGACTTGGCGCTGGGGGTGGGGAGTTGAATTACGTTAAAAAGTC

ACTTAAGTGCCTTCCAACTCTAAGGAGAAGTACTTATCTGAGCTTTGGGG

CACACACAACTACTACTTGGCATGGAGAGTTTCTCAGCTTCATGTGTTCC

GTCTGTTATTGAAGCTGTATTGGAAAGTCGGTTCTTGTTTTTATCTGAGC

CCGCCGTTTTCATCCCATTCTTTTCGTCAAAAATATGTTACATCTGAAAG

AGGCAGATGAAAAGATTATTCTATGCTAAGAGGGTTTTGCAGCCCGATAA

TGTCACCGTTTTTTGTTGTTGTTGTTGTAGGATCGGGGTTCATTTGCAGT

CTTTTTTGTGGGTGAGCAGAGGCAGGGGCCAATAAATAAATTAAGGCGGG

ATATTTGGAGTTATTTGGTTGTAATAAAATATGTGGTTCAACCCA

>hg19_ct_ARAlincRNAs_9727_ARAlincRNA_0026.1 range=chr1:94312730-94314495 5'pad=0 3'pad=0 strand=+ repeatMasking=none

CCTGCAACTCTGGGGTCCCGGCCGGGCTGGAGCGGCCGCCGGAGAGACCT

GGTAGGCGGGGCGCTTGGGGCAGGAAGGGAGCCGGGGAGGGAAAAGCGCG

ACTGTGATTCGTACGGCTGGGCCCGGACCGTGAAGGGGACCGGGTTGCGG

CTGCCCTCACACCGACCAGGTGGGACCGTCGGACTGCGGCCGCCCTTGGC

TCAACTGGGTCGCCGACCTTGTTGAGGGGCGACCACTGCGGGGAGACCGA

GAGCCTGCGGCTGGCGTTTGAGGCGGATGGCAGTGCCCTGAGCGCGGCGG

CTGGGTCTCGGTGACACTGACGACGGGAGGCGCGGTCGGAAGAGCGCGGG

GCCGTCGCCTCTGGCTTAACATAGCAGATGCGCTGAGACTCCAACAGGTG

GCTCCGTGGCGCAATGGATAGCGCATTGGACTTCTAGAGGCTGAAGGCAT

TCAAAGGTTCCGGGTTCGAGTCCCGGCGGAGTCGTAACGCTTTTTTCCCT

CCCCCCTACAATTTATTTTCTGCCTCCATCATGTCTCTTATTTTTTTTTT

TTGCTAAACGGTTTAACTTCTCTCTCATTCCCTGCTCTCGCAGTTCACTG

CATTCGGTTCTTGCGGTCCTTTCTTAAGCGGCTCGCAGGGTCCCGAGCCC

CTCAGCTCCCCGGGCCTCGGTGGCCCAGGGCCCAGCTCAGCCGACTGGGC

AGTCGATGTAGGTCCTGAGAAGAGCGGCGGCGGCGGCGGCGGCGGCGGCG

AAGGAAAAGCGACACTGAAGCGAAGGCTCGCGGTTTCGGCCTAAAGGAAA

GTGCAGGGGAGGCCCGGGTCTCGGGTGACACCCCCACGCCTTGAGCGGGA

CTGTGGTTGCGAGGGAGGGACGAGAAAACACGCCTACGCCCTGCGTGCGC

TGTGCTGACTACCTCTCCAGCATGGAGGCTTCCAGATGAGCAGACACCCG

AATCCCAGCGGGAGCCCGTCAAAAATGGAGATTTCTGCCGTCACTCTGGG

ACTCTTAATTCGGTCCGTCTGGGGTGGGGCCCAGGAATCTGCATTTCTGA

TAAGGTCACCCGCTCCCCTCCCCCATTATTCCAGTGCAAAGAGGTCCTAG

GCCCAGTGCCCATCAGCTCCTCAAGACAGGAATTACTATATCTTTTTCTG

ACAACTGTTAACTTTGTACAAGGTTAGCAAATAAATCCAGGAATGAATGG

AATCTTAAAACTCGTAAAACAACAATGAAAGGTAATTCACACAAAAGATA

CAAAATCCAAAATTGTCAAAAAAGATACAGGGAAAAGTAAACATCCATGT

TATTCCTATAGTCCAGCCTTGCAATTCTCTCCACAAGCCATTAGTGGTAA

TGGTTTCTTGGAAATATTTTCAGATTTATTTTATGCCCATATCAGCATTT

TTAACATCTTTTGTCATAAATATGTCGTAAATAACAATGTTATTGATACA

TATCCTTTTAAAAACACAATGGTAGTATGCTATACTGGCTTGTTTTACTT

ATGAATATATGTAGAGCTGCCTCGTTCCATTGTACGTATGTATGGTAATG

ATTTAACAGTCCCTAATAGCTGGACATTTAGATTGCCAGTATTTTCCTAT

TACAAACAATGCATCAGTGAACATCCTTGTTCTTTTATTATTGTACACAA

GTACAGGTACATCTATCTGTAGGATAGATTCCTAGAAAGTTGAATTGCTG

GTTCAAAGGTTTGTGCGTTCTTAATCTGTACATTTTTTAATATATATTGG

CAAATTGCCCCATAGA

>hg19_ct_ARAlincRNAs_9727_ARAlincRNA_0027.6 range=chr1:95104017-95209549 5'pad=0 3'pad=0 strand=- repeatMasking=none

ATGTACACTAGATTTTGATGGCATTTTTCCAGGGACCCTGGGAGCATCTG

TGTTCTCGTGTTCTTCTTGCCTGTGAGGCCTCTTCTCTCTGAGGACAGAT

GGTAGGACAGAGAGAAGACAGCAAATGGAAAACAAAGGCTGCTATTTTTT

TGTGGGGTGAATTCCTTGGAAGATGAGGGCCAAGTCTTATTCTTCTCAGT

CTCTTCTGCATCTAGCAGAAGAATAATGGTTGCACAGAAAATCTTGGTTC

AGTGGTTGAGTGAATGAATGGATGAACAGGCTCTTGCACACAAAGAAGAG

ACATGATGTATTTGCATCCTTCCCTGCTTCAGCCAGTTTTCTGAAGCTCA

GAGGGACACCTTGTATTGCTGGATGATAAAAACAGGAGCAAAGTGATGAA

GTGCTGACAAGGCAACAATAGAACATGAGAGATTCACTGCTGTGTAGGAA

GAGATCTTCGGTGACCATGTAGCCTGAAGCTCTCATTTTGTCATCGAGGG

ACACCAAAACTCAGAACAGATAGAGTCTTGCTCTGTGACCCAGGCTTGTG

TGCAGCGGTACGATATTGGCTCACTGCGACCTCCACCTCCCAGGTTCAAG

CAATTCTCCTGCCTCAG

>hg19_ct_ARAlincRNAs_9727_ARAlincRNA_0027.8 range=chr1:95123089-95197993 5'pad=0 3'pad=0 strand=- repeatMasking=none

GTGAATTCCTTGGAAGATGAGGGCCAAGTCTTATTCTTCTCAGTCTCTTC

TGCATCTAGCAGAAGAATAATGGTTGCACAGAAAATCTTGGTTCAGTGGT

TGAGTGAATGAATGGATGAACAGGCTCTTGCACACAAAGAAGAGACATGA

TGTATTTGCATCCTTCCCTGCTTCAGCCAGGTCCCCAGACAGTCCCGGTG

TGCACACTTCCCTGCTTCTGTTCATGATGTTCTCTGAACCTGGGATCACC

TTTCCCCTTCCTCTTACCTGCCCGATCCTACTTGTCCTCCCAGATTCAGT

TTTCTGAAGCTCAGAGGGACACCTTGTATTGCTGGATGATAAAAACAGGA

GCAAAGTGATGAAGTGCTGACAAGGCAACAATAGAACATGAGAGATTCAC

TGCTGTGTAGGAAGAGATCTTCGGTGACCATGTAGCCTGAAGCTCTCATT

TTGTCATCGAGGGACACCAAAACTCAGAACAGCAGCAGCTCACGCTGCTG

ATGCAACACAGGTGAAGAGCACCTTCCCCTCCCCCACCTGTGGGCTGATT

CCCACCACGTGGATCCCAAGGCCATCCCAGGAACTCTTTGGAGGGGAGAA

GCCCAGTGGAAACTGTCAGGCCTCTGAGCACAAGCCAAGCCATCGCATCC

CCTGTGACTTGCACGTATATGCCCAGATGGCCTGAAGTAACTGAAGAATC

ACAAAAGAAGTGAAAAGGCCCTGCCCCACCTTAACTGATGACATTCCACC

ATTGTGATTTGTTCCTGCCCCACCTTCACTGAGTGATTAACCCTGTGAAT

TTCCTTCTCCTGGCTCAGAAGCTCCCCCACTGAGCACCTTGTGACCCCCG

CCCCTGCCCACCAGAGAACAACCCCCTTTGACTGTAATTTTCCATTACCT

TCCCAAATCCTATAAAACGGCCCCACCCCTATCTCCCTTCGCTGACTCTC

TTTTCGGACTCAGCCCGCCTGCACCCAGGTGAAATAAACAGCCATGTTGC

TCACA

>hg19_ct_ARAlincRNAs_9727_ARAlincRNA_0027.9 range=chr1:95123481-95285837 5'pad=0 3'pad=0 strand=- repeatMasking=none

CTCTGCGCCTGGCCCGGCGACTGCGGGCGCTGCGGTCCACTGGGTCCGCC

TCTTCCCGGCATGGGTTTCTGCCCAGCTCACCCGCTGTCAGCTGGGGTCC

TGCTCTGGTGGGAGGAAGAGGCTCAGACGCTTCCCTGCCCTCTCGCCTCA

ACCACCTCGAGGCAGCGGCTCCCAGGATGTGCACTTTGACGACTAAAGCT

GAGCCGGCGCCGCCACGACCTTGGGCGGGTGGTCGGCCTCTGCCCTGAGC

AGGAAGTAGAAAGTCTCAGCAGACCCTTCCTGAGGGCCGAGCAACAGTGT

AGTGGCGTATTCCACATAGCAAACAGCAGCAGCTCACGCTGCTGATGCAA

CACAGGTGAAGAGCACCTTCCCCTCCCCCACCTGTGGGCTGATTCCCACC

ACGTGGATCCCAAGGCCATCCCAGGAACTCTTTGGAGGGGAGAAGCCCAG

TGGAAAC

>hg19_ct_ARAlincRNAs_9727_ARAlincRNA_0027.10 range=chr1:95123762-95197901 5'pad=0 3'pad=0 strand=- repeatMasking=none

TCAGTGGTTGAGTGAATGAATGGATGAACAGGCTCTTGCACACAAAGAAG

AGACATGATGTATTTGCATCCTTCCCTGCTTCAGCCAGTTTTCTGAAGCT

CAGAGGGACACCTTGTATTGCTGGATGATAAAAACAGGAGCAAAGTGATG

AAGTGCTGACAAGGCAACAATAGAACATGAGAGATTCACTGCTGTGTAGG

AAGAGATCTTCGGTGACCATGTAGCCTGAAGCTCTCATTTTGTCATCGAG

GGACACCAAAACTCAGAACAGTGCTAAGCTCCTACATCCTGTTCTGGAAG

GACAATCCTGAGCACACGATGAGCCATATAAACTTCAGACTGACGCTGAG

TGAAAGAATGCTGGAAAAGCATCACAGGCCAGGGCAGCAAAGTCTTCAAG

GTCATCCGTGCTCTGATGATGTCACGCTTCTTCCCTGTCTGGAAGACATT

TCCCCAAGAGCATACCACCAACATCAGGGAAATGGAATCCAACTGGTCGC

TGCAAAGTTTGCTGAGCACACAGTGATAAAGATGGCAAGAAGGTCCTGAG

AGAAACGCAATATTTTTGTGCAGAATGGGATGTTCTGCCTTTGTCCCATG

CTTTGAAATTTACCACATGAAAAATAAAATTAAATACTGATCATCAGTGT

TATGTCTGGATCTGGCCACTGCTTAGGACAACTTCGTGCAT

>hg19_ct_ARAlincRNAs_9727_ARAlincRNA_0027.5 range=chr1:95138453-95285837 5'pad=0 3'pad=0 strand=- repeatMasking=none

CTCTGCGCCTGGCCCGGCGACTGCGGGCGCTGCGGTCCACTGGGTCCGCC

TCTTCCCGGCATGGGTTTCTGCCCAGCTCACCCGCTGTCAGCTGGGGTCC

TGCTCTGGTGGGAGGAAGAGGCTCAGACGCTTCCCTGCCCTCTCGCCTCA

ACCACCTCGAGGCAGCGGCTCCCAGGATGTGCACTTTGACGACTAAAGCT

GAGCCGGCGCCGCCACGACCTTGGGCGGGTGGTCGGCCTCTGCCCTGAGC

AGGAAGTAGAAAGTCTCAGCAGACCCTTCCTGAGGGCCGAGCAACAGTGT

AGTGGCGTATTCCACATAGCAAACAGATGTACACTAGATTTTGATGGCAT

TTTTCCAGGGACCCTGGGAGCATCTGTGTTCTCGTGTTCTTCTTGCCTGT

GAGGCCTCTTCTCTCTGAGGACAGATGGTAGGACAGAGAGAAGACAGCAA

ATGGAAAACAAAGGCTGCTATTTTTTTGTGGGGCAGCATTTCTTCCTGTG

ACAATATGGTGTAAGACAAAATTGTGGCCTGCCATGAAGTTGTGAGAGAA

AAGCATCCCTCATTTCTTCAGGAAAGGCCACTGCCGTTCCCTCCGGCTCC

TCTTCTCAGGAGTTTGTGCATTCATGAGGTAAGTGACAGAGTCACTCTCA

AGCTCCTGCTGTATAAAATTTCTGTAACAAATAGATGCTTGTCCCAGATG

GCTCCAGCCAGGCTGATGGGGAGGATGTGGGTTCCCTAGCACTTCCCAGT

TTGCAGCATAAGTGTATTAAAACTTCCATGTCTTACTTAAAATAGAACTC

CTAAAGGCACACACATAACTTCAAGTTTTCTCAGCATTTCATGACTTTTT

TTAAAAAAAGCTTGGACCAATTCAGTTTTCACTCTTGCAAACAGGGTATA

AAAGCTTCTGTTTCTTCCCCAATGTCAACGCTTGGTAATCTTATTCTTCG

TGAATTCCTTGGAAGATGAGGGCCAAGTCTTATTCTTCTCAGTCTCTTCT

GCATCTAGCAGAAGAATAATGGTTGCACAGAAAATCTTGGTTCAGTGGTT

GAGTGAATGAATGGATGAACAGGCTCTTGCACACAAAGAAGAGACATGAT

GTATTTGCATCCTTCCCTGCTTCAGCCAGGTAAGATCTACTAGCTCAAAC

TTATCACAGCCCTGTTTCTGAGCTGGTCCCACGAATAGAGTTTTTGTTTT

TCACCAGCAAGCAGGCTGTTTCCAAGCTGGGATGATAATTGTTGATGTGG

CACTTGCTAAGCTCCTACATCCTGTTCTGGAAGGACAATCCTGAGCACAC

GATGAGCCATATAAACTTCAGACTGACGCTGAGTGAAAGAATGCTGGAAA

AGCATCACAGGCCAGGGCAGCAAAGTCTTCAAGGTCATCCGTGCTCTGAT

GATGTCACGCTTCTTCCCTGTCTGGAAGACATTTCCCCAAGAGCATACCA

CCAACATCAGGGAAATGGAATCCAACTGGTCGCTGCAAAGTTTGCTGAGC

ACACAGTGATAAAGATGGCAAGAAGGTCCTGAGAGAAACGCAATATTTTT

GTGCAGAATGGGATGTTCTGCCTTTGTCCCATGCTTTGAAATTTACCACA

TGAAAAATAAAATTAAATACTGATCATCA

>hg19_ct_ARAlincRNAs_9727_ARAlincRNA_0027.1 range=chr1:95138474-95285837 5'pad=0 3'pad=0 strand=- repeatMasking=none

CTCTGCGCCTGGCCCGGCGACTGCGGGCGCTGCGGTCCACTGGGTCCGCC

TCTTCCCGGCATGGGTTTCTGCCCAGCTCACCCGCTGTCAGCTGGGGTCC

TGCTCTGGTGGGAGGAAGAGGCTCAGACGCTTCCCTGCCCTCTCGCCTCA

ACCACCTCGAGGCAGCGGCTCCCAGGATGTGCACTTTGACGACTAAAGCT

GAGCCGGCGCCGCCACGACCTTGGGCGGGTGGTCGGCCTCTGCCCTGAGC

AGGAAGTAGAAAGTCTCAGCAGACCCTTCCTGAGGGCCGAGCAACAGTGT

AGTGGCGTATTCCACATAGCAAACAGGTGAATTCCTTGGAAGATGAGGGC

CAAGTCTTATTCTTCTCAGTCTCTTCTGCATCTAGCAGAAGAATAATGGT

TGCACAGAAAATCTTGGTTCAGTGGTTGAGTGAATGAATGGATGAACAGG

CTCTTGCACACAAAGAAGAGACATGATGTATTTGCATCCTTCCCTGCTTC

AGCCAGTTTTCTGAAGCTCAGAGGGACACCTTGTATTGCTGGATGATAAA

AACAGGAGCAAAGTGATGAAGTGCTGACAAGGCAACAATAGAACATGAGA

GATTCACTGCTGTGTAGGAAGAGATCTTCGGTGACCATGTAGCCTGAAGC

TCTCATTTTGTCATCGAGGGACACCAAAACTCAGAACAGTTGTCACCCTC

TAAACATTGCAGTGCTAAGCTCCTACATCCTGTTCTGGAAGGACAATCCT

GAGCACACGATGAGCCATATAAACTTCAGACTGACGCTGAGTGAAAGAAT

GCTGGAAAAGCATCACAGGCCAGGGCAGCAAAGTCTTCAAGGTCATCCGT

GCTCTGATGATGTCACGCTTCTTCCCTGTCTGGAAGACATTTCCCCAAGA

GCATACCACCAACATCAGGGAAATGGAATCCAACTGGTCGCTGCAAAGTT

TGCTGAGCACACAGTGATAAAGATGGCAAGAAGGTCCTGAGAGAAACGCA

ATATTTTTGTGCAGAATGGGATGTTCTGCCTTTGTCCCATGCTTTGAAAT

TTACCACATGAAAAAT

>hg19_ct_ARAlincRNAs_9727_ARAlincRNA_0027.2 range=chr1:95138685-95285775 5'pad=0 3'pad=0 strand=- repeatMasking=none

GGGTTTCTGCCCAGCTCACCCGCTGTCAGCTGGGGTCCTGCTCTGGTGGG

AGGAAGAGGCTCAGACGCTTCCCTGCCCTCTCGCCTCAACCACCTCGAGG

CAGCGGCTCCCAGGATGTGCACTTTGACGACTAAAGCTGAGCCGGCGCCG

CCACGACCTTGGGCGGGTGGTCGGCCTCTGCCCTGAGCAGGAAGTAGAAA

GTCTCAGCAGACCCTTCCTGAGGGCCGAGCAACAGTGTAGTGGCGTATTC

CACATAGCAAACAGGTCCCCAGACAGTCCCGGTGTGCACACTTCCCTGCT

TCTGTTCATGATGTTCTCTGAACCTGGGATCACCTTTCCCCTTCCTCTTA

CCTGCCCGATCCTACTTGTCCTCCCAGATTCAGTTTTCTGAAGCTCAGAG

GGACACCTTGTATTGCTGGATGATAAAAACAGGAGCAAAGTGATGAAGTG

CTGACAAGGCAACAATAGAACATGAGAGATTCACTGCTGTGTAGGAAGAG

ATCTTCGGTGACCATGTAGCCTGAAGCTCTCATTTTGTCATCGAGGGACA

CCAAAACTCAGAACAGATTCTGTGGTGAGCTTATGGCACTGAAATGGTGT

GACAAGAAGGAGGTGACAATGTTGTCAACATTCCACAATGATACTGTGAT

TGAAGTAAACAACAGAAATGGAAAGAAGACTAAGAAGCCATGTGTCATTT

TGGATTATCACGAGAATGTGGGAACAGTGGACTGGCTGATCAGATGCTCA

CTTCCTATCCAACTGAGCACAAAAGGCACAAGGTTTCGTCTAAGAAATTC

TTGCTAAGCTCCTACATCCTGTTCTGGAAGGACAATCCTGAGCACACGAT

GAGCCATATAAACTTCAGACTGACGCTGAGTGAAAGAATGCTGGAAAAGC

ATCACAGGCCAGGGCAGCAAAGTCTTCAAGGTCATCCGTGCTCT

>hg19_ct_ARAlincRNAs_9727_ARAlincRNA_0027.3 range=chr1:95144981-95285766 5'pad=0 3'pad=0 strand=- repeatMasking=none

CCCAGCTCACCCGCTGTCAGCTGGGGTCCTGCTCTGGTGGGAGGAAGAGG

CTCAGACGCTTCCCTGCCCTCTCGCCTCAACCACCTCGAGGCAGCGGCTC

CCAGGATGTGCACTTTGACGACTAAAGCTGAGCCGGCGCCGCCACGACCT

TGGGCGGGTGGTCGGCCTCTGCCCTGAGCAGGAAGTAGAAAGTCTCAGCA

GACCCTTCCTGAGGGCCGAGCAACAGTGTAGTGGCGTATTCCACATAGCA

AACAGTTTTCTGAAGCTCAGAGGGACACCTTGTATTGCTGGATGATAAAA

ACAGGAGCAAAGTGATGAAGTGCTGACAAGGCAACAATAGAACATGAGAG

ATTCACTGCTGTGTAGGAAGAGATCTTCGGTGACCATGTAGCCTGAAGCT

CTCATTTTGTCATCGAGGGACACCAAAACTCAGAACAG

>hg19_ct_ARAlincRNAs_9727_ARAlincRNA_0027.11 range=chr1:95170995-95197993 5'pad=0 3'pad=0 strand=- repeatMasking=none

GTGAATTCCTTGGAAGATGAGGGCCAAGTCTTATTCTTCTCAGTCTCTTC

TGCATCTAGCAGAAGAATAATGGTTGCACAGAAAATCTTGGTTCAGTGGT

TGAGTGAATGAATGGATGAACAGGCTCTTGCACACAAAGAAGAGACATGA

TGTATTTGCATCCTTCCCTGCTTCAGCCAGGTCCCCAGACAGTCCCGGTG

TGCACACTTCCCTGCTTCTGTTCATGATGTTCTCTGAACCTGGGATCACC

TTTCCCCTTCCTCTTACCTGCCCGATCCTACTTGTCCTCCCAGATTCAG

>hg19_ct_ARAlincRNAs_9727_ARAlincRNA_0027.4 range=chr1:95170995-95207970 5'pad=0 3'pad=0 strand=- repeatMasking=none

GCAGCATTTCTTCCTGTGACAATATGGTGTAAGACAAAATTGTGGCCTGC

CATGAAGTTGTGAGAGAAAAGCATCCCTCATTTCTTCAGGAAAGGCCACT

GCCGTTCCCTCCGGCTCCTCTTCTCAGGAGTTTGTGCATTCATGAGGTGA

ATTCCTTGGAAGATGAGGGCCAAGTCTTATTCTTCTCAGTCTCTTCTGCA

TCTAGCAGAAGAATAATGGTTGCACAGAAAATCTTGGTTCAGTGGTTGAG

TGAATGAATGGATGAACAGGCTCTTGCACACAAAGAAGAGACATGATGTA

TTTGCATCCTTCCCTGCTTCAGCCAGGTCCCCAGACAGTCCCGGTGTGCA

CACTTCCCTGCTTCTGTTCATGATGTTCTCTGAACCTGGGATCACCTTTC

CCCTTCCTCTTACCTGCCCGATCCTACTTGTCCTCCCAGATTCAG

>hg19_ct_ARAlincRNAs_9727_ARAlincRNA_0027.7 range=chr1:95207825-95285652 5'pad=0 3'pad=0 strand=- repeatMasking=none

TTGACGACTAAAGCTGAGCCGGCGCCGCCACGACCTTGGGCGGGTGGTCG

GCCTCTGCCCTGAGCAGGAAGTAGAAAGTCTCAGCAGACCCTTCCTGAGG

GCCGAGCAACAGTGTAGTGGCGTATTCCACATAGCAAACAGGCAGCATTT

CTTCCTGTGACAATATGGTGTAAGACAAAATTGTGGCCTGCCATGAAGTT

GTGAGAGAAAAGCATCCCTCATTTCTTCAGGAAAGGCCACTGCCGTTCCC

TCCGGCTCCTCTTCTCAGGAGTTTGTGCATTCATGAG

>hg19_ct_ARAlincRNAs_9727_ARAlincRNA_0027.12 range=chr1:95207825-95285837 5'pad=0 3'pad=0 strand=- repeatMasking=none

CTCTGCGCCTGGCCCGGCGACTGCGGGCGCTGCGGTCCACTGGGTCCGCC

TCTTCCCGGCATGGGTTTCTGCCCAGCTCACCCGCTGTCAGCTGGGGTCC

TGCTCTGGTGGGAGGAAGAGGCTCAGACGCTTCCCTGCCCTCTCGCCTCA

ACCACCTCGAGGCAGCGGCTCCCAGGATGTGCACTTTGACGACTAAAGCT

GAGCCGGCGCCGCCACGACCTTGGGCGGGTGGTCGGCCTCTGCCCTGAGC

AGGAAGTAGAAAGTCTCAGCAGACCCTTCCTGAGGGCCGAGCAACAGTGT

AGTGGCGTATTCCACATAGCAAACAGATGTACACTAGATTTTGATGGCAT

TTTTCCAGGGACCCTGGGAGCATCTGTGTTCTCGTGTTCTTCTTGCCTGT

GAGGCCTCTTCTCTCTGAGGACAGATGGTAGGACAGAGAGAAGACAGCAA

ATGGAAAACAAAGGCTGCTATTTTTTTGTGGGAAAAGAGGATCCCCTCCA

GGGATCCATGGTCTCATTGGGATTTTCCTGTTTGGTCTGTGTCCTGTTCT

TGAGTCTCCGCAGGCAGCATTTCTTCCTGTGACAATATGGTGTAAGACAA

AATTGTGGCCTGCCATGAAGTTGTGAGAGAAAAGCATCCCTCATTTCTTC

AGGAAAGGCCACTGCCGTTCCCTCCGGCTCCTCTTCTCAGGAGTTTGTGC

ATTCATGAG

>hg19_ct_ARAlincRNAs_9727_ARAlincRNA_0028.1 range=chr1:100719458-100729383 5'pad=0 3'pad=0 strand=+ repeatMasking=none

AGTGACGTTTACCAAAATGCAAGATGCTAATTAATGAAGGTCTTAAATTA

CTTGTTTGGCTCCTTGGTGTGCCTGACTGGTATTCAACTTCTTCCTGGTT

TAGTCTTGGGAGAGTGTATGTGTCGAGGAATTTATCCATTTCTTCTAGAT

TCCTTTTGGGTTTTCTATGTAGACAGTCTCATCATTAAGATGATATCAAA

ATTAAGGTGTTGTTTTTGCCTGTCCTATCTTATGTCTTTTTTCTTTCTTG

TAAAATCTGTCTAGGACTTCTACCATCTGTTTAAAAAAAGCCATAATAGT

GGGCATTCTTATCTTGTTAATAATATTAAAAGGAAGGAGCCTAATATTTT

ACTACTAAACATGATGTTTGCTTGACTCCCAGTAGGGCCGAAAAAGAAAA

TGAAGTTTGCTGTTGAATTTTTTAGATGCCATTTGTTATGGTTTGGCTCT

GTGTCCCCACCCAAATCTCATGTCAAGTTGTAATCCCCAAGTGTCAGGGG

AGGGACCTGGTAGGAGGTGATTGGATCATGGAGAAATCCCTTGCTGTTCT

TGTGATAGTGTGTTCTCACAAGATCTGGTTGTTGAAAAGTGTGTGGCACT

TAAAAGTGTGTACCTCGCCCCCATCTCCTCTCTCTTTCTCTCCTGCCACC

ATGTAAGTACATGCCTTGCTTCCCCTTTGCCTTCTGCTATGATTATAAGT

TTTCTGAGGCCTCCCCAGCCATGCAGAACTGTGAGTGAATGAAATCTCTT

TTCTTTATAAATTACCCAGTTTCAGGTAGTTCTTTATAGCCATGTGAAAT

GGACTAATATACCATTAATAACACTAAGAGAGTTTTATCCCTAGCTTTGC

TTGATTTTGTTTCATCATAAATGGATCTTTTTTTTTTTCCAATCCTTTTC

CTGCATCTATTGGTGTGATCTTAGGTTTTTGATACATCAGCATATTTTTC

CAATTTTAAATTGATCTTCAATCCCAACTTGGTTATAATGTGTTGTATTT

TACAAACATTGTTGAATTTGATTGTAAATATTTTGACTGAGACTGGTGCA

TCTAAATTATAAATAAAGTTGACCTATAATTTTTCTTCCCCAAGTTGTCT

TTGGTTTTGGCATCAAGATCATACTAGCCTTATAAAATGTACTGGGGAGG

TCTATTTCATGAGTTCTGGAGGGAAACATGTATAATAACTGAAGGTTTTA

TATATATATATATCTGTGTATATATATCTGTATATATATCTTATGTATAT

ATATATCTGTATATATATGTACATGGCCAATTATGTATGTATATAATTGC

CCTTAGATCAAGTTTATTAGTTGTTTTATTAGTTGTCTTGTCTTTCCTAA

ATTTTCATTTGCTTGATCTATAAATAAGTGAGCTAAAACTTCACAATATG

GTAGTGGATTCACCAGTTTCTTCAATTTCTGACTTACATACTTTGAAGCT

ATTTATTACACACAAAGGTTTGAATAACTATTTTCCTGGGGAACACAATT

TTTTATTATTTTGTAGTGACTCTTTTTGGTCAATGATAATGCTTTTTGTA

TTAAAGTCTAATTTAATACAAAATACAGGTATACCACGAATATAGGTATA

CCACCTTTCTTTTTGTTGGCATTTATACGGCATCTCTTTTCCATCTTTTT

ATTTTCAACTCCTCACGATCCTTAATGTTTTTGGTGCATCTCTTGTAAAT

GGCACTTTTTTTTTTCAACCAATCTCTGGCTTTTTTATTTTTTAAACTTG

TGGGTATGTGTCATCCAACAATCTCTGGCTTTTCATCAGAAAGTTTATTT

ACTTTCATTGTTACTATTGATATATATGAATTTATTTCTATTTTGTTACT

TTAGTGCTTTCTTTTCATCCTCTTTTTTTTTAATGCTCATTATGATATTA

GGTTTTTGTTCATTTAATCTGTGTTTTCCTGTTAGTCCATTTTTTCCTTT

GGCTGGATTAGAAGTTATACACTTTCATGGTTATCCTTGAAACTTTACCT

TGCGTACTTAAAGACTTGAATTATGTCAACTCTATTAATTGATATCTTAA

CTACAAAGAGAGACCTTAGAATTCTTTAACTTTGTCATCCACCTCTCGTC

TTACATACTTTTGTTGTCTGGTGTTATTTCATGTAATGTTTGCTCAGATT

CAACTATGTGTTTACAATTTCTTTGCCCATCACTTCTATCTCAGACTCTC

CTTCTGAAGTAATTTTTCTTTCTGAAGTACATCTTTTAGAACTTCCTTTA

AAAAGTGTAGGTAGATGTTAAATTCAGTTTCCATGTACCTGGAAATTATT

TTATTTCTATACTTTAATTCTTGAAAGATAATTTTGCTGAGTGCCCAATT

CTAGGTTGATGGTTATTTTCTGCCAGCATTCTGAATGTACAATTCTACTC

TTTCTGACTTCCACTGTTTTATTGAGGAATCAATTATTGGTCTGTCATTT

GTCATTAAAATAACCAAGGATTTAACCCAACTCTGCCACTTTATTGTTCT

GTCATCTTGGCAGGTCACTTCCAATTCCCTAGGCCTCAGTCCTTCATTAA

CCAAAGAGAGATAAGAGCACCAACCTCACAGCTTTGTTATGAACATTAAG

AGAGATCATGTATATGAAAGCACTACACAAGTGCTTATTATTACCACAAT

TAGCTCATCTCTACATTATATTACTGTCTTGTTCTTTTCCCCCTACCACT

GGAAATAGTTAGAATGGGGCAAATGGGTCATAGCTAGGAATTCATAGCCA

AAATTAGAAATAAAAAGGCGAGGCTTCTTCTGGGAATGTTAAGTTTCTGT

AATAAGAATCTAAGATGTGTTGCGGGAAGTCAGGGACCCCGAACGGAGGG

ACCGGCTGGAGCCACGGCAGAGGAACA

>hg19_ct_ARAlincRNAs_9727_ARAlincRNA_0029.4 range=chr1:117568104-117569408 5'pad=0 3'pad=0 strand=- repeatMasking=none

GTTTATCTTTTAAAAACCGTATACCTGTTTTACAAGGATCTCACAAAGAT

TAACAATATGTATGCTATGAAGGATCCCATCTGCCTCAGAGAAGATACGC

TTTTCATCACAGACTGTATACTTTTTACAGTTCTTATCTATTTATTCTAC

TGCAAAAACTGCCGATTCTACAGAGCCTATTAATTCCACTGGGAAAACAT

GTACGGAATGTGCTCATGAGTATGGGACTTCCTGTAATTGTTGGAAAGAA

GAAAAAGGCTGATAGAAAAAACATTTTGGCCTGGCACTCAGGAGAAAACT

TCTGCTGAGGAATGAACAGGGAAAAACAATGATGGACCACCTCAAGGAAC

AAATCGGATAGCTTCGACTTCCCCTCTGGAAGCCCTGCTGCGCTGGGATG

CACCTATCCCTACTTCTTAGATGTTCCCATATGTTCTCAACAGCATTCTG

TCCCTCCTAAGCAGTGTCACTAAAGCTAGCTGGTCTCTTAGGATTGTTAC

CTTTATTGATGATTTTCCCTTTGTGCTTCCTGGTGTCTGGCACCAAGACC

ACACCCCTACTTCTCCAATGGCCCTTTCCCTCAGTAAGCTTAGCTTATTG

CTCACCTCCCACCTTCTCCCTGAACCCCAGCAGAAAGACATGGACCTGTC

AGATGCTCCCGATATTTAAGTGTCTATGCCAAAATCTCATGTGGACCTTG

TTTGTTCAAATGAGTTACTATTGAGAGAGACAGCAAAGTCATCTTCTTTA

GGGAACCTGAAAATTGTCTTCAGTCTGAAAATTGTCTTCAGAAGGAGCAA

AAGATAGGTGAGAGGGAGAGGATAAGAGGAGAGCCATGTATTCTCCTGAC

CATGAGCTCGCGGTAGATAAACCCCTGGTTACCTGAAGGCAGCACCGTGA

GCACCATCCGCTGTGACTCATCGGATGCTTGATTAATCCACTTGCTTGGG

TGTCCATGGAGCTGCCACTCTGCCACCCTACACCAGTACATTCCTGCATC

CTCCATCTCCACCTGATGAAGCTTCAGGACAAAGTCTGTAGAGGATGAAC

GGTAACAGTGCAGGTGCCTCCTGAGCCCCTCTTCCCCATACTCCAGCAAG

CCATCATGTTGCAAGTGCACCAGCAATTTACTTCCAGAGTTTTCTCTGTT

CCAGTACCACATCACAGAGTACAGAGTGGCTGAGCTGCCTACACTCTCCA

GGCTGCAGCGGATGGCCACTTCTCTGTGCTCAGTCACATTTTCGGTCCAG

TACACTTTGGAGACACGTACCTTACTTCCTAGAAAATAAAATGGCAGTAA

ATTAG

>hg19_ct_ARAlincRNAs_9727_ARAlincRNA_0029.3 range=chr1:117568104-117575606 5'pad=0 3'pad=0 strand=- repeatMasking=none

TGAATTAAATTGGATTGACTGCAACCACATGGGCAAAAGATACTATATAA

AGGCATGCTGAAGCATATGATCATCCCGCAGCTGCAGTCCGCCGGGAAGC

ACTATCAATCAGCAGATACACACGCAGAGCACGCACAAGCAGCCCCAAAG

TGGCTCCTGGCCACACGGCCAGGAAAGCAAAGGCCCAGAGGACTGCAAAC

ACCACAGGTTTCAGGAAACAGTCTATTGAAGCAGAAAGACACATGCTTAA

AGAGACCAATTACTAAATGTAGTCTTCCAATTACATTTATTTATGACATG

ATATCTGAAATCAGGGCTGTTCTGAAATATTCAATGCTAACTGTAGCTGT

GCTTTTACTGGAAAATTGTAATGACATAAGCAATTTCTTCTTCAAAGGTT

AAGCTACAGGATTCATGGGATTATGGATGCTTAGAGATGAAAGAGGTTTA

GAGGTCATTTTGACCAACCTCCTGCTGCAAGAATTCCATTTTTGGCATTG

CCGACGAATCCTACATGTTAAAGAACATCTCTTTGATGATAGTCAAGAAA

GAATCTAGACATTCGCTCACCTCCTGCACTGTGAGCTCCTTGAGGGTTTA

TCTTTTAAAAACCGTATACCTGTTTTACAAGGATCTCACAAAGATTAACA

ATATGTATGCTATGAAGGATCCCATCTGCCTCAGAGAAGATACGCTTTTC

ATCACAGACTGTATACTTTTTACAGTTCTTATCTATTTATTCTACTGCAA

AAACTGCCGATTCTACAGAGCCTATTAATTCCACTGGGAAAACATGTACG

GAATGTGCTCATGAGTATGGGACTTCCTGTAATTGTTGGAAAGAAGAAAA

AGGCTGATAGAAAAAACATTTTGGCCTGGCACTCAGGAGAAAACTTCTGC

TGAGGAATGAACAGGGAAAAACAATGATGGACCACCTCAAGGAACAAATC

GGATAGCTTCGACTTCCCCTCTGGAAGCCCTGCTGCGCTGGGATGCACCT

ATCCCTACTTCTTAGATGTTCCCATATGTTCTCAACAGCATTCTGTCCCT

CCTAAGCAGTGTCACTAAAGCTAGCTGGTCTCTTAGGATTGTTACCTTTA

TTGATGATTTTCCCTTTGTGCTTCCTGGTGTCTGGCACCAAGACCACACC

CCTACTTCTCCAATGGCCCTTTCCCTCAGTAAGCTTAGCTTATTGCTCAC

CTCCCACCTTCTCCCTGAACCCCAGCAGAAAGACATGGACCTGTCAGATG

CTCCCGATATTTAAGTGTCTATGCCAAAATCTCATGTGGACCTTGTTTGT

TCAAATGAGTTACTATTGAGAGAGACAGCAAAGTCATCTTCTTTAGGGAA

CCTGAAAATTGTCTTCAGTCTGAAAATTGTCTTCAGAAGGAGCAAAAGAT

AGGTGAGAGGGAGAGGATAAGAGGAGAGCCATGTATTCTCCTGACCATGA

GCTCGCGGTAGATAAACCCCTGGTTACCTGAAGGCAGCACCGTGAGCACC

ATCCGCTGTGACTCATCGGATGCTTGATTAATCCACTTGCTTGGGTGTCC

ATGGAGCTGCCACTCTGCCACCCTACACCAGTACATTCCTGCATCCTCCA

TCTCCACCTGATGAAGCTTCAGGACAAAGTCTGTAGAGGATGAACGGTAA

CAGTGCAGGTGCCTCCTGAGCCCCTCTTCCCCATACTCCAGCAAGCCATC

ATGTTGCAAGTGCACCAGCAATTTACTTCCAGAGTTTTCTCTGTTCCAGT

ACCACATCACAGAGTACAGAGTGGCTGAGCTGCCTACACTCTCCAGGCTG

CAGCGGATGGCCACTTCTCTGTGCTCAGTCACATTTTCGGTCCAGTACAC

TTTGGAGACACGTACCTTACTTCCTAGAAAATAAAATGGCAGTAAATTAG

>hg19_ct_ARAlincRNAs_9727_ARAlincRNA_0029.2 range=chr1:117568919-117602112 5'pad=0 3'pad=0 strand=- repeatMasking=none

TTGGCCCGCGCTGCTGCTTCCTGTGTGTACTGGATGTTCTCTAGGCTCGC

CCAGCGTGGAAGCCCCGAGTTACCAGCCGCAACGTGGAAACCACAGAAGG

GGAAATAGATGACTTTTGGTTATCCAGATCTCTTTGAATCTTGTTCCTGT

CAACTGACTTATTAGTGCCATCTCCTAATTCTGTGTCAACTTGACAAGCC

TGCTGTCTTTCCCTTCATCCAAGTTGCCAATAAAAATGCTCAACAAGGCA

GAGTGCTTTAGCAAGACCTGGAGAGATCTCCTACAGCCAACCTCCTGCTG

CAAGAATTCCATTTTTGGCATTGCCGACGAATCCTACATGTTAAAGAACA

TCTCTTTGATGATAGTCAAGAAAGAATCTAGACATTCGCTCACCTCCTGC

ACTGTGAGCTCCTTGAGGGTTTATCTTTTAAAAACCGTATACCTGTTTTA

CAAGGATCTCACAAAGATTAACAATATGTATGCTATGAAGGATCCCATCT

GCCTCAGAGAAGATACGCTTTTCATCACAGACTGTATACTTTTTACAGTT

CTTATCTATTTATTCTACTGCAAAAACTGCCGATTCTACAGAGCCTATTA

ATTCCACTGGGAAAACATGTACGGAATGTGCTCATGAGTATGGGACTTCC

TGTAATTGTTGGAAAGAAGAAAAAGGCTGATAGAAAAAACATTTTGGCCT

GGCACTCAGGAGAAAACTTCTGCTGAGGAATGAACAGGGAAAAACAATGA

TGGACCACCTCAAGGAACAAATCGGATAGCTTCGACTTCCCCTCTGGAAG

CCCTGCTGCGCTGGGATGCACCTATCCCTACTTCTTAGATGTTCCCATAT

GTTCTCAACAGCATTCTGTCCCTCCTAAGCAGTGTCACTAAAGCTAGCTG

GTCTCTTA

>hg19_ct_ARAlincRNAs_9727_ARAlincRNA_0029.1 range=chr1:117574736-117593278 5'pad=0 3'pad=0 strand=- repeatMasking=none

TGGGGTTTCGCCAGTATTTTTTGATGAATGTATGCTTAATTTGAGGCATC

CCAAAGAAAATTCAATATGAAAAATGCCGTGGGATAATTATCTCCTTTAT

TTTGGATATGACCATTTTATATATGAAATGGAAAACTCTAAATCTTTTTC

TCTGTTTTTTCAATAAGTATGTTATGCTAATAATATATATCAAGTGTGCA

ATCAACCAAAACACTGTTTTCATTTTTCATGCGTGAGCTCATCTCCCCTA

TCTGGTGCATCTAATTGCCTAAACTTAACTATGGATTCTATATTTCATGT

TAATTTTGATTCATTATTCTAGGTTATCCAGATCTCTTTGAATCTTGTTC

CTGTCAACTGACTTATTAGTGCCATCTCCTAATTCTGTGTCAACTTGACA

AGCCTGCTGTCTTTCCCTTCATCCAAGTTGCCAATAAAAATGCTCAACAA

GGCAGAGTGCTTTAGCAAGACCTGGAGAGATCTCCTACAGGTAAACACGT

TGCCATGGTCTTTACACCTTGGGTATGATCATTCACCCAAGCATGGAGTC

ACATAGTGTCCACGTTGTTCCTCTTGTTCAAAGGCCTTGCTGCAGTTCAT

GCATGCGGAATCTATAGCTGTCCCATGACTTACCAGCCTAGCAGAAAGCA

GCACATCAGAAAAGGAAGTGAGTTTAACTTGGCATGTCATTTTCTTTCTT

TCTTTTCTTTTTTTTTTTTGAGACAGAATCTCGCGGTCTCAATGGAGCCT

GATGAATGAATCAATTGCATGAGTACATTCCACACATGGGAACCCAGGGA

TGGCTGCCATCATGCTATACAAAGTACAGATCATCACTGAACATTAGGCT

GGCAGCTCTGAATGGGGCTGGCAAAGAGCAGCAGAGGTTCATGGCCTGGA

TTTTCTCTGATCTCATTTGTATCCTTCATCCTAAATTTAGTTTTGTTTTT

TGAAATG

>hg19_ct_ARAlincRNAs_9727_ARAlincRNA_0030.1 range=chr1:120388521-120389383 5'pad=0 3'pad=0 strand=+ repeatMasking=none

TGTGACCCTGGGCTGCTCAGCATTCATGTGGAAATGAAGGAAGGAGGACT

GGATCAATCCCATTTGAAAGCATCCTTCTCTGCAGCCCACACAATCCTCC

AATGACACTGACAGAGGTGTACCCTCCTAGTGTACTGGGACCAAAATTCG

GAAGTGTCTGCAAACTTGCTTTAACCGTATGGGAAATAACCTCTATCACC

TGGAATTTCCTTGGGACTTTGGAAAATACAAGAGAAGTATGAGACTTGGG

TCTTCCCTTGGCTGTGTTTAATTCACTCTTCTATGGAATACCAATGATTC

TCACTAAGACTTTTGCCTTTTTATAACCACAATGTATGCTTTATGGAG

>hg19_ct_ARAlincRNAs_9727_ARAlincRNA_0031.1 range=chr1:142884065-142887263 5'pad=0 3'pad=0 strand=+ repeatMasking=none

TTTCATTCAGTTGTCATTTTCAGTGCATGTTTTCTGATTGTATAAAAGCT

CTTCCATGCGAGAGAGTTGATGTTAAAACAGTAGATTATTACCCTGAAGT

GTATTTTCACCAGGTAAAGAAAGCCTTTTATGGTTCACTGAGGACAGTCA

AACCCTTCAAAATCTAGAATCTGATGACTGGATCTTCTGAGAACATCAGA

GAAGGACATTGTTAAAGTGAACTAAGTATGGCCTGAGAAGGACTCCATAA

TTCTATATATGAGTCCTTGTGGGTGAACTGCAACCTACCTTAATAGGTAT

ACAAGAATGAAAAACTAACTTAAGAGTATGCACCTGGAACAACAGCTACA

TCTTGGCCAATCCCAATGGCCAAACTTCAACCACTCAGGCACTGCCAAAT

GTTCAAAATGTGTTCAAACAAGGCAAACGCTGAGTTGTTTCTGTACCTCA

CTTCCGATTTCGGTATGCCACTTCCCTTTTGTCTATAAATCTTCTTCCAC

CACATGACTGCACTGGAGTCTCTGTGAATCTGCTGTGATTCTGGGGACTG

TCCGATTCATGAATCGCTTTATTGCTAAATTAAACTCCTTTAAAGTTTT

>hg19_ct_ARAlincRNAs_9727_ARAlincRNA_0032.1 range=chr1:143255459-143255760 5'pad=0 3'pad=0 strand=- repeatMasking=none

AGATTTCATTAAATGTAGGAGACATAAAATATAAGAATCATCACTTTGCA

AATATCATAGTAATAATTGTTGCAAGAAAGAACCTTGGAGGGATGCTAAG

ATTAGTGGTGAATATATGTTGGGAAAGAACATATTTGCATAATATGAAAG

TATCTTCCCACAAGATAATTATAGAATAGTAACTTCAAAGTGGAGATGTC

AACTTAGCCAAGTGATCAAAGTTAACATTACTAATAATAAGATAAATGAA

CTTCATGTAACTTCTTATATGATGCACTGAGTAGGACAAAACATCAATTC

TA

>hg19_ct_ARAlincRNAs_9727_ARAlincRNA_0033.10 range=chr1:149239531-149245285 5'pad=0 3'pad=0 strand=+ repeatMasking=none

ATTCCTATCAGAGCCTGAGTCACAGACAGTACCTTAGACTCTAAGCTTAG

GCACCTTGTGCCTTGTTTTTGTGGCTAAGGATTCTCAGAGTTGTAGGATG

CTGACAAAGTTTATCCCAAAGGCTCTGATTGTACTTTCTCCACAAGCTTC

AGCATGGCAGTATCTCTCACCACTTGTTTTGGTCCAAGGGCAGAAATGAG

CATCCTGGAAACCAATCAGTACTTGCTCTCTGAACTGGAAAAGTGCAAAG

AGAACTTCCGAGACCTCACAGAGAAATTCCTGACATCCAAAGCTACTGCC

TACTCCCTGGCCAATCACCTGCAGAAATATAGATACATGATCCCTTAATT

CAGGCTCAGGCTGAAGAACTGACCCACTTACGACAGAAGATACAAGAAGG

GAGAGGTGTCTGCTACCTTTTCACCCAGCATGTGAAGAACACAGTCAAGT

CTTTTGAGGGCCTTCTCAGGAACACTGGCATTGCCTACTACCAGAGACAG

AGATTCTGTGAGCAAATGGTACAAGGAAGCCAGCTAACAGAGATCCTTGT

CAGAAAACTTGCCACAG

>hg19_ct_ARAlincRNAs_9727_ARAlincRNA_0033.7 range=chr1:149239531-149250644 5'pad=0 3'pad=0 strand=+ repeatMasking=none

ATTCCTATCAGAGCCTGAGTCACAGACAGTACCTTAGACTCTAAGCTTAG

GCACCTTGTGCCTTGTTTTTGTGGCTAAGGATTCTCAGAGTTGTAGGATG

CTGACAAAGTTTATCCCAAAGGATACATGATCCCTTAATTCAGGCTCAGG

CTGAAGAACTGACCCACTTACGACAGAAGATACAAGAAGGGAGAGGTGTC

TGCTACCTTTTCACCCAGCATGTGAAGAACACAGTCAAGTCTTTTGAGGG

CCTTCTCAGGAACACTGGCATTGCCTACTACCAGAGACAGAGATTCTGTG

AGCAAATGGTACAAGGAAGCCAGCTAACAGAGATCCTTGTCAGAAAACTT

GCCACAGAATCAACCCCAATGCTCACTAACAAACTGAGAAGAAAAAAAAA

GAATTACCCCCAACAACATCTGAGGAAGCAGAACAGGGACAGACTTGAAG

GCACCTGGGCAGTCCTCTGGTTTTGGAGTAGAG

>hg19_ct_ARAlincRNAs_9727_ARAlincRNA_0033.2 range=chr1:149239531-149250980 5'pad=0 3'pad=0 strand=+ repeatMasking=none

ATTCCTATCAGAGCCTGAGTCACAGACAGTACCTTAGACTCTAAGCTTAG

GCACCTTGTGCCTTGTTTTTGTGGCTAAGGATTCTCAGAGTTGTAGGATG

CTGACAAAGTTTATCCCAAAGGCTCTGATTGTACTTTCTCCACAAGCTTC

AGCATGGCAGTATCTCTCACCACTTGTTTTGGTCCAAGGGCAGAAATGAG

CATCCTGGAAACCAATCAGTACTTGCTCTCTGAACTGGAAAAGTGCAAAG

AGAACTTCCGAGACCTCACAGAGAAATTCCTGACATCCAAAGCTACTGCC

TACTCCCTGGCCAATCACCTGCAGAAATATAAGTGTGAAGAGTGCAAAGA

CCTTATAGAATCTGTGCTGGAGGAGGAGCTGCAGTTTCAGGAGAGGGAGC

TGACCAAATTGCCAAGGCCAGCAGCAAGGCTCCGGATACATGATCCCTTA

ATTCAGGCTCAGGCTGAAGAACTGACCCACTTACGACAGAAGATACAAGA

AGGGAGAGGTGTCTGCTACCTTTTCACCCAGCATGTGAAGAACACAGTCA

AGTCTTTTGAGGGCCTTCTCAGGAACACTGGCATTGCCTACTACCAGAGA

CAGAGATTCTGTGAGCAAATGGTACAAGGAAGCCAGCTAACAGAGATCCT

TGTCAGAAAACTTGCCACAGAATCAACCCCAATGCTCACTAACAAACTGA

GAAGAAAAAAAAAGAATTACCCCCAACAACATCTGAGGAAGCAGAACAGG

GACAGACTTGAAGGCACCTGGGCAGTCCTCTGGTTTTGGAGTAGAGGCAG

CTGACGTCTGGTGATGATTCACCATATCAGGAACACCCTGTCCAGTGGGC

TCAGGCAAGCCTGCCAGGCCACTTCTCTAAAGACACAACCTGAGGCCTCC

TGCAATAATTCTCACAGAGCATTCTGAACATACAT

>hg19_ct_ARAlincRNAs_9727_ARAlincRNA_0033.1 range=chr1:149239531-149251131 5'pad=0 3'pad=0 strand=+ repeatMasking=none

ATTCCTATCAGAGCCTGAGTCACAGACAGTACCTTAGACTCTAAGCTTAG

GCACCTTGTGCCTTGTTTTTGTGGCTAAGGATTCTCAGAGTTGTAGGATG

CTGACAAAGTTTATCCCAAAGGCTCTGATTGTACTTTCTCCACAAGCTTC

AGCATGGCAGTATCTCTCACCACTTGTTTTGGTCCAAGGGCAGAAATGAG

CATCCTGGAAACCAATCAGTACTTGCTCTCTGAACTGGAAAAGTGCAAAG

AGAACTTCCGAGACCTCACAGAGAAATTCCTGACATCCAAAGCTACTGCC

TACTCCCTGGCCAATCACCTGCAGAAATATAGATACATGATCCCTTAATT

CAGGCTCAGGCTGAAGAACTGACCCACTTACGACAGAAGATACAAGAAGG

GAGAGGTGTCTGCTACCTTTTCACCCAGCATGTGAAGAACACAGTCAAGT

CTTTTGAGGGCCTAAAATCACAATGGTAAGAAAATGAAGACAGGCAAAAG

CCACTGGCTCCCAG

>hg19_ct_ARAlincRNAs_9727_ARAlincRNA_0033.5 range=chr1:149239868-149255822 5'pad=0 3'pad=0 strand=+ repeatMasking=none

GCTCTGATTGTACTTTCTCCACAAGCTTCAGCATGGCAGTATCTCTCACC

ACTTGTTTTGGTCCAAGGGCAGAAATGAGCATCCTGGAAACCAATCAGTA

CTTGCTCTCTGAACTGGAAAAGTGCAAAGAGAACTTCCGAGACCTCACAG

AGAAATTCCTGACATCCAAAGCTACTGCCTACTCCCTGGCCAATCACCTG

CAGAAATATAGATACATGATCCCTTAATTCAGGCTCAGGCTGAAGAACTG

ACCCACTTACGACAGAAGATACAAGAAGGGAGAGGTGTCTGCTACCTTTT

CACCCAGCATGTGAAGAACACAGTCAAGTCTTTTGAGGGCCTTCTCAGGA

ACACTGGCATTGCCTACTACCAGAGACAGAGATTCTGTGAGCAAATGGTA

CAAGGAAGCCAGCTAACAGAGATCCTTGTCAGAAAACTTGCCACAGAAAA

TCACAATGGTAAGAAAATGAAGACAGGCAAAAGCCACTGGCTCCCAGGCA

CACTTTACTCCCAGTCAAAAGTGGACTGTATACTTCCTCTTTGATGTGAT

GGTCCCTTTGGTTTTGGTGCCTCTTCCATGAGCTTCAGATCAGAACTAGC

TATGTGGTTCCAGGCTGCCCTGGTTTAATCCCTTGTCTTGCCTACTTCAC

CTGGCTCAAGAGGGAAGTCCAGGAGGAAGAAATGGATGAAGTCCTGGAGG

ACTCACTAGATGAACAGTATTTGACTCATTCCAGCTGCCATGACTCCCAC

CAGCTTCCCAGCAGCAATGCCTTCCTCTTTGATGCACAGGAAGGCCCCTC

GGCTGTGGAAATAGCCAAAGATGAGATCCAGAGCCAGTGGCAGCACCTGA

AAGAGATCCTTTTCATCAACAACTGCCTGTGAGAAAAGCTAGAACATCAT

CACAGCAGCTTTGACGAAGAAAATGGATGCACCTCTAACCTCTACAGGCA

AATCATAGATTCCATTGTCCAGCTGTAAAATGAAAACAGAGTTCTCAGAG

AAGAATACCGGAGACTCCCCCGACTTGCCTGAATCAAATTTCCAGAGGTA

AGTGGTGAAAGGCTACAATATGAAGTCACTCTCAGATGGGTCCCTTCTCT

TGCCACACTCCACTGTAGAAATAAAAGAGGACGGGGAGGCCAGAGCTGCT

CAAAACCTTATAACCCACAATTCTCATAGTCATTCTCAACTTAGAGAGGG

CTGCTTTCTAGACACACCCCCTCCAGGCCCCCAGACTGCCAGGCTCCAGG

AATAAGTAGCTCTAAGAAATACAGTATTTCGATGCGAGCCACACCCCACT

ATTGCAAATTGGAGAAGAGAAGGACAAACTTAAAAGGTGAAAAGATTTAA

CAGAACAACAGGGTGAAACCCCATCTCTAATAAAAATACAAAACTTAGCT

GGGCGTGGTGGCATGCACCTGTAGTCCCAGCTACTCAGGAGGCTGAGGCA

GGAGAATCATTTCAACCCAGGAGGCAGACGTTGCAGCGAGCCAAGTTTGC

ACCACTGCATTCTAGCCTGAGTGACAGAGCATGACTCCATCTCAAAAAAA

AAAGAAAAAAAG

>hg19_ct_ARAlincRNAs_9727_ARAlincRNA_0033.9 range=chr1:149239868-149256853 5'pad=0 3'pad=0 strand=+ repeatMasking=none

GCTCTGATTGTACTTTCTCCACAAGCTTCAGCATGGCAGTATCTCTCACC

ACTTGTTTTGGTCCAAGGGCAGAAATGAGCATCCTGGAAACCAATCAGTA

CTTGCTCTCTGAACTGGAAAAGTGCAAAGAGAACTTCCGAGACCTCACAG

AGAAATTCCTGACATCCAAAGCTACTGCCTACTCCCTGGCCAATCACCTG

CAGAAATATAGATGCACCTCTAACCTCTACAGGCAAATCATAGATTCCAT

TGTCCAGCTGTAAAATGAAAACAGAGTTCTCAGAGAAGAATACCGGAGAC

TCCCCCGACTTGCCTGAATCAAATTTCCAGAGGTAAGTGGTGAAAGGCTA

CAATATGAAGTCACTCTCAGATGGGTCCCTTCTCTTGCCACACTCCACTG

TAGAAATAAAAGAGGACGGGGAGGCCAGAGCTGCTCAAAACCTTATAACC

CACAATTCTCATAGTCATTCTCAACTTAGAGAGGGCTGCTTTCTAGACAC

ACCCCCTCCAGGCCCCCAGACTGCCAGGCTCCAGGAATAAGTAGCTCTAA

GAAATACAGTATTTCGATGCGAGCCACACCCCACTATTGCAAATTGGAGA

AGAGAAGGACAAACTTAAAAGGTGAAAAGATTTAACAGAACAACAGGGTG

AAACCCCATCTCTAATAAAAATACAAAACTTAGCTGGGCGTGGTGGCATG

CACCTGTAGTCCCAGCTACTCAGGAGGCTGAGGCAGGAGAATCATTTCAA

CCCAGGAGGCAGACGTTGCAGCGAGCCAAGTTTGCACCACTGCATTCTAG

CCTGAGTGACAGAGCATGACTCCATCTCAAAAAAAAAAGAAAAAAAGAAA

GACAGAAAAGATTTAAGAGAACAATTATTTTAATTGAATACATTTTGTCA

AGATTAAATCAACTTTCCCCTAGAATACTGAAAGAATAAGCAATCATAAT

TTAGATTCTAAGTTACTGGCCTTTGAGAAAGTGTGAAATCCAAAAAGATG

CCATGAGCCAAAAAAGAGCAAAAGTCCTAATTTTCAAACTGATGAAAATA

AAGTACTAAATAATTGAAATCATCGTAGTTCATCTTGGTCCTTGGCAAAA

TAAAAATTTATTGATTGAAATTATAATATCCTAGCTACCTTTTTGTGCCT

AGCTCTTACCACTGAGGAGAAAAACCAAACACAAACAAAAGTATGTTCAA

ATTGGCAAAAATGTTTACAAACCTGCAAAATCTCTTCAGCCTATCCCTTA

AACTGCAGACTTGGTATTTACTAAGCTCTGCTGTGTACAATACCCACCTT

AATATCCATTCTTGCACAAGTTCTGTTAGGTATAAGCCAAAATCACATGG

GATTTTTTTTTTTACCCTCTCATGCCTATGTTTGCTTTTGCTTCCAAAGA

CCCTTCTGAGTAAGGACTGTCCTCAGACTACTGGAGTCACTTAGTCACAA

GCTTGCCTGAGGCTCAGAGCTGGCAATACCTGGCAGTTTATGTCAATCCC

ACATGCAACCCTGCCTGCAAAGTATTTACCCATGATTGACAGGTGCAGGT

GTATGAAAGCCCAGCTCCTTGACTTGAGGGGTGCGTTTGTGGTGAATTAG

CTTTGGGAAAAGGGAATCCAGAGGCCAACTTTGTAAAAATCCAGATAGGT

AAATGGAGAGAAATCTTTGCCTTGGAAGTCCAATTGAGAAAGATAGTGAT

TTACTTCGGACTGTGAAAGGAGAGACTAAAATCTTTACTTTCTCATGGGA

AAGAAGCCTATGGTGAAAGAGAAACAAAGAAAATGGTGGGAAATAGGGTA

GGTCTACTCAGTAGCTTTAAACCATTCTGTCATATTATAAAGCCTGGTAA

GGTTTTAACTAACACAAGTGCCTGGACT

>hg19_ct_ARAlincRNAs_9727_ARAlincRNA_0033.6 range=chr1:149239868-149265510 5'pad=0 3'pad=0 strand=+ repeatMasking=none

GCTCTGATTGTACTTTCTCCACAAGCTTCAGCATGGCAGTATCTCTCACC

ACTTGTTTTGGTCCAAGGGCAGAAATGAGCATCCTGGAAACCAATCAGTA

CTTGCTCTCTGAACTGGAAAAGTGCAAAGAGAACTTCCGAGACCTCACAG

AGAAATTCCTGACATCCAAAGCTACTGCCTACTCCCTGGCCAATCACCTG

CAGAAATATAAGTCTGCTCTCTAACCCGAGCTAGAGTGCAGTGACGCGAT

CTTGGCTGACTGCAGTCTGTGCCTCCTGGGCTCAAGCAGTCATCCCACCT

CAGCCTCCCAAGTAGCTGGGACTACAGAAAATCACAATGGTAAGAAAATG

AAGACAGGCAAAAGCCACTGGCTCCCAGGCACACTTTACTCCCAGTCAAA

AGTGGACTGTATACTTCCTCTTTGATGTGATGGTCCCTTTGGTTTTGGTG

CCTCTTCCATGAGCTTCAGATCAGAACTAGCTATGTGGTTCCAGGCTGCC

CTGGTTTAATCCCTTGTCTTGCCTACTTCACCTGGCTCAAGAGGGAAGTC

CAGGAGGAAGAAATGGATGAAGTCCTGGAGGACTCACTAGATGAACAGTA

TTTGACTCATTCCAGCTGCCATGACTCCCACCAGCTTCCCAGCAGCAATG

CCTTCCTCTTTGATGCACAGGAAGGCCCCTCGGCTGTGGAAATAGCCAGA

TGCACCTCTAACCTCTACAGGCAAATCATAGATTCCATTGTCCAGCTGTA

AAATGAAAACAGAGTTCTCAGAGAAGAATACCGGAGACTCCCCCGACTTG

CCTGAATCAAATTTCCAGAGGTAAGTGGTGAAAGGCTACAATATGAAGTC

ACTCTCAGATGGGTCCCTTCTCTTGCCACACTCCACTGTAGAAATAAAAG

AGGACGGGGAGGCCAGAGCTGCTCAAAACCTTATAACCCACAATTCTCAT

AGTCATTCTCAACTTAGAGAGGGCTGCTTTCTAGACACACCCCCTCCAGG

CCCCCAGACTGCCAGGCTCCAGGAATAAGTAGCTCTAAGAAATACAGTAT

TTCGATGCGAGCCACACCCCACTATTGCAAATTGGAGAAGAGAAGGACAA

ACTTAAAAGGTGAAAAGATTTAACAGAACAACAGGGTGAAACCCCATCTC

TAATAAAAATACAAAACTTAGCTGGGCGTGGTGGCATGCACCTGTAGTCC

CAGCTACTCAGGAGGCTGAGGCAGGAGAATCATTTCAACCCAGGAGGCAG

ACGTTGCAGCGAGCCAAGTTTGCACCACTGCATTCTAGCCTGAGTGACAG

AGCATGACTCCATCTCAAAAAAAAAAGAAAAAAAGAAAGACAGAAAAGAT

TTAAGAGAACAATTATTTTAATTGAATACATTTTGTCAAGATTAAATCAA

CTTTCCCCTAGAATACTGAAAGAATAAGCAATCATAATTTAGATTCTAAG

TTACTGGCCTTTGAGAAAGTGTGAAATCCAAAAAGATGCCATGAGCCAAA

AAAGAGCAAAAGTCCTAATTTTCAAACTGATGAAAATAAAGTACTAAATA

ATTGAAATCATCGTAGTTCATCTTGGTCCTTGGCAAAATAAAAATTTATT

GATTGAAATTATAATATCCTAGCTACCTTTTTGTGCCTAGCTCTTACCAC

TGAGGAGAAAAACCAAACACAAACAAAAGTATGTTCAAATTGGCAAAAAT

GTTTACAAACCTGCAAAATCTCTTCAGCCTATCCCTTAAACTGCAGACTT

GGTATTTACTAAGCTCTGCTGTGTACAATACCCACCTTAATATCCATTCT

TGCACAAGTTCTGTTAGGTATAAGCCAAAATCACATGGGATTTTTTTTTT

TACCCTCTCATGCCTATGTTTGCTTTTGCTTCCAAAGACCCTTCTGAGTA

AGGACTGTCCTCAGACTACTGGAGTCACTTAGTCACAAGCTTGCCTGAGG

CTCAGAGCTGGCAATACCTGGCAGTTTATGTCAATCCCACATGCAACCCT

GCCTGCAAAGTATTTACCCATGATTGACAGGTGCAGGTGTATGAAAGCCC

AGCTCCTTGACTTGAGGGGTGCGTTTGTGGTGAATTAGCTTTGGGAAAAG

GGAATCCAGAGGCCAACTTTGTAAAAATCCAGATAGGTAAATGGAGAGAA

ATCTTTGCCTTGGAAGTCCAATTGAGAAAGATAGTGATTTACTTCGGACT

GTGAAAGGAGAGACTAAAATCTTTACTTTCTCATGGGAAAGAAGCCTATG

GTGAAAGAGAAACAAAGAAAATGGTGGGAAATAGGGTAGGTCTACTCAGT

AGCTTTAAACCATTCTGTCATATTATAAAGCCTGGTAAGGTTTTAACTAA

CACAAGTGCCTGGACTGCTGAGGAGGAGGAAATACAGGAAGTATTGGTCT

TGCTGTTCAGAGTGGCAGAGGAGAAAGAAAATCCCAGCCGTCTTGGAAAT

TCACAGAGAGAATATGAAAATCAGGTTGTCCTGTACAGTATCTGCAATCA

GTTACGATACAGAAATAACTTAAACATGTCAAGAAAGATGAACGCAGATA

CTATGAGGAACTGCTAAACTACAGCCGAGATCATCTCATGCTGTACCCTT

ACCGTCTATTGGATATTATGGTGAAGAGCTTGAGCATAACACCATTTTCA

TATTACACTGGGATTATGGAGAATATTATGAACAGTGGGCACAATTTTAC

TGCTGCTGACTGTCTAAGGCTTCTTGGCATAGGAAGAAACCAGTATATTG

ATCTTATGATTCAGTGTAGATCATCAAAAAAATTCTTCAGAAGGAAAACA

GCCGGTGATCTTCTACCAATAAAGCCAGTGGAAATTGCCATAGAGGCATG

GTGGGTGGTGCAGGCTGGATATATCACAGAAGATGACATCAAGATATGCA

CTTGGCCTGAGAAATGTGCTACTGATAAGACCGTTGATTCAGGCCCTCAA

CTCTCTGGATCACTAGATTACAATGTAGTACATAGTTTGTATAAAAAAGG

ATTTATTTATCTGGATGTACCAATGTCTGATGACAGTTGTATAGCAGTTG

CACCCCTTGAAGGTTTTGTAATGAATCGAGTGCTTATTTTGATTATTTTG

AAACTCTACTCTATAAGATATTTGTTTCAGTAGATGAGCACACAAATGTG

GCAGAGCTTGCAAATGTCCTTGAGATTGACTTATCCCTGGTTAAGAATGC

TGTTTCAATGTATTGCTGATTGGGCTTTGCCCATAAGAAGGGACAAGTAA

TAAATTTGGATCAACTTCATTCATCATGGAAGAATGTTCCATCCATAAAC

AGATTAAAGAGTACCTTAGATCCATAGAAGATGCTCTTGTCATGGGGTGG

AGGGGAAAGTAGGAGGCCTGTACAAGAAGCTTCATCGGCAACTGACACTG

ATACAAATAGTCAAGAAGATCCAGCTGACACAGCCAGTGTAAGAAGCCTG

AGTCTGTCTGCAGGACACACGAAGCACATCGCATTCCTGTTTGACTCCAC

TCTTACTGCCTTCTTAAAGATGGGAAATCTTTCACCAGTTCAGAGCACTG

GTGAAAGAGAAGCACAGAGATATTTTGATCATGCGCTTACTCTGAGAAAC

ACAATACTGTTTCTGCGTCATAACAAAGATCTAGTTGTGCAAACTGCACA

GCCAGACCAACCCAATTATGGTTTTCCTCTGGATCTCTTACGCTGTGAAA

GCCTTCTTGGTCTGGACCCTGCAACTGGCAGCAGAGTTCTAAACAAAAAT

TACACACTGCTTGTTTCCATGGCTCCCCTCACCAATGAAATCCGGCCTGT

CAGCAGCTGCACCCCTCAGCATATTGGACCAGCTATCCCAGAAGTCAGCT

CTGTCTGGTTTAAACAGTACATTTACGTTTATCATATCACTGGACAAGGA

CCACCATCCCTTTTATTATCCAAAGGTACAAGACCTCGAAAACTGCCAGA

TATATTTCAGAGTTATGATCGATTGCTAATAACATCTTGGTGTCATGATC

CTGGAGTAGTTCCTACCTCAAATGTGCTCACGATGTTGAATGATGCTTTA

ACACATTCTGCAGTTTTAATTCAGAGGCATGGTCTGCATGGGATAGGAGA

AACTGTCCATGTCCCATTTCCATTTGATGAAACAGAACTACAAGGAGAGT

TCACTCGTGTCAATATGGGTGTTCATAAAGCATTGCAGATACTAAGGAAC

AGAGTGGACTTACAACATCTCTGTGGATATGTCACCATGTTGAATGCTTC

CAGCCAACTTGCAAATAGAAAACTCAGTGATGCTTCTGATGAGAGAGGAA

AACCTGATTTGGCTTCTGGCTCAGATGTTAAATGGGAGTACAGAGTCATT

TGAAATGGTCATTGAAGAAGCAACTATAGATTCAGCAACAAAGCAAACCT

CTGGTGCCACAACAGAAGCAGATTGGGTTCCTCTCGAGCTGTGCTTTGGA

ATTCCACTGTTCAGTTCCGAATTAAACCGGAAAGTTTATAGGAAAATTGC

TACACATGGCCTTTGCAGAAAAGAGAGCCTTCAAAACCTCTTACATTCCA

GTAGAAAACTCTCTCTGCAAGTCCTTAACTTTGTTCACTCATTCCAGGAA

GGTGCTTCAACATTGGATATTCACACAGAGCCCAGTTTTTCAAGTTTGCT

TTCACAGTCATCGTATGCTGACATGGGTGTTCCACTTCCTGCAAAAAATT

TAATATTTAAAGATGGTGTCTTATCAGAACGGAGTGGATGGTCACCTTCC

TCACTTATTGCTAATCTCCATTTGCAATAATTTGGTTACACCATTTGCTG

CTCACACTTTCTGCCTTTTTTCTTTCTTAACGTTAGCTTTATAGTGTCAG

CCACTAAAAAGCATCCTGCTGCTGTGGAGCAATTCTTGCTTTACTAATAT

TAAAAGTTTGGGGAACATATTCATGTTTTCTGAAGTTTTGCTCCTTATTG

CACATCTTATTGCAACAAAGTGCTTTTTAGCATCCAGCACTGTATTTTTT

ACCTTGAGACAATCTGCATTTCTTTTATAAAACTAAGTATATACTTTATA

GGCTTTATGATGACTGTTATGTTTAAAAGCAGTCACTGTGAAAATTGCAA

TGATAATTTTATATGTTAGTTTATCAAACATCAATCTTGTTTAACTTTAT

ATTTTGTTACCTATACTTTGGGGGATCAAGGGAAGAGATGGAACTCTTCC

TCTGAAAATGCTTCTTGGTACTTAAAGTAGTAAAACTATAAAACAGTAAA

CATCCAGTATTGGGAGATGATATGATAGGGCATTATGAATTCCTATGGGT

ATCTAAATTATGTATGTCAATTGGACATTGTAGAAGGTATGTAAATCATC

ATGGTTATGTATAACTTAACCTTGATTTATAAGGTCTTAATTCAGATTAT

GACTATTCGTTGACATCTCATGAGAAGCTTTAGAAAACTTTCTATTTTTA

AACACCATTTATATGTGGACTTCTGTTGTCACTGACTTTGGGCTTTATAT

TTTCACAGAGTCTTTATGGAAAAAGTAGAATTTATTTTCCACTCTTGTAG

CTATAGCTGCTGCACATTTTCATCCTGATTTATTTTTTTGTTTCTTACCT

TTGATGTTTTCAAACCAAGGATTGTGATTTTAGGTTAGAATTACATATTA

GAAACATTAAGACTATGTCTTTGGATCAAAATGCTTTAGTGATTAACCTA

CTTTGAAGACATACTCTTAAGCAATCTGTATCTTAAATTTATGTGAATAC

ATTTTTAGAAAATGATAAAGAAAAATGGAATTACTTCAAAGTGTTTCTTG

AGTCATTGATTCTTTTAGCATCTCAAATGTTAATTAGAATAATTGGAATC

ACTTTTTAAACTTTTCAAGTTACCTTCCTTGGGAAGTTTGTGTAGTGTTA

CAGTTTAGTTTAGCTCCTCTTATAGGGCAATGGTTTGCTAGTTTAAAACT

GTAACCAAATGAACTGGTCAGACAACTCATATCTAAAACACTTAAAATGT

TAGAACGTTTGGGAATGTTATAACCTAAGCGTTTTTGCTGATAACTTTTT

GTTATTTATAGACATTTGTGTATTTAACATACTTACTTCTGGAAATATAT

GCCTTTCCTAAAACTTAACCACACATCCACTACCATGGCCTATGTATAGA

ATTGAATATTTTGGACCATGTTATCTGTGGCACAGTCAGTGCTGTGTTTG

AGGTAAATGCAGTAACGGTTAGTTTTCTACTTTGCCTTATAAAAGGTAGA

AACCATGTGTATGTTATGTTTGTCTATAAAAGAAAAATACTAATATTAAA

TTATTTCTTATGACTGTGAGTCACTCACTTATTTTTCCAATAATTGATAT

TGTACATTCTTAGTGCCATTAGGTATGTATGTATGTAACTTTTACAGTTT

TTCAGCTGAAAGTTGTATTTTTTTTTAATCAGGGCTCTTTAATCTCATTT

TAATTTCCTTTGTTTGAATGAACTGTAGTTATTTTCTGTATTCCTATATT

AACCATCTAAACCAACTGTAATGACACGTACACTAATGAAGAATTGAACA

TTTGTATTTGT

>hg19_ct_ARAlincRNAs_9727_ARAlincRNA_0033.3 range=chr1:149242295-149251326 5'pad=0 3'pad=0 strand=+ repeatMasking=none

GATGCAGGCATGTCTGTCTTCTCTCAGAGTGTGAAGAGTGCAAAGACCTT

ATAGAATCTGTGCTGGAGGAGGAGCTGCAGTTTCAGGAGAGGGAGCTGAC

CAAATTGCCAAGGCCAGCAGCAAGGCTCCGGATACATGATCCCTTAATTC

AGGCTCAGGCTGAAGAACTGACCCACTTACGACAGAAGATACAAGAAGGG

AGAGGTGTCTGCTACCTTTTCACCCAGCATGTGAAGAACACAGTCAAGTC

TTTTGAGGGCCTTCTCAGGAACACTGGCATTGCCTACTACCAGAGACAGA

GATTCTGTGAGCAAATGGTACAAGGAAGCCAGCTAACAGAGATCCTTGTC

AGAAAACTTGCCACAGAGTCTGCTCTCTAACCCGAGCTAGAGTGCAGTGA

CGCGATCTTGGCTGACTGCAGTCTGTGCCTCCTGGGCTCAAGCAGTCATC

CCACCTCAGCCTCCCAAGTAGCTGGGACTACAGGTGTGTGCCACCACACC

TGGCTAATTTTCATATTTTTTGTAGAGACAGGGTTTCTCCATGTTGCCCA

GGCTGGTCTTGAACTACTGAGCTCAAGTGACCCATCTGCTGTGGCCTCCC

AAAGTTCTGGGATTACAGATATGAGCCACTGTGCCTGGCCTGAGAGTTCT

TAGTTTCAATCAGTAAGAGAACAAACTGAGAAGAAAAAAAAAGAATTACC

CCCAACAACATCTGAGGAAGCAGAACAGGGACAGACTTGAAGGCACCTGG

GCAGTCCTCTGGTTTTGGAGTAGAGGCAGCTGACGTCTGGTGATGATTCA

CCATATCAGGAACACCCTGTCCAGTGGGCTCAGGCAAGCCTGCCAGGCCA

CTTCTCTAAAGACACAACCTGAGGCCTCCTGCAATAATTCTCACAGAGCA

TTCTGAACATACATAAAATCACAATGGTAAGAAAATGAAGACAGGCAAAA

GCCACTGGCTCCCAGGTAACTCTACATGATGAAGGGGCTGATAATGGACT

GACCAAATCTAGAGAGAATTCCAGAAGCAAGGACTCAAGAGGTTAAAGGT

CCCAGATCTAGAAAAAGCAGAAACTGCTGATTGTTCCTTTTTTTGTTGAC

TGATTATACAATGTGTCCTTTTAAAAATGGTTCTCTGTTCTCTTTGCAGT

TGTTGTATTG

>hg19_ct_ARAlincRNAs_9727_ARAlincRNA_0033.4 range=chr1:149245050-149265510 5'pad=0 3'pad=0 strand=+ repeatMasking=none

GATACATGATCCCTTAATTCAGGCTCAGGCTGAAGAACTGACCCACTTAC

GACAGAAGATACAAGAAGGGAGAGGTGTCTGCTACCTTTTCACCCAGCAT

GTGAAGAACACAGTCAAGTCTTTTGAGGGCCTTCTCAGGAACACTGGCAT

TGCCTACTACCAGAGACAGAGATTCTGTGAGCAAATGGTACAAGGAAGCC

AGCTAACAGAGATCCTTGTCAGAAAACTTGCCACAGAGTCTGCTCTCTAA

CCCGAGCTAGAGTGCAGTGACGCGATCTTGGCTGACTGCAGTCTGTGCCT

CCTGGGCTCAAGCAGTCATCCCACCTCAGCCTCCCAAGTAGCTGGGACTA

CAGAATCAACCCCAATGCTCACTAACAAACTGAGAAGAAAAAAAAAGAAT

TACCCCCAACAACATCTGAGGAAGCAGAACAGGGACAGACTTGAAGGCAC

CTGGGCAGTCCTCTGGTTTTGGAGTAGAGAAAATCACAATGGTAAGAAAA

TGAAGACAGGCAAAAGCCACTGGCTCCCAGAAGATGAGATCCAGAGCCAG

TGGCAGCACCTGAAAGAGATCCTTTTCATCAACAACTGCCTGTGAGAAAA

GCTAGAACATCATCACAGCAGCTTTGACGAAGAAAATGGATGCACCTCTA

ACCTCTACAGGCAAATCATAGATTCCATTGTCCAGCTGTAAAATGAAAAC

AGAGTTCTCAGAGAAGAATACCGGAGACTCCCCCGACTTGCCTGAATCAA

ATTTCCAGAGGTGAACATAGACGTGGAGTTCCACATCCGGCACAACTACC

CCTGGAACAAGTTGCCGGCCAACGTGAGGCAGTCATTAAGTGGAGATAGC

TCATCATGGAGGTCTTCATCTTTGTCTTCACATCAAGTAGGCTGAGGAGG

AGGAAATACAGGAAGTATTGGTCTTGCTGTTCAGAGTGGCAGAGGAGAAA

GAAAATCCCAGCCGTCTTGGAAATTCACAGAGAGAATATGAAAATCAGGT

TGTCCTGTACAGTATCTGCAATCAGTTACGATACAGAAATAACTTAAACA

TGTCAAGAAAGATGAACGCAGATACTATGAGGAACTGCTAAACTACAGCC

GAGATCATCTCATGCTGTACCCTTACCGTCTATTGGATATTATGGTGAAG

AGCTTGAGCATAACACCATTTTCATATTACACTGGGATTATGGAGAATAT

TATGAACAGTGGGCACAATTTTACTGCTGCTGACTGTCTAAGGCTTCTTG

GCATAGGAAGAAACCAGTATATTGATCTTATGATTCAGTGTAGATCATCA

AAAAAATTCTTCAGAAGGAAAACAGCCGGTGATCTTCTACCAATAAAGCC

AGTGGAAATTGCCATAGAGGCATGGTGGGTGGTGCAGGCTGGATATATCA

CAGAAGATGACATCAAGATATGCACTTGGCCTGAGAAATGTGCTACTGAT

AAGACCGTTGATTCAGGCCCTCAACTCTCTGGATCACTAGATTACAATGT

AGTACATAGTTTGTATAAAAAAGGATTTATTTATCTGGATGTACCAATGT

CTGATGACAGTTGTATAGCAGTTGCACCCCTTGAAGGTTTTGTAATGAAT

CGAGTGCTTATTTTGATTATTTTGAAACTCTACTCTATAAGATATTTGTT

TCAGTAGATGAGCACACAAATGTGGCAGAGCTTGCAAATGTCCTTGAGAT

TGACTTATCCCTGGTTAAGAATGCTGTTTCAATGTATTGCTGATTGGGCT

TTGCCCATAAGAAGGGACAAGTAATAAATTTGGATCAACTTCATTCATCA

TGGAAGAATGTTCCATCCATAAACAGATTAAAGAGTACCTTAGATCCATA

GAAGATGCTCTTGTCATGGGGTGGAGGGGAAAGTAGGAGGCCTGTACAAG

AAGCTTCATCGGCAACTGACACTGATACAAATAGTCAAGAAGATCCAGCT

GACACAGCCAGTGTAAGAAGCCTGAGTCTGTCTGCAGGACACACGAAGCA

CATCGCATTCCTGTTTGACTCCACTCTTACTGCCTTCTTAAAGATGGGAA

ATCTTTCACCAGTTCAGAGCACTGGTGAAAGAGAAGCACAGAGATATTTT

GATCATGCGCTTACTCTGAGAAACACAATACTGTTTCTGCGTCATAACAA

AGATCTAGTTGTGCAAACTGCACAGCCAGACCAACCCAATTATGGTTTTC

CTCTGGATCTCTTACGCTGTGAAAGCCTTCTTGGTCTGGACCCTGCAACT

GGCAGCAGAGTTCTAAACAAAAATTACACACTGCTTGTTTCCATGGCTCC

CCTCACCAATGAAATCCGGCCTGTCAGCAGCTGCACCCCTCAGCATATTG

GACCAGCTATCCCAGAAGTCAGCTCTGTCTGGTTTAAACAGTACATTTAC

GTTTATCATATCACTGGACAAGGACCACCATCCCTTTTATTATCCAAAGG

TACAAGACCTCGAAAACTGCCAGATATATTTCAGAGTTATGATCGATTGC

TAATAACATCTTGGTGTCATGATCCTGGAGTAGTTCCTACCTCAAATGTG

CTCACGATGTTGAATGATGCTTTAACACATTCTGCAGTTTTAATTCAGAG

GCATGGTCTGCATGGGATAGGAGAAACTGTCCATGTCCCATTTCCATTTG

ATGAAACAGAACTACAAGGAGAGTTCACTCGTGTCAATATGGGTGTTCAT

AAAGCATTGCAGATACTAAGGAACAGAGTGGACTTACAACATCTCTGTGG

ATATGTCACCATGTTGAATGCTTCCAGCCAACTTGCAAATAGAAAACTCA

GTGATGCTTCTGATGAGAGAGGAAAACCTGATTTGGCTTCTGGCTCAGAT

GTTAAATGGGAGTACAGAGTCATTTGAAATGGTCATTGAAGAAGCAACTA

TAGATTCAGCAACAAAGCAAACCTCTGGTGCCACAACAGAAGCAGATTGG

GTTCCTCTCGAGCTGTGCTTTGGAATTCCACTGTTCAGTTCCGAATTAAA

CCGGAAAGTTTATAGGAAAATTGCTACACATGGCCTTTGCAGAAAAGAGA

GCCTTCAAAACCTCTTACATTCCAGTAGAAAACTCTCTCTGCAAGTCCTT

AACTTTGTTCACTCATTCCAGGAAGGTGCTTCAACATTGGATATTCACAC

AGAGCCCAGTTTTTCAAGTTTGCTTTCACAGTCATCGTATGCTGACATGG

GTGTTCCACTTCCTGCAAAAAATTTAATATTTAAAGATGGTGTCTTATCA

GAACGGAGTGGATGGTCACCTTCCTCACTTATTGCTAATCTCCATTTGCA

ATAATTTGGTTACACCATTTGCTGCTCACACTTTCTGCCTTTTTTCTTTC

TTAACGTTAGCTTTATAGTGTCAGCCACTAAAAAGCATCCTGCTGCTGTG

GAGCAATTCTTGCTTTACTAATATTAAAAGTTTGGGGAACATATTCATGT

TTTCTGAAGTTTTGCTCCTTATTGCACATCTTATTGCAACAAAGTGCTTT

TTAGCATCCAGCACTGTATTTTTTACCTTGAGACAATCTGCATTTCTTTT

ATAAAACTAAGTATATACTTTATAGGCTTTATGATGACTGTTATGTTTAA

AAGCAGTCACTGTGAAAATTGCAATGATAATTTTATATGTTAGTTTATCA

AACATCAATCTTGTTTAACTTTATATTTTGTTACCTATACTTTGGGGGAT

CAAGGGAAGAGATGGAACTCTTCCTCTGAAAATGCTTCTTGGTACTTAAA

GTAGTAAAACTATAAAACAGTAAACATCCAGTATTGGGAGATGATATGAT

AGGGCATTATGAATTCCTATGGGTATCTAAATTATGTATGTCAATTGGAC

ATTGTAGAAGGTATGTAAATCATCATGGTTATGTATAACTTAACCTTGAT

TTATAAGGTCTTAATTCAGATTATGACTATTCGTTGACATCTCATGAGAA

GCTTTAGAAAACTTTCTATTTTTAAACACCATTTATATGTGGACTTCTGT

TGTCACTGACTTTGGGCTTTATATTTTCACAGAGTCTTTATGGAAAAAGT

AGAATTTATTTTCCACTCTTGTAGCTATAGCTGCTGCACATTTTCATCCT

GATTTATTTTTTTGTTTCTTACCTTTGATGTTTTCAAACCAAGGATTGTG

ATTTTAGGTTAGAATTACATATTAGAAACATTAAGACTATGTCTTTGGAT

CAAAATGCTTTAGTGATTAACCTACTTTGAAGACATACTCTTAAGCAATC

TGTATCTTAAATTTATGTGAATACATTTTTAGAAAATGATAAAGAAAAAT

GGAATTACTTCAAAGTGTTTCTTGAGTCATTGATTCTTTTAGCATCTCAA

ATGTTAATTAGAATAATTGGAATCACTTTTTAAACTTTTCAAGTTACCTT

CCTTGGGAAGTTTGTGTAGTGTTACAGTTTAGTTTAGCTCCTCTTATAGG

GCAATGGTTTGCTAGTTTAAAACTGTAACCAAATGAACTGGTCAGACAAC

TCATATCTAAAACACTTAAAATGTTAGAACGTTTGGGAATGTTATAACCT

AAGCGTTTTTGCTGATAACTTTTTGTTATTTATAGACATTTGTGTATTTA

ACATACTTACTTCTGGAAATATATGCCTTTCCTAAAACTTAACCACACAT

CCACTACCATGGCCTATGTATAGAATTGAATATTTTGGACCATGTTATCT

GTGGCACAGTCAGTGCTGTGTTTGAGGTAAATGCAGTAACGGTTAGTTTT

CTACTTTGCCTTATAAAAGGTAGAAACCATGTGTATGTTATGTTTGTCTA

TAAAAGAAAAATACTAATATTAAATTATTTCTTATGACTGTGAGTCACTC

ACTTATTTTTCCAATAATTGATATTGTACATTCTTAGTGCCATTAGGTAT

GTATGTATGTAACTTTTACAGTTTTTCAGCTGAAAGTTGTATTTTTTTTT

AATCAGGGCTCTTTAATCTCATTTTAATTTCCTTTGTTTGAATGAACTGT

AGTTATTTTCTGTATTCCTATATTAACCATCTAAACCAACTGTAATGACA

CGTACACTAATGAAGAATTGAACATTTGTATTTGT

>hg19_ct_ARAlincRNAs_9727_ARAlincRNA_0033.8 range=chr1:149251081-149252120 5'pad=0 3'pad=0 strand=+ repeatMasking=none

AAAATCACAATGGTAAGAAAATGAAGACAGGCAAAAGCCACTGGCTCCCA

GGCACACTTTACTCCCAGTCAAAAGTGGACTGTATACTTCCTCTTTGATG

TGATGGTCCCTTTGGTTTTGGTGCCTCTTCCATGAGCTTCAGATCAGAAC

TAGCTATGTGGTTCCAGGCTGCCCTGGTTTAATCCCTTGTCTTGCCTACT

TCACCTGGCTCAAGAGGGAAGTCCAGGAGGAAGAAATGGATGAAGTCCTG

GAGGACTCACTAGATGAACAGTATTTGACTCATTCCAGCTGCCATGACTC

CCACCAGCTTCCCAGCAGCAATGCCTTCCTCTTTGATGCACAGGAAGGCC

CCTCGGCTGTGGAAATAGCCA

>hg19_ct_ARAlincRNAs_9727_ARAlincRNA_0033.11 range=chr1:149254297-149254404 5'pad=0 3'pad=0 strand=+ repeatMasking=none

AAGATGAGATCCAGAGCCAGTGGCAGCACCTGAAAGAGATCCTTTTCATC

AACAACTGCCTGTGAGAAAAGCTAGAACATCATCACAGCAGCTTTGACGA

AGAAAATG

>hg19_ct_ARAlincRNAs_9727_ARAlincRNA_0034.6 range=chr1:149575345-149576700 5'pad=0 3'pad=0 strand=+ repeatMasking=none

CAGCTACGTCACCCTGAGGTGTCCTTAGGCAGAACAGAAAGTGAGAATCA

GAGCAGTGTCTCATTTCTGAACACCTGGATTGTAGCTTTCCCTGGACTGA

CTGTCAAAGGAGGTGGTTTTAAAAAGCAAGCCAAACAGGTTTCCATCCTT

TAGGAAGACCCTGGAATGTGCCAGTTGATTCTGACAATTTCTCATGAACA

GAAACACCTAGGAAGGGAGAACTGGATTTATTTAATGGGGATGTGTTCAA

GGCAAGACCGAATTCAGAAGGATATCGACGTCGTGATCCAGAAGTCCAGA

GCTGAGGACTGCCTGTTTGCAG

>hg19_ct_ARAlincRNAs_9727_ARAlincRNA_0034.8 range=chr1:149575345-149616230 5'pad=0 3'pad=0 strand=+ repeatMasking=none

CAGCTACGTCACCCTGAGGTGTCCTTAGGCAGAACAGAAAGTGAGAATCA

GAGCAGTGTCTCATTTCTGAACACCTGGATTGTAGCTTTCCCTGGACTGA

CTGTCAAAGGAGGTGGTTTTAAAAAGCAAGCCAAACAGGTTTCCATCCTT

TAGGAAGACCCTGGAATGTGCCAGTTGATTCTGACAATTTCTCATGAACA

GAAACACCTAGGAAGGGAGAACTGGTGAGTCTTTTCAAGAGGAGGAAGTC

CCTCAAAAGGGGCTGTGTTTCGCAAACAGCGCCCCAAAAGGGCTGAAATA

CTTCGATCTGGAGGAAAAAGACTCAGGCACTGAGGGAAGGGGAGGGTGCA

CATCTGGAGTAATTTCAAGGCTTTTTCCGTGTTATCTGAGGTGGCAACAA

GTAAAGATTTGTTCGTTTTATTAGATTTATTTAATGGGGATGTGTTCAAG

GCAAGACCGAATTCAGAAGGATATCGACGTCGTGATCCAGAAGTCCAGAG

CTGAGGACTGCCTGTTTGCAGGGCCCGCCTGGCAGGACGACTGAGCAAGT

TCTTGGAAAACCAGAGATATTAGAGCGGTGAATCGCGCTGGTCACGTTGG

ACACCTGCGCGTTAGGAGATTCTGGAGCCAGAGGGAGAGACCCCAGAAAC

GCCCCGGTCGGCGACCCAGCCCGAGACGCCTGGGGTCCCAAGGGAAGCTG

AACGCCCGGTGGGCTCCCGGGATGGTTCTTCCGGTTCTTTGTGCCGCCTT

CACCCAGTGAAGGAGCCTGTATCCACCCTGCCCAGTCGCTGTTGGGCTGC

TGCGGAGCTTCCGCTGCCATCTTCGGATCCTGTGTTCCGCCCGGGGCTCC

ACCAGGGCAGGGATGGTGGTGAGGGTCGCTCGTGGGTCCCCTGGCAGGGA

GCAGGGTCTGGCACTCACAAGGGCGCACGACTAGGACTTGTCGAATGAAT

CCCTTGTCGCCTTTAGCTTTTAGTCCTTTGAAGAGAG

>hg19_ct_ARAlincRNAs_9727_ARAlincRNA_0034.7 range=chr1:149575345-149616885 5'pad=0 3'pad=0 strand=+ repeatMasking=none

CAGCTACGTCACCCTGAGGTGTCCTTAGGCAGAACAGAAAGTGAGAATCA

GAGCAGTGTCTCATTTCTGAACACCTGGATTGTAGCTTTCCCTGGACTGA

CTGTCAAAGGAGGTGGTTTTAAAAAGCAAGCCAAACAGGTTTCCATCCTT

TAGGAAGACCCTGGAATGTGCCAGTTGATTCTGACAATTTCTCATGAACA

GAAACACCTAGGAAGGGAGAACTGGATTTATTTAATGGGGATGTGTTCAA

GGCAAGACCGAATTCAGAAGGATATCGACGTCGTGATCCAGAAGTCCAGA

GCTGAGGACTGCCTGTTTGCAGGGCCCGCCTGGCAGGACGACTGAGCAAG

TTCTTGGAAAACCAGAGATATTAGAGCGGTGAATCGCGCTGGTCACGTTG

GACACCTGCGCGTTAGGAGATTCTGGAGCCAGAGGGAGAGACCCCAGAAA

CGCCCCGGTCGGCGACCCAGCCCGAGACGCCTGGGGTCCCAAGGGAAGCT

GAACGCCCGGTGGGCTCCCGGGATGGTTCTTCCGGTTCTTTGTGCCGCCT

TCACCCAGTGAAGGAGCCTGTATCCACCCTGCCCAGTCGCTGTTGGGCTG

CTGCGGAGCTTCCGCTGCCATCTTCGGATCCTGTGTTCCGCCCGGGGCTC

CACCAGGGCAGGGATGGTGGTGAGGGTCGCTCGTGGGTCCCCTGGCAGGG

AGCAGGGTCTGGCACTCACAAGGGCGCACGACTAGGACTTGTCGAATGAA

TCCCTTGTCGCCTTTAGCTTTTAGTCCTTTGAAGAGAGGTGAGAGTGGAA

ATCAAGAGATTTTTTTCCACGGGGAAGTTCTTTTTACAAAGCGTTGATTT

CTTGGCACCCCGCGGGGCGGGCAACTGACACGACCTCCGGTGCACCTTCT

GCGCTGTGGAGCCTCTGGGGCTCAGCTGGGCGGTGGTCGGGTCGTGGGGC

GGTAGGGCGGGAGCGGGGGAAGGGAAAGCAAAAGCTGGAAAAGAAGCAGG

GGAGTTGTGAACCAGACATCCAGACCTCCTGAAGGGCTCGTGCAGACGTA

CAGGCGGGATCTTCTGGAAGTGAGAATTGTTTTTGTTTGTTTATCTATTG

TAGCAGAATGGGGAAATGGAGAGAGAACCTGAAAGAGCCCCAAACTCGAG

GACCTATTGCTCCCCAAGAATAACATCTTCCAGAACTAGACAGAAAACTA

GGCGTCTGGGAACCCTGAAATCCTTGGAGTAGATCATCATGACCCTCTGT

GTTCCTTTTGGCAAATGACTTGCTTCCATTGTTTGTTTGTTCAATTGTCT

GTTTGTTAAATAAATAAAACCCTTTTTATGTATCTTTAAAATTACATTGG

TTCTATTATTTTATGATTACAAACGATGCTGCAGTCATCATTCTTGTACA

CTTCTCATTGGCCACTGGTGTATTTCTATAGGGTGGAGGCCTG

>hg19_ct_ARAlincRNAs_9727_ARAlincRNA_0034.5 range=chr1:149575482-149616885 5'pad=0 3'pad=0 strand=+ repeatMasking=none

GGTTTCCATCCTTTAGGAAGACCCTGGAATGTGCCAGTTGATTCTGACAA

TTTCTCATGAACAGAAACACCTAGGAAGGGAGAACTGGGCCCGCCTGGCA

GGACGACTGAGCAAGTTCTTGGAAAACCAGAGATATTAGAGCGGTGAATC

GCGCTGGTCACGTTGGACACCTGCGCGTTAGGAGATTCTGGAGCCAGAGG

GAGAGACCCCAGAAACGCCCCGGTCGGCGACCCAGCCCGAGACGCCTGGG

GTCCCAAGGGAAGCTGAACGCCCGGTGGGCTCCCGGGATGGTTCTTCCGG

TTCTTTGTGCCGCCTTCACCCAGTGAAGGAGCCTGTATCCACCCTGCCCA

GTCGCTGTTGGGCTGCTGCGGAGCTTCCGCTGCCATCTTCGGATCCTGTG

TTCCGCCCGGGGCTCCACCAGGGCAGGGATGGTGAATGGGGAAATGGAGA

GAGAACCTGAAAGAGCCCCAAACTCGAGGACCTATTGCTCCCCAAGAATA

ACATCTTCCAGAACTAGACAGAAAACTAGGCGTCTGGGAACCCTGAAATC

CTTGGAGTAGATCATCATGACCCTCTGTGTTCCTTTTGGCAAATGACTTG

CTTCCATTGTTTGTTTGTTCAATTGTCTGTTTGTTAAATAAATAAAACCC

TTTTTATGTATCTTTAAAATTACATTGGTTCTATTATTTTATGATTACAA

ACGATGCTGCAGTCATCATTCTTGTACACTTCTCATTGGCCACTGGTGTA

TTTCTATAGGGTGGAGGCCTG

>hg19_ct_ARAlincRNAs_9727_ARAlincRNA_0034.1 range=chr1:149576149-149576387 5'pad=0 3'pad=0 strand=+ repeatMasking=none

GTGGAAGGCCGGAGTGCGGGCCGTGAGGGGTGTGCGCGCTTGAGCCGGAG

CGGGGCTCGCCCCTCGTCGGCTCCCGCCGCCCAGCCGGTGATCGCTCTCC

GGCCGTCCCCGGCACCCTCGGTCCCCCACGGCGGTTGGTCCGGGCGGGGG

AAGGAGAAAGTGAGACTCGGTTTCATCACCAATCCATCGCCAGAAGGGGA

GGAAATTGGAATCCAGCAGCGGCGAGCAGCAGCTGGGCG

>hg19_ct_ARAlincRNAs_9727_ARAlincRNA_0034.3 range=chr1:149576149-149582604 5'pad=0 3'pad=0 strand=+ repeatMasking=none

GTGGAAGGCCGGAGTGCGGGCCGTGAGGGGTGTGCGCGCTTGAGCCGGAG

CGGGGCTCGCCCCTCGTCGGCTCCCGCCGCCCAGCCGGTGATCGCTCTCC

GGCCGTCCCCGGCACCCTCGGTCCCCCACGGCGGTTGGTCCGGGCGGGGG

AAGGAGAAAGTGAGACTCGGTTTCATCACCAATCCATCGCCAGAAGGGGA

GGAAATTGGAATCCAGCAGCGGCGAGCAGCAGCTGGGCGGTCACATCTGG

AAATGGAAAGCCGACCTCCCCCTCCTCCTCCACCTCCTCCTCCTCCTCCT

CCTCCTCCTCCTCCTCTCACCCAGGATCACTTCCGAAACCACTTGGCCTT

CAGCCCCTGCCTCGGCCAGAGGTTTCATTTTTAACTGAATATTTACGAAA

GCTGAAAGCGTGCGAGGGGGGTGGGGTGGAAATAGCGGCTGCTTCTTTTC

CAAGGATTTATTTAATGGGGATGTGTTCAAGGCAAGACCGAATTCAGAAG

GATATCGACGTCGTGATCCAGAAGTCCAGAGCTGAGGACTGCCTGTTTGC

AGATTTCAGATACTCAGACTCCACCTTTACTTTTACCTATGTTGGCGGCC

CCAGAAGGTATTTATGGGTGTAGAGTTGCTTCATTTCCTGTTCACACTCT

TGTGTTTCCTTTGTTCTTAGCGTGTTCCGTGTTCGGGTATTTTGGTTGCT

TGTTTGATTACTGTGGCTTTTCTTTACCTACAACCCAAGCCTGACGTATT

AGCAATAAACTCCTTAAAACCCAAAGGTTTGGGCTTCTGTTCCTTTCACT

TGCAGTCAGACATGGAGTTAGTGGTAGAAGAAACAGAAGGGGTAACCTGC

ATGGTGACAGCTACTGAGGGGATGGATAGGAAAGCAGGCTGAGTCCCTGG

GGCCAGTGGTTACCAAAGCCAAGGAGAGAGCAAGGGGAGCCCAGTGGGCC

TGGCCATGGACTGCTCTGGAATTCCGAGTGTGAACTTTCAGCCAAGAAGG

TAGTGTGAAAATATTACTGTGAGGTTTTAAAAGTACACAAATAACAATTG

TTTTTTGTAAAAAGAAAAAAAAAAAGTTAGAAAAAGAGATATGCCAAAAA

GAAGAAAGTAAAAAATACATGACTTCTGTCTTCTGTTAACATATTAAAAT

TTATGCTTTTAGATTTTTATGTGTCTATATACATGTAAAATATATATGCA

TCCCCAAATTGTATTATACTCTTTTGAGACCTCACATTTTTTTCACCCAA

GAATATAATATTAATTTCTTTCCTTTAGAAAAGACATGAGACTAAAGAAT

GTTTATTGAGTTGAGAGTTTCCCAATCAAGGAGTATGACTCTGTATAATA

ATAAATCTAATGTTTTATTAGAAATTATAATAAAATTAGAAATTTAATAT

TTGGCAGGATGTGAGTTGCCTGCATATGTACACCCTTTATTATATGACTA

GACACTCTTTTGTGTGGTCATGGATATATTGGCATCTTTTGAAGTGTATC

CTTTTTGTTTCAGGAGCTTCTGTCTAGATGAATAGCAAGTGTGACACTGA

TAATAGTAATCGAATATTCCTCCAAGTACAGGCTCCTTCCTCTTTCTCCT

GGACCTTTGCACATGCTGCCTGGAAAACTCACCCACAACCTACTTTCCAT

CCTCTCCTTCGGCTCATCCCTGCTCAGCCCTCTTTGTTGAGATATTTGCC

AAGCCAGATTCTCTGAGAAGCCTTTCCTGGCATCTTCGTCTATATAATTC

TGCTAGTTGTTTCTAAAACTATGTTGTTAGTGTATATTGTAATCACCTAT

TTATGTCTGTTTGACTGTGAGCCTCTTGGAGATGGGAATTCTCTCCTATG

CATTGCGGTTTCCCCAGCCCCTAGCACACTGTCTAGCCCAGTATTCCTTC

TCAGTAAGTAGGTGTTGAATGACTTACAAAATGAATGCTAAAAGACCTGG

TAGACAAGAAGGAATTTCTTAGCCTAAATGACCAGATGTGGAGAAGATAC

TCAAACACTTCAGGGTCCCCAATCTGGTGATGTCACTTGCAGTGGAATTT

CATATGAATCCAGTGTGACAGAAGTTGCCTAGTCAAGTTGTAAGTCCTCT

TCTCTGGGTCACATGTTCAAATACTTTTAGGGGACAGATAATGCAATATT

TAAGATACAGGAAGTGATGAGGACTGTGGCACACTGGAGAGTTTGGACTC

CAAGAGTAATTAATTCAGTTGAAAAAATCAACAGCACTCTGTTGATCAAG

CAAACTTTCTAAGGGCCCTAATCCACCTGGGCCTGCAGTATGTAATTCCA

TAACTCATGGGATGCCATCAAGAGAGACAATCTACTTGATTGTTAAAGGG

CCTTTGTTTTCTTTGGAGTTCCCCAGGAAGGAGAAATGTATGTGTGTGCA

GGGGTGAGGAGGGGGGGAGAGTGAAGAAACAATACTATCCCCTTAAAAAG

TTTTGTCAGTTTTCATTATGAAAGAAGATCAAACGTGCCCACTCCAGAAA

TCTTGGCATATGCAGTTATTTTGAAGCCATTCAGCGTTCTTCAAGATGTC

ACAATGAAAAGAGTGATGATTTTTTAAATAAAAAATTAACATGTTTCTCT

TGGCAA

>hg19_ct_ARAlincRNAs_9727_ARAlincRNA_0034.2 range=chr1:149576149-149592306 5'pad=0 3'pad=0 strand=+ repeatMasking=none

GTGGAAGGCCGGAGTGCGGGCCGTGAGGGGTGTGCGCGCTTGAGCCGGAG

CGGGGCTCGCCCCTCGTCGGCTCCCGCCGCCCAGCCGGTGATCGCTCTCC

GGCCGTCCCCGGCACCCTCGGTCCCCCACGGCGGTTGGTCCGGGCGGGGG

AAGGAGAAAGTGAGACTCGGTTTCATCACCAATCCATCGCCAGAAGGGGA

GGAAATTGGAATCCAGCAGCGGCGAGCAGCAGCTGGGCGCTTTAAATCAT

CTAGGAACTTCATATAAAATTTAATTCCAGTTTCAACTAGTTGTGGAGCA

TTTGATCCAAAATAAAATGAAAGTCCTCTCTGAAGCTGTAGAGGAGGCTC

AAGAATCAGAACAACCTGAAGTTCTTTAAGCTGTCAGTTGAAGGACTAGG

TTTGACTGCAATGCAGTGTGAATATCGCACATGGGACCATTTGTCATACT

GTCAGATGGCAATACATAGATGGATAGAATTTTGCTGTTTGATCAAAGCT

GTGCCATCGGAAACTTGTTTTCCCAAGTATGGGTACATTCTCCTCTTTTC

CTGAGTGGAGAAACCTAGATCTGAGCAAAAAGCATGAATCAATACACCTT

GGGAGGCAGGGTGTGAAATATTTGATGCCATTTCCTTTGCTTGTAAATTG

TTCATTTCATTTGAATATAAATATAAAAGGACTTCTACCAAGAACATGAA

ACTAACATTTTGCCAAGCTAAATTTTAATGAATTTATTTGGACGTTTTTA

TAATCACTTAATATTTTCAGTTCATGTGCTAATATTAACTCTACTTACTG

GAGAGAAAGAAATGGTATTCAATATGATAACTGCCTTGTTACTGCAGAAA

TGTAGAAGTTTGGCATTTTAAGTATCAAATATTTTAAGTCAAAAAATTAC

CAGTGTCCCACAGCACAGAGAGAGAATATATGTTTGCTTGTGTCCCTTTA

ATCTTTCCCCTGTAAAATTAAGCCTAGGGGCCTTACACTATTTTAAATTT

TCAAAATTAGGTCATGTAAATGTTGTGTCAGATTTCCAATTCATAAGTAG

TATACTTATGAATACTGAATAGTGAACATAGGGTACATTTATATCCTGCT

GATTGCTTAGCTGTGGA

>hg19_ct_ARAlincRNAs_9727_ARAlincRNA_0034.9 range=chr1:149576149-149616885 5'pad=0 3'pad=0 strand=+ repeatMasking=none

GTGGAAGGCCGGAGTGCGGGCCGTGAGGGGTGTGCGCGCTTGAGCCGGAG

CGGGGCTCGCCCCTCGTCGGCTCCCGCCGCCCAGCCGGTGATCGCTCTCC

GGCCGTCCCCGGCACCCTCGGTCCCCCACGGCGGTTGGTCCGGGCGGGGG

AAGGAGAAAGTGAGACTCGGTTTCATCACCAATCCATCGCCAGAAGGGGA

GGAAATTGGAATCCAGCAGCGGCGAGCAGCAGCTGGGCGAGCTACCAGCT

TTGAGGGCAGAACCCGCGTGGCCCGAGCGGGGGTCTAGGCGAAGGGGCAG

TGCGTGGTATTTCCAGCCCCACGTAGTGCCCAAAGCCTGCAAGCACGCCT

CCTGGGTTCCAGCAGCGGCGGCAGAGCGGGGTTGGTTGGACCCACCAGGG

CAGCGCAGGGGCTGCTGCTTCGCCACCTGCTGCTTCGCCACGAAAGAGTT

CCCGTGCCGTGGGAGCAAGTCTGGGACCTCTGGTCGGACCGGAGAGTCGC

AGCTGTGTGTTAGGGCTAGGATGGCTCCTGGATGCGCGTGACAATGGGGA

AATGGAGAGAGAACCTGAAAGAGCCCCAAACTCGAGGACCTATTGCTCCC

CAAGAATAACATCTTCCAGAACTAGACAGAAAACTAGGCGTCTGGGAACC

CTGAAATCCTTGGAGTAGATCATCATGACCCTCTGTGTTCCTTTTGGCAA

ATGACTTGCTTCCATTGTTTGTTTGTTCAATTGTCTGTTTGTTAAATAAA

TAAAACCCTTTTTATGTATCTTTAAAATTACATTGGTTCTATTATTTTAT

GATTACAAACGATGCTGCAGTCATCATTCTTGTACACTTCTCATTGGCCA

CTGGTGTATTTCTATAGGGTGGAGGCCTG

>hg19_ct_ARAlincRNAs_9727_ARAlincRNA_0034.27 range=chr1:149576181-149616885 5'pad=0 3'pad=0 strand=+ repeatMasking=none

TGCGCGCTTGAGCCGGAGCGGGGCTCGCCCCTCGTCGGCTCCCGCCGCCC

AGCCGGTGATCGCTCTCCGGCCGTCCCCGGCACCCTCGGTCCCCCACGGC

GGTTGGTCCGGGCGGGGGAAGGAGAAAGTGAGACTCGGTTTCATCACCAA

TCCATCGCCAGAAGGGGAGGAAATTGGAATCCAGCAGCGGCGAGCAGCAG

CTGGGCGGGCCCGCCTGGCAGGACGACTGAGCAAGTTCTTGGAAAACCAG

AGATATTAGAGCGGTGAATCGCGCTGGTCACGTTGGACACCTGCGCGTTA

GGAGATTCTGGAGCCAGAGGGAGAGACCCCAGAAACGCCCCGGTCGGCGA

CCCAGCCCGAGACGCCTGGGGTCCCAAGGGAAGCTGAACGCCCGGTGGGC

TCCCGGGATGGTTCTTCCGGTTCTTTGTGCCGCCTTCACCCAGTGAAGGA

GCCTGTATCCACCCTGCCCAGTCGCTGTTGGGCTGCTGCGGAGCTTCCGC

TGCCATCTTCGGATCCTGTGTTCCGCCCGGGGCTCCACCAGGGCAGGGAT

GGTGCAGAATGGGGAAATGGAGAGAGAACCTGAAAGAGCCCCAAACTCGA

GGACCTATTGCTCCCCAAGAATAACATCTTCCAGAACTAGACAGAAAACT

AGGCGTCTGGGAACCCTGAAATCCTTGGAGTAGATCATCATGACCCTCTG

TGTTCCTTTTGGCAAATGACTTGCTTCCATTGTTTGTTTGTTCAATTGTC

TGTTTGTTAAATAAATAAAACCCTTTTTATGTATCTTTAAAATTACATTG

GTTCTATTATTTTATGATTACAAACGATGCTGCAGTCATCATTCTTGTAC

ACTTCTCATTGGCCACTGGTGTATTTCTATAGGGTGGAGGCCTG

>hg19_ct_ARAlincRNAs_9727_ARAlincRNA_0034.26 range=chr1:149576450-149616783 5'pad=0 3'pad=0 strand=+ repeatMasking=none

CTCCTCCTCCTCCTCTCACCCAGGATCACTTCCGAAACCACTTGGCCTTC

AGCCCCTGCCTCGGCCAGAGGTTTCATTTTTAACTGAATATTTACGAAAG

CTGAAAGCGTGCGAGGGGGGTGGGGTGGAAATAGCGGCTGCTTCTTTTCC

AAGGATTTATTTAATGGGGATGTGTTCAAGGCAAGACCGAATTCAGAAGG

ATATCGACGTCGTGATCCAGAAGTCCAGAGCTGAGGACTGCCTGTTTGCA

GCTTTAAATCATCTAGGAACTTCATATAAAATTTAATTCCAGTTTCAACT

AGTTGTGGAGCATTTGATCCAAAATAAAATGAAAGTCCTCTCTGAAGCTG

TAGAGGAGGCTCAAGAATCAGAACAACCTGAAGTTCTTTAAGCTGTCAGT

TGAAGGACTAGGGGCCCGCCTGGCAGGACGACTGAGCAAGTTCTTGGAAA

ACCAGAGATATTAGAGCGGTGAATCGCGCTGGTCACGTTGGACACCTGCG

CGTTAGGAGATTCTGGAGCCAGAGGGAGAGACCCCAGAAACGCCCCGGTC

GGCGACCCAGCCCGAGACGCCTGGGGTCCCAAGGGAAGCTGAACGCCCGG

TGGGCTCCCGGGATGGTTCTTCCGGTTCTTTGTGCCGCCTTCACCCAGTG

AAGGAGCCTGTATCCACCCTGCCCAGTCGCTGTTGGGCTGCTGCGGAGCT

TCCGCTGCCATCTTCGGATCCTGTGTTCCGCCCGGGGCTCCACCAGGGCA

GGGATGGTGGTGAGGGTCGCTCGTGGGTCCCCTGGCAGGGAGCAGGGTCT

GGCACTCACAAGGGCGCACGACTAGGACTTGTCGAATGAATCCCTTGTCG

CCTTTAGCTTTTAGTCCTTTGAAGAGAGGTGAGAGTGGAAATCAAGAGAT

TTTTTTCCACGGGGAAGTTCTTTTTACAAAGCGTTGATTTCTTGGCACCC

CGCGGGGCGGGCAACTGACACGACCTCCGGTGCACCTTCTGCGCTGTGGA

GCCTCTGGGGCTCAGCTGGGCGGTGGTCGGGTCGTGGGGCGGTAGGGCGG

GAGCGGGGGAAGGGAAAGCAAAAGCTGGAAAAGAAGCAGGGGAGTTGTGA

ACCAGACATCCAGACCTCCTGAAGGGCTCGTGCAGACGTACAGGCGGGAT

CTTCTGGAAGTGAGAATTGTTTTTGTTTGTTTATCTATTGTAGCAGAATG

GGGAAATGGAGAGAGAACCTGAAAGAGCCCCAAACTCGAGGACCTATTGC

TCCCCAAGAATAACATCTTCCAGAACTAGACAGAAAACTAGGCGTCTGGG

AACCCTGAAATCCTTGGAGTAGATCATCATGACCCTCTGTGTTCCTTTTG

GCAAATGACTTGCTTCCATTGTTTGTTTGTTCAATTGTCTGTTTGTTAAA

TAAATAAAACCCTTTTTATGTATCTTTAAAA

>hg19_ct_ARAlincRNAs_9727_ARAlincRNA_0034.28 range=chr1:149576491-149616111 5'pad=0 3'pad=0 strand=+ repeatMasking=none

TTGGCCTTCAGCCCCTGCCTCGGCCAGAGGTTTCATTTTTAACTGAATAT

TTACGAAAGCTGAAAGCGTGCGAGGGGGGTGGGGTGGAAATAGCGGCTGC

TTCTTTTCCAAGGATTTATTTAATGGGGATGTGTTCAAGGCAAGACCGAA

TTCAGAAGGATATCGACGTCGTGATCCAGAAGTCCAGAGCTGAGGACTGC

CTGTTTGCAGTCAGACATGGAGTTAGTGGTAGAAGAAACAGAAGGGGTAA

CCTGCATGGTGACAGCTACTGAGGGGATGGATAGGAAAGCAGGCTGAGTC

CCTGGGGCCAGTGGTTACCAAAGCCAAGGAGAGAGCAAGGGGAGCCCAGT

GGGCCTGGCCATGGACTGCTCTGGAATTCCGAGTGTGAACTTTCAGCCAA

GAAGGACGGGGATCCAGAAGGCGTTGTCGGTGACATCACGGAAAGGGCGA

TTTCTATGTAGATGAGGCAGCGCAGGGGCTGCTGCTTCGCCACCTGCTGC

TTCGCCACGAAAGAGTTCCCGTGCCGTGGGAGCAAGTCTGGGACCTCTGG

TCGGACCGGAGAGTCGCAGCTGTGTGTTAGGGCTAGGATGGCTCCTGGAT

GCGCGTGACGCAAGTGACCTTGCGTGTAAAGGGGCCCGCCTGGCAGGACG

ACTGAGCAAGTTCTTGGAAAACCAGAGATATTAGAGCGGTGAATCGCGCT

GGTCACGTTGGACACCTGCGCGTTAGGAGATTCTGGAGCCAGAGGGAGAG

ACCCCAGAAACGCCCCGGTCGGCGACCCAGCCCGAGACGCCTGGGGTCCC

AAGGGAAGCTGAACGCCCGGTGGGCTCCCGGGATGGTTCTTCCGGTTCTT

TGTGCCGCCTTCACCCAGTGAAGGAGCCTGTATCCACCCTGCCCAGTCGC

TGTTGGGCTGCTGCGGAGCTTCCGCTGCCATCTTCGGATCCTGTGTTCCG

CCCGGGGCTCCACCAGGGCAGGGATGGTG

>hg19_ct_ARAlincRNAs_9727_ARAlincRNA_0034.25 range=chr1:149576555-149616111 5'pad=0 3'pad=0 strand=+ repeatMasking=none

AGCGTGCGAGGGGGGTGGGGTGGAAATAGCGGCTGCTTCTTTTCCAAGGA

TTTATTTAATGGGGATGTGTTCAAGGCAAGACCGAATTCAGAAGGATATC

GACGTCGTGATCCAGAAGTCCAGAGCTGAGGACTGCCTGTTTGCAGATTT

CAGATACTCAGACTCCACCTTTACTTTTACCTATGTTGGCGGCCCCAGAA

GATTAGCAATAAACTCCTTAAAACCCAAAGGTTTGGGCTTCTGTTCCTTT

CACTTGCAGTCAGACATGGAGTTAGTGGTAGAAGAAACAGAAGGGGTAAC

CTGCATGGTGACAGCTACTGAGGGGATGGATAGGAAAGCAGGCTGAGTCC

CTGGGGCCAGTGGTTACCAAAGCCAAGGAGAGAGCAAGGGGAGCCCAGTG

GGCCTGGCCATGGACTGCTCTGGAATTCCGAGTGTGAACTTTCAGCCAAG

AAGGTAGTGTGAAAATATTACTGTGAGGTTTTAAAAGTACACAAATAACA

ATTGTTTTTTGTAAAAAGGCCCGCCTGGCAGGACGACTGAGCAAGTTCTT

GGAAAACCAGAGATATTAGAGCGGTGAATCGCGCTGGTCACGTTGGACAC

CTGCGCGTTAGGAGATTCTGGAGCCAGAGGGAGAGACCCCAGAAACGCCC

CGGTCGGCGACCCAGCCCGAGACGCCTGGGGTCCCAAGGGAAGCTGAACG

CCCGGTGGGCTCCCGGGATGGTTCTTCCGGTTCTTTGTGCCGCCTTCACC

CAGTGAAGGAGCCTGTATCCACCCTGCCCAGTCGCTGTTGGGCTGCTGCG

GAGCTTCCGCTGCCATCTTCGGATCCTGTGTTCCGCCCGGGGCTCCACCA

GGGCAGGGATGGTG

>hg19_ct_ARAlincRNAs_9727_ARAlincRNA_0034.4 range=chr1:149576603-149616657 5'pad=0 3'pad=0 strand=+ repeatMasking=none

GATTTATTTAATGGGGATGTGTTCAAGGCAAGACCGAATTCAGAAGGATA

TCGACGTCGTGATCCAGAAGTCCAGAGCTGAGGACTGCCTGTTTGCAGGG

CCCGCCTGGCAGGACGACTGAGCAAGTTCTTGGAAAACCAGAGATATTAG

AGCGGTGAATCGCGCTGGTCACGTTGGACACCTGCGCGTTAGGAGATTCT

GGAGCCAGAGGGAGAGACCCCAGAAACGCCCCGGTCGCAGAATGGGGAAA

TGGAGAGAGAACCTGAAAGAGCCCCAAACTCGAGGACCTATTGCTCCCCA

AGAATAACATCTTCCAGAACTAGACAGAAAACTAGGCGTCTGGGAACCC

>hg19_ct_ARAlincRNAs_9727_ARAlincRNA_0034.24 range=chr1:149576613-149616786 5'pad=0 3'pad=0 strand=+ repeatMasking=none

ATGGGGATGTGTTCAAGGCAAGACCGAATTCAGAAGGATATCGACGTCGT

GATCCAGAAGTCCAGAGCTGAGGACTGCCTGTTTGCAGCAGAATGGGGAA

ATGGAGAGAGAACCTGAAAGAGCCCCAAACTCGAGGACCTATTGCTCCCC

AAGAATAACATCTTCCAGAACTAGACAGAAAACTAGGCGTCTGGGAACCC

TGAAATCCTTGGAGTAGATCATCATGACCCTCTGTGTTCCTTTTGGCAAA

TGACTTGCTTCCATTGTTTGTTTGTTCAATTGTCTGTTTGTTAAATAAAT

AAAACCCTTTTTATGTATCTTTAAAATTA

>hg19_ct_ARAlincRNAs_9727_ARAlincRNA_0034.23 range=chr1:149576633-149577662 5'pad=0 3'pad=0 strand=+ repeatMasking=none

AGACCGAATTCAGAAGGATATCGACGTCGTGATCCAGAAGTCCAGAGCTG

AGGACTGCCTGTTTGCAGATTTCAGATACTCAGACTCCACCTTTACTTTT

ACCTATGTTGGCGGCCCCAGAAG

>hg19_ct_ARAlincRNAs_9727_ARAlincRNA_0034.21 range=chr1:149576911-149592306 5'pad=0 3'pad=0 strand=+ repeatMasking=none

AGCTACCAGCTTTGAGGGCAGAACCCGCGTGGCCCGAGCGGGGGTCTAGG

CGAAGGGGCAGTGCGTGGTATTTCCAGCCCCACGTAGTGCCCAAAGCCTG

CAAGCACGCCTCCTGGGTTCCAGCAGCGGCGGCAGAGCGGGGTTGGTTGG

ACCCACCAGCTTGATAATCCCCCTGATTCCCAGACTCCTTGGAATAGCTC

CACAGCTATCAGTGGTTCTGCCACAGGTTCAGAGCCTTCCCAGTACTGCC

TTCTTATTTGAGAGGTGTGCTAAGAGCTATAAAGCAGAGGCTTCAATTGT

ACACAATTGGGAAGTTTAGGCAAAAGTCATTTCTTCCCTATATTTTGTCA

TGCTTATCTCCTGTCTCTTTCTGTTTTACAGATTAGCAATAAACTCCTTA

AAACCCAAAGGTTTGGGCTTCTGTTCCTTTCACTTGCAGTCAGACATGGA

GTTAGTGGTAGAAGAAACAGAAGGGGTAACCTGCATGGTGACAGCTACTG

AGGGGATGGATAGGAAAGCAGGCTGAGTCCCTGGGGCCAGTGGTTACCAA

AGCCAAGGAGAGAGCAAGGGGAGCCCAGTGGGCCTGGCCATGGACTGCTC

TGGAATTCCGAGTGTGAACTTTCAGCCAAGAAGGTAGTGTGAAAATATTA

CTGTGAGGTTTTAAAAGTACACAAATAACAATTGTTTTTTGTAAAAAGAA

AAAAAAAAAGTTAGAAAAAGAGATATGCCAAAAAGAAGAAAGTAAAAAAT

ACATGACTTCTGTCTTCTGTTAACATATTAAAATTTATGCTTTTAGATTT

TTATGTGTCTATATACATGTAAAATATATATGCATCCCCAAATTGTATTA

TACTCTTTTGAGACCTCACATTTTTTTCACCCAAGAATATAATATTAATT

TCTTTCCTTTAGAAAAGACATGAGACTAAAGAATGTTTATTGAGTTGAGA

GTTTCCCAATCAAGGAGTATGACTCTGTATAATAATAAATCTAATGTTTT

ATTAGAAATTATAATAAAATTAGAAATTTAATATTTGGCAGGATGTGAGT

TGCCTGCATATGTACACCCTTTATTATATGACTAGACACTCTTTTGTGTG

GTCATGGATATATTGGCATCTTTTGAAGTGTATCCTTTTTGTTTCAGGAG

CTTCTGTCTAGATGAATAGCAAGTGTGACACTGATAATAGTAATCGAATA

TTCCTCCAAGTACAGGCTCCTTCCTCTTTCTCCTGGACCTTTGCACATGC

TGCCTGGAAAACTCACCCACAACCTACTTTCCATCCTCTCCTTCGGCTCA

TCCCTGCTCAGCCCTCTTTGTTGAGATATTTGCCAAGCCAGATTCTCTGA

GAAGCCTTTCCTGGCATCTTCGTCTATATAATTCTGCTAGTTGTTTCTAA

AACTATGTTGTTAGTGTATATTGTAATCACCTATTTATGTCTGTTTGACT

GTGAGCCTCTTGGAGATGGGAATTCTCTCCTATGCATTGCGGTTTCCCCA

GCCCCTAGCACACTGTCTAGCCCAGTATTCCTTCTCAGTAAGTAGGTGTT

GAATGACTTACAAAATGAATGCTAAAAGACCTGGTAGACAAGAAGGAATT

TCTTAGCCTAAATGACCAGATGTGGAGAAGATACTCAAACACTTCAGGGT

CCCCAATCTGGTGATGTCACTTGCAGTGGAATTTCATATGAATCCAGTGT

GACAGAAGTTGCCTAGTCAAGTTGTAAGTCCTCTTCTCTGGGTCACATGT

TCAAATACTTTTAGGGGACAGATAATGCAATATTTAAGATACAGGAAGTG

ATGAGGACTGTGGCACACTGGAGAGTTTGGACTCCAAGAGTAATTAATTC

AGTTGAAAAAATCAACAGCACTCTGTTGATCAAGCAAACTTTCTAAGGGC

CCTAATCCACCTGGGCCTGCAGTATGTAATTCCATAACTCATGGGATGCC

ATCAAGAGAGACAATCTACTTGATTGTTAAAGGGCCTTTGTTTTCTTTGG

AGTTCCCCAGGAAGGAGAAATGTATGTGTGTGCAGGGGTGAGGAGGGGGG

GAGAGTGAAGAAACAATACTATCCCCTTAAAAAGTTTTGTCAGTTTTCAT

TATGAAAGAAGATCAAACGTGCCCACTCCAGAAATCTTGGCATATGCAGT

TATTTTGAAGCCATTCAGCGTTCTTCAAGATGTCACAATGAAAAGAGTGA

TGATTTTTTAAATAAAAAATTAACATGTTTCTCTTGGCAAGTTTGACTGC

AATGCAGTGTGAATATCGCACATGGGACCATTTGTCATACTGTCAGATGG

CAATACATAGATGGATAGAATTTTGCTGTTTGATCAAAGCTGTGCCATCG

GAAACTTGTTTTCCCAAGTATGGGTACATTCTCCTCTTTTCCTGAGTGGA

GAAACCTAGATCTGAGCAAAAAGCATGAATCAATACACCTTGGGAGGCAG

GGTGTGAAATATTTGATGCCATTTCCTTTGCTTGTAAATTGTTCATTTCA

TTTGAATATAAATATAAAAGGACTTCTACCAAGAACATGAAACTAACATT

TTGCCAAGCTAAATTTTAATGAATTTATTTGGACGTTTTTATAATCACTT

AATATTTTCAGTTCATGTGCTAATATTAACTCTACTTACTGGAGAGAAAG

AAATGGTATTCAATATGATAACTGCCTTGTTACTGCAGAAATGTAGAAGT

TTGGCATTTTAAGTATCAAATATTTTAAGTCAAAAAATTACCAGTGTCCC

ACAGCACAGAGAGAGAATATATGTTTGCTTGTGTCCCTTTAATCTTTCCC

CTGTAAAATTAAGCCTAGGGGCCTTACACTATTTTAAATTTTCAAAATTA

GGTCATGTAAATGTTGTGTCAGATTTCCAATTCATAAGTAGTATACTTAT

GAATACTGAATAGTGAACATAGGGTACATTTATATCCTGCTGATTGCTTA

GCTGTGGA

>hg19_ct_ARAlincRNAs_9727_ARAlincRNA_0034.29 range=chr1:149577608-149582604 5'pad=0 3'pad=0 strand=+ repeatMasking=none

ATTTCAGATACTCAGACTCCACCTTTACTTTTACCTATGTTGGCGGCCCC

AGAAGTCAGACATGGAGTTAGTGGTAGAAGAAACAGAAGGGGTAACCTGC

ATGGTGACAGCTACTGAGGGGATGGATAGGAAAGCAGGCTGAGTCCCTGG

GGCCAGTGGTTACCAAAGCCAAGGAGAGAGCAAGGGGAGCCCAGTGGGCC

TGGCCATGGACTGCTCTGGAATTCCGAGTGTGAACTTTCAGCCAAGAAGG

TAGTGTGAAAATATTACTGTGAGGTTTTAAAAGTACACAAATAACAATTG

TTTTTTGTAAAAAGAAAAAAAAAAAGTTAGAAAAAGAGATATGCCAAAAA

GAAGAAAGTAAAAAATACATGACTTCTGTCTTCTGTTAACATATTAAAAT

TTATGCTTTTAGATTTTTATGTGTCTATATACATGTAAAATATATATGCA

TCCCCAAATTGTATTATACTCTTTTGAGACCTCACATTTTTTTCACCCAA

GAATATAATATTAATTTCTTTCCTTTAGAAAAGACATGAGACTAAAGAAT

GTTTATTGAGTTGAGAGTTTCCCAATCAAGGAGTATGACTCTGTATAATA

ATAAATCTAATGTTTTATTAGAAATTATAATAAAATTAGAAATTTAATAT

TTGGCAGGATGTGAGTTGCCTGCATATGTACACCCTTTATTATATGACTA

GACACTCTTTTGTGTGGTCATGGATATATTGGCATCTTTTGAAGTGTATC

CTTTTTGTTTCAGGAGCTTCTGTCTAGATGAATAGCAAGTGTGACACTGA

TAATAGTAATCGAATATTCCTCCAAGTACAGGCTCCTTCCTCTTTCTCCT

GGACCTTTGCACATGCTGCCTGGAAAACTCACCCACAACCTACTTTCCAT

CCTCTCCTTCGGCTCATCCCTGCTCAGCCCTCTTTGTTGAGATATTTGCC

AAGCCAGATTCTCTGAGAAGCCTTTCCTGGCATCTTCGTCTATATAATTC

TGCTAGTTGTTTCTAAAACTATGTTGTTAGTGTATATTGTAATCACCTAT

TTATGTCTGTTTGACTGTGAGCCTCTTGGAGATGGGAATTCTCTCCTATG

CATTGCGGTTTCCCCAGCCCCTAGCACACTGTCTAGCCCAGTATTCCTTC

TCAGTAAGTAGGTGTTGAATGACTTACAAAATGAATGCTAAAAGACCTGG

TAGACAAGAAGGAATTTCTTAGCCTAAATGACCAGATGTGGAGAAGATAC

TCAAACACTTCAGGGTCCCCAATCTGGTGATGTCACTTGCAGTGGAATTT

CATATGAATCCAGTGTGACAGAAGTTGCCTAGTCAAGTTGTAAGTCCTCT

TCTCTGGGTCACATGTTCAAATACTTTTAGGGGACAGATAATGCAATATT

TAAGATACAGGAAGTGATGAGGACTGTGGCACACTGGAGAGTTTGGACTC

CAAGAGTAATTAATTCAGTTGAAAAAATCAACAGCACTCTGTTGATCAAG

CAAACTTTCTAAGGGCCCTAATCCACCTGGGCCTGCAGTATGTAATTCCA

TAACTCATGGGATGCCATCAAGAGAGACAATCTACTTGATTGTTAAAGGG

CCTTTGTTTTCTTTGGAGTTCCCCAGGAAGGAGAAATGTATGTGTGTGCA

GGGGTGAGGAGGGGGGGAGAGTGAAGAAACAATACTATCCCCTTAAAAAG

TTTTGTCAGTTTTCATTATGAAAGAAGATCAAACGTGCCCACTCCAGAAA

TCTTGGCATATGCAGTTATTTTGAAGCCATTCAGCGTTCTTCAAGATGTC

ACAATGAAAAGAGTGATGATTTTTTAAATAAAAAATTAACATGTTTCTCT

TGGCAA

>hg19_ct_ARAlincRNAs_9727_ARAlincRNA_0034.22 range=chr1:149577608-149591237 5'pad=0 3'pad=0 strand=+ repeatMasking=none

ATTTCAGATACTCAGACTCCACCTTTACTTTTACCTATGTTGGCGGCCCC

AGAAGCTTTAAATCATCTAGGAACTTCATATAAAATTTAATTCCAGTTTC

AACTAGTTGTGGAGCATTTGATCCAAAATAAAATGAAAGTCCTCTCTGAA

GCTGTAGAGGAGGCTCAAGAATCAGAACAACCTGAAGTTCTTTAAGCTGT

CAGTTGAAGGACTAG

>hg19_ct_ARAlincRNAs_9727_ARAlincRNA_0034.19 range=chr1:149615886-149616111 5'pad=0 3'pad=0 strand=+ repeatMasking=none

CCAGAAACGCCCCGGTCGGCGACCCAGCCCGAGACGCCTGGGGTCCCAAG

GGAAGCTGAACGCCCGGTGGGCTCCCGGGATGGTTCTTCCGGTTCTTTGT

GCCGCCTTCACCCAGTGAAGGAGCCTGTATCCACCCTGCCCAGTCGCTGT

TGGGCTGCTGCGGAGCTTCCGCTGCCATCTTCGGATCCTGTGTTCCGCCC

GGGGCTCCACCAGGGCAGGGATGGTG

>hg19_ct_ARAlincRNAs_9727_ARAlincRNA_0034.18 range=chr1:149615886-149616643 5'pad=0 3'pad=0 strand=+ repeatMasking=none

CCAGAAACGCCCCGGTCGGCGACCCAGCCCGAGACGCCTGGGGTCCCAAG

GGAAGCTGAACGCCCGGTGGGCTCCCGGGATGGTTCTTCCGGTTCTTTGT

GCCGCCTTCACCCAGTGAAGGAGCCTGTATCCACCCTGCCCAGTCGCTGT

TGGGCTGCTGCGGAGCTTCCGCTGCCATCTTCGGATCCTGTGTTCCGCCC

GGGGCTCCACCAGGGCAGGGATGGTGCAGAATGGGGAAATGGAGAGAGAA

CCTGAAAGAGCCCCAAACTCGAGGACCTATTGCTCCCCAAGAATAACATC

TTCCAGAACTAGACAGAAAACTAG

>hg19_ct_ARAlincRNAs_9727_ARAlincRNA_0034.17 range=chr1:149616221-149629857 5'pad=0 3'pad=0 strand=+ repeatMasking=none

TTGAAGAGAGAATGGGGAAATGGAGAGAGAACCTGAAAGAGCCCCAAACT

CGAGGACCTATTGCTCCCCAAGAATAACATCTTCCAGAACTAGACAGAAA

ACTAGGCGTCTGGGAACCCTGAAATCCTTGGAGTAGATCATCATGACCCT

CTGTGTTCCTTTTGGCAAATGACTTGCTTCCATTGTTTGTTTGTTCAATT

GTCTGTTTGTTAAATAAATAAAACCCTTTTTATGTATCTTTAAAATTACA

TTGGTTCTATTATTTTATGATTACAAACGATGCTGCAGTCATCATTCTTG

TACACTTCTCATTGGCCACTGGTGTATTTCTATAGGGTGGAGGCCTGATT

ACCTGGCACTGGGTCTTACCTCTGGCATTCCTATCAGAGCCTGAACCACA

GACAGTACCTTAGAGTCTAAGCTTAGATACCTTTGTCTTGTTTTTGTGGC

TAAGGATTCTCAGAGTTGCGGGATGCTGATGAAATTTATCCCAAAAAGTG

TGAAGAGTGCAAAGACCTTATAGAATCTGTGCTGGAGGAGGAGGTGCAGT

TTCAGGAGAGGGAGCTGGCCGAATTGCCGAGGTCAGCTGCAAGGCTCCG

>hg19_ct_ARAlincRNAs_9727_ARAlincRNA_0034.16 range=chr1:149626959-149636207 5'pad=0 3'pad=0 strand=+ repeatMasking=none

ATTACCTGGCACTGGGTCTTACCTCTGGCATTCCTATCAGAGCCTGAACC

ACAGACAGTACCTTAGAGTCTAAGCTTAGATACCTTTGTCTTGTTTTTGT

GGCTAAGGATTCTCAGAGTTGCGGGATGCTGATGAAATTTATCCCAAAAG

ATACATGATCCCTTAATTCAGGCTCAGGCTGAAGAACTGACCCACTTACA

ACAGAAGATACAGGAAGGGAGAGGTGTCTGCTACCTTTTCACCCAGCATG

TGAAGAACACAGTCAAGTCTTTTGAGGGCCTTCTCAGGAACACTGACATT

GCCTACTACCAGAGACAGAGATTCTGTGAGCAAATGGTACAAGGAAGCCA

GCTGACAGAGATCCTTGTCAGAAAACTTGCCACAGAATCAACCCCAATGC

TCACTAACAAACTGAGAAGAAAAAAAAGAATTACCCCCAACAACATCTGA

GGAAGCACAACAGGGACAGA

>hg19_ct_ARAlincRNAs_9727_ARAlincRNA_0034.14 range=chr1:149627007-149637707 5'pad=0 3'pad=0 strand=+ repeatMasking=none

CCACAGACAGTACCTTAGAGTCTAAGCTTAGATACCTTTGTCTTGTTTTT

GTGGCTAAGGATTCTCAGAGTTGCGGGATGCTGATGAAATTTATCCCAAA

AAGTCTTGCTCTCTTACCCAAGCTGGAGTGCAGTGACATGATCTTGGCTG

ACTGCAATCTGTGCCTCCTGGGCTCAAGCAATCTTCCCACCTCAGCCTCC

CAAGTAGCTGGGACTACAGGTGTGTGCCACCACACCTGGCTAATGTTTGT

ATTTTTTGTAGAGATAGGGTTTCTCCATGTTGTCCAGGCTGGTCTTGAAC

TACTGAGCTGAAGTAATCCACCTGCTTTGGCCTCCCAAAGTGCTGGGATT

ACAGATATGAGCCACTGTGCCTGGCCTGAGAGTTCTTAGTTTCAATCAGT

AAGAGAATCAGAACTAGCTATGTGGTTCCAGGCTGCCTTGGTTTAATCCC

TTGTCTTGCCTACTTCACCTGGCTCAAGAGGGAAGTCCAGGAGGAAGAAA

TGGATGAAGTCCTGGAGTACTCACTAGATGAACAGTATTTGACTCATTCC

AGCTGCCATGACTCCCACCAGCTTCCCAGCAGCAATGCCTTCCTCTTTGA

TGCACAGGAAGGCCCC

>hg19_ct_ARAlincRNAs_9727_ARAlincRNA_0034.12 range=chr1:149627341-149629857 5'pad=0 3'pad=0 strand=+ repeatMasking=none

ATGGCAGTATCTGCCACCACTTGTTTTGGTCCAAGGGCAGAAATGAGCAT

CCTGGAAACCAATCAGTACTTGCGCTCTGAACTGGAAAAGTGCAAACAGA

ACTTCCAAGACCTCACAGAGAAATTCCTGACATCCAAAGCTACTGCCTAC

TGCCTGGCCAATCACCTGCAGAAATGTATGTGAAGAGTGCAAAGACCTTA

TAGAATCTGTGCTGGAGGAGGAGGTGCAGTTTCAGGAGAGGGAGCTGGCC

GAATTGCCGAGGTCAGCTGCAAGGCTCCG

>hg19_ct_ARAlincRNAs_9727_ARAlincRNA_0034.13 range=chr1:149632456-149636159 5'pad=0 3'pad=0 strand=+ repeatMasking=none

GATACATGATCCCTTAATTCAGGCTCAGGCTGAAGAACTGACCCACTTAC

AACAGAAGATACAGGAAGGGAGAGGTGTCTGCTACCTTTTCACCCAGCAT

GTGAAGAACACAGTCAAGTCTTTTGAGGGCCTTCTCAGGAACACTGACAT

TGCCTACTACCAGAGACAGAGATTCTGTGAGCAAATGGTACAAGGAAGCC

AGCTGACAGAGATCCTTGTCAGAAAACTTGCCACAGAATCAACCCCAATG

CTCACTAACAAACTGAGAAGAAA

>hg19_ct_ARAlincRNAs_9727_ARAlincRNA_0034.11 range=chr1:149632456-149636735 5'pad=0 3'pad=0 strand=+ repeatMasking=none

GATACATGATCCCTTAATTCAGGCTCAGGCTGAAGAACTGACCCACTTAC

AACAGAAGATACAGGAAGGGAGAGGTGTCTGCTACCTTTTCACCCAGCAT

GTGAAGAACACAGTCAAGTCTTTTGAGGGCCTTCTCAGGAACACTGACAT

TGCCTACTACCAGAGACAGAGATTCTGTGAGCAAATGGTACAAGGAAGCC

AGCTGACAGAGATCCTTGTCAGAAAACTTGCCACAGAAAATCACAATGGT

AAGAAAAATGAAGACAGGCAAAAGCCACTGGCTCCCAG

>hg19_ct_ARAlincRNAs_9727_ARAlincRNA_0034.20 range=chr1:149632456-149636930 5'pad=0 3'pad=0 strand=+ repeatMasking=none

GATACATGATCCCTTAATTCAGGCTCAGGCTGAAGAACTGACCCACTTAC

AACAGAAGATACAGGAAGGGAGAGGTGTCTGCTACCTTTTCACCCAGCAT

GTGAAGAACACAGTCAAGTCTTTTGAGGGCCTTCTCAGGAACACTGACAT

TGCCTACTACCAGAGACAGAGATTCTGTGAGCAAATGGTACAAGGAAGCC

AGCTGACAGAGATCCTTGTCAGAAAACTTGCCACAGGCAGCTGACGTCTG

GTGATGATTCACCATATCAGGAACACCCTGTCCAGTGGGCTAAGGCAAGC

CTGCCAGGCCACTTCTCTAAAGACACAACCTGAGGCCTCCTGCAATAATT

CTCACAGAGCATTCTGAACATACATAAAATCACAATGGTAAGAAAAATGA

AGACAGGCAAAAGCCACTGGCTCCCAGGTAACTCTACATGATGAAGGGGC

TGATAATGGACTGACCAAATCTAGAGAGAATTCCAGAAGCAAGGACTCAA

GAGGTTGAAGGTCCCAGATCTAGAAAAGGCAGAAACTGCTGATTGTTCCT

TTTTTTGTTGACTGATTATACAATGTGTCCTTTTAAAAATGGTTCTCTGT

TCTCTTTGCAGTTGTTGTATTG

>hg19_ct_ARAlincRNAs_9727_ARAlincRNA_0034.15 range=chr1:149636123-149636735 5'pad=0 3'pad=0 strand=+ repeatMasking=none

AATCAACCCCAATGCTCACTAACAAACTGAGAAGAAAAAAAAGAATTACC

CCCAACAACATCTGAGGAAGCACAACAGGGACAGACTTGAAGGCACCTGG

GCAGTCCTCTGGTTTTGGAGTAGAGAAAATCACAATGGTAAGAAAAATGA

AGACAGGCAAAAGCCACTGGCTCCCAG

>hg19_ct_ARAlincRNAs_9727_ARAlincRNA_0034.10 range=chr1:149636144-149637707 5'pad=0 3'pad=0 strand=+ repeatMasking=none

ACAAACTGAGAAGAAAAAAAAGAATTACCCCCAACAACATCTGAGGAAGC

ACAACAGGGACAGACTTGAAGGCACCTGGGCAGTCCTCTGGTTTTGGAGT

AGAGGCAGCTGACGTCTGGTGATGATTCACCATATCAGGAACACCCTGTC

CAGTGGGCTAAGGCAAGCCTGCCAGGCCACTTCTCTAAAGACACAACCTG

AGGCCTCCTGCAATAATTCTCACAGAGCATTCTGAACATACATATCAGAA

CTAGCTATGTGGTTCCAGGCTGCCTTGGTTTAATCCCTTGTCTTGCCTAC

TTCACCTGGCTCAAGAGGGAAGTCCAGGAGGAAGAAATGGATGAAGTCCT

GGAGTACTCACTAGATGAACAGTATTTGACTCATTCCAGCTGCCATGACT

CCCACCAGCTTCCCAGCAGCAATGCCTTCCTCTTTGATGCACAGGAAGGC

CCC

>hg19_ct_ARAlincRNAs_9727_ARAlincRNA_0035.1 range=chr1:150038695-150039082 5'pad=0 3'pad=0 strand=- repeatMasking=none

CGCTCTGAGCAATTGGACTCTGCCAGCTCTAACTCCGGCGGGACTCTGAA

ACTCAGGCAAGAGCCGAGGTGACTGGGTCCTTTGAAATGGGTAGGGGACA

CGTATCCGGTAGAACTAAAACTAGCGTGGGGATTTGAAGCCAAAACTCTC

AAGTTTTTACTGAATCTTAAAACCATCAACTTCCAAATGTCTGCATTTGT

CTTTACACCACACAGGTCTCCCCTCTATCATGGCATTTGTCGAAAAGGAG

GGTAGATGTTTGTTTTTCGCACCTTTACTTTGGGGAATTGTAAAATCAGG

AAGATAATAAGAAACAATTGTTAGAAATCAGTAACTAAAAATGCTTATGA

ATATTTCCACCAAAATAATGACCAGTGTTTGTGAGGTC

>hg19_ct_ARAlincRNAs_9727_ARAlincRNA_0036.3 range=chr1:150493823-150505262 5'pad=0 3'pad=0 strand=+ repeatMasking=none

TTCCACTTGTTCTACACCCTCTCCAATTCTTGAGGTTGTCAATGTGGTTA

GCCATTCTGATATTCACTGAGTGCATAGTGTGTGCGCCAGGCCCTGTTCT

GGGAGATGACAGGCAAGGCCACTGACTTCGTGGAGCTCGCCCCCTAACGG

TGAAGACAAACAGTGACTGACTAAGGAAAAAAAAAAAAG

>hg19_ct_ARAlincRNAs_9727_ARAlincRNA_0036.1 range=chr1:150504637-150509813 5'pad=0 3'pad=0 strand=+ repeatMasking=none

CCTCCCAAAGTGCTGGGATTACAGGCATGAGCCACCGCACCCGGCCAGCC

TTTGCTTTTTTAAAGTTGGTTTTCCTTTATTTCCACAAGACCCAGGAACG

AAAGCGCAGGGAGCAGAGGCTGGACCGTCCTCTGTCAGTTCCCTCGTTCC

CCTGGAGTGGAGCTCTGAGCCCTGGGACCACAGAGTTGGCTGTTACACTG

GAAACACTGATCAGACCTTTGGACTCAGCTGAGATATTCACTGAGTGCAT

AGTGTGTGCGCCAGGCCCTGTTCTGGGAGATGACAGGCAAGGCCACTGAC

TTCGTGGAGCTCGCCCCCTAACGGTGAAGACAAACAGTGACTGACTAAGG

AAAAAAAAAAAAGAACCCTGACGCTTGGCATGGTCCATGGCCCCCTCCAC

ACAGCCAGTTGGCCAGGGAGCCGAGGCAGCCTGTAAACGCTCTGCCTGGA

GCTGCTCGTAAACAAAAACAGAGTGATCCACAAAAGGGCCCCAGAGTCTG

GCACTGAATGGAATCCTACCTTCGGTCCAGCAACTGGAGAACGAAAGTCC

ACATCCTGATGACTGGTTCTGCACCTGGGGCTTAGAGCACATAGCAAGGG

GCTGAGAAGCAGCCACCACATAATCAGTGACTGCAATTTCATCACACAGT

CCTCCATGCCAGGCCCTGTGGGGAAACCAAGATATGTAAGGCAAGCCCTG

CTCTTCGGAATTTCTAATCTCAGTACTGAAAAGTTAATAACATAAGGACG

CAGCCTATCTGTGGTAACACCATGGCATGAGCTGTGTGGATTCCAAGTGC

TGAATGAAGCTGGAAGGGCAGAGGTCACATCGGGCCAGTATGTGCTTCTT

GGAAGAGGTGGCATTTGAGTCACTTGAAGAATGAGCAGGAGATGAGGGAT

GGGAAGGAGAAGGCATTCCTGGGATGCCATGAGCAAAGTGTCACAGTTTT

GCATTGATCACTGTCATCCATGTAAGAGCAAGGACAGCTCAGGCAGGTGG

TGGCCATAGAATGGAGGTGGACACATAAATATGTGGAGAAAGAAGGAAGT

CTTTGGGTTAAAAGGCCAAGACTTGACACCTGTGAGGACCTGGAATCTAA

CCCTGTGTTTGTCTGTAAACCCAAGGCAAGTCACCCTTCTTCCGAGGCAC

AGTTTCTTCACCTGTAAAATGAAGGGACTGGGCAAGACGGTTTCTAAAGT

TCCTAGGGCCAGGTGCAGTGGCTCACGCCTATCTTCACAATACTTTGGGA

GGCTGATGCAGAAGGATCACTGGAGCTCCCTCAAGACCAGCCTGGGGAAC

ACAGGAAGAACCTTGTCTCTACAAAAAATTAAAAATATTAGCTGGACATG

GTGGCAGGACCTGTGGTTCCAGCTACTCAGGAGGCTGAGTGGGGAGGATA

TTTTGAACCTGGGAGGTCGAGGCTGCAGTGAGCTGTGATCACACCACTGC

ACTTCAGCCTGGGCAATAAATAAATAAACAAGTAACAAAGTTCCTTACTA

CTTTAGAATCAGGGTAGAAAGGAGTGAAGATGACTTTAGGATTTTGAAGA

ACAGGCCCAGCAGCAGGAGGTGAGGAGTCTGGAGAACAGCAGATCTGGGG

AGAGGCCTTCAGCTTTAGATAAAATGCGTGGACCCCTTCACGACTCTTCC

TTCTGCTGGGGTCCTCCAGCGCTGCCACAGGCTGACCCGTTCTCAGACTT

GCATGGGACGTGTGTTGCCTCCCTACGTCTCTCCCTGAGATGTGAGCCTG

GCTACATCAAGGCCAGAGCAATGAGAGGCAGTGGCCCTTGCCCCAGCTTT

TCCCCAATGAGGAAGCACCATCATCACCAACACCATCTTGCCACGATAGC

ACTGGAGAGCCTGCAGGTGAAGGTGGGGATCCCCTAAATTCACAATTAAG

TTCCTTTACCCCAGGGAAATGGGCACTGGAAACAGAAACAGGCTGTTATT

GAGAAATCAATTAACATATGGTCCCTGCATACTTCTGTTTGAGGGCCCCA

ATTAGCTCATACCTGGTTCACAGGCAAACTTTCC

>hg19_ct_ARAlincRNAs_9727_ARAlincRNA_0036.2 range=chr1:150504726-150509813 5'pad=0 3'pad=0 strand=+ repeatMasking=none

ACCCAGGAACGAAAGCGCAGGGAGCAGAGGCTGGACCGTCCTCTGTCAGT

TCCCTCGTTCCCCTGGAGTGGAGCTCTGAGCCCTGGGACCACAGAGTTGG

CTGTTACACTGGAAACACTGATCAGACCTTTGGACTCAGCTGAGATATTC

ACTGAGTGCATAGTGTGTGCGCCAGGCCCTGTTCTGGGAGATGACAGGCA

AGGCCACTGACTTCGTGGAGCTCGCCCCCTAACGGTGAAGACAAACAGTG

ACTGACTAAGGAAAAAAAAAAAAGAACCCTGACGCTTGGCATGGTCCATG

GCCCCCTCCACACAGCCAGTTGGCCAGGGAGCCGAGGCAGCCTGTAAACG

CTCTGCCTGGAGCTGCTCGTAAACAAAAACAGAGTGATCCACAAAAGGGC

CCCAGAGTCTGGCACTGAATGGAATCCTACCTTCGGTCCAGCAACTGGAG

AACGAAAGTCCACATCCTGATGACTGGTTCTGCACCTGGGGCTTAGAGCA

CATAGCAAGGGGCTGAGAAGCAGCCACCACATAATCAGTGACTGCAATTT

CATCACACAGTCCTCCATGCCAGGCCCTGTGGGGAAACCAAGATATGTAA

GGCAAGCCCTGCTCTTCGGAATTTCTAATCTCAGTACTGAAAAGTTAATA

ACATAAGGACGCAGCCTATCTGTGGTAACACCATGGCATGAGCTGTGTGG

ATTCCAAGTGCTGAATGAAGCTGGAAGGGCAGAGGTCACATCGGGCCAGT

ATGTGCTTCTTGGAAGAGGTGGCATTTGAGTCACTTGAAGAATGAGCAGG

AGATGAGGGATGGGAAGGAGAAGGCATTCCTGGGATGCCATGAGCAAAGT

GTCACAGTTTTGCATTGATCACTGTCATCCATGTAAGAGCAAGGACAGCT

CAGGCAGGTGGTGGCCATAGAATGGAGGTGGACACATAAATATGTGGAGA

AAGAAGGAAGTCTTTGGGTTAAAAGGCCAAGACTTGACACCTGTGAGGAC

CTGGAATCTAACCCTGTGTTTGTCTGTAAACCCAAGGCAAGTCACCCTTC

TTCCGAGGCACAGTTTCTTCACCTGTAAAATGAAGGGACTGGGCAAGACG

GTTTCTAAAGTTCCTAGGGCCAGGTGCAGTGGCTCACGCCTATCTTCACA

ATACTTTGGGAGGCTGATGCAGAAGGATCACTGGAGCTCCCTCAAGACCA

GCCTGGGGAACACAGGAAGAACCTTGTCTCTACAAAAAATTAAAAATATT

AGCTGGACATGGTGGCAGGACCTGTGGTTCCAGCTACTCAGGAGGCTGAG

TGGGGAGGATATTTTGAACCTGGGAGGTCGAGGCTGCAGTGAGCTGTGAT

CACACCACTGCACTTCAGCCTGGGCAATAAATAAATAAACAAGTAACAAA

GTTCCTTACTACTTTAGAATCAGGGTAGAAAGGAGTGAAGATGACTTTAG

GATTTTGAAGAACAGGCCCAGCAGCAGGAGGTGAGGAGTCTGGAGAACAG

CAGATCTGGGGAGAGGCCTTCAGCTTTAGATAAAATGCGTGGACCCCTTC

ACGACTCTTCCTTCTGCTGGGGTCCTCCAGCGCTGCCACAGGCTGACCCG

TTCTCAGACTTGCATGGGACGTGTGTTGCCTCCCTACGTCTCTCCCTGAG

ATGTGAGCCTGGCTACATCAAGGCCAGAGCAATGAGAGGCAGTGGCCCTT

GCCCCAGCTTTTCCCCAATGAGGAAGCACCATCATCACCAACACCATCTT

GCCACGATAGCACTGGAGAGCCTGCAGGTGAAGGTGGGGATCCCCTAAAT

TCACAATTAAGTTCCTTTACCCCAGGGAAATGGGCACTGGAAACAGAAAC

AGGCTGTTATTGAGAAATCAATTAACATATGGTCCCTGCATACTTCTGTT

TGAGGGCCCCAATTAGCTCATACCTGGTTCACAGGCAAACTTTCC

>hg19_ct_ARAlincRNAs_9727_ARAlincRNA_0037.1 range=chr1:154650042-154650380 5'pad=0 3'pad=0 strand=- repeatMasking=none

CCGAGAGCGAGCGAGGGCTGTGAGGACTGCCAGCACGCTGTCATCTCTCA

ATCCCCCCTCTAAACAGGACACCCCAACTGCTGTTGGGAATTTGGCCGAT

GACCACTCTAGCTACTTCCTGCTGGATAGAGGCGAAGAAGGGGCCCTGTA

GTTGTGGTGTCCTTCAGAGGGGAACTCTCTAGGCCAGGGGAAGTGCCAGT

GGGTCGGTCCAGGGGTCCTCGGTAGAAGTTGTTAGTTGAACTCATTTGGG

GTTCCATTTGTAAGACCATCTGTAGCTTGATGGCCTCAATTCTAGAGGAA

ACAAATTTGACAAGAAGGTTAAAAATACAGGGCCCAAAG

>hg19_ct_ARAlincRNAs_9727_ARAlincRNA_0037.2 range=chr1:154650042-154650380 5'pad=0 3'pad=0 strand=- repeatMasking=none

CCGAGAGCGAGCGAGGGCTGTGAGGACTGCCAGCACGCTGTCATCTCTCA

ATCCCCCCTCTAAACAGGACACCCCAACTGCTGTTGGGAATTTGGCCGAT

GACCACTCTAGCTACTTCCTGCTGGATAGAGGCGAAGAAGGGGCCCTGTA

GTTGTGGTGTCCTTCAGAGGGGAACTCTCTAGGCCAGGGGAAGTGCCAGT

GGGTCGGTCCAGGGGTCCTCGGTAGAAGTTGTTAGTTGAACTCATTTGGG

GTTCCATTTGTAAGACCATCTGTAGCTTGATGGCCTCAATTCTAGAGGAA

ACAAATTTGACAAGAAGGTTAAAAATACAGGGCCCAAAG

>hg19_ct_ARAlincRNAs_9727_ARAlincRNA_0038.1 range=chr1:161369772-161370514 5'pad=0 3'pad=0 strand=+ repeatMasking=none

AGGAAACTTCCGGCGCAGTCAGGGCTGCAGCCAGAGCCCGGAGATAGTTG

GGCAGGGCTCCAGGCGTCCCCGAATGCTCGTCGTGGAGTTTTCCTGATCC

TGTCGGGTCCCTAAGGAACGCTTGGCGGGGAGCGTGGACGCCCTGGGGCC

AGGCCACCGCGGTCACTGAGAGCCACCCCCCCGGAAACTGGAGAAAAACG

GAGTAGAAGAGACCTAGGGGACAGACTTGGCCGGGTCAAGGGAAACCAGC

TCATATTTGGAACAAAATACGCCTTATGTTTGCCCAGCACTGAATTTAGA

AGCATTTTCACATTCTCGAGTTTAACTAGTTTTGCAACAGTGCCACGAGC

AAATTATTAAACGTGTTTTAAAGATGAAGAGACTGAAGCTAGGTAAATTG

GACTTGGGTAAGATGGAAGCGCTACTTAACGTTAAGGAGAGTCTGCGACC

CTGATGTTCTCAGGCTGTATCTCGCTTAGTGGGACTACACCTTATTATTG

TCGGAGGAAACAAATGAAGAGGAAAGAGACCGTGAAGAGTATTCCTGGAG

GGACTCAAGATACCGCTGTAGGTAGAACCCCAAGATAGCTGTGTAGAGTG

ACAGCTGATGTCGAATGACTTCATCTTTTCCACTGCTGCGGGTGAAATTA

TCTGAGGAGGTAGGAAGAGGAGATGGTTTAGGGATGCCATATGACAAAAG

CTTTGGAAAATTAAGAAGAGGCCGGGCGCGGTGGCTCATGCCT

>hg19_ct_ARAlincRNAs_9727_ARAlincRNA_0039.1 range=chr1:167138325-167139808 5'pad=0 3'pad=0 strand=+ repeatMasking=none

ACTCTCTCTCCTCAGTGATCTCACTCCCTCAATTCACTTCACACATCACT

TTACAGTGACGCCATCCTTGGACTCCCTAGCAAAATCCACCCCCTTCCGT

CACTCGCTAATTGCTTAACCAGTTCTACTTTCCTTCATAGCAGTGATCAC

CACCTAGAATATCACACATTTATTGGTTTGTCTTTCCTCAAGGGAATGTA

AACTTCACCAGTACAGGGACTTTTTCAATTTCATTCCCTGCTATATTTTA

AGCATCTAGTACAATGCCTGGCACAGAGCAGGTATATTAGTCAGAGTTCT

CCAGAGAGACAGAATGAATAGGCTATGGACAGACACATAGAAAGATATAA

GAGGAGATTGATTAGGGGAATTGGCTCATGTGATTATGGAGGCTGAGAAG

TACCATGACAGGCTACCTCTACGTTAGAGACCTCAGGAAGCTGGTAGCAT

GACCTAGTCCAAGTCTAAAGACCTCTGAACCAGAGAAGCCGATAGTGTAA

CTCTCAATCCAAGGCCAAAAGCCCAAGAAAACCAGGGAGAGGCCACTGGT

GCAGGTCCTGACCTGACCTAAAGGCCAGTAAGTCTGGAGTTCTGATGTCC

AAGGGTAGGAGAAGAAGGGTGTGCCAGCTCCAGAAGGGAGGGGAAAGAAT

TTGCCTTTCTTCTACCTTTTTGCTCTCTTCAGGCCCCCCTTGATTGGACA

GTGCCTATGTACATTGAGGGCAGACTTCCTCCCTCAGTCCACTGACTCAC

ACGCCGGTCTCCTCTGGAAGCACCCTCATGGACACACCCAGAAACAATGC

TGTACCAGTTCTCTAGGTGTTCCTTAATCTTGTCAAGTTTGGCACCTAAA

ATTAACCATTACAGTAGGTGTTCAGTAAACATTTGTTGAATACATAAATG

GGAGGTCCTGGGGCTCCTTAAGAAAAAACAACAACAACAACAAAAAACCT

CCTCCTTCCCCACCCAATCTCACAATGTCCCTGGAAAGAATGATCATTTT

TATTTCATAACTTTATTTCATGGAGATAAATTGTTTTTATTCACATATAG

TTTGAACCTTCCCCTCCTTTTCATAATTTTTTCCTACCAACACCCTAACC

TCCTATAGAGTGAGAGAGAAAGAGAGCGGGAGGACACGTGCCAGTTCGTG

TGCCATCAAAGGAATCGGCCTTTCTTTCATGGCAATGCCTTTGGAGCAAA

CCTGAAGCCTGTAAGGCAACAACAATGACAGTCTCTTTCCCACCTCCCAG

AAGAGGATGAGAATAAATCCAGCGATAATGGGAAGAGTGTGATGTGAGGC

AGAGCTAACATCACAGAGATCCCAGGGAACATTATTTTTCCCTCTCCTTT

CTAAAGACACCATAAAAAGCTCCAGATTTTTCTTTCTGAGTGGTGACTGC

TTTAAAGAATATAAAATCTGAGATACTGTGAACCCCCACATGATTTCACC

TCTGGCAACTGATGTGAGACACAGTGATGGGACT

>hg19_ct_ARAlincRNAs_9727_ARAlincRNA_0040.1 range=chr1:167140209-167141126 5'pad=0 3'pad=0 strand=- repeatMasking=none

TCTAACACCCACTGGTGTTCTGCAATTGAATTCTGGCATTAACCACCCAG

AGTTAGGGCAGATCTCATGTGACGGTTAATTTTACGTGTCAGTTTAACTG

GATTCAGGGCTGCCTATACGGCTGATGAAGCATTGTTTCTGGGCATGCCT

GTGAGGGTGTGAGTCAGTGGACTGAGAGAGAGCAGAAGATTCACCCTTAA

GGGGGCAGTCACCATCCAATCGTCAGTGGCCCTGGCTGGCACAAGCAGGC

AGAAGAAGGGGGCTTGTTCTCTCTCTCTCCCCCTTTTTCTCTCTCTCCCT

TCTGGGATGGAATGCTTTTTTTCCTCCTGACTTTGGACATAAGACTCAGG

CCCTTCTGCTTTTGGACTCTGGGACTTGTGCCAGTGGCCTTCTGGGGACA

GTCAGGTCTGTAGCCTCAGATGACGGGGGCCTGCACTGTCTGCTTCCCTG

TTCTGAGGCTTCTAGACTTGGACTGAACCATTCATGCTACCAGCTTTGAA

CTGTGCTACCGGCTTCTCCGGTTTTCCAGCTCACAGACAACTATTGTGGG

ACTTCTCCACTTCCATGATTGTGTGAGCCAACTCCCCCTAATAAATCACC

TTTCATATATCCCACTGATTCTGTCTCTCTGGAGGACCCTGAGTAATACA

CCCCACAAATGAAGGGCACAGTCCCCCTCACGAGGGTCTCAACTTCAGAC

ACCAGCCACAAGTTCAGGGGGTCTCCAGGCCACCTACACTTCGGACTAAC

TAGATACTCAGGGATTCTCATGACCTGGTCAGGTTAGATAATTCACTGGA

ACGACTCACAGAACTCAGGAAAGTGCTCTACTTCCAATTACAGACACTTA

CGAGAAAGGATATAGGTCAGGACCAGCCACATGAAGAGACACATGGGGTG

AGGGCTGAGAGAGTCCCA

>hg19_ct_ARAlincRNAs_9727_ARAlincRNA_0041.1 range=chr1:167141473-167142460 5'pad=0 3'pad=0 strand=+ repeatMasking=none

TGGGTACACAGAAGTCAAGAACTGAGGTTTGGGAACTTCTGCCTAGATTT

CAGAGGATGTATGAAAACACCTGAATATCTGGGCAGAAGCTTGCTACAGG

GGCAGAACCCTCATGGGGAACCTCTGCTAGGGCAGTGCAGAAGGGAAATG

TGGGGTCAGAGCCCCCACACAGAGTCCCCACTGGGGCACTTCCTAGTGGA

GCTGTGAGAAGAAGACCACCATCCTGCAGACCCCAGAATGGTAGATCCAC

AACGGCTTGCACCATGTGCCTGGAAAAGCCACAGACACTCAATGCCAGCC

CATGAAAGCAGCCAGGAGTGGGACTGTACCCTGCAAAGCCACAGGAGTGG

AGCTGCCCAAAGCCATGGGAGCCCAACTCTTGCATCAGTGTGACCTGGAT

GTGAGACATGGAGTCAAAGGAGATTATTTCAGAGATTTAAGATTCAGTCA

CTGCCTTACTGGATTTTGGGCTTGTATGGGGCCTGTAGCCGCTTTGTTTT

GGCCAATTTCTCCCATTTGGGACAACTGTATTTACCCAATGCCTGTACCC

CCATTGTATCTAGGAAGTAACTAACTTGCTTGTGATTTTACAGGCTCATA

GGTGGAAGGGACTTGCCTTGTCTCAGATATGACTTTGGACTTGGACTTTC

AAGTTAATGCTGAAATGAGTTAAGACCTTGGGGGACTGTTGGGAAGGCAT

GATTGGTTTTGAAATGTGAGGATATGATATTTGGGAGGGGCTGGGGTGGA

ATGATATGGTTTGGCCATGTCCCCATCCATATCTCATCTTGAATTGTAGC

TCCCATAATTCCCACGTTTTGTGGAAGGGACCTAGTAGGGGGTAATTGAG

TCATGGGGCAGGTTTTTCCTGTGCTGTTCTCATGATAGTGAATAAGTCTC

ATGAGATCTGATGGTTTTATAAAAGGGCAATTCCCCTGCACTTGCTCTTT

CTTGCCTGCCACCATGTAAGGTATGCCTTTGCTCCTCC

>hg19_ct_ARAlincRNAs_9727_ARAlincRNA_0042.1 range=chr1:169457386-169458299 5'pad=0 3'pad=0 strand=- repeatMasking=none

GAATCTTTAAAACATTAAACTAAGTGAAAGAAGCTAGACACAAAAGGTCA

CAAATTGTATGTAACCTCCAGAATAGACAAATCCATAGGCAGAAAGCATA

TTAGTGTTTGCCAGGGACTAAGGGGAGGAGGGAACAGGGAGTGACTGCTT

AATGGGTATCAGTTTCCTGTAGGGTGGTGAAAATGTCTTGGAATTAGACA

GAAGTGATGGTTTCACAATATTATGAATGTAGTAATGCCACTGGGTTGTA

CACTTAATTAATGGTTAATTTTATGTTATGTGAATATTGCCTCGATTTAA

AAAATATATTCTATTTACCTTTGTGTTAGATTCCTATTGCTGCACAACAA

ATGAACAAAAATTTAGCGGCTTTAAAATATTACCACATCAGCTTAGCTCA

CAGTTCTGTAGATCCAGGTGGGCTTGAGTGGATTCTCTGCTTACGGTTTC

GCAGACCAAAATTAAGATGGTGACCCGGCTGGGCTCTTCTCGGAAGAATC

CCACTTCCAAGCTCATTTAGGTTTTTAGCAGAATTCAGTTCCTTATGATT

CTGGGACTGATATCCTGATTCTCTGCTGGCTGTCAGCCAGGAGCCAGTTT

CAGTTGCTATAGGATGCCCACATTTCTTGGTATGTAGCCCCCTCCAACAG

CAGCACGTCAAATTCTTCTCATCCTTCAAATCTCTCTGACTTCCTCTTCT

GCTCTTAGCAGACGGAAAATCTCTGCTTTTAAAGGGCTGTTGTGATTAGA

TTGGGCCCACCTGGAAAATCTCACATCTGTCATAAAACGTAACATAATAA

CAGGAGTATTGCCTCATCAAATTCATAGGTTTCACCTCACACTCAAATGG

GTGGGGGTTATACAAGGGCAAGCGTCACTGGGGATCATTCTTAGAATTCT

GCCTATCGCAATGA

>hg19_ct_ARAlincRNAs_9727_ARAlincRNA_0043.1 range=chr1:179850742-179851730 5'pad=0 3'pad=0 strand=- repeatMasking=none

CCGGCCCCTTCCCTCTCGGATGGGGGCCCTGGGGGTGACGTACACACCCC

ATCCTTCCCGCACCGCCTCTGCCCGCCGCCCGTCGCCCGCCATAGTTGTT

GACGTAGCTTTAGTCGATCGCGAAGATGGCTTTATGGACCCTAGGTTCTC

CTGCCGGTGGTGGCGATGGCTTCTCGGGGGTGTTGGGCGGTGGCCGCTGT

GGTCAGGGAGGCAGTTGCGAGTCGCTGGGGCCGCCGCCTGCGTCGTCGCT

GCTGTGTAGCAAACCTGCCTCTGGGCCCATGGTGTGTCTTGAGGGCTGTG

AGAGAGGCAGTGCCTGAGCTGGGTCAGCTGCTGTGGATCGCTCCCGCCTC

CTGGCTGGAGGCACCAAAGAGGAGGAGAAGGGGGAGGAGGACGTCTCTGC

CATCGCCCTTAAGTAAAGGAGGGGTGGGGGCGATAGAAGGATGGCAGGGC

AACTTGCCTGTCCTAAGTCTAGCCGCTCGTGGCAAGTGTTGACTGCGTGT

AGGAGACTCCCGTCCTATCTCCCAGTACCAAGTCTCGGGGAAAAGGAGTG

CGACGAGGGTGGAGAAGACCCAAGTTTCGCTTCTCGGCCCCAGGCTGTTG

CGCAGTTTCCCTGGAATCTTTTTGATCGACTGCTACGTTGACCATGTCCT

TCCCAGTGTCCTTAGCAGGAGTGATGGCCAGAGAGAGAACTTTTGCACAT

GCCATGTTTTCGTGGCAAAAATTTTAGTTTCAAGTACCTTATGAGGGCGG

TTACCTAGTTTTGAATTTTGTCACTTGTTGAGGGTGTACAAAATCCTATC

TAGAGTCATACTCGTTGGATGTATTTTCTTTTCCGAAATGTACCCATAGG

ACACATAACCTTATTCCTGGTCTTTAATATTTCTCGGTTTTATACCAAGC

AGCAGTGTTCGGAAGACCATGTTAGTGCTACATTGTGCACAGTGAGAAAT

GAACAAGTTTTTTAATTAAAGACAGCACTGTAATTTTAA

>hg19_ct_ARAlincRNAs_9727_ARAlincRNA_0043.2 range=chr1:179850742-179851730 5'pad=0 3'pad=0 strand=- repeatMasking=none

CCGGCCCCTTCCCTCTCGGATGGGGGCCCTGGGGGTGACGTACACACCCC

ATCCTTCCCGCACCGCCTCTGCCCGCCGCCCGTCGCCCGCCATAGTTGTT

GACGTAGCTTTAGTCGATCGCGAAGATGGCTTTATGGACCCTAGGTTCTC

CTGCCGGTGGTGGCGATGGCTTCTCGGGGGTGTTGGGCGGTGGCCGCTGT

GGTCAGGGAGGCAGTTGCGAGTCGCTGGGGCCGCCGCCTGCGTCGTCGCT

GCTGTGTAGCAAACCTGCCTCTGGGCCCATGGTGTGTCTTGAGGGCTGTG

AGAGAGGCAGTGCCTGAGCTGGGTCAGCTGCTGTGGATCGCTCCCGCCTC

CTGGCTGGAGGCACCAAAGAGGAGGAGAAGGGGGAGGAGGACGTCTCTGC

CATCGCCCTTAAGTAAAGGAGGGGTGGGGGCGATAGAAGGATGGCAGGGC

AACTTGCCTGTCCTAAGTCTAGCCGCTCGTGGCAAGTGTTGACTGCGTGT

AGGAGACTCCCGTCCTATCTCCCAGTACCAAGTCTCGGGGAAAAGGAGTG

CGACGAGGGTGGAGAAGACCCAAGTTTCGCTTCTCGGCCCCAGGCTGTTG

CGCAGTTTCCCTGGAATCTTTTTGATCGACTGCTACGTTGACCATGTCCT

TCCCAGTGTCCTTAGCAGGAGTGATGGCCAGAGAGAGAACTTTTGCACAT

GCCATGTTTTCGTGGCAAAAATTTTAGTTTCAAGTACCTTATGAGGGCGG

TTACCTAGTTTTGAATTTTGTCACTTGTTGAGGGTGTACAAAATCCTATC

TAGAGTCATACTCGTTGGATGTATTTTCTTTTCCGAAATGTACCCATAGG

ACACATAACCTTATTCCTGGTCTTTAATATTTCTCGGTTTTATACCAAGC

AGCAGTGTTCGGAAGACCATGTTAGTGCTACATTGTGCACAGTGAGAAAT

GAACAAGTTTTTTAATTAAAGACAGCACTGTAATTTTAA

>hg19_ct_ARAlincRNAs_9727_ARAlincRNA_0044.1 range=chr1:197870959-197871580 5'pad=0 3'pad=0 strand=- repeatMasking=none

ATGGGCTTTTAAGGCAGCTAACGAGGCTCTGATGAGCTGCCTGGACGGGG

AAGAGCCTGGTGTTTCCACATCTTTGGACTTAAATTCTAAGCTCTTCCTT

CTTCCCCCGCGCCCACCCAGCTGAAAGGTGGACATTTTTTGTTATGTCAG

ACTGGACTTGGGATCATTCAGTATAATCTCCACAACTGTTAAGATCCCAG

AAAGTGATCAAAAGACCCGAGGTTTGTGAATAACCACCTTGGGCCTATCC

AGAAAAGGCTGAGTGTTTCCAGGTAGTATGTGATAGGAATAAAGGTGGAA

GCGGAGCCTTCTTTGCTTGGTTCTTTGGCCCACCCTAGAATAAGGGCCAA

AAGTAAAATCGCACACTGAATAAGAAAAGGTGTTGTGTTCATGTTGCTGA

ATATTTACAAAATCCAACCTAAAACTATTATCTCCTTTCTGATGGATGAA

AGTGATCTTGATACCGTCACATAATTGTATTTGAGAAAATTACGGCAAGC

CCGGGAAGTAATTTCAGGTGTTAGGATTAAATTAACACCACCCCATCTTC

CTACCCCCCGTCTGGGTTTCAAGAAAAGGAGGGCTGAAGAGTACCAATTT

TGTTAGTGCTCTTCAATACTGG

>hg19_ct_ARAlincRNAs_9727_ARAlincRNA_0045.1 range=chr1:201087612-201088941 5'pad=0 3'pad=0 strand=- repeatMasking=none

CAGTCACCATGGGGGCTAAGAGTAGAGGAAAAGCTCTTCCACTGTTTCAG

AGTCACATTTTTATCTAGTAAGTTGCCTAATGCTGATGCAACTGCTATTT

TTAATTCAAAACAACAAAATACATCTAATTGTATTAATACATATTTTATG

TATGTGTACATAGTGAAAGTTTAAGTGATACGAATTGCTTCACAAACACA

GGGAGAAAATTAGTAGATGTTGACAGAAAACTCAGTAGCTTTTCCTAAGG

GGTCTTCTCTTACCCTGAATTTTCCAGGAGACCTAGGGACCCACAGTCCC

CAAAGGGAGCTGTCACAGGAAGTGATGACTTCGACACCCGTGGGGTCGGG

ATGCGAATTTCATCAATCTGTTGCCGCCACTGCGTCTCACCACCACACCG

TGGCGCTATTGATAGCTGGTTCTGCCCAGTCCCATGAACTTAGCCGATGA

GTAGGTTCCCTGCTACTGATAGATCCTCCTGGGGCCTTTGCTCAGGTTTT

CTAAGCTCAGGCAAGGGGGCTCCTGCTGCTTGACTCAGTCTGATGATACT

CCAGTCTGTATCTTCCCAGCAGCAGAACTCCATGGGCCACCCTCCAAACA

CATCTTCAAGGGGGTGCTACCCTCCCAAAAGATGACCGGGGTGACCTTTA

GTTCTTCTCTTAGCAGAAGCTAAATAACAGCATTATAATTGGAATCTTCA

GCGGGACATCCCCTCCAGGAATTATCATTATAATTGTAATTCCTCTCCAG

CAGGAATAATTTACTAAGATCCTTTTGTTAGGTCAGTGTAAACCTCTCTG

TTCCTTTGATCAGATGAGAAGCCACTAGTAGAAATACTGAGGGAAGAGGT

GGGAAACTGAGGTGGTGGATTAACCTTTATTAGTTCATGAATTCCTAACA

GTGGGCCTTTAAAAAGTGACTTACCCTTCATGTGCCTCAAGTTTCTCACC

CGTAAAACCAGACAATATTAGCACCTACCTCTCAAGGATGGCGTGAAAAT

TAAATGAGATGATTCACATCAGGCTCTTAGCATGAGGTCTTGCACATCAT

GAATAATAAGTGTAGCTATGATTATCATTATTATTATTATTTCACTACAA

TGTTTGTTTGCTGTTCTATCCTAGTACCTAGAGCAGTCCCAGGATATCCA

GGACCCTCTGTACATATTTACTGAATGAAAATCACGCCGCTTAATGGTCA

TATGCTACACTGTGTTCCTCAGCTTCTGGTATTCAGTGATAGATGCAGAA

CACAATTCCCTTGCAGCTGTGTACCCCCCTATGGTATCTTGCAGATAGGT

TATGAGTTCTCTTTTTTTTTTTTTTTCTTG

>hg19_ct_ARAlincRNAs_9727_ARAlincRNA_0046.3 range=chr1:207326646-207356068 5'pad=0 3'pad=0 strand=+ repeatMasking=none

TGGCATTCCCAGTGCTTCCTGTGGGACAGGACTGCCATGGGCTTCCCATG

AAAATTCCCAGACCAGTGGGGAGATAGAATGTCCACTTCCAGTTCCCTCC

TTCCATCTTGGAAACTGTGGGTCCAGGGAAGTTCTCTAAAAACACTGAAG

AGGCATCAGATCATGCACTCCCAGAGACCTCCACTTGCACTCCTTGAGGG

CTCCACCCTCGATAGAAAAGGAGAAATCGCAGCTTCACTCGTCTCTAGGC

TGTGGAAAGTCTCCAATTCAACTCTGTTCCAAATGATGCTGGTCACTGTT

TTGTTGGCTACCATTCTTGGTGACTGTGGTCCTCCACCTGAGTTACCATT

TGCTTTTCCAATAAATCCGTTGTATGATACTGAATTCAAAACTGGAACTA

CTCTGAAGTACACCTGCCACCCTGGGCATGGTAAAATCAATTCAAGTCGA

CTGATTTGTGATGCCAAAGACTCGTGGAACTATAGTATCTTTTGTGCAAT

TCCTCCTAGAGAAACGATGCAGAAATCCAGAATTAATCAATGGGATAGTG

GAAGTTAAAAAAGATCTTCTCCTTGGTTCAACCATATAATTCAGCTGCTC

AGAGGGGTTTTTCTTAATTGGCTCAACCACCAGTCATTGTCAGATCCAAG

GTAAAGGAGTTGATTGGAGTGATCCTCTCCCAGAACGTGTATTGCCAAGT

GCGAGCCCCCTCCAGACATCAGGAATGGGAAGCACAGCGGTGGAGATCAA

GAATTCTACACATATGCCTCCTCTGTCACCTACAGCTGCAACCCCTACTT

CTCACTCATAGGCAACGTCTCCATCTCCTGCACCGTGGAGAATGAAACAA

TAGGTGTCTGGAGCCCAAACCCTCCTATCTGTGAAAGTATGTTGCCCAAC

ACCAGATCTGGAGAATATCAGAATCATAAATGAAAGGAGGTATTTCACTG

GTAGATGTGTCTATGCCTATGGAGACTATATTTCATATATGTGTGATGAA

GGCTATTACCCTATTTCTGTTGACGGGGAGAGTTCCTGCCACACAGATGG

CACATGGAAGCCTAAAATGCCAGCATGTGAGTTTGCAGTTACCCTCCCAG

TATTGCCCATGGACACTACAAGGAAGTTATTTTAATAACTCCTTATCCTG

AGGCTACATATGAATGTGATGAAGGATATGTTTTGGCTGGATTTGCTACA

ATCTACTGCAAGTCTTTTCACTGGCAACTTGCACCTCCTCAATGTAAAGC

TCTGTGTCTGAAACCAGAAATAGTGAATGGAAGGCTGTCTGTGGATAAGG

ATCAGTATGTTGAGTCTGAAAATGTTACCATTGAATGTGATTCTGGCTAT

GGTGTGGTTGGTCTCAAAAGTATCACTTGCTCAGAGAAGAGAACCTGGTA

CCCAGAAGTGCCCAGGTGTGAGTGG

>hg19_ct_ARAlincRNAs_9727_ARAlincRNA_0046.4 range=chr1:207326646-207357255 5'pad=0 3'pad=0 strand=+ repeatMasking=none

TGGCATTCCCAGTGCTTCCTGTGGGACAGGACTGCCATGGGCTTCCCATG

AAAATTCCCAGACCAGTGGGGAGATAGAATGTCCACTTCCAGTTCCCTCC

TTCCATCTTGGAAACTGTGGGTCCAGGGAAGTTCTCTAAAAACACTGAAG

AGGCATCAGATCATGCACTCCCAGAGACCTCCACTTGCACTCCTTGAGGG

CTCCACCCTCGATAGAAAAGGAGAAATCGCAGCTTCACTCGTCTCTAGGC

TGTGGAAAGTCTCCAATTCAACTCTGTTCCAAATGATGCTGGTCACTGTT

TTGTTGGCTACCATTCTTGGTGACTGTGGTCCTCCACCTGAGTTACCATT

TGCTTTTCCAATAAATCCGTTGTATGATACTGAATTCAAAACTGGAACTA

CTCTGAAGTACACCTGCCACCCTGGGCATGGTAAAATCAATTCAAGTCGA

CTGATTTGTGATGCCAAAGACTCGTGGAACTATAGTATCTTTTGTGCAAA

GAAACGATGCAGAAATCCAGAATTAATCAATGGGATAGTGGAAGTTAAAA

AAGATCTTCTCCTTGGTTCAACCATATAATTCAGCTGCTCAGAGGGGTTT

TTCTTAATTGGCTCAACCACCAGTCATTGTCAGATCCAAGGTAAAGGAGT

TGATTGGAGTGATCCTCTCCCAGAACGTGTATTGCCAAGTGCGAGCCCCC

TCCAGACATCAGGAATGGGAAGCACAGCGGTGGAGATCAAGAATTCTACA

CATATGCCTCCTCTGTCACCTACAGCTGCAACCCCTACTTCTCACTCATA

GGCAACGTCTCCATCTCCTGCACCGTGGAGAATGAAACAATAGGTGTCTG

GAGCCCAAACCCTCCTATCTGTGAAAGTAAGTCAGAATGATTAGTTCTGC

TTTGATCTCTTTTATTTGTTTAAAAACAAAAGGAAGGCATATTTATTAGG

GAAGATGGAATGATTCAAAGAGAAACTAATCACTGCAATGATGGAGTCAT

TCATTTGTTCAACTAATCTTGTTACATGCTGTATCAGTCAGAATAGCATA

GGCTGTGCTGCAGTAGTAATCAACACCTAAATCTCAAGGCCTTTATACAG

TAAAAGTTTTTTTCAAGTTTCTTCTTAGTGCACTGCTAGCCTGGGAAACT

CCCTAGGGCAGCTGTCACTTATATGGTAATCCAGATTGTTTAGATCTTTG

ACACCTCCATGTCAAAACTTCCACAATTAGTTCAGCGGGAGAAGAGAACA

AGGAAAGTCAGTCTCTGGCTCTTCAGTTCTTTGACCTGGAAGTGACATAT

ATCACTTGCCATTACAGCCTTTTGACCAGAACTAGTTCCTTATTTCTGCC

TAATACAGAATAACCGGGAAACACTGTCTTTTGTTTACCTTACAATAACT

GGAAGTATGGGTGGACACTAACAATGTCAACCATGCATGCCTAAGGAGCA

AACAAAACACCAACACCAGCAGGTAATAAAGGTAATAAAACACAATGAAA

GAAAGTATTTTCCCTTGGCCTCTCTATGGAAGCCAGAAGAGATGTTACGT

CAATATCAGTAATGATTCAGCTTGCTGGTTTAGGACATTATTCTTTACTG

CAAATAAAGTGCCTAAAAACCATAGTATCACATATTGTTTTGACTCAGTA

CAGGACACAGCCAATGCCGGGGTGAGAGAGAGCATAACAGTGAAAAGACT

GCAAGAAGTGGAAAAGGCAGGAGCTGAGGCTGGATAAGTTAGTAGGGGCC

AGGTTACGAAAGCTTTGTGTGTCACTTTCAGGAGTTTGGAATGAAGGAAA

AGGGAAGACATTAAAAGGACAATGTGAAGTGAAACAAAACATAACTAATA

GGCACCTGTTGCAACCTTTCAGCCAGGAAGTGAAAGTGGCTTCAACTGTA

GCCACAGCAGTGAGCCTGGAGGAAAGTTGAGGAAGTATAGAGGAATTAAG

ATGGCAGGATTGACAGGACATGATGACTAACATGATGTGGGGTGAAGGCG

GCTTGTTCTCTGCTTATGGAGGCGCAGCACAGATTTCCCGATACCCTGGC

TGCCTCTGCATGACACCATATCATTACCATGGGTCACTGACCCCCAACCA

AAATCATCCAGGAAATTAGGCCATTTAATGGAAGGATTTGGAGACATTGA

GATTTGGACTTCAGGGTTTAGATATTGCCCATTCTTATTGATGAGAAAGT

TGGAGGACTTTACATGACTAACATGATGGGACCTCATTTTGCTGAGGTTT

CTGGCAACTGAAAGGAAGTATACAAATCTCCAAAATGTTTTCTTTGTTCA

GCGCCTATTAAATATATATTTGTATCAGGCCCTCCTTGAAGCCATGCCAA

CAGTTACAGTCACATCTTTACTGCCATTTCTCTCTGATAACTCAGATGTC

TTTTTGTATGCTTCTTTAGAAATTGTCTGTCGTCGACCACAGATTCCAAA

GGCAATCTTTGTTTCTGGATTTGGACCCCTCTATACTTACAAAGACTCTA

TTATGGTTAACTGTGAGGAAGGTTATATCCTCAGAGGCAGCAGTTTAATC

TATTGTGAAACGAATAATGAGTGGTATCCTTCTGTTCCCTCTTGCATATC

CAGTGAGTATGGACTACGATGGAGTTCCAATATTTGGATCTAAAACTATC

CTGACATATAGGTGTGGCATGCTCACAGGCACAAGCCAACTCTACTCCCA

ATAATCATTTTAAGATAAATTGGTACTTTTCTTGTCAAAAGGAAGATTTT

TTTGTTAATAATCTCTTAGCGCTATTGGAAGAATTCCAAATATATAGTCA

TGAGACCAATGACTTCATAGATATTGTAGACTTATATATTTGCTATAAAA

ATTTTCCAGGAGACAATAGTAAAGTGTTTCAGTCTAACAAAATCAATGTA

TTACGAGAATTTTTTGAACAATGGAAGTAAGATTCCTTTAAGACAGTTCT

TTTTTATAATGAGGCACCTGAAGGTTGTGAGCAAGTGCTCACAGGCAGAA

AACTCATGCAGTGTCTCCCAAGCCCAGAGGATGTGAAAGTGGCCCTGGAG

GTGTATAAGCTGTCTCTGGAGATAAAACAACTTGAAAAAGAGAGAGACAA

ATTGATGAACACCCATCAGAAATTTTCTGAAAAAGAGGAAATGAAGGACT

TATTTTTCCCTTCAAATCAACATACAGAATCTTCACTCATCCACCCTACT

CTTCCTTGA

>hg19_ct_ARAlincRNAs_9727_ARAlincRNA_0046.7 range=chr1:207338841-207356068 5'pad=0 3'pad=0 strand=+ repeatMasking=none

ATGCACTCCCAGAGACCTCCACTTGCACTCCTTGAGGGCTCCACCCTCGA

TAGAAAAGGAGAAATCGCAGCTTCACTCGTCTCTAGGCTGTGGAAAGTCT

CCAATTCAACTCTGTTCCAAATGATGCTGGTCACTGTTTTGTTGGCTACC

ATTCTTGGTGACTGTGGTCCTCCACCTGAGTTACCATTTGCTTTTCCAAT

AAATCCGTTGTATGATACTGAATTCAAAACTGGAACTACTCTGAAGTACA

CCTGCCACCCTGGGCATGGTAAAATCAATTCAAGTCGACTGATTTGTGAT

GCCAAAGACTCGTGGAACTATAGTATCTTTTGTGCAATTTTTCTTAATTG

GCTCAACCACCAGTCATTGTCAGATCCAAGGTAAAGGAGTTGATTGGAGT

GATCCTCTCCCAGAACGTGTATTGCCAAGTGCGAGCCCCCTCCAGACATC

AGGAATGGGAAGCACAGCGGTGGAGATCAAGAATTCTACACATATGCCTC

CTCTGTCACCTACAGCTGCAACCCCTACTTCTCACTCATAGGCAACGTCT

CCATCTCCTGCACCGTGGAGAATGAAACAATAGGTGTCTGGAGCCCAAAC

CCTCCTATCTGTGAAAAAATTGTCTGTCGTCGACCACAGATTCCAAAGGC

AATCTTTGTTTCTGGATTTGGACCCCTCTATACTTACAAAGACTCTATTA

TGGTTAACTGTGAGGAAGGTTATATCCTCAGAGGCAGCAGTTTAATCTAT

TGTGAAACGAATAATGAGTGGTATCCTTCTGTTCCCTCTTGCATATCCAA

ATGGTTGCACTGTCCTACCGGACATTTCCTATGCTTCCTGGGAGAGAAAT

GACTACAACCTAAGTGATCACGAAATATTTGAAATTGGAACTGAGTTGAA

ATATCTATGCAAACCTGGCTATAGACCTGTTTTAGATGAGCCTCTGACTG

TGACTTGTCAGGAAAATTTGACATGGACATCTTCCAATGAGTGTGAGGTA

TGTTGCCCAACACCAGATCTGGAGAATATCAGAATCATAAATGAAAGGAG

GTATTTCACTGGTAGATGTGTCTATGCCTATGGAGACTATATTTCATATA

TGTGTGATGAAGGCTATTACCCTATTTCTGTTGACGGGGAGAGTTCCTGC

CACACAGATGGCACATGGAAGCCTAAAATGCCAGCATGTGAGCCAGGTAA

GAATATTGAGAGAACCTGTCATAATCATTTCCTAGATTCCCCAAATGATT

CCACTTGGTTCCACAAAAGATCGAAGTACTTAAAACTAACTGAATTTGAG

GTTGTTCCTGCAAGAAGCATTGTGCAAAAAGGATCACAGATACACATCAC

TAGCACCATGCAAGGCAGCTATAGAACAAAATATGTTGGAAGACAAACTT

ACAGGAGAAATTTTCTTTTAAGAGAAAACAGTAGAATTGATGAAGTCTTT

CAGTCTCATTTTGTCATTTTCCTGCATGTGTACTGATGTTTTAGTAAAGA

GGGACACTACCTCACAATGCTCCATATTTATAAGTCAAGGGAGAAAACAT

GATTTACATATTGTGTAACAATGGAAGCTCCAGGTTGAAGAAGTGCTCTG

TTAGGGAGAGTCTTGAAGCCTAGTGGCTGACGGTGTCAGCCTTTTCTTTC

CTTGCTTTAACTCAGACCTACTGAACCACCTCTCTGTTGAAATACCACGA

AGGAGAAGAAATAAGCATCTAAGGTGATAAACATCTGTCTCTTATCAGGA

TAAGACACTGAAAATGTTATTATTATGGAAGTATCTTCATTATGAAAGAC

TCTTCACATCAACACGATGGTGATTACTCGTTTGCAGTTACCCTCCCAGT

ATTGCCCATGGACACTACAAGGAAGTTATTTTAATAACTCCTTATCCTGA

GGCTACATATGAATGTGATGAAGGATATGTTTTGGCTGGATTTGCTACAA

TCTACTGCAAGTCTTTTCACTGGCAACTTGCACCTCCTCAATGTAAAGCT

CTGTGTCTGAAACCAGAAATAGTGAATGGAAGGCTGTCTGTGGATAAGGA

TCAGTATGTTGAGTCTGAAAATGTTACCATTGAATGTGATTCTGGCTATG

GTGTGGTTGGTCTCAAAAGTATCACTTGCTCAGAGAAGAGAACCTGGTAC

CCAGAAGTGCCCAGGTGTGAGTGG

>hg19_ct_ARAlincRNAs_9727_ARAlincRNA_0046.2 range=chr1:207338841-207357870 5'pad=0 3'pad=0 strand=+ repeatMasking=none

ATGCACTCCCAGAGACCTCCACTTGCACTCCTTGAGGGCTCCACCCTCGA

TAGAAAAGGAGAAATCGCAGCTTCACTCGTCTCTAGGCTGTGGAAAGTCT

CCAATTCAACTCTGTTCCAAATGATGCTGGTCACTGTTTTGTTGGCTACC

ATTCTTGGTGACTGTGGTCCTCCACCTGAGTTACCATTTGCTTTTCCAAT

AAATCCGTTGTATGATACTGAATTCAAAACTGGAACTACTCTGAAGTACA

CCTGCCACCCTGGGCATGGTAAAATCAATTCAAGTCGACTGATTTGTGAT

GCCAAAGACTCGTGGAACTATAGTATCTTTTGTGCAATTTTTCTTAATTG

GCTCAACCACCAGTCATTGTCAGATCCAAGGTAAAGGAGTTGATTGGAGT

GATCCTCTCCCAGAACGTGTATTGCCAAGTGCGAGCCCCCTCCAGACATC

AGGAATGGGAAGCACAGCGGTGGAGATCAAGAATTCTACACATATGCCTC

CTCTGTCACCTACAGCTGCAACCCCTACTTCTCACTCATAGGCAACGTCT

CCATCTCCTGCACCGTGGAGAATGAAACAATAGGTGTCTGGAGCCCAAAC

CCTCCTATCTGTGAAAAAATTGTCTGTCGTCGACCACAGATTCCAAAGGC

AATCTTTGTTTCTGGATTTGGACCCCTCTATACTTACAAAGACTCTATTA

TGGTTAACTGTGAGGAAGGTTATATCCTCAGAGGCAGCAGTTTAATCTAT

TGTGAAACGAATAATGAGTGGTATCCTTCTGTTCCCTCTTGCATATCCAA

ATGGTTGCACTGTCCTACCGGACATTTCCTATGCTTCCTGGGAGAGAAAT

GACTACAACCTAAGTGATCACGAAATATTTGAAATTGGAACTGAGTTGAA

ATATCTATGCAAACCTGGCTATAGACCTGTTTTAGATGAGCCTCTGACTG

TGACTTGTCAGGAAAATTTGACATGGACATCTTCCAATGAGTGTGAGGAG

GCACCTGAAGGTTGTGAGCAAGTGCTCACAGGCAGAAAACTCATGCAGTG

TCTCCCAAGCCCAGAGGATGTGAAAGTGGCCCTGGAGGTGTATAAGCTGT

CTCTGGAGATAAAACAACTTGAAAAAGAGAGAGACAAATTGATGAACACC

CATCAGAAATTTTCTGAAAAAGAGGAAATGAAGGACTTATTTTTCCCTTC

AAATCAACATACAGAATCTTCACTCATCCACCCTACTCTTCCTTGAGCAC

CATCACTCTCATAAGGGTCTGGGCTAGGAGAACTTTCTTATTACTGCCAG

AGAAAAATGTGTAATTATTGGTAATCTAGCCTTTTGTAGCTCAAATGTCC

AGTTTCCTGGACTTGAAGTGCTGGTTAATTAAATAATTATTTACCAAATA

TCAGTTTGTATTGTCATCTTGGTCAATATGTTGCATAAATATGATACACT

CCAGTACACAAATCCACTGCCTAAAGTTTCTGTAATTAACTTTGCCTCAC

CTTTTTCGTACCTCAGAAAGAATGGAAGCCAGGAAATTCAAACCATGTTC

ATACCATAATCTTTATCTGAATGCCTTTACTCTGAATCCCACATGTGCCT

ACCCTTAACCTACAGAAAAGGATACAAAACTCAAAGGATTTTCTGTAACA

TTACAGACAAAACCATTGTCTGTTTGATGGCCCCTGCCCATTAGTAACTC

ATCGCTTTACCCCTGCACAGAATTCCACTCTTCTCACTTTTAACAGCACT

TTTAACAGATTGGGCTTGCATGCTGCTCAACTTTATAGAAATGGAATCAT

CCATTATGCACTCCTTCATATATGTTTTTTCCTTTAAATATTATATTTGT

GAGATTCATCC

>hg19_ct_ARAlincRNAs_9727_ARAlincRNA_0046.5 range=chr1:207338841-207357870 5'pad=0 3'pad=0 strand=+ repeatMasking=none

ATGCACTCCCAGAGACCTCCACTTGCACTCCTTGAGGGCTCCACCCTCGA

TAGAAAAGGAGAAATCGCAGCTTCACTCGTCTCTAGGCTGTGGAAAGTCT

CCAATTCAACTCTGTTCCAAATGATGCTGGTCACTGTTTTGTTGGCTACC

ATTCTTGGTGACTGTGGTCCTCCACCTGAGTTACCATTTGCTTTTCCAAT

AAATCCGTTGTATGATACTGAATTCAAAACTGGAACTACTCTGAAGTACA

CCTGCCACCCTGGGCATGGTAAAATCAATTCAAGTCGACTGATTTGTGAT

GCCAAAGACTCGTGGAACTATAGTATCTTTTGTGCAATTCCTCCTAGAGA

AACGATGCAGAAATCCAGAATTAATCAATGGGATAGTGGAAGTTAAAAAA

GATCTTCTCCTTGGTTCAACCATATAATTCAGCTGCTCAGAGGGGTTGCC

AAGTGCGAGCCCCCTCCAGACATCAGGAATGGGAAGCACAGCGGTGGAGA

TCAAGAATTCTACACATATGCCTCCTCTGTCACCTACAGCTGCAACCCCT

ACTTCTCACTCATAGGCAACGTCTCCATCTCCTGCACCGTGGAGAATGAA

ACAATAGGTGTCTGGAGCCCAAACCCTCCTATCTGTGAAAGTAAGTCAGA

ATGATTAGTTCTGCTTTGATCTCTTTTATTTGTTTAAAAACAAAAGGAAG

GCATATTTATTAGGGAAGATGGAATGATTCAAAGAGAAACTAATCACTGC

AATGATGGAGTCATTCATTTGTTCAACTAATCTTGTTACATGCTGTATCA

GTCAGAATAGCATAGGCTGTGCTGCAGTAGTAATCAACACCTAAATCTCA

AGGCCTTTATACAGTAAAAGTTTTTTTCAAGTTTCTTCTTAGTGCACTGC

TAGCCTGGGAAACTCCCTAGGGCAGCTGTCACTTATATGGTAATCCAGAT

TGTTTAGATCTTTGACACCTCCATGTCAAAACTTCCACAATTAGTTCAGC

GGGAGAAGAGAACAAGGAAAGTCAGTCTCTGGCTCTTCAGTTCTTTGACC

TGGAAGTGACATATATCACTTGCCATTACAGCCTTTTGACCAGAACTAGT

TCCTTATTTCTGCCTAATACAGAATAACCGGGAAACACTGTCTTTTGTTT

ACCTTACAATAACTGGAAGTATGGGTGGACACTAACAATGTCAACCATGC

ATGCCTAAGGAGCAAACAAAACACCAACACCAGCAGGTAATAAAGGTAAT

AAAACACAATGAAAGAAAGTATTTTCCCTTGGCCTCTCTATGGAAGCCAG

AAGAGATGTTACGTCAATATCAGTAATGATTCAGCTTGCTGGTTTAGGAC

ATTATTCTTTACTGCAAATAAAGTGCCTAAAAACCATAGTATCACATATT

GTTTTGACTCAGTACAGGACACAGCCAATGCCGGGGTGAGAGAGAGCATA

ACAGTGAAAAGACTGCAAGAAGTGGAAAAGGCAGGAGCTGAGGCTGGATA

AGTTAGTAGGGGCCAGGTTACGAAAGCTTTGTGTGTCACTTTCAGGAGTT

TGGAATGAAGGAAAAGGGAAGACATTAAAAGGACAATGTGAAGTGAAACA

AAACATAACTAATAGGCACCTGTTGCAACCTTTCAGCCAGGAAGTGAAAG

TGGCTTCAACTGTAGCCACAGCAGTGAGCCTGGAGGAAAGTTGAGGAAGT

ATAGAGGAATTAAGATGGCAGGATTGACAGGACATGATGACTAACATGAT

GTGGGGTGAAGGCGGCTTGTTCTCTGCTTATGGAGGCGCAGCACAGATTT

CCCGATACCCTGGCTGCCTCTGCATGACACCATATCATTACCATGGGTCA

CTGACCCCCAACCAAAATCATCCAGGAAATTAGGCCATTTAATGGAAGGA

TTTGGAGACATTGAGATTTGGACTTCAGGGTTTAGATATTGCCCATTCTT

ATTGATGAGAAAGTTGGAGGACTTTACATGACTAACATGATGGGACCTCA

TTTTGCTGAGGTTTCTGGCAACTGAAAGGAAGTATACAAATCTCCAAAAT

GTTTTCTTTGTTCAGCGCCTATTAAATATATATTTGTATCAGGCCCTCCT

TGAAGCCATGCCAACAGTTACAGTCACATCTTTACTGCCATTTCTCTCTG

ATAACTCAGATGTCTTTTTGTATGCTTCTTTAGAAATTGTCTGTCGTCGA

CCACAGATTCCAAAGGCAATCTTTGTTTCTGGATTTGGACCCCTCTATAC

TTACAAAGACTCTATTATGGTTAACTGTGAGGAAGGTTATATCCTCAGAG

GCAGCAGTTTAATCTATTGTGAAACGAATAATGAGTGGTATCCTTCTGTT

CCCTCTTGCATATCCAAATGGTTGCACTGTCCTACCGGACATTTCCTATG

CTTCCTGGGAGAGAAATGACTACAACCTAAGTGATCACGAAATATTTGAA

ATTGGAACTGAGTTGAAATATCTATGCAAACCTGGCTATAGACCTGTTTT

AGATGAGCCTCTGACTGTGACTTGTCAGGAAAATTTGACATGGACATCTT

CCAATGAGTGTGAGGTATGTTGCCCAACACCAGATCTGGAGAATATCAGA

ATCATAAATGAAAGGAGGTATTTCACTGGTAGATGTGTCTATGCCTATGG

AGACTATATTTCATATATGTGTGATGAAGGCTATTACCCTATTTCTGTTG

ACGGGGAGAGTTCCTGCCACACAGATGGCACATGGAAGCCTAAAATGCCA

GCATGTGAGCCAGGTAAGAATATTGAGAGAACCTGTCATAATCATTTCCT

AGATTCCCCAAATGATTCCACTTGGTTCCACAAAAGATCGAAGTACTTAA

AACTAACTGAATTTGAGGTTGTTCCTGCAAGAAGCATTGTGCAAAAAGGA

TCACAGATACACATCACTAGCACCATGCAAGGCAGCTATAGAACAAAATA

TGTTGGAAGACAAACTTACAGGAGAAATTTTCTTTTAAGAGAAAACAGTA

GAATTGATGAAGTCTTTCAGTCTCATTTTGTCATTTTCCTGCATGTGTAC

TGATGTTTTAGTAAAGAGGGACACTACCTCACAATGCTCCATATTTATAA

GTCAAGGGAGAAAACATGATTTACATATTGTGTAACAATGGAAGCTCCAG

GTTGAAGAAGTGCTCTGTTAGGGAGAGTCTTGAAGCCTAGTGGCTGACGG

TGTCAGCCTTTTCTTTCCTTGCTTTAACTCAGACCTACTGAACCACCTCT

CTGTTGAAATACCACGAAGGAGAAGAAATAAGCATCTAAGGTGATAAACA

TCTGTCTCTTATCAGGATAAGACACTGAAAATGTTATTATTATGGAAGTA

TCTTCATTATGAAAGACTCTTCACATCAACACGATGGTGATTACTCGGAG

GCACCTGAAGGTTGTGAGCAAGTGCTCACAGGCAGAAAACTCATGCAGTG

TCTCCCAAGCCCAGAGGATGTGAAAGTGGCCCTGGAGGTGTATAAGCTGT

CTCTGGAGATAAAACAACTTGAAAAAGAGAGAGACAAATTGATGAACACC

CATCAGAAATTTTCTGAAAAAGAGGAAATGAAGGACTTATTTTTCCCTTC

AAATCAACATACAGAATCTTCACTCATCCACCCTACTCTTCCTTGAGCAC

CATCACTCTCATAAGGGTCTGGGCTAGGAGAACTTTCTTATTACTGCCAG

AGAAAAATGTGTAATTATTGGTAATCTAGCCTTTTGTAGCTCAAATGTCC

AGTTTCCTGGACTTGAAGTGCTGGTTAATTAAATAATTATTTACCAAATA

TCAGTTTGTATTGTCATCTTGGTCAATATGTTGCATAAATATGATACACT

CCAGTACACAAATCCACTGCCTAAAGTTTCTGTAATTAACTTTGCCTCAC

CTTTTTCGTACCTCAGAAAGAATGGAAGCCAGGAAATTCAAACCATGTTC

ATACCATAATCTTTATCTGAATGCCTTTACTCTGAATCCCACATGTGCCT

ACCCTTAACCTACAGAAAAGGATACAAAACTCAAAGGATTTTCTGTAACA

TTACAGACAAAACCATTGTCTGTTTGATGGCCCCTGCCCATTAGTAACTC

ATCGCTTTACCCCTGCACAGAATTCCACTCTTCTCACTTTTAACAGCACT

TTTAACAGATTGGGCTTGCATGCTGCTCAACTTTATAGAAATGGAATCAT

CCATTATGCACTCCTTCATATATGTTTTTTCCTTTAAATATTATATTTGT

GAGATTCATCC

>hg19_ct_ARAlincRNAs_9727_ARAlincRNA_0046.1 range=chr1:207339940-207357870 5'pad=0 3'pad=0 strand=+ repeatMasking=none

GTGACTGTGGTCCTCCACCTGAGTTACCATTTGCTTTTCCAATAAATCCG

TTGTATGATACTGAATTCAAAACTGGAACTACTCTGAAGTACACCTGCCA

CCCTGGGCATGGTAAAATCAATTCAAGTCGACTGATTTGTGATGCCAAAG

ACTCGTGGAACTATAGTATCTTTTGTGCAAAATGGTTGCACTGTCCTACC

GGACATTTCCTATGCTTCCTGGGAGAGAAATGACTACAACCTAAGTGATC

ACGAAATATTTGAAATTGGAACTGAGTTGAAATATCTATGCAAACCTGGC

TATAGACCTGTTTTAGATGAGCCTCTGACTGTGACTTGTCAGGAAAATTT

GACATGGACATCTTCCAATGAGTGTGAGGTATGTTGCCCAACACCAGATC

TGGAGAATATCAGAATCATAAATGAAAGGAGGTATTTCACTGGTAGATGT

GTCTATGCCTATGGAGACTATATTTCATATATGTGTGATGAAGGCTATTA

CCCTATTTCTGTTGACGGGGAGAGTTCCTGCCACACAGATGGCACATGGA

AGCCTAAAATGCCAGCATGTGAGCCAGTTTGCAGTTACCCTCCCAGTATT

GCCCATGGACACTACAAGGAAGTTATTTTAATAACTCCTTATCCTGAGGC

TACATATGAATGTGATGAAGGATATGTTTTGGCTGGATTTGCTACAATCT

ACTGCAAGTCTTTTCACTGGCAACTTGCACCTCCTCAATGTAAAGCTCTG

TGTCTGAAACCAGAAATAGTGAATGGAAGGCTGTCTGTGGATAAGGATCA

GTATGTTGAGTCTGAAAATGTTACCATTGAATGTGATTCTGGCTATGGTG

TGGTTGGTCTCAAAAGTATCACTTGCTCAGAGAAGAGAACCTGGTACCCA

GAAGTGCCCAGGTGTGAGTGGGAGGCACCTGAAGGTTGTGAGCAAGTGCT

CACAGGCAGAAAACTCATGCAGTGTCTCCCAAGCCCAGAGGATGTGAAAG

TGGCCCTGGAGGTGTATAAGCTGTCTCTGGAGATAAAACAACTTGAAAAA

GAGAGAGACAAATTGATGAACACCCATCAGAAATTTTCTGAAAAAGAGGA

AATGAAGGACTTATTTTTCCCTTCAAATCAACATACAGAATCTTCACTCA

TCCACCCTACTCTTCCTTGAGCACCATCACTCTCATAAGGGTCTGGGCTA

GGAGAACTTTCTTATTACTGCCAGAGAAAAATGTGTAATTATTGGTAATC

TAGCCTTTTGTAGCTCAAATGTCCAGTTTCCTGGACTTGAAGTGCTGGTT

AATTAAATAATTATTTACCAAATATCAGTTTGTATTGTCATCTTGGTCAA

TATGTTGCATAAATATGATACACTCCAGTACACAAATCCACTGCCTAAAG

TTTCTGTAATTAACTTTGCCTCACCTTTTTCGTACCTCAGAAAGAATGGA

AGCCAGGAAATTCAAACCATGTTCATACCATAATCTTTATCTGAATGCCT

TTACTCTGAATCCCACATGTGCCTACCCTTAACCTACAGAAAAGGATACA

AAACTCAAAGGATTTTCTGTAACATTACAGACAAAACCATTGTCTGTTTG

ATGGCCCCTGCCCATTAGTAACTCATCGCTTTACCCCTGCACAGAATTCC

ACTCTTCTCACTTTTAACAGCACTTTTAACAGATTGGGCTTGCATGCTGC

TCAACTTTATAGAAATGGAATCATCCATTATGCACTCCTTCATATATGTT

TTTTCCTTTAAATATTATATTTGTGAGATTCATCC

>hg19_ct_ARAlincRNAs_9727_ARAlincRNA_0046.8 range=chr1:207340677-207347144 5'pad=0 3'pad=0 strand=+ repeatMasking=none

AGAAACGATGCAGAAATCCAGAATTAATCAATGGGATAGTGGAAGTTAAA

AAAGATCTTCTCCTTGGTTCAACCATATAATTCAGCTGCTCAGAGGGGTT

TTTCTTAATTGGCTCAACCACCAGTCATTGTCAGATCCAAGGTAAAGGAG

TTGATTGGAGTGATCCTCTCCCAGAACGTGTATTGCCAAGTGCGAGCCCC

CTCCAGACATCAGGAATGGGAAGCACAGCGGTGGAGATCAAGAATTCTAC

ACATATGCCTCCTCTGTCACCTACAGCTGCAACCCCTACTTCTCACTCAT

AGGCAACGTCTCCATCTCCTGCACCGTGGAGAATGAAACAATAGGTGTCT

GGAGCCCAAACCCTCCTATCTGTGAAAAAATTGTCTGTCGTCGACCACAG

ATTCCAAAGGCAATCTTTGTTTCTGGATTTGGACCCCTCTATACTTACAA

AGACTCTATTATGGTTAACTGTGAGGAAGGTTATATCCTCAGAGGCAGCA

GTTTAATCTATTGTGAAACGAATAATGAGTGGTATCCTTCTGTTCCCTCT

TGCATATCCA

>hg19_ct_ARAlincRNAs_9727_ARAlincRNA_0046.6 range=chr1:207344969-207357870 5'pad=0 3'pad=0 strand=+ repeatMasking=none

TTTTTCTTAATTGGCTCAACCACCAGTCATTGTCAGATCCAAGGTAAAGG

AGTTGATTGGAGTGATCCTCTCCCAGAACGTGTAAATGGTTGCACTGTCC

TACCGGACATTTCCTATGCTTCCTGGGAGAGAAATGACTACAACCTAAGT

GATCACGAAATATTTGAAATTGGAACTGAGTTGAAATATCTATGCAAACC

TGGCTATAGACCTGTTTTAGATGAGCCTCTGACTGTGACTTGTCAGGAAA

ATTTGACATGGACATCTTCCAATGAGTGTGAGCGGTGTCAGCCTTTTCTT

TCCTTGCTTTAACTCAGACCTACTGAACCACCTCTCTGTTGAAATACCAC

GAAGGAGAAGAAATAAGCATCTAAGGTGATAAACATCTGTCTCTTATCAG

GATAAGACACTGAAAATGTTATTATTATGGAAGTATCTTCATTATGAAAG

ACTCTTCACATCAACACGATGGTGATTACTCGTTTGCAGTTACCCTCCCA

GTATTGCCCATGGACACTACAAGGAAGTTATTTTAATAACTCCTTATCCT

GAGGCTACATATGAATGTGATGAAGGATATGTTTTGGCTGGATTTGCTAC

AATCTACTGCAAGTCTTTTCACTGGCAACTTGCACCTCCTCAATGTAAAG

CTCTGTGTCTGAAACCAGAAATAGTGAATGGAAGGCTGTCTGTGGATAAG

GATCAGTATGTTGAGTCTGAAAATGTTACCATTGAATGTGATTCTGGCTA

TGGTGTGGTTGGTCTCAAAAGTATCACTTGCTCAGAGAAGAGAACCTGGT

ACCCAGAAGTGCCCAGGTGTGAGTGGGAGGCACCTGAAGGTTGTGAGCAA

GTGCTCACAGGCAGAAAACTCATGCAGTGTCTCCCAAGCCCAGAGGATGT

GAAAGTGGCCCTGGAGGTGTATAAGCTGTCTCTGGAGATAAAACAACTTG

AAAAAGAGAGAGACAAATTGATGAACACCCATCAGAAATTTTCTGAAAAA

GAGGAAATGAAGGACTTATTTTTCCCTTCAAATCAACATACAGAATCTTC

ACTCATCCACCCTACTCTTCCTTGAGCACCATCACTCTCATAAGGGTCTG

GGCTAGGAGAACTTTCTTATTACTGCCAGAGAAAAATGTGTAATTATTGG

TAATCTAGCCTTTTGTAGCTCAAATGTCCAGTTTCCTGGACTTGAAGTGC

TGGTTAATTAAATAATTATTTACCAAATATCAGTTTGTATTGTCATCTTG

GTCAATATGTTGCATAAATATGATACACTCCAGTACACAAATCCACTGCC

TAAAGTTTCTGTAATTAACTTTGCCTCACCTTTTTCGTACCTCAGAAAGA

ATGGAAGCCAGGAAATTCAAACCATGTTCATACCATAATCTTTATCTGAA

TGCCTTTACTCTGAATCCCACATGTGCCTACCCTTAACCTACAGAAAAGG

ATACAAAACTCAAAGGATTTTCTGTAACATTACAGACAAAACCATTGTCT

GTTTGATGGCCCCTGCCCATTAGTAACTCATCGCTTTACCCCTGCACAGA

ATTCCACTCTTCTCACTTTTAACAGCACTTTTAACAGATTGGGCTTGCAT

GCTGCTCAACTTTATAGAAATGGAATCATCCATTATGCACTCCTTCATAT

ATGTTTTTTCCTTTAAATATTATATTTGTGAGATTCATCC

>hg19_ct_ARAlincRNAs_9727_ARAlincRNA_0047.1 range=chr1:211753057-211754161 5'pad=0 3'pad=0 strand=+ repeatMasking=none

TACTCGGTCCTGTCGGAGGAGGCCGACAGACGCATAAACAAGTGCGACTG

TAACTTGGGACTCTGCTGGACGCCGAGCTGAGGAGTTAGTCCTCCCGAAG

GTCTGCTAGGGGCGTGGACCGAATCTTTGTACATGTTTTGGGAAAGCCAG

TTTCTCCTATCACCATCAACCCCTGGAGACCTTGTGTGAACTCAGGTGTC

TGTCACTGATTATCTAAGGACAGTTCAGGGTACAGACTTCAGGCAGGAGA

AACCTGGGCTCTGCTCCTTGCCTTGTCAATAGCCAGCTGTGTGGCTTTGA

GCAAGTCACCTAATCTCTCTAGACCTGTTTCCCCATCTATAAAGGACTGG

ACAAATGCAACTCTGAAGATCCCCGTCTGACTCCAAAATTCTAAGGCTCT

ATGATTTAACATTTATTTATAGACTACACACGGTGGAATCTTGTGCTGGT

TGCTATGGGGAGGTGGAGGGGCAGTAAGAATACAAAGGAACGAGTTTCTG

TCCAGGTGGAGGTAAAATATAAACACGACATTTTAAAATTACGATGGAAT

ACTCTTGATAAATTGTGCTATTTTAGACTGAATACATAAGCACTGTGTTG

TGGGTGGAGATGAGTTGATCTGGAAAGGAACAGCTTGCCCATCTAAGGTG

GGGTGGTGGCTGGAATCAGAGTGGCTTAAGCTGGACCTGAAAGGCCTCAC

CACAGCCCCTGCGCAGCAGAAGGACCTCCCCACCCACTGCCCTAGATTAC

TACTGGACAAATGCAGTGCCAGCTATTGCCTGGAGATGTGTATACAAGGC

TTGTTTGGAGCTGTTATTTTGCATTGTTTTGGTATTCTTGTAATTCCTAT

GAAATTTTTTTCACTTGTATGTAGTGTGTTTCTAGTCTCCCCATCCAGAC

TGGAGAGCTGGATCCTCACCTTCTGTTTCTGTTTTGCTGGAATATAAAGA

ATGGAGGAAAATTTTCAAGACATTATTAAGGAAAAAATGATAGAATTTGA

TGATTAATTGGGTATTGGGAAGGAGGAAGCTAGGTAGGAGAACAAAGATA

GTTATAATGTTTCTAACCTGACAAGGAAAATACTGCCTTAAACAGGTGAG

GAAGT

>hg19_ct_ARAlincRNAs_9727_ARAlincRNA_0048.1 range=chr1:224138976-224141403 5'pad=0 3'pad=0 strand=- repeatMasking=none

AAGAGTTGGAGGAGGCAGTATGGCAGTATGGTGAGACCCTGTCTCTATTA

TTTTAAAAAATTGACAGGCTTTACCCTGGAAGGCTTATACACAATTTAAA

CACCCCTCATAGTATAAAAAAGTGCCCATTTCACTGCACCTTTGCCAGCA

CAGGGTATTATAATTTAGTAAGTCATTTTTTGTTTGATTATTTTACATAG

ACAAAAGAACTCATATTACTTTACTTGTCACATTTCAACATCTTTCCTCA

GCTTATTAGCTCTATTTCTTTTCTGTCTGTAAATGGTTATTGTTGTTTTG

TTCTTTGAGACAGGGTCTTGCTCTGTCACCAGGCTGGACTGTAGTGGCAT

AATCATGCCTCACTGCAGCCTTGACCTCCCAGGCTCAAACTTCCGCATTC

CGAATAGCTGGGACTACAAGTGTGCACCACCACCCCCAGCTAACTTTTTT

CTTCTTTTGGATAGAGACAGGGTCTCACTGTGTTGTCCAGACCGGTCTCT

AGCTCCTGGCCTTAAGCAATCCTCCTGCATTAGCTTCTCAAATTGCTGGA

ATTTCAGGCATGAGCCACCGTGCCTGGCCTGGGCTAGTCCTATATTCTCT

AGAGTTCTCTTTACTTTGTGCTAGTCAATCTCTCATTATGCTGTTCACCT

GTTATAATGAATAATTCTCTGTATTAAATTTTACCACTTTAAACTTTTGA

GTGGTTTATGCTTCCTGATTGGACTCTGACTAATATGTTAGGAAGGGTCC

CAGGAGATAAACCCACACAGATGGGATTTGGGCAGTGCTGAGCTCTTTGC

CAGTGGGAAATGGGATGCTGGTGATTTCCAGTAGGTGACCTCACAGTGAC

TCAAGCTACCACTTACTGTTGATTGTGACGAAATGCCAGCTGAGGCACAT

GCCTTGGGAGCTAAGTGGTTGCTGCACTTGACCACTGTGAAGACTGGTGT

GGGAAGAAGGGTCGTTTCTGATGCACTTGAGCAGGGGTCCCCAACCCCTG

AGCCATGGAGCCGCAAGGAGCCACACAGCAGGAGGTGAGTGGTGTCGAGT

GAGGGAGTGAGGAAAGCTTCGTCTGTATTTACAGCCACTCCCCTTTGCTC

ACATTCCCGCCTGAGCTCCACCTTCTCAGATCAGCAGCAGCATTAGATTC

TCATAGAACGCACCCTGTTGTGAACCGTGCATGTGAGGGATCTAGGTTGC

GCTGTCCTTAATGAGAGTCTAATACCTATTGATCTGTCACTTCCTCCCAT

CACGCTCAGGTGGGACCATCCAGTTGCAGGAAAACAAGCTTAACACGCCC

ACTGATTCTACATTATGCTCCTACCTCCCGGCAGCCTCTCCAGGCCCAGA

ACTTTCTCCAGTCAGCCTCTACAGACCAAGCTCATGACTCACAATGGCCT

ATTTAGGCCCATACCCTACCTCACGGCAGTCTCCGCAGATGAGCCTACTG

CCTCACAACAGCCTCCACAGGCACAGCTCCATCGTTACAATGGCCTCTTT

AGACCCAGCTCCTGCCTCCCAGCCTTCTCTCCAGGCCCTGAACTTTCTCA

AGTCGACCTCACCAGGCCCAGCTCATGCTTCTTTGCAGCCTCTCCAGGCC

CAACTCCTGCATCTTGGTGGCCCCTCCAGGCCCAGCCTGTGCCTCCCGTC

GGCCTCTACAATCCCAACATCTGCCTCACAGCAGATTCTTCACGCCCAGC

CTCTGCCTCACAGTGGACCCTCCAGACCCAGATGGTGTCTCACTGTGGCA

TCCTCAGGCGAAGCTCCTGCCTTTCAGCAGCCTCTCCAGGCCCAGCTCCT

CCTGCCTCCCAGTGGCCTCTTTCGGCCCAGCCCAGCTCATGCCTCCCGGC

GGCCTTCCCAAGCCCCGCTTTTGACTTTTGGTGGCCTCTGCAGGCCTCGA

CAAGGCCCAGCCTCCTGCCTCCCGAAGGCCTGCACAGGCCCAGCCTCTGC

CTCACAGCGGACTCTCCACGCCCAGCTAGCTCTCGCCTCACTGCGGCCTC

CCCAGTCCAAAGCTCCTGCCTTTCGGCCACTTCGGCAGGTCCAGCTACTG

CCTGCCAGTGGCCTCTTTAGGCCCAGCTCATTCCTCACAACGGCCTTTCC

AGGCCCCGTTTTTCCCTTCCGGCAGCCTCTTGGCTTCTAATTTGTTTATC

TTTTGTGTATAAATCCCAAAATATGGAATTTTGGAATATTTCCACCATTA

TATATTTTGGTCGGTAATTTATTTGGAGTGAGTTTCTGCACCATGCCCGA

ATTTTTTATTTTATTTTCCTTATTATTTGGTGTTAAACAGGTTTAATGAC

AGTCATGGCAACTTTTTGGCACAATGAAAAATATC

>hg19_ct_ARAlincRNAs_9727_ARAlincRNA_0048.2 range=chr1:224139096-224141141 5'pad=0 3'pad=0 strand=- repeatMasking=none

TATTTCTTTTCTGTCTGTAAATGGTTATTGTTGTTTTGTTCTTTGAGACA

GGGTCTTGCTCTGTCACCAGGCTGGACTGTAGTGGCATAATCATGCCTCA

CTGCAGCCTTGACCTCCCAGGCTCAAACTTCCGCATTCCGAATAGCTGGG

ACTACAAGTGTGCACCACCACCCCCAGCTAACTTTTTTCTTCTTTTGGAT

AGAGACAGGGTCTCACTGTGTTGTCCAGACCGGTCTCTAGCTCCTGGCCT

TAAGCAATCCTCCTGCATTAGCTTCTCAAATTGCTGGAATTTCAGGCATG

AGCCACCGTGCCTGGCCTGGGCTAGTCCTATATTCTCTAGAGTTCTCTTT

ACTTTGTGCTAGTCAATCTCTCATTATGCTGTTCACCTGTTATAATGAAT

AATTCTCTGTATTAAATTTTACCACTTTAAACTTTTGAGTGGTTTATGCT

TCCTGATTGGACTCTGACTAATATGTTAGGAAGGGTCCCAGGAGATAAAC

CCACACAGATGGGATTTGGGCAGTGCTGAGCTCTTTGCCAGTGGGAAATG

GGATGCTGGTGATTTCCAGTAGGTGACCTCACAGTGACTCAAGCTACCAC

TTACTGTTGATTGTGACGAAATGCCAGCTGAGGCACATGCCTTGGGAGCT

AAGTGGTTGCTGCACTTGACCACTGTGAAGACTGGTGTGGGAAGAAGGGT

CGTTTCTGATGCACTTGAGCAGGGGTCCCCAACCCCTGAGCCATGGAGCC

GCAAGGAGCCACACAGCAGGAGGTGAGTGGTGTCGAGTGAGGGAGTGAGG

AAAGCTTCGTCTGTATTTACAGCCACTCCCCTTTGCTCACATTCCCGCCT

GAGCTCCACCTTCTCAGATCAGCAGCAGCATTAGATTCTCATAGAACGCA

CCCTGTTGTGAACCGTGCATGTGAGGGATCTAGGTTGCGCTGTCCTTAAT

GAGAGTCTAATACCTATTGATCTGTCACTTCCTCCCATCACGCTCAGGTG

GGACCATCCAGTTGCAGGAAAACAAGCTTAACACGCCCACTGATTCTACA

TTATGGTGAATTCTATAATTATTTTATTATATATTACAGTGTAATAATGG

AAATGAAGTGCCTAATAAATGTGAATGTGCTTAAATCTTTTGGCCCAGCT

CCTACCTCCCGGCAGCCTCTCCAGGCCCAGAACTTTCTCCAGTCAGCCTC

TACAGACCAAGCTCATGACTCACAATGGCCTATTTAGGCCCATACCCTAC

CTCACGGCAGTCTCCGCAGATGAGCCTACTGCCTCACAACAGCCTCCACA

GGCACAGCTCCATCGTTACAATGGCCTCTTTAGACCCAGCTCCTGCCTCC

CAGCCTTCTCTCCAGGCCCTGAACTTTCTCAAGTCGACCTCACCAGGCCC

AGCTCATGCTTCTTTGCAGCCTCTCCAGGCCCAACTCCTGCATCTTGGTG

GCCCCTCCAGGCCCAGCCTGTGCCTCCCGTCGGCCTCTACAATCCCAACA

TCTGCCTCACAGCAGATTCTTCACGCCCAGCCTCTGCCTCACAGTGGACC

CTCCAGACCCAGATGGTGTCTCACTGTGGCATCCTCAGGCGAAGCTCCTG

CCTTTCAGCAGCCTCTCCAGGCCCAGCTCCTCCTGCCTCCCAGTGGCCTC

TTTCGGCCCAGCCCAGCTCATGCCTCCCGGCGGCCTTCCCAAGCCCCGCT

TTTGACTTTTGGTGGCCTCTGCAGGCCTCGACAAGGCCCAGCCTCCTGCC

TCCCGAAGGCCTGCACAGGCCCAGCCTCTGCCTCACAGCGGACTCTCCAC

GCCCAGCTAGCTCTCGCCTCACTGCGGCCTCCCCAGTCCAAAGCTCCTGC

CTTTCGGCCACTTCGGCAGGTCCAGCTACTGCCTGCCAGTGGCCTCTTTA

GGCCCAGCTCATTCCTCACAACGGCCTTTCCAGGCCCCGTTTTTCCCTTC

CGGCAGCCTCTTGGCTTCTAATTTGTTTATCTTTTGTGTATAAATCCCAA

AATATGGAATTTTGGAATATTTCCACCATTATATATTTTGGTCGGT

>hg19_ct_ARAlincRNAs_9727_ARAlincRNA_0049.1 range=chr1:224148819-224150006 5'pad=0 3'pad=0 strand=- repeatMasking=none

TAATGGGATATGGGATGCTGGTGATTTCCAGGAAGTGAGCTCACAATGAC

TCAAGCTGCCACATAGTGTTGATTGTGAAATGCCAGTTGAAGCATATGTC

CTGCGAGCTTGGGGGTGCTACAAGTTGACCACTGCAGCAGTAAAGATGAC

TCTGAAGAATGGCGTGGGATGGATCCTTTCAAATGCACTTGAGCAGCAGT

CTCCAACCACAGGGCCACAGAGCTGGAGCTGGATCTACCATGAAAGACTT

CTGAATCCAGGAAGAGAGACTGACTGGGCAACATGTTATTCAGGTACAAA

AAGATTTGGACTGTAACTTAAAAATGATCAAATAATAGTGCATG

>hg19_ct_ARAlincRNAs_9727_ARAlincRNA_0049.2 range=chr1:224148895-224150032 5'pad=0 3'pad=0 strand=- repeatMasking=none

CCAAGGAGCAGTGCTGAGCTCCTTGCTAATGGGATATGGGATGCTGGTGA

TTTCCAGGAAGTGAGCTCACAATGACTCAAGCTGCCACATAGTGTTGATT

GTGAAATGCCAGTTGAAGCATATGTCCTGCGAGCTTGGGGGTGCTACAAG

TTGACCACTGCAGCAGTAAAGATGACTCTGAAGAATGGCGTGGGATGGAT

CCTTTCAAATGCACTTGAGCAGCAGTCTCCAACCACAGGGCCACAGAGCT

GGAGGCTGGATCTACCATGAAAGACTTCTGAATCCAGGAAGAGAG

>hg19_ct_ARAlincRNAs_9727_ARAlincRNA_0050.2 range=chr1:224151249-224158096 5'pad=0 3'pad=0 strand=- repeatMasking=none

TGAAGATGCTGCACTTCTGGCCTTGAATATGGAGTCACGAGAGATGGGAC

CTCAGTATGTTGCCACGGCTGACCTTGAACTCCTGCACTCAAGGGATTTT

CCTACCCTGGCCTCCCAAAGTATTGGTATTACAGGCATGAGCCATTGTGC

CCACCGTCTCTGGTTCTTAACCTTCTGCCTCCCTCTTCCAGTTTTAAAGA

ATGCTTGTAATTACATGGGCTCTCCTAGATACTCCAGGATAATCTTGTTT

TAAGGTCAGCTGATGAGCAACATTAATTTTATCTGCACTATTAATTCCCC

CTTCCTATGTAATTGTGCTGTGTAACATAGGACATGAGCAATTGGTGGCG

GTGGGGGTTATTACTTTGGCCACCACAGTAACTATTTTATGCCAGGTACT

CAGCTAAGCACTGGTGAATTAAGCATGAATAACACACACTCCCTAATCTC

CATCCATTCATGGGAGGAGCACTTCACCTGCCATGCTCCTGAGAATCTCG

GGAGTCAGAGAAGTCTTCTATGAGGAGGTGATGCCAAAGCGGACAAGTGA

CAGAGGAGTCAAAGCTAGCTAGGAAGGGAGTAGAGGTTTAAGGGGAAGCA

TATTATAAGCAGAGGATATTACCCACTTCAGAGACTCCCAGAGGAGAAAG

AGTGTGCGTTCAAGAGGCAGATGAGGCTCAGTTGGACTCCATAGCAGATG

AAATGGAGAGGGGCAAGCAGTGAGGCTGCCTTGCAAGGCAGGGCAGAGCA

GGGGCTGTTAAGGAGTTTGGACTTAATCCCTGAGGCAAGGAGAAGTGATG

TAAATGGAGGAGTAACATGATGAGATTCATGGATTAGAGACATGGCTCAG

GCTGCTGTAGAGAAGGCACCAGGGAGAGCAGATGGCTCAATGGGTGTGCA

GGAGACCTCTCCCTGAGTTTAGGGAGAGGTTTTTAAAACAGAAGAAGTTT

GAGTAATTTAAATGATGATGGGAAGGAGCTAAAAGTGGGGGATAGGTTAA

AGATACAGGAAAGCAGGAGGAAGAACTGACAAGT

>hg19_ct_ARAlincRNAs_9727_ARAlincRNA_0050.1 range=chr1:224151249-224159470 5'pad=0 3'pad=0 strand=- repeatMasking=none

TATAGATGGGGTCTTCTTATGTTGCCCAGGTTGGTCTCAAATTGCTAGGC

TCAAGTGATTCCACCCACCTCTGCCTCCCACAGTGCTGGGATTACGGGCA

TGAGCCAGCGCATCTGGCCACCTTATTTTCAATTACTGGCTCAATGTAAT

GGCTCCATCTCAGGAACAGCCAATGAAAGAGATGCACAGGACAAGAGATG

GGACCTCAGTATGTTGCCACGGCTGACCTTGAACTCCTGCACTCAAGGGA

TTTTCCTACCCTGGCCTCCCAAAGTATTGGTATTACAGGCATGAGCCATT

GTGCCCACCGTCTCTGGTTCTTAACCTTCTGCCTCCCTCTTCCAGTTTTA

AAGAATGCTTGTAATTACATGGGCTCTCCTAGATACTCCAGGATAATCTT

GTTTTAAGGTCAGCTGATGAGCAACATTAATTTTATCTGCACTATTAATT

CCCCCTTCCTATGTAATTGTGCTGTGTAACATAGGACATGAGCAATTGGT

GGCGGTGGGGGTTATTACTTTGGCCACCACAGTAACTATTTTATGCCAGG

TACTCAGCTAAGCACTGGTGAATTAAGCATGAATAACACACACTCCCTAA

TCTCCATCCATTCATGGGAGGAGCACTTCACCTGCCATGCTCCTGAGAAT

CTCGGGAGTCAGAGAAGTCTTCTATGAGGAGGTGATGCCAAAGCGGACAA

GTGACAGAGGAGTCAAAGCTAGCTAGGAAGGGAGTAGAGGTTTAAGGGGA

AGCATATTATAAGCAGAGGATATTACCCACTTCAGAGACTCCCAGAGGAG

AAAGAGTGTGCGTTCAAGAGGCAGATGAGGCTCAGTTGGACTCCATAGCA

GATGAAATGGAGAGGGGCAAGCAGTGAGGCTGCCTTGCAAGGCAGGGCAG

AGCAGGGGCTGTTAAGGAGTTTGGACTTAATCCCTGAGGCAAGGAGAAGT

GATGTAAATGGAGGAGTAACATGATGAGATTCATGGATTAGAGACATGGC

TCAGGCTGCTGTAGAGAAGGCACCAGGGAGAGCAGATGGCTCAATGGGTG

TGCAGGAGACCTCTCCCTGAGTTTAGGGAGAGGTTTTTAAAACAGAAGAA

GTTTGAGTAATTTAAATGATGATGGGAAGGAGCTAAAAGTGGGGGATAGG

TTAAAGATACAGGAAAGCAGGAGGAAGAACTGACAAGT

>hg19_ct_ARAlincRNAs_9727_ARAlincRNA_0050.4 range=chr1:224151308-224161773 5'pad=0 3'pad=0 strand=- repeatMasking=none

TTAAATGTCTTTTTGTCACTTCTAGCTGGACCTACCATGAAAGACTTCTG

AATCCAGGAAGAGAAACTGACTGGGCAACATGTTATTCAGAGATGGGACC

TCAGTATGTTGCCACGGCTGACCTTGAACTCCTGCACTCAAGGGATTTTC

CTACCCTGGCCTCCCAAAGTATTGGTATTACAGGCATGAGCCATTGTGCC

CACCGTCTCTGGTTCTTAACCTTCTGCCTCCCTCTTCCAGTTTTAAAGAA

TGCTTGTAATTACATGGGCTCTCCTAGATACTCCAGGATAATCTTGTTTT

AAGGTCAGCTGATGAGCAACATTAATTTTATCTGCACTATTAATTCCCCC

TTCCTATGTAATTGTGCTGTGTAACATAGGACATGAGCAATTGGTGGCGG

TGGGGGTTATTACTTTGGCCACCACAGTAACTATTTTATGCCAGGTACTC

AGCTAAGCACTGGTGAATTAAGCATGAATAACACACACTCCCTAATCTCC

ATCCATTCATGGGAGGAGCACTTCACCTGCCATGCTCCTGAGAATCTCGG

GAGTCAGAGAAGTCTTCTATGAGGAGGTGATGCCAAAGCGGACAAGTGAC

AGAGGAGTCAAAGCTAGCTAGGAAGGGAGTAGAGGTTTAAGGGGAAGCAT

ATTATAAGCAGAGGATATTACCCACTTCAGAGACTCCCAGAGGAGAAAGA

GTGTGCGTTCAAGAGGCAGATGAGGCTCAGTTGGACTCCATAGCAGATGA

AATGGAGAGGGGCAAGCAGTGAGGCTGCCTTGCAAGGCAGGGCAGAGCAG

GGGCTGTTAAGGAGTTTGGACTTAATCCCTGAGGCAAGGAGAAGTGATGT

AAATGGAGGAGTAACATGATGAGATTCATGGATTAGAGACATGGCTCAGG

CTGCTGTAGAGAAGGCACCAGGGAGAGCAGATGGCTCAATGGGTGTGCAG

GAGACCTCTCCCTGAGTTTAGGGAGAGGTTTTTAAAACAGAAGAAGTTTG

AGTAATTTAAATGATGATGGGAAG

>hg19_ct_ARAlincRNAs_9727_ARAlincRNA_0050.3 range=chr1:224159276-224161773 5'pad=0 3'pad=0 strand=- repeatMasking=none

TTAAATGTCTTTTTGTCACTTCTAGCTGGACCTACCATGAAAGACTTCTG

AATCCAGGAAGAGAAACTGACTGGGCAACATGTTATTCAGATGGGGTCTT

CTTATGTTGCCCAGGTTGGTCTCAAATTGCTAGGCTCAAGTGATTCCACC

CACCTCTGCCTCCCACAGTGCTGGGATTACGGGCATGAGCCAGCGCATCT

GGCCACCTTATTTTCAATTACTGGCTCAATGTAATGGCTCCATCTCAGGA

ACAGCCAATGAAAGAGATGCACAGGACAAG

>hg19_ct_ARAlincRNAs_9727_ARAlincRNA_0051.1 range=chr1:224388869-224389773 5'pad=0 3'pad=0 strand=+ repeatMasking=none

AGCAAATATTAGGAAAATGACACTCCCTATTTTAATCAAAATATGATCTC

CTTGTAACATACACAATGTAAAGTAGATAATCTTATCCTTATTTTAGGGA

GGAGGAAACTGAGAATCAAGGATGTTAGGTAACTTGCATCAAGTTGGCCA

GCTTGATCTCTGAGTAGTGGCAGAACCAGAACCCGATTTGAACCAGCCTC

ACTCTAAGCCCCAGCCCTTGCCACACCACCACCACCCATCAATTACATAT

CCACAAAGAACCTCCGTCTGGTCACGTATGAAATGAGGGATTGCTGCCGT

CCAGTTGGGAATCCTGGAGTCTGAATGCCCCACCACAGACGGCAGTGATC

AGAAAGATATTCTTCTCTGGGGAGGGCTGGGCCCCATCCACTCCCCAGTC

AACTCCCCACATTCCAGGGAGATGCAGTGAGCTCTTCTCTGCAGCCAGCT

CAGGCTCATGACTGCACTGCCAGGGAAGAGAGTAACATTGCTCCAGACCC

ATTTGTACCTGACTGCTCTAAAGGTTACCTTGGATCTGGGAAAGTTTGCT

GAGTAAATTAATAACATAAGCAAGGACGTAAGAAGAACTTTAACCTACCT

CACAGAATTCTATACTAAGAGGCCAAACTGTTTTTGAGAACCAAAGGACA

ATGCATACAACGAGTCTGGTTATTCCTAGAGAATCCTAACTGGGAGCTGA

CAATATTGATGGGGCTCCTGCCAAGTGCCGGGGCCTGTGCTCATCTCTTT

ATGGACTGTTTCATCCTTACATTGGTATTATCCTCATTTTGCAGCCAAGA

AAATAAACTAATAGGAAGGAATAAGTAACTTGTCTAAAGTCATGCAGCCA

AAATTCTAACCCAATGTCTACAAAGTCCATGACATTGCATTTTTCTTTTT

CTTTT

>hg19_ct_ARAlincRNAs_9727_ARAlincRNA_0052.1 range=chr1:226307482-226308995 5'pad=0 3'pad=0 strand=- repeatMasking=none

CGCGTGGGAACTCTCTTAGCAAGGGCACTGACGACGTCCTACTAACTGCC

TTACGACTGTATTTTAAAGGAAGGTGGGAGGCAGGGCCCCTCATTTCCAA

GTGCACCACCCACCAAAGCTGTGATCTTGTCACTGCTCAATACGAGCCTG

GAAAATGATGTACAGCCCTTCCTTTAAGCGTGCTCGGTAAAAGGAGATCC

ATCCCAACAAGCAGAAGCTGATAATCTGCTTGAAAAGGGATTCCTCCACT

TACGAAATTATTTGCAACGGGATTTTTCTCATTGAGTAAATTGTAGGCAA

ATCACCTGTGAATGAATATTTGTAACAGGCCAGCGGATTTGGAAATGAGT

CTTACTGTAAAATAAGTACTTCCGTGGTAAAAATGGTTCCAGGGCCAAGG

ATGGGCCTGTGGTTCAGCTGGAGCATGCTGCCGGCTCACCTGATGGGCGC

CTAGGACGTTCTCTAACGGGCCTCTGAGCCTGGTTCCCTCCCATTCCCTC

TGCATCCACTCCAGCAGGGAGTCCAGGAGGATTCTACCCTTCCTTGGCCA

GAGGATGCTGAAGCTGGGCCCCTTCCTACCTGTTTCCTCTCTTGTGCCCC

ATCTCCTAGGGGAGATCCTTTGCTCCACTAGGCTCGACTGGAAGAGACCG

GGAGGCCATGCAGGGAGCACCCTCACTGGCCAGGCACCCAAGGTGTGCCA

GGCACGGCCTCCCAGGCTGTAGCTCTACCAGGAGTGGGTTCTCCTTGTGA

AGATTCTGACTTTGCTTGTCCCACCTGGCTTGGGGACTCCCACCCTCGCT

CAACCACAGCATTGCCAAACTCAGTAGGGCATCTCTCTGGACACAGAGGG

ATGGAGGAAATGGCCACTCAGGCTCCAGAACTGCACGGGAATTCTGGGCC

ATATCTTGGCCCACCAGCCTCAGCTCACCCTGGCCACTCAAGCCTCGGCC

TTCCTGGGGCCCCTTGGTTCTGGGGAGGGACCCATTAGTAAATACCATTG

CTCACTTGCTGCTTTTAAATCTCCTGAACGTTGGCGGGGCACAGTGGCTC

ACACTTGTAATCCCAGCACTTTGAGAGGCAGAGGTGGGTGGATCATTTGA

GGCCAGGAGTTCGAGACCAGCCTGGCCAAGACAGTGAAACCCCGTCTCTA

CTAAAAATACAAACATTAGCCAGGCGTAGTGATGTACGCCTGTAGTCCCA

GCTACTTGGGAGGCTGAGGCAAGAGAATCACTTGAACCAGTGAGCAGAGG

TTGCAGTGAGCCAAGGTCGCACCACTGTACTCCAGCCTGGGTGACAGAGT

GAGACCCTGTCTCAAAAAAGCAAACAACAAAAAACAAAACAAAACAAACA

AAAAGCCCAAAAATCAATCTCCTGAACGTTGTGAATTTTTCATTAAATCC

AAAAAGCTGGTGGAACGAGCACAGGCTTTCCACCCTCTCACCTGCAGAAG

AGGCGGAAGCCAGTGCTGGTTTGATGGTGCCCTCTTCATTCCCATTGAGG

TTGGAAAGCTGCCC

>hg19_ct_ARAlincRNAs_9727_ARAlincRNA_0053.1 range=chr1:227969696-227970879 5'pad=0 3'pad=0 strand=+ repeatMasking=none

CATGCGCCACCACACCTGGCTAACTTTGTATTTTTAGTAGAGATGGGGTT

TCTCCATGTTGGTCAGCCTGGTCTCAACCTCTCGACCTCAAGTGATCCGC

CCGCCTCAGCCTCCCACAGTGCTGAGATTACAGGCGTGAGCCACTGCACC

CAGCACTCTGATGTTATTTTTTGTTTGATCTGGGAAATTGTTTTCTGCTT

TTTCTTATTTGGTTTATACTTAGCAGCCTGCAGAAGGAGACCTTGCCCTG

TCCCCTCTGGGGTCCTTCCACTAAGGCCACAGGTGAGTGAGTGCTCCCAG

CCTGTCTGGACACCCCAAGGAGGCCAGTGTTGGCCCTCAAGCCTAGCCCA

AGGCATGCGGACACCATGGCCCCCCCTCCTGTCAGTGCTGGGAGCCGGCA

GCAAGGGCTCACCATGCCTGCAGCCACCGTGCAGACCCCCATCAAGCCTG

GAAGAGGCTGCACCCTGTCCTCAGGACCTCACAGTGTGAGTGGGGTCTCC

TCTGTCCCTGACCCCTCGCTGTTCTCAGGACTTCACAGTGTGGGTGGGGT

CTCCCCTGTCCCTGACCTCTCACTGTCAGCAAGTCCTTGCAATATCAGGT

GAGGCCTTTTGTGTCACTTGACCATTCCTTGTCCACTGGACCTCAAAACT

AACATGATATACAGTAGTAAGAATGTAGAAACTTTCCCACTAAAATCACG

ACCAAGGCCCATACATCCGCTCTCACCATCTCCTTTCAGCACTTTAAGCT

CTAACAGATGGAACAAGGCAAGAAAAGGAAGTTAAAGTTTAATGATACTG

AAAGAAGAAACAAACTCTCTTTGTTCACAAATGACATTTTCCAGGTAGAA

AGTGTAAAAAGGGATCAAGAAAATCCCCTAGAATAATGAACAACTATTCA

AGATTTCAAAATATACAAAAGTTAATCACTTTCCTATATACCAACAATGG

ATGAGTGGATTTGAAGTTAAAAACAAAGTACTTCTACTTTAGCACCCCCA

AACAATAAAATACTTAGGTACAAGTCTAAAAAAAGTGTACTCTTTCTATA

TATATGAGAAAAACTACAAAACCCTGATGAACAAAAGAAGAACCAAATAA

ATGGAGAAAAATTCCATGTTTATGGATAAGAAGACTCAGTATTTTCAACA

TGTTTGTTCCTTCCAGCTTGATCTATAGATTCAA

>hg19_ct_ARAlincRNAs_9727_ARAlincRNA_0054.1 range=chr1:234663637-234666927 5'pad=0 3'pad=0 strand=- repeatMasking=none

CTTTTAGGTTCATCTCTGTGAATAATCATCACAAATATTTACTGAGCATC

CTTCATCTCCTGGAAGCCTCGTGGATTATCTTGCTGAATCCTCACCACAG

CAGCCCGCTGAGGGAGGTGCTATCATCGTCCCTGTTTTTCAAGGGAGAGA

ATCAAGGTTTGGGCAGGGACAAGGTCTTGCTATGTTGCCCAAGCTGATCT

TGAACTCCTGGCCTCAAGCAATCCCAATAGTTCGGTCTCCCAAATTGCTG

AGATTGTGGGTACAAGCCACTGCACCTGGACTTCTTCTGACATTATAAAG

CAAAGAGCATTTTCAAAAGCTGATGCTACTTGTGCATATTCCATATTTGC

TGAGCGCTTACTGTGTCCCCATTAAGTGCCAGGCACAGTTTTCAGCACTG

CTTTAAAGTGTCTACAAGCTTAGAAAGGGCATAGGCATCTCTTGAAATCC

AGTGTTCTAGGGAAAGTTTTTCCTGTGGTTTTGCACAGAACCCACCGCTT

CCTGTCCAAGGTGACAGCTTTAGCTGGGAGAGAAATTAAAAATGCCTCCT

TGGAATAGCTATCTGATGTGTCTAAATATGATTGCTGATGTTAGCAATAG

GACCTGCCTATTAGCAATGGTTTGGGCAACTGTATGTGTGAGGCAGGGAG

AGAAAGAGGAAGAAAATACAGCTCTCACTATTGTTTCCAAATGGCAAGAC

TGGTTTTCCTGCTACCTACTAACCAGTGTAGTTATTAGTGTTACATATCA

CCTCTGCATCTTATTTATGTAATATTCCAAGAAGCTAAAAAAAAAAACCT

CTTTTTTTTTTTTTTTTTACAAATCTTAGATTTTATGAACACTTGCTAAT

AGCCCCCCCCTCCTAGATCGTCATTGCTGTGTAACAAATTGTCATACATT

TAGCAGTTTAAAATACCACACATTTACTCACAGCTTCCATGGGCCACGAG

TGTGGGGACGAGTGACTGGGTCGTCTGCTCAGGGTCTCTTACAAGACTCC

ATCAGCGTTTCAGCTGGGGCTGGGGTCTCATCTGAGGCTCAGGGTCCTCA

CTCCAGGTCACAGGGCAGAATTCATTTCTTTCAAGCTACGGATCTCATGG

CAGCTTGCTTTTTCAAGGCCAGCAAGGGAGAGAGTGAATCTGCTGCTTTT

AGTGTCTGACCTCTCGATCTTCTTTTAAATGGCTCATCTGATTAGGGCAG

ACCCACCCAGGATAATCTCTCTTTTGACTAACTTACAGTCAACTCATTAG

AGATCCTAATTACATCTGCAAAAATCCCCTCACCTTTGCCTGATAATGTG

ACATCATCACGGGGGCAATATCCCATCACTTTTGCCATATACTATTGGCT

AGAAGCAAATTAAGGTCCTGCTACCACTCAAGGGGAGGGGATTACACAAG

GGCATGGGACATCAGGGGTCATCTTAGAATTCTGGCAACCAGAATAAGCT

ACTTGTGGATAGACATGTTGTCATACATATTTTTTGTTCAGTCATTATGC

TTAGCAGAGAAATAGGCACACAGCAGAAAAATAACTGATTGGTACGGAGA

ATTTAGTGATTGGAAACGAATTAATATTGTTAAGACACAAAGTTTCTTGC

AGAGCACACTCTGAAAAAGGTCACTTTTTACCCCTCAATATGTCTTAATG

GGTTGAGGTAGAGAGAAGCGTGGGTAGGTATCAATCACGAGAGCACACAG

GCTAAAAGAAAGAAAAAATGAGCTGGGACATCTAAGTTCAACTTCCACTT

CCTCCTATACAAGCTGGGTGACCTTTTCCTATCTAGAAAGTGGGAGAAGT

AGTTCCTGTCCTCCCTTACAGGGCTGCTGAGCAGGCAGAATGAGATGATG

GGCTGAGAGGGCTTGGAGAGGACAAACTGTGGGTACAAAAGTCTTATTGT

CCTGGCCCGGATTGCTAAGGAGATGGTGGTTAATAATATGCTGCTATCTC

ATGAGGTAAATGCGTGATCACATAGCCAAGAGATGCCTTTTTGTTCTAAT

GATCACTGTAAGACCTTCTTGAGAGACAGTCCAAGTCTCTCCAGCACAAT

GGAAAATTGCATTTTCTATGCACACATTCGAGGGGAAAGAGACCCCTCCA

ACGTTCTTCTCAGTTTTACCCCAAAGTTGAGTTCTGGGTCAAAAATCTCT

CAAGCGTCCTCCACAGCTGACTTCCACAGTCCCCTGTGTGTCTCTGCAAG

GAACACAAAGGAAGTGAGATAGTGGGAAGTCTGGGGCCATCTTCGGTCAC

GTGAGAATTCCCTCTCAGTTAGCCGGGGCGTGCAGCCGCACAGAAGGAAG

GCAGCCACGCAGCTCCAGTCCAGGCTGGGAGAGAGCTTTGTAGATCCATC

TCCCACTGAGCAGGCTTCCCTCCTGCAAGGAGAGACTCTACCGATAATGT

GTTAAGATCAGATTAATGCAGGAAATACACATGAAATAAATAATAACAGT

GTAATAACAGTGGCTGTCATTGCAATGGTTATTTCTGTTAAAAATGTGAG

ATCTACACTACGGATTTCTTAGGATAATTTTGCCCTTACTTAAAAGAAAC

TGACAGATGTACTTTATAAAAATGAAAATAATTTAACAAATAAAGAATTG

GAGAATATTGACTCACCA

>hg19_ct_ARAlincRNAs_9727_ARAlincRNA_0054.2 range=chr1:234663637-234667525 5'pad=0 3'pad=0 strand=- repeatMasking=none

GCAGGGCCGACTGCATTCCCACGGCGACGGCCTCCTGGCCCGCATCATGA

CTGTTGTTTTCCTCGCGGTGGCCATAGCTTTTGGATTTTACATTCAGATT

GGTTCAAGGGGCTTTTTCCTTTCTCTGTTTGAAGGAAGCCACCCTCTCGA

TCCCCAGTCAGCGTGGCGTCCTCTCAGCAACCCGCTCCTGGTCTTGCTTT

GACTCCTGTGCTCCCGGGCTGCCGAGAACACGTCTGGAGCTTTTAAATCA

GGCTTTTAGGTTCATCTCTGTGAATAATCATCACAAATATTTACTGAGCA

TCCTTCATCTCCTGGAAGCCTCGTGGATTATCTTGCTGAATCCTCACCAC

AGCAGCCCGCTGAGGGAGGTGCTATCATCGTCCCTGTTTTTCAAGGGAGA

GAATCAAGGTTTGGGCAGGGACAAGGTCTTGCTATGTTGCCCAAGCTGAT

CTTGAACTCCTGGCCTCAAGCAATCCCAATAGTTCGGTCTCCCAAATTGC

TGAGATTGTGGGTACAAGCCACTGCACCTGGACTTCTTCTGACATTATAA

AGCAAAGAGCATTTTCAAAAGCTGATGCTACTTGTGCATATTCCATATTT

GCTGAGCGCTTACTGTGTCCCCATTAAGTGCCAGGCACAGTTTTCAGCAC

TGCTTTAAAGTGTCTACAAGCTTAGAAAGGGCATAGGCATCTCTTGAAAT

CCAGTGTTCTAGGGAAAGTTTTTCCTGTGGTTTTGCACAGAACCCACCGC

TTCCTGTCCAAGGTGACAGCTTTAGCTGGGAGAGAAATTAAAAATGCCTC

CTTGGAATAGCTATCTGATGTGTCTAAATATGATTGCTGATGTTAGCAAT

AGGACCTGCCTATTAGCAATGGTTTGGGCAACTGTATGTGTGAGGCAGGG

AGAGAAAGAGGAAGAAAATACAGCTCTCACTATTGTTTCCAAATGGCAAG

ACTGGTTTTCCTGCTACCTACTAACCAGTGTAGTTATTAGTGTTACATAT

CACCTCTGCATCTTATTTATGTAATATTCCAAGAAGCTAAAAAAAAAAAC

CTCTTTTTTTTTTTTTTTTTACAAATCTTAGATTTTATGAACACTTGCTA

ATAGCCCCCCCCTCCTAGATCGTCATTGCTGTGTAACAAATTGTCATACA

TTTAGCAGTTTAAAATACCACACATTTACTCACAGCTTCCATGGGCCACG

AGTGTGGGGACGAGTGACTGGGTCGTCTGCTCAGGGTCTCTTACAAGACT

CCATCAGCGTTTCAGCTGGGGCTGGGGTCTCATCTGAGGCTCAGGGTCCT

CACTCCAGGTCACAGGGCAGAATTCATTTCTTTCAAGCTACGGATCTCAT

GGCAGCTTGCTTTTTCAAGGCCAGCAAGGGAGAGAGTGAATCTGCTGCTT

TTAGTGTCTGACCTCTCGATCTTCTTTTAAATGGCTCATCTGATTAGGGC

AGACCCACCCAGGATAATCTCTCTTTTGACTAACTTACAGTCAACTCATT

AGAGATCCTAATTACATCTGCAAAAATCCCCTCACCTTTGCCTGATAATG

TGACATCATCACGGGGGCAATATCCCATCACTTTTGCCATATACTATTGG

CTAGAAGCAAATTAAGGTCCTGCTACCACTCAAGGGGAGGGGATTACACA

AGGGCATGGGACATCAGGGGTCATCTTAGAATTCTGGCAACCAGAATAAG

CTACTTGTGGATAGACATGTTGTCATACATATTTTTTGTTCAGTCATTAT

GCTTAGCAGAGAAATAGGCACACAGCAGAAAAATAACTGATTGGTACGGA

GAATTTAGTGATTGGAAACGAATTAATATTGTTAAGACACAAAGTTTCTT

GCAGAGCACACTCTGAAAAAGGTCACTTTTTACCCCTCAATATGTCTTAA

TGGGTTGAGGTAGAGAGAAGCGTGGGTAGGTATCAATCACGAGAGCACAC

AGGCTAAAAGAAAGAAAAAATGAGCTGGGACATCTAAGTTCAACTTCCAC

TTCCTCCTATACAAGCTGGGTGACCTTTTCCTATCTAGAAAGTGGGAGAA

GTAGTTCCTGTCCTCCCTTACAGGGCTGCTGAGCAGGCAGAATGAGATGA

TGGGCTGAGAGGGCTTGGAGAGGACAAACTGTGGGTACAAAAGTCTTATT

GTCCTGGCCCGGATTGCTAAGGAGATGGTGGTTAATAATATGCTGCTATC

TCATGAGGTAAATGCGTGATCACATAGCCAAGAGATGCCTTTTTGTTCTA

ATGATCACTGTAAGACCTTCTTGAGAGACAGTCCAAGTCTCTCCAGCACA

ATGGAAAATTGCATTTTCTATGCACACATTCGAGGGGAAAGAGACCCCTC

CAACGTTCTTCTCAGTTTTACCCCAAAGTTGAGTTCTGGGTCAAAAATCT

CTCAAGCGTCCTCCACAGCTGACTTCCACAGTCCCCTGTGTGTCTCTGCA

AGGAACACAAAGGAAGTGAGATAGTGGGAAGTCTGGGGCCATCTTCGGTC

ACGTGAGAATTCCCTCTCAGTTAGCCGGGGCGTGCAGCCGCACAGAAGGA

AGGCAGCCACGCAGCTCCAGTCCAGGCTGGGAGAGAGCTTTGTAGATCCA

TCTCCCACTGAGCAGGCTTCCCTCCTGCAAGGAGAGACTCTACCGATAAT

GTGTTAAGATCAGATTAATGCAGGAAATACACATGAAATAAATAATAACA

GTGTAATAACAGTGGCTGTCATTGCAATGGTTATTTCTGTTAAAAATGTG

AGATCTACACTACGGATTTCTTAGGATAATTTTGCCCTTACTTAAAAGAA

ACTGACAGATGTACTTTATAAAAATGAAAATAATTTAACAAATAAAGAAT

TGGAGAATATTGACTCACCA

>hg19_ct_ARAlincRNAs_9727_ARAlincRNA_0054.3 range=chr1:234665688-234667525 5'pad=0 3'pad=0 strand=- repeatMasking=none

GCAGGGCCGACTGCATTCCCACGGCGACGGCCTCCTGGCCCGCATCATGA

CTGTTGTTTTCCTCGCGGTGGCCATAGCTTTTGGATTTTACATTCAGATT

GGTTCAAGGGGCTTTTTCCTTTCTCTGTTTGAAGGAAGCCACCCTCTCGA

TCCCCAGTCAGCGTGGCGTCCTCTCAGCAACCCGCTCCTGGTCTTGCTTT

GACTCCTGTGCTCCCGGGCTGCCGAGAACACGTCTGGAGCTTTTAAATCA

GGGGACAAGGTCTTGCTATGTTGCCCAAGCTGATCTTGAACTCCTGGCCT

CAAGCAATCCCAATAGTTCGGTCTCCCAAATTGCTGAGATTGTGGGTACA

AGCCACTGCACCTGGACTTCTTCTGACATTATAAAGCAAAGAGCATTTTC

AAAAGCTGATGCTACTTGTGCATATTCCATATTTGCTGAGCGCTTACTGT

GTCCCCATTAAGTGCCAGGCACAGTTTTCAGCACTGCTTTAAAGTGTCTA

CAAGCTTAGAAAGGGCATAGGCATCTCTTGAAATCCAGTGTTCTAGGGAA

AGTTTTTCCTGTGGTTTTGCACAGAACCCACCGCTTCCTGTCCAAGGTGA

CAGCTTTAGCTGGGAGAGAAATTAAAAATGCCTCCTTGGAATAGCTATCT

GAT

>hg19_ct_ARAlincRNAs_9727_ARAlincRNA_0055.7 range=chr1:243242159-243251534 5'pad=0 3'pad=0 strand=- repeatMasking=none

CTCGAGCTGTAAAAGCCAAAGGTCCGGTGATGATCCCATACCCTTTTTTC

CAGTCTCATGTTGAAGATTTTTATGTAGAAGGCCTTCCCAAAGGAATTTT

TTTTTTTTTTTTGAGATGGAGTTTTCACTCTTATCGCCCAGGCTGGGGGG

CAATGGCGCAACCTTGCTGGTCACTGCAACCTCTGCCTCCTGGGTTCACT

GGACCTACCATGAAAGACTTCTGAATCCAGGAAGAGAAACTGACTGGGCA

ACATGTTATTCAGGTACAAAAAGACTTCGACTGTAACTCAAAAATGATCA

AATAATAGTGCATGCATCAAGTGCAATGGGAAGCTCTTCTGGAGAGGGAG

AGAAGCTTCCAGTTAAGGTGACATTGAAGCCAAGTCCTGTAAGA

>hg19_ct_ARAlincRNAs_9727_ARAlincRNA_0055.5 range=chr1:243242159-243254816 5'pad=0 3'pad=0 strand=- repeatMasking=none

AAAGTCCTGTAATCTTATGTTCATGGGCGTTTACACAATGGAGTTACTGT

TCATCATGGGGGTACCGTGGACAAGCCCAGGGCTGCCGGCGAGTCATGCC

ATCCTTACACGTTTCTCCTTGTAAGAGCCACCTAAGCTGAAGATTCCCTT

GAGAACAAGTACTGTCCTGCGGTTTCATGGCCCTTCTTCCATTTGTGGTT

CTTGCGAAGTGGAATTTAAATGACATCTTATCAAGATGGATAAACCCTAG

TTTCCCAGTGCTGGAATATAGAAAATGGATGGACAAGTAAATCCCACTCA

GCACCCATAGTCCAGGCATGGGGACCTCAACACACCTGAGCCCCAGACAT

CACCTTTCATTCTGGACCTACCATGAAAGACTTCTGAATCCAGGAAGAGA

AACTGACTGGGCAACATGTTATTCAGGTACAAAAAGACTTCGACTGTAAC

TCAAAAATGATCAAATAATAGTGCATGCATCAAGTGCAATGGGAAGCTCT

TCTGGAGAGGGAGAGAAGCTTCCAGTTAAGGTGACATTGAAGCCAAGTCC

TGTAAGA

>hg19_ct_ARAlincRNAs_9727_ARAlincRNA_0055.6 range=chr1:243242159-243255900 5'pad=0 3'pad=0 strand=- repeatMasking=none

GTAGAGGTGGCCTGCACTCCTCAGCTTGTGCTGCCCGTCTCGAATGACTG

GAGTTTCCTGCTTCTGTCACTACACCTCCCACCCTCTCCCATCACCTGCT

CTGCTCTTACAAGGATCCGAAGAAATGGAATAATCGTATCGCTGATCTAT

GTAAACAAATTGAAGAATTGTCTGAAAGAAAATATGGTATGTCTAAACTG

GAAAAGTCCTGTAATCTTATGTTCATGGGCGTTTACACAATGGAGTTACT

GTTCATCATGGGGGTACCGTGGACAAGCCCAGGGCTGCCGGCGAGTCATG

CCATCCTTACACGTTTCTCCTTGTAAGACATGAACTTATGAATTCAACAG

GTGAAGATTTACAACTTGATAAATCAGCTTTGTGAGAGCCACCTAAGCTG

AAGATTCCCTTGAGAACAAGTACTGTCCTGCGGTTTCATGGCCCTTCTTC

CATTTGTGGTTCTTGCGAAGTGGAATTTAAATGACATCTTATCAAGATGG

ATAAACCCTAGTTTCCCAGTGCTGGAATATAGAAAATGGATGGACAAGTA

AATCCCACTCAGCACCCATAGTCCAGGCATGGGGACCTCAACACACCTGA

GCCCCAGACATCACCTTTCATTCTGGACCTACCATGAAAGACTTCTGAAT

CCAGGAAGAGAAACTGACTGGGCAACATGTTATTCAGGTACAAAAAGACT

TCGACTGTAACTCAAAAATGATCAAATAATAGTGCATGCATCAAGTGCAA

TGGGAAGCTCTTCTGGAGAGGGAGAGAAGCTTCCAGTTAAGGTGACATTG

AAGCCAAGTCCTGTAAGA

>hg19_ct_ARAlincRNAs_9727_ARAlincRNA_0055.10 range=chr1:243245192-243252681 5'pad=0 3'pad=0 strand=- repeatMasking=none

ACATGAACTTATGAATTCAACAGGTGAAGATTTACAACTTGATAAATCAG

CTTTGTGAGAGCCACCTAAGCTGAAGATTCCCTTGAGAACAAGTACTGTC

CTGCGGTTTCATGGCCCTTCTTCCATTTGTGGTTCTTGCGAAGTGGAATT

TAAATGACATCTTATCAAGATGGATAAACCCTAGTTTCCCAGTGCTGGAA

TATAGAAAATGGATGGACAAGTAAATCCCACTCAGCACCCATAGTCCAGG

CATGGGGACCTCAACACACCTGAGCCCCAGACATCACCTTTCATT

>hg19_ct_ARAlincRNAs_9727_ARAlincRNA_0055.9 range=chr1:243245192-243255900 5'pad=0 3'pad=0 strand=- repeatMasking=none

GTAGAGGTGGCCTGCACTCCTCAGCTTGTGCTGCCCGTCTCGAATGACTG

GAGTTTCCTGCTTCTGTCACTACACCTCCCACCCTCTCCCATCACCTGCT

CTGCTCTTACAAGGATCCGAAGCCACCTAAGCTGAAGATTCCCTTGAGAA

CAAGTACTGTCCTGCGGTTTCATGGCCCTTCTTCCATTTGTGGTTCTTGC

GAAGTGGAATTTAAATGACATCTTATCAAGATGGATAAACCCTAGTTTCC

CAGTGCTGGAATATAGAAAATGGATGGACAAGTAAATCCCACTCAGCACC

CATAGTCCAGGCATGGGGACCTCAACACACCTGAGCCCCAGACATCACCT

TTCATT

>hg19_ct_ARAlincRNAs_9727_ARAlincRNA_0055.2 range=chr1:243251430-243265020 5'pad=0 3'pad=0 strand=- repeatMasking=none

CTACGTCACTGGGGCGCTACGGTGCCTGGAGCTGGGCGGTCTTCTCGTCA

GAGTGGGGACTGGTGAGAGCGACCTCCCCGCCAGGTCCTGTGTGTTGCCG

GCTGAAGAAGGGTAGCTGAAAAATTCAGACCCAGCACAGTGTTTATGTTG

GTCAAAAATAGAAAACTATGGCGCGGCCGAGGCGGGAGGACCCTTCAGGC

CAAGAGCAGCCTAGCAACATGGCGCAACCCCATCTCTGTAGTCCTACCTC

AGCCCCCCAGCTACTTGAACCCAAAGATTCAAGGCTCCAGTGAGCTATGA

TCCCACCACAGCATTCCAGCCTGCGAGATTGAGGTAGAGGTGGCCTGCAC

TCCTCAGCTTGTGCTGCCCGTCTCGAATGACTGGAGTTTCCTGCTTCTGT

CACTACACCTCCCACCCTCTCCCATCACCTGCTCTGCTCTTACAAGGATC

CGAAGAAATGGAATAATCGTATCGCTGATCTATGTAAACAAATTGAAGAA

TTGTCTGAAAGAAAATATGACATGAACTTATGAATTCAACAGGTGAAGAT

TTACAACTTGATAAATCAGCTTTGTGAGCTCGAGCTGTAAAAGCCAAAGG

TCCGGTGATGATCCCATACCCTTTTTTCCAGTCTCATGTTGAAGATTTTT

ATGTAGAAGGCCTTCCCAAAGGAATTTTTTTTT

>hg19_ct_ARAlincRNAs_9727_ARAlincRNA_0055.8 range=chr1:243252623-243255496 5'pad=0 3'pad=0 strand=- repeatMasking=none

GTGGAGTCTTGCTCTGTCATCCAGGCTAGCAGTGGCGTGATCTTGGCTCG

CTGCAATGTCTGCCTCCTGGGTTCAGATGATTCTCCTGCCTCAGCCTCCT

GAGTAGCTGGGACTGCAGGCGCCCGCCACCATGCCCTGCTGTTTTGTATT

TTTGAGAAATGGAATAATCGTATCGCTGATCTATGTAAACAAATTGAAGA

ATTGTCTGAAAGAAAATATGGTATGTCTAAACTGGAAAAGTCCTGTAATC

TTATGTTCATGGGCGTTTACACAATGGAGTTACTGTTCATCATGGGGGTA

CCGTGGACAAGCCCAGGGCTGCCGGCGAGTCATGCCATCCTTACACGTTT

CTCCTTGTAAGACATGAACTTATGAATTCAACAGGTGAAGATTTACAACT

TGATAAATCAGCTTTGTGAG

>hg19_ct_ARAlincRNAs_9727_ARAlincRNA_0055.4 range=chr1:243252623-243265035 5'pad=0 3'pad=0 strand=- repeatMasking=none

GGCGCGGGTGTTCGCCTACGTCACTGGGGCGCTACGGTGCCTGGAGCTGG

GCGGTCTTCTCGTCAGAGTGGGGACTGGTGAGAGCGACCTCCCCGCCAGG

TCCTGTGTGTTGCCGGCTGAAGAAGGGTAGCTGAAAAATTCAGACCCAGC

ACAGTGTTTATGTTGGTCAAAAATAGAAAACTATGGCGCGGCCGAGGCGG

GAGGACCCTTCAGGCCAAGAGCAGCCTAGCAACATGGCGCAACCCCATCT

CTGTAGTCCTACCTCAGCCCCCCAGCTACTTGAACCCAAAGATTCAAGGC

TCCAGTGAGCTATGATCCCACCACAGCATTCCAGCCTGCGAGATTGAGAT

GATGATTATTCCCCACCTTCTAAGAGACAAAGACCAACGAGCCACCACAG

CCACCAGTCCCAGAACCCGCCAATGCTGGGGAACGGAAAATGAGGGAGTT

CAACTCTGGTAAGTTCTCAGCGAAATCCATGACCTTTTCCTTTATCTTCT

GGACTCTCAATGTGACTGATGAAAGTTACCACATGCTCTGCAGGGGGAAA

TGGTTTAGCATGTGTTACTACATCTTAATCACATCTTTGTAAAGCCAGGA

GCATTTTACAAGTCATGTTACAGACATTGTTTAAACATAGTCTGTATTTA

CCAAAGTATAGGACGTTGTATCATCTCATATTAATTAGTTAGTTGGCTCA

AAATGAGTGCTAATGACTTAGTAATTCAGTGATTTCTGTTAGCTTTAAAA

CCTTTATTTCAGAACTATTTCACCGGCCTGCACTCCTCAGCTTGTGCTGC

CCGTCTCGAATGACTGGAGTTTCCTGCTTCTGTCACTACACCTCCCACCC

TCTCCCATCACCTGCTCTGCTCTTACAAGGATCCGAACATGAACTTATGA

ATTCAACAGGTGAAGATTTACAACTTGATAAATCAGCTTTGTGAG

>hg19_ct_ARAlincRNAs_9727_ARAlincRNA_0055.3 range=chr1:243254693-243260889 5'pad=0 3'pad=0 strand=- repeatMasking=none

GAAAGTTATTTGAGGAGAAGAGGAGACTTACAATGATGATTCAAATGAAG

GAAACTAAAAAGTAATGAAGCAAGGCAGAGGAAAAAGCAGTATTCACTTG

AGCACATCCCAAAAGAATAACATTTCAAATGTAACTAGGAAAAAGTATGC

TGAAGTTCGCAATACAGAAATAATTATTAATAAGATAGCTTTAAAGCCCT

GCTCAGCTTTTGAATGTTGGGAATTGACCCAGAGGTGGCTGTAACCTAAG

ATGGTTCCTTCAGTAATGACCATTTTTTCTTTTTCAAGATGATGATTATT

CCCCACCTTCTAAGAGACAAAGACCAACGAGCCACCACAGCCACCAGTCC

CAGAACCCGCCAATGCTGGGGAACGGAAAATGAGGGAGTTCAACTCTGGC

CCTCACAATCCAGTGGAGGAGACGAAACTCATCTGCCTCTGTCCCTCTGG

GCACGCCTCATGCCAGGTGCATCTGTGGACAGGGGCCACGCTCCTGGGCT

TCCAAAGTTGGAGAAAGCTGCCAGGCTCAGCGAATGACTGGAGTTTCCTG

CTTCTGTCACTACACCTCCCACCCTCTCCCATCACCTGCTCTGCTCTTAC

AAGGATCCGAGTGGAGTCTTGCTCTGTCATCCAGGCTAGCAGTGGCGTGA

TCTTGGCTCGCTGCAATGTCTGCCTCCTGGGTTCAGATGATTCTCCTGCC

TCAGCCTCCTGAGTAGCTGGGACTGCAGGCGCCCGCCACCATGCCCTGCT

GTTTTGTATTTTTGAGAAATGGAATAATCGTATCGCTGATCTATGTAAAC

AAATTGAAGAATTGTCTGAAAGAAAATATGGTATGTCTAAACTGGAAAAG

TCCTGTAATCTTATGTTCATGGGCGTTTACACAATGGAGTTACTGTTCAT

CATGGGGGTACCGTGGACAAGCCCAGGGCTGCCGGCGAGTCATGCCATCC

TTACACGTTTCTCCTTGTAA

>hg19_ct_ARAlincRNAs_9727_ARAlincRNA_0055.1 range=chr1:243254833-243260601 5'pad=0 3'pad=0 strand=- repeatMasking=none

ATGATGATTATTCCCCACCTTCTAAGAGACAAAGACCAACGAGCCACCAC

AGCCACCAGTCCCAGAACCCGCCAATGCTGGGGAACGGAAAATGAGGGAG

TTCAACTCTGAGAAATGGAATAATCGTATCGCTGATCTATGTAAACAAAT

TGAAGAATTGTCTGAAAGAAAATATG

>hg19_ct_ARAlincRNAs_9727_ARAlincRNA_0056.1 range=chr1:249130803-249131409 5'pad=0 3'pad=0 strand=- repeatMasking=none

TTGAAGCATGAGCAGAGGGCCAGCACGCCACAGATGATCAATGAGTGTTT

GCTGACCCGCATATGACAACACGGATTAAGGAAGACAAATCTGGTAGCAG

CAGGCAAGAAAGCTGAAAAGCAAGACACAGGATGCAGGTCTGGAGTGATC

AGGGCCTGAACCAGGAAGGGATAACATTGAGAGAGACATGGAAAATGCGC

TTGAAGTATATATATTTTTATTGTAAAGAATCATACACAGATAAGGTAGT

ATTATATAGAGAATGATTAGTTGAGGATATTTCCAGAGTCTTTAAGCCAG

AGAAATGTACTAATTTAGACATTTCTGGACTCATTGCTCAGTACTGATGT

CATGTTTTTAAGGACACTGACACACTTGGCTAATCCCAGAGACATGAAGA

GGATGGTGACTAGGGTGGTCACATAATTAAAGGGGCTGGGGATATTTAAC

CTAGAAAGAGAAACATTTTTTGTGCACATAATGACTGTACACAAATTCTA

ACAAACTTTCATGTGAAAGAGGACTGGATTAGATGACTTTCTTCTGTGTA

GCTTTGGAGGGAAAAACTCAAACAATGGGGGGAAAGAACAGGAAGCTGGA

TTTAGGT

>hg19_ct_ARAlincRNAs_9727_ARAlincRNA_0057.1 range=chr1:249166967-249168821 5'pad=0 3'pad=0 strand=- repeatMasking=none

AGGGAGACTTTTTGAAATCTGTGACATAATTTTAAGACCAGCCATTTTTG

GCATACCCAGAGCTATCTTTTTGGATCATTTTGTGCTGCTGGCATTTTTT

CTCCGTTCTTAAGATATCATGAGTAAAGACACTGACTACATATACGGCGC

GCATAAACTGCAGTAGAGTCCATTTCAGGTAACTACTCTGCAAAATGAGG

ACACCAGGTGCGTTTTGCCCTTTGTTTAGAAGTCAAGTCATAGCTGACTC

TGGGAACATCACCTATGGTTAGCCCTGCGGCAACAGTTTTTAAAACGGCT

TACTTTCCTGACCGGGAATCGAACCCGGGCCGCGGCGGTGAGAGCGCCGA

ATCCTAACCACTAGACCACCAGGGAAGACATGATGCGTCATTTAGCTGCT

TCCCTCCCCTTGGTAGGTAGCCCCCTTGCTGCGGCAGACAGGGAAAACCG

GGCAGCAAAGATGAGTAGTCTAGTTGTAACCTTCTTCCTGGAGAAGACAA

TAAAATACCAACGGACTCCGGGAAAATTCTGGGGTTTCTTCCGGGGAATA

AAATGCTAATAAAACGAATGTGTCAGAAGTGGGATTCGAACCCACGCCTC

CATCCGGAGACCAGAAAGCCCGCAGGCAAGGTGCTTACCTTGAGTCTGGC

GCCTTAGACCACTCGGCCATCCTGACACTCGGAAGCAGAGCGCTTCTCAC

TTAGCCTCATACTTGTGTCCGCTGGTGCCAGCCTGCCGCGTCACACCCGA

GTTGCGGCCGTGGGAACCCTACTGACTGGTCGCGACGTTTCAGCGCTTCC

CGTTCCCGGCGGAGCTCTGGAGAAAGCCGCGACTGCAATGCAGCCAGGAG

TGGCCGATAGCGCTCTCCCAGGCCGGGAATCGAAGCTAACGACCAGGGGT

TAGTGACCGTGTGCCAGCGCCTGGCCCCGCAAACGCAGGACAATGCCTGA

GAAAGGCAAAGCTCGAGCTACCGCCTCACCTTCCGGCCCAAGCTGGGGAA

ACTCTGGCAAGCAGTTACTACTCGGAAAGTAGTTGGAAAAACGCTTGCCA

CTAAGTGACGAATGGATGCTTTTGTTTTTCTTCATTATCATCATTATTAT

AGCGGCTTTCTGCTTTATTGTGCCTGCGCGTACCCTCCCCTCAAGGTCCA

CCGCGCTTCCTCCACGAAACCACACAGATACGGTTGGCGATAACGGCCGG

ATCGGGAGGTCCTAGGTCAGAGGGGCCTGATGCGGGCCTCGCCTGAGGTG

AGGGAAGTCAGGCTGCGTTTACACAGCAGTTAACGAGTGCCTTAGCGTCC

AATGGGAAGGCCCGGGACTCGCAGTGCAGAGCAGCTCTGGCAGTGTCCTC

CCGGTGGGCTGTGAGGTTACCTGTGAGCCCAGATACCACTCGTCCAGGCA

TTCATTCGTTGAATCTTAACATCGAGTGTCCACTGGGAACACAACGGTAA

ATAGACAAAGCCCCAACTTGGTGAGCTGATTTGCTAGTGAAGGAGATGAC

CAGCACAATCACATGTACAGAGTGTGAGGAAAAACCAAAAGCAATGAAGT

GGTGGAGAAGTTCTATTTTTAGACTGTGCAGTTAAGTAAGGCCTCACTAA

AGTGACTTTTGCTAAAATATTTTTCAAAAACTAAAATATATGTCATGCAG

CCGGGCGCGGTGGCTCATGCCTGTAATCCCGCCACCACGTTGGGAGGTGG

GCGGATCACCTGAGGTCGGGAGTTCGAGACCAGCCTGACCAACATGGAGA

AACCCCGTCTCTA

>hg19_ct_ARAlincRNAs_9727_ARAlincRNA_0291.1 range=chr2:1753646-1757701 5'pad=0 3'pad=0 strand=+ repeatMasking=none

CATTCCATCGTGAGGAGGGGCCCTGTGACTGCAGATACTCAATGCCTTCC

AAGTGCGCTGGTGCCACCTTCAAGTGACACTTCTCAGAGTGTCACCCAGT

GGCCCAGAGGCTCCCTAGTGTGTAGTCCACCCTCGCTCAGACACGGCGGC

CTGTACCTGACATCCACACTCCCCACCTGCTGCCACCCACAGGAGCATTT

CAGGGCATCACGAATTGGTGGCTTTTCTTTCCAGTTCAACAGGCATCTCC

CAAGTCACCTGTGGTGATTGCTTTTATATGTCCACGTGGCCACACTCATC

TAGACATTGCTCTGAAGTACTTAGACGCAGTGAGTGAAGAGACACTTGGT

CCCGGGCCCCCTTCTCTGCACACACTGCCCTGTGTGCTACAGCATGCCAG

GCAGGGTGCCAGGACAGCAGGCCCGTGAAGACCATGGCGGCGGCTGTCTG

AAGACCAGCTCACAGCAGGAGCTGCGCTGAGATCTTCTCCTGGGTGCTCA

TGCTGGGGCCCGAGGTCGTATGGAAAAGGCTTGTCCCCCTTGCTGGATGT

CACAAGGCTCAGCCACGTGGATAGAAGCTGCTTGGATGACCACATTAAGT

GAATGGCCGCATCTGTGCACTGGCTCTGCAGCCCTGGGCAGCGACACAGA

GACAGCAAGCAGAGCATCCATCACCAGGATTCTCAGGCAGGTGTTACACC

AGAGCTCATGCGTGTCTGGCCCCAGGCTCTGAGCTGTGGCGCATTCTTCT

TGCACATGCCCTGTAGGCCGGGTGAAATGTTGTCACTGAAGGTGAGCTAG

GAAGTCACAGGGCAGGTTTCACAAGAGACACATCACAAAGACCCTGCTCA

TAAAACAGGATGTGGGAGAGAAGCCAGCCAAAACCAAGATGGTGGCAAAA

GAAACCTCTGTTCATTATACACTAATCATAATGCATTTGCATATGCTCAA

TGACGTGCCTGGCAGCACCAAGACAGTTTACAGACGCCATGGCAACGTCT

GGAAGTTACCCTATGGGGTCTGAAGTGGGGAGGAATTCTCAGTTCTTGGG

GAATTCCCTGCCTTTTTCCCAGAAAACTCATGAATAATCCACCTGTTGTT

CGGCATATAATCAAGAAATAACCATAAAAATAGCCACCCTTTTATTCCTG

TGCGGTAACCACCCATTTATTTCTTTACTTTCTTGATTAACCTGATTTCA

CTCTGTCCTCTTGCTCTTTTTTTTTTTTTGAGACAGAGTCTCGCCCTGTC

ACCAGGCTGGAGTGCAGTGGCATGAACTCAGCTCACTGCAACCTCCACCT

CCCGGGTTCCAGCGATTCTCCTGCCTCAGCCTCCCGAGTAGCTGGGATTA

CAGATGTGCACCACCACGTCCAGCTAATCTTTGTATTTTTAGTAGAGATG

GGATTTCACCATGTTGGCCAGGCTGGTCTCAAAATCCTGACCTCAAGCAA

TCCACCTGCCTCGGCCTCCCAAAGTGCTGGGATTACAGGCGTGAGCCACT

GTGCCCAGCCTGTCCTCTCACTCTTGAATTCCTTCCTGCATGACACTAAG

AACCCATGTGACCTCCCGGGATGAGCCCCGATTTGGGGGTTCACCTGTGA

TAAAGTAAGCTGGTGAACAGCAGGGAAGCACTCTGTGTCTGTAGCTTCCT

GTGAGTATGCAATCATTTCAAAATTAAAAGTTTGTTAAAGCTATAAAAAT

TATAAAATAAATTAATAAAA

>hg19_ct_ARAlincRNAs_9727_ARAlincRNA_0291.2 range=chr2:1754993-1757189 5'pad=0 3'pad=0 strand=+ repeatMasking=none

CACACATAGTACTTAGACGCAGTGAGTGAAGAGACACTTGGTCCCGGGCC

CCCTTCTCTGCACACACTGCCCTGTGTGCTACAGCATGCCAGGCAGGGTG

CCAGGACAGCAGGCCCGTGAAGACCATGGCGGCGGCTGTCTGAAGACCAG

CTCACAGCAGGAGCTGCGCTGAGATCTTCTCCTGGGTGCTCATGCTGGGG

CCCGAGGTCGTATGGAAAAGGCTTGTCCCCCTTGCTGGATGTCACAAGGC

TCAGCCACGTGGATAGAAGCTGCTTGGATGACCACATTAAGTGAATGGCC

GCATCTGTGCACTGGCTCTGCAGCCCTGGGCAGCGACACAGAGACAGCAA

GCAGAGCATCCATCACCAGGATTCTCAGGCAGGTGTTACACCAGAGCTCA

TGCGTGTCTGGCCCCAGGCTCTGAGCTGTGGCGCATTCTTCTTGCACATG

CCCTGTAGGCCGGGTGAAATGTTGTCACTGAAGGTGAGCTAGGAAGTCAC

AGGGCAGGTTTCACAAGAGACACATCACAAAGACCCTGCTCATAAAACAG

GATGTGGGAGAGAAGCCAGCCAAAACCAAGATGGTGGCAAAAGAAACCTC

TGTTCATTATACACTAATCATAATGCATTTGCATATGCTCAATGACGTGC

CTGGCAGCACCAAGACAGTTTACAGACGCCATGGCAACGTCTGGAAGTTA

CCCTATGGGGTCTGAAGTGGGGAGGAATTCTCAGTTCTTGGGGAATTCCC

TGCCTTTTTCCCAGAAAACTCATGAATAATCCACCTGTTGTTCGGCATAT

AATCAAGAAATAACCATAAAAATAGCCACCCTTTTATTCCTGTGCGGTAA

CCACCCATTTATTTCTTTACTTTCTTGATTAACCTGATTTCACTCTGTCC

>hg19_ct_ARAlincRNAs_9727_ARAlincRNA_0292.1 range=chr2:8806773-8810423 5'pad=0 3'pad=0 strand=+ repeatMasking=none

TTTCTGGTCAAATTTATTACATGATAAATTAGTATATTGTTATACAATCA

TTTCAATATAGAGTGTATAACAGCATGCACATCTCTGTCCCCCAGCCCAA

GAAACAGCACATTCAGCATGCACCACTGAAGCTCCTGGAGTAAACCTCCA

GCCCTGGTCCTCGGCCCCCGCCTGGAGAAGTAACCATGTGGATTCAGCAC

TCCTGGCCCTCCGCACTGCTCCATACTTTCATACTCACGTTGGAAACAAT

GAGTAGTGTTGTGTTGTTTGTTTGAACACTCTGGATACACCAGCATCATA

TTGGACATATACTTCTGTGGCCAGGTGGTCTTCTTCCATCCTTATGTCTT

TTATCTTCATCCATTTTGACGTGTGCAGCTCTAGTCCATTTATTTTTACT

GCTCTCTAGAACTCCACTGTTGGGCACTCCCACATTATCCCCTCTCCCAT

AGATGGAAACTTAGATGTTTTCATAATGACAAACAAATGCCGCAGTCAAC

ACGTGTGCACATTCATGCGCATGCCCATGGCTGGAACTGTGGGGTCAAGG

ATGGGCACATCTTCAGTTCTACTCAGAATGCCCCTTTGCATTCCAGTGTG

GTGGGATTGATGCCCATGCTCCCTGGCAGTATTGGAGAATTCCCACTGCT

CCACCCCCACACCACCCCACTGTACACCTTCCTACACTTAGTTCCTATTC

CCTTCCTGGACTCACCGCGTTTCTGCCACCCTGGCCTCGATGTTCCATGT

ATGTACCAGGCACTCATGCCTTGGAACTTTTACACTGGCTGTTCCCTCTT

CCACAAATGCTGTTACCCAGGTACAGTCATGTTCTCTCCTTCCTTTCTTC

AAGTCTCTGTGCAAATGTCACCTTCTCAGTGCAACCTATTGGGGCCACCT

TATTCACAACTGTACCCAATCCTTGCTCCTGGCACTTCTCCTTTATCACC

TACACCTTTATCACCTTCTAATAACCTATGTGATTCACTCACCAATTTTA

TTTCTTTGTCTCTTCCCACCAAAATGTAATATCCAGGTGGTGAGGGATTG

TTGTTGATTTTGTTTCCTGGTATTTCCTAAGTTCTGAGGAGAGTGCTTGG

CACATAATAGGCATTCAGTAAATACTTGCTGAACACATGGATGGATGAAT

GGAAGAATGGATGGAAGGATGGAAGGATGGATGGATGAAGTGATCAATGG

ATGATGGGATGAATGGATGGATGGATGAAGGGACGAAGGGATGGATGGAT

GGTTGGATGGAGCAATGGATGGATGGATAGATCAATGGATGGATGGATGA

AAAGATGAATGGATGAATGGATGGAAAAATGGATGAAGGGATGAAGAGTA

AATGGATGGATGGATGGATGGAGGAATGGACATATGGATAAAGGGATGAA

TGGGTGAATGGATGGATAAAGGGATGGATGGAAGTAAATGGACGGATGGA

TAAATGAATGGATGGATGAAGGGGTGAATGAATGGTAGACGATGGATGAA

GGGATGAGTTGATGAAGGAATGGATGGATGAATGGGTGGATGGATGGATG

GACGGATGGATGAAGGGATGAATGGAAGGAAGGAAGGATGGATGGATGGA

TGGATGAAGGGATGAATGAGTGGATGGATGGATGGATGGACGGATGGATG

AAGGGATGAATGGAAGGAAGGATGGATGGATGGATGAAGGGATGGATGGA

TTGATGAAGGGACGGATGGCTGGATGGAGTGATGGATGAAGGGATGGATG

GATGGGTGGATGGATGAGGGGATGGATGGATGAGGGGATGGATAGATGGA

TGGATGGATGTCTTAAAGCTCAGTCATTTGTGCTACTATTCACACTGGCT

GCAATTTGTGAGCTTGGAAAACCAGTCCTTTTTTCCAGCCTGAGGTAAGA

TCTGATTCTTCACAAGGCTGTTTCACTACTGACATGTTTTCATCAGATAC

GTTTGCATGTTCATTAACTCATGTAAACATTATTAAAAGGACTATGTAAA

CAGCTTCCTACACGTTTCCTCTTGCTTTCATTCAGGTATAATTATATGAA

AACACAATGTACCATATGTGCCAACGGGCCATTCCCTTTCTTGTGCTTCC

AAACGACAATTCCCCTCTGCAAACACACTCAGATACCAGGTCTTTACATT

TCTCCTTCTACTCCAAATCTAATCTGCCTTCTGAAGACAAAAATCTCAAA

GCCCCTGCCCAATGATATGGCAGCCAACAATGTCCATTCTCACCCTAATA

TGGTCTAGGAACTCACTCTTACCTTTAAAAGTGCTTTGCAAATTGTAAAA

GGGTATTCAAAAATGGTTACTTCCTCCTTCACTGTACCCTTGTCACAACT

ATTCAAATAGTCATAAACATTCAATGCTGCCAAACATGATGGTGGTTTCC

AATCCTCTTCACCCTACCCAAGTCCAACATGTCACCCCTCCAATAGTCAC

TATGTCCCCTCACACAGGAAACATCTCAGAAACAGGCATGAGTGTCCTGA

CAGCTGCAGAGGAATAAGTATGCTGAAGGTCACTGAAAGTGACTGGCCAG

CAGCCTCTAAATCAGTTCTATTTTCTTCCTGGCATACTACTCGTCTAGAT

CTTCCAGCCCCCTCCCTTGCAGTTAGGTATGGCCATGTGACCGAGTTCTA

ACACAGTGGTTCTCAACCAGGACAATTTTGCCTCCCGGGGACATCTAGCA

ATGTCTGGAGAGGAGGCAACTGTGGTTGTCACAACTAGAGGAGAGAGGAG

AGTTCTATGGGAATCTAGTGAGTCTCTGAGGCCAGAGAGCATCCTGCTGA

ACATTCCACAATGCACAAGACAGCTCCTCACAACAATAAACACCATTCAC

AGGCTTTGTGGCTTTGTTCTAACTCTGATTAGCCTTTGGCCAGCTCAAAT

GCCCATGGTGCCAAAACCCTACACTCACCCTGTACTAATAGTTGAGAAAC

CCAGTTCTAACCAATGGAATGTGAGCTAATGTGTTGGAGCCAATTCCTTG

CCTGATCCATAAAAACCAGCCAAGTGCAGTTGTCCAAGCTCTTTCCTCTT

CCTTAGTCTGGGTGGAGATGATCCCAGGCAACTTGGAAGCCACACGCCGA

ATACAATGGGGCCACAAAAATGGGAAGAGCTTACATCTGTCACTGCTTGA

ATGAGAGCTGTCTGCCCATCAGTAACACACATTTTACACTACGAAATAAA

CTAGCATGTTAGAGATCTGGGTTTGTTCAAGATACCAGCTGGCATTAGCT

AACTCAGTCACAAATACTTTTGCTTGTTTTCTTGAATAGATTCTCCAATA

TATGGAGCTCCATTTGAGCCAACATCACCTGGGGGACATCTGGATTGGCC

AGTCCTATTTCAAGTGGGAGAGGGATCTCCAGGGTACATTTCTCTGGAAA

TCAACTTATAACTGATATAACGAGCTGGCTTATGCAAAGACACAGGAGGG

GTCTGTGGGCCCATTTAAGTCTCTGAGGAACTTTTGAAATTGGTCCTCTT

TAGATAAGC

>hg19_ct_ARAlincRNAs_9727_ARAlincRNA_0293.1 range=chr2:32580962-32581793 5'pad=0 3'pad=0 strand=- repeatMasking=none

CAGCTGTTCGGCCGTGGCCGGAAGCGGACCTCTGGGTCTCGGTCACGGCT

CTGGTGTGAGAATAAACAATCAGAAGCCTCTCGTTTGGATTGGAAGGAAC

CGAGCGACCTTCCCAAACCTGTACCTTTTACCACGCCTGCCCTCCCCATT

CCCAAGGCTTTCTGTTGAACAGAAGTACCAGATACGAGGTGCTGGAAGCG

CATAGTTAAGCTTAGTTTCTCTATCCACATTCCAAACAACCCGAGTTACC

GGAATTAATAGGAGTGTGGGTCTGTGTGAGTGTGTGGTGGGGTGTGTTGG

AGGTCTAGAAAGGACTAATCAATTTACTAATCATCCCCCCTTGGAGAATT

TTTTTAAAACTTTTTATTTTGAAATAGATTACAGTTGAAAATATACTACA

GACCTCTGTGGTCTTTCTCTCCAAAAGCCATAACCCCTATCTAACTATGA

GAAAACCATTTGACAAACCCAAACTGTAGGACATTATTTTTATAAAATAC

CTAAACACTACTCAAAGATAGTACAGAGAGATCCAATTTACTCTTTACCC

AGTTTCCCCCAATGGTTGCATATTACATAATTCTAGTACAATCTCAAAAG

GAGGGATTTGATAGTTGTATGATGTGTGTATAGTTCAATGTCATTTTATC

ACATGTAGACCTACGCACCATAGTCAAGATGCAGAGTTGTATGTTCTATA

TGTTTTGTGATCATTAGGTTATTTTGGAAAGAACTAGTTTTCCTTAAAAA

CCTAATCAAAGATCTGTTGTGAGGATTAAGAATCATTTGTAAAATGTGTA

GAAGAGCATCTGACAAGTATATACTACGTGCT

>hg19_ct_ARAlincRNAs_9727_ARAlincRNA_0294.1 range=chr2:62421666-62422204 5'pad=0 3'pad=0 strand=- repeatMasking=none

CGTGCGCTCCGCGGGGAGGCAAGCCTAACCAGGGGCTCACCTGGAAGCGT

CCCCGCCCCCTGGTCCTTATCTCTGAATCGAAACCGAACCTGGACCCATC

TAGATAATTGGACTGAGCAATTAAGGACGCTTCGCTCCTAACTGTTAGCA

TTTTTTCCTAAGGCTCTCGGAGGCGGAATCAGCTGGAAAGGAACATTGCT

AAGTAGGAATTTCAGGCACCTGTGAACTGAGCCCACTCAATAATCGGCCC

TTTCCCTTAACCAAACTCAGCAAAGGAAGAAAGACCAAGTTGCTGCGGAT

GCACAGAGCCACGTGGGTGCGGGTCCTCTAGAAACAGGAATTCAGAGTCA

GTAGATATGGAAATGTTATGGTTTGCAGTCTTCGGACCATGCTGCTTTCA

TGCTGTTCACATGATTCTCCTCGGACTGCGAGGTAAAGTCCAAGAAACAA

GTCGAATGTTTGTATTTTCATAGAAAAAACATCTGTAAGGGTGCACACCG

ACTGTTAACCACGGTTACGCGGGGAGGGGAAACAAGGGA

>hg19_ct_ARAlincRNAs_9727_ARAlincRNA_0295.1 range=chr2:71294649-71295255 5'pad=0 3'pad=0 strand=- repeatMasking=none

CGAACACTCGCTCAGATTGTTGCCCTGCGCTCGTGTGAATGTACGGATCC

GGGAGGAATTGGGACCTAAACAGACAAGCCCTCGAGGCGGTCCCGGCAGC

CCTGAGGAGGGAGCCTTCACCTCTTCCTACCCACCCAGTTTACTTCTTGT

GGAGGATATTTGCAGGGACATAGCCCACTGGGAGAACTAGCAGATACGGC

TTTACAAGTTTTTTATTGCCTTTCAGCCCCCATTCCGCTCCCCTTCCTTG

CGCTGCGTCGGGAAAGGGAATGGGATCCCTAGGGGTGCAAATACAGGCCA

AGTCAGGGAGAGCTGTGGCTCCTGAGGGCCTCCTCTGGCTCCTCCGGGAA

GTTTTGGGCCCTCAAGGAGCAGCTTCGCGGGCCAGGAGGGCGGTTAGCTG

CCCTTGGCATCTTTTTGGGGAAATCAGTTGGCCTGTCGCGGGTCGAACTC

TGTCCCCCTTGACTGCCTGCCATCTTCTGGAGAAACCCCTAATAGGCTGC

TGTGTCTGGTTATGCTTTAGTGAGAAAATTAGGCTTTTTAAGCTTTCTCA

TTTCCTACCCTACCACGTTGTAATTCACAAAAGTAAAAAGTAAATCACTA

CATGATA

>hg19_ct_ARAlincRNAs_9727_ARAlincRNA_0296.1 range=chr2:73954511-73955520 5'pad=0 3'pad=0 strand=- repeatMasking=none

GAAGGAGGTACAAAATGGGATGGGTAAAGTATAACTGCCACAGGCATTCA

CCCAGGAATGTCAGCAATGAAAGAGAACGAATTCCTCTCTCCAGGCAGTA

TGGAGTCCCAGGAAGACTCTAAGTTCTGCTACTGATGGATGAACTGGGTG

CCTGTAGAGTGTGGAAGGCCTAGGCATGGGTTCTAAACTCCTTCTCAGCT

GCCTGACCCCCAAAGTCCGGCCTCTGGCGGTGGGGAAATAGAATTACAAA

CAAAATCTATCCCAGCAAGCCTCTCTGCAGAGGTAGAAGAGGAAGAAAAC

ACTTTCATCATTGAAGAGGCATTAACTCAGAGTGAGATGTGCATCACAGA

CAATGATCCGCTAAGATGCTGCAAAGACAGAAATCTCACGCTTATCTAGC

CAGTGGGTCCAACCCATTGTTCACTGCACGCATTCTCAAGAAGAGTAATG

CCTAGTCCTCAGATCAGTGGACTTCCCAGCACCTTTGGCCACACCCATTT

ATTGGAGATTCAACCTCAATTTACCTGACCACCTGCGTTTGCTAATTACC

TTGTTACAAAGGAAAATAAACTCTTACCTTCGTGACAGGAGGTAGATTTA

CTACTCACAGCAGATGTTAGGCCCCCACCATCCCACAGAGACTGGCAGAT

AGGGCCCTGGCTGCTCTGATGATGACGTTTCAAAGACGGGCTCCCAGGTC

CTTCAGGAACACAGTCCTGGGTTGCAAAGCTGGCAAGAGGCTTATTTAGT

TGTCAAAAAGCATTGCATGCCCCTCAAAGGGGCAGAGGAAGATTTACAAT

GACAAGTTTTCCAAAGAAAACGCTCAGAGAGGACACCACTTCCCTTGTTT

TGTTTTGTTTTGTTTTTTATTATTATACTTTAAGTTTCAGGGTACATGTG

CACAATGTGCAGGTTAGTTACATGTGTATACATGTGCCATGCTGGGGTGC

TGCACCCATTAACTCGTCATTTAGCATTAGGTATATCTCCTAATGCTATT

AGTCCCCCCT

>hg19_ct_ARAlincRNAs_9727_ARAlincRNA_0297.1 range=chr2:74345626-74345994 5'pad=0 3'pad=0 strand=- repeatMasking=none

ATCAGTTTCCTGGGTGCAATAATTTTATGTAGTTATGAAGGAGAATGACT

GTTCTTAGGACATACAGACAAAAGTATTTACAGGAGAAGTGTCTTGATGT

CTGCAACAAATTCCCAAATGGTTCATATATATGTGTTATATATATGTTTA

TATATATGTAAATGTAAATATATGTGTGTGTGTATGTGTATTCAGAGAGA

AAAGCAAATACAATGTGACTTTTGAACAATGTCAGTTAAGCATTCTGACT

AACCCACATATACATTTTGACTCCCCAAAAACTTTACTAATAGGCTACTG

TTGACCAGAAGCCTTACTGATAATATAGTTATTTTGTATGTTATATGTAT

TATATACTGTATTCTTACA

>hg19_ct_ARAlincRNAs_9727_ARAlincRNA_0298.1 range=chr2:74346187-74347301 5'pad=0 3'pad=0 strand=- repeatMasking=none

GGGAGGAGCCCGAAGCAGCGTCCTTCCGGGAGCACCCAGGGAAGAACCTG

GAGGTCCAACCTGTGGGCTCTTAAGAGAAGGGAGGCGGCCTATCGAAAGT

CCTAGAGGCCCGAGGTAACAGGGCTTCAGGGGCCACTAGTACGTCTTTCC

TGCTGACACCTCAGGCCTGTAACAGAAAACAGAAGCACCCAAGGAATGTC

AAAAGGCCACAGGAATAAGTTTGAAGGGGCTGCCGTTGGTTGAATTTTGT

ATAATTTGACAAGTATTTAAAACATTCAATTAAAAAGAATCCATGGGTTC

ATATTACCACAACTACTACACTAATATTAATATAAAAGGAGAGAGAAGTA

AAGATGCTTTTTCAAAACAGTAGAATGACAACCAATAAATATAGGAATTA

TAAAATTGGAAATCCACCATTTTGCAGCAATCGTGGTAATAATTGATTCA

GCCAAGAATCACCAGTGGAAGCTGAAACTATTAGGTGAAAGACTGGGAAT

AGGTTATTCACACAGTCTTAAAGTAACACCTCCTGGATTACTTATTAATT

ATAAAGGGGAAATTTTTCAGGCAATGGAGACATTTGCTGGACATTGCCTT

AGCCAAAAGTCAAACTTAGCATTTCCAGTAATAGGACAAACTGAGACTGT

GTGCCTCCTGGTGTGAGGAACAGGAAGAACACACATCATCTGGGTAGTAT

TTCTAATAGGCTCAACCTGATTAATGAAGAAACAATCAGACAAATCAAAA

TTGAGGAACATTCTGCAAAGCAATTGACTTGAGTTCTTAAAAGATGGCAA

TATCTTTTTTTTTTTTTTTTTTGAGATAGAGTCTCACTCTGTCTCCCAGG

CTGAAGTGCAGTGGTGCGATCTCGGCTCACTGCAACCTCCGCCTCCCCGG

TTCAAGCAATTCTCCTGCCTCAGCCTCCGAAGTATCTGGGATTACAGGCA

CGTGACACCATACCCAGCTAATTTTTGGATTTTTAGTAGAGCCAGGGTTT

CACTATGTTGGCCAGGGTGGTCTCAAACTCCTGACCTCAAGTGATCCCGC

CTCAGCCTCCCAAAGTGCTGGGATTACAGGCGTGAGCCACCGCACCCAGC

CAAAAGTTGGCAATA

>hg19_ct_ARAlincRNAs_9727_ARAlincRNA_0299.7 range=chr2:75145004-75159917 5'pad=0 3'pad=0 strand=+ repeatMasking=none

GTCTGAAGTTCAGAGGAGAGTTCAGGCTGGAAAGAATATATTCGGAGTTG

TAAACGTGAGGCCAGGCAGGATCACGAGGAAGTGAGTGTCAGTAGAGAAG

AATGTGCATAGCCTTAGGCACTTGAGTGAATAGGCTCAACTGTGTGGAAG

TCTGCTGACAGGTCATCTAAGATGAGGACTCTCAACTGACCGTTGGATTT

GCAATGTGGACCTGAAGAGTAATTGTGGTGAAGTTGTGAGAATGAAACTG

AAGTGGGTTCAAGAAAGAACGAGAGGAGAGAAAGTGGAGTTTGAGTATAA

ATAACTCTTTTGGAGAGGATTGGTGTAATTGAATGGCAGGGGTATGAGAT

TTGAGGTCAAGGAAATATTTTTATTATTTTTTACGATGAGAGAAATTGTA

GTACACATGTATATTTATGGGAATGACTCAGTAGAAAGACCAAAAATTTC

ATATGTGAGAGAAGGACCAATTGATGAAGCGATGTTCTTGCGTGGGCGCG

AGGATGGGATCGAGTGCACAGCGGAAGGTGGCACTTAGGAACACGCAGCA

CTCGTCCATGCCAGCAGGAGGCAAGGCAGAATGGAAATAAAACTTTAACC

AAAAGAGCATGGGCAGATATTTCCCGGATCTGAACAGGAATAGCTGGTGT

CTGGACTCTATGAAGAATCTTCATGTGCAGTGATAGAAGAATAAACACAT

TTGCAGACTTTTGTAGTGCACAGTGACCGGTGACCAGCAGCATGTGTTTC

CATAGGCATCTTCTCTCAGTACCCCAGAAGGTGAATTGCCAGTGAGTCCT

GCCAGGATGCCCCTCGGTGACTGCACCACCCA

>hg19_ct_ARAlincRNAs_9727_ARAlincRNA_0299.8 range=chr2:75145004-75170624 5'pad=0 3'pad=0 strand=+ repeatMasking=none

GTCTGAAGTTCAGAGGAGAGTTCAGGCTGGAAAGAATATATTCGGAGTTG

TAAACGTGAGGCCAGGCAGGATCACGAGGAAGTGAGTGTCAGTAGAGAAG

AATGTGCATAGCCTTAGGCACTTGAGTGAATAGGCTCAACTGTGTGGAAG

TCTGCTGACAGGTCATCTAAGATGAGGACTCTCAACTGACCGTTGGATTT

GCAATGTGGACCTGAAGAGTAATTGTGGTGAAGTTGTGAGAATGAAACTG

AAGTGGGTTCAAGAAAGAACGAGAGGAGAGAAAGTGGAGTTTGAGTATAA

ATAACTCTTTTGGAGAGGATTGGTGTAATTGAATGGCAGGGGTATGAGAT

TTGAGGTCAAGGAAATATTTTTATTATTTTTTACGATGAGAGAAATTGTA

GTACACATGTATATTTATGGGAATGACTCAGTAGAAAGACCAAAAATTTC

ATATGTGAGAGAAGGACCAATTGATGAAGCGATGTTCTTGCGTGGGCGCG

AGGATGGGATCGAGTGCACAGCGGAAGGTGGCACTTAGGAACACGCAGCA

CTCGTCCATGCCAGCAGGAGGCAAGGCAGAATGGCAGACTTTTGTAGTGC

ACAGTGACCGGTGACCAGCAGCATGTGTTTCCATAGGCATCTTCTCTCAG

TACCCCAGAAGGTGAATTGCCAGTGAGTCCTGCCAGGATGCCCCTCGGTG

ACTGCACCACCCAGTAAAAAGACATCCCTTAACTATGGCAAAACAACAAC

AACAACGACGACAAAAAACAAAACAAAACAAAACAAAACCCTGCATCAAT

TTGATAAATAATCCTCCCCGTTGATGTCACTGGTTTTTTTCTGCACATCT

TCTCACTGATATTTAAATGCTGAGCTTACACTTTCCATGGACATGCTTAA

TGACTAAACAATAGTTGAATTCTAAATTAATCAACTGACCAACCAGTAAG

AATTTATTGAGCAACTCCTTTGAAGCCGTCTATCTGTTAGGTGCTGTGGG

AGCACAAAAGAAATAGGACATCATCTCTGCCTCAGGGAATTTAGTGTTTT

GGGGAAAACCTGAGTGGTATACACAAAGCAATTGAGGAACAACCTAGGAC

CTTGTAGTCCATTAGGATTTCCAAAAATAGAAAGAGAATGGAAAGTCACC

TGGCTAGGAGAAGCCAGTGGAAACTTGACACGAAGAAAAAAGAGCAGCTA

ATTTCATTCCTGTCCACCAGTTATTTATGTGTTTATCTTTAATTACATTT

GTTTGATTTCCCTTATTAAAGTCTGATGTCTTAAAAAAGCAGAAAAGTGA

GGCAGGTCAGCAGGGGATGTAAGTTGGGAAGAAAGACAGGTGAGGGCAAG

AATTTAGGCAGGAGCCACAGTGTTGGTTGTGCAGGTGAAGGTCAGGTGAC

GGAGGGTAACCAGTCATGGATGACCCAGGCAGGAGCCATAACCAAAATGT

TAGAAAAAGTTGGTAAGAAATTGTTCCAGGCCAGGCGCAGTGGCTCATGC

TTGTAATTCCAGCACTTTGGGAGGCCAAGGTAGGCGGATCACTTGAGGTC

AGGAGTTTGAAACCAGCCTGGCCAACATGGAGATACCCTGTCTCTACTAA

AAATACAAAAACTATCTGGGCATGGTGGCACGTGCCTGTAATCCCAGCTA

CTCAGGAGGCTGAGGCAGGAGAATCACTTCAACCCAGGAGACAGAGATTG

CAGTGAGCCGAGATTGCGCCACTGCACTCCAGCCTGGGTGACAGAGTGAG

ACACCATCTCAAAAAAAAAAAAAAAAAAAAGTTTTCCAGGAACAAGGGCA

GAACTTAGCTACTGAGTTCTACCGAAGGCCAGGATCTGAGTCAAGCACCA

AGGTTAACTCCCAGGGAAGCCAAATGTTCATAAAAGGAAATCTTTCCAAA

TCACAAATATCAGGGCCTGCTAGGTAAATTCTAGCTTCTCTGTGCCAAGG

GCTGTGTTCCTACTCTCAAGCGCCACTCACTGACTGTCTGGGTGGTGCTG

TGGGCTTCTGTACTTTTAGCTTCATCAGCTGCACCTGTCCTCTTTTCCCA

TAAGACGCCAGGACCCTCAGAATCTTCCCTCTTACCAGAAATTTGGGTTT

TGGGCTGGGTCCCTGGACCGAATTCTGACACTCTAAGTGTTTGCAGACTA

TTTTGATAGAGGTAGTGATTATGATGCCAGAAAAGTGAAGCCAGTTTTTA

TTAGAATGTTGGCCATCTTGAATAATCCATTCCCCCACTTCCATGGGACA

TCTTCAAAGAGATTTTTAAAAGACAAATATGATCTCTACTAATGAGATAT

CTTCAGAAGGTAGAATTAAAGCAAAATTAATGTACACAGAATTCCTATTT

CAATATATTTATTATTCACAACATTTCTACACACACACACACACACACAC

ACACGCATACACACACACACACACACATTCCTGCACAGGTAATTTATAGT

GGCTAATCCACTCAGTACTGTGGACTTTGGTTTTAGAGAGCATTGTTTTC

AAGGGTAGTACAGATAATCTGCCTCCAACATATTCTATTCTTAGGGCTCT

AAAGTCAAAGGAGTTTGTACAGACCCCTCCCTGCTAAGGTAATTATGTCC

TCTTCATATCTTGCATGAATGTTTGCTCTTAGAGTGATGATCTGAAATAT

CAACATCCTGCAGGACCTAGTCTGGTCTGGAAGAAGAAGAGATATGTCAT

CTCTATGGGCTTTGATCTTGGTAGGCATATTTATTCACCAGGTACACAAA

TGCAACTTTAAACCCTCAGCCACATTTAGTAAACTATTTCTTCATCTGTT

GCAGTCTTATTCTGATGGGAAAAATTTCCTCTGTTTTTCAAGTTTAATTT

ATTATTTTTTCAAATTACAAGAGCAATGCATATTATTGTAACCAGAGAGA

TAATGCCAAGATCGGTAGAGAAAAACTTAATAATCTCGACCTCCTATAAC

CATCTCTCTTCCCATCCCCCTGAGGTAATAGCAAATGTTAACAGCCTGCT

ACCTCTGCACATCTTCCTTCCTTCATGATTTCCCTACACACACACCCATA

CACACACGTGCGCATGCACACACACACCCACTCAAACACACGGTGTACGT

CCAGGAGTCAGAAGTGGCTACAGAGATGGAGTCTTCATTATTAAGGTGAG

AAAATGATAAGACAAATCACCCTTCTATTTCTACCTCCATGGAAAGCTAA

ATCTTCCATTTATATTTTTATAGTCTTTTCAGCACTGTCTTAATGGTCTT

GAGAACATATTACCCTGAAAATGTAAAAATAGAGCCAAAGAGGTGAAAGC

TCCTTGCGTGGCTTCTGACCAGGGATGGCTGCTTCCATGGGCTTTAGCTC

TGCCAGTTCTCCCTAGACTGGGGCAGGTAGGCAGTGAGGATTTCAGCATA

TATTAAGTGCCAAATAGAAACAAATATTTGTTTCTATTATTAGAGTAAAA

CAACAGAAGCCTGTATTTTGACCAGAGGCCTGGAATCTACTACTTTGTGA

ATTAGCTAAGCAGCCCCAGTTGAACGTGAACCAGCCTGGAACAGAGTGGA

CTGCAGAGGCAATAAACAAGGATGTCGTGAAGACCCTGTTTCTTTCTAGC

GTGGCATCCCAGGATAAAATATACAATTAATAGATACTGGAAGGCACACT

TTCTGATTATGGAGAAACCATCAAATTTGGCCATGGTTTTGCATAGGGGC

TAGGTTGGAAATTCAACTAGAACAATTTAATGTCTACTGGCAAAGCTTCT

TAATTATGGAATGAAAGCTACCTCAAAGTGCCTGGAGGCTTGAGCTGAAT

GGTGAGATCTTCATTGACCTGACCTCCTCTGTGCTGAAGTTTGCTGACTT

GAGGAGACCTCCCCTAAGAACAGACCCTCCCCTCAGGGCTGCCTTCAGCG

ATGATGATTGACGCTGGCAAGTCAGTCAAGTGGGAAGTTTGCAGCACACA

CGGCTCAAAGGACTCACCCAGTTTGCCCTGTTGGAGGATGTTAACCTTTG

CACACTATTTTTGACATTTAATTTTTCCTTCCATTTAGTGCATGACTCTT

TTTTTGTCTTTCTAGTAAATTCATTTAGTTAGTATTATTCTTTGCCTATT

TTGTCTTTTAAACTTCTGGTTTTTAATCTTTCGTATTTTATATTATTTAT

TCTTGGTGTTCATGCTTCTTATTCTCTTTTCTGCTATTTTATCTTATTGT

TGTGATAATAGTATATTTGCAGTAAGCTCCTTTTACTAACTTCATTTATT

TCTACATAATGTTTTATCTGGCTTTGACCACAACTATATAAGGGGACAGC

AAAGTGGAGCCACAAATGGGAAGATTGTGAAGAGGGCCCTTGACCAAATA

AAGAGGAAACCTTTTCCCTGAAGAGGACTTTTCAGCAACCCAACTAATTC

ACTCATTTTTCTCAAAGTTGAATTTTCTTCCACAAAAACCCCTTGACTTT

GTTATTGAGTAGATCCTCAGGCCTAGGAAAATAATGTCTACATCGACCAA

GGAGATATCTGAACTACCAGTACTTCCTGCAGCATCTTTGTTTTGGTTGG

CCTGGGCAGGTGGTGGGGCACGGGGGTGAAGGGAGGGGTCTGAGGTGGGT

AGAAAACAAGGGTATCCCCAGGGCTATGGGAGGAAGGTCTAGTGGACTGG

AACCATTGTATCTGTCGATTAGTGGAGGCAATCATATTTGTGATCCTTGA

GCTAATGGTCTAGTAAGACTATAAACTTGTTCATAATTAAAGCTGGCAAG

AGGGTTCTTACCCCAGGTAATGAGACAAGAGTGAACCACAGGAGGAAGCC

AGCACAGAGACCGTATGTAATGAGTCCCTTCATGGGAAAGGGATGGAAAA

CAGTCCTCAGGCTAGCAGCAGAGGGAGTGCTGTCTGTTGTGGGTCTTGAC

CTGCTTAAGAGGTCTGACTTGAGGTGAGGCTGAAAGACAGCCCCGCTGGG

GGCTTCAAGATAACGATGGACGGATAGAGTTCTCTCACCGCTGAATATCT

CCTAAGGCATTAGTAAAACACTATTTAAATATTTCCTCTGTAACTCAGTT

GTGCTATTAAAGCTCTGTTTCTCAAAGTAGGTCTGTAGATGTTCTTGAGA

GAGTGAGTGTTTCATGAGAATTTTCAAATTTTCTGCTTTCGTTTTGAAGA

TAATCTTAAAGAAATAAAATCAAAATGTTCTACTACATTATTTTTAGTGC

TGAAGGCTGAGTTTGGTGCCAAATGAAGAACAAATAACATCACGGCATGC

TATGGAAATGTCGATTTTGCATTAAGTTTGAATAGAGAAAGATGTGGGAA

AATTGATCAATTTTGTGGCCCAAAGGTAGTACAGACATCATTAGTTCAAA

ATGTCTTCTGTCATAGCAGTGGCATATCATGTTGAGAAGTTATTGTGAGA

CATACCAACTTGAACTGTCATTCAATAGCAGGAAAGAATACTGTTATTTT

TATTCAATAGTATTTTTAATGTTAATCTTCAGTATTTTTATTATTATGTA

CTTATTGTGGTGCACTATTAAATTTATCAGCCCCTCAAACTAATGGATTA

GTAGTTAAACAAGCTTATTTACCAAAAACGATTAATCCTACGATAACTTG

AAGAAAGACTTAAGATATGCTTCCAGTATTGTTCAAAGTCAAAAGAATCA

AGAGGACAGAAAGTGAAGCATAAAACATACAAATAGTAGGTGTATTAAAC

CCAACGCCAGCAATTTAGTTCCAGCAAAATATCATTTATCACACTGATGT

TTTCTATATGAACTCTAGTGGCTTTTGTATGTTTGCTTGTTTTTAATTTG

CAGCTTTGCTTGAGTTTTGAAGTAATTTAAGAGCTATATGTATGGGAAAT

TTATATCTGTTCATTTTGAAGTAACACAATAAAAATAATGTAGAGCAACA

GTA

>hg19_ct_ARAlincRNAs_9727_ARAlincRNA_0299.14 range=chr2:75145275-75145586 5'pad=0 3'pad=0 strand=+ repeatMasking=none

AGAGGAGAGAAAGTGGAGTTTGAGTATAAATAACTCTTTTGGAGAGGATT

GGTGTAATTGAATGGCAGGGGTATGAGATTTGAGGTCAAGGAAATATTTT

TATTATTTTTTACGATGAGAGAAATTGTAGTACACATGTATATTTATGGG

AATGACTCAGTAGAAAGACCAAAAATTTCATATGTGAGAGAAGGACCAAT

TGATGAAGCGATGTTCTTGCGTGGGCGCGAGGATGGGATCGAGTGCACAG

CGGAAGGTGGCACTTAGGAACACGCAGCACTCGTCCATGCCAGCAGGAGG

CAAGGCAGAATG

>hg19_ct_ARAlincRNAs_9727_ARAlincRNA_0299.13 range=chr2:75145328-75170618 5'pad=0 3'pad=0 strand=+ repeatMasking=none

GTAATTGAATGGCAGGGGTATGAGATTTGAGGTCAAGGAAATATTTTTAT

TATTTTTTACGATGAGAGAAATTGTAGTACACATGTATATTTATGGGAAT

GACTCAGTAGAAAGACCAAAAATTTCATATGTGAGAGAAGGACCAATTGA

TGAAGCGATGTTCTTGCGTGGGCGCGAGGATGGGATCGAGTGCACAGCGG

AAGGTGGCACTTAGGAACACGCAGCACTCGTCCATGCCAGCAGGAGGCAA

GGCAGAATGGTAAAAAGACATCCCTTAACTATGGCAAAACAACAACAACA

ACGACGACAAAAAACAAAACAAAACAAAACAAAACCCTGCATCAATTTGA

TAAATAATCCTCCCCGTTGATGTCACTGGTTTTTTTCTGCACATCTTCTC

ACTGATATTTAAATGCTGAGCTTACACTTTCCATGGACATGCTTAATGAC

TAAACAATAGTTGAATTCTAAATTAATCAACTGACCAACCAGTAAGAATT

TATTGAGCAACTCCTTTGAAGCCGTCTATCTGTTAGGTGCTGTGGGAGCA

CAAAAGAAATAGGACATCATCTCTGCCTCAGGGAATTTAGTGTTTTGGGG

AAAACCTGAGTGGTATACACAAAGCAATTGAGGAACAACCTAGGACCTTG

TAGTCCATTAGGATTTCCAAAAATAGAAAGAGAATGGAAAGTCACCTGGC

TAGGAGAAGCCAGTGGAAACTTGACACGAAGAAAAAAGAGCAGCTAATTT

CATTCCTGTCCACCAGTTATTTATGTGTTTATCTTTAATTACATTTGTTT

GATTTCCCTTATTAAAGTCTGATGTCTTAAAAAAGCAGAAAAGTGAGGCA

GGTCAGCAGGGGATGTAAGTTGGGAAGAAAGACAGGTGAGGGCAAGAATT

TAGGCAGGAGCCACAGTGTTGGTTGTGCAGGTGAAGGTCAGGTGACGGAG

GGTAACCAGTCATGGATGACCCAGGCAGGAGCCATAACCAAAATGTTAGA

AAAAGTTGGTAAGAAATTGTTCCAGGCCAGGCGCAGTGGCTCATGCTTGT

AATTCCAGCACTTTGGGAGGCCAAGGTAGGCGGATCACTTGAGGTCAGGA

GTTTGAAACCAGCCTGGCCAACATGGAGATACCCTGTCTCTACTAAAAAT

ACAAAAACTATCTGGGCATGGTGGCACGTGCCTGTAATCCCAGCTACTCA

GGAGGCTGAGGCAGGAGAATCACTTCAACCCAGGAGACAGAGATTGCAGT

GAGCCGAGATTGCGCCACTGCACTCCAGCCTGGGTGACAGAGTGAGACAC

CATCTCAAAAAAAAAAAAAAAAAAAAGTTTTCCAGGAACAAGGGCAGAAC

TTAGCTACTGAGTTCTACCGAAGGCCAGGATCTGAGTCAAGCACCAAGGT

TAACTCCCAGGGAAGCCAAATGTTCATAAAAGGAAATCTTTCCAAATCAC

AAATATCAGGGCCTGCTAGGTAAATTCTAGCTTCTCTGTGCCAAGGGCTG

TGTTCCTACTCTCAAGCGCCACTCACTGACTGTCTGGGTGGTGCTGTGGG

CTTCTGTACTTTTAGCTTCATCAGCTGCACCTGTCCTCTTTTCCCATAAG

ACGCCAGGACCCTCAGAATCTTCCCTCTTACCAGAAATTTGGGTTTTGGG

CTGGGTCCCTGGACCGAATTCTGACACTCTAAGTGTTTGCAGACTATTTT

GATAGAGGTAGTGATTATGATGCCAGAAAAGTGAAGCCAGTTTTTATTAG

AATGTTGGCCATCTTGAATAATCCATTCCCCCACTTCCATGGGACATCTT

CAAAGAGATTTTTAAAAGACAAATATGATCTCTACTAATGAGATATCTTC

AGAAGGTAGAATTAAAGCAAAATTAATGTACACAGAATTCCTATTTCAAT

ATATTTATTATTCACAACATTTCTACACACACACACACACACACACACAC

GCATACACACACACACACACACATTCCTGCACAGGTAATTTATAGTGGCT

AATCCACTCAGTACTGTGGACTTTGGTTTTAGAGAGCATTGTTTTCAAGG

GTAGTACAGATAATCTGCCTCCAACATATTCTATTCTTAGGGCTCTAAAG

TCAAAGGAGTTTGTACAGACCCCTCCCTGCTAAGGTAATTATGTCCTCTT

CATATCTTGCATGAATGTTTGCTCTTAGAGTGATGATCTGAAATATCAAC

ATCCTGCAGGACCTAGTCTGGTCTGGAAGAAGAAGAGATATGTCATCTCT

ATGGGCTTTGATCTTGGTAGGCATATTTATTCACCAGGTACACAAATGCA

ACTTTAAACCCTCAGCCACATTTAGTAAACTATTTCTTCATCTGTTGCAG

TCTTATTCTGATGGGAAAAATTTCCTCTGTTTTTCAAGTTTAATTTATTA

TTTTTTCAAATTACAAGAGCAATGCATATTATTGTAACCAGAGAGATAAT

GCCAAGATCGGTAGAGAAAAACTTAATAATCTCGACCTCCTATAACCATC

TCTCTTCCCATCCCCCTGAGGTAATAGCAAATGTTAACAGCCTGCTACCT

CTGCACATCTTCCTTCCTTCATGATTTCCCTACACACACACCCATACACA

CACGTGCGCATGCACACACACACCCACTCAAACACACGGTGTACGTCCAG

GAGTCAGAAGTGGCTACAGAGATGGAGTCTTCATTATTAAGGTGAGAAAA

TGATAAGACAAATCACCCTTCTATTTCTACCTCCATGGAAAGCTAAATCT

TCCATTTATATTTTTATAGTCTTTTCAGCACTGTCTTAATGGTCTTGAGA

ACATATTACCCTGAAAATGTAAAAATAGAGCCAAAGAGGTGAAAGCTCCT

TGCGTGGCTTCTGACCAGGGATGGCTGCTTCCATGGGCTTTAGCTCTGCC

AGTTCTCCCTAGACTGGGGCAGGTAGGCAGTGAGGATTTCAGCATATATT

AAGTGCCAAATAGAAACAAATATTTGTTTCTATTATTAGAGTAAAACAAC

AGAAGCCTGTATTTTGACCAGAGGCCTGGAATCTACTACTTTGTGAATTA

GCTAAGCAGCCCCAGTTGAACGTGAACCAGCCTGGAACAGAGTGGACTGC

AGAGGCAATAAACAAGGATGTCGTGAAGACCCTGTTTCTTTCTAGCGTGG

CATCCCAGGATAAAATATACAATTAATAGATACTGGAAGGCACACTTTCT

GATTATGGAGAAACCATCAAATTTGGCCATGGTTTTGCATAGGGGCTAGG

TTGGAAATTCAACTAGAACAATTTAATGTCTACTGGCAAAGCTTCTTAAT

TATGGAATGAAAGCTACCTCAAAGTGCCTGGAGGCTTGAGCTGAATGGTG

AGATCTTCATTGACCTGACCTCCTCTGTGCTGAAGTTTGCTGACTTGAGG

AGACCTCCCCTAAGAACAGACCCTCCCCTCAGGGCTGCCTTCAGCGATGA

TGATTGACGCTGGCAAGTCAGTCAAGTGGGAAGTTTGCAGCACACACGGC

TCAAAGGACTCACCCAGTTTGCCCTGTTGGAGGATGTTAACCTTTGCACA

CTATTTTTGACATTTAATTTTTCCTTCCATTTAGTGCATGACTCTTTTTT

TGTCTTTCTAGTAAATTCATTTAGTTAGTATTATTCTTTGCCTATTTTGT

CTTTTAAACTTCTGGTTTTTAATCTTTCGTATTTTATATTATTTATTCTT

GGTGTTCATGCTTCTTATTCTCTTTTCTGCTATTTTATCTTATTGTTGTG

ATAATAGTATATTTGCAGTAAGCTCCTTTTACTAACTTCATTTATTTCTA

CATAATGTTTTATCTGGCTTTGACCACAACTATATAAGGGGACAGCAAAG

TGGAGCCACAAATGGGAAGATTGTGAAGAGGGCCCTTGACCAAATAAAGA

GGAAACCTTTTCCCTGAAGAGGACTTTTCAGCAACCCAACTAATTCACTC

ATTTTTCTCAAAGTTGAATTTTCTTCCACAAAAACCCCTTGACTTTGTTA

TTGAGTAGATCCTCAGGCCTAGGAAAATAATGTCTACATCGACCAAGGAG

ATATCTGAACTACCAGTACTTCCTGCAGCATCTTTGTTTTGGTTGGCCTG

GGCAGGTGGTGGGGCACGGGGGTGAAGGGAGGGGTCTGAGGTGGGTAGAA

AACAAGGGTATCCCCAGGGCTATGGGAGGAAGGTCTAGTGGACTGGAACC

ATTGTATCTGTCGATTAGTGGAGGCAATCATATTTGTGATCCTTGAGCTA

ATGGTCTAGTAAGACTATAAACTTGTTCATAATTAAAGCTGGCAAGAGGG

TTCTTACCCCAGGTAATGAGACAAGAGTGAACCACAGGAGGAAGCCAGCA

CAGAGACCGTATGTAATGAGTCCCTTCATGGGAAAGGGATGGAAAACAGT

CCTCAGGCTAGCAGCAGAGGGAGTGCTGTCTGTTGTGGGTCTTGACCTGC

TTAAGAGGTCTGACTTGAGGTGAGGCTGAAAGACAGCCCCGCTGGGGGCT

TCAAGATAACGATGGACGGATAGAGTTCTCTCACCGCTGAATATCTCCTA

AGGCATTAGTAAAACACTATTTAAATATTTCCTCTGTAACTCAGTTGTGC

TATTAAAGCTCTGTTTCTCAAAGTAGGTCTGTAGATGTTCTTGAGAGAGT

GAGTGTTTCATGAGAATTTTCAAATTTTCTGCTTTCGTTTTGAAGATAAT

CTTAAAGAAATAAAATCAAAATGTTCTACTACATTATTTTTAGTGCTGAA

GGCTGAGTTTGGTGCCAAATGAAGAACAAATAACATCACGGCATGCTATG

GAAATGTCGATTTTGCATTAAGTTTGAATAGAGAAAGATGTGGGAAAATT

GATCAATTTTGTGGCCCAAAGGTAGTACAGACATCATTAGTTCAAAATGT

CTTCTGTCATAGCAGTGGCATATCATGTTGAGAAGTTATTGTGAGACATA

CCAACTTGAACTGTCATTCAATAGCAGGAAAGAATACTGTTATTTTTATT

CAATAGTATTTTTAATGTTAATCTTCAGTATTTTTATTATTATGTACTTA

TTGTGGTGCACTATTAAATTTATCAGCCCCTCAAACTAATGGATTAGTAG

TTAAACAAGCTTATTTACCAAAAACGATTAATCCTACGATAACTTGAAGA

AAGACTTAAGATATGCTTCCAGTATTGTTCAAAGTCAAAAGAATCAAGAG

GACAGAAAGTGAAGCATAAAACATACAAATAGTAGGTGTATTAAACCCAA

CGCCAGCAATTTAGTTCCAGCAAAATATCATTTATCACACTGATGTTTTC

TATATGAACTCTAGTGGCTTTTGTATGTTTGCTTGTTTTTAATTTGCAGC

TTTGCTTGAGTTTTGAAGTAATTTAAGAGCTATATGTATGGGAAATTTAT

ATCTGTTCATTTTGAAGTAACACAATAAAAATAATGTAGAGCA

>hg19_ct_ARAlincRNAs_9727_ARAlincRNA_0299.11 range=chr2:75155366-75165913 5'pad=0 3'pad=0 strand=+ repeatMasking=none

GAGTTTGATGTCATCACCCTGCAAACGATGAAACAAATCCCCATTTTATT

GATGACTGATAACTTCAAATGTGACAAGAGGATTCATTTCCTTATTCAAT

CATTCCACAAATATTCACTGAGCATCTCCTACGTGCCAGGCACCAGGCTG

GACTTTAGGATCAATCATAATAATTTTCAGGTTATGGATAATGCCAATGG

GCACTTAGGATGATCAGGGAGGGCAGTTCTGAGAAGGGGCATATGAGCTG

TGTTCCAGTTGTCTATTGCTGTGTGACAAACTGCCCCAAAACTTAATGGC

TTAAAACAATGACAGAAATTACATTACTCATGAATCTGCAGTTTAGGCAG

GTGGGGACAGCATGTCTCTGCTCCACCTAACATCAGCTGAAGGTGGGAAC

TGGAATCATCTGGAGGCTTCCTCACTTACATGTCTAGTGGTTGATGCTGA

CTGATGTTGGCTGTTGGCAAGGACCTCTGACAGGTCTGTTCCCCGAAACA

CCTGCATGTGGTACATGGCTTTGTCACAGCATGATGGCTGGGTTCCAAGG

GTGAGCCTCACAAGACAGAGAACCAGGTGGAGGCTGTGCCATCTTTGGTC

ACCTAGTCTAAGAAGTTACAGTCACTTCTATACCATTTAATTCATTAGTA

GCAAGCCACTGAGGCTAGTCCATGTTCAAGAGGAGGGGAATTGGATTCCC

CCTTTTTTTAGGATAGAAAAGAATTTGTGGACATGTTTTCAAATGACCAC

AAACTATGACCTAAAGAAATTAGTGGAAGAGCATTCCAGGAAGAATTGTA

AAGTCTCTGATGCTGGAAAGGCTTGGTGTGTTTAAGGAAATGAAAGGCCA

GAAAGGCTTGAGCACAGCAAGGGCAGAAGGTAAGATTTGAGGTTAGATGT

GGACAGAAGCCATATTAGGCAGTTATAGGTGCTTGGATTTTATTCTGGAT

GCAGTGAGGGCTGTTGAAAGATTTAAGGTTTGTATTCACTTCTTGAAAGG

ATCACTCCAGCTAATATGCAGAGAATGGATTGAGGTGAGATAAGGGTAGG

GAAAACCAGTTAGGATGCTATTGGAGTGGTTTGGGGAGAGAGAGTGATGG

CTTGGACAAGAATGTTGGCACTGGAGATGAAAAGAAGTAGATAGAAATGG

TGGAAGTAAATGAACAAGTCTACCAGGAGACTGAATGTGAGAGAAGGAAA

GGAAGGCAGGAAACAAGAATTCTTGGGTTTTAGGCTCATGTCCATCTTGA

TGGTGGAGCCCTTTCTGTGATTGGAAGACTAAGAGAGGAACTTGTTGATA

CGGGCAAAAGGTAAAATCCAGAGTCCAGTTCTAGGCACGTCAAACTTGAG

AGGTTTGTAAGGTATACTGAAGGCAAGAAGTAGACATTTGGATATATGAG

CCTGAAACTCAGTGGAGAGCTTGGGACTGGAAATATAAATTTGGGAATTA

TCAGCATATGGATGGTATTTAAAGCTGTAGGCCTCGGTGAGGTCACCAGG

GGAAAATCTAGAACAAAAGAGTGTGGAGACTGAGCCCTGTAAAGGGTAGC

CTGCAAAGGAGATTGAAAAGGAGTGGCCAAAAAAGACAGCAGGGAAACTA

GGGCATTGGATGTTATAGATACCCGAGAAAGCGTTGGTCTGAATGACCGT

TAAGACTCCTCCAGATATGAATTTCTGTAACTTTATGGATGTTTCAAATC

CCTTCAAGCTGGGAAATTCTGTCTCTGTTGCATCTTTGGGATAACAAACT

TCCTGAAGAATCACTGTGTCTTTTCAAAGACTGAAAATGGATTCCCAGCA

ATGTGAAATCTTGTGCCTGAGTCCTAAAGTGATCAAAGGTAAGGTTGAGC

CTAGGACAAAATGAGGATCCCCTGGAGGGAAGCTAGGGCAACACAGTAAG

TGGCAAAAGAATACGAGTGAGGTTATTTGACTATCCTCTTCCAGTAGGGC

TGGCTCAGCTATGGCAGACCCCAGGGTGGTTAGGACAAATGGCAGCCCCA

TACAGGGACATACATGGACACTGATCCTTGTTTTTTTCCTTTTTGGGGCC

TTTCTGAATCTCCACTCCATGTGCAGGGTTGACCTGTTTCCTTGAGGAAC

TCTGAGATTTGCATCAATGGAGTTATCTGAGGGCTCTGTGGCTTGGGCTC

TGTGCAGGAGGGCAGCCTGGCAATTGGATTGTTCCTGCTGGCTGGTGCCA

TTGTGTGATATTGAGTCACCAGAATGGTGCCGATGCACTGCTTTTGGGTG

ATAATCAGGCGCAGGATGAATTCGGGGCACTGGGCTACTGATGGTCAACT

GTTAGCACCTGGGCTTGGGCTGTGTGTGGGCACCCATGCCTCAGCTCTCA

CCTGTTCATGTTCCTCCATGGTACCATCTCTGATTTGTGGCGTTCAAAGG

CGTAGGATGGCAGCAAGCCTCCTTTACCCTGTATATCCATCCCCTTGCTG

TTGGCACCTAAGGTTGTCACCCCATGCCCTCATCAGTCTCTGTACCACAT

ATCAGGAAGTCCTCCAGTGGTGGCTATGCCTGCCTTCTCTGAGGACTTTA

AAGACCTTGGCTTTGCCACGCCAACAGGCTCTTCCAACCAGTGCCTTTCC

TCCAAATGATATGGCGATGTTTTGCTTTCCCAGGTCGATCTTAAGCCATT

CCCAACAAGGAGTGGACATCCTTGTGTGAAAAACACTGTTTTTCTTGCTC

CAGTCACGTCTCCTTGCCTTTCTTGTCTTTTGGTGGTAGTGTGCAGAAGC

AGAGGTGCAGGAAATGTTGAAAGGGATCACATTCTAAGAATGTAGTTATA

ATGGCCTGAAGAATTCAAGAGAATACATGGTTGGAAGATGTGTCACTTTA

TGGTTACACTATAAAACTCCAAATGAAAAATAAATTATCATTCATACTTT

CCTGAATATTCTGGGTAAGGCTTGCATTTTGGTCATTATTAATGAAGTAC

AGAAACAACCTCTGAGAGAGTGGTTGGATGCCAGTATTTGCAGGTGCCAC

GATGAATGATGCAGCACTATCAGTCAAGATGCAACCAACATTAGACAAAT

GGTATCAGAGAACCACAGAGGCGAAAAAACAAAACCAAAACCCAAAACTA

TATTGTTTCAATACAAACAGGTTCCAAAAGCTATGACATCGTGAGTGACA

ACAATGTTGCCGCCTCTTTCTCACAATAGGAAATAAAACTTTAACCAAAA

GAGCATGGGCAGATATTTCCCGGATCTGAACAGGAATAGCTGGTGTCTGG

ACTCTATGAAGAATCTTCATGTGCAGTGATAGAAGAATAAACACATTTGT

AAAAAGACATCCCTTAACTATGGCAAAACAACAACAACAACGACGACAAA

AAACAAAACAAAACAAAACAAAACCCTGCATCAATTTGATAAATAATCCT

CCCCGTTGATGTCACTGGTTTTTTTCTGCACATCTTCTCACTGATATTTA

AATGCTGAGCTTACACTTTCCATGGACATGCTTAATGACTAAACAATAGT

TGAATTCTAAATTAATCAACTGACCAACCAGTAAGAATTTATTGAGCAAC

TCCTTTGAAGCCGTCTATCTGTTAGGTGCTGTGGGAGCACAAAAGAAATA

GGACATCATCTCTGCCTCAGGGAATTTAGTGTTTTGGGGAAAACCTGAGT

GGTATACACAAAGCAATTGAGGAACAACCTAGGACCTTGTAGTCCATTAG

GATTTCCAAAAATAGAAAGAGAATGGAAAGTCACCTGGCTAGGAGAAGCC

AGTGGAAACTTGACACGAAGAAAAAAG

>hg19_ct_ARAlincRNAs_9727_ARAlincRNA_0299.10 range=chr2:75155366-75170624 5'pad=0 3'pad=0 strand=+ repeatMasking=none

GAGTTTGATGTCATCACCCTGCAAACGATGAAACAAATCCCCATTTTATT

GATGACTGATAACTTCAAATGTGACAAGAGGATTCATTTCCTTATTCAAT

CATTCCACAAATATTCACTGAGCATCTCCTACGTGCCAGGCACCAGGCTG

GACTTTAGGATCAATCATAATAATTTTCAGGTTATGGATAATGCCAATGG

GCACTTAGGATGATCAGGGAGGGCAGTTCTGAGAAGGGGCATATGAGCTG

TGTTCCAGTTGTCTATTGCTGTGTGACAAACTGCCCCAAAACTTAATGGC

TTAAAACAATGACAGAAATTACATTACTCATGAATCTGCAGTTTAGGCAG

GTGGGGACAGCATGTCTCTGCTCCACCTAACATCAGCTGAAGGTGGGAAC

TGGAATCATCTGGAGGCTTCCTCACTTACATGTCTAGTGGTTGATGCTGA

CTGATGTTGGCTGTTGGCAAGGACCTCTGACAGGTCTGTTCCCCGAAACA

CCTGCATGTGGTACATGGCTTTGTCACAGCATGATGGCTGGGTTCCAAGG

GTGAGCCTCACAAGACAGAGAACCAGGTGGAGGCTGTGCCATCTTTGGTC

ACCTAGTCTAAGAAGTTACAGTCACTTCTATACCATTTAATTCATTAGTA

GCAAGCCACTGAGGCTAGTCCATGTTCAAGAGGAGGGGAATTGGATTCCC

CCTTTTTTTAGGATAGAAAAGAATTTGTGGACATGTTTTCAAATGACCAC

AAACTATGACCTAAAGAAATTAGTGGAAGAGCATTCCAGGAAGAATTGTA

AAGTCTCTGATGCTGGAAAGGCTTGGTGTGTTTAAGGAAATGAAAGGCCA

GAAAGGCTTGAGCACAGCAAGGGCAGAAGGTAAGATTTGAGGTTAGATGT

GGACAGAAGCCATATTAGGCAGTTATAGGTGCTTGGATTTTATTCTGGAT

GCAGTGAGGGCTGTTGAAAGATTTAAGGTTTGTATTCACTTCTTGAAAGG

ATCACTCCAGCTAATATGCAGAGAATGGATTGAGGTGAGATAAGGGTAGG

GAAAACCAGTTAGGATGCTATTGGAGTGGTTTGGGGAGAGAGAGTGATGG

CTTGGACAAGAATGTTGGCACTGGAGATGAAAAGAAGTAGATAGAAATGG

TGGAAGTAAATGAACAAGTCTACCAGGAGACTGAATGTGAGAGAAGGAAA

GGAAGGCAGGAAACAAGAATTCTTGGGTTTTAGGCTCATGTCCATCTTGA

TGGTGGAGCCCTTTCTGTGATTGGAAGACTAAGAGAGGAACTTGTTGATA

CGGGCAAAAGGTAAAATCCAGAGTCCAGTTCTAGGCACGTCAAACTTGAG

AGGTTTGTAAGGTATACTGAAGGCAAGAAGTAGACATTTGGATATATGAG

CCTGAAACTCAGTGGAGAGCTTGGGACTGGAAATATAAATTTGGGAATTA

TCAGCATATGGATGGTATTTAAAGCTGTAGGCCTCGGTGAGGTCACCAGG

GGAAAATCTAGAACAAAAGAGTGTGGAGACTGAGCCCTGTAAAGGGTAGC

CTGCAAAGGAGATTGAAAAGGAGTGGCCAAAAAAGACAGCAGGGAAACTA

GGGCATTGGATGTTATAGATACCCGAGAAAGCGTTGGTCTGAATGACCGT

TAAGACTCCTCCAGATATGAATTTCTGTAACTTTATGGATGTTTCAAATC

CCTTCAAGCTGGGAAATTCTGTCTCTGTTGCATCTTTGGGATAACAAACT

TCCTGAAGAATCACTGTGTCTTTTCAAAGACTGAAAATGGATTCCCAGCA

ATGTGAAATCTTGTGCCTGAGTCCTAAAGTGATCAAAGGTAAGGTTGAGC

CTAGGACAAAATGAGGATCCCCTGGAGGGAAGCTAGGGCAACACAGTAAG

TGGCAAAAGAATACGAGTGAGGTTATTTGACTATCCTCTTCCAGTAGGGC

TGGCTCAGCTATGGCAGACCCCAGGGTGGTTAGGACAAATGGCAGCCCCA

TACAGGGACATACATGGACACTGATCCTTGTTTTTTTCCTTTTTGGGGCC

TTTCTGAATCTCCACTCCATGTGCAGGGTTGACCTGTTTCCTTGAGGAAC

TCTGAGATTTGCATCAATGGAGTTATCTGAGGGCTCTGTGGCTTGGGCTC

TGTGCAGGAGGGCAGCCTGGCAATTGGATTGTTCCTGCTGGCTGGTGCCA

TTGTGTGATATTGAGTCACCAGAATGGTGCCGATGCACTGCTTTTGGGTG

ATAATCAGGCGCAGGATGAATTCGGGGCACTGGGCTACTGATGGTCAACT

GTTAGCACCTGGGCTTGGGCTGTGTGTGGGCACCCATGCCTCAGCTCTCA

CCTGTTCATGTTCCTCCATGGTACCATCTCTGATTTGTGGCGTTCAAAGG

CGTAGGATGGCAGCAAGCCTCCTTTACCCTGTATATCCATCCCCTTGCTG

TTGGCACCTAAGGTTGTCACCCCATGCCCTCATCAGTCTCTGTACCACAT

ATCAGGAAGTCCTCCAGTGGTGGCTATGCCTGCCTTCTCTGAGGACTTTA

AAGACCTTGGCTTTGCCACGCCAACAGGCTCTTCCAACCAGTGCCTTTCC

TCCAAATGATATGGCGATGTTTTGCTTTCCCAGGTCGATCTTAAGCCATT

CCCAACAAGGAGTGGACATCCTTGTGTGAAAAACACTGTTTTTCTTGCTC

CAGTCACGTCTCCTTGCCTTTCTTGTCTTTTGGTGGTAGTGTGCAGAAGC

AGAGGTGCAGGAAATGTTGAAAGGGATCACATTCTAAGAATGTAGTTATA

ATGGCCTGAAGAATTCAAGAGAATACATGGTTGGAAGATGTGTCACTTTA

TGGTTACACTATAAAACTCCAAATGAAAAATAAATTATCATTCATACTTT

CCTGAATATTCTGGGTAAGGCTTGCATTTTGGTCATTATTAATGAAGTAC

AGAAACAACCTCTGAGAGAGTGGTTGGATGCCAGTATTTGCAGGTGCCAC

GATGAATGATGCAGCACTATCAGTCAAGATGCAACCAACATTAGACAAAT

GGTATCAGAGAACCACAGAGGCGAAAAAACAAAACCAAAACCCAAAACTA

TATTGTTTCAATACAAACAGGTTCCAAAAGCTATGACATCGTGAGTGACA

ACAATGTTGCCGCCTCTTTCTCACAATAGGAAATAAAACTTTAACCAAAA

GAGCATGGGCAGATATTTCCCGGATCTGAACAGGAATAGCTGGTGTCTGG

ACTCTATGAAGAATCTTCATGTGCAGTGATAGAAGAATAAACACATTTGA

ACCAAGTTCACCATGGAGAGAGTGAGTGGGTTGGGGAAAAGGTGTTGTAT

GTTTGAGGAGCGAGGAGAAGGTGTCAAATAATCATCCAGGCAGACTTTTG

TAGTGCACAGTGACCGGTGACCAGCAGCATGTGTTTCCATAGGCATCTTC

TCTCAGTACCCCAGAAGGTGAATTGCCAGTGAGTCCTGCCAGGATGCCCC

TCGGTGACTGCACCACCCAGTAAGTCATGGCTGTGTACTTCCCATCAAGG

TCTGGATCTCAGCTCAGGTAGTGGGGGCTGCTCTTCCATAGTCATTCCAT

CCTTGTGTGCTCTGCCTCAGCCCTGGGGATAGTGGCCGCTTTCTGTACAT

GTCAGTCCTCTATTCTTTAGACTTCTTGTTACCCCATTAGCAGCCAATTC

TCTTTTGTTACTGCTTAGCAGCCAACTTTTTTTATATTAAACTTTCCCCA

ACCGGGACAGCAAAGTGGAGCCACAAATGGGAAGATTGTGAAGAGGGCCC

TTGACCAAATAAAGAGGAAACCTTTTCCCTGAAGAGGACTTTTCAGCAAC

CCAACTAATTCACTCATTTTTCTCAAAGTTGAATTTTCTTCCACAAAAAC

CCCTTGACTTTGTTATTGAGTAGATCCTCAGGCCTAGGAAAATAATGTCT

ACATCGACCAAGGAGATATCTGAACTACCAGTACTTCCTGCAGCATCTTT

GTTTTGGTTGGCCTGGGCAGGTGGTGGGGCACGGGGGTGAAGGGAGGGGT

CTGAGGTGGGTAGAAAACAAGGGTATCCCCAGGGCTATGGGAGGAAGGTC

TAGTGGACTGGAACCATTGTATCTGTCGATTAGTGGAGGCAATCATATTT

GTGATCCTTGAGCTAATGGTCTAGTAAGACTATAAACTTGTTCATAATTA

AAGCTGGCAAGAGGGTTCTTACCCCAGGTAATGAGACAAGAGTGAACCAC

AGGAGGAAGCCAGCACAGAGACCGTATGTAATGAGTCCCTTCATGGGAAA

GGGATGGAAAACAGTCCTCAGGCTAGCAGCAGAGGGAGTGCTGTCTGTTG

TGGGTCTTGACCTGCTTAAGAGGTCTGACTTGAGGTGAGGCTGAAAGACA

GCCCCGCTGGGGGCTTCAAGATAACGATGGACGGATAGAGTTCTCTCACC

GCTGAATATCTCCTAAGGCATTAGTAAAACACTATTTAAATATTTCCTCT

GTAACTCAGTTGTGCTATTAAAGCTCTGTTTCTCAAAGTAGGTCTGTAGA

TGTTCTTGAGAGAGTGAGTGTTTCATGAGAATTTTCAAATTTTCTGCTTT

CGTTTTGAAGATAATCTTAAAGAAATAAAATCAAAATGTTCTACTACATT

ATTTTTAGTGCTGAAGGCTGAGTTTGGTGCCAAATGAAGAACAAATAACA

TCACGGCATGCTATGGAAATGTCGATTTTGCATTAAGTTTGAATAGAGAA

AGATGTGGGAAAATTGATCAATTTTGTGGCCCAAAGGTAGTACAGACATC

ATTAGTTCAAAATGTCTTCTGTCATAGCAGTGGCATATCATGTTGAGAAG

TTATTGTGAGACATACCAACTTGAACTGTCATTCAATAGCAGGAAAGAAT

ACTGTTATTTTTATTCAATAGTATTTTTAATGTTAATCTTCAGTATTTTT

ATTATTATGTACTTATTGTGGTGCACTATTAAATTTATCAGCCCCTCAAA

CTAATGGATTAGTAGTTAAACAAGCTTATTTACCAAAAACGATTAATCCT

ACGATAACTTGAAGAAAGACTTAAGATATGCTTCCAGTATTGTTCAAAGT

CAAAAGAATCAAGAGGACAGAAAGTGAAGCATAAAACATACAAATAGTAG

GTGTATTAAACCCAACGCCAGCAATTTAGTTCCAGCAAAATATCATTTAT

CACACTGATGTTTTCTATATGAACTCTAGTGGCTTTTGTATGTTTGCTTG

TTTTTAATTTGCAGCTTTGCTTGAGTTTTGAAGTAATTTAAGAGCTATAT

GTATGGGAAATTTATATCTGTTCATTTTGAAGTAACACAATAAAAATAAT

GTAGAGCAACAGTA

>hg19_ct_ARAlincRNAs_9727_ARAlincRNA_0299.6 range=chr2:75158545-75159416 5'pad=0 3'pad=0 strand=+ repeatMasking=none

GAAATAAAACTTTAACCAAAAGAGCATGGGCAGATATTTCCCGGATCTGA

ACAGGAATAGCTGGTGTCTGGACTCTATGAAGAATCTTCATGTGCAGTGA

TAGAAGAATAAACACATTTGTAAGAACCAAGTTCACCATGGAGAGAGTGA

GTGGGTTGGGGAAAAGGTGTTGTATGTTTGAGGAGCGAGGAGAAGGTGTC

AAATAATCATCCAG

>hg19_ct_ARAlincRNAs_9727_ARAlincRNA_0299.9 range=chr2:75158545-75159917 5'pad=0 3'pad=0 strand=+ repeatMasking=none

GAAATAAAACTTTAACCAAAAGAGCATGGGCAGATATTTCCCGGATCTGA

ACAGGAATAGCTGGTGTCTGGACTCTATGAAGAATCTTCATGTGCAGTGA

TAGAAGAATAAACACATTTGCAGACTTTTGTAGTGCACAGTGACCGGTGA

CCAGCAGCATGTGTTTCCATAGGCATCTTCTCTCAGTACCCCAGAAGGTG

AATTGCCAGTGAGTCCTGCCAGGATGCCCCTCGGTGACTGCACCACCCA

>hg19_ct_ARAlincRNAs_9727_ARAlincRNA_0299.15 range=chr2:75158545-75159921 5'pad=0 3'pad=0 strand=+ repeatMasking=none

GAAATAAAACTTTAACCAAAAGAGCATGGGCAGATATTTCCCGGATCTGA

ACAGGAATAGCTGGTGTCTGGACTCTATGAAGAATCTTCATGTGCAGTGA

TAGAAGAATAAACACATTTGCAGACTTTTGTAGTGCACAGTGACCGGTGA

CCAGCAGCATGTGTTTCCATAGGCATCTTCTCTCAGTACCCCAGAAGGTG

AATTGCCAGTGAGTCCTGCCAGGATGCCCCTCGGTGACTGCACCACCCAG

TAA

>hg19_ct_ARAlincRNAs_9727_ARAlincRNA_0299.5 range=chr2:75158545-75170624 5'pad=0 3'pad=0 strand=+ repeatMasking=none

GAAATAAAACTTTAACCAAAAGAGCATGGGCAGATATTTCCCGGATCTGA

ACAGGAATAGCTGGTGTCTGGACTCTATGAAGAATCTTCATGTGCAGTGA

TAGAAGAATAAACACATTTAGGGGACACTGGAGGATGAACAGTATTTCCT

AGTTTAATGTTTTTTTTTTTTTTTCTAGGCAGACTTTTGTAGTGCACAGT

GACCGGTGACCAGCAGCATGTGTTTCCATAGGCATCTTCTCTCAGTACCC

CAGAAGGTGAATTGCCAGTGAGTCCTGCCAGGATGCCCCTCGGTGACTGC

ACCACCCAGTAAAAAGACATCCCTTAACTATGGCAAAACAACAACAACAA

CGACGACAAAAAACAAAACAAAACAAAACAAAACCCTGCATCAATTTGAT

AAATAATCCTCCCCGTTGATGTCACTGGTTTTTTTCTGCACATCTTCTCA

CTGATATTTAAATGCTGAGCTTACACTTTCCATGGACATGCTTAATGACT

AAACAATAGTTGAATTCTAAATTAATCAACTGACCAACCAGTAAGAATTT

ATTGAGCAACTCCTTTGAAGCCGTCTATCTGTTAGGTGCTGTGGGAGCAC

AAAAGAAATAGGACATCATCTCTGCCTCAGGGAATTTAGTGTTTTGGGGA

AAACCTGAGTGGTATACACAAAGCAATTGAGGAACAACCTAGGACCTTGT

AGTCCATTAGGATTTCCAAAAATAGAAAGAGAATGGAAAGTCACCTGGCT

AGGAGAAGCCAGTGGAAACTTGACACGAAGAAAAAAGAGCAGCTAATTTC

ATTCCTGTCCACCAGTTATTTATGTGTTTATCTTTAATTACATTTGTTTG

ATTTCCCTTATTAAAGTCTGATGTCTTAAAAAAGCAGAAAAGTGAGGCAG

GTCAGCAGGGGATGTAAGTTGGGAAGAAAGACAGGTGAGGGCAAGAATTT

AGGCAGGAGCCACAGTGTTGGTTGTGCAGGTGAAGGTCAGGTGACGGAGG

GTAACCAGTCATGGATGACCCAGGCAGGAGCCATAACCAAAATGTTAGAA

AAAGTTGGTAAGAAATTGTTCCAGGCCAGGCGCAGTGGCTCATGCTTGTA

ATTCCAGCACTTTGGGAGGCCAAGGTAGGCGGATCACTTGAGGTCAGGAG

TTTGAAACCAGCCTGGCCAACATGGAGATACCCTGTCTCTACTAAAAATA

CAAAAACTATCTGGGCATGGTGGCACGTGCCTGTAATCCCAGCTACTCAG

GAGGCTGAGGCAGGAGAATCACTTCAACCCAGGAGACAGAGATTGCAGTG

AGCCGAGATTGCGCCACTGCACTCCAGCCTGGGTGACAGAGTGAGACACC

ATCTCAAAAAAAAAAAAAAAAAAAAGTTTTCCAGGAACAAGGGCAGAACT

TAGCTACTGAGTTCTACCGAAGGCCAGGATCTGAGTCAAGCACCAAGGTT

AACTCCCAGGGAAGCCAAATGTTCATAAAAGGAAATCTTTCCAAATCACA

AATATCAGGGCCTGCTAGGTAAATTCTAGCTTCTCTGTGCCAAGGGCTGT

GTTCCTACTCTCAAGCGCCACTCACTGACTGTCTGGGTGGTGCTGTGGGC

TTCTGTACTTTTAGCTTCATCAGCTGCACCTGTCCTCTTTTCCCATAAGA

CGCCAGGACCCTCAGAATCTTCCCTCTTACCAGAAATTTGGGTTTTGGGC

TGGGTCCCTGGACCGAATTCTGACACTCTAAGTGTTTGCAGACTATTTTG

ATAGAGGTAGTGATTATGATGCCAGAAAAGTGAAGCCAGTTTTTATTAGA

ATGTTGGCCATCTTGAATAATCCATTCCCCCACTTCCATGGGACATCTTC

AAAGAGATTTTTAAAAGACAAATATGATCTCTACTAATGAGATATCTTCA

GAAGGTAGAATTAAAGCAAAATTAATGTACACAGAATTCCTATTTCAATA

TATTTATTATTCACAACATTTCTACACACACACACACACACACACACACG

CATACACACACACACACACACATTCCTGCACAGGTAATTTATAGTGGCTA

ATCCACTCAGTACTGTGGACTTTGGTTTTAGAGAGCATTGTTTTCAAGGG

TAGTACAGATAATCTGCCTCCAACATATTCTATTCTTAGGGCTCTAAAGT

CAAAGGAGTTTGTACAGACCCCTCCCTGCTAAGGTAATTATGTCCTCTTC

ATATCTTGCATGAATGTTTGCTCTTAGAGTGATGATCTGAAATATCAACA

TCCTGCAGGACCTAGTCTGGTCTGGAAGAAGAAGAGATATGTCATCTCTA

TGGGCTTTGATCTTGGTAGGCATATTTATTCACCAGGTACACAAATGCAA

CTTTAAACCCTCAGCCACATTTAGTAAACTATTTCTTCATCTGTTGCAGT

CTTATTCTGATGGGAAAAATTTCCTCTGTTTTTCAAGTTTAATTTATTAT

TTTTTCAAATTACAAGAGCAATGCATATTATTGTAACCAGAGAGATAATG

CCAAGATCGGTAGAGAAAAACTTAATAATCTCGACCTCCTATAACCATCT

CTCTTCCCATCCCCCTGAGGTAATAGCAAATGTTAACAGCCTGCTACCTC

TGCACATCTTCCTTCCTTCATGATTTCCCTACACACACACCCATACACAC

ACGTGCGCATGCACACACACACCCACTCAAACACACGGTGTACGTCCAGG

AGTCAGAAGTGGCTACAGAGATGGAGTCTTCATTATTAAGGTGAGAAAAT

GATAAGACAAATCACCCTTCTATTTCTACCTCCATGGAAAGCTAAATCTT

CCATTTATATTTTTATAGTCTTTTCAGCACTGTCTTAATGGTCTTGAGAA

CATATTACCCTGAAAATGTAAAAATAGAGCCAAAGAGGTGAAAGCTCCTT

GCGTGGCTTCTGACCAGGGATGGCTGCTTCCATGGGCTTTAGCTCTGCCA

GTTCTCCCTAGACTGGGGCAGGTAGGCAGTGAGGATTTCAGCATATATTA

AGTGCCAAATAGAAACAAATATTTGTTTCTATTATTAGAGTAAAACAACA

GAAGCCTGTATTTTGACCAGAGGCCTGGAATCTACTACTTTGTGAATTAG

CTAAGCAGCCCCAGTTGAACGTGAACCAGCCTGGAACAGAGTGGACTGCA

GAGGCAATAAACAAGGATGTCGTGAAGACCCTGTTTCTTTCTAGCGTGGC

ATCCCAGGATAAAATATACAATTAATAGATACTGGAAGGCACACTTTCTG

ATTATGGAGAAACCATCAAATTTGGCCATGGTTTTGCATAGGGGCTAGGT

TGGAAATTCAACTAGAACAATTTAATGTCTACTGGCAAAGCTTCTTAATT

ATGGAATGAAAGCTACCTCAAAGTGCCTGGAGGCTTGAGCTGAATGGTGA

GATCTTCATTGACCTGACCTCCTCTGTGCTGAAGTTTGCTGACTTGAGGA

GACCTCCCCTAAGAACAGACCCTCCCCTCAGGGCTGCCTTCAGCGATGAT

GATTGACGCTGGCAAGTCAGTCAAGTGGGAAGTTTGCAGCACACACGGCT

CAAAGGACTCACCCAGTTTGCCCTGTTGGAGGATGTTAACCTTTGCACAC

TATTTTTGACATTTAATTTTTCCTTCCATTTAGTGCATGACTCTTTTTTT

GTCTTTCTAGTAAATTCATTTAGTTAGTATTATTCTTTGCCTATTTTGTC

TTTTAAACTTCTGGTTTTTAATCTTTCGTATTTTATATTATTTATTCTTG

GTGTTCATGCTTCTTATTCTCTTTTCTGCTATTTTATCTTATTGTTGTGA

TAATAGTATATTTGCAGTAAGCTCCTTTTACTAACTTCATTTATTTCTAC

ATAATGTTTTATCTGGCTTTGACCACAACTATATAAGGGGACAGCAAAGT

GGAGCCACAAATGGGAAGATTGTGAAGAGGGCCCTTGACCAAATAAAGAG

GAAACCTTTTCCCTGAAGAGGACTTTTCAGCAACCCAACTAATTCACTCA

TTTTTCTCAAAGTTGAATTTTCTTCCACAAAAACCCCTTGACTTTGTTAT

TGAGTAGATCCTCAGGCCTAGGAAAATAATGTCTACATCGACCAAGGAGA

TATCTGAACTACCAGTACTTCCTGCAGCATCTTTGTTTTGGTTGGCCTGG

GCAGGTGGTGGGGCACGGGGGTGAAGGGAGGGGTCTGAGGTGGGTAGAAA

ACAAGGGTATCCCCAGGGCTATGGGAGGAAGGTCTAGTGGACTGGAACCA

TTGTATCTGTCGATTAGTGGAGGCAATCATATTTGTGATCCTTGAGCTAA

TGGTCTAGTAAGACTATAAACTTGTTCATAATTAAAGCTGGCAAGAGGGT

TCTTACCCCAGGTAATGAGACAAGAGTGAACCACAGGAGGAAGCCAGCAC

AGAGACCGTATGTAATGAGTCCCTTCATGGGAAAGGGATGGAAAACAGTC

CTCAGGCTAGCAGCAGAGGGAGTGCTGTCTGTTGTGGGTCTTGACCTGCT

TAAGAGGTCTGACTTGAGGTGAGGCTGAAAGACAGCCCCGCTGGGGGCTT

CAAGATAACGATGGACGGATAGAGTTCTCTCACCGCTGAATATCTCCTAA

GGCATTAGTAAAACACTATTTAAATATTTCCTCTGTAACTCAGTTGTGCT

ATTAAAGCTCTGTTTCTCAAAGTAGGTCTGTAGATGTTCTTGAGAGAGTG

AGTGTTTCATGAGAATTTTCAAATTTTCTGCTTTCGTTTTGAAGATAATC

TTAAAGAAATAAAATCAAAATGTTCTACTACATTATTTTTAGTGCTGAAG

GCTGAGTTTGGTGCCAAATGAAGAACAAATAACATCACGGCATGCTATGG

AAATGTCGATTTTGCATTAAGTTTGAATAGAGAAAGATGTGGGAAAATTG

ATCAATTTTGTGGCCCAAAGGTAGTACAGACATCATTAGTTCAAAATGTC

TTCTGTCATAGCAGTGGCATATCATGTTGAGAAGTTATTGTGAGACATAC

CAACTTGAACTGTCATTCAATAGCAGGAAAGAATACTGTTATTTTTATTC

AATAGTATTTTTAATGTTAATCTTCAGTATTTTTATTATTATGTACTTAT

TGTGGTGCACTATTAAATTTATCAGCCCCTCAAACTAATGGATTAGTAGT

TAAACAAGCTTATTTACCAAAAACGATTAATCCTACGATAACTTGAAGAA

AGACTTAAGATATGCTTCCAGTATTGTTCAAAGTCAAAAGAATCAAGAGG

ACAGAAAGTGAAGCATAAAACATACAAATAGTAGGTGTATTAAACCCAAC

GCCAGCAATTTAGTTCCAGCAAAATATCATTTATCACACTGATGTTTTCT

ATATGAACTCTAGTGGCTTTTGTATGTTTGCTTGTTTTTAATTTGCAGCT

TTGCTTGAGTTTTGAAGTAATTTAAGAGCTATATGTATGGGAAATTTATA

TCTGTTCATTTTGAAGTAACACAATAAAAATAATGTAGAGCAACAGTA

>hg19_ct_ARAlincRNAs_9727_ARAlincRNA_0299.4 range=chr2:75158555-75170624 5'pad=0 3'pad=0 strand=+ repeatMasking=none

TTTAACCAAAAGAGCATGGGCAGATATTTCCCGGATCTGAACAGGAATAG

CTGGTGTCTGGACTCTATGAAGAATCTTCATGTGCAGTGATAGAAGAATA

AACACATTTGTAAAAAGACATCCCTTAACTATGGCAAAACAACAACAACA

ACGACGACAAAAAACAAAACAAAACAAAACAAAACCCTGCATCAATTTGA

TAAATAATCCTCCCCGTTGATGTCACTGGTTTTTTTCTGCACATCTTCTC

ACTGATATTTAAATGCTGAGCTTACACTTTCCATGGACATGCTTAATGAC

TAAACAATAGTTGAATTCTAAATTAATCAACTGACCAACCAGTAAGAATT

TATTGAGCAACTCCTTTGAAGCCGTCTATCTGTTAGGTGCTGTGGGAGCA

CAAAAGAAATAGGACATCATCTCTGCCTCAGGGAATTTAGTGTTTTGGGG

AAAACCTGAGTGGTATACACAAAGCAATTGAGGAACAACCTAGGACCTTG

TAGTCCATTAGGATTTCCAAAAATAGAAAGAGAATGGAAAGTCACCTGGC

TAGGAGAAGCCAGTGGAAACTTGACACGAAGAAAAAAGAGCAGCTAATTT

CATTCCTGTCCACCAGTTATTTATGTGTTTATCTTTAATTACATTTGTTT

GATTTCCCTTATTAAAGTCTGATGTCTTAAAAAAGCAGAAAAGTGAGGCA

GGTCAGCAGGGGATGTAAGTTGGGAAGAAAGACAGGTGAGGGCAAGAATT

TAGGCAGGAGCCACAGTGTTGGTTGTGCAGGTGAAGGTCAGGTGACGGAG

GGTAACCAGTCATGGATGACCCAGGCAGGAGCCATAACCAAAATGTTAGA

AAAAGTTGGTAAGAAATTGTTCCAGGCCAGGCGCAGTGGCTCATGCTTGT

AATTCCAGCACTTTGGGAGGCCAAGGTAGGCGGATCACTTGAGGTCAGGA

GTTTGAAACCAGCCTGGCCAACATGGAGATACCCTGTCTCTACTAAAAAT

ACAAAAACTATCTGGGCATGGTGGCACGTGCCTGTAATCCCAGCTACTCA

GGAGGCTGAGGCAGGAGAATCACTTCAACCCAGGAGACAGAGATTGCAGT

GAGCCGAGATTGCGCCACTGCACTCCAGCCTGGGTGACAGAGTGAGACAC

CATCTCAAAAAAAAAAAAAAAAAAAAGTTTTCCAGGAACAAGGGCAGAAC

TTAGCTACTGAGTTCTACCGAAGGCCAGGATCTGAGTCAAGCACCAAGGT

TAACTCCCAGGGAAGCCAAATGTTCATAAAAGGAAATCTTTCCAAATCAC

AAATATCAGGGCCTGCTAGGTAAATTCTAGCTTCTCTGTGCCAAGGGCTG

TGTTCCTACTCTCAAGCGCCACTCACTGACTGTCTGGGTGGTGCTGTGGG

CTTCTGTACTTTTAGCTTCATCAGCTGCACCTGTCCTCTTTTCCCATAAG

ACGCCAGGACCCTCAGAATCTTCCCTCTTACCAGAAATTTGGGTTTTGGG

CTGGGTCCCTGGACCGAATTCTGACACTCTAAGTGTTTGCAGACTATTTT

GATAGAGGTAGTGATTATGATGCCAGAAAAGTGAAGCCAGTTTTTATTAG

AATGTTGGCCATCTTGAATAATCCATTCCCCCACTTCCATGGGACATCTT

CAAAGAGATTTTTAAAAGACAAATATGATCTCTACTAATGAGATATCTTC

AGAAGGTAGAATTAAAGCAAAATTAATGTACACAGAATTCCTATTTCAAT

ATATTTATTATTCACAACATTTCTACACACACACACACACACACACACAC

GCATACACACACACACACACACATTCCTGCACAGGTAATTTATAGTGGCT

AATCCACTCAGTACTGTGGACTTTGGTTTTAGAGAGCATTGTTTTCAAGG

GTAGTACAGATAATCTGCCTCCAACATATTCTATTCTTAGGGCTCTAAAG

TCAAAGGAGTTTGTACAGACCCCTCCCTGCTAAGGTAATTATGTCCTCTT

CATATCTTGCATGAATGTTTGCTCTTAGAGTGATGATCTGAAATATCAAC

ATCCTGCAGGACCTAGTCTGGTCTGGAAGAAGAAGAGATATGTCATCTCT

ATGGGCTTTGATCTTGGTAGGCATATTTATTCACCAGGTACACAAATGCA

ACTTTAAACCCTCAGCCACATTTAGTAAACTATTTCTTCATCTGTTGCAG

TCTTATTCTGATGGGAAAAATTTCCTCTGTTTTTCAAGTTTAATTTATTA

TTTTTTCAAATTACAAGAGCAATGCATATTATTGTAACCAGAGAGATAAT

GCCAAGATCGGTAGAGAAAAACTTAATAATCTCGACCTCCTATAACCATC

TCTCTTCCCATCCCCCTGAGGTAATAGCAAATGTTAACAGCCTGCTACCT

CTGCACATCTTCCTTCCTTCATGATTTCCCTACACACACACCCATACACA

CACGTGCGCATGCACACACACACCCACTCAAACACACGGTGTACGTCCAG

GAGTCAGAAGTGGCTACAGAGATGGAGTCTTCATTATTAAGGTGAGAAAA

TGATAAGACAAATCACCCTTCTATTTCTACCTCCATGGAAAGCTAAATCT

TCCATTTATATTTTTATAGTCTTTTCAGCACTGTCTTAATGGTCTTGAGA

ACATATTACCCTGAAAATGTAAAAATAGAGCCAAAGAGGTGAAAGCTCCT

TGCGTGGCTTCTGACCAGGGATGGCTGCTTCCATGGGCTTTAGCTCTGCC

AGTTCTCCCTAGACTGGGGCAGGTAGGCAGTGAGGATTTCAGCATATATT

AAGTGCCAAATAGAAACAAATATTTGTTTCTATTATTAGAGTAAAACAAC

AGAAGCCTGTATTTTGACCAGAGGCCTGGAATCTACTACTTTGTGAATTA

GCTAAGCAGCCCCAGTTGAACGTGAACCAGCCTGGAACAGAGTGGACTGC

AGAGGCAATAAACAAGGATGTCGTGAAGACCCTGTTTCTTTCTAGCGTGG

CATCCCAGGATAAAATATACAATTAATAGATACTGGAAGGCACACTTTCT

GATTATGGAGAAACCATCAAATTTGGCCATGGTTTTGCATAGGGGCTAGG

TTGGAAATTCAACTAGAACAATTTAATGTCTACTGGCAAAGCTTCTTAAT

TATGGAATGAAAGCTACCTCAAAGTGCCTGGAGGCTTGAGCTGAATGGTG

AGATCTTCATTGACCTGACCTCCTCTGTGCTGAAGTTTGCTGACTTGAGG

AGACCTCCCCTAAGAACAGACCCTCCCCTCAGGGCTGCCTTCAGCGATGA

TGATTGACGCTGGCAAGTCAGTCAAGTGGGAAGTTTGCAGCACACACGGC

TCAAAGGACTCACCCAGTTTGCCCTGTTGGAGGATGTTAACCTTTGCACA

CTATTTTTGACATTTAATTTTTCCTTCCATTTAGTGCATGACTCTTTTTT

TGTCTTTCTAGTAAATTCATTTAGTTAGTATTATTCTTTGCCTATTTTGT

CTTTTAAACTTCTGGTTTTTAATCTTTCGTATTTTATATTATTTATTCTT

GGTGTTCATGCTTCTTATTCTCTTTTCTGCTATTTTATCTTATTGTTGTG

ATAATAGTATATTTGCAGTAAGCTCCTTTTACTAACTTCATTTATTTCTA

CATAATGTTTTATCTGGCTTTGACCACAACTATATAAGGGGACAGCAAAG

TGGAGCCACAAATGGGAAGATTGTGAAGAGGGCCCTTGACCAAATAAAGA

GGAAACCTTTTCCCTGAAGAGGACTTTTCAGCAACCCAACTAATTCACTC

ATTTTTCTCAAAGTTGAATTTTCTTCCACAAAAACCCCTTGACTTTGTTA

TTGAGTAGATCCTCAGGCCTAGGAAAATAATGTCTACATCGACCAAGGAG

ATATCTGAACTACCAGTACTTCCTGCAGCATCTTTGTTTTGGTTGGCCTG

GGCAGGTGGTGGGGCACGGGGGTGAAGGGAGGGGTCTGAGGTGGGTAGAA

AACAAGGGTATCCCCAGGGCTATGGGAGGAAGGTCTAGTGGACTGGAACC

ATTGTATCTGTCGATTAGTGGAGGCAATCATATTTGTGATCCTTGAGCTA

ATGGTCTAGTAAGACTATAAACTTGTTCATAATTAAAGCTGGCAAGAGGG

TTCTTACCCCAGGTAATGAGACAAGAGTGAACCACAGGAGGAAGCCAGCA

CAGAGACCGTATGTAATGAGTCCCTTCATGGGAAAGGGATGGAAAACAGT

CCTCAGGCTAGCAGCAGAGGGAGTGCTGTCTGTTGTGGGTCTTGACCTGC

TTAAGAGGTCTGACTTGAGGTGAGGCTGAAAGACAGCCCCGCTGGGGGCT

TCAAGATAACGATGGACGGATAGAGTTCTCTCACCGCTGAATATCTCCTA

AGGCATTAGTAAAACACTATTTAAATATTTCCTCTGTAACTCAGTTGTGC

TATTAAAGCTCTGTTTCTCAAAGTAGGTCTGTAGATGTTCTTGAGAGAGT

GAGTGTTTCATGAGAATTTTCAAATTTTCTGCTTTCGTTTTGAAGATAAT

CTTAAAGAAATAAAATCAAAATGTTCTACTACATTATTTTTAGTGCTGAA

GGCTGAGTTTGGTGCCAAATGAAGAACAAATAACATCACGGCATGCTATG

GAAATGTCGATTTTGCATTAAGTTTGAATAGAGAAAGATGTGGGAAAATT

GATCAATTTTGTGGCCCAAAGGTAGTACAGACATCATTAGTTCAAAATGT

CTTCTGTCATAGCAGTGGCATATCATGTTGAGAAGTTATTGTGAGACATA

CCAACTTGAACTGTCATTCAATAGCAGGAAAGAATACTGTTATTTTTATT

CAATAGTATTTTTAATGTTAATCTTCAGTATTTTTATTATTATGTACTTA

TTGTGGTGCACTATTAAATTTATCAGCCCCTCAAACTAATGGATTAGTAG

TTAAACAAGCTTATTTACCAAAAACGATTAATCCTACGATAACTTGAAGA

AAGACTTAAGATATGCTTCCAGTATTGTTCAAAGTCAAAAGAATCAAGAG

GACAGAAAGTGAAGCATAAAACATACAAATAGTAGGTGTATTAAACCCAA

CGCCAGCAATTTAGTTCCAGCAAAATATCATTTATCACACTGATGTTTTC

TATATGAACTCTAGTGGCTTTTGTATGTTTGCTTGTTTTTAATTTGCAGC

TTTGCTTGAGTTTTGAAGTAATTTAAGAGCTATATGTATGGGAAATTTAT

ATCTGTTCATTTTGAAGTAACACAATAAAAATAATGTAGAGCAACAGTA

>hg19_ct_ARAlincRNAs_9727_ARAlincRNA_0299.1 range=chr2:75159310-75165545 5'pad=0 3'pad=0 strand=+ repeatMasking=none

TTTCTTTGTGAAATAGGAACCAAGTTCACCATGGAGAGAGTGAGTGGGTT

GGGGAAAAGGTGTTGTATGTTTGAGGAGCGAGGAGAAGGTGTCAAATAAT

CATCCAGGCAGACTTTTGTAGTGCACAGTGACCGGTGACCAGCAGCATGT

GTTTCCATAGGCATCTTCTCTCAGTACCCCAGAAGGTGAATTGCCAGTGA

GTCCTGCCAGGATGCCCCTCGGTGACTGCACCACCCAGTAAAAAGACATC

CCTTAACTATGGCAAAACAACAACAACAACGACGACAAAAAACAAAACAA

AACAAAACAAAACCCTGCATCAATTTGATAAATAATCCTCCCCGTTGA

>hg19_ct_ARAlincRNAs_9727_ARAlincRNA_0299.2 range=chr2:75159310-75170624 5'pad=0 3'pad=0 strand=+ repeatMasking=none

TTTCTTTGTGAAATAGGAACCAAGTTCACCATGGAGAGAGTGAGTGGGTT

GGGGAAAAGGTGTTGTATGTTTGAGGAGCGAGGAGAAGGTGTCAAATAAT

CATCCAGGCAGACTTTTGTAGTGCACAGTGACCGGTGACCAGCAGCATGT

GTTTCCATAGGCATCTTCTCTCAGTACCCCAGAAGGTGAATTGCCAGTGA

GTCCTGCCAGGATGCCCCTCGGTGACTGCACCACCCAGTAAGTCATGGCT

GTGTACTTCCCATCAAGGTCTGGATCTCAGCTCAGGTAGTGGGGGCTGCT

CTTCCATAGTCATTCCATCCTTGTGTGCTCTGCCTCAGCCCTGGGGATAG

TGGCCGCTTTCTGTACATGTCAGTCCTCTATTCTTTAGACTTCTTGTTAC

CCCATTAGCAGCCAATTCTCTTTTGTTACTGCTTAGCAGCCAACTTTTTT

TATATTAAACTTTCCCCAACCAAATTATAGTGTGGTTTCTGTGTCTTAAT

TGGACCCCGGGTGATACAGTTTGCGTTTTGAAAGTGAAGAATTGGTAAAA

AGACATCCCTTAACTATGGCAAAACAACAACAACAACGACGACAAAAAAC

AAAACAAAACAAAACAAAACCCTGCATCAATTTGATAAATAATCCTCCCC

GTTGATGTCACTGGTTTTTTTCTGCACATCTTCTCACTGATATTTAAATG

CTGAGCTTACACTTTCCATGGACATGCTTAATGACTAAACAATAGTTGAA

TTCTAAATTAATCAACTGACCAACCAGTAAGAATTTATTGAGCAACTCCT

TTGAAGCCGTCTATCTGTTAGGTGCTGTGGGAGCACAAAAGAAATAGGAC

ATCATCTCTGCCTCAGGGAATTTAGTGTTTTGGGGAAAACCTGAGTGGTA

TACACAAAGCAATTGAGGAACAACCTAGGACCTTGTAGTCCATTAGGATT

TCCAAAAATAGAAAGAGAATGGAAAGTCACCTGGCTAGGAGAAGCCAGTG

GAAACTTGACACGAAGAAAAAAGAGCAGCTAATTTCATTCCTGTCCACCA

GTTATTTATGTGTTTATCTTTAATTACATTTGTTTGATTTCCCTTATTAA

AGTCTGATGTCTTAAAAAAGCAGAAAAGTGAGGCAGGTCAGCAGGGGATG

TAAGTTGGGAAGAAAGACAGGTGAGGGCAAGAATTTAGGCAGGAGCCACA

GTGTTGGTTGTGCAGGTGAAGGTCAGGTGACGGAGGGTAACCAGTCATGG

ATGACCCAGGCAGGAGCCATAACCAAAATGTTAGAAAAAGTTGGTAAGAA

ATTGTTCCAGGCCAGGCGCAGTGGCTCATGCTTGTAATTCCAGCACTTTG

GGAGGCCAAGGTAGGCGGATCACTTGAGGTCAGGAGTTTGAAACCAGCCT

GGCCAACATGGAGATACCCTGTCTCTACTAAAAATACAAAAACTATCTGG

GCATGGTGGCACGTGCCTGTAATCCCAGCTACTCAGGAGGCTGAGGCAGG

AGAATCACTTCAACCCAGGAGACAGAGATTGCAGTGAGCCGAGATTGCGC

CACTGCACTCCAGCCTGGGTGACAGAGTGAGACACCATCTCAAAAAAAAA

AAAAAAAAAAAGTTTTCCAGGAACAAGGGCAGAACTTAGCTACTGAGTTC

TACCGAAGGCCAGGATCTGAGTCAAGCACCAAGGTTAACTCCCAGGGAAG

CCAAATGTTCATAAAAGGAAATCTTTCCAAATCACAAATATCAGGGCCTG

CTAGGTAAATTCTAGCTTCTCTGTGCCAAGGGCTGTGTTCCTACTCTCAA

GCGCCACTCACTGACTGTCTGGGTGGTGCTGTGGGCTTCTGTACTTTTAG

CTTCATCAGCTGCACCTGTCCTCTTTTCCCATAAGACGCCAGGACCCTCA

GAATCTTCCCTCTTACCAGAAATTTGGGTTTTGGGCTGGGTCCCTGGACC

GAATTCTGACACTCTAAGTGTTTGCAGACTATTTTGATAGAGGTAGTGAT

TATGATGCCAGAAAAGTGAAGCCAGTTTTTATTAGAATGTTGGCCATCTT

GAATAATCCATTCCCCCACTTCCATGGGACATCTTCAAAGAGATTTTTAA

AAGACAAATATGATCTCTACTAATGAGATATCTTCAGAAGGTAGAATTAA

AGCAAAATTAATGTACACAGAATTCCTATTTCAATATATTTATTATTCAC

AACATTTCTACACACACACACACACACACACACACGCATACACACACACA

CACACACATTCCTGCACAGGTAATTTATAGTGGCTAATCCACTCAGTACT

GTGGACTTTGGTTTTAGAGAGCATTGTTTTCAAGGGTAGTACAGATAATC

TGCCTCCAACATATTCTATTCTTAGGGCTCTAAAGTCAAAGGAGTTTGTA

CAGACCCCTCCCTGCTAAGGTAATTATGTCCTCTTCATATCTTGCATGAA

TGTTTGCTCTTAGAGTGATGATCTGAAATATCAACATCCTGCAGGACCTA

GTCTGGTCTGGAAGAAGAAGAGATATGTCATCTCTATGGGCTTTGATCTT

GGTAGGCATATTTATTCACCAGGTACACAAATGCAACTTTAAACCCTCAG

CCACATTTAGTAAACTATTTCTTCATCTGTTGCAGTCTTATTCTGATGGG

AAAAATTTCCTCTGTTTTTCAAGTTTAATTTATTATTTTTTCAAATTACA

AGAGCAATGCATATTATTGTAACCAGAGAGATAATGCCAAGATCGGTAGA

GAAAAACTTAATAATCTCGACCTCCTATAACCATCTCTCTTCCCATCCCC

CTGAGGTAATAGCAAATGTTAACAGCCTGCTACCTCTGCACATCTTCCTT

CCTTCATGATTTCCCTACACACACACCCATACACACACGTGCGCATGCAC

ACACACACCCACTCAAACACACGGTGTACGTCCAGGAGTCAGAAGTGGCT

ACAGAGATGGAGTCTTCATTATTAAGGGGACAGCAAAGTGGAGCCACAAA

TGGGAAGATTGTGAAGAGGGCCCTTGACCAAATAAAGAGGAAACCTTTTC

CCTGAAGAGGACTTTTCAGCAACCCAACTAATTCACTCATTTTTCTCAAA

GTTGAATTTTCTTCCACAAAAACCCCTTGACTTTGTTATTGAGTAGATCC

TCAGGCCTAGGAAAATAATGTCTACATCGACCAAGGAGATATCTGAACTA

CCAGTACTTCCTGCAGCATCTTTGTTTTGGTTGGCCTGGGCAGGTGGTGG

GGCACGGGGGTGAAGGGAGGGGTCTGAGGTGGGTAGAAAACAAGGGTATC

CCCAGGGCTATGGGAGGAAGGTCTAGTGGACTGGAACCATTGTATCTGTC

GATTAGTGGAGGCAATCATATTTGTGATCCTTGAGCTAATGGTCTAGTAA

GACTATAAACTTGTTCATAATTAAAGCTGGCAAGAGGGTTCTTACCCCAG

GTAATGAGACAAGAGTGAACCACAGGAGGAAGCCAGCACAGAGACCGTAT

GTAATGAGTCCCTTCATGGGAAAGGGATGGAAAACAGTCCTCAGGCTAGC

AGCAGAGGGAGTGCTGTCTGTTGTGGGTCTTGACCTGCTTAAGAGGTCTG

ACTTGAGGTGAGGCTGAAAGACAGCCCCGCTGGGGGCTTCAAGATAACGA

TGGACGGATAGAGTTCTCTCACCGCTGAATATCTCCTAAGGCATTAGTAA

AACACTATTTAAATATTTCCTCTGTAACTCAGTTGTGCTATTAAAGCTCT

GTTTCTCAAAGTAGGTCTGTAGATGTTCTTGAGAGAGTGAGTGTTTCATG

AGAATTTTCAAATTTTCTGCTTTCGTTTTGAAGATAATCTTAAAGAAATA

AAATCAAAATGTTCTACTACATTATTTTTAGTGCTGAAGGCTGAGTTTGG

TGCCAAATGAAGAACAAATAACATCACGGCATGCTATGGAAATGTCGATT

TTGCATTAAGTTTGAATAGAGAAAGATGTGGGAAAATTGATCAATTTTGT

GGCCCAAAGGTAGTACAGACATCATTAGTTCAAAATGTCTTCTGTCATAG

CAGTGGCATATCATGTTGAGAAGTTATTGTGAGACATACCAACTTGAACT

GTCATTCAATAGCAGGAAAGAATACTGTTATTTTTATTCAATAGTATTTT

TAATGTTAATCTTCAGTATTTTTATTATTATGTACTTATTGTGGTGCACT

ATTAAATTTATCAGCCCCTCAAACTAATGGATTAGTAGTTAAACAAGCTT

ATTTACCAAAAACGATTAATCCTACGATAACTTGAAGAAAGACTTAAGAT

ATGCTTCCAGTATTGTTCAAAGTCAAAAGAATCAAGAGGACAGAAAGTGA

AGCATAAAACATACAAATAGTAGGTGTATTAAACCCAACGCCAGCAATTT

AGTTCCAGCAAAATATCATTTATCACACTGATGTTTTCTATATGAACTCT

AGTGGCTTTTGTATGTTTGCTTGTTTTTAATTTGCAGCTTTGCTTGAGTT

TTGAAGTAATTTAAGAGCTATATGTATGGGAAATTTATATCTGTTCATTT

TGAAGTAACACAATAAAAATAATGTAGAGCAACAGTA

>hg19_ct_ARAlincRNAs_9727_ARAlincRNA_0299.12 range=chr2:75159788-75167866 5'pad=0 3'pad=0 strand=+ repeatMasking=none

GCAGACTTTTGTAGTGCACAGTGACCGGTGACCAGCAGCATGTGTTTCCA

TAGGCATCTTCTCTCAGTACCCCAGAAGGTGAATTGCCAGTGAGTCCTGC

CAGGATGCCCCTCGGTGACTGCACCACCCAGTAAAAAGACATCCCTTAAC

TATGGCAAAACAACAACAACAACGACGACAAAAAACAAAACAAAACAAAA

CAAAACCCTGCATCAATTTGATAAATAATCCTCCCCGTTGATGTCACTGG

TTTTTTTCTGCACATCTTCTCACTGATATTTAAATGCTGAGCTTACACTT

TCCATGGACATGCTTAATGACTAAACAATAGTTGAATTCTAAATTAATCA

ACTGACCAACCAGTAAGAATTTATTGAGCAACTCCTTTGAAGCCGTCTAT

CTGTTAGGTGCTGTGGGAGCACAAAAGAAATAGGACATCATCTCTGCCTC

AGGGAATTTAGTGTTTTGGGGAAAACCTGAGTGGTATACACAAAGCAATT

GAGGAACAACCTAGGACCTTGTAGTCCATTAGGATTTCCAAAAATAGAAA

GAGAATGGAAAGTCACCTGGCTAGGAGAAGCCAGTGGAAACTTGACACGA

AGAAAAAAGAGCAGCTAATTTCATTCCTGTCCACCAGTTATTTATGTGTT

TATCTTTAATTACATTTGTTTGATTTCCCTTATTAAAGTCTGATGTCTTA

AAAAAGCAGAAAAGTGAGGCAGGTCAGCAGGGGATGTAAGTTGGGAAGAA

AGACAGGTGAGGGCAAGAATTTAGGCAGGAGCCACAGTGTTGGTTGTGCA

GGTGAAGGTCAGGTGACGGAGGGTAACCAGTCATGGATGACCCAGGCAGG

AGCCATAACCAAAATGTTAGAAAAAGTTGGTAAGAAATTGTTCCAGGCCA

GGCGCAGTGGCTCATGCTTGTAATTCCAGCACTTTGGGAGGCCAAGGTAG

GCGGATCACTTGAGGTCAGGAGTTTGAAACCAGCCTGGCCAACATGGAGA

TACCCTGTCTCTACTAAAAATACAAAAACTATCTGGGCATGGTGGCACGT

GCCTGTAATCCCAGCTACTCAGGAGGCTGAGGCAGGAGAATCACTTCAAC

CCAGGAGACAGAGATTGCAGTGAGCCGAGATTGCGCCACTGCACTCCAGC

CTGGGTGACAGAGTGAGACACCATCTCAAAAAAAAAAAAAAAAAAAAGTT

TTCCAGGAACAAGGGCAGAACTTAGCTACTGAGTTCTACCGAAGGCCAGG

ATCTGAGTCAAGCACCAAGGTTAACTCCCAGGGAAGCCAAATGTTCATAA

AAGGAAATCTTTCCAAATCACAAATATCAGGGCCTGCTAGGTAAATTCTA

GCTTCTCTGTGCCAAGGGCTGTGTTCCTACTCTCAAGCGCCACTCACTGA

CTGTCTGGGTGGTGCTGTGGGCTTCTGTACTTTTAGCTTCATCAGCTGCA

CCTGTCCTCTTTTCCCATAAGACGCCAGGACCCTCAGAATCTTCCCTCTT

ACCAGAAATTTGGGTTTTGGGCTGGGTCCCTGGACCGAATTCTGACACTC

TAAGTGTTTGCAGACTATTTTGATAGAGGTAGTGATTATGATGCCAGAAA

AGTGAAGCCAGTTTTTATTAGAATGTTGGCCATCTTGAATAATCCATTCC

CCCACTTCCATGGGACATCTTCAAAGAGATTTTTAAAAGACAAATATGAT

CTCTACTAATGAGATATCTTCAGAAGGTAGAATTAAAGCAAAATTAATGT

ACACAGAATTCCTATTTCAATATATTTATTATTCACAACATTTCTACACA

CACACACACACACACACACACGCATACACACACACACACACACATTCCTG

CACAGGTAATTTATAGTGGCTAATCCACTCAGTACTGTGGACTTTGGTTT

TAGAGAGCATTGTTTTCAAGGGTAGTACAGATAATCTGCCTCCAACATAT

TCTATTCTTAGGGCTCTAAAGTCAAAGGAGTTTGTACAGACCCCTCCCTG

CTAAGGTAATTATGTCCTCTTCATATCTTGCATGAATGTTTGCTCTTAGA

GTGATGATCTGAAATATCAACATCCTGCAGGACCTAGTCTGGTCTGGAAG

AAGAAGAGATATGTCATCTCTATGGGCTTTGATCTTGGTAGGCATATTTA

TTCACCAGGTACACAAATGCAACTTTAAACCCTCAGCCACATTTAGTAAA

CTATTTCTTCATCTGTTGCAGTCTTATTCTGATGGGAAAAATTTCCTCTG

TTTTTCAAGTTTAATTTATTATTTTTTCAAATTACAAGAGCAATGCATAT

TATTGTAACCAGAGAGATAATGCCAAGATCGGTAGAGAAAAACTTAATAA

TCTCGACCTCCTATAACCATCTCTCTTCCCATCCCCCTGAGGTAATAGCA

AATGTTAACAGCCTGCTACCTCTGCACATCTTCCTTCCTTCATGATTTCC

CTACACACACACCCATACACACACGTGCGCATGCACACACACACCCACTC

AAACACACGGTGTACGTCCAGGAGTCAGAAGTGGCTACAGAGATGGAGTC

TTCATTATTAAG

>hg19_ct_ARAlincRNAs_9727_ARAlincRNA_0299.3 range=chr2:75161163-75170624 5'pad=0 3'pad=0 strand=+ repeatMasking=none

ATGACATTTGTTATCTCACAGCTCTGGAAGCTAGACTTCTGAAATCAAGT

TGTTGGCAGGATTGGTTCCTTCTGAGGGCAGTGAGGGAAGGATCCATTCC

AGGCTTCTCTCCTTGGCTTATTGGTAAAAAGACATCCCTTAACTATGGCA

AAACAACAACAACAACGACGACAAAAAACAAAACAAAACAAAACAAAACC

CTGCATCAATTTGATAAATAATCCTCCCCGTTGATGTCACTGGTTTTCCA

TCTCAAAAAAAAAAAAAAAAAAAAGTTTTCCAGGAACAAGGGCAGAACTT

AGCTACTGAGTTCTACCGAAGGCCAGGATCTGAGTCAAGCACCAAGGTTA

ACTCCCAGGGAAGCCAAATGTTCATAAAAGGAAATCTTTCCAAATCACAA

ATATCAGGGCCTGCTAGGTAAATTCTAGCTTCTCTGTGCCAAGGGCTGTG

TTCCTACTCTCAAGCGCCACTCACTGACTGTCTGGGTGGTGCTGTGGGCT

TCTGTACTTTTAGCTTCATCAGCTGCACCTGTCCTCTTTTCCCATAAGAC

GCCAGGACCCTCAGAATCTTCCCTCTTACCAGAAATTTGGGTTTTGGGCT

GGGTCCCTGGACCGAATTCTGACACTCTAAGTGTTTGCAGACTATTTTGA

TAGAGGTAGTGATTATGATGCCAGAAAAGTGAAGCCAGTTTTTATTAGAA

TGTTGGCCATCTTGAATAATCCATTCCCCCACTTCCATGGGACATCTTCA

AAGAGATTTTTAAAAGACAAATATGATCTCTACTAATGAGATATCTTCAG

AAGGTAGAATTAAAGCAAAATTAATGTACACAGAATTCCTATTTCAATAT

ATTTATTATTCACAACATTTCTACACACACACACACACACACACACACGC

ATACACACACACACACACACATTCCTGCACAGGTAATTTATAGTGGCTAA

TCCACTCAGTACTGTGGACTTTGGTTTTAGAGAGCATTGTTTTCAAGGGT

AGTACAGATAATCTGCCTCCAACATATTCTATTCTTAGGGCTCTAAAGTC

AAAGGAGTTTGTACAGACCCCTCCCTGCTAAGGTAATTATGTCCTCTTCA

TATCTTGCATGAATGTTTGCTCTTAGAGTGATGATCTGAAATATCAACAT

CCTGCAGGACCTAGTCTGGTCTGGAAGAAGAAGAGATATGTCATCTCTAT

GGGCTTTGATCTTGGTAGGCATATTTATTCACCAGGTACACAAATGCAAC

TTTAAACCCTCAGCCACATTTAGTAAACTATTTCTTCATCTGTTGCAGTC

TTATTCTGATGGGAAAAATTTCCTCTGTTTTTCAAGTTTAATTTATTATT

TTTTCAAATTACAAGAGCAATGCATATTATTGTAACCAGAGAGATAATGC

CAAGATCGGTAGAGAAAAACTTAATAATCTCGACCTCCTATAACCATCTC

TCTTCCCATCCCCCTGAGGTAATAGCAAATGTTAACAGCCTGCTACCTCT

GCACATCTTCCTTCCTTCATGATTTCCCTACACACACACCCATACACACA

CGTGCGCATGCACACACACACCCACTCAAACACACGGTGTACGTCCAGGA

GTCAGAAGTGGCTACAGAGATGGAGTCTTCATTATTAAGGTGAGAAAATG

ATAAGACAAATCACCCTTCTATTTCTACCTCCATGGAAAGCTAAATCTTC

CATTTATATTTTTATAGTCTTTTCAGCACTGTCTTAATGGTCTTGAGAAC

ATATTACCCTGAAAATGTAAAAATAGAGCCAAAGAGGTGAAAGCTCCTTG

CGTGGCTTCTGACCAGGGATGGCTGCTTCCATGGGCTTTAGCTCTGCCAG

TTCTCCCTAGACTGGGGCAGGTAGGCAGTGAGGATTTCAGCATATATTAA

GTGCCAAATAGAAACAAATATTTGTTTCTATTATTAGAGTAAAACAACAG

AAGCCTGTATTTTGACCAGAGGCCTGGAATCTACTACTTTGTGAATTAGC

TAAGCAGCCCCAGTTGAACGTGAACCAGCCTGGAACAGAGTGGACTGCAG

AGGCAATAAACAAGGATGTCGTGAAGACCCTGTTTCTTTCTAGCGTGGCA

TCCCAGGATAAAATATACAATTAATAGATACTGGAAGGCACACTTTCTGA

TTATGGAGAAACCATCAAATTTGGCCATGGTTTTGCATAGGGGCTAGGTT

GGAAATTCAACTAGAACAATTTAATGTCTACTGGCAAAGCTTCTTAATTA

TGGAATGAAAGCTACCTCAAAGTGCCTGGAGGCTTGAGCTGAATGGTGAG

ATCTTCATTGACCTGACCTCCTCTGTGCTGAAGTTTGCTGACTTGAGGAG

ACCTCCCCTAAGAACAGACCCTCCCCTCAGGGCTGCCTTCAGCGATGATG

ATTGACGCTGGCAAGTCAGTCAAGTGGGAAGTTTGCAGCACACACGGCTC

AAAGGACTCACCCAGTTTGCCCTGTTGGAGGATGTTAACCTTTGCACACT

ATTTTTGACATTTAATTTTTCCTTCCATTTAGTGCATGACTCTTTTTTTG

TCTTTCTAGTAAATTCATTTAGTTAGTATTATTCTTTGCCTATTTTGTCT

TTTAAACTTCTGGTTTTTAATCTTTCGTATTTTATATTATTTATTCTTGG

TGTTCATGCTTCTTATTCTCTTTTCTGCTATTTTATCTTATTGTTGTGAT

AATAGTATATTTGCAGTAAGCTCCTTTTACTAACTTCATTTATTTCTACA

TAATGTTTTATCTGGCTTTGACCACAACTATATAAGGGGACAGCAAAGTG

GAGCCACAAATGGGAAGATTGTGAAGAGGGCCCTTGACCAAATAAAGAGG

AAACCTTTTCCCTGAAGAGGACTTTTCAGCAACCCAACTAATTCACTCAT

TTTTCTCAAAGTTGAATTTTCTTCCACAAAAACCCCTTGACTTTGTTATT

GAGTAGATCCTCAGGCCTAGGAAAATAATGTCTACATCGACCAAGGAGAT

ATCTGAACTACCAGTACTTCCTGCAGCATCTTTGTTTTGGTTGGCCTGGG

CAGGTGGTGGGGCACGGGGGTGAAGGGAGGGGTCTGAGGTGGGTAGAAAA

CAAGGGTATCCCCAGGGCTATGGGAGGAAGGTCTAGTGGACTGGAACCAT

TGTATCTGTCGATTAGTGGAGGCAATCATATTTGTGATCCTTGAGCTAAT

GGTCTAGTAAGACTATAAACTTGTTCATAATTAAAGCTGGCAAGAGGGTT

CTTACCCCAGGTAATGAGACAAGAGTGAACCACAGGAGGAAGCCAGCACA

GAGACCGTATGTAATGAGTCCCTTCATGGGAAAGGGATGGAAAACAGTCC

TCAGGCTAGCAGCAGAGGGAGTGCTGTCTGTTGTGGGTCTTGACCTGCTT

AAGAGGTCTGACTTGAGGTGAGGCTGAAAGACAGCCCCGCTGGGGGCTTC

AAGATAACGATGGACGGATAGAGTTCTCTCACCGCTGAATATCTCCTAAG

GCATTAGTAAAACACTATTTAAATATTTCCTCTGTAACTCAGTTGTGCTA

TTAAAGCTCTGTTTCTCAAAGTAGGTCTGTAGATGTTCTTGAGAGAGTGA

GTGTTTCATGAGAATTTTCAAATTTTCTGCTTTCGTTTTGAAGATAATCT

TAAAGAAATAAAATCAAAATGTTCTACTACATTATTTTTAGTGCTGAAGG

CTGAGTTTGGTGCCAAATGAAGAACAAATAACATCACGGCATGCTATGGA

AATGTCGATTTTGCATTAAGTTTGAATAGAGAAAGATGTGGGAAAATTGA

TCAATTTTGTGGCCCAAAGGTAGTACAGACATCATTAGTTCAAAATGTCT

TCTGTCATAGCAGTGGCATATCATGTTGAGAAGTTATTGTGAGACATACC

AACTTGAACTGTCATTCAATAGCAGGAAAGAATACTGTTATTTTTATTCA

ATAGTATTTTTAATGTTAATCTTCAGTATTTTTATTATTATGTACTTATT

GTGGTGCACTATTAAATTTATCAGCCCCTCAAACTAATGGATTAGTAGTT

AAACAAGCTTATTTACCAAAAACGATTAATCCTACGATAACTTGAAGAAA

GACTTAAGATATGCTTCCAGTATTGTTCAAAGTCAAAAGAATCAAGAGGA

CAGAAAGTGAAGCATAAAACATACAAATAGTAGGTGTATTAAACCCAACG

CCAGCAATTTAGTTCCAGCAAAATATCATTTATCACACTGATGTTTTCTA

TATGAACTCTAGTGGCTTTTGTATGTTTGCTTGTTTTTAATTTGCAGCTT

TGCTTGAGTTTTGAAGTAATTTAAGAGCTATATGTATGGGAAATTTATAT

CTGTTCATTTTGAAGTAACACAATAAAAATAATGTAGAGCAACAGTA

>hg19_ct_ARAlincRNAs_9727_ARAlincRNA_0300.1 range=chr2:88316306-88320971 5'pad=0 3'pad=0 strand=+ repeatMasking=none

AGGCGTGGCCACCAAGGGGCCGGGCCCTGCACAGCGCCCTGGGGAAGCTG

GTTTGAAAGCTCTGGAGCGACCCCAATGCCGGGTAAAGGACAAGATTTGC

CCAGACGGAAGGGAATTTGGGGTGAAGTGGCCGTAAAAGTGTGGGACGAA

ACTGTTTCGGGCAGAGCGGTGGGAACTCACCTGAGGAAAGCATGGGGGAG

GAGTCACTAATAAGAATGAGTGGCAGGGACAGGGCGGCCTTGTAACGCCC

TCGGTGTTGGAAGGGAAGACCGTACAGCGCGATGGGGACCAGGGCGGCGG

GCCGCAGCCAGGGTGTGGGTGGGCGACGGTCAAGGTGCCTTGAAGCGGAT

TCTGTCCTCGGGAGCTGCGGTTCTTCCTCCTCCGCCCTCACCGGCGGGCG

ACCTTAGGCAGGGCGTGAAATGCAACGGGAGCCCTGGAGGGAGGGTGAAG

CCGCGCGTGCAGGAGGAACCTCCTTTTGGCGAGTTCAGGCCCCGCAGCTG

CGGAGGTGGGGGACCCTGTGTGGCAGCCTGCTGAGAGGCTGCGTGCTCCG

CGCGGTTGGAGGGGGGAACCTCCAGCCCACCTCCTCTTTTTCAGCCGAGA

AACCGCTAGTTACCCCCTTTCTTATGCGACCAAGATTGTAATAACCCTGT

CACCTTTCACGGCCCTCCGGGTCTGCGCCCATCCTCCTCTGAGGATTCGC

TCATCCACCCCGAGGCCTGTTTTCCTCACTTGTTGTCCCTCTGGCAGACC

CTAGGCCCCTTGGAGTCCAGCCACCATCTATCTGCCCGCTGTTTCCACCC

ACCACACTCCCTCCCCAGTTTGAAAGTTCAAAACTGTCTTACAGCTACTG

CCTGGGGTCTAGGAATCCAGAACATTCCACACATTGCCTAAGTCAACAAC

CTGTTGCACACACACACCTGCAACTGTTTTGCAAAAGTATGTGGATCTGG

TTGTCATTGATAAAATGCAAATTAATTTAGATGTATTATTCAATTCATAG

TATAATTTTGTTGTATTTTCAAATCAACAGATATTTACGTAATTTCGAAG

CAGCTCTTCACAAAATATTCATCAGTTACAGAAGGAAGTCACTTCACAGT

GGAAAAATCTGGCAGGTGCCACCTTAATCAAGTAACCAAAATGAACATCA

TCAGTAATAGGACAAATTGCAATCGTGTGCCTCCTGATAGTCTAGCAAGA

AGAACACAGCATCACTTCTATGATGCTCCTGCCAATAATATATAATTGGA

ATCTAATCCTAAAGCAACAAATTCAAATTAAGGAAAATTCTACAAAATAA

TTGGCCTGTAATAGAAGTGTTAAAGGTCATGAAAGTTAAGGAAAGACTGA

AGAACTGTTCCAGACTGAAGATGAAAGAGATGTGAGAAATAAGTGCAGTG

GTGATTCTGAACTGGATCCTGACTTGGCAAAACTTGAATAGGTCTGTTGG

TTGGATGGTAGAAATATAGTAATGTTGATTTCCTGATTTTTGCTAGCTAT

ATTACAATTTTATTGGATTCAGTCCTTGTTTACAGAAGACACACAGTATG

TAGGGGTTATAAGAAGTCAGCTCAGGAACTTTCAAATCATTTAGCAAAAC

AAAAGTTCTTCGTACTTTAATTCCAAGACTTCTATAAATTTGAGATTATT

TTTAAAATACTTAAAAAAAACTCCTTTAAAGTCTCTCAACATGCTGTCCT

ATTAATAATAGATTTCTAAATTTTTTATTTTTATCAACGTTTTGTGTGCC

CATAGTTTGAAGTATTAAATTGTTTTTATTTATTAAG

>hg19_ct_ARAlincRNAs_9727_ARAlincRNA_0301.1 range=chr2:88487627-88488450 5'pad=0 3'pad=0 strand=- repeatMasking=none

CTCACTTCATCTGGTACTAAGATCCTGGCTTTTTCCATTAAGTGGTGGGC

GTGTGTGTGTGTGTGTGTGTGTGGGTGTGTGTGTGTATTAGTCTGCTCAG

GACTGGGTGAGATTTAAATAATAGAAATTTATTTCCTCCCAGTTCTAGAG

GATGGAGGTCTCAGATCGAGGTGCCCTCAGCGTTGCTTTAATTCTGGGGC

CCCTCTCCTTGGCCTGCAGATAGCAGCCTTCTTCCTCTGACTTCACGCAG

TCTTCCCTCTGTGCCACATGTCTATGTCTTAATTTCCTCTTATAAAATAA

GGGACACCAGTCCTACTGGTTTAGGGCCCAGCATAAAGACTTCATTTTAA

TTACCTTTTTAAAGACCCTATCTCCAAATACAGTTACATTCAGAGATACT

GTGGATTCAACATATAATGGAATTGACACTATTCAGCTCGCGTGGTGTGT

GTGTGTGTTACAGGGGTGGGCACTTCTCCAGACTTCATCTCGTTCTCATC

CTTTTTCTCATTTCCCCAAGACTCCCTGTGGCCAGTGTAGCCTCAGTTCA

GGTTTCCTGTCGATGTGCATTGTGCCTGGTGTGGCCTTATTCTGGCCTCC

CTCACAGGCATGGTGGCCTCTCAGAGCTGGATAGGTCTCAGAACCATTCA

GGGGATGGGGTTTCCCTGTGACCTTGGCCCTGCCCTGAGGTCCTCAGAAT

ATGGCAGCAAAAGAGAAGGAGCCACCTGCACGGGCCTGCCCACAGCAGCG

CCCAGTGCCAGTTAAAGAGGCCCGGAAAACAGGGCTGCAGCAGCCCCTGG

AGGACTCTTGGAGAAGCACAGCCA

>hg19_ct_ARAlincRNAs_9727_ARAlincRNA_0301.2 range=chr2:88488430-88489157 5'pad=0 3'pad=0 strand=- repeatMasking=none

GCAAGATAAGACTAATTCACAAAGCAATTAGTAAAAAATACAAGATAGCT

TAAAAGTACTAAATTGTTCAGTGCAGACTATAATTGTGTTAATAGTAAGA

ACCATTTGTATGGCATTTGAGAGTTTACCCAGCACTTGGAAATACATTAT

CTCATCTCATGTAATCCTCAAAGCAAACCAGCAAGGTAAGTACAGATGTT

CCTCAACTTACAATAGTTACATCCCAATAAACCCTTCGTAAGTTGAAAAT

ATCCTAAGTTGAAAATGGGTAATACCCTCAACCTACAGAAACATCATCAT

TTGGCCTAGCCTAAATGTGCTCAGAACACTGACATTAGCCTACAGTTGGG

CAAAGTCATCTGTCAATACAGCACACTGTGGAATATGGGCTATTTATCCT

CGTGATCACAGGGCTGACCGGGAGCTGGGCTCACTGCTGCTGCCCTGCGT

TACAAGAGACTATCATACTGCATATCGTTAGCCTGGGGAAAAATCAAAAT

CTGAAGTCTACTGAATGCATACGAACTCAGGTCTACCGGATGCATATTGC

TTTCGCACCATCACCATCCTAAAGTTGCAAAGTCTTAAGTCAGGGATCAT

CTGTATTGTTCGATGCAGATGAGGAAACCACGGCTCACAGAGATTAAATG

GCTTAGTTGTGATCACTACCAGTAAGTGGGTGACTCAGAAAAGACTCCAC

CCTGATTCTCACTTCATCTGGTACTAAG

>hg19_ct_ARAlincRNAs_9727_ARAlincRNA_0302.1 range=chr2:95885833-95889194 5'pad=0 3'pad=0 strand=+ repeatMasking=none

GCAATGTGTGGGGAGGGGTGGGTTTTAGAAATCTGTCTGCTCAGTTGGTC

TTCTCTGATACCACCCCAGGGTAAAGGTGGAGGACTGAGGAGCCTTGTTA

CAGCCTGGTGAGGGTGGAAGTATAGGCGTTTGTTGGCGGGGATAGGACCA

TAGCTTTCTTCTGTGGTCTTTGAAGTAGGATTGTGGTTTTCTAAACATTT

TCTGTCTTGCTAGGCTGCTCCCTTCCCAGTCCTTTGGCTAAAAAAAGCAG

ATTTTTCTTGGGGCTTTTTTTTTTTTTTTGGTCTGTGCCTGTTGGCGTTT

CCGGGTTGCCAGCTTCTCTAGAACCCGATCTGGGATAGATGAGGCAATAA

GAAAGCCCAAGAAAGTCACTGCTGTGTTCTTCCTCAGGTTCTGAGGTCCC

TGGCCAGTCCATTCTCTCTCCACCTTTTAGAACTTTCTTATGTTTGCTTT

GTATATAATATCCCGGATTTTTAGTTGTAGTTAGTGAGAGCGATAGGAGA

AAGTAGTGCTCCACCTTTCCCGTGACATCCTGGTGCTTGACACTGAAATC

ACACATCAAGTTGTGGTATATATAGGGTTTGTGGTATATAACCTAAGGGT

TTGTGGTATATACAGGTCATTTGTCCTGAAGATAAGCTGCCAATTTGATA

GGTAGAAACTATCAGCCTGCTTCATCTAGCTTTGAAGGAACAATAGTTTA

TAAATTGAGGAAACACCAGTATTTAATTGTAGGTTATAAACTGGAGAAGA

ATAGCTATGACTAAGAATATAAACTATAGGGTTACATTTTTTTATTTGTA

TTTTTGTTTGAAAAACATATGAGAAGCACTGGAGAACTGGACTCAGAATT

TTGGATTTTTTTACAATTATATATTATGTACATGTGGCTATCTGTTCACT

TTAATTTGCCATATGACATATGATATCATAGAGAATCAACAAAATAAAGA

ATGAAAATAACTGAGATTTGGGACAAGTCACCTTCATCCTTTGAAATCTG

TTTCCTCATAGATATATGTTTTAAAATTTAATGCCTACTTGAGAATGATT

GCAAGAGTTAGGTCGAAAATGGATGCAAATGTGGTTTGTAAGCTATCAGG

TGCTACGTAACTATTAGGTATTATTCATATAGTCATTTAGAAGACATATC

CATCATGTATGCTTCACAGTGGTGTAGCCCAATAAAAACAACTGTGAATA

TAATGGAATGAGGCTTTTTATCTTTTTAAGTAAGTTTTCTGGGATCCTGT

TAAAATCTGCATCTTTTGAGATAATCATATGGTTCCTTTTAATCTATTAA

TGTGGTAAAGTACATTGATTTTCTGATGTTAAAGAATCCTCGCACTTCTG

GGATGCAATTTCGTGATTTTTTTCTCCATCTCTGGGGGAGTTTGTGTCCC

TTGAATTTTAGTTGCATTCATCTGTTAAACTGGGATTATTGTGTTTGTAG

TGTGTGGTATGGTTGGTTTTAGGGGCTATTTAGATTTACTGTTTCTTCTA

GAAAGTTGTAGGTTTTTCTAAAAATGTATCCTTTTTGGTCATCGAGTTGT

TTAAAATATTTTTATTTTCAAGTACTTGTTCTATTTGAACTTAGTCCCTT

TTTTCTTTCCTGATATTGCTTATTTGTATCCTCTCTTGGTAATGTTTCTA

GAGATGTGTCAACTTTACTAGTCTTTTAAAATAACTGACTTTTTGAACTT

TTGCAAAAATCAACTTTTATCAAAGTGCACAATAAAATTCACCCACTTAA

AGGTGTTTGCTATGTTTGACGAAGATATTACCCTTGTAACTACCACCATA

ATTGTGTGTGTGGTTATAATAGTTCCTTCTTCTCCTCCTCCCTCCCTCTC

TCTTCCTCCTCCCTATCTTCCTCCTTCTCCTTTCACCCCTTTCTCTTTCT

TCCTCCCTTCTTCCTTTCTTCCTTCCACAGTTCTGTCTTAAAATGACCTA

TGTAGACTTGTGTCACCATCACCACAAAGGGTACAGAACAATTCCACTGC

ACCAACAAATTCCACCTGTGCTGCTTTTTTTTAAGAGTGGCTCCCCACCC

CGCATTGAGTATAAAGGCTTTGATTTTCATCAGTGTTGTGTCATTTATTC

ATTTTTATTGCTGAGTAGTTTTCCATGTTAGGTACCACAATATTCTTAGC

CATTAACCCACTGAAGCACATTTAGGTTGTTTATAATTTTTAGTGATTAT

GAATAAAACTATTAGAAACATTAGGTTTTTGTATGAACATAAATATTCAT

CTCTGTAGGGTAAATATTGGAGTGAAATGGTTAGGTCATATGGTGAGTGT

ATGTTTACCTTTGTAACACAGGGCCAACCTTTTCCAGAATGATTAATTTT

GTTTTCCCACCAGCAACGTATGAAGATGTGGTTGCTCCATAATTTGCCAA

CACCAGGTATTCTCATTGTTTTTATTTTATCCATTCTAGTAGATGTGTAG

TGGTATCTCAATGTGGCTTCAATTTGCATTTCCTTAGTGACTAATGATGT

TGAGCATCTTTTCGTGTGCTTATTGTCCATCTGTGTATCTTTGATGAAGT

GTTCAAATCTTTTGCCATTTTAAAAGAATTCTATTGTTTGTTTTCCTAGT

TTAGTTTTGTAGTTTGTGGCTTGTCTTTTTATTCTCTTTTATTCAATTTT

TATTTCAGTGTCTTTTATAAAGCAAAGGTTTTAAATGTTAATGAAGTCCA

GTTTTTCCCTCTATGGACCATACTTTGGTGTCATATTTAAGAACCCCATC

CCTAACTAATGTTTAGGAAATTTTTCTTCTAGTAGCTTAATGATAATGTT

TTTCACTTACATCCATTTTAGGTAATTTTTATATAAGGTGTTAAATAAAG

GCCCAGCTCATTGTTTTTACATGTGGTTATCCAGATGTTTCAGCATCATT

TGTGGAAAACATAGTGTTTCTCCATTGAATTGCCTTTGCCCTTTTGTCAA

CAATTGATTGATCATAGTTAAGCGGGTCTGTTTCTCAGTTTTCTGTTCTA

TTCCATTGATCTATGTGTCCATCCCTTTGCCAAAGCCACACATTGTCTTG

ATTACTGTAGCTTTGTAGTAAATCTCAAAATCAGGTAATGTAAGCCCTTC

AAATTTATTCTTTTTCTAATTTATATGTTTCCACTGTCATATATTTGTCT

ATATATTATGTCAAGTTTTACTTTATATATTTTGACACTCAGTTGTTATC

CAAATACAGGCTAAGAATTGTCAAATCTCTGGTGATTTGAATGTTTTCCC

ATTATGTAGCAACTTTCTTTAAACATAAGAATACTTTATAACAGATAATA

TTGGGGTTTTTTTGGGGGGGAGGAGGTTTGAAACAAGGTCTCACTCTCTC

ACCCAGGCTGGA

>hg19_ct_ARAlincRNAs_9727_ARAlincRNA_0303.1 range=chr2:96199792-96201769 5'pad=0 3'pad=0 strand=- repeatMasking=none

AAGAGCATGATGATTAGTCTGTTCTGTACTGGATGTCATGTAGTGCCTAG

GTATCAATGATAAAAATTTCAGTGGAATCTGTGAGAATACCTTCCTTCCT

TGAACTTACATTTCACTGAAGGAGTGATGAAGTTAATAATCATTATAATA

ATTTGACTATTTAGTGTAATGTTCAAGGCACTGTAAAGAGCTCAATATCA

GGAAGAGTTTCTGGCTATCCAAACTACAAGTTCAAAAGGCTTCTATATAA

GAAACCTATGTACCAACACTGGAAATAATAGAATAAAATATGCTAGAATT

AGCAAGGCATGGTGTCAGTAGATTCCAATTCTGCTGCAAGATGCCACATT

ATCTATAAATTAGCCCTGCTTATGATTTTCCTATAAACTTGTTGCATTAC

ATGTTGTGGTTCTACAGTCTGAGATCTTCCCAAATCTTTCATATCTCATC

AGTTGATTTCTTTATCACTGGGGGTCTGAAACCCAAAACGATTTGCTTCT

CTGATGTTCACATTCATAGTTCTTTTACAGGAGAAGATCATGAAGCAAAT

GAGATCTTTATGGGCAAAAATTCAAGAAAACCAAAGAAATTTAAGTGAGA

AAAGCAGGAAACCCAGCCAGTGGATTGTAAGTATTAGGCCTTTTCCCTCA

GATTCAACCTCAGACAGACATGCTAGAAATGTATCCACTTATCACTTGAA

TGGAAATCGTGTTGGTTAGGATTTGAGGAAGATTTTTTCTCATGGCTTCC

AATCCTGAGGGTACAATGCAGCATTGATTACTGCTCAGAGAGAGTGGTCA

GGCTATGCAGTAGGGAGACTTGGTACTAAAGACTAATTTAAAAACACAGA

GATCATAATACTGCAAACAATTATCTGTGAATCAACCATTAATTCCAATG

GCTCTCATGTAGGTTTTTCAATATCAAAGGGTATATATTGAAGAAATGAT

CAATAATAACCTTTCTTCAGGTGTTTCAGGAAGTCATATAATGAATAAAA

GTGGTATGAGAAAAGAATGAATTTGACTTCTAGTATTGTTAAGAAACAAT

AAAGATAAATGAAAGAAAGGGAGGAATGATTGATTTCATGGTTCTGAGAA

GTAGGAAGACACAAATTTATGTACATAGCTTATTGGTAGAATACATGAGT

CAGTCTAGGCTTGGTGTCTCAAGTCCATAATCCGTATAATTTGGGAGACC

GAAGCAGGAGAATCACTTGAGACCAGGAGTTTCAAACCAGCCTGGCCATC

AACATAGTGAGACCCCTAACTTTATTATGAAGAAAAGATGAAAGAGATAA

AGAAAAGAGAACCTGAGTTAAAGTGAATAAAGGATGTGTAAGAGGAGGAG

GATGAAGTCAGAATAAATATGTCATCTAGGAAATCCAGGTCCCTGCAGTG

CTATGTGCAGCTATGGGCAGAGATGACCAGAGCTGAGTAGAGGAAGCTGC

ATTCAGTTCCCTATGAGAAGTAAAACAACATATAGAGAAACTTACAGAGG

ATGACAGAAAGATTTTACAGCAACTTAAGAAAAGCAAAAACGCAAAATGG

TTCAGAAAAAAAAAAACACCTAAGAAAAATCTATAAGGAGCTGATGAAAA

TGTGCCATGATCTAGATGTGGAGCTGCTCCAGGAAAGAACCAATAACATG

CCTAAAAAATTTTTATATTATTTGAAGTTCATACCTTACAACAAGCTTGT

CCAACCCATGGTCTTTGGGCCACACATGGCCAAGGATGGCTTTGAACATG

GCCCGACACAAATTCCTAAACTTTCTTGAAACATGATGAGATTTTTGTTT

GTCATTTTATTAAAGCTCTTCAGCTATAGTTAATGTTAGTGTATTTCATG

TGTGGCCCAAGACAATGCTTTTTTCTAATGAGGCCCAGGGAAGCCAAAAG

ATTGCACACCACTGGCTTAGGTGATATTATTTATTCAACACCATAGATGT

GTGTGTGTATATATATGTATATGTGTGT

>hg19_ct_ARAlincRNAs_9727_ARAlincRNA_0304.4 range=chr2:96472866-96480524 5'pad=0 3'pad=0 strand=- repeatMasking=none

GAGAAGGGAAGGAAGCCCATGTGAGGACACTGAAGAAGGAGTGAGCAGAC

AATAAGAAGCCCACAGAAGACAGAGAAGGAACAACTAGAGGGAGAAGCCA

AGGCAGTTTCTCCACAGACACTACCCAAAGCAGTCCTTCACTACAGTGGC

AGACAGACCTGAAAATTTTCATCTGAAGCAGCAGAGTGAACTGCAGAGTC

AGAGATAGAATCTCACTATGTTGACCAGGCTGGTCTTGAACTCTTGGCAC

CCAAGCGATCCTTTTGCCTGGAATCCCAAAGTGTCAGATTTACTGAAGAA

TATTTCATTGTAATTACTTTTTATACTTTATAGGTCAAGAGCTCTGTTTT

AAAGACAAAATTTATTGAATATACTTTTTCAAACAAA

>hg19_ct_ARAlincRNAs_9727_ARAlincRNA_0304.1 range=chr2:96472866-96480539 5'pad=0 3'pad=0 strand=- repeatMasking=none

GTGATGGCATCAGCTGAGAAGGGAAGGAAGCCCATGTGAGGACACTGAAG

AAGGAGTGAGCAGACAATAAGAAGCCCACAGAAGACAGAGAAGGAACAAC

TAGAGGGAGAAGCCAAGGCAGTTTCTCCACAGACACTACCCAAAGCAGTC

CTTCACTACAGTGGCAGACAGACCTGAAAATTTTCATCTGAAGCAGCAGA

GTGAACTGCAGAGTCAGAGATAGAATCTCACTATGTTGACCAGGCTGGTC

TTGAACTCTTGGCACCCAAGCGATCCTTTTGCCTGGAATCCCAAAGTGTC

AGATTTACTGAAGAATATTTCATTGTAATTACTTTTTATACTTTATAGGT

CAAGAGCTCTGTTTTAAAGACAAAATTTATTGAATATACTTTTTCAAACA

AA

>hg19_ct_ARAlincRNAs_9727_ARAlincRNA_0304.5 range=chr2:96472866-96485133 5'pad=0 3'pad=0 strand=- repeatMasking=none

ATCAAACTACCATTGACATTCTTCACAGAATTAAAAAAAAAACTACTTTA

AATTTCATATGCAACCAAAAAAGAGACTCAATAGTCAAGACAATCCTAAG

CAAAAAGAACAAAGCTGGAGGCATCATGCTATATGACTTCAAACTATACT

GCAAGGCCACACTAATCAAAACAGCATGGTACTGATACCAAAACAGACAC

ACAGACCAATGGAACAGAATAGAGATCTCAGAAATAAGACCACACATCTA

CAACCATCTGATCTTTGACAAACCTGACAAAAACAAGCAATAGGGGAAGG

AATACCTATTTATTCATTTATTTATTTTGAGACAAAGTCTCACTCTGTCA

CCAGGCTGGAGTGCAGTGGCATGATCTCAGTTCATTGCAACCTCTGCCTC

CCGGATTCAAGTGATTCTCCTGCCTCAGTCTCCCGAGTAGCTGGGACTAC

AGGTTCGAGCCACCACGCCCAGCTAGTTTTTGTATTTTTAGTAGAGACGG

GATTTCACCATGTTGGCCAGGATGGTCTTGATCTCTTGACCTCAAGATCC

ACCCATATCAGCCTCCCAAAGTGCTGAGATTATAGATGTGAGCCACCGCA

CTTGGCCAGGATTCCCTATTTAAATGGTGCTGGGAAAACTGACTAGCCAT

ATGCAGAAAACTGAAACTGGACCTCATCCTTACACCTTATGCAAAAATTA

ACTCAAGATGGATTAAAAACTTAAATGTAAAACCCCAAACCGTAAAAAAC

CCTAGAAGAAAATCTAGGAAGTTCCATTCAGGACATAGGCATAGGCAAAG

ATTTTATGATGAAATCATCAATAGCAATTGCAACAAAAGCAAAAATTGAC

AAATGGGATCTAATTAAACGTAAGCACTTCTGCACAGGGAAAGAAACTAT

CATCAGAGTGAACAAGCAACCTATAGAATGGGAGAATATTTTTGCCATCT

ACCAATCTGACAAAGGTCTAATATCCAGAATCTACAAGGAACTTAAACAA

ATTTACAAGAAAATAATAACCCCATCAAAATGTGGGCAAAGGCCATGAAC

AGACATTCTGAAAAGAAGACATTTATGCGTCCAACAAACATATGAAAAAA

AAAAAGCTCAACATCAGTGTTTATTAGAGAAATGCAAATCAAAACCACAA

TGAGATACCATCTCATGCCAGTCAGAATGGCAATTATTAAAAAGTCAAGA

AACAACAGATGCTAGAGAGGCTATGGAGAAACAGGAACACTTTTATACTG

TTGGGAACGTAAACTAGTTCAACCATTGTGGAAGACAGTGTGGCAATTCC

TGAAGGATCTAGAAGCAGAAATACCATTTGACCCAGCAATCCCATTACTA

GGTTTATATCCAAAGAAATCTAAATCATTCTATTTTAAAGATACATGCAC

ACTTATGTTTATTGCAGCACTATTCACAATAGCAAAGACATGGCATCAAC

CCAAATTTCTATCAATGATAGATTGGATAAAGAAAATGTGATACATATGC

ACCATGGAATACTATGCAGCCATAAAAAGGAATGAGATCCTGTCTTTTGC

AGGGACATGGATGAAGCTGGAAGCCATAAACTTCAGCCAATTAACACAGG

AACAGGAAACCAAACACCACATGTTCTCATAAGTGGGAACTGAACAATGA

GAACACATGGACACGGAGGGGCAGAACACACACCGGAGCCTGTTGGGAAG

GTAGGGGGAAGGAGAGCATCAAGATAAATAGCTAATGCATGTGGGGCTTA

ATACCTAGGTGATAGGTTGATAGGTGCAGCAAACCACCATGGCACATGTT

TAACTATGTAACAAACCTGCACATCCTGTACATGTATCCTGGAACTTAAA

ATAAAATAGAAAAGACAAAGAAGGATATTACATAATGGCAAAGGCTTCAA

TTCAACAAGAAGACCTAACTATCCTAAATATATATTCATCCAACACAGGA

GCACCCAGATTCATAAGTAAAGTTCTTAGAGACCTACAAAGTATGTTTCA

CAGTAATAGTGGGAGACTTTCACACTCCAATGACAGTATTAGATCATTGA

GGCAAAAAAATGAACAAAGATATTCAGGACCTGAACTCAACATTGGATCA

AATGGATCTGATAGACCTTTACAGAACTCTGCACTCAAAAACAACAGAAT

ATGCATTCCTCACATCATCATATGCCACATACTCTAAAATCAACCACATA

ATTGGACATAAAGCAATCCTCAGCAAATGCAAAACAACTGAACTCATACC

AAATACACACTGAGATCACAGTGCAGTAAAAATAGAAGACTAAGAAAATT

GCTGAAAATCATGCAATTACATGGAAATCAATCAACATGCTCCTGAATGA

CTTTTTAATAAATAATGAAATTAAGGCAGAAAACAAGAAGTTCTTTGAAA

ATAATGAGAACAAAGTTGCAACATACTAGAGCCTCTGGACATAGCTAAGG

CAATGTTAGGAGGGAAATTTATAGCACTAAATCCCACATCAAAAAGTTAG

GAAGAACTCAAATTAATAACCTAACATCACAACGGAAAGAACTAGAGAAG

CAAGACAAAACCCCAAAGCTAGAGGAAGACAAGAAATAACTGAAAATCCG

AGCTGAATTCAAAGAAACCGAGACATGAAGAAAAAAAAATTCAAAAGATC

TACGAATTCAGGGTAGGTTTCTTGAAAATATTAATAAGAAAGTCTGCTAG

CAGACTAATACAGAGGATGATTGAAATAAACACAATTAGAAATGACAAAG

GGAATGTAATTACCACTGACCCCACAGAAATAGAAACAACCATCAGAAAC

TACTGCAAACACTTCTGTGCATACAAACTAGAAAACTTCAAAGAGATGGA

TAAATTCCTGGACAAATACACCCTCCCACAACTGAGCCAGGAAGAAATTG

ATTTGCTGAACAGACCAATAACAAGCTCCGAAATTGAATCAGTAATAAAT

AACCTACCAATCAAAAAAAGCCCAGAACATGATGGATTCCCAGCCATATT

CTACTAGAAGTACAAAGAAGAGCTGGTACCATTTTTACAGGAACTATTTG

AAAATATTGAGGAGGAGGAACTCCTCCCCAACTCATTCTATGAGGCCAAC

ATCATCTTGATACCAAAATCTGGCACACACACACACACACACACACACAC

ACACACACACACACAAACTTCAGGCCAATACCCTTGATGAACATCAATGC

AAAAATCCTCAACAAAATACGGGCAAACCAAATCCAGCAGCACATCAAAA

AGTTAATCCATCATGATCAAGTATGCTTCATCCCCAGGATGCAAGGTTGC

CTCAACATACACGTCAATTAATCTGATTCATCCCATAAACAAAACTAAAG

ATAAAAACCATGTGATTATCTCAATATATGCAGAAAAGGCTTTCAATAAA

ATTCAAGGCCTCTCCATATTAAAAACTCTAAAAAATCTGGGTATTGAGGA

AACATAGCTCAAAGTGATGAGCTGTTTTTGTACCAGTATCATGCTGTTTT

GGTTACTGTAGCCCTGTAGTATAGTTTGAAGTTGGGTAACATGATGCCTC

CAGCTTTGTTCTTTTTGCTGAGGATTGCTTGGCTATTAGGGCTCTTTTTT

TTTTTGGTTCCATATGAATTTTGAAATAGTTTGCTCTAGTTCTGTGAGGA

ATGCCATTGGTAATTTAATAGGGATAACTTGCATCTGTAAATTACTTTGG

GCAGTATAGCCATTTTAATGATGTTAATTCTTCCTATCCATGAGCATGAA

ATGTTTTTCCATTTGTTTGTGTCTTCTCTGATTTCTTTGAGCAGTGTTTT

GTAATTCTCCTAGAGATCTCTTAGAGATCTTTCACCTCCCTGGTTAGCTG

TATTCCTAGGTATTTTATTTTTTCTGTGTGTAGCAATTATGAATGGGATT

ATGTTCTTCATTTGACTCTCTGCTTTACTGCTAGGAATTTTTGCACATTG

ATTTTGTATGCTAAGACTTTGCTAAAGTTTATCAGCAGAAGGAGCTTTGG

GGCTAAGACTATGGGGTTTTCTAGATATAGAATCATGTCATCTGTAAACA

GAGACAGTTTGACTTCCTGTCTATTCCTCTCTTCCCTCCTCTATTTGGAT

GCCCTTCCAGTTTTGCACATTCAGTGTAATGTTGGCTGTGGGTTTGCCAT

GGCTAGCTCTCATCATTTTGGCACTTCCGACTAGAAGTGCGAGAGGGGCC

AGTGTTGTTGTTACCTGAAAGGTAAGTGCAGCCCACAAAAATGCAGTGAA

GAAGAAGATATATGCATATGTTTAAGTTAATACAATTGAGATACATTTAG

CAGACAGAATCAATAGAGTTGATGAGTGACTGACTGGATGTGTGGGGAGT

TTATATCACTCCCAGGTTTTTGACTTGGGCAACCGGGCACTCAAGAAGGA

AAAAGAGGATCCAGGGAGAGGAACTTTTCTGAGGACTGGGGTAGGGCTGA

ACAGCTGCATTCGAGACTGGTGGAGGGTGGGCTGGGCATGGGATGCACAA

ATGGAAATTCCACTGGGTCTGCAGCTCACACATAGGCATGACCAGCATAG

AGATAGAGAGGCCCCAGTGCTGCTGAGTAACTGTGATTCCCCAGGTGATG

GCATCAGCTGAGAAGGGAAGGAAGCCCATGTGAGGACACTGAAGAAGGAG

TGAGCAGACAATAAGAAGCCCACAGAAGACAGAGAAGGAACAACTAGAGG

GAGAAGCCAAGGCAGTTTCTCCACAGACACTACCCAAAGCAGTCCTTCAC

TACAGTGGCAGACAGACCTGAAAATTTTCATCTGAAGCAGCAGAGTGAAC

TGCAGAGTCAGAGATAGAATCTCACTATGTTGACCAGGCTGGTCTTGAAC

TCTTGGCACCCAAGCGATCCTTTTGCCTGGAATCCCAAAGTGTCAGATTT

ACTGAAGAATATTTCATTGTAATTACTTTTTATACTTTATAGGTCAAGAG

CTCTGTTTTAAAGACAAAATTTATTGAATATACTTTTTCAAACAAA

>hg19_ct_ARAlincRNAs_9727_ARAlincRNA_0304.6 range=chr2:96472866-96485206 5'pad=0 3'pad=0 strand=- repeatMasking=none

ATGGGTGGGAAGAATCAGTCTCATGAAAATGGCCATACTGCCCAAAGTAA

TTTATAGATTCAGTGCTATTCACATCAAACTACCATTGACATTCTTCACA

GAATTAAAAAAAAAACTACTTTAAATTTCATATGCAACCAAAAAAGAGAC

TCAATAGTCAAGACAATCCTAAGCAAAAAGAACAAAGCTGGAGGCATCAT

GCTATATGACTTCAAACTATACTGCAAGGCCACACTAATCAAAACAGCAT

GGTACTGATACCAAAACAGACACACAGACCAATGGAACAGAATAGAGATC

TCAGAAATAAGACCACACATCTACAACCATCTGATCTTTGACAAACCTGA

CAAAAACAAGCAATAGGGGAAGGAATACCTATTTATTCATTTATTTATTT

TGAGACAAAGTCTCACTCTGTCACCAGGCTGGAGTGCAGTGGCATGATCT

CAGTTCATTGCAACCTCTGCCTCCCGGATTCAAGTGATTCTCCTGCCTCA

GTCTCCCGAGTAGCTGGGACTACAGGTTCGAGCCACCACGCCCAGCTAGT

TTTTGTATTTTTAGTAGAGACGGGATTTCACCATGTTGGCCAGGATGGTC

TTGATCTCTTGACCTCAAGATCCACCCATATCAGCCTCCCAAAGTGCTGA

GATTATAGATGTGAGCCACCGCACTTGGCCAGGATTCCCTATTTAAATGG

TGCTGGGAAAACTGACTAGCCATATGCAGAAAACTGAAACTGGACCTCAT

CCTTACACCTTATGCAAAAATTAACTCAAGATGGATTAAAAACTTAAATG

TAAAACCCCAAACCGTAAAAAACCCTAGAAGAAAATCTAGGAAGTTCCAT

TCAGGACATAGGCATAGGCAAAGATTTTATGATGAAATCATCAATAGCAA

TTGCAACAAAAGCAAAAATTGACAAATGGGATCTAATTAAACGTAAGCAC

TTCTGCACAGGGAAAGAAACTATCATCAGAGTGAACAAGCAACCTATAGA

ATGGGAGAATATTTTTGCCATCTACCAATCTGACAAAGGTCTAATATCCA

GAATCTACAAGGAACTTAAACAAATTTACAAGAAAATAATAACCCCATCA

AAATGTGGGCAAAGGCCATGAACAGACATTCTGAAAAGAAGACATTTATG

CGTCCAACAAACATATGAAAAAAAAAAAGCTCAACATCAGTGTTTATTAG

AGAAATGCAAATCAAAACCACAATGAGATACCATCTCATGCCAGTCAGAA

TGGCAATTATTAAAAAGTCAAGAAACAACAGATGCTAGAGAGGCTATGGA

GAAACAGGAACACTTTTATACTGTTGGGAACGTAAACTAGTTCAACCATT

GTGGAAGACAGTGTGGCAATTCCTGAAGGATCTAGAAGCAGAAATACCAT

TTGACCCAGCAATCCCATTACTAGGTTTATATCCAAAGAAATCTAAATCA

TTCTATTTTAAAGATACATGCACACTTATGTTTATTGCAGCACTATTCAC

AATAGCAAAGACATGGCATCAACCCAAATTTCTATCAATGATAGATTGGA

TAAAGAAAATGTGATACATATGCACCATGGAATACTATGCAGCCATAAAA

AGGAATGAGATCCTGTCTTTTGCAGGGACATGGATGAAGCTGGAAGCCAT

AAACTTCAGCCAATTAACACAGGAACAGGAAACCAAACACCACATGTTCT

CATAAGTGGGAACTGAACAATGAGAACACATGGACACGGAGGGGCAGAAC

ACACACCGGAGCCTGTTGGGAAGACACTACCCAAAGCAGTCCTTCACTAC

AGTGGCAGACAGACCTGAAAATTTTCATCTGAAGCAGCAGAGTGAACTGC

AGAGTCAGAGATAGAATCTCACTATGTTGACCAGGCTGGTCTTGAACTCT

TGGCACCCAAGCGATCCTTTTGCCTGGAATCCCAAAGTGTCAGATTTACT

GAAGAATATTTCATTGTAATTACTTTTTATACTTTATAGGTCAAGAGCTC

TGTTTTAAAGACAAAATTTATTGAATATACTTTTTCAAACAAA

>hg19_ct_ARAlincRNAs_9727_ARAlincRNA_0304.3 range=chr2:96479064-96481963 5'pad=0 3'pad=0 strand=- repeatMasking=none

CAGGCCAATACCCTTGATGAACATCAATGCAAAAATCCTCAACAAAATAC

GGGCAAACCAAATCCAGCAGCACATCAAAAAGTTAATCCATCATGATCAA

GTATGCTTCATCCCCAGGATGCAAGGTTGCCTCAACATACACGTCAATTA

ATCTGATTCATCCCATAAACAAAACTAAAGATAAAAACCATGTGATTATC

TCAATATATGCAGAAAAGGCTTTCAATAAAATTCAAGGCCTCTCCATATT

AAAAACTCTAAAAAATCTGGGTATTGAGGAAACATAGCTCAAAGTGATGA

GCTGTTTTTGTACCAGTATCATGCTGTTTTGGTTACTGTAGCCCTGTAGT

ATAGTTTGAAGTTGGGTAACATGATGCCTCCAGCTTTGTTCTTTTTGCTG

AGGATTGCTTGGCTATTAGGGCTCTTTTTTTTTTTGGTTCCATATGAATT

TTGAAATAGTTTGCTCTAGTTCTGTGAGGAATGCCATTGGTAATTTAATA

GGGATAACTTGCATCTGTAAATTACTTTGGGCAGTATAGCCATTTTAATG

ATGTTAATTCTTCCTATCCATGAGCATGAAATGTTTTTCCATTTGTTTGT

GTCTTCTCTGATTTCTTTGAGCAGTGTTTTGTAATTCTCCTAGAGATCTC

TTAGAGATCTTTCACCTCCCTGGTTAGCTGTATTCCTAGGTATTTTATTT

TTTCTGTGTGTAGCAATTATGAATGGGATTATGTTCTTCATTTGACTCTC

TGCTTTACTGCTAGGAATTTTTGCACATTGATTTTGTATGCTAAGACTTT

GCTAAAGTTTATCAGCAGAAGGAGCTTTGGGGCTAAGACTATGGGGTTTT

CTAGATATAGAATCATGTCATCTGTAAACAGAGACAGTTTGACTTCCTGT

CTATTCCTCTCTTCCCTCCTCTATTTGGATGCCCTTCCAGTTTTGCACAT

TCAGTGTAATGTTGGCTGTGGGTTTGCCATGGCTAGCTCTCATCATTTTG

GCACTTCCGACTAGAAGTGCGAGAGGGGCCAGTGTTGTTGTTACCTGAAA

GGTAAGTGCAGCCCACAAAAATGCAGTGAAGAAGAAGATATATGCATATG

TTTAAGTTAATACAATTGAGATACATTTAGCAGACAGAATCAATAGAGTT

GATGAGTGACTGACTGGATGTGTGGGGAGTTTATATCACTCCCAGGTTTT

TGACTTGGGCAACCGGGCACTCAAGAAGGAAAAAGAGGATCCAGGGAGAG

GAACTTTTCTGAGGACTGGGGTAGGGCTGAACAGCTGCATTCGAGACTGG

TGGAGGGTGGGCTGGGCATGGGATGCACAAATGGAAATTCCACTGGGTCT

GCAGCTCACACATAGGCATGACCAGCATAGAGATAGAGAGGCCCCAGTGC

TGCTGAGTAACTGTGATTCCCCAGGTGATGGCATCAGCTGAGAAGGGAAG

GAAGCCCATGTGAGGACACTGAAGAAGGAGTGAGCAGACAATAAGAAGCC

CACAGAAGACAGAGAAGGAACAACTAGAGGGAGAAGCCAAGGCAGGCATG

TGTGATAACATATAGGAACTGAGGGAGAGGACATTTCAAGATGGAGGGGA

TGCCATACAACAGGACTATGTGATGGTTTTTGGCTGTGTCCATAGGAAGT

CACAACAGGCAAGGGAAAGAAACCAGAACCCAGTCATGGAGTTAAGAAGT

GAGTCAGAGAGGAGATGGGTAGGGACAGTGAGGTAAGGCCTCTTTCTAAG

GAAGTTTGGCTGAAGGATAGACTAGCTGGACACATGCTGGCTGTGTGGGG

TAGAGGGAGGAATGATGGAGGGTAGGAGAGCCTTGAGCCTGCGAGAAGAG

TCTCCTAGAATAGAGAAGCTGAGGTTAAAGTTGTGGAAGACAGTGGGGAT

AACTGAGTGACAGATAATCAGGAGAAGAAAAGGAGATCCAGAATCATGAC

AGAGAGATGACCTTTGCCAAGAGCACAGCCATCTTTCACTGTCACAGAGA

GGTAGGACAAAACGATTGGTGTTCAAGAATTGGTTTGTAGCACAATATTT

TAACTATGTCCTTTAAAAAGTTTCTCCACAGACACTACCCAAAGCAGTCC

TTCACTACAGTGGCAGACAGACCTGAAAATTTTCATCTGAAGCAGCAGAG

TGAACTGCAGAGTCAGGTCACCAACCAGTGTGGAGGCTAAAAGCGGGCCT

CTTTGGGGATTCCAGGAACAAAGATCACCACAACTGGCTCCCCTTCAACT

ATGTTAGATGGCAACTTGCCTTCAGTATGGTGAAACACATCAGTTAAGAC

TGGGGTTGTGCATGGCAGGACTTTCTACAAGGACACCCAGTCTCCTTAAT

AAACATGAGATGCTCTCTTTCCAGAAGTTCTCTTGCCTGACACAGCATAG

GAAGATGCTGAACGGCCACACAGTGACCCATTG

>hg19_ct_ARAlincRNAs_9727_ARAlincRNA_0304.2 range=chr2:96482472-96485133 5'pad=0 3'pad=0 strand=- repeatMasking=none

ATCAAACTACCATTGACATTCTTCACAGAATTAAAAAAAAAACTACTTTA

AATTTCATATGCAACCAAAAAAGAGACTCAATAGTCAAGACAATCCTAAG

CAAAAAGAACAAAGCTGGAGGCATCATGCTATATGACTTCAAACTATACT

GCAAGGCCACACTAATCAAAACAGCATGGTACTGATACCAAAACAGACAC

ACAGACCAATGGAACAGAATAGAGATCTCAGAAATAAGACCACACATCTA

CAACCATCTGATCTTTGACAAACCTGACAAAAACAAGCAATAGGGGAAGG

AATACCTATTTATTCATTTATTTATTTTGAGACAAAGTCTCACTCTGTCA

CCAGGCTGGAGTGCAGTGGCATGATCTCAGTTCATTGCAACCTCTGCCTC

CCGGATTCAAGTGATTCTCCTGCCTCAGTCTCCCGAGTAGCTGGGACTAC

AGGTTCGAGCCACCACGCCCAGCTAGTTTTTGTATTTTTAGTAGAGACGG

GATTTCACCATGTTGGCCAGGATGGTCTTGATCTCTTGACCTCAAGATCC

ACCCATATCAGCCTCCCAAAGTGCTGAGATTATAGATGTGAGCCACCGCA

CTTGGCCAGGATTCCCTATTTAAATGGTGCTGGGAAAACTGACTAGCCAT

ATGCAGAAAACTGAAACTGGACCTCATCCTTACACCTTATGCAAAAATTA

ACTCAAGATGGATTAAAAACTTAAATGTAAAACCCCAAACCGTAAAAAAC

CCTAGAAGAAAATCTAGGAAGTTCCATTCAGGACATAGGCATAGGCAAAG

ATTTTATGATGAAATCATCAATAGCAATTGCAACAAAAGCAAAAATTGAC

AAATGGGATCTAATTAAACGTAAGCACTTCTGCACAGGGAAAGAAACTAT

CATCAGAGTGAACAAGCAACCTATAGAATGGGAGAATATTTTTGCCATCT

ACCAATCTGACAAAGGTCTAATATCCAGAATCTACAAGGAACTTAAACAA

ATTTACAAGAAAATAATAACCCCATCAAAATGTGGGCAAAGGCCATGAAC

AGACATTCTGAAAAGAAGACATTTATGCGTCCAACAAACATATGAAAAAA

AAAAAGCTCAACATCAGTGTTTATTAGAGAAATGCAAATCAAAACCACAA

TGAGATACCATCTCATGCCAGTCAGAATGGCAATTATTAAAAAGTCAAGA

AACAACAGATGCTAGAGAGGCTATGGAGAAACAGGAACACTTTTATACTG

TTGGGAACGTAAACTAGTTCAACCATTGTGGAAGACAGTGTGGCAATTCC

TGAAGGATCTAGAAGCAGAAATACCATTTGACCCAGCAATCCCATTACTA

GGTTTATATCCAAAGAAATCTAAATCATTCTATTTTAAAGATACATGCAC

ACTTATGTTTATTGCAGCACTATTCACAATAGCAAAGACATGGCATCAAC

CCAAATTTCTATCAATGATAGATTGGATAAAGAAAATGTGATACATATGC

ACCATGGAATACTATGCAGCCATAAAAAGGAATGAGATCCTGTCTTTTGC

AGGGACATGGATGAAGCTGGAAGCCATAAACTTCAGCCAATTAACACAGG

AACAGGAAACCAAACACCACATGTTCTCATAAGTGGGAACTGAACAATGA

GAACACATGGACACGGAGGGGCAGAACACACACCGGAGCCTGTTGGGAAG

GTAGGGGGAAGGAGAGCATCAAGATAAATAGCTAATGCATGTGGGGCTTA

ATACCTAGGTGATAGGTTGATAGGTGCAGCAAACCACCATGGCACATGTT

TAACTATGTAACAAACCTGCACATCCTGTACATGTATCCTGGAACTTAAA

ATAAAATAGAAAAGACAAAGAAGGATATTACATAATGGCAAAGGCTTCAA

TTCAACAAGAAGACCTAACTATCCTAAATATATATTCATCCAACACAGGA

GCACCCAGATTCATAAGTAAAGTTCTTAGAGACCTACAAAGTATGTTTCA

CAGTAATAGTGGGAGACTTTCACACTCCAATGACAGTATTAGATCATTGA

GGCAAAAAAATGAACAAAGATATTCAGGACCTGAACTCAACATTGGATCA

AATGGATCTGATAGACCTTTACAGAACTCTGCACTCAAAAACAACAGAAT

ATGCATTCCTCACATCATCATATGCCACATACTCTAAAATCAACCACATA

ATTGGACATAAAGCAATCCTCAGCAAATGCAAAACAACTGAACTCATACC

AAATACACACTGAGATCACAGTGCAGTAAAAATAGAAGACTAAGAAAATT

GCTGAAAATCATGCAATTACATGGAAATCAATCAACATGCTCCTGAATGA

CTTTTTAATAAATAATGAAATTAAGGCAGAAAACAAGAAGTTCTTTGAAA

ATAATGAGAACAAAGTTGCAACATACTAGAGCCTCTGGACATAGCTAAGG

CAATGTTAGGAGGGAAATTTATAGCACTAAATCCCACATCAAAAAGTTAG

GAAGAACTCAAATTAATAACCTAACATCACAACGGAAAGAACTAGAGAAG

CAAGACAAAACCCCAAAGCTAGAGGAAGACAAGAAATAACTGAAAATCCG

AGCTGAATTCAAAGAAACCGAGACATGAAGAAAAAAAAATTCAAAAGATC

TACGAATTCAGG

>hg19_ct_ARAlincRNAs_9727_ARAlincRNA_0305.1 range=chr2:96490039-96493285 5'pad=0 3'pad=0 strand=- repeatMasking=none

AAAATCTTCAACCAAGAATTTCATATCCAGCTAAACTAAGCTTCCTAAAT

GAAGGAGAAATGAGATCATTTACAGACAAGCAAATTCTGAGGTAATTCAT

TACCACCACATCTGCCTTACAAGAGATTTTAGAAAGGAGGACTAAATATA

GAAAGGAAAGACCACTACACACTAATACAAAAACATACTTAAACACACAG

ACCAGTGACACTATAAAGCAACCACACAAACAAGCCAACATAATAACCAG

CCAACAGCACAATGACAAGATCAAATCTACACAAATCAATACTAACCTTG

AAAGTAAATGGACAAATGCCCCACTTAAAAGGCAGAGTGGCAAGCTAGAT

TAAAAAAAAAAAAAAGCGAGACCCAATGGTATGTCGTCTTCAAGAGACCC

ATCTCACACATAATGACACTCATCGTCTCAAACTAAAAGGATGGAGGAAA

ATCTACCAGACAAATAGAAAACAGAAAAAAAGCAGAGGTTGCAATCCTAA

TTTCAGACAAAACAGATTTCAAATGAACAATAATTTTTCAAAAAGACAAG

GGGGCAGGGGCAAGATAGCCAACTAGAAGCAGCTGCAATTCGAGGCTCCC

ACTGAGAAGAACTAAAACAGTGTGCAAATCCTGCACCAGCAACTGACATA

TCCAGGTTCTATGATCAGGACTGACTAGGTAGTTGGCATGGCCCATAGAG

AACAAGGAAAGATGGGCTGGTGGATTGGCCCACCTGGGAGCCACATGGGG

CAAGGGGAGCCCTCACCCTCAGCCAGCCAAGGGAGGCAGTGAGTGAGCAT

GCTACCCAGCCTGGGAAACTGCTTTTTCCATGGATCTTTGCAATCCACAG

ATCAGAAGATCCCACTCATGAGACCACACCACCAGGGCCTTGGGTGCCAA

CTACAGAGCCATGCAGATTCTCAACAGCCACTCAGCTGGAGTCTGCCTAA

AAAGTTGGGGAGGGTTGTCATCATCACTGTGGCTGCCTGCTGCCTAAACC

CTCTGAGTTCCCTGGGGGAGGGGGAGCAGTCATCACTGTGGTTGCTGGCT

GCCTAAGACAACTGAGCTTCCCAAGAGAGGGGCAGTCATCATCACTGCAG

CTGCCTGCTGCCTGAGGAAACTGAGCTCCCCAAGAAGGGACAGCAGCCAT

CACTGTGGCTGCTAGCTGCCTAAGACACTGAACTCCTGGGGAGGAAGGGC

GGCAGCCATTTCTACAGAGCCAGGCTGCTGTTTTTCCTTTGCTGATGCCA

GGGAGACTGGACGGCTTGGTCCCAAGAGGTATTCCCCACAGCGCAGCATA

CTGGCTGTGGCAGATCATGGCCAGACTGCCTCTTTAGGCTGACCCTCACC

CATCCCTCCTCACTGGGTGGGGCATCCCTGCAGGAACTCCAGCAACTCAA

GCCAGGGAATTAGGGAGAGAACTCTGATCTCTCTAAGTCTGAGTCCCTAG

CAGGAGGGGTGGCTGGCTGTTGTCTCCACAAACCAGAAGACTTATTCTTT

CCCCCTGCTCACTCTGAGGAATCCAGGCATCCCAGACGAGTGGGATTTCC

CCCAGCACAGCATACCCCCTTCACAAAGGGACAACTAAAGTGCTTCATTA

AGCAAGTCCTGGATCCTGTGCCCCCCAACTGGGTGAGACACCCCAATGGG

TCACCAGACACCTTATACAAGAGCATTTCTACTGGCATCAGGTGGGTGCC

CCTCAAGGACAGAGATCCCAGAGGAAGGAGTGGGGTCTCATCTTTGCTGT

TCTCCAGCACTCTCTGGTGACATCTTCAGGTGTGGGAGGGACCCAGATAA

GTAGGGCTTGAAGTGAATCCCCAGCAAACTGCAGCAGCCCTACAGAAGAG

GTGCCTGACTGTTCAAAGGAAAACAGAAAGCAACAACAACATCAACCAAA

AAGTCCCCACGAAAACCTCATCTAAAGGTCAGCAGCCTCAAAGATCAAAA

TGAGACAAACTCATGAAGATGAGAAAGGAATGAAAAACCCCTCACAACTC

AAAAGGCCAGAGTGGCTTGTTTACTCCAAATGATCACAACACCTCTACAG

CAAGGGCACAGTGCTGGGAGGAGGTTGAGATGGATGAATTGACAGAAGTA

GGCTTCAGAAGGTGGGTAGTAGCAAACTTCACTGAGCTAAAGGAGCACGT

TCTAACCCAACACATTGGAACGAATCCCAGAACTTAAAGATTGGTTCTCT

AAAATAAGACAGACAAAAATAAAAAAGAATAAAACGGAAGGAACAAAACC

TCCAATAAGTATGGGGTTATGTATAGAGGCCAATTCTACAAATCACTGGC

ATCCCTGAAAGGGAGGTGGAGAAATCAATGCATTGGGTTAGAACATGCTC

CTTTAGCTCTGTGAAATTTGTTATTACCCACCTTCTGAACCCTACTTCTG

TCAGCAAAGAAGCTAAGAACCATGTTAAAAGGTTACAGAAGATGCTAACT

AGAATAACCAGTTTAGAGAGGAACATAAATGACCGGAGCCAACTATAAAG

CACATAAGGGGAACTTCGTGATGCAAACACAAGTATCAACAGCTGAATTG

ATCAAGCAGAAAAAAGAATATCAGAGCTTGAAGACTATCTTGCCAAAATA

AGGCAGGCAGACAAGATTAGAGAAAAAAGAATGAAAAGGAATGAACAAAA

CCTCAAAGAACTGTGGAACTATGTAAAAGACCAAACCTATGACTGCTTGG

ACTACCTGAAAGAGACAAGGAGAATGGAGCCACGTTGGGAAAACACACTT

CAAGATATCATCCAGGAGAACTTCCCCAACCTAGCAAGACAGGCCAACAT

TCAAATTCAGGAAATCCAGAGAACCCCAGTAAGATACTCCACAAGAAAAT

CAACCCTCAAGACACATAGTCATCAGATTCTCCAAGATCAAAATGAAGGA

AAAAATGTTAAAGGCAGCCAGAGACAAGGGCAGGTCACCTACAAGGGGAA

GCCCGTCAGACTAACCGTGGGCCTCTCAGCAGAAACCCTACAAGCCAGAA

GACAGTTGGGTCCAATGGTCAACATTCTTAGAGAAAAGAATTTCTAACCT

AGAATTTCATATCTGGCCAAACTAACCTTCATAAGTGAAGGAGAAATCCT

TCTCAGACAAGCAAATGCTGAGGGAATTTATCACCACCAGGCCTGCCTTG

CAAGACCTCCTGAAGGAAGCACTAAAGATGGAAAGGAATCAGAATGATGC

TGGCCTCATAAAATGAGTTAGGGAGGATTTCCTCTTTTTCTATTGAT

>hg19_ct_ARAlincRNAs_9727_ARAlincRNA_0306.1 range=chr2:101768122-101771872 5'pad=0 3'pad=0 strand=+ repeatMasking=none

GAACTCGGGAGAGCTGCCGTCCCTGACCTCGACCTGAGACGTGGAGGAGG

GACAGGGAAAAAGCCTTCTTCTCGCAGCTGCAGGTTGCCCCACCGAGGGC

ATTTTTCCGCACCGAGGGGGCGGGGGGCAGAATGCACGCCAGAAAACCTA

AGTGCGCTGGGTTTAAAATAGCAACAACAACAAAAATTACAAATAACCAA

AAAACAGCATAAGAATTAAAAACAGTTATATTCCAATGTTTTTAATACTT

GGTTCACATTTTGGATGCTCTCCCCAGGGATCCGACGGCGCTGCGGAGGC

GGCAGCGATGTTTGGGATCCTAAAGAAAGCATCAGACCAGACTCAGGTCT

GGACGTCCCTCAAGTGGCCCGGAGGTACCGATATTAGTGTCACCGCCGTC

AGTGCCCTGCCCAGAAATAAAACCTTTGTTCTGAGAGAAATTGAAAAGCT

GGAAAATATGGAGTAGGCGATGTGAAGAGAAACTACAGGGAATGTAGAGG

TCGGGTTTTAAATGCGTATTTCCTCAAGGATGTTGTGGAAGCAGAGACAA

GGTGGCTGGCCTGGAGCAGGCTGGAGGACCTCCAAGGATCTCCACAGGGA

GGCAGTTACATTTAACACCCTACCTAGCAAAAGAAGAAAAGAACAACCCC

ATGAAAACATCCCTGGGGTTTTAACAAAGGAATCCCTGACAAAACGTGAC

TGATAACTAGGACAGAACTGAAAAGTGGAAGTAAAGTCAATCCCGACCCC

CCAGGACGCCTGGGAACTCAGGGAAGCGCCTCCAGAAGGGAACAGCTCAG

AAACCCTGACCAGACCTGGCGGACAAGAACCGTGTGGGCGGGGGACAGGA

ACGCCCACCCAGAGGCCGGGAGAGCATGGTCCAGGACTGGCAGCCACTAA

CAGCCTCTGAATGAAACTGAGGAAGGAAGTCAATGGTAATCGGGACCATT

AGGGTGCACCAGGGCATATAGAACAGTCGGGGACGGTAAGAAAACATTCC

TTCCAGAGACATTCACTGGCACCTCCCGCGCAGACACACTGCCAGGCCCG

GGAATCGCAGAGATGAACTGGGAGACTTGTCTGCCCTGGAGAACTCACAG

CTGTTGAGCAGCAAATCTCTTCAACCTTTCGGAGATGGACTGCATTTCCT

ACCAAGACCCTGGCTGATAGCAGAGTGACCCCAGGAAACACAGTTCCTCA

ATTGTGGGATTCTGGCTGGGTTACCCACAGATTCGTGCGTGCATAGCGAA

CAAAAGGGATGCTAGTAACCCGTGGCATGGAGGGGCAGCAGGATTACTCC

AGTTAGCACTGGGAATAGTGTGAGGGGCATATGGACCACAAAGCTTTGGG

GGCTTTTGCTGCTGTTGCTCTTGATTGCTATGGCAACAGTAATAATTATA

AAGATTGTAGAGTGAAATGGATGTTTCTGACTGCCCTGGAGAGTTTTTAA

ACAATGGAAGAGATAAACATGAAGTATTAAATTCTTATTTTAAGGTACAG

ATATGCTGAAATCCAAAGAACCATTTATCTGGGATAGGCACAGGACAGAA

TAAGTTCATCTTCAGATACTAAATATAATTGTACAGATTACAGAATTGTG

GCAAAGGTTAAGTGCTCAACCTCAGCAAGTTCTCTTGCTAAGATTAGGAC

ACTGATTGGGAAGGAGTAGGACCATAGACTTGTGATAGGAACATTTGGAC

AGACACAGACAAAGCCGAATTCCCAAATCTCAAGGCTCCCTTGTATACAG

AAGCAGCCCCGCCTCCCCTGTGCAAGGAAATTAGCCTTCACATACACTAA

GAGCTTTCAAGACTTCACTGGGGCCTCTGCCTCCCACATGGATGCCTGTT

CTCCTCCAAACTTGCCCCTGATGCACCTCATTGACTCCAGGAGCATAACA

AGAATAAACTCTCTACAGAGCCCAGAAGGAGAAATGCAAGGTCAGCCAGA

GAAGAGGAGGATGGAAATAGATTATATACCACGAAAGCTGGAAAATCTTG

CCAATATGTGTTGCAGAGAAGCTGAGAACTGTGTATGGGAATGGATTCTA

AAGTGTTAGACTAAGGAGGATGAAATACTAGGTTAGGCCAGGCATGGTGG

CTCACACCTATATTCCCAGCACTTTGGAAGGCAGAGACAGGAGGATAGCT

TGAGGCCAGGAGTTCAGGACCAGCCTCGGCAACGCGGTGAGACACCGTCT

CTACAAAAAATGTAAAAATTAGCCACGTGTGGTGGTGCACGCGTGCCTCT

GGTCCCAGCTGCTCAAAGGCTGAGGTGGGAGGATCATTTGAACCTAGGAG

GTCAAGGCTGCAGAGACCTATAATGGCACCATTGCACTTCAGCCTGGTGA

CAGAGCAAGACCCTGTCTCGAATAAAATATAAAGTAAGATTCAGCAAAAT

TTATGAATATGCATACATTCCCCTGGGATTTATGATTTAGTGTGTTATAT

ATTGGAGTGATTAAAATGTTTTAGAC

>hg19_ct_ARAlincRNAs_9727_ARAlincRNA_0307.1 range=chr2:105865861-105866251 5'pad=0 3'pad=0 strand=- repeatMasking=none

TGCCGTGGCCACCAGCCTCCACCTTTCAACAGCCAGAGCATTGAAACTAC

CTTCTTTCCTTTAATGTAATCAAATTCTTGGGCAGAGTTGATCGTAATCA

GTGTTGACTGTTTGTTATTTTAAATAGTAATTTTTTTTCTTATCCTGGCA

AAGTTCATCTCCTTGTGGCTCCAGATTTTCAGTCTAGTTATTTTATGTCT

TCCACCATTTGTGTCTGAAAGGTTAAATGTGGTGCCTTTTTTTCCTTACC

GCATGCATAAATGCATGTATTTTCTGTATTTGGACGTAGAACTCTGTGTG

TTGGGGGCTGGGATAAAGAAAGAGGCAACCATCTTTGGCTGATGTGTTAG

AGATATGCTTACTTTACAATAAACAAAGGCTCTTTGTGAAA

>hg19_ct_ARAlincRNAs_9727_ARAlincRNA_0308.1 range=chr2:120030214-120039443 5'pad=0 3'pad=0 strand=- repeatMasking=none

GTCTTCTGGTTGAGTGAGGAAGAGGTTTCAGTTTGGTGATGGCAACGTCC

TTAAGACTTCACTTGCTTCCCTCTCTCTGTTCTTTTGAGAGCGGGTCTCC

ACCAAGCATTTTAGCAGGGTCTCACTCTGTCACCCCAGGCTGGAGTGCAG

TGGCAGGATGGCAGCTCACTGCAGCCTCGACCACCCTGGCTCAAGCTATC

CTCCCACCTCAGCCTAAGAGAAACTCTTGGAACGGAAAACCAAATACCAC

ATATCCTCAGTTATAAGTGGGAGCTAAATGATGAGAACACGTGGACACAA

AGAGGGGAACAGCAGACACCGAGGCCTACTTGAGGGTGGAAGTTGGGAGG

AGGGAGAGGAGCAGAAAAGATAACTTTTGGGTACTGGGCTTAATACCCGG

GTGATGAAATAATCTGTACAACAAACCCCCATGAGATGAGTTTTCCTGTG

TAACACCGAACCTAAAATAAAAGCTAAAAATAAAATTAAGTTAAAAGAGA

AGCTGTCATTTTTCTCATAACCTAATTACAAACCTATGTGGTAAGAACTA

TAATCACCCTCATGGTCTCACT

>hg19_ct_ARAlincRNAs_9727_ARAlincRNA_0308.2 range=chr2:120038723-120040231 5'pad=0 3'pad=0 strand=- repeatMasking=none

CCGGGGTGCTCACCCTGTCCGTCAGGGCCGGCAGAGACGGACGCTTTACA

GGTGTTTGTTAACCTGGACCATGCTGGACGGGAGGCTTGGAGGCACCAAG

ATGCCAAATCTCTTACCCATCCCTCCAGCCAGGCGTCTCAAGTGGCTAAA

GAATAAATGCATGAAAATTAAGAGCCAGCTCTGCCTGCGAAAGAGAACAG

GAGCAAGCTGGCTGTTGGCTGTCATCTGGAGTCACCTACTGGGAAGTGCC

GAATCACCCCAGACCAAGGACAGGAAGGAGGCTTGGTCTTCTGGTTGAGT

GAGGAAGAGGTTTCAGTTTGGTGATGGCAACGTCCTTAAGACTTCACTTG

CTTCCCTCTCTCTGTTCTTTTGAGAGCGGGTCTCCACCAAGCATTTTAGC

AGGGTCTCACTCTGTCACCCCAGGCTGGAGTGCAGTGGCAGGATGGCAGC

TCACTGCAGCCTCGACCACCCTGGCTCAAGCTATCCTCCCACCTCAGCCT

AAGAGAAACTCTTGGAACGGAAAACCAAATACCACATATCCTCAGTTATA

AGTGGGAGCTAAATGATGAGAACACGTGGACACAAAGAGGGGAACAGCAG

ACACCGAGGCCTACTTGAGGGTGGAAGTTGGGAGGAGGGAGAGGAGCAGA

AAAGATAACTTTTGGGTACTGGGCTTAATACCCGGGTGATGAAATAATCT

GTACAACAAACCCCCATGAGATGAGTTTTCCTGTGTAACACCGAACCTAA

AATAAAA

>hg19_ct_ARAlincRNAs_9727_ARAlincRNA_0308.3 range=chr2:120039327-120040100 5'pad=0 3'pad=0 strand=- repeatMasking=none

GGCGTCTCAAGTGGCTAAAGAATAAATGCATGAAAATTAAGAGCCAGCTC

TGCCTGCGAAAGAGAACAGGAGCAAGCTGGCTGTTGGCTGTCATCTGGAG

TCACCTACTGGGAAGTGCCGAATCACCCCAGACCAAGGACAGGAAGGAGG

CTTGTTTCCAGAACATGTACAACATGCTCCTAGTGGGCCTGGGGATGGTT

GAGGGTGGGTAGCGCAGTAAGACAGTTACTGTTACTTCAGTTTTCTTTCA

GGTCTTCTGGTTGAGTGAGGAAGAGGTTTCAGTTTGGTGATGGCAACGTC

CTTAAGACTTCACTTGCTTCCCTCTCTCTGTTCTTTTGAGAGCGGGTCTC

CACCAAGCATTTTAGCAG

>hg19_ct_ARAlincRNAs_9727_ARAlincRNA_0309.1 range=chr2:120446872-120448187 5'pad=0 3'pad=0 strand=+ repeatMasking=none

TACCGAAGAGACTGACAAGTACACCTGCAGTGACAACACGTGAGTTCAGT

GCTGTGAAGATGTGTGAGGGAGCATAGGAGGAGGGCAGCCAACTCAGCTG

GGATGGGAGGTAGTCAGGGAGGACTTCCCAGGAGAGGTGACCCTGTTGCT

GAAACCCCTACGAGTGAATGGAAGGAAGTCAACAACAATAATACCAAACA

CTTATTTAACACTTATTTTGTGCCAAGTGCTTTACATATAAGAACTCATA

AAATTTCCACAACAATCCTATTATCCCCATCTTACAGGTAGGGAAATCAA

GGCATAAAGAAGTCGAATGGCTTCTCACCAGCCAGTGCTGGGCTTGGGAA

CTGAACCCAGGCAGTCTGGCTCTAATGCCTGTGCCCTTAACCACTCCACC

TTCACTTAGGCAAGTGAAACTGGCCATGGGAACAGCCTGTACCAAGCCAG

GAATGAGACAGTTTATGTAGGCTTTATCAGGGTCCAACCAAGAGATTGAA

ACCACACTGTAATGTGAACAGAGAGAATTTAATATAAAGGATTATTATTC

TATAAGGAATATTATTACTATTATAACAACAGAGGGTTGGAGTGATGAGG

ACTGGCTTGTAAGAAGTCCAGAGCCACCTGGACTCAGAGCCACCCTAGCT

TTGGATTTCTTCCAGTCTCAGCCCCCTAGAGCCCTTGCTCCCCAAAGAGC

CTTGCTCCCCGCTAGAGGTCAAAGGTAAGTCATAGCAATGGACCCAGACT

CTTGTTATTACCCATGACATGGTTTGAATCTGTGTCCCCAACCAAATCTC

ATGTTGAATTGTAATCCCCAGTGTTGGAGGTGGGGCCTGGTGGGAGGTGA

TTGGATCGTGGGGGTGGCTTCTAATGGTTTAGCACTGTCCCCCTGGTGCT

GTCTCATGAGTTCTCACGAAATCTGGTTATTTAAAAGTATGTGGCACTTT

CCCCTTCGCTCTCTCCTTCCTGCTCCAGTCGTGTAGGACGTGCCTGCTTC

CCCTTCACCTTCCGCCATGTTTGTAAGTTTCCCGAGGCCTCCCCAGAAGC

CGTCATGCTTCCTGTACAGCCTGTGGAACCATGAGCCAGTTAAACCTCTT

TTATTTATAAATTTACCCAGTCTCAGGTAGTTCTTTATAACAATGCGAGA

ATGAACTAATACAGCCCACCCCTACCCCATACCTCTCCTACCCTCTCCCC

AGCAGGCTGTGAGGACAGGGTGCTCATGCCAAACCCTGAAAGCATTACAA

AGGCTGCAGGGCCTAAGCTGGGGCCTGGGCCTGAGGAGCAAACTGAGGAG

CAGAGCCCACGTGGAG

>hg19_ct_ARAlincRNAs_9727_ARAlincRNA_0310.12 range=chr2:130748029-130785985 5'pad=0 3'pad=0 strand=- repeatMasking=none

TTCACCACCAGCAACTTCAGAACCCAGTACTGGAATGCCATCAGCCAGCA

GGCCCCTGCCATCATCTATGACTTCTATCTGTGGCTCACTGGAAGGAAAC

CCAGGTGGTTAGATAACGCTGTTATTGACGAGATCACACCCAAGCTGATC

AGAGATCTGCCCAATTCTTGCACCTACCGCAAGGCCT

>hg19_ct_ARAlincRNAs_9727_ARAlincRNA_0310.13 range=chr2:130748029-130799113 5'pad=0 3'pad=0 strand=- repeatMasking=none

TCTCAATCTCCTGACCTCGTGATCTGCCTGCCATGGCCTCACAAAATGCT

GGGATTACAGGCATGAGCCACTGTGCCCGGCTGGTTCTGACATATTTTTA

AGAAAGTTTAGGCTGATGCTATGGTAACTCAGTTATTGATGATACATGTC

ATCTTGTAAGTAGGAGTATTCAATATTCATGGTTTTGAAGCAATTTCAGA

GTACTATAATCACCACACTTGACAGACTTCAGAATTCATTGAAAATCTCT

GGAAATAATTAGCAATTTGTTATCATAGACTCTAAACAGACCTTCAAAAT

GCATTACATAGATTTGGTAAAACAGATTGTCAGTCTGCCAAAAGTAATCA

CTCTGGTTTGATTTGCATTGAATGAAAATTTTAAAATGTAAAGCTAAACT

CTTCAGGATCACTGCACTGGGTTTCTATAGGACAACACTGAACAGGGCTG

CCACTGAAACACCTTGGATGAGAGCCTCAAACTCCCTGAACACAGCTTCC

AGCACACTACTGGGAGAATGATTGGGACTCAAAAATATGTGCAATGAATG

TACTTACGACCTTGTTGGAAAGGAAGTAAAGAGGTTGTATGTAATTACCT

CAAATTAATACTGCTACAGATAAAGTAGTAATTAGCACTGACAAAGTAAT

AGTAGCGCTAAAGTAGTATTAATAGCCCTACACTAGTAGTAGTAATAAAG

TGGCATCGCTATTGATTCTATCCGTCTACCTACCTACCTGTCTGGATAGG

AGGGCATAGGATTTGACTGTAAGATTGGGCTTAGCAGTGAAAACAAAAGA

GCTCATGCATGCTTTGGACAGGTATGCACTCAAACTTGGTATCATGTGAC

TGTTCTGAATTCCAGAACCAAGGCTAAAAGGTGGAAGTTTTGTTTGACTG

TGTCTCACATTTTCCTTTTGGTCTATTGTCTAACACATGTTGCTAGAATT

CTTTTTATTCTGACTACCTTTTATGTATAGACAACGCCATTATTGACGAG

ATCACTCCCAAGCGGATTGGAGATTGTCCCAATACTTAGACCTATAGCAA

GGCCTTGGGAGAAATGGTGGTGCAGCAGGAGAGCAGGAACCTAACCATTG

CCATCCTAAGGCCCTCCATTGTGCGGAGCAACGTCGCACCAGCTTTTCCT

GGGTTGGGTTGATAATCTAAATGGATGTAGCTGACTCATTATTGCGGCGG

TACGGGCTGGTGAAAGTGGAGATGGACGGCAGGATGGATTCACTTGGCCA

CATGGCGCGAAGCTGGGAAGACGGACACCGGTGAGTGGCTGCCCGGGAGG

GCTGGTCGGGGCGCGGACAGGCGGGCATGGTTCTGCCAAGGATTTTGCTT

TATTTATCGCAAGATGGGGTATTTCCTCCTTTCTTCAGTTTATAATTGCA

TGAATTAGTGCAGTGAATTGAGGATGCAGTAAAAATATCTTCAAAGATTA

TTAAATTCGTTATTATAAAACACATAGAAGAGTTTATGTGTGTATATGGA

AAGCAGGTATACATCAATAATTCTTAATGAATACAAGAAAGAACTACCAA

TATTGGGGCAAATTTTTCAAATACAAACATCAGTGAATATAGGCAAGGCC

TTTTCTTTTTTATTATTCTATTTTAAGTTCTAGGGTACATGTGCACAACG

TGCAGGTTTGTTACATAGGTAGACATGTGCCGGCAACGCCTTTTCAATAA

TGTCTTACAAGGAGAAACGTGGCTCCTCTAGGAACGTGAGGAACACGAGT

AGTTCAAGAAAATGCTGAAGATGCGCTTGGCTGGGAAGCTAGGATAGGCT

GACGAAGTCAGACTTAGTGTGGGAACCTGTTAAGCAGTGGATCGATGTTA

GCTGTTTTCACTTATTGCACTAGTATAATCAGTTTGATGATACATACTTT

AGCCCCAGCTGTTACTTAAGACCTGGTTTCACATTTTATGTTCTTTTTCC

TGTGCAGGCTGGGAAAGGGTTTCTTCTGTCCATAAAAGCTACTCCAATGG

CTGTGGGAGACTTAATTCCAATTCCAGGTGATACAGCCGTCAATCTCCCA

CTAGCTGTAGGATGGTGTGTGTGCTGCAGTTCACAGGGCAAAGGAGATAG

AAGTGGATGAAATGAGAGAATTTTCTTTAATTGAACTCTGGTTAATGCCA

AAAGTGTTCAATCACTATGTGTGGGGAAGTTTCCTGGTACAAAGGAAAAA

AAAACAGACCTAAGTCAGTGTTAGTCTACCACTGTACATCTGGTAACCTC

AATCCCTGCAACCGGGGCAAAATGGGTTTCCAGGTCTTGGCAACCTTTGA

AATTCCAATTCCATTTGAGAGAGCTTTGACGAGGCCATATGCTGATTTCA

CCACCAGCAACTTCAGAACCCAGTACTGGAATGCCATCAGCCAGCAGGCC

CCTGCCATCATCTATGACTTCTATCTGTGGCTCACTGGAAGGAAACCCAG

GTGGTTAGATAACGCTGTTATTGACGAGATCACACCCAAGCTGATCAGAG

ATCTGCCCAATTCTTGCACCTACCGCAAGGCCT

>hg19_ct_ARAlincRNAs_9727_ARAlincRNA_0310.11 range=chr2:130748029-130808704 5'pad=0 3'pad=0 strand=- repeatMasking=none

GCGGTTGTGGCTCAGGGGTCAGCTCCTGCTAGTGCCAGGACACTACTGGG

AGGCTGGGACCCGACCAAAGCCCATGGTGTTTCTGGCCTGAGAACAAGGT

GTCTTGGGACCATAAGGCCAGGCCACCAATGGCCATTGGGTCATAGGGGC

TCAGCCCCAATCTTTGTCTTTCCCTGGCTCCTTCTGATTCAGTCCCATCA

GGGCCCTGGATCCCAAGACTCAGCATCCAAGGTCCCCTCCAGGAATCCTG

GCAGCTCAGCATACTTTATCCTGTTTCATCTGAGAGCAAAAATGTAAAAT

TGGATGCACAGAAAAGTGACTCAAAGTGCTTAATGACTAGAAGAAATCTA

GGAGCAGCAAGAAGGTAATGTGGAGGGAGGGACCTCCATGACCGGTGTCT

GCAGAGCCAGGGGTACAGGCACCCAGTGCTGTGGCCTGGCACCACCTGCC

TCTCAGAGGGTGGGTGGCACACTCCTTAACCAGAGGACAGCAGGCCTGGT

CACCAGCTTTTCTACCTGTCCCTGTAAGCATCACATTGCTGGAGGAAAAT

CTCATGCCAGAGCTTGGACCATCCCTAGCTCGGGTTAGGGGTTGTCCCTT

GGTGACCTAAATGAAAAAACAGGTCCAGAACAGAGTTCCTGATGCTGGAC

ACTCATTCAGTCTTTGAATCGTGGGAGGGGAGGCCTGGTACTAGGTAGAC

CTAACCTCTTTGAGGAACCACAGAGCCCAAGGCTGGAAACCTCCAGAATC

CTCCACCCCCTGATCCTCCCTGGGGACCCCTGTGGCCTGTCTCACTGAGC

ACTCTTCCATCTGTAGATGTCTGGGCTGCTGTACAAGGGAGTCCCCTTTC

AGGTGTGGTGCTAGACATGGTCACTCCTGCTGGATGTCTAGGTGGTAGAA

ACCAAGGACCTAGGGAAATACCAGCTACAGCCTTTCCCCGCTCATCCAGA

GCAGGACAAACAGGCCAGGCGGTGTCAGGAGCCCAGGTCTCCAGCTGGAG

GGAACGTCAACCCTGCGGTGGGAGTAGGGGCCCTTTGCACATCCTAGGCA

CAGATGGTAATGTAGACACCACAGGTAAGCTGGGCTTGGTACCTACCCCT

CCCCGGATTCAGAAAGAAACCAAACAAGGAGCTTTGTGCGGAATGAAACC

TCCTTTCCTCCCAGAAGCACTGCTGACTGTTTGGTGGTTGCCATTTGTGG

CAGTGAGCCTTTGTTTGTTCTGAGGTTGGGCTGGTTTCTCCTCTTGGTCC

TGCCCTACAGATCATAAAGGAGAACAGCAAGAGGTCCCCAGCAAACATCC

ACAGATGGCCTTGGAACCTCACCCTGCAGGAATGCCAGTGAACATACTGC

TGACATCTTGGAGCTCAGTACCCTCATAGTGTAACGGCGTCAGTAGATCT

GCCTGTGCTTGGACTTCCTGTACTACCCATTCCTGAGGGGCGATGCTTCT

GCAGGGCCTGTGACTTGGTGCACAACTTCAGACACCATCATCTTGCAGCA

GCACCGCACCCTCACTAGCCAGGGTGTTGATGACTTCCTCAAGGCCAAGG

CCACATTCAAGGCTTCGGACTTTATTGATGCGCTTGTGCTGAGCAAGGTG

GCTTCTCCAGGATCTTAATTCAGGAGGTAGAATGGAGCTTGAGATCAAGT

GTCTGATCAAGCCTCAGTGTATGGGCGCTGTTCATCCTCTGGTGCTGAAG

CAGCCAAGAGACCCAAGTCTGCCTGGCTGCCTCTTAGGATATGACAGCAG

AGCCAGTGGCCTCTACTAGATCCTGTACAACCTCACAAAACACCCAGACA

TCGGGAGTGCTGCCAGCCTGTGATGCAAGAGTCCTAATCCTGAAGACATT

GAATGACCTGTCATTCTGCTGTTTTTACCAAAAAGGATCATGAGGATCAG

AGAGGAAAAGTCACTTGCCCAAAGTCACACAGCTGAACAGTGGTGGAGTT

CAACTTTGACCATGGGCTGTCTGGCCCCAAGGTGTATGCTTGCTTCTCTC

CCAAGAGACTCCTTTCTTATCAGGCTCAAATGAATGAAAGGAGGATGTTA

AAGACAACGCCATTATTGACGAGATCACTCCCAAGCGGATTGGAGATTGT

CCCAATACTTAGACCTATAGCAAGGCCTTGGGAGAAATGGTGGTGCAGCA

GGAGAGCAGGAACCTAACCATTGCCATCCTAAGGCCCTCCATTGTGCGGA

GCAACGTCGCACCAGCTTTTCCTGACCTAAGTCAGTGTTAGTCTACCACT

GTACATCTGGTAACCTCAATCCCTGCAACCGGGGCAAAATGGTTAATTCA

TAATATTTGTGTGTCTTCTCTTTTCTTCTCAGCTAGTCTCCAAAGGGTTT

ATTTTATTTCTTTTTTTGAAAGAACCAACTTCTTCAGGCTTTATTGACTT

TTTTCTTTGTTCTTTCTTTCTTTTTTTTTTTTTTTTTGAGATGGAGTCTC

GCTCTTGTTGCCCAGGCTGGAGTACAATGGCATTTTCTTGGCTCACCACG

ACCTCGGCCTCCTGGGTTGAAGCAGTTCTCCTGCCTCAGCCTCCTGAGTA

GAGTAGCTGGGATTACAGACATGCGCCACCACACCTGGCTAATTTTGTAT

TTTTAGTAGAGATGGGGTTTCTCCACGTTGGTCAGGCTGGTCTTGAACTC

CCGACCTCAGGTGATCCACCCACCTTGGCCTCCAAAAGTGCTGGGATTAT

AGGCATGAGCCACCGCGCCCAGCCTATTGACTCTTTATTATGCTGCTTTC

TTTTCTATTGCATAGATGTATTGCCTTATCTCTTTTTTCCCCTTCCTCCT

GCTTTCTTTGATTATGTTTTTCTTCTCTTTAACTTAGAAATATAGCCCAT

GAATATTTGGCTTGTCTAATACATCATTTATGGCTTCATATTTCCCTCAA

GTGCCACTTTGGCTGTATCCCACACATTTTGTTGTATAATGTTTATGTTA

TCATTACTCTCTAATCCTTCAACATTTTTCATTTGAATTTCTTCTTTGAC

CTATGAGGATGTAGAGTGCAGTTTCCAATTGCCAAATTACATTTTCTACA

TGTGCATGAGTTTCTCCTTGTCCCTCACATCAGACAGACATTAGCTTAAT

GGTTAAAAGTCTTGGGCTTTGAGATCTAATAGACTTGGATTCAAATCATA

GCTCATTCACTCACTCCTGTATTTATTAAATAGACATTATTCTTGGTGCT

GAGGATATATCACTAAACAGAAATCCATGCTCACATAGATGTTATATGCT

GATATGCGTCCCACCATTTAGTAGACTCCACTCTTATTGTATCAGTTGGA

AAAAGTCTCTCAGTTTCTCCCTGTAGATGAATTTTTTTATCTGTCAGTGA

GAATAATAATTATTCTATTCTAGGGAGCTCTTGGGAGAACGTAGTGAGAC

AAAGTGCTTAGTACAGTGGTTAGAACAGTGAACATTTTAGTAAATGCTTG

CTATTACCATATGGACAGCTTTCTTAGAAAGCCTTTCCAAACCTAGCCCC

ACCCCTCCCCTTCCTGGCACCCAGACAGAGCTAGATGCTTCTTTTGTATT

CCTCCAGAGCATATTGTACTGGCCTCTAGCAGAATACTCAGGCAAAATAT

TGTAATCATGAATTGTCCACATCTCCCACCAGACTGCCAGTTCTTTAAAG

ACAAAGAATATATCATATGTAATTTTTTTTTACCTCAGCATCTACCACAG

TGCCTGGCAAGTGTTAAATGCTAAATAAATATATTTGAAATGACTAAAGG

AAGGATTGATACAGATTTAGGAAAAGGAAAGTAAGTGTATGTTGCTGAGA

TATCTATTGATTCGTTTAGTCAAAAAATGTTTATTAGGCACTGATTACCT

TCAGCCACTTTTCAAGGTACTGTATAGGTTCAAACAAAACAGACAAAATT

TTGTTATGATACTTAAATCTTAGAAAGGAGAAATATACATCAGATATGGT

TGAGTGATTGCAGAAAAATAAAACAGATTAAAGAAGTAAACAGTATTGTG

GGAAGGCTGCTATTTAATATGTGATGGCCAAAGAAAGTCTCTCTGATAAG

GTGTCATCTTAGTAGAGACCTGAAAGAAGTGAGAGGGCAAATCAGAGGGA

CACGTGGATGAAATATTCCAAGTTGAAGAAATAGCAAGTGCTGAAGTTCT

TTAATCATATTCAAGGACAGCAAGGAGGCCAGTGTGGCTGCAGCAGAAGC

ACCGAGAGGGAGAGCGGTAAGAGGTGAAGTCAGAGTGGTGGGCAGAGAGA

GTGAGGATGCAGAGAGTGCAAACCCTTGCAGACCATGGCTTTGCCTCTAA

ATGAGAAGAGATTCCGCTGAAGGGCTCTGAATGGGGACGTTCCTGTAATA

CACTCTGAGTTACATTTTACAAGAATCCCTCTGGCTGCTACATGGAGGTC

CGACTAGGGTTACAGGGGTAGTGGCAGAGACCACTTAGGACAGTATTGCA

ACATTCCAGGCTACAGTAGTGGTTTGGATCAGAGAGATAGCAGGTGGTAA

TTATGATTCTGGGTATTTTTCAAACAGAGCTGATAGATTTTGTTCAATTG

TGTGTGGGTTGTTAAAGAAAGAGAAGAATCAAGGATAACCCCAAAGTTTT

TGGCTGAACTACTAAACAAATTGAATTAGCATTTGGTTAGAGGTAGAAGA

CTGGTGAAATAGCAAGTTTGTGTATGTATTGGGAGGGGGGTGGTTAGCAG

TGGGTGTATCAGGAGGCCCATTTTAGAGCTGTTAAATGTGAATGTCTCTT

ACACCTCCAGGTGGAAATGTTGAATAAGTATATGGATATATAGATCAGAA

CATCAGTGTTGAGATCAGAATCTCTTATGCTATCAACCTGTACATAACTT

TCTTATTTACCACCATCTTTTTAAAGTATAAATCCCTATGGACCAGATGA

TTCTTGAGAATCTTCTCTGAAGAGGAGTTATATTTTTCAGATAGATTTTT

AGATTTTAAAAAAATGCCAATTTTTTTAAGTAGGGCAATAATCGTATTAC

TATAATAACTGACATCATAATTGAATAATGAGCTTAGGCATATGCTTTGC

AAAAACCTACATACCTTAAATAAAATTGAATTCAAATGAGTATCTTTTCA

AAAGAACTGCTGAATTCACAAATGTATGTAAACAGTTCTTAGTTCAGCTT

AATCTAAGTATTAATATAAAAACACTACTATGGATAAAACCCTGGATACC

AGAGTGGACAGTTGAGTCTTACATATTCCCAGAGAAATATTTAAAGTAAG

CTAACACAAGAAAATCTTGCCTTTAGAATTTTTAAGTTAAACTTTTAATA

AAGATTCAAATAAAATATTAAGAAACTTTCTAAATGGTTCTGACAGTAAT

GGTCCAGCCAGGGAAATAAAGTTTCTAGCCTCTTTCTTGGGGCTTTCAGA

ATAGACTAGATAACCTTAGAATTAAATGTGGATTTATGTTTTTCTGAACA

GTACCCCCTTTTATTGACTACATGTTTCCCCAAGAATTTGTTTTAGAGTA

GATGTTTCAAATAAAGGGTCCAATTCTTAACCCATTCACATTGTCTCATT

TTGCAGATAACCACTTGCCTAGCATAAAAATCTTTACGTTGTTCTCAGAA

ATTGATTCTTGACTCTACACAGCAAATCCTTCGTCTTTATTGCTGTGACC

CATTATAAATACCTTCCACTATGATGGGCTTATATTCTCCTATTCTCCTC

TAGCACTTACTCTCTTTTTTCCCCCAGGTTTCCAGGTCTTGGCAACCTTT

GAAATTCCAATTCCATTTGAGAGAGCTTTGACGAGGCCATATGCTGATTT

CACCACCAGCAACTTCAGAACCCAGTACTGGAATGCCATCAGCCAGCAGG

CCCCTGCCATCATCTATGACTTCTATCTGTGGCTCACTGGAAGGAAACCC

AGCTACCGAAGGAAGATACCCTCATCAACCCAATTTTACAAATGGAGAAA

TAGAAGTTAAGGGAAGAATCTGAAGTAGTCTCAAAGGCAGTGACAGGAAG

GATGTGGAGAAAGCTGAGTGTCAAAGTCAGTATTCAGGACCGGCTTTACT

GCTACTTAGAGATGAATGAAGAAATCAGAGGGAACGCAGTGTGCTGATGC

TAAAGCAGCTGTCACCACCCAGCTGTGTGACATAGGACATATTCTTTCTC

TGTCTCACTTGACTAATATGATATGTCAGAGGAGACATGATTGTAATTGC

CTAAAGCAATTCTTGTGATCAAGACTCAGAAGCACGAACAGTATTGCCCT

CTGTGTTAGCCCCTTTATAAGGGAGGATATCATCTTCAGCATGCTGAATT

GTCATCTTTCTTAGCAGTGCAAATGACTAAAACTTAGCCAATGTAGAGTT

TGTCCAAATTTGGAGCTCATAACTCAGTTCTTGAGCAAAGTGAAAAGAAA

ACATTGTGATTATGGGGAAAATATTTGACGGGACTTATCAAATAAAGATA

GGAAAAGAAGAAAACCCAAATATTATAGGCAGAAATGCTAAAGGTTTTAA

AATATGTCAGGATTGGAAGAAGGCATGGATAAAGAACAAAGTTCAGTTAG

GAAAGAGAAACACAGAAGGAAGAGACACAATAAAAGTCATTATGTGCAAG

GCCAAGATGGCTCAAAGAGCAACCAGCCACCTCTGCAGCCTGCCACCTCC

TGCTGGCAAGATTTGTTTTTGCATCCTGTGAAGAGCCAAGGAGGCACCAG

GGCATAAGTCTACTCACTTATATCTGTCTGGAACATAACGCTTGTTTGTT

TTTACAACAAATAAAATTGATCTTGAATGTGGTTAGATAACGCTGTTATT

GACGAGATCACACCCAAGCTGATCAGAGATCTGCCCAATTCTTGCACCTA

CCGCAAGGCCT

>hg19_ct_ARAlincRNAs_9727_ARAlincRNA_0310.18 range=chr2:130748029-130808704 5'pad=0 3'pad=0 strand=- repeatMasking=none

GCGGTTGTGGCTCAGGGGTCAGCTCCTGCTAGTGCCAGGACACTACTGGG

AGGCTGGGACCCGACCAAAGCCCATGGTGTTTCTGGCCTGAGAACAAGGT

GTCTTGGGACCATAAGGCCAGGCCACCAATGGCCATTGGGTCATAGGGGC

TCAGCCCCAATCTTTGTCTTTCCCTGGCTCCTTCTGATTCAGTCCCATCA

GGGCCCTGGATCCCAAGACTCAGCATCCAAGGTCCCCTCCAGGAATCCTG

GCAGCTCAGCATACTTTATCCTGTTTCATCTGAGAGCAAAAATGTAAAAT

TGGATGCACAGAAAAGTGACTCAAAGTGCTTAATGACTAGAAGAAATCTA

GGAGCAGCAAGAAGGTAATGTGGAGGGAGGGACCTCCATGACCGGTGTCT

GCAGAGCCAGGGGTACAGGCACCCAGTGCTGTGGCCTGGCACCACCTGCC

TCTCAGAGGGTGGGTGGCACACTCCTTAACCAGAGGACAGCAGGCCTGGT

CACCAGCTTTTCTACCTGTCCCTGTAAGCATCACATTGCTGGAGGAAAAT

CTCATGCCAGAGCTTGGACCATCCCTAGCTCGGGTTAGGGGTTGTCCCTT

GGTGACCTAAATGAAAAAACAGGTCCAGAACAGAGTTCCTGATGCTGGAC

ACTCATTCAGTCTTTGAATCGTGGGAGGGGAGGCCTGGTACTAGGTAGAC

CTAACCTCTTTGAGGAACCACAGAGCCCAAGGCTGGAAACCTCCAGAATC

CTCCACCCCCTGATCCTCCCTGGGGACCCCTGTGGCCTGTCTCACTGAGC

ACTCTTCCATCTGTAGATGTCTGGGCTGCTGTACAAGGGAGTCCCCTTTC

AGGTGTGGTGCTAGACATGGTCACTCCTGCTGGATGTCTAGGTGGTAGAA

ACCAAGGACCTAGGGAAATACCAGCTACAGCCTTTCCCCGCTCATCCAGA

GCAGGACAAACAGGCCAGGCGGTGTCAGGAGCCCAGGTCTCCAGCTGGAG

GGAACGTCAACCCTGCGGTGGGAGTAGGGGCCCTTTGCACATCCTAGGCA

CAGATGGTAATGTAGACACCACAGGTAAGCTGGGCTTGGTACCTACCCCT

CCCCGGATTCAGAAAGAAACCAAACAAGGAGCTTTGTGCGGAATGAAACC

TCCTTTCCTCCCAGAAGCACTGCTGACTGTTTGGTGGTTGCCATTTGTGG

CAGTGAGCCTTTGTTTGTTCTGAGGTTGGGCTGGTTTCTCCTCTTGGTCC

TGCCCTACAGATCATAAAGGAGAACAGCAAGAGGTCCCCAGCAAACATCC

ACAGATGGCCTTGGAACCTCACCCTGCAGGAATGCCAGTGAACATACTGC

TGACATCTTGGAGCTCAGTACCCTCATAGTGTAACGGCGTCAGTAGATCT

GCCTGTGCTTGGACTTCCTGTACTACCCATTCCTGAGGGGCGATGCTTCT

GCAGGGCCTGTGACTTGGTGCACAACTTCAGACACCATCATCTTGCAGCA

GCACCGCACCCTCACTAGCCAGGGTGTTGATGACTTCCTCAAGGCCAAGG

CCACATTCAAGGCTTCGGACTTTATTGATGCGCTTGTGCTGAGCAAGGTG

GTTAGATAACGCTGTTATTGACGAGATCACACCCAAGCTGATCAGAGATC

TGCCCAATTCTTGCACCTACCGCAAGGCCT

>hg19_ct_ARAlincRNAs_9727_ARAlincRNA_0310.3 range=chr2:130769638-130803153 5'pad=0 3'pad=0 strand=- repeatMasking=none

TTCCTAGAAGCTTTAATTGAATGAAAGTTCCTAGTAGATCTGTACCTACT

AAAAACCACACTTCTGAAGCTACGTGGCCACCAGAAGACACAGCTAGTCT

GCCATGTAAAAAAGGAAAGGTGGCGTGTGCCCTGAAGGCGCAGGGGTGAG

AGGCAGGGAAATGGAGACCCCAACAGCCAGCATCAGTGGCCCTCATCACA

GCCCTCCAGGAGATATCAAAGGAGGCTGGGAAAGGGTTTCTTCTGTCCAT

AAAAGCTACTCCAATGGCTGTGGGAGACTTAATTCCAATTCCAGGTGATA

CAGCCGTCAATCTCCCACTAGCTGTAGGATGGTGTGTGTGCTGCAGTTCA

CAGGTTTCCAGGTCTTGGCAACCTTTGAAATTCCAATTCCATTTGAGAGA

GCTTTGACGAGGCCATATGCTGATTTCACCACCAGCAACTTCAGAACCCA

GTACTGGAATGCCATCAGCCAGCAGGCCCCTGCCATCATCTATGACTTCT

ATCTGTGGCTCACTGGAAGGAAACCCAGTTTTTCTCAAAATCTGAGGAGC

TGCTACGGAAAGCACACCAGATCTCATGTGTTGAAACGTGAACATGCTCA

CCACAGACCAAGGACGTTCAACCTGTCAATCCCCAGGATTTCCAAGCCAG

GGGTTTCATCTCTTACTTGTGGAAAATTTGTTCATTTGATTATGAACCAA

TCAGCTTTTCATTGTGTCAAAATCAAA

>hg19_ct_ARAlincRNAs_9727_ARAlincRNA_0310.9 range=chr2:130783566-130791950 5'pad=0 3'pad=0 strand=- repeatMasking=none

ACCTAAGTCAGTGTTAGTCTACCACTGTACATCTGGTAACCTCAATCCCT

GCAACCGGGGCAAAATGGGTTTCCAGGTCTTGGCAACCTTTGAAATTCCA

ATTCCATTTGAGAGAGCTTTGACGAGGCCATATGCTGATTTCACCACCAG

CAACTTCAGAACCCAGTACTGGAATGCCATCAGCCAGCAGGCCCCTGCCA

TCATCTATGACTTCTATCTGTGGCTCACTGGAAGGAAACCCAGCTACCGA

AGGAAGATACCCTCATCAACCCAATTTTACAAATGGAGAAATAGAAGTTA

AGGGAAGAATCTGAAGTAGTCTCAAAGGCAGTGACAGGAAGGATGTGGAG

AAAGCTGAGTGTCAAAGTCAGTATTCAGGACCGGCTTTACTGCTACTTAG

AGATGAATGAAGAAATCAGAGGGAACGCAGTGTGCTGATGCTAAAGCAGC

TGTCACCACCCAGCTGTGTGACATAGGACATATTCTTTCTCTGTCTCACT

TGACTAATATGATATGTCAGAGGAGACATGATTGTAATTGCCTAAAGCAA

TTCTTGTGATCAAGACTCAGAAGCACGAACAGTATTGCCCTCTGTGTTAG

CCCCTTTATAAGGGAGGATATCATCTTCAGCATGCTGAATTGTCATCTTT

CTTAGCAGTGCAAATGACTAAAACTTAGCCAATGTAGAGTTTGTCCAAAT

TTGGAGCTCATAACTCAGTTCTTGAGCAAAGTGAAAAGAAAACATTGTGA

TTATGGGGAAAATATTTGACGGGACTTATCAAATAAAGATAGGAAAAGAA

GAAAACCCAAATATTATAGGCAGAAATGCTAAAGGTTTTAAAATATGTCA

GGATTGGAAGAAGGCATGGATAAAGAACAAAGTTCAGTTAGGAAAGAGAA

ACACAGAAGGAAGAGACACAATAAAAGTCATTATGTATTCTGTGAGAAGT

CAGTAAGATTTGTTGGAAGTGGGTTGGTTTGTTGTATGGTATGTATTTTA

GCAATAATCTTTATGGCAGAGAAAGCTAAAATCCTTTAGCTTGCGTGAAT

GATCACTTGCTGAATTCTTCGAGGTAGGCATGATGAAGGAGGGTTTAGAG

GAGACACAGACACAATGAACTGACCTAGATAGAAAGCCTTAGTATACTCA

GCTAGGAATAGTGATTCTGAGGACACACTGTGACATGATTATGCCATTAC

ATGTATGGTAGTGATGGGGATGATAGAAGGAAGAACTTATGGCATATTTT

CACCCCCCCAAAAATCAGTTAAATATTGGGACACTAACCATCCAGGTCTA

GAAAAGTCACATGCCATAGCCATGGTATTGCACATCATTCATCTTGCATT

CTTTGAGAATAAGAAGATCAGTAAATAGTTCAGAAGTGGGAAGCTTTGTC

CAGGCCTGTGTGTGAACCCAATATTTTGTTTAGAAATAGAACAAGTAAGT

TCATTGCTATAGCATAACACAAAATTTGCATAAGTGGTGGTCAGCAAATC

CTTGAATGCTGCTTAATGTGAGAGGTTGGTAAAATCCTTTGTGCAACACT

CTAACTCCCTGAATGTTTTGCTGTGCTGGGACCTGTGCATGCCAGGCAAG

GCCAAGATGGCTCAAAGAGCAACCAGCCACCTCTGCAGCCTGCCACCTCC

TGCTGGCAAGATTTGTTTTTGCATCCTGTGAAGAGCCAAGGAGGCACCAG

GGCATAAGTCTACTCACTTATATCTGTCTGGAACATAACGCTTGTTTGTT

TTTACAACAAATAAAATTGATCTTGAATAAAAACTGAG

>hg19_ct_ARAlincRNAs_9727_ARAlincRNA_0310.4 range=chr2:130783566-130808704 5'pad=0 3'pad=0 strand=- repeatMasking=none

GCGGTTGTGGCTCAGGGGTCAGCTCCTGCTAGTGCCAGGACACTACTGGG

AGGCTGGGACCCGACCAAAGCCCATGGTGTTTCTGGCCTGAGAACAAGGT

GTCTTGGGACCATAAGGCCAGGCCACCAATGGCCATTGGGTCATAGGGGC

TCAGCCCCAATCTTTGTCTTTCCCTGGCTCCTTCTGATTCAGTCCCATCA

GGGCCCTGGATCCCAAGACTCAGCATCCAAGGTCCCCTCCAGGAATCCTG

GCAGCTCAGCATACTTTATCCTGTTTCATCTGAGAGCAAAAATGTAAAAT

TGGATGCACAGAAAAGTGACTCAAAGTGCTTAATGACTAGAAGAAATCTA

GGAGCAGCAAGAAGGTAATGTGGAGGGAGGGACCTCCATGACCGGTGTCT

GCAGAGCCAGGGGTACAGGCACCCAGTGCTGTGGCCTGGCACCACCTGCC

TCTCAGAGGGTGGGTGGCACACTCCTTAACCAGAGGACAGCAGGCCTGGT

CACCAGCTTTTCTACCTGTCCCTGTAAGCATCACATTGCTGGAGGAAAAT

CTCATGCCAGAGCTTGGACCATCCCTAGCTCGGGTTAGGGGTTGTCCCTT

GGTGACCTAAATGAAAAAACAGGTCCAGAACAGAGTTCCTGATGCTGGAC

ACTCATTCAGTCTTTGAATCGTGGGAGGGGAGGCCTGGTACTAGGTAGAC

CTAACCTCTTTGAGGAACCACAGAGCCCAAGGCTGGAAACCTCCAGAATC

CTCCACCCCCTGATCCTCCCTGGGGACCCCTGTGGCCTGTCTCACTGAGC

ACTCTTCCATCTGTAGATGTCTGGGCTGCTGTACAAGGGAGTCCCCTTTC

AGGTGTGGTGCTAGACATGGTCACTCCTGCTGGATGTCTAGGTGGTAGAA

ACCAAGGACCTAGGGAAATACCAGCTACAGCCTTTCCCCGCTCATCCAGA

GCAGGACAAACAGGCCAGGCGGTGTCAGGAGCCCAGGTCTCCAGCTGGAG

GGAACGTCAACCCTGCGGTGGGAGTAGGGGCCCTTTGCACATCCTAGGCA

CAGATGGTAATGTAGACACCACAGGTAAGCTGGGCTTGGTACCTACCCCT

CCCCGGATTCAGAAAGAAACCAAACAAGGAGCTTTGTGCGGAATGAAACC

TCCTTTCCTCCCAGAAGCACTGCTGACTGTTTGGTGGTTGCCATTTGTGG

CAGTGAGCCTTTGTTTGTTCTGAGGTTGGGCTGGTTTCTCCTCTTGGTCC

TGCCCTACAGATCATAAAGGAGAACAGCAAGAGGTCCCCAGCAAACATCC

ACAGATGGCCTTGGAACCTCACCCTGCAGGAATGCCAGTGAACATACTGC

TGACATCTTGGAGCTCAGTACCCTCATAGTGTAACGGCGTCAGTAGATCT

GCCTGTGCTTGGACTTCCTGTACTACCCATTCCTGAGGGGCGATGCTTCT

GCAGGGCCTGTGACTTGGTGCACAACTTCAGACACCATCATCTTGCAGCA

GCACCGCACCCTCACTAGCCAGGGTGTTGATGACTTCCTCAAGGCCAAGG

CCACATTCAAGGCTTCGGACTTTATTGATGCGCTTGTGCTGAGCAAGGTG

GCTTCTCCAGGATCTTAATTCAGGAGGTAGAATGGAGCTTGAGATCAAGT

GTCTGATCAAGCCTCAGTGTATGGGCGCTGTTCATCCTCTGGTGCTGAAG

CAGCCAAGAGACCCAAGTCTGCCTGGCTGCCTCTTAGGATATGACAGCAG

AGCCAGTGGCCTCTACTAGATCCTGTACAACCTCACAAAACACCCAGACA

TCGGGAGTGCTGCCAGCCTGTGATGCAAGAGTCCTAATCCTGAAGACATT

GAATGACCTGTCATTCTGCTGTTTTTACCAAAAAGGATCATGAGGATCAG

AGAGGAAAAGTCACTTGCCCAAAGTCACACAGCTGAACAGTGGTGGAGTT

CAACTTTGACCATGGGCTGTCTGGCCCCAAGGTGTATGCTTGCTTCTCTC

CCAAGAGACTCCTTTCTTATCAGGCTCAAATGAATGAAAGGAGGATGTTA

AAGTTCCTAGAAGCTTTAATTGAATGAAAGTTCCTAGTAGATCTGTACCT

ACTAAAAACCACACTTCTGAAGCTACGTGGCCACCAGAAGACACAGCTAG

TCTGCCATGTAAAAAAGGAAAGGTGGCGTGTGCCCTGAAGGCGCAGGGGT

GAGAGGCAGGGAAATGGAGACCCCAACAGCCAGCATCAGTGGCCCTCATC

ACAGCCCTCCAGGAGATATCAAAGGAGACAACGCCATTATTGACGAGATC

ACTCCCAAGCGGATTGGAGATTGTCCCAATACTTAGACCTATAGCAAGGC

CTTGGGAGAAATGGTGGTGCAGCAGGAGAGCAGGAACCTAACCATTGCCA

TCCTAAGGCCCTCCATTGTGCGGAGCAACGTCGCACCAGCTTTTCCTGGG

TTGGGTTGATAATCTAAATGGATGTAGCTGACTCATTATTGCGGGCAAAG

GAGATAGAAGTGGATGAAATGAGAGAATTTTCTTTAATTGAACTCTGGTT

AATGCCAAAAGTGTTCAATCACTATGTGTGGGGAAGTTTCCTGGTACAAA

GGAAAAAAAAACAGATTTGGCTGTGTTAGTATGTGGTAAACAGAATTAAA

TTTTTTTATGTGAACTCGTACATATGTGCTTCATAATATGTCACTCCTAT

TTAACATCTCTTTCACACACACACATCCCCCCACCTCCGCACACACCCTT

TTGCTAGTCTTCTTCCATTTTGCTTATTAACCCAAAGAAAGAAAAAAATA

CAGAAGCCTTCTTGCATAGGTTGCTTTAATCAGATGAATTATTGAAGCCA

CTCTGTCTAAATATTATTTTCCCTTATTTTTCTTTCATTAGTTTTTCATT

CAGTTGTATTTATTTAATTTTTATTTCATTTTCCAGACCTAAGTCAGTGT

TAGTCTACCACTGTACATCTGGTAACCTCAATCCCTGCAACCGGGGCAAA

ATGGCTACCGAAGGAAGATACCCTCATCAACCCAATTTTACAAATGGAGA

AATAGAAGTTAAGGGAAGAATCTGAAGTAGTCTCAAAGGCAGTGACAGGA

AGGATGTGGAGAAAGCTGAGTGTCAAAGTCAGTATTCAGGACCGGCTTTA

CTGCTACTTAGAGATGAATGAAGAAATCAGAGGGAACGCAGTGTGCTGAT

GCTAAAGCAGCTGTCACCACCCAGCTGTGTGACATAGGACATATTCTTTC

TCTGTCTCACTTGACTAATATGATATGTCAGAGGAGACATGATTGTAATT

GCCTAAAGCAATTCTTGTGATCAAGACTCAGAAGCACGAACAGTATTGCC

CTCTGTGTTAGCCCCTTTATAAGGGAGGATATCATCTTCAGCATGCTGAA

TTGTCATCTTTCTTAGCAGTGCAAATGACTAAAACTTAGCCAATGTAGAG

TTTGTCCAAATTTGGAGCTCATAACTCAGTTCTTGAGCAAAGTGAAAAGA

AAACATTGTGATTATGGGGAAAATATTTGACGGGACTTATCAAATAAAGA

TAGGAAAAGAAGAAAACCCAAATATTATAGGCAGAAATGCTAAAGGTTTT

AAAATATGTCAGGATTGGAAGAAGGCATGGATAAAGAACAAAGTTCAGTT

AGGAAAGAGAAACACAGAAGGAAGAGACACAATAAAAGTCATTATGTATT

CTGTGAGAAGTCAGTAAGATTTGTTGGAAGTGGGTTGGTTTGTTGTATGG

TATGTATTTTAGCAATAATCTTTATGGCAGAGAAAGCTAAAATCCTTTAG

CTTGCGTGAATGATCACTTGCTGAATTCTTCGAGGTAGGCATGATGAAGG

AGGGTTTAGAGGAGACACAGACACAATGAACTGACCTAGATAGAAAGCCT

TAGTATACTCAGCTAGGAATAGTGATTCTGAGGACACACTGTGACATGAT

TATGCCATTACATGTATGGTAGTGATGGGGATGATAGAAGGAAGAACTTA

TGGCATATTTTCACCCCCCCAAAAATCAGTTAAATATTGGGACACTAACC

ATCCAGGTCTAGAAAAGTCACATGCCATAGCCATGGTATTGCACATCATT

CATCTTGCATTCTTTGAGAATAAGAAGATCAGTAAATAGTTCAGAAGTGG

GAAGCTTTGTCCAGGCCTGTGTGTGAACCCAATATTTTGTTTAGAAATAG

AACAAGTAAGTTCATTGCTATAGCATAACACAAAATTTGCATAAGTGGTG

GTCAGCAAATCCTTGAATGCTGCTTAATGTGAGAGGTTGGTAAAATCCTT

TGTGCAACACTCTAACTCCCTGAATGTTTTGCTGTGCTGGGACCTGTGCA

TGCCAGGCAAGGCCAAGATGGCTCAAAGAGCAACCAGCCACCTCTGCAGC

CTGCCACCTCCTGCTGGCAAGATTTGTTTTTGCATCCTGTGAAGAGCCAA

GGAGGCACCAGGGCATAAGTCTACTCACTTATATCTGTCTGGAACATAAC

GCTTGTTTGTTTTTACAACAAATAAAATTGATCTTGAATAAAAACTGAG

>hg19_ct_ARAlincRNAs_9727_ARAlincRNA_0310.5 range=chr2:130783566-130808704 5'pad=0 3'pad=0 strand=- repeatMasking=none

GCGGTTGTGGCTCAGGGGTCAGCTCCTGCTAGTGCCAGGACACTACTGGG

AGGCTGGGACCCGACCAAAGCCCATGGTGTTTCTGGCCTGAGAACAAGGT

GTCTTGGGACCATAAGGCCAGGCCACCAATGGCCATTGGGTCATAGGGGC

TCAGCCCCAATCTTTGTCTTTCCCTGGCTCCTTCTGATTCAGTCCCATCA

GGGCCCTGGATCCCAAGACTCAGCATCCAAGGTCCCCTCCAGGAATCCTG

GCAGCTCAGCATACTTTATCCTGTTTCATCTGAGAGCAAAAATGTAAAAT

TGGATGCACAGAAAAGTGACTCAAAGTGCTTAATGACTAGAAGAAATCTA

GGAGCAGCAAGAAGGTAATGTGGAGGGAGGGACCTCCATGACCGGTGTCT

GCAGAGCCAGGGGTACAGGCACCCAGTGCTGTGGCCTGGCACCACCTGCC

TCTCAGAGGGTGGGTGGCACACTCCTTAACCAGAGGACAGCAGGCCTGGT

CACCAGCTTTTCTACCTGTCCCTGTAAGCATCACATTGCTGGAGGAAAAT

CTCATGCCAGAGCTTGGACCATCCCTAGCTCGGGTTAGGGGTTGTCCCTT

GGTGACCTAAATGAAAAAACAGGTCCAGAACAGAGTTCCTGATGCTGGAC

ACTCATTCAGTCTTTGAATCGTGGGAGGGGAGGCCTGGTACTAGGTAGAC

CTAACCTCTTTGAGGAACCACAGAGCCCAAGGCTGGAAACCTCCAGAATC

CTCCACCCCCTGATCCTCCCTGGGGACCCCTGTGGCCTGTCTCACTGAGC

ACTCTTCCATCTGTAGATGTCTGGGCTGCTGTACAAGGGAGTCCCCTTTC

AGGTGTGGTGCTAGACATGGTCACTCCTGCTGGATGTCTAGGTGGTAGAA

ACCAAGGACCTAGGGAAATACCAGCTACAGCCTTTCCCCGCTCATCCAGA

GCAGGACAAACAGGCCAGGCGGTGTCAGGAGCCCAGGTCTCCAGCTGGAG

GGAACGTCAACCCTGCGGTGGGAGTAGGGGCCCTTTGCACATCCTAGGCA

CAGATGGTAATGTAGACACCACAGGTAAGCTGGGCTTGGTACCTACCCCT

CCCCGGATTCAGAAAGAAACCAAACAAGGAGCTTTGTGCGGAATGAAACC

TCCTTTCCTCCCAGAAGCACTGCTGACTGTTTGGTGGTTGCCATTTGTGG
[truncated: 2,117,881 more chars]
