# Supplementary material for: Chromatin Landscape Distinguishes the Genomic Loci of Hundreds of Androgen-Receptor-Associated LincRNAs From the Loci of Non-associated LincRNAs
Source: Front Genet. 2018 Apr 25;9:132. doi: 10.3389/fgene.2018.00132 (PMC5985396; doi:10.3389/fgene.2018.00132)
Supplement: Supplementary file 11 [file Data_Sheet_1.pdf]

Supplementary Figures for

Chromatin landscape distinguishes the genomic loci of hundreds of androgen-receptor-associated lincRNAs from the loci of non-associated lincRNAs

Lucas F. daSilva[1,2†], Felipe C. Beckedorff[1,2†], Ana C. Ayupe[1],

Murilo S. Amaral[1,2], Vinícius Mesel[2], Alexandre Videira[1,2], Eduardo M. Reis[1],

João C. Setubal[1,3] and Sergio Verjovski-Almeida[1,2*]

[1]Departamento de Bioquímica, Instituto de Química, Universidade de São Paulo, 05508-900 São Paulo, SP, Brasil.

[2]Laboratório de Expressão Gênica em Eucariotos, Instituto Butantan, 05503-900 São Paulo, SP, Brasil

[3]Biocomplexity Institute of Virginia Tech, Blacksburg, VA, USA

[†]These authors have contributed equally to this work.

*Corresponding author E-mail: verjo@iq.usp.br

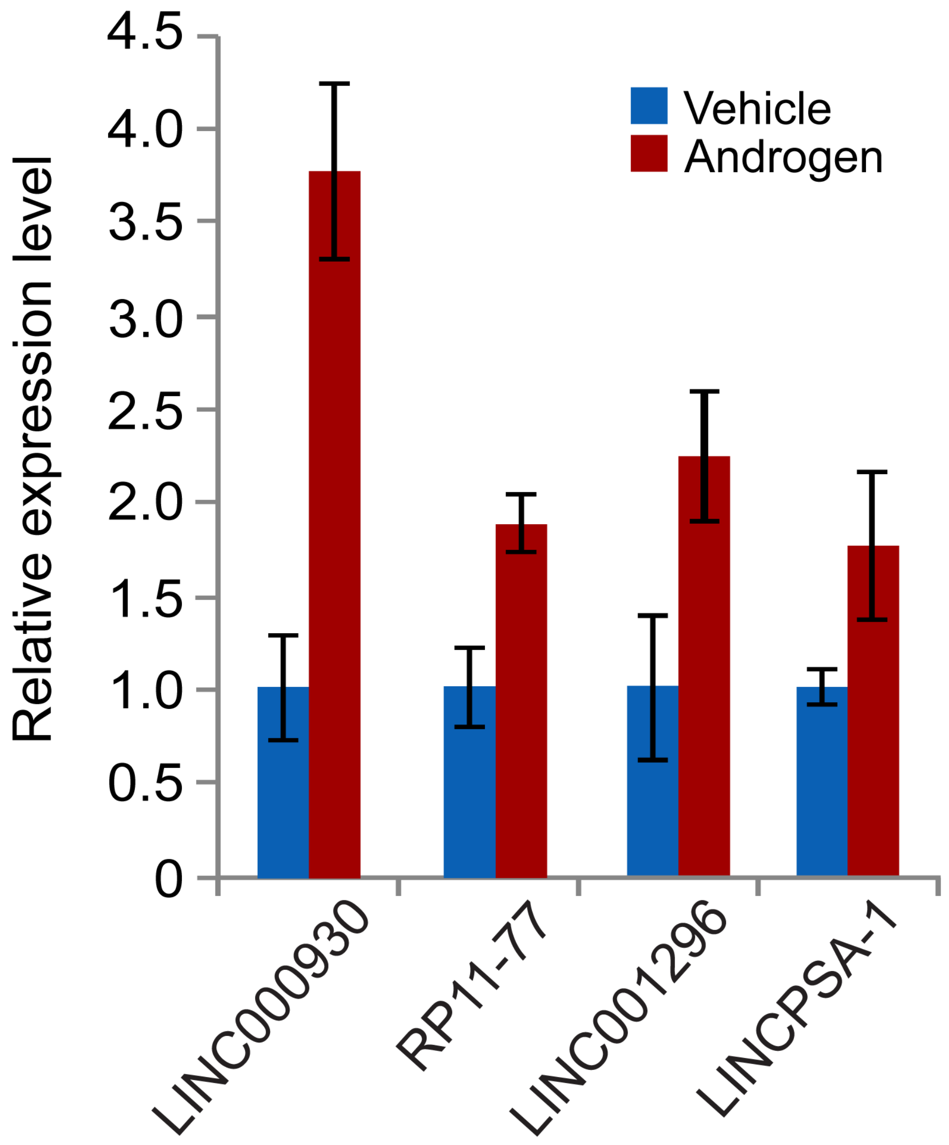

Fig. S1 - Validation by RT-qPCR of the lincRNAs expression activation induced by androgen in LNCaP cells. LNCaP cells grown in RPMI 1640 with 10% (w/v) charcoal-stripped FBS were treated for 24 h with 1 nM R1881 or with vehicle (ethanol), the RNA was extracted, the expression of the indicated genes was measured by RT-qPCR and the expression level in the presence of androgen is shown relative to the level in the presence of vehicle.

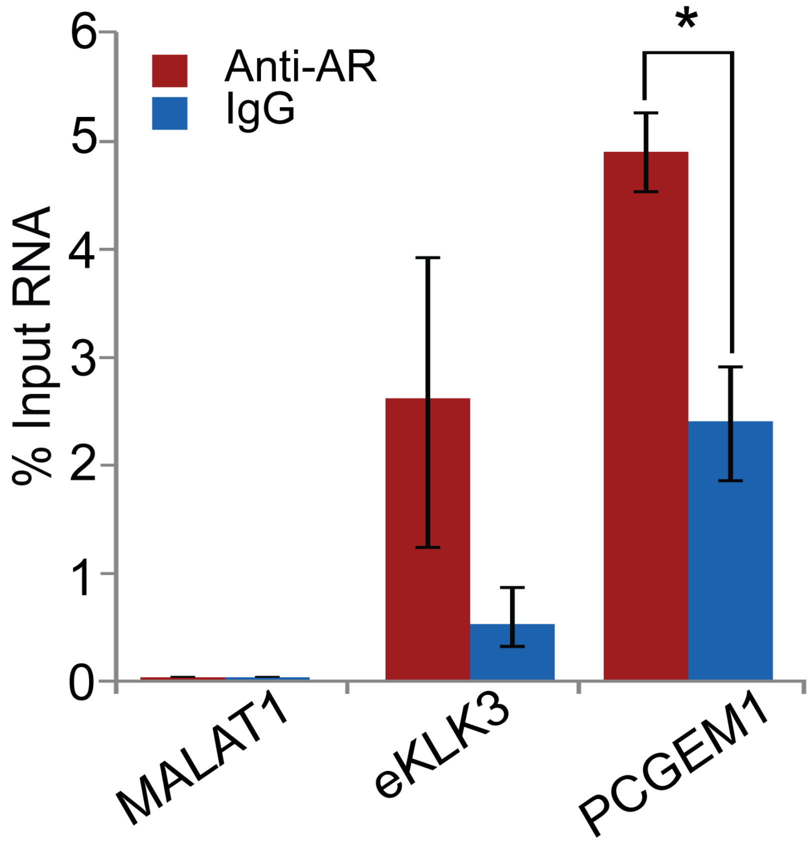

Fig. S2 – Validation of RNA IP by RT-qPCR. The endogenous levels of lincRNAs that were co-immunoprecipitated with AR (red bars) were measured by RT-qPCR in LNCaP cells treated for 6 h with 0.1 nM androgen, and the results are shown as % input. Non-specific RNA co-immunoprecipitated with control IgG from non-immunized rabbit was included as a negative control (blue bars). The levels of AR-associated *eKLK3* and *PCGEM1* lincRNAs, known to bind to AR were measured as positive controls, whereas the level of *MALAT1* lincRNA, not expected to bind to AR, was measured as a negative control. (*) = $p < 0.05$, t-test.

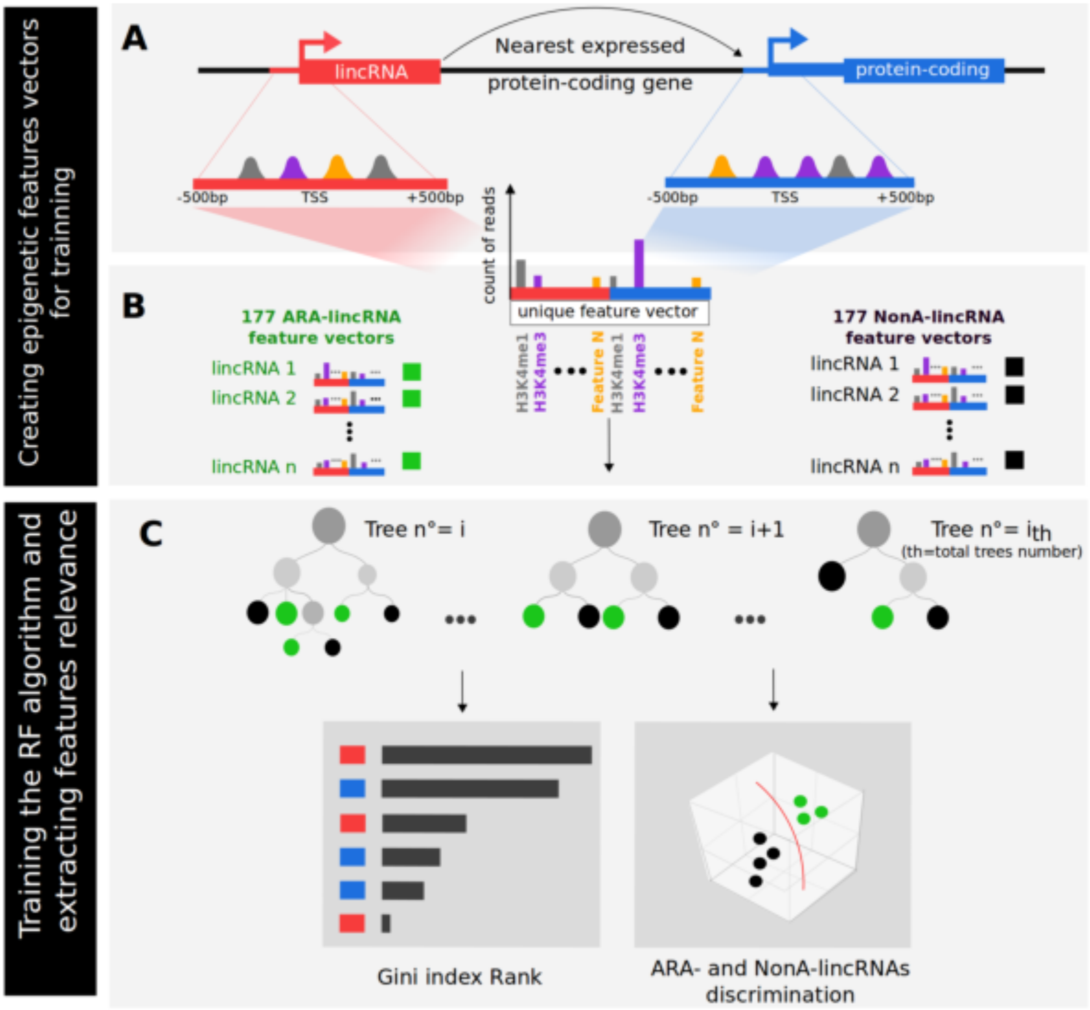

Fig. S3 - Schematic computational pipeline for identifying the epigenetic signatures at the TSS of lincRNAs and of protein-coding gene neighbors. (**A**) Step 1 involved mapping to the genome of the lincRNAs that were obtained in the RIP-seq experiment (red) (both ARA-lincRNAs and NonA-lincRNAs), assigning of a neighbor protein-coding gene (blue) to each lincRNA (ARA- or NonA-lincRNA), and counting the abundance of ChIP-seq/DNAse-Seq reads for all epigenetic marks (yellow, purple and gray peaks) that were found at the TSS of each pair of lincRNA-protein-coding gene using a +/- 500 bp window. (**B**) In Step 2 the computed abundance of the different epigenetic marks were combined and converted into a unique feature vector for each lincRNA and its protein-coding gene neighbor. (**C**) Step 3 involved machine learning and extraction of epigenetic marks that better separate ARA- from NonA-lincRNAs through the Random Forest algorithm.

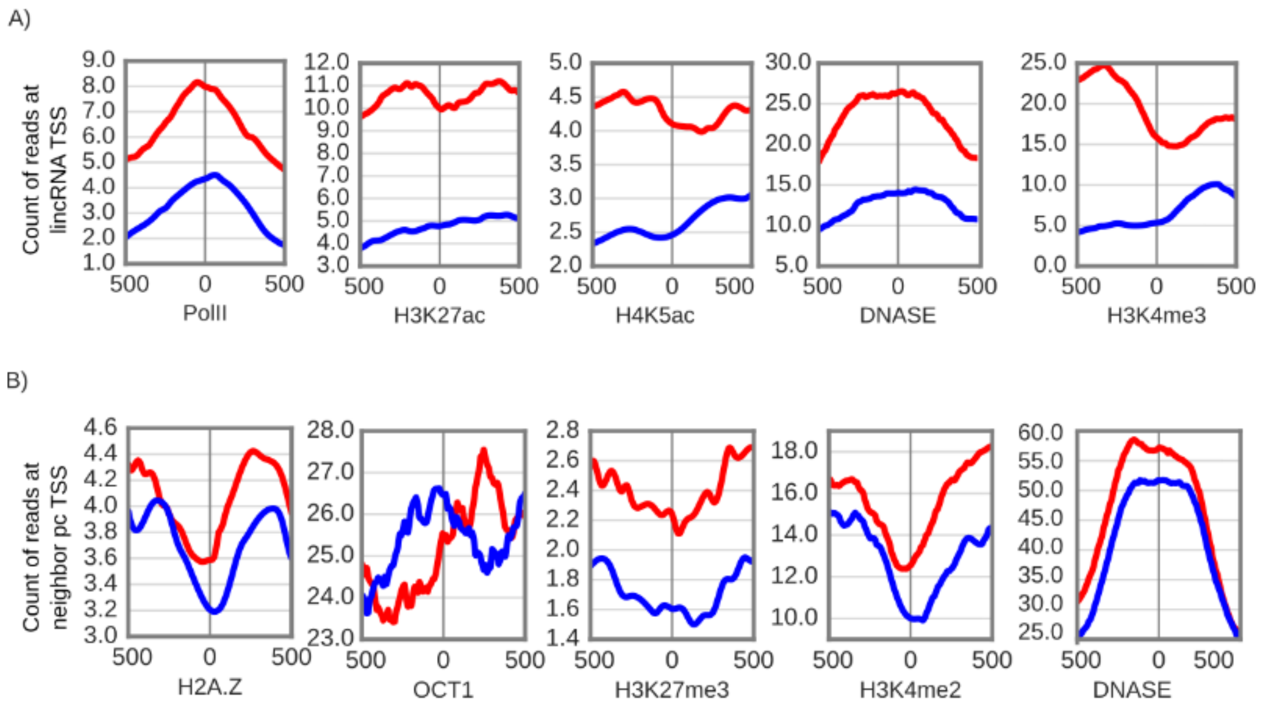

Fig. S4 - Distribution of epigenetic marks around the TSS of lincRNAs and of their neighbor protein coding genes. Epigenetic marks shown are the top five most relevant marks of Fig. 3A. A total of 177 ARA-lincRNAs with androgen-activated neighbor protein-coding genes, as well as a control set of 177 Non-A-lincRNAs were analyzed. (**A**) Density distribution of ChIP-Seq read counts of the indicated epigenetic mark around the TSS (+/- 500 bp) of ARA-lincRNAs (red) and of NonA-lincRNAs (blue). (**B**) Density distribution of ChIP-Seq read counts of the indicated epigenetic mark around the TSS (+/- 500 bp) of protein-coding (pc) genes that are neighbor to ARA-lincRNAs (red) and to NonA-lincRNAs (blue).

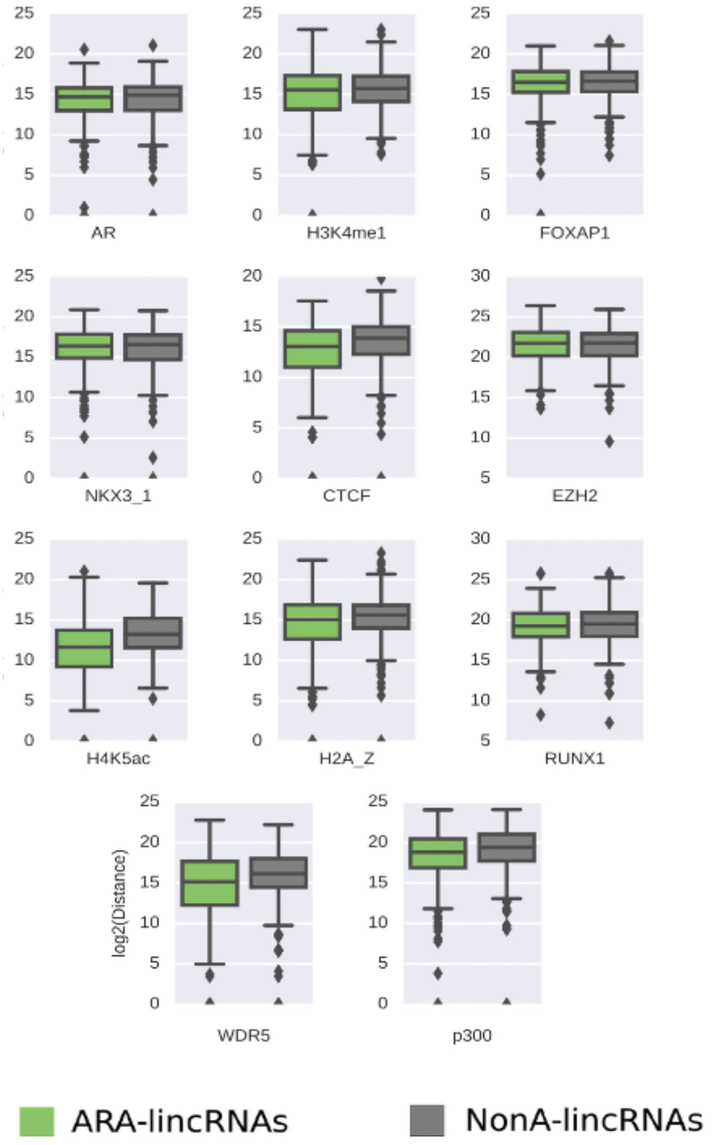

Fig. S5 - Box plot of log2 distance along the genome between the indicated epigenetic mark and the TSS of lincRNAs in LNCaP cells. Log2 (distance) in bp between the genomic locations of the indicated epigenetic mark and of the nearest TSS for either the ARA-lincRNAs (green) or the NonA-lincRNAs (gray) expressed in LNCaP cells. A statistical t-test was applied and showed that the average distances were not significantly different between the two groups of lincRNAs, for each of the epigenetic marks shown.
